# An Alternative HIV-1 Non-Nucleoside Reverse Transcriptase Inhibition Mechanism: Targeting the p51 Subunit

**DOI:** 10.3390/molecules25245902

**Published:** 2020-12-13

**Authors:** Kwok-Fong Chan, Chinh Tran-To Su, Alexander Krah, Ser-Xian Phua, Joshua Yi Yeo, Wei-Li Ling, Peter J. Bond, Samuel Ken-En Gan

**Affiliations:** 1 Bioinformatics Institute, A*STAR, 30 Biopolis Street, #07-01 Matrix, Singapore 138671, Singapore; chankf@bii.a-star.edu.sg (K.-F.C.); chinhsutranto@bii.a-star.edu.sg (C.T.-T.S.); kraha@bii.a-star.edu.sg (A.K.); bii-apdlab@bii.a-star.edu.sg (S.-X.P.); yeoyj@bii.a-star.edu.sg (J.Y.Y.); lingwl@bii.a-star.edu.sg (W.-L.L.); peterjb@bii.a-star.edu.sg (P.J.B.); 2 Experimental Drug Development Centre, A*STAR, 10 Biopolis Road Chromos #05-01, Singapore 138670, Singapore; 3 p53 Laboratory, A*STAR, 8A Biomedical Grove, #06-04/05 Neuros/Immunos, Singapore 138648, Singapore

**Keywords:** HIV, novel p51 drug target, NNRTIs, drug resistance

## Abstract

The ongoing development of drug resistance in HIV continues to push for the need of alternative drug targets in inhibiting HIV. One such target is the Reverse transcriptase (RT) enzyme which is unique and critical in the viral life cycle—a rational target that is likely to have less off-target effects in humans. Serendipitously, we found two chemical scaffolds from the National Cancer Institute (NCI) Diversity Set V that inhibited HIV-1 RT catalytic activity. Computational structural analyses and subsequent experimental testing demonstrated that one of the two chemical scaffolds binds to a novel location in the HIV-1 RT p51 subunit, interacting with residue Y183, which has no known association with previously reported drug resistance. This finding supports the possibility of a novel druggable site on p51 for a new class of non-nucleoside RT inhibitors that may inhibit HIV-1 RT allosterically. Although inhibitory activity was shown experimentally to only be in the micromolar range, the scaffolds serve as a proof-of-concept of targeting the HIV RT p51 subunit, with the possibility of medical chemistry methods being applied to improve inhibitory activity towards more effective drugs.

## 1. Introduction

Human immunodeficiency virus (HIV) remains as one of the major global pandemics, with millions infected worldwide [1]. Current therapies, including pre-exposure prophylaxis (PrEP) for infection prevention [2] and cocktail antiretroviral therapy (ART) for treatment, target the various stages of the HIV life cycle [3]. This encompasses protease inhibitors (PIs) to interfere with protease binding to its substrate gag and gag-pol during viral maturation [4]; reverse transcriptase inhibitors (RTIs) to inhibit viral DNA production [5,6]; integrase inhibitors to block viral DNA insertion into the host genome [7]; fusion inhibitors [8]; chemokine receptor antagonists [9]; and attachment inhibitors [10].

While integrase inhibitors are recommended by the World Health Organization (WHO) [11] as a first line treatment option, there are safety concerns for pregnant women. On the other hand, the RTI drug class, comprising of both nucleoside reverse transcriptase inhibitors (NRTIs) and non-nucleoside reverse transcriptase inhibitors (NNRTIs), is safer and more affordable. RTIs target the reverse transcriptase (RT) enzyme that is unique to viruses, making it a safer target and more suitable than integrase inhibitors for children, pregnant women, or individuals treated for tuberculosis with rifampicin. Recently, a two-drug switch regimen [12] that involved a combination of NNRTI (rilpivirine) and PI (cobicistat-boosted darunavir [12]) was shown effective, keeping RTIs as a recommended component [11] in the standard triple-inhibitor ART.

Both NRTI and NNRTI are used in ART, each inhibiting via different modus operandi. NRTIs are nucleotide analogues and bind to the nucleotide binding site to competitively block reverse transcription [13], leading to the termination of the DNA elongation process [14]. In contrast, NNRTIs bind to a site located near the polymerase active site, disrupting the structural alignment of the deoxyribonucleotide triphosphates (dNTPs) and template/primer substrates at the RT “primer grip”; thus, inducing effects on the polymerase active site indirectly and non-competitively [15] to inhibit reverse transcription [16]. Unlike NRTIs, which mimic nucleotides to directly interfere with cellular replication machinery [17], NNRTIs can vary in structure, generally do not exhibit off-target effects, and have been found to non-competitively induce conformational changes in the RT structure [18]. Therefore, NNRTI drug resistant mutations typically occur at the less conserved drug-binding pocket [19].

HIV-1 RT is a heterodimer of the p66 and p51 subunits. The larger p66 subunit contains five subdomains: fingers, palm, thumb, connection, and RNase H. Despite having an identical sequence to that of p66, excluding the RNase H subdomain, the p51 subunit forms a more compact structure [20]. However, efforts to target hot spots on the p51 domain [21,22] were not clinically successful with no known inhibitors to p51 reported to date.

In our search for alternative NNRTIs against HIV-1 RT, we serendipitously found two inhibitory chemical scaffolds that exhibited RT inhibition. While inhibiting only at micromolar range, one of the compounds elicited the possibility of non-canonical targeting at the RT p51 subunit. We therefore attempted to investigate the underlying mechanism for their interactions on the RT using computational docking and molecular dynamic (MD) simulations, finding a possible alternative HIV-1 RT inhibition mechanism via the RT p51 subunit.

## 2. Results

### 2.1. Two Compound Scaffolds that Inhibit HIV-1 RT Activity

**Figure 1:**
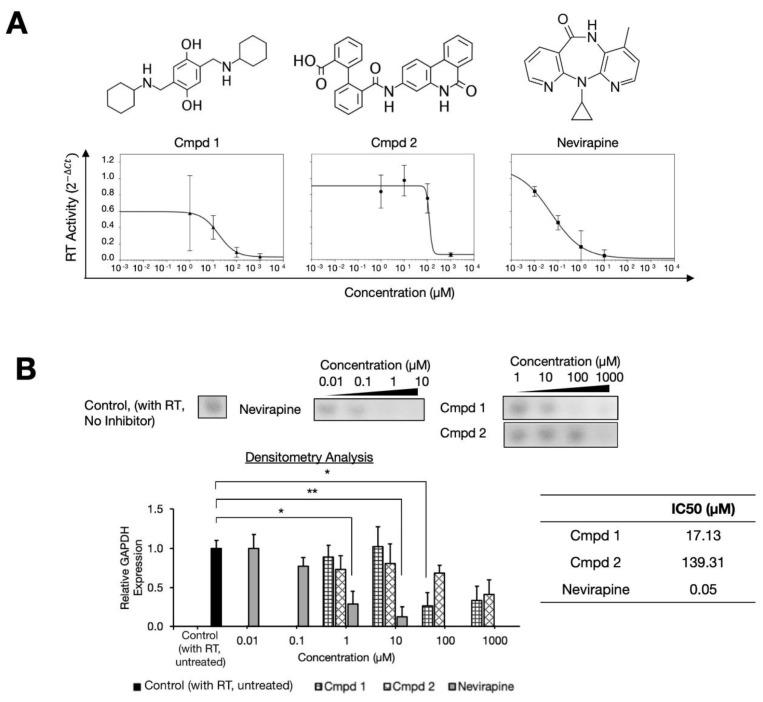
The two compounds that inhibited HIV-1 reverse transcriptase (RT) function. (**A**) RT inhibition was demonstrated via the best-fit curves (using ΔCt) of compound 1 (NSC48443), compound 2 (NSC127133), and nevirapine (as a positive control). Chemical structures of the compounds are shown on the top. (**B**) Densitometry analysis is shown with various concentrations of compounds 1 and 2 (1 µM to 1000 µM), nevirapine (0.01 µM to 10 µM), and the control. Asterisks depict *p*-values of statistical tests of the mean difference against the untreated control (with RT and no inhibitors added), *p <* 0.05 (*) and *p <* 0.01 (**). All of the experiments were performed in triplicates. The densitometric values were estimated and normalized against those of the control, using Fiji software [26].

From 40 compounds of the National Cancer Institute Developmental Therapeutic Program’s (NCI/DTP) Diversity Set V, we serendipitously identified two chemical compounds, NSC48443 and NSC127133 (referred to as compounds 1 and 2, respectively) that inhibited HIV-1 RT activity using the reverse transcriptase—quantitative polymerase chain reaction (RT-qPCR) assay (Supplementary Figure S1 and Figure 1). In these assays, the glyceraldehyde 3-phosphate dehydrogenase (GAPDH) housekeeping gene was used for cDNA synthesis control, given its constitutive expression [23]. A known NNRTI, nevirapine, was used as a positive control. Compared to nevirapine inhibiting at half maximal inhibitory concentration (IC_50_) at 50 nM in our assay, and in agreement to previous reports [24,25], the two compounds inhibited the RT activity at micromolar concentrations of IC_50_ at 17.13 µM and 139.31 µM, respectively (Figure 1B).

### 2.2. RT Binding Sites of the Two Compounds

**Figure 2:**
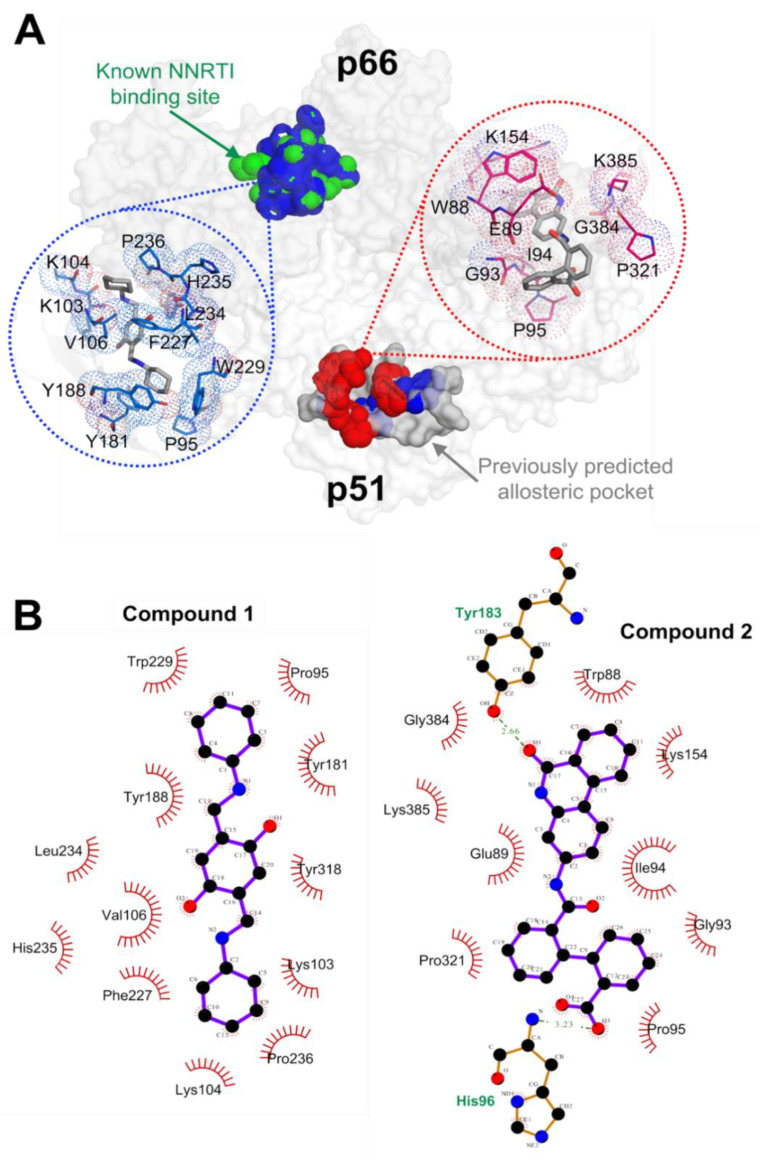
Structural analyses of HIV-1 RT interaction with the two inhibitory compounds. (**A**) Binding sites of compound 1 (blue surface and lines) and compound 2 (red surface and lines) on HIV-1 RT. One of the two binding sites for compound 1 overlaps with the known non-nucleoside reverse transcriptase inhibitor (NNRTI) binding pocket (green, on p66), whereas the other overlaps with our previously predicted allosteric pocket [28] (grey surface on p51), at which compound 2 was also found to bind (red). The two chemical compounds are presented in grey sticks within the binding pockets. (**B**) Two-dimensional (2D) presentation of the interactions between the compounds and their binding sites, constructed using LigPlot+ [29].

To identify the binding sites of compounds 1 and 2 on the HIV-1 RT heterodimer structure (including p66 and p51 subunits), blind docking experiments were performed using AutoDock Vina [27], followed by MD simulations with various initial bound conformations of each compound as inputs (Supplementary Figure S2). Compound 1 bound to two different sites on the HIV-1 RT (blue in Figure 2A) with one site on the p66 subunit close to a loop located on p51 comprising of residues S134 to P140, and another site on the p51 subunit, whereas compound 2 bound only to one site on the p51 subunit (red in Figure 2A). The interacting residues of the binding sites on p66 (to compound 1) and on p51 (to compound 2) are shown in Figure 2B.

**Figure 3:**
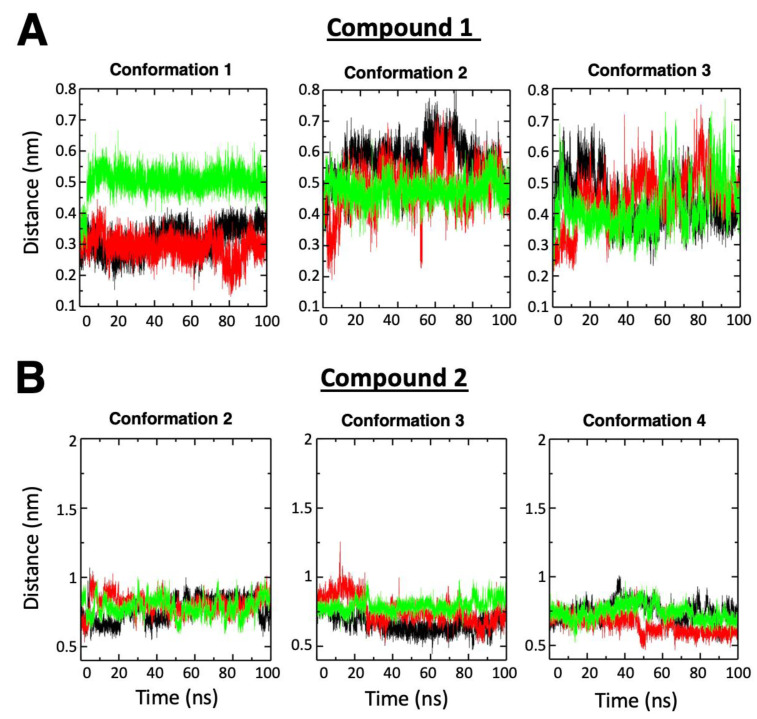
The center-of-mass distances between ligand and the respective binding site on RT during the molecular dynamic (MD) simulations. Data are shown for (**A**) compound 1 and (**B**) compound 2, calculated using independent triplicates (black, green, and red) of 100 ns trajectories. For compound 2, only the results of the three setups that exhibited stable ligand binding (i.e., conformation 2, 3, and 4) are shown. The remaining data are shown in Supplementary Figure S3.

Compound 1 occupied the site on p66 with more favorable predicted binding energies than the binding site on p51 (Supplementary Table S2), and with a more stable bound conformational state at p66 than at the p51 site (Figure 3A, showing the more stable center-of-mass distances between the binding site and the compound during simulations). Given that more hydrophobic contacts (but fewer hydrogen bonds) were detected between compound 1 and its p66 binding site than the p51 binding site (Supplementary Table S2), hydrophobic interactions are the dominant binding force for association with p66. Hydrophobic contacts were observed among L100, K103, V106, Y181, Y188, P225, F227, L234, P236, and Y318, along with less prominent interactions among P95, S105, and W229. Unstable/weak hydrogen bonds were detected between compound 1 and residue K101 (Supplementary Figure S3).

On the other hand, compound 2 maintained stable binding to the p51 subunit in three out of six successful setups for the MD simulations—each setup had a unique initial compound 2 conformation (Figure 2 and Supplementary Figure S2). In the three stable conformations, compound 2 exhibited low binding energies to the same site on p51, with the lowest in conformation 3 (Supplementary Table S3); thus, the binding site for conformation 3 was deemed as the most likely binding mode. Compared to compound 1 when binding to the p66 subunit, the binding site of compound 2 on p51 is more solvated and polarized due to several polar/charged nearby residues, such as H96, K154, and K385. Consistent trends in energy contributions (with increasing interior dielectric constants, shown in Supplementary Table S3) showed that the binding may be dominated by electrostatic interactions. Y183 established strong hydrogen bonds with compound 2, whereas others (W88, E89, G93, I94, P95, K154, P157, and K183) made hydrophobic contacts with compound 2 (Supplementary Figure S3).

### 2.3. Experimental Inhibition on Separate HIV-1 RT p66 and p51 Subunits

**Figure 4:**
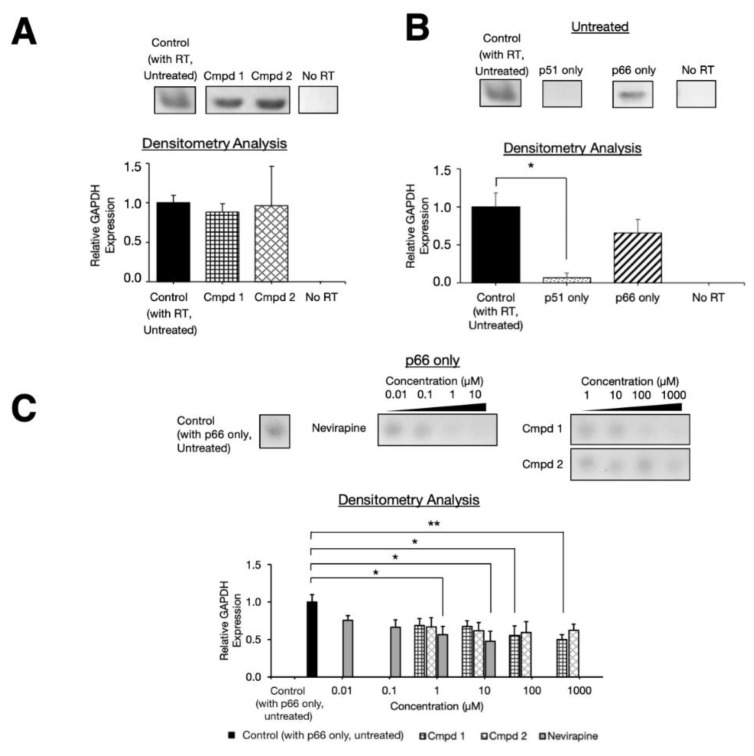
Inhibition analysis of individual RT subunits p66 and p51 by the identified compounds. (**A**) Results of post-cDNA synthesis analysis when treated with the two compounds. The control was performed using DMSO with RT. (**B**) RT-qPCR results of individual RT p51 and p66 subunits with no inhibitors added. (**C**) Densitometry analysis of the various concentrations of compounds 1 and 2 (1 µM to 1000 µM) and nevirapine (0.01 µM to 10 µM) in the p66 subunit alone. Asterisks depict *p*-values of statistical tests of the mean difference against the untreated control (with RT and no inhibitors added), *p <* 0.05 (*) and *p <* 0.01 (**). All of the experiments were performed in triplicates to quantify the GAPDH gene products on 2% agar gels. All of the band sizes were estimated ~120 bp using GelApp [33]. The p51 and p66 only gels were performed on the same gel but separate from each other. The densitometric values were estimated and normalized against those of the control, using the Fiji software [26].

Results of our independent triplicates confirmed that the inhibition did not occur during the GAPDH qPCR step since PCR product bands were still detected when the compounds were added only right after the cDNA synthesis step (Figure 4A). In separate experiments utilizing p51 and p66 subunits separately, the untreated RT-qPCR reactions did not show detectable bands for p51 alone (Figure 4B), agreeing with previous findings [30,31,32] that the p51 subunits alone did not exhibit considerable RT activity.

For the p66-only reactions, PCR product bands with no significant differences (*p* < 0.05) of relative GAPDH expression levels were observed between those expressed in p66 only, and in the whole heterodimer RT (Figure 4B). Proceeding to qPCR assays on the p66-only condition to further quantify the inhibitory activity of the two identified compounds (Figure 4C), we found that compound 1 inhibited p66 at concentrations of 100 µM or higher whereas no significant differences of the relative GAPDH expression were found for compound 2 when compared to the control with no inhibitors. The positive control nevirapine inhibited the RT function via p66 at concentrations higher than 10 µM (Figure 4C). These findings highlighted that compound 1, but not compound 2, inhibited RT via p66 subunits.

## 3. Discussion

In this study, we demonstrated that HIV RT p51 could be targeted for novel RTIs, showing the molecular interactions that can form with such inhibitors by experimentally screening 40 compounds of the NCI Diversity Set V, serendipitously finding two compounds that could inhibit the HIV-1 RT. Using RT-qPCR to estimate the inhibition efficiency with a comparison to known NNRTI nevirapine as the positive control (IC_50_ of 0.05 µM), in agreement with previously established enzyme assays [24,25], the two compounds were found to inhibit the RT activity at micromolar concentrations: compound 1 with IC_50_ at 17.13 µM and compound 2 with IC_50_ at 139.31 µM.

Our initial computational blind docking results identified two potential binding sites (on p66 and p51 subunits) for compound 1 (Figure 2). Structural analyses demonstrated that compound 1 bound energetically favorably to the binding site on p66, which was also supported by our p66 only assay (Figure 4A). Compound 1 was found to bind to the p66 site predominantly via hydrophobic interactions. On the other hand, compound 2 bound to the p51 subunit, driven predominantly by electrostatic interactions with its more polar pocket (Supplementary Table S2).

Computational analyses showed that both compounds interact with the RT p51 subunit (with less pronounced propensity for compound 1 at its second binding site in p51), distant from the polymerase site located on p66 (Figure 2A), suggesting an underlying allosteric mechanism. Interestingly, compound 2 established stable hydrogen bonds with residue Y183 of p51 (Figure 2B). To date, there are no known clinical mutations reported at this residue of p51 in the latest update report [34], nor in a recent selection-free in vitro mutagenesis study [35], suggesting conservation of this residue and the possibility for compound 2 to be used against drug resistant HIV-1 variants.

It should be noted that compound 2 also interacted weakly (e.g., via conformations 5 and 6, as shown in Supplementary Figure S2) with another site on the p51 subunit in a similar binding mode (data not shown) to that of the other dominant site, further supporting that compound 2 would target p51 rather than p66. We found the first binding site on p51 for compound 2 to overlap with our previously predicted allosteric pocket [28]. Since the structural fold of p51 differs from that of p66 (e.g., overall root mean squared deviation (RMSD) ~4.9 Å, excluding the RNase H region), there is support that the main inhibition site of compound 2 may be located on p51.

Previous studies [30,31,32] showed that a single HIV-1 RT subunit p51 monomer or homodimer have severely reduced RT activity levels as compared to those in the p51-p66 heterodimer RT. We observed this decreased activity in our RT-qPCR assay where no bands were visibly detected in the case of RT p51 alone (Figure 4B). This caveat posed a challenge to experimentally test the direct inhibition of the two compounds on the p51 subunit, particularly for compound 2. However, since compound 2 exhibited the RT activity inhibition in the assays using the heterodimer p66-p51 (Figure 1B), but not in p66 homodimer (Figure 4C), we logically conclude that compound 2 inhibited RT activity via the p51 subunit that was also supported by our computational analyses (Figure 4A). Since the DNA polymerase active site is on the p66 subunit, compound 2 may therefore require higher concentrations (IC_50_ 139.31 µM) compared to the direct inhibition of compound 1 on p66 (IC_50_ 17.13 µM).

Despite inhibiting the RT function only at a micromolar range, the two compounds can serve as scaffold for further development, e.g., in fragment-based ligand design [36], given the need of alternative NNRTIs in the increasing emergence of viral drug cross resistance mutations [34]. Our limited mice experiments (unpublished) showed no obvious toxicity effects at dosages up to 7 mg/kg for both the compounds, preliminarily showing them to be relatively safe. Given that RT is unique to viruses, the inhibitory effects of the two compounds (especially compound 1) were assessed as NNRTIs. On the other hand, compound 2 demonstrated the feasibility of inhibiting RT via p51, revealing molecular interactions for a druggable site to guide the modification and development of new class of p51 inhibitors, further validating the novel p51 druggable pocket that we had previously identified computationally [28] that further screening can be performed.

**Figure 5:**
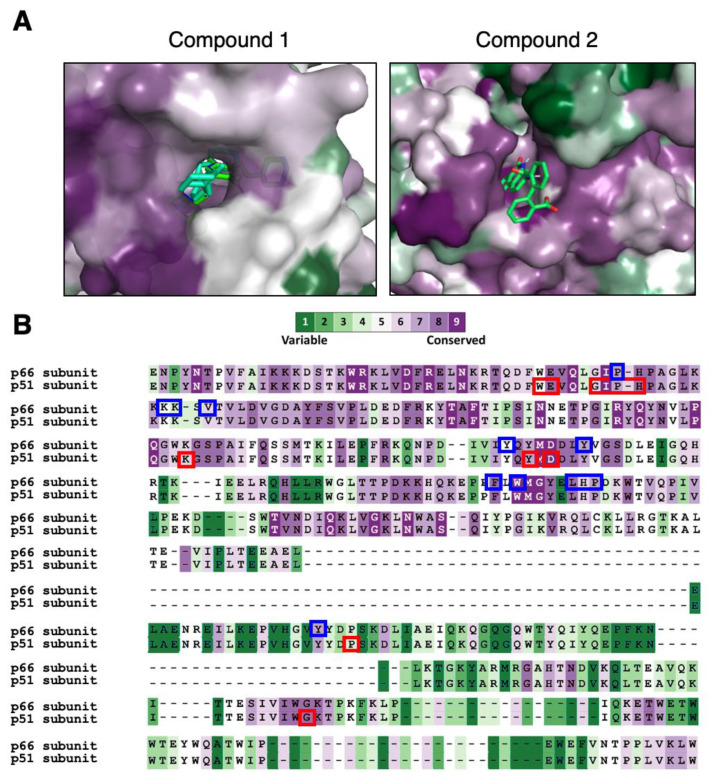
Two binding sites on HIV-1 RT that are highly conserved. (**A**) The binding sites on p66 and p51 complexed with compound 1 and 2, respectively, are shown in surface representation (colored according to the level of conservation among the RT families). The two potential inhibitory compounds are shown as sticks. (**B**) Sequence alignment of the binding sites on p66 and p51, for compound 1 (blue boxes) and compound 2 (red boxes). The conservation color scheme is the same as in (**A**). An animated binding of the compounds at the pockets via augmented reality can be viewed using our “*APD AR Holistic Review*” app available on both Google and Apple app stores (for more details, see Poh et al. [47,48]).

Our structural conservation analysis showed the compound 2 binding region on HIV-1 p51 to be conserved across multiple RTs (Figure 5 and Supplementary Data). Since viruses can develop cross-resistance to multiple drugs [28,37,38], and Y183 has yet emerged as a drug resistant mutated location, compound 2 serves as a potentially crucial scaffold for targeting drug-resistant HIV-1 variants, and with the conservation of the site across a wide range of RT families, also as a possible broad-spectrum antiviral RTI. By taking a holistic approach [39] in studying the whole structure of the target proteins and looking for common sites and how resistance develops, as demonstrated for HIV-1 Gag [40], HIV-1 Protease [37], and antibodies [41,42,43,44,45,46], the search for common druggable conserved sites across protein families can add to the success of developing broad-spectrum therapeutics. Nevertheless, given that the search for the p51 inhibitor is still in its infancy stage, it requires more direct evidence of inhibition of p51 and inhibitors that work at nM concentrations before it can be furthered upon for clinical use.

In conclusion, we demonstrated the feasibility of inhibiting HIV RT via the p51 subunit on a previously identified druggable pocket, revealing possible RTI scaffolds, and the necessary structural engagements for further improvements. Despite inhibiting only at micromolar range, the scaffolds are a promising starting point for further development of new HIV-1 RT lead candidates and even potential broad-spectrum RTIs.

## 4. Materials & Methods

### 4.1. RNA Extraction

RNA was extracted from EXPI293F (Invitrogen, Cat no. A14527) cells using TRIzol Reagent (Thermo Fisher Scientific, Cat. No.: 15596018) according to the manufacturer’s recommendations. The RNA was treated with RNase-free DNase I (Roche, Cat. No.: 04716728001) and stored at −80 °C.

### 4.2. Library Reagents Preparation

Forty NCI Diversity Set V chemical compounds from the National Cancer Institute (NCI) Developmental Therapeutic Program’s Open Compound Repository, National Institutes of Health (NIH) (http://dtp.cancer.gov) and Nevirapine (MedChemExpress LLC, Cat No. HY-10570) were reconstituted in dimethyl sulfoxide (DMSO) and stored at 4 °C. The chemicals were initially screened at 300 µM, and potential hit compounds were then used at various concentrations: 1000 µM, 100 µM, 10 µM, and 1 µM.

### 4.3. cDNA Synthesis Reverse Transcription (HIV-1)

All of the library compounds were first warmed to 37 °C for 10 min for better solubility prior to addition into the RT reaction mix. To form HIV-1 RT, Sino Biological HIV-1 reverse transcriptase p51 (isolate HXB2, Cat No.: 40244-V07E) and p66 (isolate HXB2, Cat No.: 40244-V07E1) subunits were both used to form the RT complex for cDNA synthesis. The 20 µL cDNA synthesis mix is as follows: 0.5 µL of random hexamers (Thermo Fisher Scientific, Cat No.: SO142, 100µM), 0.5 µL of Oligo d(T)s (Thermo Fisher Scientific, Cat No.: SO131, 100 µM), 1.0 µL of RiboLock RNase Inhibitor (Thermo Fisher Scientific, Cat No.: EO0381, 40 U/µL), 1.0 µL of dNTP mixture (1st Base, Cat. No.: BIO-5120, 10 mM of each dNTP), 4.0 µL of 5 × RT Buffer (250 mM Tris-HCl pH 8.3, 375 mM KCl, 22.5 mM MgCl_2_), 5.0 µL of DEPC-Treated Water, 1.0 µL of RT p66 (0.33 µg/µL), 2.0 µL of RT p66 (0.17 µg/µL), 4.0 µL of candidate inhibitors and 1.0 µg of DNase-treated RNA. The reaction mixes were prepared on ice, followed by incubation in ProFlex 3x 32-Well PCR System (Applied Biosystems) at 25 °C for 18 min, followed by 37 °C for 1 h, and RT termination at 85 °C for 5 min. Reaction mixtures containing DMSO only, without RT were used as the negative control.

### 4.4. Quantitative Polymerase Chain Reaction (qPCR) Gene Amplification

The quantification of gene expression was obtained from Applied Biosystems StepOnePlus^TM^. The GAPDH qPCR reactions were setup according to manufacturer recommendations, containing the reagents PowerUp^TM^ SYBR^®^ Green Master Mix (2X) (Applied Biosystems, Cat No.: A25741), Human GAPDH Forward Primer (5′– ACAACTTTGGTATCGTGGAAGG –3′) and Human GAPDH Reverse Primer (5′– GCCATCACGCCACAGTTTC –3′). The final concentration of DMSO in the reaction mixture is adjusted to 0.25%. Cycling conditions were set at 1 cycle of 50 °C (2 min), 95 °C (2 min), 40 cycles of 95 °C (5 s), 60 °C (30 s). A final melt curve was performed at 95 °C (15 s), 60 °C (ramp rate at 0.3 °C/s), and finally 95 °C (15 s). Independent triplicates were performed with “No RT”, “No Primer”, and “No Template Controls” (NTC). GAPDH amplification was quantified using comparative threshold cycle (Ct) as the following ΔCt method:

(1) $$\;2^{-\Delta Ct}=\;2^{-\left(\;Ct\left(inhibitor-treated\;sample\right)\;-\;Ct\left(treated\;sample\right)\;\right)}$$

Agarose gel electrophoresis analysis was carried out on the qPCR products using 2% agar gels. Bands were validated using GelApp [33]. Densitometry analysis was performed using the Fiji software [26].

### 4.5. Inhibitory Concentration Calculation

The half-maximal inhibitory concentration (IC_50_) of the compound is defined as the amount of compound required to block HIV-1 RT activity by 50%. The best-fit curves were plotted using the relative amount of the amplified GAPDH as calculated in the previous step (using ΔCt method). Curve fitting was applied using Hill equation [49] and IC_50_ was obtained directly from the calculated best fit curve parameters using AAT BioQuest IC_50_ calculator [50].

### 4.6. Statistical Analysis

One-way Analysis Of Variance (ANOVA) followed by Dunnett’s multiple comparison tests were performed using R software (version 3.6.2) [51] to compare the mean values of fold differences relative to the untreated controls (which contains RT and without inhibitor compound, unless otherwise stated). Values of *p* < 0.05 were deemed statistically significant.

### 4.7. Structural Docking

The chemical structures of the two compounds (NSC48443 and NSC127133 from the NCI/DTP Diversity Set V) were retrieved from PubChem library, Compound Identifier (CID): 241,217 and 278,037 (particularly from the ligand code 6B3 in this entry) for compounds 1 and 2, respectively. Wild type HIV-1 RT structure (Protein Data Bank (PDB): 3T19) was processed as previously described [28].

We performed rigid blind docking using the program AutoDock Vina [27] to find potential binding sites in the HIV-1 RT structure for the two identified compounds. We next performed focused docking with flexible treatment of side chains for residues identified as part of the binding sites (as receptor) derived from the initial blind docking. The compound-bound conformations that were significantly different from others and had favorable docking scores were selected for further analysis and subsequent MD simulations.

### 4.8. MD Simulations of HIV-1 RT Complexes

We simulated the whole HIV-1 RT p66-p51 with the selected compound conformations using GROMACS (version 2018) [52], with the CHARMM36m [53] forcefield for the protein, the CHARMM general force field (CGenFF) [54] for the ligand, and the TIP3P [55] water model. Nosé-Hoover [56] and Parrinello-Rahman [57] thermostat and barostat were used to maintain constant temperature and pressure at 300 K and 1 bar, respectively. The particle mesh Ewald (PME) method was used for electrostatics, with a real-space cut-off of 12 Å. A 12-Å cut-off was used to calculate the van der Waal’s potential, switching the energy function after 10 Å. Bonds involving hydrogen atoms were restrained using the LINear Constraint Solver (LINCS) [58] algorithm. We carried out a 20 ns equilibration, restraining the protein backbone atoms, followed by unrestrained simulations for 100 ns in triplicate.

### 4.9. Conventional MD Simulation Analysis

Hydrophobic contacts were calculated using a 4.0 Å cut-off between the ligand heavy atoms and protein carbon atoms. The number of water molecules around the ligand was determined using a cut-off of 3.5 Å between the ligand heavy atoms and water oxygen atoms. Hydrogen bonds were calculated between donor and acceptors of the ligand and the protein using a cut-off of 3.5 Å, with a maximum angle between donor-hydrogen atom and acceptor of 30°. To determine stable positions of the ligand in each binding site, we calculated distances between the center of mass of the ligand and of the binding site residues, which were defined via the LigPlot+ program [29].

### 4.10. Binding Energy Calculations

The molecular mechanics Poisson–Boltzmann Surface Area (MM-PBSA) method was applied to calculate the binding energies of the compounds and their corresponding binding sites on the HIV-1 RT subunits, using *g_mmpbsa* [59]. Various internal dielectric constants of the solute [60,61,62] (ε_in_) were used to estimate the polarizability of the binding sites, e.g., ε_in_ = 2 (hydrophobic), ε_in_ = 8 (polar), or ε_in_ = 20 (highly charged). The external dielectric constant ε_out_ was set to 80 (for water). The calculations were performed using the last 50 ns of each MD simulation trajectory for all independent replicas.

### 4.11. Analysis of Structural Conservation among RT Proteins

Multiple sequence alignment of the HIV-1 RT sequences with 152 retrovirus RT sequences (retrieved from National Center for Biotechnology Information(NCBI) RefSeq Databases) was first performed using ClustalW [63]. The resulting alignment was then subjected to the ConSurf server [64,65] with default parameters to study the conservation of the regions of interest across the RT families.

## Supplementary Materials

The following are available online: Figure S1: The agarose gel electrophoresis of RT-PCR GAPDH products treated with 40 compounds from the NCI/DTP Diversity Set V. We found two compounds (labeled 8 and 20, thereafter named as compound 1 and 2, respectively) showed the RT inhibition in all independent triplicates. Note that the inhibition by compound 25 was not reproduced in all the independent triplicates and hence it was excluded in our subsequent analysis; Figure S2: Initial ligand bound conformations used for different setups of the MD simulations; Figure S3: Molecular simulation analyses of the binding sites for compounds 1 and 2. (A) The center-of-mass distances between the binding sites and compound 2 for three initial conformations that exhibited unstable ligand bound positions, calculated using independent triplicates (black, green and red) of 100 ns trajectories. Probability plots of interactions with their respective binding sites are shown for (B) compound 1 and (C) compound 2; Table S1: Decomposition of binding energies for compound 1 in the binding sites on RT p66 and p51 subunits. MM-PBSA calculations were carried out with the program *g_mmpbsa* [59] for the last 50 ns of each trajectory, using various internal dielectric constants ε_in_ for the solute [60,61,62]. Analyses were performed across independent triplicate simulations, using mean and standard deviation; Table S2: Additional binding analyses for the two compounds in their binding sites on the RT p66 and p51 subunits. Hydrophobic contacts between protein and ligand were calculated using a 4.0 Å cut-off. Hydrogen bonds between protein and ligand were calculated using a cut-off of 3.5 Å and maximum angle between donor-hydrogen atom and acceptor of 30°. The number of water molecules around the ligand was determined using a cut-off of 3.5 Å. Analyses were performed across independent triplicate simulations, using mean and standard deviation; Table S3: Decomposition of binding energies for compound 2 in the binding sites on RT p51 subunits. MM-PBSA calculations were carried out with the program *g_mmpbsa* [59] for the last 50 ns of each trajectory, using various internal dielectric constants ε_in_ for the solute. Analyses were performed across independent triplicate simulations. Only results for the three setups that exhibited stable ligand binding (i.e., conformation 2, 3, and 4) are shown. Analyses were performed across independent triplicate simulations, using mean and standard deviation.

## Accession Codes

PDB code for HIV-1 Reverse Transcriptase in Figure 5 is 3T19.
