# Supplementary material for: An Alternative HIV-1 Non-Nucleoside Reverse Transcriptase Inhibition Mechanism: Targeting the p51 Subunit
Source: Molecules. 2020 Dec 13;25(24):5902. doi: 10.3390/molecules25245902 (PMC7763519; doi:10.3390/molecules25245902)
Supplement: Supplementary file 1 [file molecules-25-05902-s001.zip › Supplementary Materials/Supplementary Data 2.pdf]

ConSurf Color-Coded MSA

```
001 YP_004442836.1   --------------------------------------------------
002 NP_056728.1      --------------------------------------------------
003 YP_009508537.1   --------------------------------------------------
004 YP_004732983.2   --------------------------------------------------
005 YP_009508407.1   --------------------------------------------------
006 NP_040939.1      --------------------------------------------------
007 YP_003864102.1   --------------------------------------------------
008 YP_009002585.1   --------------------------------------------------
009 YP_003284237.1   --------------------------------------------------
010 YP_009268869.1   --------------------------------------------------
011 YP_009506251.1   --------------------------------------------------
012 NP_955611.1      --------------------------------------------------
013 YP_008567619.1   --------------------------------------------------
014 NP_149413.1      --------------------------------------------------
015 YP_004442824.1   --------------------------------------------------
016 YP_001931967.1   --------------------------------------------------
017 YP_002455786.1   --------------------------------------------------
018 YP_009109694.1   --------------------------------------------------
019 NP_789739.1      --------------------------------------------------
020 NP_955619.1      --------------------------------------------------
021 YP_224289.1      --------------------------------------------------
022 YP_009182100.1   --------------------------------------------------
023 NP_705927.1      --------------------------------------------------
024 YP_009508577.1   --------------------------------------------------
025 YP_004442830.1   --------------------------------------------------
026 NP_758887.1      --------------------------------------------------
027 YP_009508406.1   --------------------------------------------------
028 YP_610965.1      --------------------------------------------------
029 YP_001931961.1   --------------------------------------------------
030 YP_241114.1      --------------------------------------------------
031 YP_006732334.1   --------------------------------------------------
032 YP_009041481.1   --------------------------------------------------
033 YP_001036293.1   --------------------------------------------------
034 YP_605811.1      --------------------------------------------------
035 NP_955577.1      --------------------------------------------------
036 YP_003208050.1   --------------------------------------------------
037 NP_789740.1      --------------------------------------------------
038 YP_004442839.1   --------------------------------------------------
039 YP_009507791.1   --------------------------------------------------
040 YP_004581513.1   --------------------------------------------------
041 NP_043933.1      --------------------------------------------------
042 YP_009507248.1   --------------------------------------------------
043 YP_002117531.1   --------------------------------------------------
044 YP_004347415.1   --------------------------------------------------
045 NP_056891.1      -------------------------------------------------M
046 YP_009513242.1   --------------------------------------------------
047 YP_009352866.1   --------------------------------------------------
048 YP_009165750.1   --------------------------------------------------
049 NP_040840.1      --------------------------------------------------
050 YP_004222728.1   --------------------------------------------------
051 YP_009506264.1   --------------------------------------------------
052 YP_567050.1      --------------------------------------------------
053 YP_009508571.1   --------------------------------------------------
054 NP_056803.1      --------------------------------------------------
055 NP_955564.1      --------------------------------------------------
056 YP_009508411.1   --------------------------------------------------
057 YP_009140788.1   --------------------------------------------------
058 YP_009508410.1   --------------------------------------------------
059 NP_040333.1      --------------------------------------------------
060 YP_008992013.1   --------------------------------------------------
061 NP_041734.1      --------------------------------------------------
062 NP_056895.1      --------------------------------------------------
063 YP_009109689.1   --------------------------------------------------
064 YP_002519387.1   --------------------------------------------------
065 YP_595725.1      --------------------------------------------------
066 YP_009508551.1   --------------------------------------------------
067 YP_001497148.1   --------------------------------------------------
068 NP_041186.1      --------------------------------------------------
069 NP_663784.1      --------------------------------------------------
```

```
070 YP_009508443.1   --------------------------------------------------
071 YP_003987465.1   --------------------------------------------------
072 NP_861410.1      --------------------------------------------------
073 YP_009345071.1   --------------------------------------------------
074 YP_009508582.1   --------------------------------------------------
075 NP_569141.1      --------------------------------------------------
076 YP_002916057.1   --------------------------------------------------
077 NP_777384.2      --------------------------------------------------
078 YP_233110.1      --------------------------------------------------
079 NP_542258.1      --------------------------------------------------
080 YP_009130664.1   --------------------------------------------------
081 YP_009508566.1   --------------------------------------------------
082 YP_006607892.1   --------------------------------------------------
083 YP_009506270.1   --------------------------------------------------
084 NP_042513.1      --------------------------------------------------
085 YP_006907834.1   --------------------------------------------------
086 YP_004442827.1   --------------------------------------------------
087 YP_009408594.1   --------------------------------------------------
088 YP_009508561.1   --------------------------------------------------
089 NP_056907.1      --------------------------------------------------
090 YP_002308474.1   --------------------------------------------------
091 YP_007761644.1   --------------------------------------------------
092 NP_040550.1      --------------------------------------------------
093 NP_758808.1      --------------------------------------------------
094 YP_009345075.1   --------------------------------------------------
095 NP_068729.1      --------------------------------------------------
096 NP_047255.1      MSGASSGTAIGAHLFGVSPEYRVLIGDEGAGPSKSLSEVSFSVWYRSRAA
097 YP_006273075.1   --------------------------------------------------
098 YP_009508888.1   --------------------------------------------------
099 NP_395469.1      --------------------------------------------------
100 YP_009508546.1   --------------------------------------------------
101 NP_056762.1      --------------------------------------------------
102 YP_009508556.1   --------------------------------------------------
103 NP_569153.1      --------------------------------------------------
104 NP_043924.1      --------------------------------------------------
105 YP_009243641.1   --------------------------------------------------
106 YP_004300274.1   --------------------------------------------------
107 NP_056848.1      --------------------------------------------------
108 YP_006495799.1   --------------------------------------------------
109 YP_009508409.1   --------------------------------------------------
110 NP_687035.1      --------------------------------------------------
111 NP_045937.2      --------------------------------------------------
112 NP_612577.1      --------------------------------------------------
113 NP_619548.1      --------------------------------------------------
114 NP_056880.1      ------------------------------------------------MG
115 YP_009121747.1   --------------------------------------------------
116 YP_009113237.2   --------------------------------------------------
117 YP_009508408.1   --------------------------------------------------
118 NP_955579.1      --------------------------------------------------
119 YP_009229919.1   --------------------------------------------------
120 YP_004442833.1   --------------------------------------------------
121 NP_862833.2      --------------------------------------------------
122 NP_040563.1      --------------------------------------------------
123 YP_443922.1      -----MASSQPLKMKFSEYLDQRMQDRAGGKVQWKDHKKVWERLKLRWTQ
124 NP_057933.2      --------------------------------------------------
125 YP_009116631.1   --------------------------------------------------
126 YP_009513249.1   --------------------------------------------------
127 YP_223871.1      --------------------------------------------------
128 NP_127504.1      --------------------------------------------------
129 NP_044929.1      --------------------------------------------------
130 NP_057860.1      --------------------------------------------------
131 NP_040973.1      --------------------------------------------------
132 NP_777317.1      --------------------------------------------------
133 NP_054716.1      --------------------------------------------------
134 YP_001856242.1   --------------------------------------------------
135 NP_954565.2      -------------------------------------------------M
136 NP_569150.1      --------------------------------------------------
137 NP_659397.1      --------------------------------------------------
138 YP_009513211.1   --------------------------------------------------
139 YP_233107.1      --------------------------------------------------
140 YP_009109692.1   --------------------------------------------------
141 NP_057849.4      --------------------------------------------------
142 NP_049560.1      --------------------------------------------------
```

```
143 YP_009506267.1       - - - - - - - - - - - - - - - - - - - - - - - - - - - - - - - - - - - - - - - - - - - - - - - - - -
144 NP_039820.1          - - - - - - - - - - - - - - - - - - - - - - - - - - - - - - - - - - - - - - - - - - - - - - - - - -
145 NP_056790.1          - - - - - - - - - - - - - - - - - - - - - - - - - - - - - - - - - - - - - - - - - - - - - - - - - -
146 YP_002321513.1       - - - - - - - - - - - - - - - - - - - - - - - - - - - - - - - - - - - - - - - - - - - - - - - - - -
147 NP_955591.1          - - - - - - - - - - - - - - - - - - - - - - - - - - - - - - - - - - - - - - - - - - - - - - - - - -
148 YP_001956722.2       - - - - - - - - - - - - - - - - - - - - - - - - - - - - - - - - - - - - - - - - - - - - - - - - - -
149 NP_056902.1          - - - - - - - - - - - - - - - - - - - - - - - - - - - - - - - - - - - - - - - - - - - - - - - - - -
150 NP_056886.1          - - - - - - - - - - - - - - - - - - - - - - - - - - - - - - - - - - - - - - - - - - - - - - - - - -
151 NP_041261.1          - - - - - - - - - - - - - - - - - - - - - - - - - - - - - - - - - - - - - - - - - - - - - - - - - -
```

```
001 YP_004442836.1       - - - - - - - - - - - - - - - - - - - - - - - - - - - - - - - - - - - - - - - - - - - - - - - - - -
002 NP_056728.1          - - - - - - - - - - - - - - - - - - - - - - - - - - - - - - - - - - - - - - - - - - - - - - - - - -
003 YP_009508537.1       - - - - - - - - - - - - - - - - - - - - - - - - - - - - - - - - - - - - - - - - - - - - - - - - - -
004 YP_004732983.2       - - - - - - - - - - - - - - - - - - - - - - - - - - - - - - - - - - - - - - - - - - - - - - - - - -
005 YP_009508407.1       - - - - - - - - - - - - - - - - - - - - - - - - - - - - - - - - - - - - - - - - - - - - - - - - - -
006 NP_040939.1          - - - - - - - - - - - - - - - - - - - - - - - - - - - - - - - - - - - - - - - - - - - - - - - - - -
007 YP_003864102.1       - - - - - - - - - - - - - - - - - - - - - - - - - - - - - - - - - - - - - - - - - - - - - - - - - -
008 YP_009002585.1       - - - - - - - - - - - - - - - - - - - - - - - - - - - - - - - - - - - - - - - - - - - - - - - - - -
009 YP_003284237.1       - - - - - - - - - - - - - - - - - - - - - - - - - - - - - - - - - - - - - - - - - - - - - - - - - -
010 YP_009268869.1       - - - - - - - - - - - - - - - - - - - - - - - - - - - - - - - - - - - - - - - - - - - - - - - - - -
011 YP_009506251.1       - - - - - - - - - - - - - - - - - - - - - - - - - - - - - - - - - - - - - - - - - - - - - - - - - -
012 NP_955611.1          - - - - - - - - - - - - - - - - - - - - - - - - - - - - - - - - - - - - - - - - - - - - - - - - - -
013 YP_008567619.1       - - - - - - - - - - - - - - - - - - - - - - - - - - - - - - - - - - - - - - - - - - - - - - - - - -
014 NP_149413.1          - - - - - - - - - - - - - - - - - - - - - - - - - - - - - - - - - - - - - - - - - - - - - - - - - -
015 YP_004442824.1       - - - - - - - - - - - - - - - - - - - - - - - - - - - - - - - - - - - - - - - - - - - - - - - - - -
016 YP_001931967.1       - - - - - - - - - - - - - - - - - - - - - - - - - - - - - - - - - - - - - - - - - - - - - - - - - -
017 YP_002455786.1       - - - - - - - - - - - - - - - - - - - - - - - - - - - - - - - - - - - - - - - - - - - - - - - - - -
018 YP_009109694.1       - - - - - - - - - - - - - - - - - - - - - - - - - - - - - - - - - - - - - - - - - - - - - - - - - -
019 NP_789739.1          - - - - - - - - - - - - - - - - - - - - - - - - - - - - - - - - - - - - - - - - - - - - - - - - - -
020 NP_955619.1          - - - - - - - - - - - - - - - - - - - - - - - - - - - - - - - - - - - - - - - - - - - - - - - - - -
021 YP_224289.1          - - - - - - - - - - - - - - - - - - - - - - - - - - - - - - - - - - - - - - - - - - - - - - - - - -
022 YP_009182100.1       - - - - - - - - - - - - - - - - - - - - - - - - - - - - - - - - - - - - - - - - - - - - - - - - - -
023 NP_705927.1          - - - - - - - - - - - - - - - - - - - - - - - - - - - - - - - - - - - - - - - - - - - - - - - - - -
024 YP_009508577.1       - - - - - - - - - - - - - - - - - - - - - - - - - - - - - - - - - - - - - - - - - - - - - - - - - -
025 YP_004442830.1       - - - - - - - - - - - - - - - - - - - - - - - - - - - - - - - - - - - - - - - - - - - - - - - - - -
026 NP_758887.1          - - - - - - - - - - - - - - - - - - - - - - - - - - - - - - - - - - - - - - - - - - - - - - - - - -
027 YP_009508406.1       - - - - - - - - - - - - - - - - - - - - - - - - - - - - - - - - - - - - - - - - - - - - - - - - - -
028 YP_610965.1          - - - - - - - - - - - - - - - - - - - - - - - - - - - - - - - - - - - - - - - - - - - - - - - - - -
029 YP_001931961.1       - - - - - - - - - - - - - - - - - - - - - - - - - - - - - - - - - - - - - - - - - - - - - - - - - -
030 YP_241114.1          - - - - - - - - - - - - - - - - - - - - - - - - - - - - - - - - - - - - - - - - - - - - - - - - - -
031 YP_006732334.1       - - - - - - - - - - - - - - - - - - - - - - - - - - - - - - - - - - - - - - - - - - - - - - - - - -
032 YP_009041481.1       - - - - - - - - - - - - - - - - - - - - - - - - - - - - - - - - - - - - - - - - - - - - - - - - - -
033 YP_001036293.1       - - - - - - - - - - - - - - - - - - - - - - - - - - - - - - - - - - - - - - - - - - - - - - - - - -
034 YP_605811.1          - - - - - - - - - - - - - - - - - - - - - - - - - - - - - - - - - - - - - - - - - - - - - - - - - -
035 NP_955577.1          - - - - - - - - - - - - - - - - - - - - - - - - - - - - - - - - - - - - - - - - - - - - - - - - - -
036 YP_003208050.1       - - - - - - - - - - - - - - - - - - - - - - - - - - - - - - - - - - - - - - - - - - - - - - - - - -
037 NP_789740.1          - - - - - - - - - - - - - - - - - - - - - - - - - - - - - - - - - - - - - - - - - - - - - - - - - -
038 YP_004442839.1       - - - - - - - - - - - - - - - - - - - - - - - - - - - - - - - - - - - - - - - - - - - - - - - - - -
039 YP_009507791.1       - - - - - - - - - - - - - - - - - - - - - - - - - - - - - - - - - - - - - - - - - - - - - - - - - -
040 YP_004581513.1       - - - - - - - - - - - - - - - - - - - - - - - - - - - - - - - - - - - - - - - - - - - - - - - - - -
041 NP_043933.1          - - - - - - - - - - - - - - - - - - - - - - - - - - - - - - - - - - - - - - - - - - - - - - - - - -
042 YP_009507248.1       - - - - - - - - - - - - - - - - - - - - - - - - - - - - - - - - - - - - - - - - - - - - - - - - - -
043 YP_002117531.1       - - - - - - - - - - - - - - - - - - - - - - - - - - - - - - - - - - - - - - - - - - - - - - - - - -
044 YP_004347415.1       - - - - - - - - - - - - - - - - - - - - - - - - - - - - - - - - - - - - - - - - - - - - - - - - - -
045 NP_056891.1          G Q E L S Q H E R Y V E Q L K Q A L K T R G V K V K Y A D L L K F F D F V K D T C P W F P Q E G T I
046 YP_009513242.1       - - - - - - - - - - - - - - - - - - - - - - - - - - - - - - - - - - - - - - - - - - - - - - - - - -
047 YP_009352866.1       - - - - - - - - - - - - - - - - - - - - - - - - - - - - - - - - - - - - - - - - - - - - - - - - - -
048 YP_009165750.1       - - - - - - - - - - - - - - - - - - - - - - - - - - - - - - - - - - - - - - - - - - - - - - - - - -
049 NP_040840.1          - - - - - - - - - - - - - - - - - - - - - - - - - - - - - - - - - - - - - - - - - - - - - - - - - -
050 YP_004222728.1       - - - - - - - - - - - - - - - - - - - - - - - - - - - - - - - - - - - - - - - - - - - - - - - - - -
051 YP_009506264.1       - - - - - - - - - - - - - - - - - - - - - - - - - - - - - - - - - - - - - - - - - - - - - - - - - -
052 YP_567050.1          - - - - - - - - - - - - - - - - - - - - - - - - - - - - - - - - - - - - - - - - - - - - - - - - - -
053 YP_009508571.1       - - - - - - - - - - - - - - - - - - - - - - - - - - - - - - - - - - - - - - - - - - - - - - - - - -
054 NP_056803.1          - - - - - - - - - - - - - - - - - - - - - - - - - - - - - - - - - - - - - - - - - - - - - - - - - -
055 NP_955564.1          - - - - - - - - - - - - - - - - - - - - - - - - - - - - - - - - - - - - - - - - - - - - - - - - - -
056 YP_009508411.1       - - - - - - - - - - - - - - - - - - - - - - - - - - - - - - - - - - - - - - - - - - - - - - - - - -
057 YP_009140788.1       - - - - - - - - - - - - - - - - - - - - - - - - - - - - - - - - - - - - - - - - - - - - - - - - - -
058 YP_009508410.1       - - - - - - - - - - - - - - - - - - - - - - - - - - - - - - - - - - - - - - - - - - - - - - - - - -
059 NP_040333.1          - - - - - - - - - - - - - - - - - - - - - - - - - - - - - - - - - - - - - - - - - - - - - - - - - -
060 YP_008992013.1       - - - - - - - - - - - - - - - - - - - - - - - - - - - - - - - - - - - - - - - - - - - - - - - - - -
061 NP_041734.1          - - - - - - - - - - - - - - - - - - - - - - - - - - - - - - - - - - - - - - - - - - - - - - - - - -
```

```
062 NP_056895.1       --------------------------------------------------
063 YP_009109689.1    ------------------------MGQTLTTPLSLTLTHFSDVRARAHNL
064 YP_002519387.1    --------------------------------------------------
065 YP_595725.1       --------------------------------------------------
066 YP_009508551.1    --------------------------------------------------
067 YP_001497148.1    ------------------------MGQTLTTPLTLTLTHFSDVQARAHNL
068 NP_041186.1       --------------------------------------------------
069 NP_663784.1       --------------------------------------------------
070 YP_009508443.1    --------------------------------------------------
071 YP_003987465.1    --------------------------------------------------
072 NP_861410.1       --------------------------------------------------
073 YP_009345071.1    --------------------------------------------------
074 YP_009508582.1    --------------------------------------------------
075 NP_569141.1       --------------------------------------------------
076 YP_002916057.1    --------------------------------------------------
077 NP_777384.2       --------------------------------------------------
078 YP_233110.1       --------------------------------------------------
079 NP_542258.1       --------------------------------------------------
080 YP_009130664.1    --------------------------------------------------
081 YP_009508566.1    --------------------------------------------------
082 YP_006607892.1    --------------------------------------------------
083 YP_009506270.1    --------------------------------------------------
084 NP_042513.1       --------------------------------------------------
085 YP_006907834.1    --------------------------------------------------
086 YP_004442827.1    --------------------------------------------------
087 YP_009408594.1    --------------------------------------------------
088 YP_009508561.1    --------------------------------------------------
089 NP_056907.1       --------------------------------------------------
090 YP_002308474.1    --------------------------------------------------
091 YP_007761644.1    --------------------------------------------------
092 NP_040550.1       --------------------------------------------------
093 NP_758808.1       --------------------------------------------------
094 YP_009345075.1    --------------------------------------------------
095 NP_068729.1       --------------------------------------------------
096 NP_047255.1       RLVIFCLVASFLVPCLTFLIAETVMGQTITTPLSLTLDHWSEVRARAHNQ
097 YP_006273075.1    --------------------------------------------------
098 YP_009508888.1    --------------------------------------------------
099 NP_395469.1       --------------------------------------------------
100 YP_009508546.1    --------------------------------------------------
101 NP_056762.1       --------------------------------------------------
102 YP_009508556.1    --------------------------------------------------
103 NP_569153.1       --------------------------------------------------
104 NP_043924.1       --------------------------------------------------
105 YP_009243641.1    --------------------------------------------------
106 YP_004300274.1    --------------------------------------------------
107 NP_056848.1       --------------------------------------------------
108 YP_006495799.1    --------------------------------------------------
109 YP_009508409.1    --------------------------------------------------
110 NP_687035.1       --------------------------------------------------
111 NP_045937.2       --------------------------------------------------
112 NP_612577.1       --------------------------------------------------
113 NP_619548.1       --------------------------------------------------
114 NP_056880.1       VSGSKGQKLFVSVLQRLLSERGLHVKESSAIEFYQFLIKVSPWFPEEGGL
115 YP_009121747.1    --------------------------------------------------
116 YP_009113237.2    --------------------------------------------------
117 YP_009508408.1    --------------------------------------------------
118 NP_955579.1       --------------------------------------------------
119 YP_009229919.1    --------------------------------------------------
120 YP_004442833.1    --------------------------------------------------
121 NP_862833.2       --------------------------------------------------
122 NP_040563.1       --------------------------------------------------
123 YP_443922.1       EGYLAGGAPTRVQLSTMEGELREGKQRAKDSEVDLNRQHVFRKEGTKQRE
124 NP_057933.2       ------------------------MGQTVTTPLSLTLGHWKDVERIAHNQ
125 YP_009116631.1    --------------------------------------------------
126 YP_009513249.1    --------------------------------------------------
127 YP_223871.1       --------------------------------------------------
128 NP_127504.1       --------------------------------------------------
129 NP_044929.1       --------------------------------------------------
130 NP_057860.1       --------------------------------------------------
131 NP_040973.1       --------------------------------------------------
132 NP_777317.1       --------------------------------------------------
133 NP_054716.1       --------------------------------------------------
134 YP_001856242.1    --------------------------------------------------
```

```
135 NP_954565.2        G Q E L S Q H E R Y V E Q L K Q A L K T R G V K V K Y A D L L K F F D F V K D T C P W F P Q E G T I
136 NP_569150.1        - - - - - - - - - - - - - - - - - - - - - - - - - - - - - - - - - - - - - - - - - - - - - - - - - -
137 NP_659397.1        - - - - - - - - - - - - - - - - - - - - - - - - - - - - - - - - - - - - - - - - - - - - - - - - - -
138 YP_009513211.1     - - - - - - - - - - - - - - - - - - - - - - - - - - - - - - - - - - - - - - - - - - - - - - - - - -
139 YP_233107.1        - - - - - - - - - - - - - - - - - - - - - - - - - - - - - - - - - - - - - - - - - - - - - - - - - -
140 YP_009109692.1     - - - - - - - - - - - - - - - - - - - - - - - - - - - - - - - - - - - - - - - - - - - - - - - - - -
141 NP_057849.4        - - - - - - - - - - - - - - - - - - - - - - - - - - - - - - - - - - - - - - - - - - - - - - - - - -
142 NP_049560.1        - - - - - - - - - - - - - - - - - - - - - - - - - - - - - - - - - - - - - - - - - - - - - - - - - -
143 YP_009506267.1     - - - - - - - - - - - - - - - - - - - - - - - - - - - - - - - - - - - - - - - - - - - - - - - - - -
144 NP_039820.1        - - - - - - - - - - - - - - - - - - - - - - - - - - - - - - - - - - - - - - - - - - - - - - - - - -
145 NP_056790.1        - - - - - - - - - - - - - - - - - - - - - - - - - - - - - - - - - - - - - - - - - - - - - - - - - -
146 YP_002321513.1     - - - - - - - - - - - - - - - - - - - - - - - - - - - - - - - - - - - - - - - - - - - - - - - - - -
147 NP_955591.1        - - - - - - - - - - - - - - - - - - - - - - - - - - - - - - - - - - - - - - - - - - - - - - - - - -
148 YP_001956722.2     - - - - - - - - - - - - - - - - - - - - - - - - - - - - - - - - - - - - - - - - - - - - - - - - - -
149 NP_056902.1        - - - - - - - - - - - - - - - - - - - - - - - - - - - - - - - - - - - - - - - - - - - - - - - - - -
150 NP_056886.1        - - - - - - - - - - - - - - - - - - - - - - - - - - - - - - - - - - - - - - - - - - - - - - - - - -
151 NP_041261.1        - - - - - - - - - - - - - - - - - - - - - - - - - - - - - - - - - - - - - - - - - - - - - - - - - -
```

```
001 YP_004442836.1     - - - - - - - - - - - - - - - - - - - - - - - - - - - - - - - - - - - - - - - - - - - - - - - - - -
002 NP_056728.1        - - - - - - - - - - - - - - - - - - - - - - - - - - - - - - - - - - - - - - - - - - - - - - - - - -
003 YP_009508537.1     - - - - - - - - - - - - - - - - - - - - - - - - - - - - - - - - - - - - - - - - - - - - - - - - - -
004 YP_004732983.2     - - - - - - - - - - - - - - - - - - - - - - - - - - - - - - - - - - - - - - - - - - - - - - - - - -
005 YP_009508407.1     - - - - - - - - - - - - - - - - - - - - - - - - - - - - - - - - - - - - - - - - - - - - - - - - - -
006 NP_040939.1        - - - - - - - - - - - - - - - - - - - - - - - - - - - - - - - - - - - - - - - - - - - - - - - - - -
007 YP_003864102.1     - - - - - - - - - - - - - - - - - - - - - - - - - - - - - - - - - - - - - - - - - - - - - - - - - -
008 YP_009002585.1     - - - - - - - - - - - - - - - - - - - - - - - - - - - - - - - - - - - - - - - - - - - - - - - - - -
009 YP_003284237.1     - - - - - - - - - - - - - - - - - - - - - - - - - - - - - - - - - - - - - - - - - - - - - - - - - -
010 YP_009268869.1     - - - - - - - - - - - - - - - - - - - - - - - - - - - - - - - - - - - - - - - - - - - - - - - - - -
011 YP_009506251.1     - - - - - - - - - - - - - - - - - - - - - - - - - - - - - - - - - - - - - - - - - - - - - - - - - -
012 NP_955611.1        - - - - - - - - - - - - - - - - - - - - - - - - - - - - - - - - - - - - - - - - - - - - - - - - - -
013 YP_008567619.1     - - - - - - - - - - - - - - - - - - - - - - - - - - - - - - - - - - - - - - - - - - - - - - - - - -
014 NP_149413.1        - - - - - - - - - - - - - - - - - - - - - - - - - - - - - - - - - - - - - - - - - - - - - - - - - -
015 YP_004442824.1     - - - - - - - - - - - - - - - - - - - - - - - - - - - - - - - - - - - - - - - - - - - - - - - - - -
016 YP_001931967.1     - - - - - - - - - - - - - - - - - - - - - - - - - - - - - - - - - - - - - - - - - - - - - - - - - -
017 YP_002455786.1     - - - - - - - - - - - - - - - - - - - - - - - - - - - - - - - - - - - - - - - - - - - - - - - - - -
018 YP_009109694.1     - - - - - - - - - - - - - - - - - - - - - - - - - - - - - - - - - - - - - - - - - - - - - - - - - -
019 NP_789739.1        - - - - - - - - - - - - - - - - - - - - - - - - - - - - - - - - - - - - - - - - - - - - - - - - - -
020 NP_955619.1        - - - - - - - - - - - - - - - - - - - - - - - - - - - - - - - - - - - - - - - - - - - - - - - - - -
021 YP_224289.1        - - - - - - - - - - - - - - - - - - - - - - - - - - - - - - - - - - - - - - - - - - - - - - - - - -
022 YP_009182100.1     - - - - - - - - - - - - - - - - - - - - - - - - - - - - - - - - - - - - - - - - - - - - - - - - - -
023 NP_705927.1        - - - - - - - - - - - - - - - - - - - - - - - - - - - - - - - - - - - - - - - - - - - - - - - - - -
024 YP_009508577.1     - - - - - - - - - - - - - - - - - - - - - - - - - - - - - - - - - - - - - - - - - - - - - - - - - -
025 YP_004442830.1     - - - - - - - - - - - - - - - - - - - - - - - - - - - - - - - - - - - - - - - - - - - - - - - - - -
026 NP_758887.1        - - - - - - - - - - - - - - - - - - - - - - - - - - - - - - - - - - - - - - - - - - - - - - - - - -
027 YP_009508406.1     - - - - - - - - - - - - - - - - - - - - - - - - - - - - - - - - - - - - - - - - - - - - - - - - - -
028 YP_610965.1        - - - - - - - - - - - - - - - - - - - - - - - - - - - - - - - - - - - - - - - - - - - - - - - - - -
029 YP_001931961.1     - - - - - - - - - - - - - - - - - - - - - - - - - - - - - - - - - - - - - - - - - - - - - - - - - -
030 YP_241114.1        - - - - - - - - - - - - - - - - - - - - - - - - - - - - - - - - - - - - - - - - - - - - - - - - - -
031 YP_006732334.1     - - - - - - - - - - - - - - - - - - - - - - - - - - - - - - - - - - - - - - - - - - - - - - - - - -
032 YP_009041481.1     - - - - - - - - - - - - - - - - - - - - - - - - - - - - - - - - - - - - - - - - - - - - - - - - - -
033 YP_001036293.1     - - - - - - - - - - - - - - - - - - - - - - - - - - - - - - - - - - - - - - - - - - - - - - - - - -
034 YP_605811.1        - - - - - - - - - - - - - - - - - - - - - - - - - - - - - - - - - - - - - - - - - - - - - - - - - -
035 NP_955577.1        - - - - - - - - - - - - - - - - - - - - - - - - - - - - - - - - - - - - - - - - - - - - - - - - - -
036 YP_003208050.1     - - - - - - - - - - - - - - - - - - - - - - - - - - - - - - - - - - - - - - - - - - - - - - - - - -
037 NP_789740.1        - - - - - - - - - - - - - - - - - - - - - - - - - - - - - - - - - - - - - - - - - - - - - - - - - -
038 YP_004442839.1     - - - - - - - - - - - - - - - - - - - - - - - - - - - - - - - - - - - - - - - - - - - - - - - - - -
039 YP_009507791.1     - - - - - - - - - - - - - - - - - - - - - - - - - - - - - - - - - - - - - - - - - - - - - - - - - -
040 YP_004581513.1     - - - - - - - - - - - - - - - - - - - - - - - - - - - - - - - - - - - - - - - - - - - - - - - - - -
041 NP_043933.1        - - - - - - - - - - - - - - - - - - - - - - - - - - - - - - - - - - - - - - - - - - - - - - - - - -
042 YP_009507248.1     - - - - - - - - - - - - - - - - - - - - - - - - - - - - - - - - - - - - - - - - - - - - - - - - - -
043 YP_002117531.1     - - - - - - - - - - - - - - - - - - - - - - - - - - - - - - - - - - - - - - - - - - - - - - - - - -
044 YP_004347415.1     - - - - - - - - - - - - - - - - - - - - - - - - - - - - - - - - - - - - - - - - - - - - - - - - - -
045 NP_056891.1        D I K R W R R V G D C F Q D Y Y N T F G P E K V P V T A F S Y W N L I K E L I D K K E V N P Q V M A
046 YP_009513242.1     - - - - - - - - - - - - - - - - - - - - - - - - - - - - - - - - - - - - - - - - - - - - - - - - - -
047 YP_009352866.1     - - - - - - - - - - - - - - - - - - - - - - - - - - - - - - - - - - - - - - - - - - - - - - - - - -
048 YP_009165750.1     - - - - - - - - - - - - - - - - - - - - - - - - - - - - - - - - - - - - - - - - - - - - - - - - - -
049 NP_040840.1        - - - - - - - - - - - - - - - - - - - - - - - - - - - - - - - - - - - - - - - - - - - - - - - - - -
050 YP_004222728.1     - - - - - - - - - - - - - - - - - - - - - - - - - - - - - - - - - - - - - - - - - - - - - - - - - -
051 YP_009506264.1     - - - - - - - - - - - - - - - - - - - - - - - - - - - - - - - - - - - - - - - - - - - - - - - - - -
052 YP_567050.1        - - - - - - - - - - - - - - - - - - - - - - - - - - - - - - - - - - - - - - - - - - - - - - - - - -
053 YP_009508571.1     - - - - - - - - - - - - - - - - - - - - - - - - - - - - - - - - - - - - - - - - - - - - - - - - - -
```

```
054 NP_056803.1       --------------------------------------------------
055 NP_955564.1       --------------------------------------------------
056 YP_009508411.1    --------------------------------------------------
057 YP_009140788.1    --------------------------------------------------
058 YP_009508410.1    --------------------------------------------------
059 NP_040333.1       --------------------------------------------------
060 YP_008992013.1    --------------------------------------------------
061 NP_041734.1       --------------------------------------------------
062 NP_056895.1       --------------------------------------------------
063 YP_009109689.1    SVGVRKGRWQTFCSSEWPTLHVGWPRDGTFDLSVILQVKTKVMDPGPHGH
064 YP_002519387.1    --------------------------------------------------
065 YP_595725.1       --------------------------------------------------
066 YP_009508551.1    --------------------------------------------------
067 YP_001497148.1    SLEVRKGRWRTYCSSEWPTLSVGWPRDGTFDLSIILQVKTKVMDPGPRGH
068 NP_041186.1       --------------------------------------------------
069 NP_663784.1       --------------------------------------------------
070 YP_009508443.1    --------------------------------------------------
071 YP_003987465.1    --------------------------------------------------
072 NP_861410.1       --------------------------------------------------
073 YP_009345071.1    --------------------------------------------------
074 YP_009508582.1    --------------------------------------------------
075 NP_569141.1       --------------------------------------------------
076 YP_002916057.1    --------------------------------------------------
077 NP_777384.2       --------------------------------------------------
078 YP_233110.1       --------------------------------------------------
079 NP_542258.1       --------------------------------------------------
080 YP_009130664.1    --------------------------------------------------
081 YP_009508566.1    --------------------------------------------------
082 YP_006607892.1    --------------------------------------------------
083 YP_009506270.1    --------------------------------------------------
084 NP_042513.1       --------------------------------------------------
085 YP_006907834.1    --------------------------------------------------
086 YP_004442827.1    --------------------------------------------------
087 YP_009408594.1    --------------------------------------------------
088 YP_009508561.1    --------------------------------------------------
089 NP_056907.1       --------------------------------------------------
090 YP_002308474.1    --------------------------------------------------
091 YP_007761644.1    --------------------------------------------------
092 NP_040550.1       --------------------------------------------------
093 NP_758808.1       --------------------------------------------------
094 YP_009345075.1    --------------------------------------------------
095 NP_068729.1       --------------------------------------------------
096 NP_047255.1       GVEVRKKKWITLCEAEWVMMNVGWPREGTFSLDNISQVEKKIFAPGPYGH
097 YP_006273075.1    --------------------------------------------------
098 YP_009508888.1    --------------------------------------------------
099 NP_395469.1       --------------------------------------------------
100 YP_009508546.1    --------------------------------------------------
101 NP_056762.1       --------------------------------------------------
102 YP_009508556.1    --------------------------------------------------
103 NP_569153.1       --------------------------------------------------
104 NP_043924.1       --------------------------------------------------
105 YP_009243641.1    --------------------------------------------------
106 YP_004300274.1    --------------------------------------------------
107 NP_056848.1       --------------------------------------------------
108 YP_006495799.1    --------------------------------------------------
109 YP_009508409.1    --------------------------------------------------
110 NP_687035.1       --------------------------------------------------
111 NP_045937.2       --------------------------------------------------
112 NP_612577.1       --------------------------------------------------
113 NP_619548.1       --------------------------------------------------
114 NP_056880.1       NLQDWKRVGREMKRYAAEHGTDSIPKQAYPIWLQLREILTEQSDLVLLSA
115 YP_009121747.1    --------------------------------------------------
116 YP_009113237.2    --------------------------------------------------
117 YP_009508408.1    --------------------------------------------------
118 NP_955579.1       --------------------------------------------------
119 YP_009229919.1    --------------------------------------------------
120 YP_004442833.1    --------------------------------------------------
121 NP_862833.2       --------------------------------------------------
122 NP_040563.1       --------------------------------------------------
123 YP_443922.1       KAEEELRIGTWAIEETRRFIMLRTPAVMGIATDMKEGGVPTISPATPAEN
124 NP_057933.2       SVDVKKRRWVTFCSAEWPTFNVGWPRDGTFNRDLITQVKIKVFSPGPHGH
125 YP_009116631.1    --------------------------------------------------
126 YP_009513249.1    --------------------------------------------------
```

```
127 YP_223871.1       - - - - - - - - - - - - - - - - - - - - - - - - - - - - - - - - - - - - - - - - - - - - - - - - - -
128 NP_127504.1       - - - - - - - - - - - - - - - - - - - - - - - - - - - - - - - - - - - - - - - - - - - - - - - - - -
129 NP_044929.1       - - - - - - - - - - - - - - - - - - - - - - - - - - - - - - - - - - - - - - - - - - - - - - - - - -
130 NP_057860.1       - - - - - - - - - - - - - - - - - - - - - - - - - - - - - - - - - - - - - - - - - - - - - - - - - -
131 NP_040973.1       - - - - - - - - - - - - - - - - - - - - - - - - - - - - - - - - - - - - - - - - - - - - - - - - - -
132 NP_777317.1       - - - - - - - - - - - - - - - - - - - - - - - - - - - - - - - - - - - - - - - - - - - - - - - - - -
133 NP_054716.1       - - - - - - - - - - - - - - - - - - - - - - - - - - - - - - - - - - - - - - - - - - - - - - - - - -
134 YP_001856242.1    - - - - - - - - - - - - - - - - - - - - - - - - - - - - - - - - - - - - - - - - - - - - - - - - - -
135 NP_954565.2       D I K R W R R V G D C F Q D Y Y N T F G P E K V P V T A F S Y W N L I K E L I D K K E V N P Q V M A
136 NP_569150.1       - - - - - - - - - - - - - - - - - - - - - - - - - - - - - - - - - - - - - - - - - - - - - - - - - -
137 NP_659397.1       - - - - - - - - - - - - - - - - - - - - - - - - - - - - - - - - - - - - - - - - - - - - - - - - - -
138 YP_009513211.1    - - - - - - - - - - - - - - - - - - - - - - - - - - - - - - - - - - - - - - - - - - - - - - - - - -
139 YP_233107.1       - - - - - - - - - - - - - - - - - - - - - - - - - - - - - - - - - - - - - - - - - - - - - - - - - -
140 YP_009109692.1    - - - - - - - - - - - - - - - - - - - - - - - - - - - - - - - - - - - - - - - - - - - - - - - - - -
141 NP_057849.4       - - - - - - - - - - - - - - - - - - - - - - - - - - - - - - - - - - - - - - - - - - - - - - - - - -
142 NP_049560.1       - - - - - - - - - - - - - - - - - - - - - - - - - - - - - - - - - - - - - - - - - - - - - - - - - -
143 YP_009506267.1    - - - - - - - - - - - - - - - - - - - - - - - - - - - - - - - - - - - - - - - - - - - - - - - - - -
144 NP_039820.1       - - - - - - - - - - - - - - - - - - - - - - - - - - - - - - - - - - - - - - - - - - - - - - - - - -
145 NP_056790.1       - - - - - - - - - - - - - - - - - - - - - - - - - - - - - - - - - - - - - - - - - - - - - - - - - -
146 YP_002321513.1    - - - - - - - - - - - - - - - - - - - - - - - - - - - - - - - - - - - - - - - - - - - - - - - - - -
147 NP_955591.1       - - - - - - - - - - - - - - - - - - - - - - - - - - - - - - - - - - - - - - - - - - - - - - - - - -
148 YP_001956722.2    - - - - - - - - - - - - - - - - - - - - - - - - - - - - - - - - - - - - - - - - - - - - - - - - - -
149 NP_056902.1       - - - - - - - - - - - - - - - - - - - - - - - - - - - - - - - - - - - - - - - - - - - - - - - - - -
150 NP_056886.1       - - - - - - - - - - - - - - - - - - - - - - - - - - - - - - - - - - - - - - - - - - - - - - - - - -
151 NP_041261.1       - - - - - - - - - - - - - - - - - - - - - - - - - - - - - - - - - - - - - - - - - - - - - - - - - -
```

```
001 YP_004442836.1    - - - - - - - - - - - - - - - - - - - - - - - - - - - - - - - - - - - - - - - - - - - - - - - - - -
002 NP_056728.1       - - - - - - - - - - - - - - - - - - - - - - - - - - - - - - - - - - - - - - - - - - - - - - - - - -
003 YP_009508537.1    - - - - - - - - - - - - - - - - - - - - - - - - - - - - - - - - - - - - - - - - - - - - - - - - - -
004 YP_004732983.2    - - - - - - - - - - - - - - - - - - - - - - - - - - - - - - - - - - - - - - - - - - - - - - - - - -
005 YP_009508407.1    - - - - - - - - - - - - - - - - - - - - - - - - - - - - - - - - - - - - - - - - - - - - - - - - - -
006 NP_040939.1       - - - - - - - - - - - - - - - - - - - - - - - - - - - - - - - - - - - - - - - - - - - - - - - - - -
007 YP_003864102.1    - - - - - - - - - - - - - - - - - - - - - - - - - - - - - - - - - - - - - - - - - - - - - - - - - -
008 YP_009002585.1    - - - - - - - - - - - - - - - - - - - - - - - - - - - - - - - - - - - - - - - - - - - - - - - - - -
009 YP_003284237.1    - - - - - - - - - - - - - - - - - - - - - - - - - - - - - - - - - - - - - - - - - - - - - - - - - -
010 YP_009268869.1    - - - - - - - - - - - - - - - - - - - - - - - - - - - - - - - - - - - - - - - - - - - - - - - - - -
011 YP_009506251.1    - - - - - - - - - - - - - - - - - - - - - - - - - - - - - - - - - - - - - - - - - - - - - - - - - -
012 NP_955611.1       - - - - - - - - - - - - - - - - - - - - - - - - - - - - - - - - - - - - - - - - - - - - - - - - - -
013 YP_008567619.1    - - - - - - - - - - - - - - - - - - - - - - - - - - - - - - - - - - - - - - - - - - - - - - - - - -
014 NP_149413.1       - - - - - - - - - - - - - - - - - - - - - - - - - - - - - - - - - - - - - - - - - - - - - - - - - -
015 YP_004442824.1    - - - - - - - - - - - - - - - - - - - - - - - - - - - - - - - - - - - - - - - - - - - - - - - - - -
016 YP_001931967.1    - - - - - - - - - - - - - - - - - - - - - - - - - - - - - - - - - - - - - - - - - - - - - - - - - -
017 YP_002455786.1    - - - - - - - - - - - - - - - - - - - - - - - - - - - - - - - - - - - - - - - - - - - - - - - - - -
018 YP_009109694.1    - - - - - - - - - - - - - - - - - - - - - - - - - - - - - - - - - - - - - - - - - - - - - - - - - -
019 NP_789739.1       - - - - - - - - - - - - - - - - - - - - - - - - - - - - - - - - - - - - - - - - - - - - - - - - - -
020 NP_955619.1       - - - - - - - - - - - - - - - - - - - - - - - - - - - - - - - - - - - - - - - - - - - - - - - - - -
021 YP_224289.1       - - - - - - - - - - - - - - - - - - - - - - - - - - - - - - - - - - - - - - - - - - - - - - - - - -
022 YP_009182100.1    - - - - - - - - - - - - - - - - - - - - - - - - - - - - - - - - - - - - - - - - - - - - - - - - - -
023 NP_705927.1       - - - - - - - - - - - - - - - - - - - - - - - - - - - - - - - - - - - - - - - - - - - - - - - - - -
024 YP_009508577.1    - - - - - - - - - - - - - - - - - - - - - - - - - - - - - - - - - - - - - - - - - - - - - - - - - -
025 YP_004442830.1    - - - - - - - - - - - - - - - - - - - - - - - - - - - - - - - - - - - - - - - - - - - - - - - - - -
026 NP_758887.1       - - - - - - - - - - - - - - - - - - - - - - - - - - - - - - - - - - - - - - - - - - - - - - - - - -
027 YP_009508406.1    - - - - - - - - - - - - - - - - - - - - - - - - - - - - - - - - - - - - - - - - - - - - - - - - - -
028 YP_610965.1       - - - - - - - - - - - - - - - - - - - - - - - - - - - - - - - - - - - - - - - - - - - - - - - - - -
029 YP_001931961.1    - - - - - - - - - - - - - - - - - - - - - - - - - - - - - - - - - - - - - - - - - - - - - - - - - -
030 YP_241114.1       - - - - - - - - - - - - - - - - - - - - - - - - - - - - - - - - - - - - - - - - - - - - - - - - - -
031 YP_006732334.1    - - - - - - - - - - - - - - - - - - - - - - - - - - - - - - - - - - - - - - - - - - - - - - - - - -
032 YP_009041481.1    - - - - - - - - - - - - - - - - - - - - - - - - - - - - - - - - - - - - - - - - - - - - - - - - - -
033 YP_001036293.1    - - - - - - - - - - - - - - - - - - - - - - - - - - - - - - - - - - - - - - - - - - - - - - - - - -
034 YP_605811.1       - - - - - - - - - - - - - - - - - - - - - - - - - - - - - - - - - - - - - - - - - - - - - - - - - -
035 NP_955577.1       - - - - - - - - - - - - - - - - - - - - - - - - - - - - - - - - - - - - - - - - - - - - - - - - - -
036 YP_003208050.1    - - - - - - - - - - - - - - - - - - - - - - - - - - - - - - - - - - - - - - - - - - - - - - - - - -
037 NP_789740.1       - - - - - - - - - - - - - - - - - - - - - - - - - - - - - - - - - - - - - - - - - - - - - - - - - -
038 YP_004442839.1    - - - - - - - - - - - - - - - - - - - - - - - - - - - - - - - - - - - - - - - - - - - - - - - - - -
039 YP_009507791.1    - - - - - - - - - - - - - - - - - - - - - - - - - - - - - - - - - - - - - - - - - - - - - - - - - -
040 YP_004581513.1    - - - - - - - - - - - - - - - - - - - - - - - - - - - - - - - - - - - - - - - - - - - - - - - - - -
041 NP_043933.1       - - - - - - - - - - - - - - - - - - - - - - - - - - - - - - - - - - - - - - - - - - - - - - - - - -
042 YP_009507248.1    - - - - - - - - - - - - - - - - - - - - - - - - - - - - - - - - - - - - - - - - - - - - - - - - - -
043 YP_002117531.1    - - - - - - - - - - - - - - - - - - - - - - - - - - - - - - - - - - - - - - - - - - - - - - - - - -
044 YP_004347415.1    - - - - - - - - - - - - - - - - - - - - - - - - - - - - - - - - - - - - - - - - - - - - - - - - - -
045 NP_056891.1       A V A Q T E E I L K S N S Q T D L T K T S Q N P D L D L I S L D S D D E G A K S S S L Q D K G L S S
```

```
046 YP_009513242.1    --------------------------------------------------
047 YP_009352866.1    --------------------------------------------------
048 YP_009165750.1    --------------------------------------------------
049 NP_040840.1       --------------------------------------------------
050 YP_004222728.1    --------------------------------------------------
051 YP_009506264.1    --------------------------------------------------
052 YP_567050.1       --------------------------------------------------
053 YP_009508571.1    --------------------------------------------------
054 NP_056803.1       --------------------------------------------------
055 NP_955564.1       --------------------------------------------------
056 YP_009508411.1    --------------------------------------------------
057 YP_009140788.1    --------------------------------------------------
058 YP_009508410.1    --------------------------------------------------
059 NP_040333.1       --------------------------------------------------
060 YP_008992013.1    --------------------------------------------------
061 NP_041734.1       --------------------------------------------------
062 NP_056895.1       --------------------------------------------------
063 YP_009109689.1    PDQVAYIITWEDLVRNPPPWVKPFLHTPSTSKSTLLALEVPKNRT-----
064 YP_002519387.1    --------------------------------------------------
065 YP_595725.1       --------------------------------------------------
066 YP_009508551.1    --------------------------------------------------
067 YP_001497148.1    PDQIAYILTWEDLIRNPPAWVKPFLPSCPLSQSTLLPLKTSKDRASTQPS
068 NP_041186.1       --------------------------------------------------
069 NP_663784.1       --------------------------------------------------
070 YP_009508443.1    --------------------------------------------------
071 YP_003987465.1    --------------------------------------------------
072 NP_861410.1       --------------------------------------------------
073 YP_009345071.1    --------------------------------------------------
074 YP_009508582.1    --------------------------------------------------
075 NP_569141.1       --------------------------------------------------
076 YP_002916057.1    --------------------------------------------------
077 NP_777384.2       --------------------------------------------------
078 YP_233110.1       --------------------------------------------------
079 NP_542258.1       --------------------------------------------------
080 YP_009130664.1    --------------------------------------------------
081 YP_009508566.1    --------------------------------------------------
082 YP_006607892.1    --------------------------------------------------
083 YP_009506270.1    --------------------------------------------------
084 NP_042513.1       --------------------------------------------------
085 YP_006907834.1    --------------------------------------------------
086 YP_004442827.1    --------------------------------------------------
087 YP_009408594.1    --------------------------------------------------
088 YP_009508561.1    --------------------------------------------------
089 NP_056907.1       --------------------------------------------------
090 YP_002308474.1    --------------------------------------------------
091 YP_007761644.1    --------------------------------------------------
092 NP_040550.1       --------------------------------------------------
093 NP_758808.1       --------------------------------------------------
094 YP_009345075.1    --------------------------------------------------
095 NP_068729.1       --------------------------------------------------
096 NP_047255.1       PDQVPYITTWRSLATDPPSWVRPFLPPPKPPTP-----------------
097 YP_006273075.1    --------------------------------------------------
098 YP_009508888.1    --------------------------------------------------
099 NP_395469.1       --------------------------------------------------
100 YP_009508546.1    --------------------------------------------------
101 NP_056762.1       --------------------------------------------------
102 YP_009508556.1    --------------------------------------------------
103 NP_569153.1       --------------------------------------------------
104 NP_043924.1       --------------------------------------------------
105 YP_009243641.1    --------------------------------------------------
106 YP_004300274.1    --------------------------------------------------
107 NP_056848.1       --------------------------------------------------
108 YP_006495799.1    --------------------------------------------------
109 YP_009508409.1    --------------------------------------------------
110 NP_687035.1       --------------------------------------------------
111 NP_045937.2       --------------------------------------------------
112 NP_612577.1       --------------------------------------------------
113 NP_619548.1       --------------------------------------------------
114 NP_056880.1       EAKSVTEEELEEGLTGLLSTSSQEKT----YGTRGTAYAEIDTEVDKLSE
115 YP_009121747.1    --------------------------------------------------
116 YP_009113237.2    --------------------------------------------------
117 YP_009508408.1    --------------------------------------------------
118 NP_955579.1       --------------------------------------------------
```

```
119 YP_009229919.1      --------------------------------------------------
120 YP_004442833.1      --------------------------------------------------
121 NP_862833.2         --------------------------------------------------
122 NP_040563.1         --------------------------------------------------
123 YP_443922.1         QTPTQRLYPDLPEDSRPPPYSPSASPSPTVQAPVLTVRGGVIKGEVHLVE
124 NP_057933.2         PDQVPYIVTWEALAFDPPPWVKPFVHPKPPPPL-----------------
125 YP_009116631.1      --------------------------------------------------
126 YP_009513249.1      --------------------------------------------------
127 YP_223871.1         --------------------------------------------------
128 NP_127504.1         --------------------------------------------------
129 NP_044929.1         --------------------------------------------------
130 NP_057860.1         --------------------------------------------------
131 NP_040973.1         --------------------------------------------------
132 NP_777317.1         --------------------------------------------------
133 NP_054716.1         --------------------------------------------------
134 YP_001856242.1      --------------------------------------------------
135 NP_954565.2         AVAQTEEILKSNSQTDLTKTSQNPDLDLISLDSDDEGAKSSSLQDKGLSS
136 NP_569150.1         --------------------------------------------------
137 NP_659397.1         --------------------------------------------------
138 YP_009513211.1      --------------------------------------------------
139 YP_233107.1         --------------------------------------------------
140 YP_009109692.1      --------------------------------------------------
141 NP_057849.4         --------------------------------------------------
142 NP_049560.1         --------------------------------------------------
143 YP_009506267.1      --------------------------------------------------
144 NP_039820.1         --------------------------------------------------
145 NP_056790.1         --------------------------------------------------
146 YP_002321513.1      --------------------------------------------------
147 NP_955591.1         --------------------------------------------------
148 YP_001956722.2      --------------------------------------------------
149 NP_056902.1         --------------------------------------------------
150 NP_056886.1         --------------------------------------------------
151 NP_041261.1         --------------------------------------------------
```

```
001 YP_004442836.1      --------------------------------------------------
002 NP_056728.1         --------------------------------------------------
003 YP_009508537.1      --------------------------------------------------
004 YP_004732983.2      --------------------------------------------------
005 YP_009508407.1      --------------------------------------------------
006 NP_040939.1         --------------------------------------------------
007 YP_003864102.1      --------------------------------------------------
008 YP_009002585.1      --------------------------------------------------
009 YP_003284237.1      --------------------------------------------------
010 YP_009268869.1      --------------------------------------------------
011 YP_009506251.1      --------------------------------------------------
012 NP_955611.1         --------------------------------------------------
013 YP_008567619.1      --------------------------------------------------
014 NP_149413.1         --------------------------------------------------
015 YP_004442824.1      --------------------------------------------------
016 YP_001931967.1      --------------------------------------------------
017 YP_002455786.1      --------------------------------------------------
018 YP_009109694.1      --------------------------------------------------
019 NP_789739.1         --------------------------------------------------
020 NP_955619.1         --------------------------------------------------
021 YP_224289.1         --------------------------------------------------
022 YP_009182100.1      --------------------------------------------------
023 NP_705927.1         --------------------------------------------------
024 YP_009508577.1      --------------------------------------------------
025 YP_004442830.1      --------------------------------------------------
026 NP_758887.1         --------------------------------------------------
027 YP_009508406.1      --------------------------------------------------
028 YP_610965.1         --------------------------------------------------
029 YP_001931961.1      --------------------------------------------------
030 YP_241114.1         --------------------------------------------------
031 YP_006732334.1      --------------------------------------------------
032 YP_009041481.1      --------------------------------------------------
033 YP_001036293.1      --------------------------------------------------
034 YP_605811.1         --------------------------------------------------
035 NP_955577.1         --------------------------------------------------
036 YP_003208050.1      --------------------------------------------------
037 NP_789740.1         --------------------------------------------------
```

038	YP_004442839.1	--
039	YP_009507791.1	--
040	YP_004581513.1	--
041	NP_043933.1	--
042	YP_009507248.1	--
043	YP_002117531.1	--
044	YP_004347415.1	--
045	NP_056891.1	TKKPKRFPVLLTAQTSKDPEDPNPSEVDWDGLEDEAAKYHNPDWPPFLTR
046	YP_009513242.1	--
047	YP_009352866.1	--
048	YP_009165750.1	--
049	NP_040840.1	--
050	YP_004222728.1	--
051	YP_009506264.1	--
052	YP_567050.1	--
053	YP_009508571.1	--
054	NP_056803.1	--
055	NP_955564.1	--
056	YP_009508411.1	--
057	YP_009140788.1	--
058	YP_009508410.1	--
059	NP_040333.1	--
060	YP_008992013.1	--
061	NP_041734.1	--
062	NP_056895.1	--
063	YP_009109689.1	--LDPPKPVLPDESQQDLLFQDPLPHPPHNPLLEPPPYNSPSPPVLSPVS
064	YP_002519387.1	--
065	YP_595725.1	--
066	YP_009508551.1	--
067	YP_001497148.1	APPKPPKPVLPDESQKDPPLLDALSSPPHNPLLQPPPYNPPLAPALTPVG
068	NP_041186.1	--
069	NP_663784.1	--
070	YP_009508443.1	--
071	YP_003987465.1	--
072	NP_861410.1	--
073	YP_009345071.1	--
074	YP_009508582.1	--
075	NP_569141.1	--
076	YP_002916057.1	--
077	NP_777384.2	--
078	YP_233110.1	--
079	NP_542258.1	--
080	YP_009130664.1	--
081	YP_009508566.1	--
082	YP_006607892.1	--
083	YP_009506270.1	--
084	NP_042513.1	--
085	YP_006907834.1	--
086	YP_004442827.1	--
087	YP_009408594.1	--
088	YP_009508561.1	--
089	NP_056907.1	--
090	YP_002308474.1	--
091	YP_007761644.1	--
092	NP_040550.1	--
093	NP_758808.1	--
094	YP_009345075.1	--
095	NP_068729.1	--
096	NP_047255.1	------LPQP-----LSPQPSAPLTSSLYPVLPKSDPPKPPVLPPDPSSP
097	YP_006273075.1	--
098	YP_009508888.1	--
099	NP_395469.1	--
100	YP_009508546.1	--
101	NP_056762.1	--
102	YP_009508556.1	--
103	NP_569153.1	--
104	NP_043924.1	--
105	YP_009243641.1	--
106	YP_004300274.1	--
107	NP_056848.1	--
108	YP_006495799.1	--
109	YP_009508409.1	--
110	NP_687035.1	--

04/06/2019 ConSurf Color-Coded MSA

```
111 NP_045937.2       --------------------------------------------------
112 NP_612577.1       --------------------------------------------------
113 NP_619548.1       --------------------------------------------------
114 NP_056880.1       HIYDEPYEEKEKADKNEEKDHVRKIKKVVQRKENSEGKRKEKDSKAFLAT
115 YP_009121747.1    --------------------------------------------------
116 YP_009113237.2    --------------------------------------------------
117 YP_009508408.1    --------------------------------------------------
118 NP_955579.1       --------------------------------------------------
119 YP_009229919.1    --------------------------------------------------
120 YP_004442833.1    --------------------------------------------------
121 NP_862833.2       --------------------------------------------------
122 NP_040563.1       --------------------------------------------------
123 YP_443922.1       GTIDVENSEPPQRSMDTDDLGPAGGRSTRGPDSLEEGSMRTQSSKRTRNP
124 NP_057933.2       ------PPSAPSLPLEPPRSTPPRSSLYPALTPSLGAKPKPQVLSDSGGP
125 YP_009116631.1    --------------------------------------------------
126 YP_009513249.1    --------------------------------------------------
127 YP_223871.1       --------------------------------------------------
128 NP_127504.1       --------------------------------------------------
129 NP_044929.1       --------------------------------------------------
130 NP_057860.1       --------------------------------------------------
131 NP_040973.1       --------------------------------------------------
132 NP_777317.1       --------------------------------------------------
133 NP_054716.1       --------------------------------------------------
134 YP_001856242.1    --------------------------------------------------
135 NP_954565.2       TKKPKRFPVLLTAQTSKDPEDPNPSEVDWDGLEDEAAKYHNPDWPPFLTR
136 NP_569150.1       --------------------------------------------------
137 NP_659397.1       --------------------------------------------------
138 YP_009513211.1    --------------------------------------------------
139 YP_233107.1       --------------------------------------------------
140 YP_009109692.1    --------------------------------------------------
141 NP_057849.4       --------------------------------------------------
142 NP_049560.1       --------------------------------------------------
143 YP_009506267.1    --------------------------------------------------
144 NP_039820.1       --------------------------------------------------
145 NP_056790.1       --------------------------------------------------
146 YP_002321513.1    --------------------------------------------------
147 NP_955591.1       --------------------------------------------------
148 YP_001956722.2    --------------------------------------------------
149 NP_056902.1       --------------------------------------------------
150 NP_056886.1       --------------------------------------MEAVIKVISSAC
151 NP_041261.1       --------------------------------------------------

001 YP_004442836.1    --------------------------------------------------
002 NP_056728.1       --------------------------------------------------
003 YP_009508537.1    --------------------------------------------------
004 YP_004732983.2    --------------------------------------------------
005 YP_009508407.1    --------------------------------------------------
006 NP_040939.1       --------------------------------------------------
007 YP_003864102.1    --------------------------------------------------
008 YP_009002585.1    --------------------------------------------------
009 YP_003284237.1    --------------------------------------------------
010 YP_009268869.1    --------------------------------------------------
011 YP_009506251.1    --------------------------------------------------
012 NP_955611.1       --------------------------------------------------
013 YP_008567619.1    --------------------------------------------------
014 NP_149413.1       --------------------------------------------------
015 YP_004442824.1    --------------------------------------------------
016 YP_001931967.1    --------------------------------------------------
017 YP_002455786.1    --------------------------------------------------
018 YP_009109694.1    --------------------------------------------------
019 NP_789739.1       --------------------------------------------------
020 NP_955619.1       --------------------------------------------------
021 YP_224289.1       --------------------------------------------------
022 YP_009182100.1    --------------------------------------------------
023 NP_705927.1       --------------------------------------------------
024 YP_009508577.1    --------------------------------------------------
025 YP_004442830.1    --------------------------------------------------
026 NP_758887.1       --------------------------------------------------
027 YP_009508406.1    --------------------------------------------------
028 YP_610965.1       --------------------------------------------------
029 YP_001931961.1    --------------------------------------------------
```

http://consurf.tau.ac.il/results/1551978523/Multicolored_MSA.html 11/172

```
030 YP_241114.1      --------------------------------------------------
031 YP_006732334.1   --------------------------------------------------
032 YP_009041481.1   --------------------------------------------------
033 YP_001036293.1   --------------------------------------------------
034 YP_605811.1      --------------------------------------------------
035 NP_955577.1      --------------------------------------------------
036 YP_003208050.1   --------------------------------------------------
037 NP_789740.1      --------------------------------------------------
038 YP_004442839.1   --------------------------------------------------
039 YP_009507791.1   --------------------------------------------------
040 YP_004581513.1   --------------------------------------------------
041 NP_043933.1      --------------------------------------------------
042 YP_009507248.1   --------------------------------------------------
043 YP_002117531.1   --------------------------------------------------
044 YP_004347415.1   --------------------------------------------------
045 NP_056891.1      PPPYNKATPSAPTVMAVVNPKEELKEKIAQLEEQIKLEELHQALISKLQK
046 YP_009513242.1   --------------------------------------------------
047 YP_009352866.1   --------------------------------------------------
048 YP_009165750.1   --------------------------------------------------
049 NP_040840.1      --------------------------------------------------
050 YP_004222728.1   --------------------------------------------------
051 YP_009506264.1   --------------------------------------------------
052 YP_567050.1      --------------------------------------------------
053 YP_009508571.1   --------------------------------------------------
054 NP_056803.1      --------------------------------------------------
055 NP_955564.1      --------------------------------------------------
056 YP_009508411.1   --------------------------------------------------
057 YP_009140788.1   --------------------------------------------------
058 YP_009508410.1   --------------------------------------------------
059 NP_040333.1      --------------------------------------------------
060 YP_008992013.1   --------------------------------------------------
061 NP_041734.1      --------------------------------------------------
062 NP_056895.1      --------------------------------------------------
063 YP_009109689.1   PTTPSAPTPSSLVSSST------PPSSPAPPELTPRTPPQTPRLRLRRAE
064 YP_002519387.1   --------------------------------------------------
065 YP_595725.1      --------------------------------------------------
066 YP_009508551.1   --------------------------------------------------
067 YP_001497148.1   PTSPPASSSSSLSPTSSPACTSAPSSTPAPPDLTPQTPPQTPRLRLRRLD
068 NP_041186.1      --------------------------------------------------
069 NP_663784.1      --------------------------------------------------
070 YP_009508443.1   --------------------------------------------------
071 YP_003987465.1   --------------------------------------------------
072 NP_861410.1      --------------------------------------------------
073 YP_009345071.1   --------------------------------------------------
074 YP_009508582.1   --------------------------------------------------
075 NP_569141.1      --------------------------------------------------
076 YP_002916057.1   --------------------------------------------------
077 NP_777384.2      --------------------------------------------------
078 YP_233110.1      --------------------------------------------------
079 NP_542258.1      --------------------------------------------------
080 YP_009130664.1   --------------------------------------------------
081 YP_009508566.1   --------------------------------------------------
082 YP_006607892.1   --------------------------------------------------
083 YP_009506270.1   --------------------------------------------------
084 NP_042513.1      --------------------------------------------------
085 YP_006907834.1   --------------------------------------------------
086 YP_004442827.1   --------------------------------------------------
087 YP_009408594.1   --------------------------------------------------
088 YP_009508561.1   --------------------------------------------------
089 NP_056907.1      --------------------------------------------------
090 YP_002308474.1   --------------------------------------------------
091 YP_007761644.1   --------------------------------------------------
092 NP_040550.1      --------------------------------------------------
093 NP_758808.1      --------------------------------------------------
094 YP_009345075.1   --------------------------------------------------
095 NP_068729.1      --------------------------------------------------
096 NP_047255.1      LIDLLTEEPPPYPGGHGPPPSGPRTPTASP-------------IASRLRE
097 YP_006273075.1   --------------------------------------------------
098 YP_009508888.1   --------------------------------------------------
099 NP_395469.1      --------------------------------------------------
100 YP_009508546.1   --------------------------------------------------
101 NP_056762.1      --------------------------------------------------
102 YP_009508556.1   --------------------------------------------------
```

```
103 NP_569153.1          --------------------------------------------------
104 NP_043924.1          --------------------------------------------------
105 YP_009243641.1       --------------------------------------------------
106 YP_004300274.1       --------------------------------------------------
107 NP_056848.1          --------------------------------------------------
108 YP_006495799.1       --------------------------------------------------
109 YP_009508409.1       --------------------------------------------------
110 NP_687035.1          --------------------------------------------------
111 NP_045937.2          --------------------------------------------------
112 NP_612577.1          --------------------------------------------------
113 NP_619548.1          --------------------------------------------------
114 NP_056880.1          DWNDDDLSPEDWDDLEEQAAHYHDDDELILPVKRKVVKKKPQALRRKPLP
115 YP_009121747.1       --------------------------------------------------
116 YP_009113237.2       --------------------------------------------------
117 YP_009508408.1       --------------------------------------------------
118 NP_955579.1          --------------------------------------------------
119 YP_009229919.1       --------------------------------------------------
120 YP_004442833.1       --------------------------------------------------
121 NP_862833.2          --------------------------------------------------
122 NP_040563.1          --------------------------------------------------
123 YP_443922.1          FLDQPFSSPPPSGSLSYRPLPQSLPSPLSRAVTTPHSYTGEHQQLRQNDI
124 NP_057933.2          LIDLLTEDPPPYRDPRPPPSDRDGNGGEATPAGEAPDPSPMASRLRGRRE
125 YP_009116631.1       --------------------------------------------------
126 YP_009513249.1       --------------------------------------------------
127 YP_223871.1          --------------------------------------------------
128 NP_127504.1          --------------------------------------------------
129 NP_044929.1          --------------------------------------------------
130 NP_057860.1          --------------------------------------------------
131 NP_040973.1          --------------------------------------------------
132 NP_777317.1          --------------------------------------------------
133 NP_054716.1          --------------------------------------------------
134 YP_001856242.1       --------------------------------------------------
135 NP_954565.2          PPPYNKATPSAPTVMAVVNPKEELKEKIAQLEEQIKLEELHQALISKLQK
136 NP_569150.1          --------------------------------------------------
137 NP_659397.1          --------------------------------------------------
138 YP_009513211.1       --------------------------------------------------
139 YP_233107.1          --------------------------------------------------
140 YP_009109692.1       --------------------------------------------------
141 NP_057849.4          --------------------------------------------------
142 NP_049560.1          --------------------------------------------------
143 YP_009506267.1       --------------------------------------------------
144 NP_039820.1          --------------------------------------------------
145 NP_056790.1          --------------------------------------------------
146 YP_002321513.1       --------------------------------------------------
147 NP_955591.1          --------------------------------------------------
148 YP_001956722.2       --------------------------------------------------
149 NP_056902.1          --------------------------------------------------
150 NP_056886.1          KTYCGKTSPSKKEIGAMLSLLQKEGLLMSPSDLYSPGSWDPITAALSQRA
151 NP_041261.1          --------------------------------------------------
```

```
001 YP_004442836.1       --------------------------------------------------
002 NP_056728.1          --------------------------------------------------
003 YP_009508537.1       --------------------------------------------------
004 YP_004732983.2       --------------------------------------------------
005 YP_009508407.1       --------------------------------------------------
006 NP_040939.1          --------------------------------------------------
007 YP_003864102.1       --------------------------------------------------
008 YP_009002585.1       --------------------------------------------------
009 YP_003284237.1       --------------------------------------------------
010 YP_009268869.1       --------------------------------------------------
011 YP_009506251.1       --------------------------------------------------
012 NP_955611.1          --------------------------------------------------
013 YP_008567619.1       --------------------------------------------------
014 NP_149413.1          --------------------------------------------------
015 YP_004442824.1       --------------------------------------------------
016 YP_001931967.1       --------------------------------------------------
017 YP_002455786.1       --------------------------------------------------
018 YP_009109694.1       --------------------------------------------------
019 NP_789739.1          --------------------------------------------------
020 NP_955619.1          --------------------------------------------------
021 YP_224289.1          --------------------------------------------------
```

```
022 YP_009182100.1   --------------------------------------------------
023 NP_705927.1      --------------------------------------------------
024 YP_009508577.1   --------------------------------------------------
025 YP_004442830.1   --------------------------------------------------
026 NP_758887.1      --------------------------------------------------
027 YP_009508406.1   --------------------------------------------------
028 YP_610965.1      --------------------------------------------------
029 YP_001931961.1   --------------------------------------------------
030 YP_241114.1      --------------------------------------------------
031 YP_006732334.1   --------------------------------------------------
032 YP_009041481.1   --------------------------------------------------
033 YP_001036293.1   --------------------------------------------------
034 YP_605811.1      --------------------------------------------------
035 NP_955577.1      --------------------------------------------------
036 YP_003208050.1   --------------------------------------------------
037 NP_789740.1      --------------------------------------------------
038 YP_004442839.1   --------------------------------------------------
039 YP_009507791.1   --------------------------------------------------
040 YP_004581513.1   --------------------------------------------------
041 NP_043933.1      --------------------------------------------------
042 YP_009507248.1   --------------------------------------------------
043 YP_002117531.1   --------------------------------------------------
044 YP_004347415.1   --------------------------------------------------
045 NP_056891.1      LKTGNETVTHPDTAGGLSRTPHWPGQHIPKGKCCASREKEEQIPKDIFPV
046 YP_009513242.1   --------------------------------------------------
047 YP_009352866.1   --------------------------------------------------
048 YP_009165750.1   --------------------------------------------------
049 NP_040840.1      --------------------------------------------------
050 YP_004222728.1   --------------------------------------------------
051 YP_009506264.1   --------------------------------------------------
052 YP_567050.1      --------------------------------------------------
053 YP_009508571.1   --------------------------------------------------
054 NP_056803.1      --------------------------------------------------
055 NP_955564.1      --------------------------------------------------
056 YP_009508411.1   --------------------------------------------------
057 YP_009140788.1   --------------------------------------------------
058 YP_009508410.1   --------------------------------------------------
059 NP_040333.1      --------------------------------------------------
060 YP_008992013.1   --------------------------------------------------
061 NP_041734.1      --------------------------------------------------
062 NP_056895.1      --------------------------------------------------
063 YP_009109689.1   GQDGPSTWQSSLFPLRTVNRTIQYWPFSASDLYNWKTHNPSFSQDPQALT
064 YP_002519387.1   --------------------------------------------------
065 YP_595725.1      --------------------------------------------------
066 YP_009508551.1   --------------------------------------------------
067 YP_001497148.1   DPNGPPTWQSSLFPLRTVNRTVQYWPFSASDLYNWKTHNPSFSQEPQALT
068 NP_041186.1      --------------------------------------------------
069 NP_663784.1      -----------MGARNSVLRGKKADELEKVRLRPGGKKKYRLKHIVWAAN
070 YP_009508443.1   --------------------------------------------------
071 YP_003987465.1   --------------------------------------------------
072 NP_861410.1      --------------------------------------------------
073 YP_009345071.1   --------------------------------------------------
074 YP_009508582.1   --------------------------------------------------
075 NP_569141.1      --------------------------------------------------
076 YP_002916057.1   --------------------------------------------------
077 NP_777384.2      --------------------------------------------------
078 YP_233110.1      --------------------------------------------------
079 NP_542258.1      --------------------------------------------------
080 YP_009130664.1   --------------------------------------------------
081 YP_009508566.1   --------------------------------------------------
082 YP_006607892.1   --------------------------------------------------
083 YP_009506270.1   --------------------------------------------------
084 NP_042513.1      --------------------------------------------------
085 YP_006907834.1   --------------------------------------------------
086 YP_004442827.1   --------------------------------------------------
087 YP_009408594.1   --------------------------------------------------
088 YP_009508561.1   --------------------------------------------------
089 NP_056907.1      --------------------------------------------------
090 YP_002308474.1   --------------------------------------------------
091 YP_007761644.1   --------------------------------------------------
092 NP_040550.1      --------------------------------------------------
093 NP_758808.1      --------------------------------------------------
094 YP_009345075.1   --------------------------------------------------
```

```
095 NP_068729.1        --------------------------------------------------
096 NP_047255.1        RRENPAEESQALPLREGPNNRPQYWPFSASDLYNWKSHNPPFSQDPVALT
097 YP_006273075.1     --------------------------------------------------
098 YP_009508888.1     --------------------------------------------------
099 NP_395469.1        --------------------------------------------------
100 YP_009508546.1     --------------------------------------------------
101 NP_056762.1        --------------------------------------------------
102 YP_009508556.1     --------------------------------------------------
103 NP_569153.1        --------------------------------------------------
104 NP_043924.1        --------------------------------------------------
105 YP_009243641.1     --------------------------------------------------
106 YP_004300274.1     --------------------------------------------------
107 NP_056848.1        --------------------------------------------------
108 YP_006495799.1     --------------------------------------------------
109 YP_009508409.1     --------------------------------------------------
110 NP_687035.1        -----------MGGGHSALSGRSLDTFEKIRLRPNGKKKYQIKHLIWAGK
111 NP_045937.2        --------------------------------------------------
112 NP_612577.1        --------------------------------------------------
113 NP_619548.1        --------------------------------------------------
114 NP_056880.1        PVGFAGAMAEAREKGDLTFT---------------------------FPV
115 YP_009121747.1     --------------------------------------------------
116 YP_009113237.2     --------------------------------------------------
117 YP_009508408.1     --------------------------------------------------
118 NP_955579.1        --------------------------------------------------
119 YP_009229919.1     --------------------------------------------------
120 YP_004442833.1     --------------------------------------------------
121 NP_862833.2        --------------------------------------------------
122 NP_040563.1        --------------------------------------------------
123 YP_443922.1        DREYLTSYNMPQEEDENDPAPTQEQIWTAAEQLNREQFPSGGGGPYQLRG
124 NP_057933.2        PPVADSTTSQAFPLRAGGNGQLQYWPFSSSDLYNWKNNNPSFSEDPGKLT
125 YP_009116631.1     --------------------------------------------------
126 YP_009513249.1     --------------------------------------------------
127 YP_223871.1        --------------------------------------------------
128 NP_127504.1        --------------------------------------------------
129 NP_044929.1        --------------------------------------------------
130 NP_057860.1        --------------------------------------------------
131 NP_040973.1        --------------------------------------------------
132 NP_777317.1        --------------------------------------------------
133 NP_054716.1        --------------------------------------------------
134 YP_001856242.1     --------------------------------------------------
135 NP_954565.2        LKTGNETVTHPDTAGGLSRTPHWPGQHIPKGKCCASREKEEQIPKDIFPV
136 NP_569150.1        --------------------------------------------------
137 NP_659397.1        --------------------------------------------------
138 YP_009513211.1     --------------------------------------------------
139 YP_233107.1        --------------------------------------------------
140 YP_009109692.1     --------------------------------------------------
141 NP_057849.4        -----------MGARASVLSGGELDRWEKIRLRPGGKKKYKLKHIVWASR
142 NP_049560.1        --------------------------------------------------
143 YP_009506267.1     --------------------------------------------------
144 NP_039820.1        --------------------------------------------------
145 NP_056790.1        --------------------------------------------------
146 YP_002321513.1     --------------------------------------------------
147 NP_955591.1        --------------------------------------------------
148 YP_001956722.2     --------------------------------------------------
149 NP_056902.1        --------------------------------------------------
150 NP_056886.1        MILGKSGELKTWGLVLGALKAAREEQVTSEQAKFWLGLGGGRVSPPGPEC
151 NP_041261.1        --------------------------------------------------

001 YP_004442836.1     --------------------------------------------------
002 NP_056728.1        --------------------------------------------------
003 YP_009508537.1     --------------------------------------------------
004 YP_004732983.2     --------------------------------------------------
005 YP_009508407.1     --------------------------------------------------
006 NP_040939.1        --------------------------------------------------
007 YP_003864102.1     --------------------------------------------------
008 YP_009002585.1     --------------------------------------------------
009 YP_003284237.1     --------------------------------------------------
010 YP_009268869.1     --------------------------------------------------
011 YP_009506251.1     --------------------------------------------------
012 NP_955611.1        --------------------------------------------------
013 YP_008567619.1     --------------------------------------------------
```

```
014 NP_149413.1         --------------------------------------------------
015 YP_004442824.1      --------------------------------------------------
016 YP_001931967.1      --------------------------------------------------
017 YP_002455786.1      --------------------------------------------------
018 YP_009109694.1      --------------------------------------------------
019 NP_789739.1         --------------------------------------------------
020 NP_955619.1         --------------------------------------------------
021 YP_224289.1         --------------------------------------------------
022 YP_009182100.1      --------------------------------------------------
023 NP_705927.1         --------------------------------------------------
024 YP_009508577.1      --------------------------------------------------
025 YP_004442830.1      --------------------------------------------------
026 NP_758887.1         --------------------------------------------------
027 YP_009508406.1      --------------------------------------------------
028 YP_610965.1         --------------------------------------------------
029 YP_001931961.1      --------------------------------------------------
030 YP_241114.1         --------------------------------------------------
031 YP_006732334.1      --------------------------------------------------
032 YP_009041481.1      --------------------------------------------------
033 YP_001036293.1      --------------------------------------------------
034 YP_605811.1         --------------------------------------------------
035 NP_955577.1         --------------------------------------------------
036 YP_003208050.1      --------------------------------------------------
037 NP_789740.1         --------------------------------------------------
038 YP_004442839.1      --------------------------------------------------
039 YP_009507791.1      --------------------------------------------------
040 YP_004581513.1      --------------------------------------------------
041 NP_043933.1         --------------------------------------------------
042 YP_009507248.1      --------------------------------------------------
043 YP_002117531.1      --------------------------------------------------
044 YP_004347415.1      --------------------------------------------------
045 NP_056891.1         TETVDGQGQAWRHHNGFDFAVIKELKTAASQYGATAPYTLAIVESVADNW
046 YP_009513242.1      --------------------------------------------------
047 YP_009352866.1      --------------------------------------------------
048 YP_009165750.1      --------------------------------------------------
049 NP_040840.1         --------------------------------------------------
050 YP_004222728.1      --------------------------------------------------
051 YP_009506264.1      --------------------------------------------------
052 YP_567050.1         --------------------------------------------------
053 YP_009508571.1      --------------------------------------------------
054 NP_056803.1         --------------------------------------------------
055 NP_955564.1         --------------------------------------------------
056 YP_009508411.1      --------------------------------------------------
057 YP_009140788.1      --------------------------------------------------
058 YP_009508410.1      --------------------------------------------------
059 NP_040333.1         --------------------------------------------------
060 YP_008992013.1      --------------------------------------------------
061 NP_041734.1         --------------------------------------------------
062 NP_056895.1         -----------------MGNSPSYNPPAGISPSDWLNLRQSAQRLNPRPS
063 YP_009109689.1      SLIESILLTHQPTWDDCQQLLQVLLTTEERQRVLLEARKNVPGPGGLPTQ
064 YP_002519387.1      --------------------------------------------------
065 YP_595725.1         --------------------------------------------------
066 YP_009508551.1      --------------------------------------------------
067 YP_001497148.1      SLIESILLTHQPTWDDCQQLLQVLLTTEERQRVLLEARKNVPGPGGFPTQ
068 NP_041186.1         --------------------------------------------------
069 NP_663784.1         ELDKFGLAESLLESKEGCQKILRVLDPLVPTGSENLKSLFNTVCVIWCLH
070 YP_009508443.1      --------------------------------------------------
071 YP_003987465.1      --------------------------------------------------
072 NP_861410.1         --------------------------------------------------
073 YP_009345071.1      --------------------------------------------------
074 YP_009508582.1      --------------------------------------------------
075 NP_569141.1         --------------------------------------------------
076 YP_002916057.1      --------------------------------------------------
077 NP_777384.2         -----------------MGNSPSYNPPAGISPSDWLNLRQSAQRLNPRPS
078 YP_233110.1         --------------------------------------------------
079 NP_542258.1         --------------------------------------------------
080 YP_009130664.1      --------------------------------------------------
081 YP_009508566.1      --------------------------------------------------
082 YP_006607892.1      --------------------------------------------------
083 YP_009506270.1      --------------------------------------------------
084 NP_042513.1         --------------------------------------------------
085 YP_006907834.1      --------------------------------------------------
086 YP_004442827.1      --------------------------------------------------
```

```
087 YP_009408594.1    --------------------------------------------------
088 YP_009508561.1    --------------------------------------------------
089 NP_056907.1       -----------MGQTYGLSSSPIPKAPRGLSTHHWLNFLQAAYRLQPGPS
090 YP_002308474.1    --------------------------------------------------
091 YP_007761644.1    --------------------------------------------------
092 NP_040550.1       --------------------------------------------------
093 NP_758808.1       --------------------------------------------------
094 YP_009345075.1    --------------------------------------------------
095 NP_068729.1       --------------------------------------------------
096 NP_047255.1       NLIESILVTHQPTWDDCQQLLQALLTGEERQRVLLEARKQVPGEDGRPTQ
097 YP_006273075.1    --------------------------------------------------
098 YP_009508888.1    --------------------------------------------------
099 NP_395469.1       --------------------------------------------------
100 YP_009508546.1    --------------------------------------------------
101 NP_056762.1       --------------------------------------------------
102 YP_009508556.1    --------------------------------------------------
103 NP_569153.1       --------------------------------------------------
104 NP_043924.1       --------------------------------------------------
105 YP_009243641.1    --------------------------------------------------
106 YP_004300274.1    --------------------------------------------------
107 NP_056848.1       --------------------------------------------------
108 YP_006495799.1    --------------------------------------------------
109 YP_009508409.1    --------------------------------------------------
110 NP_687035.1       EMERFGLHEKLLETKEGCQKIIEVLTPLEPTGSEGLKALFNLCCVIWCIH
111 NP_045937.2       --------------------------------------------------
112 NP_612577.1       --------------------------------------------------
113 NP_619548.1       --------------------------------------------------
114 NP_056880.1       VFMGESDEDDTPVWEPLPLKTLKELQSAVRTMGPSAPYTLQVVDMVASQW
115 YP_009121747.1    --------------------------------------------------
116 YP_009113237.2    --------------------------------------------------
117 YP_009508408.1    --------------------------------------------------
118 NP_955579.1       --------------------------------------------------
119 YP_009229919.1    --------------------------------------------------
120 YP_004442833.1    --------------------------------------------------
121 NP_862833.2       --------------------------------------------------
122 NP_040563.1       --------------------------------------------------
123 YP_443922.1       TFLGEVQDLQTELSQPGPRRSQRLIEQRNRTHLQAPLRLCATGRGEQYSP
124 NP_057933.2       ALIESVLITHQPTWDDCQQLLGTLLTGEEKQRVLLEARKAVRGDDGRPTQ
125 YP_009116631.1    --------------------------------------------------
126 YP_009513249.1    --------------------------------------------------
127 YP_223871.1       --------------------------------------------------
128 NP_127504.1       --------------------------------------------------
129 NP_044929.1       --------------------------------------------------
130 NP_057860.1       -----------MGQIFSRSASPIPRPPRGLAAHHWLNFLQAAYRLEPGPS
131 NP_040973.1       --------------------------------------------------
132 NP_777317.1       --------------------------------------------------
133 NP_054716.1       --------------------------------------------------
134 YP_001856242.1    --------------------------------------------------
135 NP_954565.2       TETVDGQGQAWRHHNGFDFAVIKELKTAASQYGATAPYTLAIVESVADNW
136 NP_569150.1       --------------------------------------------------
137 NP_659397.1       --------------------------------------------------
138 YP_009513211.1    --------------------------------------------------
139 YP_233107.1       --------------------------------------------------
140 YP_009109692.1    --------------------------------------------------
141 NP_057849.4       ELERFAVNPGLLETSEGCRQILGQLQPSLQTGSEELRSLYNTVATLYCVH
142 NP_049560.1       --------------------------------------------------
143 YP_009506267.1    --------------------------------------------------
144 NP_039820.1       --------------------------------------------------
145 NP_056790.1       --------------------------------------------------
146 YP_002321513.1    --------------------------------------------------
147 NP_955591.1       --------------------------------------------------
148 YP_001956722.2    --------------------------------------------------
149 NP_056902.1       --------------------------------------------------
150 NP_056886.1       IEKPATERRIDKGEEVGETTVQRDAKMAPEETATPKTVGTSCYHCGTAIG
151 NP_041261.1       --------------------------------------------------
```

```
001 YP_004442836.1    --------------------------------------------------
002 NP_056728.1       --------------------------------------------------
003 YP_009508537.1    --------------------------------------------------
004 YP_004732983.2    --------------------------------------------------
005 YP_009508407.1    --------------------------------------------------
```

```
006 NP_040939.1        --------------------------------------------------
007 YP_003864102.1     --------------------------------------------------
008 YP_009002585.1     --------------------------------------------------
009 YP_003284237.1     --------------------------------------------------
010 YP_009268869.1     --------------------------------------------------
011 YP_009506251.1     --------------------------------------------------
012 NP_955611.1        --------------------------------------------------
013 YP_008567619.1     --------------------------------------------------
014 NP_149413.1        --------------------------------------------------
015 YP_004442824.1     --------------------------------------------------
016 YP_001931967.1     --------------------------------------------------
017 YP_002455786.1     --------------------------------------------------
018 YP_009109694.1     --------------------------------------------------
019 NP_789739.1        --------------------------------------------------
020 NP_955619.1        --------------------------------------------------
021 YP_224289.1        --------------------------------------------------
022 YP_009182100.1     --------------------------------------------------
023 NP_705927.1        --------------------------------------------------
024 YP_009508577.1     --------------------------------------------------
025 YP_004442830.1     --------------------------------------------------
026 NP_758887.1        --------------------------------------------------
027 YP_009508406.1     --------------------------------------------------
028 YP_610965.1        --------------------------------------------------
029 YP_001931961.1     --------------------------------------------------
030 YP_241114.1        --------------------------------------------------
031 YP_006732334.1     --------------------------------------------------
032 YP_009041481.1     --------------------------------------------------
033 YP_001036293.1     --------------------------------------------------
034 YP_605811.1        --------------------------------------------------
035 NP_955577.1        --------------------------------------------------
036 YP_003208050.1     --------------------------------------------------
037 NP_789740.1        --------------------------------------------------
038 YP_004442839.1     --------------------------------------------------
039 YP_009507791.1     --------------------------------------------------
040 YP_004581513.1     --------------------------------------------------
041 NP_043933.1        --------------------------------------------------
042 YP_009507248.1     --------------------------------------------------
043 YP_002117531.1     --------------------------------------------------
044 YP_004347415.1     --------------------------------------------------
045 NP_056891.1        LTPTDWNTLVRAVLSGGDHLLWKSEFFENCRDTAKRNQQAGNGWD-FDML
046 YP_009513242.1     --------------------------------------------------
047 YP_009352866.1     --------------------------------------------------
048 YP_009165750.1     --------------------------------------------------
049 NP_040840.1        --------------------------------------------------
050 YP_004222728.1     --------------------------------------------------
051 YP_009506264.1     --------------------------------------------------
052 YP_567050.1        --------------------------------------------------
053 YP_009508571.1     --------------------------------------------------
054 NP_056803.1        --------------------------------------------------
055 NP_955564.1        --------------------------------------------------
056 YP_009508411.1     --------------------------------------------------
057 YP_009140788.1     --------------------------------------------------
058 YP_009508410.1     --------------------------------------------------
059 NP_040333.1        --------------------------------------------------
060 YP_008992013.1     --------------------------------------------------
061 NP_041734.1        --------------------------------------------------
062 NP_056895.1        PSDFTDLKNYIHWFHKTQKKPWTFTSGGPASCPPGKFG------------
063 YP_009109689.1     LPNEIDEGFPLTRPDWD---------------------------------
064 YP_002519387.1     --------------------------------------------------
065 YP_595725.1        --------------------------------------------------
066 YP_009508551.1     --------------------------------------------------
067 YP_001497148.1     LPNEIDEGFPLTRPDWD---------------------------------
068 NP_041186.1        --------------------------------------------------
069 NP_663784.1        AEEKVKDTEEAKKLAQRHLVAETGTAEKMPNTSRPTAPPSGKRGNYPVQQ
070 YP_009508443.1     --------------------------------------------------
071 YP_003987465.1     --------------------------------------------------
072 NP_861410.1        --------------------------------------------------
073 YP_009345071.1     --------------------------------------------------
074 YP_009508582.1     --------------------------------------------------
075 NP_569141.1        --------------------------------------------------
076 YP_002916057.1     --------------------------------------------------
077 NP_777384.2        PSDFTDLKNYIHWFHKTQKKPWTFTSGGPASCPPGKFG------------
078 YP_233110.1        --------------------------------------------------
```

```
079 NP_542258.1       --------------------------------------------------
080 YP_009130664.1    --------------------------------------------------
081 YP_009508566.1    --------------------------------------------------
082 YP_006607892.1    --------------------------------------------------
083 YP_009506270.1    --------------------------------------------------
084 NP_042513.1       --------------------------------------------------
085 YP_006907834.1    --------------------------------------------------
086 YP_004442827.1    --------------------------------------------------
087 YP_009408594.1    --------------------------------------------------
088 YP_009508561.1    --------------------------------------------------
089 NP_056907.1       DFDFQQLRRFLKLALKTPIWLNPIDYSLLASLIPKGYPGRTIEIINVLIK
090 YP_002308474.1    --------------------------------------------------
091 YP_007761644.1    --------------------------------------------------
092 NP_040550.1       --------------------------------------------------
093 NP_758808.1       --------------------------------------------------
094 YP_009345075.1    --------------------------------------------------
095 NP_068729.1       --------------------------------------------------
096 NP_047255.1       LPNVIDETFPLTRPNWD---------------------------------
097 YP_006273075.1    --------------------------------------------------
098 YP_009508888.1    --------------------------------------------------
099 NP_395469.1       --------------------------------------------------
100 YP_009508546.1    --------------------------------------------------
101 NP_056762.1       --------------------------------------------------
102 YP_009508556.1    --------------------------------------------------
103 NP_569153.1       --------------------------------------------------
104 NP_043924.1       --------------------------------------------------
105 YP_009243641.1    --------------------------------------------------
106 YP_004300274.1    --------------------------------------------------
107 NP_056848.1       --------------------------------------------------
108 YP_006495799.1    --------------------------------------------------
109 YP_009508409.1    --------------------------------------------------
110 NP_687035.1       AEQKVKDTEEAVVTVKQHYHLVDKNEKAAKKKNETTAPPGGESRNYPVVN
111 NP_045937.2       --------------------------------------------------
112 NP_612577.1       --------------------------------------------------
113 NP_619548.1       --------------------------------------------------
114 NP_056880.1       LTPSDWHQTARATLSPGDYVLWRTEYEEKSKEMVQKAAGKRKGKVSLDML
115 YP_009121747.1    --------------------------------------------------
116 YP_009113237.2    --------------------------------------------------
117 YP_009508408.1    --------------------------------------------------
118 NP_955579.1       --------------------------------------------------
119 YP_009229919.1    --------------------------------------------------
120 YP_004442833.1    --------------------------------------------------
121 NP_862833.2       --------------------------------------------------
122 NP_040563.1       --------------------------------------------------
123 YP_443922.1       WKLTDLTSMMEQLPSLHGGASAWLLQLQTLTSGFRLGLGDMRALLARATD
124 NP_057933.2       LPNEVDAAFPLERPDWD---------------------------------
125 YP_009116631.1    --------------------------------------------------
126 YP_009513249.1    --------------------------------------------------
127 YP_223871.1       --------------------------------------------------
128 NP_127504.1       --------------------------------------------------
129 NP_044929.1       --------------------------------------------------
130 NP_057860.1       SYDFHQLKKFLKIALETPVWICPINYSLLASLLPKGYPGRVNEILHILIQ
131 NP_040973.1       --------------------------------------------------
132 NP_777317.1       --------------------------------------------------
133 NP_054716.1       --------------------------------------------------
134 YP_001856242.1    --------------------------------------------------
135 NP_954565.2       LTPTDWNTLVRAVLSGGDHLLWKSEFFENCRDTAKRNQQAGNGWD-FDML
136 NP_569150.1       --------------------------------------------------
137 NP_659397.1       --------------------------------------------------
138 YP_009513211.1    --------------------------------------------------
139 YP_233107.1       --------------------------------------------------
140 YP_009109692.1    --------------------------------------------------
141 NP_057849.4       QRIEIKDTKEALDKIEEEQNKSKKKAQQAAADTGHSNQVSQN--YPIVQN
142 NP_049560.1       --------------------------------------------------
143 YP_009506267.1    --------------------------------------------------
144 NP_039820.1       --------------------------------------------------
145 NP_056790.1       --------------------------------------------------
146 YP_002321513.1    --------------------------------------------------
147 NP_955591.1       --------------------------------------------------
148 YP_001956722.2    --------------------------------------------------
149 NP_056902.1       --------------------------------------------------
150 NP_056886.1       CNCATASAPPPPYVGSGLYPSLAGVGEQQGQGGDTPPGAEQSRAEPGHAG
151 NP_041261.1       --------------------------------------------------
```

```
001 YP_004442836.1   --------------------------------------------------
002 NP_056728.1      --------------------------------------------------
003 YP_009508537.1   --------------------------------------------------
004 YP_004732983.2   --------------------------------------------------
005 YP_009508407.1   --------------------------------------------------
006 NP_040939.1      --------------------------------------------------
007 YP_003864102.1   --------------------------------------------------
008 YP_009002585.1   --------------------------------------------------
009 YP_003284237.1   --------------------------------------------------
010 YP_009268869.1   --------------------------------------------------
011 YP_009506251.1   --------------------------------------------------
012 NP_955611.1      --------------------------------------------------
013 YP_008567619.1   --------------------------------------------------
014 NP_149413.1      --------------------------------------------------
015 YP_004442824.1   --------------------------------------------------
016 YP_001931967.1   --------------------------------------------------
017 YP_002455786.1   --------------------------------------------------
018 YP_009109694.1   --------------------------------------------------
019 NP_789739.1      --------------------------------------------------
020 NP_955619.1      --------------------------------------------------
021 YP_224289.1      --------------------------------------------------
022 YP_009182100.1   --------------------------------------------------
023 NP_705927.1      --------------------------------------------------
024 YP_009508577.1   --------------------------------------------------
025 YP_004442830.1   --------------------------------------------------
026 NP_758887.1      --------------------------------------------------
027 YP_009508406.1   --------------------------------------------------
028 YP_610965.1      --------------------------------------------------
029 YP_001931961.1   --------------------------------------------------
030 YP_241114.1      --------------------------------------------------
031 YP_006732334.1   --------------------------------------------------
032 YP_009041481.1   --------------------------------------------------
033 YP_001036293.1   --------------------------------------------------
034 YP_605811.1      --------------------------------------------------
035 NP_955577.1      --------------------------------------------------
036 YP_003208050.1   --------------------------------------------------
037 NP_789740.1      --------------------------------------------------
038 YP_004442839.1   --------------------------------------------------
039 YP_009507791.1   --------------------------------------------------
040 YP_004581513.1   --------------------------------------------------
041 NP_043933.1      --------------------------------------------------
042 YP_009507248.1   --------------------------------------------------
043 YP_002117531.1   --------------------------------------------------
044 YP_004347415.1   --------------------------------------------------
045 NP_056891.1      TGSGNYSSTDAQMQYDPGLFAQIQAAATKAWRKLPVKGDPGASLTGVKQG
046 YP_009513242.1   --------------------------------------------------
047 YP_009352866.1   --------------------------------------------------
048 YP_009165750.1   --------------------------------------------------
049 NP_040840.1      --------------------------------------------------
050 YP_004222728.1   --------------------------------------------------
051 YP_009506264.1   --------------------------------------------------
052 YP_567050.1      --------------------------------------------------
053 YP_009508571.1   --------------------------------------------------
054 NP_056803.1      --------------------------------------------------
055 NP_955564.1      --------------------------------------------------
056 YP_009508411.1   --------------------------------------------------
057 YP_009140788.1   --------------------------------------------------
058 YP_009508410.1   --------------------------------------------------
059 NP_040333.1      --------------------------------------------------
060 YP_008992013.1   --------------------------------------------------
061 NP_041734.1      --------------------------------------------------
062 NP_056895.1      ---------RVPLVLATLNEVLSNDEGAPGASAPEEQPPPYDPPAILPII
063 YP_009109689.1   ------------YETAPGRESLRIYRQALLAGLKGAGKRPTNLAKVRTIT
064 YP_002519387.1   --------------------------------------------------
065 YP_595725.1      --------------------------------------------------
066 YP_009508551.1   --------------------------------------------------
067 YP_001497148.1   ------------YETAPGRESLRIYRQALLAGLKGAGKRPTNLAKVRTII
068 NP_041186.1      --------------------------------------------------
069 NP_663784.1      AGGNYVHVPLSPRTLNAWVKLVEEKKFGAEVVPGFQALSEGCTPYDINQM
070 YP_009508443.1   --------------------------------------------------
```

```
071 YP_003987465.1    --------------------------------------------------
072 NP_861410.1       --------------------------------------------------
073 YP_009345071.1    --------------------------------------------------
074 YP_009508582.1    --------------------------------------------------
075 NP_569141.1       --------------------------------------------------
076 YP_002916057.1    --------------------------------------------------
077 NP_777384.2       ---------RVPLVLATLNEVLSNDEGAPGASAPEEQPPPYDPPAILPII
078 YP_233110.1       --------------------------------------------------
079 NP_542258.1       --------------------------------------------------
080 YP_009130664.1    --------------------------------------------------
081 YP_009508566.1    --------------------------------------------------
082 YP_006607892.1    --------------------------------------------------
083 YP_009506270.1    --------------------------------------------------
084 NP_042513.1       --------------------------------------------------
085 YP_006907834.1    --------------------------------------------------
086 YP_004442827.1    --------------------------------------------------
087 YP_009408594.1    --------------------------------------------------
088 YP_009508561.1    --------------------------------------------------
089 NP_056907.1       NQTSPTPPPAPSLPEPANPPPLQQPSAPPEPHTPPPYIKPPATHCLPILH
090 YP_002308474.1    --------------------------------------------------
091 YP_007761644.1    --------------------------------------------------
092 NP_040550.1       --------------------------------------------------
093 NP_758808.1       --------------------------------------------------
094 YP_009345075.1    --------------------------------------------------
095 NP_068729.1       --------------------------------------------------
096 NP_047255.1       ------------FATPAGREHLRLYRQLLLAGLRGAARRPTNLAQVKQVV
097 YP_006273075.1    --------------------------------------------------
098 YP_009508888.1    --------------------------------------------------
099 NP_395469.1       --------------------------------------------------
100 YP_009508546.1    --------------------------------------------------
101 NP_056762.1       --------------------------------------------------
102 YP_009508556.1    --------------------------------------------------
103 NP_569153.1       --------------------------------------------------
104 NP_043924.1       -----MASNKWFVYSDEPTKVILKRDKSKEKDETKKKKIKTEQNSDAAYE
105 YP_009243641.1    --------------------------------------------------
106 YP_004300274.1    --------------------------------------------------
107 NP_056848.1       --------------------------------------------------
108 YP_006495799.1    --------------------------------------------------
109 YP_009508409.1    --------------------------------------------------
110 NP_687035.1       QNNAWVHQPLSPRTLNAWVKCVEEKRWGAEVVPMFQALSEGCLSYDVNQM
111 NP_045937.2       --------------------------------------------------
112 NP_612577.1       --------------------------------------------------
113 NP_619548.1       --------------------------------------------------
114 NP_056880.1       LGTGQFLSPSSQIKLSKDVLKDVTTNAVLAWRAIPPPGVKKTVLAGLKQG
115 YP_009121747.1    --------------------------------------------------
116 YP_009113237.2    --------------------------------------------------
117 YP_009508408.1    --------------------------------------------------
118 NP_955579.1       --------------------------------------------------
119 YP_009229919.1    --------------------------------------------------
120 YP_004442833.1    --------------------------------------------------
121 NP_862833.2       --------------------------------------------------
122 NP_040563.1       --------------------------------------------------
123 YP_443922.1       HVTMNVIMKTAGLDHLPGPTPPEVYTGPLWAELRRNYPTERDYAALSSFL
124 NP_057933.2       ------------YTTQAGRNHLVHYRQLLLAGLQNAGRSPTNLAKVKGIT
125 YP_009116631.1    --------------------------------------------------
126 YP_009513249.1    --------------------------------------------------
127 YP_223871.1       --------------------------------------------------
128 NP_127504.1       --------------------------------------------------
129 NP_044929.1       --------------------------------------------------
130 NP_057860.1       -----TQAQIPSRPAPPPPSSSTHDPPDSDPQIPPPYVEPTAPQVLPVMH
131 NP_040973.1       --------------------------------------------------
132 NP_777317.1       --------------------------------------------------
133 NP_054716.1       --------------------------------------------------
134 YP_001856242.1    --------------------------------------------------
135 NP_954565.2       TGSGNYSSTDAQMQYDPGLFAQIQAAATKAWRKLPVKGDPGASLTGVKQG
136 NP_569150.1       --------------------------------------------------
137 NP_659397.1       --------------------------------------------------
138 YP_009513211.1    --------------------------------------------------
139 YP_233107.1       --------------------------------------------------
140 YP_009109692.1    --------------------------------------------------
141 NP_057849.4       IQGQMVHQAISPRTLNAWVKVVEEKAFSPEVIPMFSALSEGATPQDLNTM
142 NP_049560.1       --------------------------------------------------
143 YP_009506267.1    --------------------------------------------------
```

```
144 NP_039820.1       --------------------------------------------------
145 NP_056790.1       --------------------------------------------------
146 YP_002321513.1    --------------------------------------------------
147 NP_955591.1       --------------------------------------------------
148 YP_001956722.2    --------------------------------------------------
149 NP_056902.1       --------------------------------------------------
150 NP_056886.1       QAPGPALTDWARVREELASTGPPVVAMPVVIKTEGPAWTPLEPKLITRLA
151 NP_041261.1       --------------------------------------------------


001 YP_004442836.1    --------------------------------------------------
002 NP_056728.1       --------------------------------------------------
003 YP_009508537.1    --------------------------------------------------
004 YP_004732983.2    --------------------------------------------------
005 YP_009508407.1    --------------------------------------------------
006 NP_040939.1       --------------------------------------------------
007 YP_003864102.1    --------------------------------------------------
008 YP_009002585.1    --------------------------------------------------
009 YP_003284237.1    --------------------------------------------------
010 YP_009268869.1    --------------------------------------------------
011 YP_009506251.1    --------------------------------------------------
012 NP_955611.1       --------------------------------------------------
013 YP_008567619.1    --------------------------------------------------
014 NP_149413.1       --------------------------------------------------
015 YP_004442824.1    --------------------------------------------------
016 YP_001931967.1    --------------------------------------------------
017 YP_002455786.1    --------------------------------------------------
018 YP_009109694.1    --------------------------------------------------
019 NP_789739.1       --------------------------------------------------
020 NP_955619.1       --------------------------------------------------
021 YP_224289.1       --------------------------------------------------
022 YP_009182100.1    --------------------------------------------------
023 NP_705927.1       --------------------------------------------------
024 YP_009508577.1    --------------------------------------------------
025 YP_004442830.1    --------------------------------------------------
026 NP_758887.1       --------------------------------------------------
027 YP_009508406.1    --------------------------------------------------
028 YP_610965.1       --------------------------------------------------
029 YP_001931961.1    --------------------------------------------------
030 YP_241114.1       --------------------------------------------------
031 YP_006732334.1    --------------------------------------------------
032 YP_009041481.1    --------------------------------------------------
033 YP_001036293.1    --------------------------------------------------
034 YP_605811.1       --------------------------------------------------
035 NP_955577.1       --------------------------------------------------
036 YP_003208050.1    --------------------------------------------MPEAAA
037 NP_789740.1       --------------------------------------------------
038 YP_004442839.1    --------------------------------------------------
039 YP_009507791.1    --------------------------------------------------
040 YP_004581513.1    --------------------------------------------------
041 NP_043933.1       --------------------------------------------------
042 YP_009507248.1    --------------------------------------------------
043 YP_002117531.1    --------------------------------------------------
044 YP_004347415.1    --------------------------------------------------
045 NP_056891.1       PDEPFADFVHRLITTAGRIFGSAEAGVDYVKQLAYENANPACQAAIRPYR
046 YP_009513242.1    --------------------------------------------------
047 YP_009352866.1    --------------------------------------------------
048 YP_009165750.1    --------------------------------------------------
049 NP_040840.1       --------------------------------------------------
050 YP_004222728.1    --------------------------------------------------
051 YP_009506264.1    --------------------------------------------------
052 YP_567050.1       --------------------------------------------------
053 YP_009508571.1    --------------------------------------------------
054 NP_056803.1       --------------------------------------------------
055 NP_955564.1       --------------------------------------------------
056 YP_009508411.1    --------------------------------------------------
057 YP_009140788.1    --------------------------------------------------
058 YP_009508410.1    --------------------------------------------------
059 NP_040333.1       --------------------------------------------------
060 YP_008992013.1    --------------------------------------------------
061 NP_041734.1       --------------------------------------------------
062 NP_056895.1       SEGNRNRHRAWALRELQDIKKEIENKAPGSQVWIQTLRLAILQADPTPAD
```

```
063 YP_009109689.1    QGKDESPAAFMERLLEGFRMYT----------------------------
064 YP_002519387.1    --------------------------------------------------
065 YP_595725.1       --------------------------------------------------
066 YP_009508551.1    --------------------------------------------------
067 YP_001497148.1    QGKEESPAAFMERLLEGFRMYT----------------------------
068 NP_041186.1       --------------------------------------------------
069 NP_663784.1       LNCVGDHQAAMQIIREIINEEAADWDSQHPIPG-PLPAGQLRDPRGSDIA
070 YP_009508443.1    --------------------------------------------------
071 YP_003987465.1    --------------------------------------------------
072 NP_861410.1       --------------------------------------------------
073 YP_009345071.1    --------------------------------------------------
074 YP_009508582.1    --------------------------------------------------
075 NP_569141.1       --------------------------------------------------
076 YP_002916057.1    --------------------------------------------------
077 NP_777384.2       SEGNRNRHRAWALRELQDIKKEIENKAPGSQVWIQTLRLAILQADPTPAD
078 YP_233110.1       --------------------------------------------------
079 NP_542258.1       --------------------------------------------------
080 YP_009130664.1    --------------------------------------------------
081 YP_009508566.1    --------------------------------------------------
082 YP_006607892.1    --------------------------------------------------
083 YP_009506270.1    --------------------------------------------------
084 NP_042513.1       --------------------------------------------------
085 YP_006907834.1    --------------------------------------------------
086 YP_004442827.1    --------------------------------------------------
087 YP_009408594.1    --------------------------------------------------
088 YP_009508561.1    --------------------------------------------------
089 NP_056907.1       PHGAPSAHSPWQMKDLQAIKQEVSTSAPGSPQFMQTVRLAIQQFDPTAKD
090 YP_002308474.1    --------------------------------------------------
091 YP_007761644.1    --------------------------------------------------
092 NP_040550.1       --------------------------------------------------
093 NP_758808.1       --------------------------------------------------
094 YP_009345075.1    --------------------------------------------------
095 NP_068729.1       --------------------------------------------------
096 NP_047255.1       QGKEETPAAFLERLKEAYRMYT----------------------------
097 YP_006273075.1    --------------------------------------------------
098 YP_009508888.1    --------------------------------------------------
099 NP_395469.1       --------------------------------------------------
100 YP_009508546.1    --------------------------------------------------
101 NP_056762.1       --------------------------------------------------
102 YP_009508556.1    --------------------------------------------------
103 NP_569153.1       --------------------------------------------------
104 NP_043924.1       TPGTAPVQKPLLETTPEAELEKVLKGLEEWGYKALEKKRDPELWNPNQEG
105 YP_009243641.1    --------------------------------------------------
106 YP_004300274.1    --------------------------------------------------
107 NP_056848.1       --------------------------------------------------
108 YP_006495799.1    --------------------------------------------------
109 YP_009508409.1    --------------------------------------------------
110 NP_687035.1       LNVIGDHQGALQILKEVINEEAAEWDRTHRPPAGPLPAGQLRDPTGSDIA
111 NP_045937.2       --------------------------------------------------
112 NP_612577.1       --------------------------------------------------
113 NP_619548.1       --------------------------------------------------
114 NP_056880.1       NEESYETFISRLEEAVYRMMPRGEGSDILIKQLAWENANSLCQDLIRPIR
115 YP_009121747.1    --------------------------------------------------
116 YP_009113237.2    --------------------------------------------------
117 YP_009508408.1    --------------------------------------------------
118 NP_955579.1       --------------------------------------------------
119 YP_009229919.1    --------------------------------------------------
120 YP_004442833.1    --------------------------------------------------
121 NP_862833.2       --------------------------------------------------
122 NP_040563.1       --------------------------------------------------
123 YP_443922.1       MNPGEGPAEYLDRAKTTWRTVHGEPHDASETSLNMWKEVVINGTPAPVKA
124 NP_057933.2       QGPNESPSAFLERLKEAYRRYT----------------------------
125 YP_009116631.1    --------------------------------------------------
126 YP_009513249.1    --------------------------------------------------
127 YP_223871.1       --------------------------------------------------
128 NP_127504.1       --------------------------------------------------
129 NP_044929.1       --------------------------------------------------
130 NP_057860.1       PHGAPPNHRPWQMKDLQAIKQEVSQAAPGSPQFMQTIRLAVQQFDPTAKD
131 NP_040973.1       --------------------------------------------------
132 NP_777317.1       --------------------------------------------------
133 NP_054716.1       --------------------------------------------------
134 YP_001856242.1    --------------------------------------------------
135 NP_954565.2       PDEPFADFVHRLITTAGRIFGSAEAGVDYVKQLAYENANPACQAAIRPYR
```

```
136 NP_569150.1       --------------------------------------------------
137 NP_659397.1       --------------------------------------------------
138 YP_009513211.1    --------------------------------------------------
139 YP_233107.1       --------------------------------------------------
140 YP_009109692.1    --------------------------------------------------
141 NP_057849.4       LNTVGGHQAAMQMLKETINEEAAEWDRVHPVHAGPIAPGQMREPRGSDIA
142 NP_049560.1       --------------------------------------------------
143 YP_009506267.1    --------------------------------------------------
144 NP_039820.1       --------------------------------------------------
145 NP_056790.1       --------------------------------------------------
146 YP_002321513.1    --------------------------------------------------
147 NP_955591.1       --------------------------------------------------
148 YP_001956722.2    --------------------------------------------------
149 NP_056902.1       --------------------------------------------------
150 NP_056886.1       DTVRTKGLRSPITMAEVEALMSSPLLPHDVTNLMRVILGPAPYALWMDAW
151 NP_041261.1       --------------------------------------------------
```

```
001 YP_004442836.1    --------------------------------------------------
002 NP_056728.1       --------------------------------------------------
003 YP_009508537.1    --------------------------------------------------
004 YP_004732983.2    --------------------------------------------------
005 YP_009508407.1    --------------------------------------------------
006 NP_040939.1       --------------------------------------------------
007 YP_003864102.1    --------------------------------------------------
008 YP_009002585.1    --------------------------------------------------
009 YP_003284237.1    --------------------------------------------------
010 YP_009268869.1    --------------------------------------------------
011 YP_009506251.1    --------------------------------------------------
012 NP_955611.1       --------------------------------------------------
013 YP_008567619.1    --------------------------------------------------
014 NP_149413.1       --------------------------------------------------
015 YP_004442824.1    --------------------------------------------------
016 YP_001931967.1    --------------------------------------------------
017 YP_002455786.1    --------------------------------------------------
018 YP_009109694.1    --------------------------------------------------
019 NP_789739.1       --------------------------------------------------
020 NP_955619.1       --------------------------------------------------
021 YP_224289.1       --------------------------------------------------
022 YP_009182100.1    --------------------------------------------------
023 NP_705927.1       --------------------------------------------------
024 YP_009508577.1    --------------------------------------------------
025 YP_004442830.1    --------------------------------------------------
026 NP_758887.1       --------------------------------------------------
027 YP_009508406.1    --------------------------------------------------
028 YP_610965.1       --------------------------------------------------
029 YP_001931961.1    --------------------------------------------------
030 YP_241114.1       --------------------------------------------------
031 YP_006732334.1    --------------------------------------------------
032 YP_009041481.1    --------------------------------------------------
033 YP_001036293.1    --------------------------------------------------
034 YP_605811.1       --------------------------------------------------
035 NP_955577.1       --------------------------------------------------
036 YP_003208050.1    PAAYAANWPKGAVLYNGVNFLAADGKTVILSDAAKRIKAGVAYLRRKQRR
037 NP_789740.1       --------------------------------------------------
038 YP_004442839.1    --------------------------------------------------
039 YP_009507791.1    --------------------------------------------------
040 YP_004581513.1    --------------------------------------------------
041 NP_043933.1       --------------------------------------------------
042 YP_009507248.1    --------------------------------------------------
043 YP_002117531.1    --------------------------------------------------
044 YP_004347415.1    --------------------------------------------------
045 NP_056891.1       KKTDLTGYIRLCSDIGPSYQQGLAMAAAFSGQTVKDFL-------NNKNK
046 YP_009513242.1    --------------------------------------------------
047 YP_009352866.1    --------------------------------------------------
048 YP_009165750.1    --------------------------------------------------
049 NP_040840.1       --------------------------------------------------
050 YP_004222728.1    --------------------------------------------------
051 YP_009506264.1    --------------------------------------------------
052 YP_567050.1       --------------------------------------------------
053 YP_009508571.1    --------------------------------------------------
054 NP_056803.1       --------------------------------------------------
```

```
055 NP_955564.1        --------------------------------------------------
056 YP_009508411.1     --------------------------------------------------
057 YP_009140788.1     --------------------------------------------------
058 YP_009508410.1     --------------------------------------------------
059 NP_040333.1        --------------------------------------------------
060 YP_008992013.1     --------------------------------------------------
061 NP_041734.1        --------------------------------------------------
062 NP_056895.1        LEQLCQYIASPVDQTAHMTSLTAAIAAAEAANTLQGFNPQNGTLTQQSAQ
063 YP_009109689.1     ------PFDPEAPEHKATVAMSFIDQAALDIKGKLQRLDGIQTHGLQELV
064 YP_002519387.1     --------------------------------------------------
065 YP_595725.1        --------------------------------------------------
066 YP_009508551.1     --------------------------------------------------
067 YP_001497148.1     ------PFTPEAPEHKATVAMSFIDQAASDIKGKLQRLDGIQTYGLQELV
068 NP_041186.1        --------------------------------------------------
069 NP_663784.1        GTTSTVDEQIQWMYRPQNPVPVGNIYRRWIQIGLQKCVRKYNPTNILDIK
070 YP_009508443.1     --------------------------------------------------
071 YP_003987465.1     --------------------------------------------------
072 NP_861410.1        --------------------------------------------------
073 YP_009345071.1     --------------------------------------------------
074 YP_009508582.1     --------------------------------------------------
075 NP_569141.1        --------------------------------------------------
076 YP_002916057.1     --------------------------------------------------
077 NP_777384.2        LEQLCQYIASPVDQTAHMTSLTAAIAAAEAANTLQGFNPQNGTLTQQSAQ
078 YP_233110.1        --------------------------------------------------
079 NP_542258.1        --------------------------------------------------
080 YP_009130664.1     --------------------------------------------------
081 YP_009508566.1     --------------------------------------------------
082 YP_006607892.1     --------------------------------------------------
083 YP_009506270.1     --------------------------------------------------
084 NP_042513.1        --------------------------------------------------
085 YP_006907834.1     --------------------------------------------------
086 YP_004442827.1     --------------------------------------------------
087 YP_009408594.1     --------------------------------------------------
088 YP_009508561.1     --------------------------------------------------
089 NP_056907.1        LQDLLQYLCSSLVVSLHHQQFHTLITEAETR-GMTGYNPMAGPLRMQANN
090 YP_002308474.1     --------------------------------------------------
091 YP_007761644.1     --------------------------------------------------
092 NP_040550.1        --------------------------------------------------
093 NP_758808.1        --------------------------------------------------
094 YP_009345075.1     --------------------------------------------------
095 NP_068729.1        --------------------------------------------------
096 NP_047255.1        ------PYDPEDPGQAASVILSFIYQSSPDIRNKLQRLEGLQGFTLSDLL
097 YP_006273075.1     --------------------------------------------------
098 YP_009508888.1     --------------------------------------------------
099 NP_395469.1        --------------------------------------------------
100 YP_009508546.1     --------------------------------------------------
101 NP_056762.1        --------------------------------------------------
102 YP_009508556.1     --------------------------------------------------
103 NP_569153.1        --------------------------------------------------
104 NP_043924.1        LDEYLFRGWVQGGLADSKKALEKNMEKFVPLFVVTMSQAVPYWRQTMQAR
105 YP_009243641.1     --------------------------------------------------
106 YP_004300274.1     --------------------------------------------------
107 NP_056848.1        --------------------------------------------------
108 YP_006495799.1     --------------------------------------------------
109 YP_009508409.1     --------------------------------------------------
110 NP_687035.1        GTTSSIQEQIEWTFNANPRIDVGAQYRKWVILGLQKVVQMYNPQKVLDIR
111 NP_045937.2        ------------MGNSSSTPPPSALKNSDLFKTMLRTQYSGSVKTRRINQ
112 NP_612577.1        --------------------------------------------------
113 NP_619548.1        --------------------------------------------------
114 NP_056880.1        KTGTIQDYIRACLDASPAVVQGMAYAAAMRGQKYSTFVKQTYGGGKGGQG
115 YP_009121747.1     --------------------------------------------------
116 YP_009113237.2     ----------------------MSLRFEESIQSLYNTERLGLIQLLDLSQ
117 YP_009508408.1     --------------------------------------------------
118 NP_955579.1        --------------------------------------------------
119 YP_009229919.1     --------------------------------------------------
120 YP_004442833.1     --------------------------------------------------
121 NP_862833.2        --------------------------------------------------
122 NP_040563.1        --------------------------------------------------
123 YP_443922.1        KLRQTVGLCSKSKAEFCSHVHHHISQHHKDAGGAESEIKALQVQLLKLQL
124 NP_057933.2        ------PYDPEDPGQETNVSMSFIWQSAPDIGRKLERLEDLKNKTLGDLV
125 YP_009116631.1     --------------------------------------------------
126 YP_009513249.1     --------------------------------------------------
127 YP_223871.1        --------------------------------------------------
```

```
128 NP_127504.1          --------------------------------------------------
129 NP_044929.1          --------------------------------------------------
130 NP_057860.1          LQDLLQYLCSSLVASLHHQQLDSLISEAETR-GITGYNPLAGPLRVQANN
131 NP_040973.1          --------------------------------------------------
132 NP_777317.1          --------------------------------------------------
133 NP_054716.1          --------------------------------------------------
134 YP_001856242.1       --------------------------------------------------
135 NP_954565.2          KKTDLTGYIRLCSDIGPSYQQGLAMAAAFSGQTVKDFL-------NNKNK
136 NP_569150.1          --------------------------------------------------
137 NP_659397.1          --------------------------------------------------
138 YP_009513211.1       --------------------------------------------------
139 YP_233107.1          --------------------------------------------------
140 YP_009109692.1       --------------------------------------------------
141 NP_057849.4          GTTSTLQEQIGWMT-NNPPIPVGEIYKRWIILGLNKIVRMYSPTSILDIR
142 NP_049560.1          --------------------------------------------------
143 YP_009506267.1       --------------------------------------------------
144 NP_039820.1          --------------------------------------------------
145 NP_056790.1          --------------------------------------------------
146 YP_002321513.1       --------------------------------------------------
147 NP_955591.1          --------------------------------------------------
148 YP_001956722.2       --------------------------------------------------
149 NP_056902.1          --------------------------------------------------
150 NP_056886.1          GVQLQTVIAAATRDPRHPANGQGRGERTNLNRLKGLADGMVGNPQGQAAL
151 NP_041261.1          --------------------------------------------------
```

```
001 YP_004442836.1       ---------------------------------------------MASRP
002 NP_056728.1          --------------------------------------------------
003 YP_009508537.1       --------------------------------------------------
004 YP_004732983.2       ---------------------------------------------MSRSR
005 YP_009508407.1       --------------------------------------------------
006 NP_040939.1          --------------------------------------------------
007 YP_003864102.1       --------------------------------------------------
008 YP_009002585.1       -------------------------------------------MSTTTTQ
009 YP_003284237.1       ---------------------------------------------MSTRG
010 YP_009268869.1       --------------------------------------------------
011 YP_009506251.1       -------------------------------------------MSQTR--
012 NP_955611.1          --------------------------------------------------
013 YP_008567619.1       --------------------------------------------------
014 NP_149413.1          -------------------------------------------------M
015 YP_004442824.1       ---------------------------------------------MASRP
016 YP_001931967.1       --------------------------------------------------
017 YP_002455786.1       --------------------------------------------------
018 YP_009109694.1       --------------------------------------------------
019 NP_789739.1          --------------------------------------------------
020 NP_955619.1          --------------------------------------------------
021 YP_224289.1          -----------------------------------------MTTRRSSLP
022 YP_009182100.1       --------------------------------------------------
023 NP_705927.1          --------------------------------------------------
024 YP_009508577.1       --------------------------------------------------
025 YP_004442830.1       ---------------------------------------------MAFTG
026 NP_758887.1          --------------------------------------------------
027 YP_009508406.1       --------------------------------------------------
028 YP_610965.1          -------------------------------------------MASYRRP
029 YP_001931961.1       --------------------------------------------------
030 YP_241114.1          --------------------------------------------------
031 YP_006732334.1       --------------------------------------------------
032 YP_009041481.1       ---------------------------------------------MSQT-
033 YP_001036293.1       -----------------------------------------------MSR
034 YP_605811.1          ---------------------------------------------MASRP
035 NP_955577.1          --------------------------------------------------
036 YP_003208050.1       WAKAQMRRAVCAANKARKAYYRLTASTPIIQVGDYGFAEPISKGFRYKGP
037 NP_789740.1          --------------------------------------------------
038 YP_004442839.1       ---------------------------------------------MASRP
039 YP_009507791.1       --------------------------------------------------
040 YP_004581513.1       --------------------------------------------------
041 NP_043933.1          --------------------------------------------------
042 YP_009507248.1       --------------------------------------------------
043 YP_002117531.1       ---------------------------------------------MSR--
044 YP_004347415.1       --------------------------------------------------
045 NP_056891.1          EKGGCCFKCGKKGHFAKNCHEHAHNNAEPKVPGLCPRCKRGKHWANECKS
046 YP_009513242.1       --------------------------------------------------
```

```
047 YP_009352866.1   ---------------------------------------------MSRT-
048 YP_009165750.1   --------------------------------------------------
049 NP_040840.1      --------------------------------------------------
050 YP_004222728.1   --------------------------------------------------
051 YP_009506264.1   ---------------------------------------------MANRG
052 YP_567050.1      --------------------------------------------------
053 YP_009508571.1   --------------------------------------------------
054 NP_056803.1      --------------------------------------------------
055 NP_955564.1      --------------------------------------------------
056 YP_009508411.1   -----------------------------------------------MSL
057 YP_009140788.1   -------------------------------------------MSSSRSR
058 YP_009508410.1   --------------------------------------------------
059 NP_040333.1      --------------------------------------------------
060 YP_008992013.1   --------------------------------------------------
061 NP_041734.1      -------------------------------------------MSRARP-
062 NP_056895.1      PNAGDLRSQYQNLWLQAWKNLPTRPSVQPWSTIVQGPAESYVEFVNRLQI
063 YP_009109689.1   REAEKVYNKRETPEEREARLIKEQEERE-----------DRRDRKRDKHL
064 YP_002519387.1   --------------------------------------------------
065 YP_595725.1      -------------------------------------------------M
066 YP_009508551.1   --------------------------------------------------
067 YP_001497148.1   REAEKVYNKRETPEEKEARLAKEQEARE-----------ERRDRKRDKHL
068 NP_041186.1      --------------------------------------------------
069 NP_663784.1      QGPKEPFQSYVDRFYKSLRAEQTDPAVKNWMTQTLLIQNANPDCKLVLKG
070 YP_009508443.1   -----------------------------------------------MSR
071 YP_003987465.1   ---------------------------------------------MMN--
072 NP_861410.1      --------------------------------------------------
073 YP_009345071.1   ----------------------------------------------MTSR
074 YP_009508582.1   --------------------------------------------------
075 NP_569141.1      --------------------------------------------------
076 YP_002916057.1   --------------------------------------------------
077 NP_777384.2      PNAGDLRSQYQNLWLQAWKNLPTRPSVQPWSTIVQGPAESYVEFVNRLQI
078 YP_233110.1      ---------------------------------------------MASRP
079 NP_542258.1      --------------------------------------------------
080 YP_009130664.1   -----------------------------------------MEMTTSTTA
081 YP_009508566.1   --------------------------------------------------
082 YP_006607892.1   --------------------------------------------------
083 YP_009506270.1   ---------------------------------------------MAQRP
084 NP_042513.1      --------------------------------------------------
085 YP_006907834.1   --------------------------------------------------
086 YP_004442827.1   ---------------------------------------------MAFQG
087 YP_009408594.1   ---------------------------------------------MVVCH
088 YP_009508561.1   --------------------------------------------------
089 NP_056907.1      PAQQGLRREYQNLWLAAFSALPGNTRDPSWAAILQGLEEPYCAFVERLNV
090 YP_002308474.1   --------------------------------------------------
091 YP_007761644.1   --------------------------------------------------
092 NP_040550.1      --------------------------------------------------
093 NP_758808.1      ----------------------------------------MSL----AVR
094 YP_009345075.1   ---------------------------------------MSSQTRTTTQA
095 NP_068729.1      --------------------------------------------------
096 NP_047255.1      KEAEKIYNKRETPEEREERLWQRQEERDKKRH-----------KEMTKVL
097 YP_006273075.1   ---------------------------------------------MNRSR
098 YP_009508888.1   --------------------------------------------------
099 NP_395469.1      --------------------------------------------------
100 YP_009508546.1   --------------------------------------------------
101 NP_056762.1      --------------------------------------------------
102 YP_009508556.1   --------------------------------------------------
103 NP_569153.1      -------------------------------------------MSTSRAR
104 NP_043924.1      NQNGKKQKNRIAELEKEVADLTSAGRGADQVIAGMDKELKKTAEKYQAKL
105 YP_009243641.1   --------------------------------------------------
106 YP_004300274.1   --------------------------------------------------
107 NP_056848.1      --------------------------------------------------
108 YP_006495799.1   ---------------------------------------------MTTR-
109 YP_009508409.1   --------------------------------------------------
110 NP_687035.1      QGPKEPFQDYVDRFYKALRAEQAPQDVKNWMTQTLLIQNANPDCKLILKG
111 NP_045937.2      DIKKQYPLWPDQGTCATKHWEQAVLIPLDSVSEETAKVLNFLRVKIQARK
112 NP_612577.1      --------------------------------------------------
113 NP_619548.1      --------------------------------------------------
114 NP_056880.1      AEGPVCFSCGKTGHIRKDCKDEKGSKRAP--PGLCPRCKKGYHWKSECKS
115 YP_009121747.1   ----------------------------------------MSFGRMPSVL
116 YP_009113237.2   EPKVLNKDLSNNIDFAIKHIVLQSRVLLKHFHSIATRQEALEKMSRYGSR
117 YP_009508408.1   -----------------------------------------------MSL
118 NP_955579.1      --------------------------------------------------
119 YP_009229919.1   ----------------------------------------MSRTRTETRA
```

120 YP_004442833.1 ---MALRG
121 NP_862833.2 --
122 NP_040563.1 --
123 YP_443922.1 KDAQKTAQPKKQMVEGAVPLPQLVHETVKQMLATPSPNPAPYPQPPYQPP
124 NP_057933.2 REAEKIFNKRETPEEREERIRRETEEKEERRRTEDEQKEKERDRRRHREM
125 YP_009116631.1 ---MSRS-
126 YP_009513249.1 --
127 YP_223871.1 --
128 NP_127504.1 --
129 NP_044929.1 --
130 NP_057860.1 PQQQGLRREYQQLWLAAFAALPGSAKDPSWASILQGLEEPYHAFVERLNI
131 NP_040973.1 --
132 NP_777317.1 --MTRP
133 NP_054716.1 --
134 YP_001856242.1 --
135 NP_954565.2 EKGGCCFKCGKKGHFAKNCHEHAHNNAEPKVPGLCPRCKRGKHWANECKS
136 NP_569150.1 ---MAQRP
137 NP_659397.1 --
138 YP_009513211.1 --
139 YP_233107.1 ---MTTQRAHLP
140 YP_009109692.1 --
141 NP_057849.4 QGPKEPFRDYVDRFYKTLRAEQASQEVKNWMTETLLVQNANPDCKTILKA
142 NP_049560.1 --
143 YP_009506267.1 ---MER
144 NP_039820.1 --MATRRL
145 NP_056790.1 --
146 YP_002321513.1 --MSSSRTQTLE
147 NP_955591.1 --
148 YP_001956722.2 --
149 NP_056902.1 --
150 NP_056886.1 LRPGELVAITASALQAFREVARLAEPAGPWADIMQGPSESFVDFANRLIK
151 NP_041261.1 --

001 YP_004442836.1 RVTGSGSRTQTVAEAGTP-----LIDDQIREY------------------
002 NP_056728.1 --
003 YP_009508537.1 --
004 YP_004732983.2 TQTTELPRATRRSTSP-----VERLDDQIRGY------------------
005 YP_009508407.1 --
006 NP_040939.1 --
007 YP_003864102.1 --
008 YP_009002585.1 TVPPVTRNATRRQQGERE---TPLFEDQIRDY------------------
009 YP_003284237.1 RVTGTS--SQTVTEPGQP-----LVEDQIRDY------------------
010 YP_009268869.1 --
011 YP_009506251.1 --THQMPSVTTTVNEQG---EGPLFEDQVRNY------------------
012 NP_955611.1 --
013 YP_008567619.1 -----MAQAQAK----------TLVEDQITQY------------------
014 NP_149413.1 AQVVRGTGSSTVLEDGA------VLDDQIRDY------------------
015 YP_004442824.1 RVSGTGSRTATIAEPGVP-----LIEDQIREY------------------
016 YP_001931967.1 --
017 YP_002455786.1 --
018 YP_009109694.1 --
019 NP_789739.1 --
020 NP_955619.1 --
021 YP_224289.1 TVTETMG-PSTSGRDGTP-----LIEDQIRDY------------------
022 YP_009182100.1 --
023 NP_705927.1 --
024 YP_009508577.1 --
025 YP_004442830.1 QGRRLGTGSTTVLEDNT------ILSDQIRDY------------------
026 NP_758887.1 --
027 YP_009508406.1 --
028 YP_610965.1 AVHNNEASTSTVP----------NQEDQIRDY------------------
029 YP_001931961.1 --
030 YP_241114.1 --
031 YP_006732334.1 --
032 YP_009041481.1 -QTVTTP----------------VVEDQIRDY------------------
033 YP_001036293.1 TATQ--EVSTSQ----------RQFEDQIRDY------------------
034 YP_605811.1 RVSGSTTRT-MVAEPGVP-----LVDDQIREY------------------
035 NP_955577.1 --
036 YP_003208050.1 GAPKKEPRTTSPAERRRRVGPKAQKEEKAQDWSVLDDGNFLASLWEPEDW
037 NP_789740.1 --
038 YP_004442839.1 RVLGSTTRT-MVAEPGTP-----LVDDQIRDY------------------

039	YP_009507791.1	--
040	YP_004581513.1	--
041	NP_043933.1	--
042	YP_009507248.1	--
043	YP_002117531.1	--SVGGA-STSAQASGEP-----LVEDQVRDY------------------
044	YP_004347415.1	--
045	NP_056891.1	KTDNQGNPIPPHQGNRVEG---------PAPGPETSLWGSQLCSSQQKQP
046	YP_009513242.1	--
047	YP_009352866.1	-ETVTIP----------------VVEDQIRDY------------------
048	YP_009165750.1	--
049	NP_040840.1	--
050	YP_004222728.1	--
051	YP_009506264.1	RVTGRSG-STTTTEPGQP-----LVEDQIRDY------------------
052	YP_567050.1	--
053	YP_009508571.1	--
054	NP_056803.1	--
055	NP_955564.1	--
056	YP_009508411.1	ARTTEGVASTSR----------PTHEDQIRDY------------------
057	YP_009140788.1	TTTQQLPAVSSRRPESIHNRDQPLFEDQVRDY------------------
058	YP_009508410.1	--
059	NP_040333.1	--
060	YP_008992013.1	--
061	NP_041734.1	--QHPVPSVTTTTSEQNR--EGPLYEDQIRDY------------------
062	NP_056895.1	SLADNLPDGVPKEPIIDSLSYANANKECQQILQGRGLVAAPVGQKLQACA
063	YP_009109689.1	TKILAAVVTEKRAGKSGETRRRPKV---------DKDQCAYCKERGHWIK
064	YP_002519387.1	--
065	YP_595725.1	TQRVRGTGSSTITEDGA------LLDHQIRDY------------------
066	YP_009508551.1	--
067	YP_001497148.1	TKILAAVVTENRTGKSGETKRRPKI---------EKDQCAYCKERGHWIK
068	NP_041186.1	--
069	NP_663784.1	LGMNPTLEEMLTACQGVGGPGQKARLMAEALKEAMGPS---PIPFAAAQQ
070	YP_009508443.1	TQLS--APSTSG----------NTMEDQIRDY------------------
071	YP_003987465.1	---------TTPREVEQP-----LVEDQIWGY------------------
072	NP_861410.1	--
073	YP_009345071.1	TETVTQPQTGIN------------FEDQIRGY------------------
074	YP_009508582.1	--
075	NP_569141.1	--
076	YP_002916057.1	--
077	NP_777384.2	SLADNLPDGVPKEPIIDSLSYANANKECQQILQGRGLVAAPVGQKLQACA
078	YP_233110.1	RVSGSTTRT-MIAEPGVP-----LVDDQIREY------------------
079	NP_542258.1	--
080	YP_009130664.1	RTQVQPSTSRTT----------PLFEDQIRNY------------------
081	YP_009508566.1	--
082	YP_006607892.1	--
083	YP_009506270.1	RVTGNGSRTEAIAEAGTP-----LIDDQIREY------------------
084	NP_042513.1	--
085	YP_006907834.1	--
086	YP_004442827.1	TRRRLGTGSTTVLEDNS------VLADQIRDY------------------
087	YP_009408594.1	SHSDGYTSTADRP-------------------------------------
088	YP_009508561.1	--
089	NP_056907.1	ALDNGLPEGTPKEPILRSLAYSNANKDCQKLLQARGHTNSPLGDMLRACQ
090	YP_002308474.1	--
091	YP_007761644.1	--
092	NP_040550.1	--
093	NP_758808.1	DRGSNPSTSSTVP----------SQQDQIRDY------------------
094	YP_009345075.1	PVRDAPATSVANA------------EDQIRDY------------------
095	NP_068729.1	--
096	NP_047255.1	ATVVAQNRDKDREESKLGDQRKIPL---------GKDQCAYCKEKGHWVR
097	YP_006273075.1	TVTQQLPAVSTRRREEIN-RDQPLFEDQIRDY------------------
098	YP_009508888.1	--
099	NP_395469.1	--
100	YP_009508546.1	--
101	NP_056762.1	--
102	YP_009508556.1	--
103	NP_569153.1	TVIEQLPPATTARVEERD--NTPLYDDQIRDY------------------
104	NP_043924.1	EELEEQLAAMTVEKEELESQVEGLKESLVEAETKKVSLMEVLTMPTRSKG
105	YP_009243641.1	--
106	YP_004300274.1	--
107	NP_056848.1	--
108	YP_006495799.1	TRTEQVPRATSTRPSSRG-EASSTLDDQIRGY------------------
109	YP_009508409.1	--
110	NP_687035.1	LGMNPTLEEMLIACQGVGGPQHKAKLMVEMMSNGQNMVQVGPQKKG---P
111	NP_045937.2	GETARQMTAHTIKKLIVGTIDKNKQQTEILQKTDESDEEMDTTNTMLFIA

```
112 NP_612577.1       --------------------------------------------------
113 NP_619548.1       --------------------------------------------------
114 NP_056880.1       KFDKDGNPLPPLETNAENSKNLVKGQSPSPAQKGDGVKGSGLNPEAPPFT
115 YP_009121747.1    GRSATTQTQTDLP----------TQEDQIRGY------------------
116 YP_009113237.2    SRTMVQQPAVTERVTEGSDGRIPLAEDQIRSY------------------
117 YP_009508408.1    VTRTREVGSTSA----------PTHEDQIRDY------------------
118 NP_955579.1       --------------------------------------------------
119 YP_009229919.1    PIRDGEGTSGNTP----------TFEDQIRSY------------------
120 YP_004442833.1    --RRLGTGSTTVLEDGT------VLSDQIRNY------------------
121 NP_862833.2       --------------------------------------------------
122 NP_040563.1       --------------------------------------------------
123 YP_443922.1       PPPLSPQYPQAYPGYGYPPRGPPQAGGHWGSRYMNNGACYNCGQPGHQAR
124 NP_057933.2       SKLLATVVSGQKQDRQGGERRRSQL---------DRDQCAYCKEKGHWAK
125 YP_009116631.1    HTRLQAPPATERATSSSD-SGTPTLEDQIRGY------------------
126 YP_009513249.1    --------------------------------------------------
127 YP_223871.1       --------------------------------------------------
128 NP_127504.1       --------------------------------------------------
129 NP_044929.1       --------------------------------------------------
130 NP_057860.1       ALDNGLPEGTPKDPILRSLAYSNANKECQKLLQARGHTNSPLGDMLRACQ
131 NP_040973.1       --------------------------------------------------
132 NP_777317.1       RIEGPVSRT-ETAEAGAP-----LVDDQIREY------------------
133 NP_054716.1       --------------------------------------------------
134 YP_001856242.1    --------------------------------------------------
135 NP_954565.2       KTDNQGNPIPPHQGNRVEG---------PAPGPETSLWGSQLCSSQQKQP
136 NP_569150.1       RITGSRT---TTAEEGTP-----LIDDQIREY------------------
137 NP_659397.1       --------------------------------------------------
138 YP_009513211.1    --------------------------------------------------
139 YP_233107.1       SINGTEVAS-TSRQPGTP-----LVEDQIRDY------------------
140 YP_009109692.1    --------------------------------------------------
141 NP_057849.4       LGPAATLEEMMTACQGVGGPGHKARVLAEAMSQVTNSATIMMQRGNFRNQ
142 NP_049560.1       --------------------------------------------------
143 YP_009506267.1    PQTS-TTLATTP----------SVFEDQVRDY------------------
144 NP_039820.1       PAVTQTDGSRTATESGVP-----EYEDQIRSY------------------
145 NP_056790.1       --------------------------------------------------
146 YP_002321513.1    ERTRPSLFYQPSAPPEEGEERPISYNDQIRDY------------------
147 NP_955591.1       --------------------------------------------------
148 YP_001956722.2    --------------------------------------------------
149 NP_056902.1       --------------------------------------------------
150 NP_056886.1       AVEGSDLPPSARAPVIIDCFRQKSQPDIQQLIRTAPSTLTTPGEIIKYVL
151 NP_041261.1       --------------------------------------------------


001 YP_004442836.1    --------------------------------------------------
002 NP_056728.1       --------------------------------------------------
003 YP_009508537.1    --------------------------------------------------
004 YP_004732983.2    --------------------------------------------------
005 YP_009508407.1    --------------------------------------------------
006 NP_040939.1       --------------------------------------------------
007 YP_003864102.1    --------------------------------------------------
008 YP_009002585.1    --------------------------------------------------
009 YP_003284237.1    --------------------------------------------------
010 YP_009268869.1    --------------------------------------------------
011 YP_009506251.1    --------------------------------------------------
012 NP_955611.1       --------------------------------------------------
013 YP_008567619.1    --------------------------------------------------
014 NP_149413.1       --------------------------------------------------
015 YP_004442824.1    --------------------------------------------------
016 YP_001931967.1    --------------------------------------------------
017 YP_002455786.1    --------------------------------------------------
018 YP_009109694.1    --------------------------------------------------
019 NP_789739.1       --------------------------------------------------
020 NP_955619.1       ------------VQPKKPPPNQPCFRCGKAGHWSRDCTQPRPPPGPCPLC
021 YP_224289.1       --------------------------------------------------
022 YP_009182100.1    --------------------------------------------------
023 NP_705927.1       --------------------------------------------------
024 YP_009508577.1    --------------------------------------------------
025 YP_004442830.1    --------------------------------------------------
026 NP_758887.1       ----------------------------------------------FFRE
027 YP_009508406.1    --------------------------------------------------
028 YP_610965.1       --------------------------------------------------
029 YP_001931961.1    --------------------------------------------------
030 YP_241114.1       --------------------------------------------------
```

031 YP_006732334.1 --
032 YP_009041481.1 --
033 YP_001036293.1 --
034 YP_605811.1 --
035 NP_955577.1 -------------------NLGDQESQGQDPPP-----------------
036 YP_003208050.1 ACPCSPIAESPAQMGPISARTIQGPPKRPTTRESAQEPIEECCFMMRQGS
037 NP_789740.1 --
038 YP_004442839.1 --
039 YP_009507791.1 --
040 YP_004581513.1 --
041 NP_043933.1 --
042 YP_009507248.1 --
043 YP_002117531.1 --
044 YP_004347415.1 --
045 NP_056891.1 ISKLTRATPGSAGLDLCSTSHTVLTPEMGPQALSTGIYGPLPPNTFGLIL
046 YP_009513242.1 --
047 YP_009352866.1 --
048 YP_009165750.1 --
049 NP_040840.1 --
050 YP_004222728.1 --
051 YP_009506264.1 --
052 YP_567050.1 --
053 YP_009508571.1 --
054 NP_056803.1 --
055 NP_955564.1 --
056 YP_009508411.1 --
057 YP_009140788.1 --
058 YP_009508410.1 --
059 NP_040333.1 -------------------TLDDQGGQGQEPPP-----------------
060 YP_008992013.1 ---M
061 NP_041734.1 --
062 NP_056895.1 HWAPKVKQPAILVHTPGPKMPGP-----------RQPAPKRPPPGPCYRC
063 YP_009109689.1 DCPKRPRDQKKPAPVLTLGEDSEQGCQGSGAPP-----------------
064 YP_002519387.1 --
065 YP_595725.1 --
066 YP_009508551.1 --
067 YP_001497148.1 DCPKRPRDSKKSTPVLTLGEESEEGRQGSGAPP-----------------
068 NP_041186.1 --
069 NP_663784.1 RKAIRYWNCGKEGHSARQCRAPRRQGCWKCGKPGHIMANCPERQAGFFRV
070 YP_009508443.1 --
071 YP_003987465.1 --
072 NP_861410.1 --
073 YP_009345071.1 --
074 YP_009508582.1 --
075 NP_569141.1 --
076 YP_002916057.1 --
077 NP_777384.2 HWAPKVKQPAILVHTPGPKMPGP-----------RQPAPKRPPPGPCYRC
078 YP_233110.1 --
079 NP_542258.1 --
080 YP_009130664.1 --
081 YP_009508566.1 --
082 YP_006607892.1 --
083 YP_009506270.1 --
084 NP_042513.1 --
085 YP_006907834.1 --
086 YP_004442827.1 --
087 YP_009408594.1 --
088 YP_009508561.1 --
089 NP_056907.1 AWTPKDKARVLVVQPRKPPPTQPCFRCGKTGHWSRDCTLPRPPPGPCPLC
090 YP_002308474.1 --
091 YP_007761644.1 --
092 NP_040550.1 --
093 NP_758808.1 --
094 YP_009345075.1 --
095 NP_068729.1 --
096 NP_047255.1 DCPKRPRKKPAN---STLLNLGDQESQGQDPPP-----------------
097 YP_006273075.1 --
098 YP_009508888.1 --
099 NP_395469.1 --
100 YP_009508546.1 --
101 NP_056762.1 --
102 YP_009508556.1 --
103 NP_569153.1 --

```
104 NP_043924.1       PKKRGPDLKQIRSLHVMADSLGMDSDGIDWDWLARQAWDYEGDEDPHVKE
105 YP_009243641.1    --------------------------------------------------
106 YP_004300274.1    --------------------------------------------------
107 NP_056848.1       --------------------------------------------------
108 YP_006495799.1    --------------------------------------------------
109 YP_009508409.1    --------------------------------------------------
110 NP_687035.1       RGPLKCFNCGKFGHMQRECKAPRQIKCFKCGKIGHMAKDCKNGQANFFRV
111 NP_045937.2       RNKRERIAQQQQADLAAQQQVLLLQREQQREQREKDIKKRDEKKKKLLPD
112 NP_612577.1       --------------------------------------------------
113 NP_619548.1       --------------------------------------------------
114 NP_056880.1       IHDLPRGTPGSAGLDLSSQKDLILSLEDGVSLVPTLVKGTLPEGTTGLII
115 YP_009121747.1    --------------------------------------------------
116 YP_009113237.2    --------------------------------------------------
117 YP_009508408.1    --------------------------------------------------
118 NP_955579.1       --------------------------------------------------
119 YP_009229919.1    --------------------------------------------------
120 YP_004442833.1    --------------------------------------------------
121 NP_862833.2       --------------------------------------------------
122 NP_040563.1       --------------------------------------------------
123 YP_443922.1       NCPLPQTDSQRRFLLNRSQGQAGRGRGQPRGPLRAPQPIPAPYSPAGPSL
124 NP_057933.2       DCPKKPRGPRGPRPQTSLLTLDDQG-QGQEPPP-----------------
125 YP_009116631.1    --------------------------------------------------
126 YP_009513249.1    --------------------------------------------------
127 YP_223871.1       ------------------------GRQGSSALR-----------------
128 NP_127504.1       --------------------------------------------------
129 NP_044929.1       --------------------------------------------------
130 NP_057860.1       AWTPKDKTKVLVVQPKKPPPNQPCFRCGKAGHWSRDCTQPRPPPGPCPLC
131 NP_040973.1       --------------------------------------------------
132 NP_777317.1       --------------------------------------------------
133 NP_054716.1       --------------------------------------------------
134 YP_001856242.1    --------------------------------------------------
135 NP_954565.2       ISKLTRATPGSAGLDLCSTSHTVLTPEMGPQALSTGIYGPLPPNTFGLIL
136 NP_569150.1       --------------------------------------------------
137 NP_659397.1       --------------------------------------------------
138 YP_009513211.1    --------------------------------------------------
139 YP_233107.1       --------------------------------------------------
140 YP_009109692.1    ----------------TLGEDSEQGCQGSGAPP-----------------
141 NP_057849.4       RKIVKCFNCGKEGHTARNCRAPRKKGCWKCGKEGHQMKDC------TERQ
142 NP_049560.1       --------------------------------------------------
143 YP_009506267.1    --------------------------------------------------
144 NP_039820.1       --------------------------------------------------
145 NP_056790.1       ------------------------GSQGSDPLP-----------------
146 YP_002321513.1    --------------------------------------------------
147 NP_955591.1       --------------------------------------------------
148 YP_001956722.2    --------------------------------------------------
149 NP_056902.1       --------------------------------------------------
150 NP_056886.1       DRQKTAPLTDQGIAAAMSSAIQPLIMAVVNRERDGQTGSGGRARGLCYTC
151 NP_041261.1       --------------------------------------------------


001 YP_004442836.1    ------------RSSRRAAYEAQRVARQAGNIIGRIV--GRQPREHTLSM
002 NP_056728.1       --------------------------------------------------
003 YP_009508537.1    ----------------------------MDLLKPLTVERKGVKIKGYWDS
004 YP_004732983.2    ------------RRMARARYLAEQRIRRSFSRNYR----------ETLER
005 YP_009508407.1    --------------------------------------------------
006 NP_040939.1       ----------------------------------MSQLWKERTYAKRMQR
007 YP_003864102.1    --------------------------------------------------
008 YP_009002585.1    ------------RNSQRRAYTTQRAVRRLRRRITG-----RQPYQETLEQ
009 YP_003284237.1    ------------RRAQRARHEAARVARNIGNIGRTIV--GRQPREHTLAL
010 YP_009268869.1    --------------------------------------------------
011 YP_009506251.1    ------------RRNQRRLFNLRKKARRAQKALTGS----RSRHQETLEQ
012 NP_955611.1       --------------------------------------------------
013 YP_008567619.1    ------------RQNQRRMHNLRQAVRRLTN--SQA----STSGQEIIEQ
014 NP_149413.1       ------------RRSQHLKHELSRKTAKALSWVK-TT--DDNPREQTLEM
015 YP_004442824.1    ------------RNSHRAAYEAQRLTRQAGNIVGRIV--GRQPREHTLSM
016 YP_001931967.1    --------------------------------------------------
017 YP_002455786.1    --------------------------------------------------
018 YP_009109694.1    --------------------------------------------------
019 NP_789739.1       --------------------------------------------------
020 NP_955619.1       QDPTHWKRDCPRLKPTIPEPEPEEDALLLDLPADIPHPKNLHRGGGLTSP
021 YP_224289.1       ------------RASARRRYEAQRVARNIGNVGRRLI--GRQTREDTLAL
022 YP_009182100.1    --------------------------------------------------
```

```
023 NP_705927.1        --------------------------------------------------
024 YP_009508577.1     --------------------------MTTPPLLQLPVEVKKTELNGFWDT
025 YP_004442830.1     ------------NRRQRAKHEMQNIVAKPSAIFKRII--GGSQSEKTLEM
026 NP_758887.1        YPLGQWQTQELPG----DAI-----DPNGPSNARDGRPSRENAVRLHEER
027 YP_009508406.1     --------------------------------------------------
028 YP_610965.1        ------------RANARLRHDAQR---QLQRVRRTLTGRQ--GYRRTLEQ
029 YP_001931961.1     --------------------------------------------------
030 YP_241114.1        --------------------------------------------------
031 YP_006732334.1     --------------------------------------------------
032 YP_009041481.1     ------------RRMQRARHEAQRILPPILGGRRRR----QIQGPRRIEE
033 YP_001036293.1     ------------RLGARRRYNLRQGLNKFKNVLTLG----T-YYAPTLEQ
034 YP_605811.1        ------------RTAARAAYEAQRIARRTGNIIGRIV--GRQPREHTLAM
035 NP_955577.1        -------------------------------EPRITLKIGGQPVTFLVDT
036 YP_003208050.1     TSSTLPPVRGSNTAAAPIQEDQVRDYRRWQRLRWNMRNRWNTTFRRTLES
037 NP_789740.1        ----------LQG----KAR-----EFSSEQTRANSPTRRELQVWGRDNN
038 YP_004442839.1     ------------RNAARAAYEAQRVARQTGNIIGRIV--GRQPREHTLAM
039 YP_009507791.1     --------------------------------------------------
040 YP_004581513.1     --------------------------------------------------
041 NP_043933.1        --------------------------------------------------
042 YP_009507248.1     --------------------------------------------------
043 YP_002117531.1     ------------RRGQRRRYEAQRVARNLQNIGRTIV--GARPREHTLAL
044 YP_004347415.1     --------------------------------------------------
045 NP_056891.1        GRSSITMKGLQVYPGVIDNDYTGEIKIMAKAVNNIVTVSQGNRIAQLILL
046 YP_009513242.1     -------------------------MDPLQLLQPLEAEIKGTKLKAHWDS
047 YP_009352866.1     ------------RRMQRARHEAQRRLPPILGGRRR-----DRQGPQRIEE
048 YP_009165750.1     --------------------------------------------------
049 NP_040840.1        ----------------------------------MPSLWKKRTYAKGLP-
050 YP_004222728.1     --------------------------------------------------
051 YP_009506264.1     ------------RRAQRARYEAQRVARSLGNIGRTIV--GRQPREHTLAL
052 YP_567050.1        --------------------------------------------------
053 YP_009508571.1     -------------------------MNPLQLLQPLQAEVKGNKLIAHWDS
054 NP_056803.1        -------------------------MNPLQLLQPLPAEVKGTKLLAHWDS
055 NP_955564.1        --------------------------------------------------
056 YP_009508411.1     ------------RLGQRRRHNLQQGMKKLSAKLFGG----P-YSR-TLEQ
057 YP_009140788.1     ------------RRNQRRRFIAEQALRRTIGKIRG------RRYNQTLEQ
058 YP_009508410.1     --------------------------------------------------
059 NP_040333.1        -------------------------------EPRITLRVGGQPVTFLVDT
060 YP_008992013.1     ASTSSSNLSTPLTLSSTLSLPKSCTSHTANKIENLVEYSYIPESAQISES
061 NP_041734.1        ------------RRGQRRIFNLRRRARRLRRSMMG------SRYQETLEQ
062 NP_056895.1        LKEGHWARDCPTKTTGPPPGPCPICKRSFPLETRLSNPQIKKLIEGGLSA
063 YP_009109689.1     -------------------------------EPRLTLSVGGHPTTFLVDT
064 YP_002519387.1     --------------------------------------------------
065 YP_595725.1        ------------RRAQHAKHEAQRIAGQALAFLR-VT--SDDPREKTLEM
066 YP_009508551.1     -------------------------MNPLQLLQPLPAEIKGTKLLAHWDS
067 YP_001497148.1     -------------------------------EPRLTLSVGGHPTTFLVDT
068 NP_041186.1        --------------------------------------------------
069 NP_663784.1        GPTGKEASQLPRDPSPSGADTNSTSGRSSSGTVGEIYAAREKAEGAEGET
070 YP_009508443.1     ------------RLGARRRYNMGLRAQKLIKTLSLG----T-FYNPTLEQ
071 YP_003987465.1     ------------RWTQRACYEAQEAGRRLANIGRTIL--GMQPREHTLAM
072 NP_861410.1        --------------------------------------------------
073 YP_009345071.1     ------------RTGQRRRYNWARQARRLRNRIRR-----SPRQPETLET
074 YP_009508582.1     --------------------------MTAPPLLQLPVEVKKTELNGFWDT
075 NP_569141.1        --------------------------------------------------
076 YP_002916057.1     --------------------------------------------------
077 NP_777384.2        LKEGHWARDCPTKTTGPPPGPCPICKRSFPLETRLSNPQIKKLIEGGLSA
078 YP_233110.1        ------------RSAARVAYEAQRIARRTGNILGRIV--GRQPREHTLAM
079 NP_542258.1        --------------------------------------------------
080 YP_009130664.1     ------------RQRERRLHNARQVVGRLHRRITGR----SQLHNQTLEQ
081 YP_009508566.1     --------------------------MYQP-QHQLQVEIHDQKLIGYWDT
082 YP_006607892.1     --------------------------------------------------
083 YP_009506270.1     ------------RNTRRAAYEAQRMARQAGNIVGRIV--GRQPREHTLSM
084 NP_042513.1        --------------------------------------------------
085 YP_006907834.1     --------------------------------------------------
086 YP_004442827.1     ------------SRGQRIKHEMQNIVARPTALFKRIT--GGSASEKTLEM
087 YP_009408594.1     ------------------------------------------------LP
088 YP_009508561.1     --------------------------MAQT---NIPVEIKGTQLNGFWDT
089 NP_056907.1        KDPSHWKRDCPQFKPPPTEEEPLLLDLPSDAIATEEKKL-----------
090 YP_002308474.1     --------------------------------------------------
091 YP_007761644.1     --------------------------------------------------
092 NP_040550.1        --------------------------------------------------
093 NP_758808.1        ------------RNMQRVRHTAERAARRIFPGR----------FNRTLES
094 YP_009345075.1     ------------RRARRTMWNASR---QLTGRRN---------NLRTLEQ
095 NP_068729.1        --------------------------------------------------
```

```
096 NP_047255.1         -------------------------------EPRITLKIGGQPVTFLVDT
097 YP_006273075.1      ------------RRNQRRRFVAEQAVRRTMGRMRG------RRYNQTLEQ
098 YP_009508888.1      -------------------------MSLQHLLQPLEAEVKGTKLKAHWDS
099 NP_395469.1         --------------------------------------------------
100 YP_009508546.1      -------------------------MNPLQLLQPLPAEIKGTKLLAHWDS
101 NP_056762.1         -------------MSLRPFTGTSRTITQDSTSESNIKKGKNSTKRELIEE
102 YP_009508556.1      -------------------------MDPLQLLQPLEAEIKGTKLKAHWDS
103 NP_569153.1         ------------RQWQRRRHNMG----RRWNQLIG-----R-PYNQTLEQ
104 NP_043924.1         AEEEAWRERQTSQPSQPSQLRPFVAAGNGHREDQWRPLTVTELPAAVTAV
105 YP_009243641.1      --------------------------------------------------
106 YP_004300274.1      --------------------------------------------------
107 NP_056848.1         --------------------------------------------------
108 YP_006495799.1      ------------RQTARLRHNVRRRLRSLSPGGFR----------NTLEQ
109 YP_009508409.1      --------------------------------------------------
110 NP_687035.1         WPLGRSETKKFCA----IQRRHSWSGTNSPPNGNSLRSSKEAPPAVCREG
111 NP_045937.2         TTQKVEQTDIGEASSSDASAQKPISTDNNPDLKVDGVLTRSQHTTVPSNI
112 NP_612577.1         --------------------------------------------------
113 NP_619548.1         --------------------------------------------------
114 NP_056880.1         GRSSNYKKGLEVLPGVIDSDFQGEIKVMVKAAKNAVIIHKGERIAQLLLL
115 YP_009121747.1      ------------RTNARRMYNLNQAVRSRLGRR----------YRRNLET
116 YP_009113237.2      ------------RRNARRRYNVQNAARRITARLSN------RPYTRTLEQ
117 YP_009508408.1      ------------RLGARRRYNMGLKAQKLVKTLSRG----K-FYNPTLEQ
118 NP_955579.1         --------------------------------------------------
119 YP_009229919.1      ------------RNIQRVKHQAGR---KLKQLRG--------QHSRSLES
120 YP_004442833.1      ------------RNGQRALYEAQNIVARPNAIFKRIF--GGSETEKTLEM
121 NP_862833.2         --------------------------------------------------
122 NP_040563.1         ------------------------TAKMLPLWQTWPPSKKLQVKKREVLL
123 YP_443922.1         PFQGMYDDNTPWEXRCPPPPEAEVQRYSVYTEPTVSCQIEGVNHRFLVDT
124 NP_057933.2         -------------------------------EPRITLKVGGQPVTFLVDT
125 YP_009116631.1      ------------RRSARMRHQAQQRLRRTFGRDFR----------NTIER
126 YP_009513249.1      ----------------------------MDLLKPLTVERKGVKIKGYWDS
127 YP_223871.1         -------------------------------EPRLKVKVGGQIIDFLVDT
128 NP_127504.1         -MTSPSDYQSNSSLATTYSNAPKLSKALSNKYDYLYEVDILKENQKISDT
129 NP_044929.1         ----------------------------MPALRPLQVEIKGNHLKGYWDS
130 NP_057860.1         QDPTHWKRDCPRLKPTIPEPEPEEDALLLDLPADIPHPKNLHRGGGLTSP
131 NP_040973.1         --------------------------------------------------
132 NP_777317.1         ------------RSIRRTAYEARRRAGMAGRTMGRML--GQQPSNRAAPS
133 NP_054716.1         ----------------------------MQALQPLQVQIKGNSLKGFYDT
134 YP_001856242.1      --------------------------------------------------
135 NP_954565.2         GRSSITMKGLQVYPGVIDNDYTGEIKIMAKAVNNIVTVSQGNRIAQLILL
136 NP_569150.1         ------------RSSRRAAYEAQRIARQTGNIIGRVV--GRQPREHTLSM
137 NP_659397.1         --------------------------------------------------
138 YP_009513211.1      --------------------------------------------------
139 YP_233107.1         ------------RRSARARYEAQRMGRNLVNIGRTII--GRRPREHTLAL
140 YP_009109692.1      -------------------------------EPRLTLSVGGHPTTFLVDT
141 NP_057849.4         ANFLREDLAFLQG----KAR-----EFSSEQTRANSPTRRELQVWGRDNN
142 NP_049560.1         --------------------------------------------------
143 YP_009506267.1      ------------RLGARRRYNMSQAAQKLAKTLTFG----K-YSQPTLEQ
144 NP_039820.1         ------------RNDQRRRHIWAGRGRRLLSIMP-----GVSSSERTLEM
145 NP_056790.1         -------------------------------EPRVTLTVEGTPIEFLVDT
146 YP_002321513.1      ------------RRMRRMGYETIRAIRQPLNRIFAR-------RREALLS
147 NP_955591.1         --------------------------------------------------
148 YP_001956722.2      -------------------------MDPLQLLQPLEAEIKGTKLKAHWDS
149 NP_056902.1         -----MQKCSKKREARGSREAPETNFPDTTEESAQQICCTRDSSDSKSVP
150 NP_056886.1         GSPGHYQAQCPKKRKSGNSRERCQLCNGMGHNAKQCRKRDGNQGQRPGKG
151 NP_041261.1         --------------------------------------------------

001 YP_004442836.1      VVNPNSELSRSLAHRARTVPGEVLYMTQRDSP-VNRVYRNRTEERM-LVT
002 NP_056728.1         --------------------------------------------------
003 YP_009508537.1      QADITCVPKDLLQEEEPVRQQNVTTIHGTQEEDVYYVNLKIDGRRINTEV
004 YP_004732983.2      RLDPEAELQLSRRRRANLVPAEVLYSLNYNEP-QNRVYQHYEEVRS--HV
005 YP_009508407.1      --------------------------------------------------
006 NP_040939.1         KERHKGKTAGKREEGDTCGAVRSSYG----ITSAPPMVQVRIGSQQRNLL
007 YP_003864102.1      --------------------------------------------------
008 YP_009002585.1      EVDPQADLQMSMQERARLVPAEVLYRSRRDTI-HHRVYSHRSEEAI-LCT
009 YP_003284237.1      LMNPEVELQRSMQERARTVPAEVLYMTRRDDI-HHRVYHHRSEERM-LVL
010 YP_009268869.1      ----SNITAGKQQEGATCGAVRAPYV----VTEAPPKIDIKVGTNWKKVL
011 YP_009506251.1      EIDPQVTLRLSMQERARLVPAEVLYRSRRDTV-HHRVYTHRSEESI-LCV
012 NP_955611.1         --------------------------------------------------
013 YP_008567619.1      SLDPQKQLMRSMTQRASIVPAEVLYHSRKDDV-NHKVYIHRSEEAL-SVT
014 NP_149413.1         VMSPEAELERSLKKRARAFPAEVIYSPRRDDI-HHRVFVGSSSQDV-MIV
```

```
015 YP_004442824.1    VVNPDSELSRSLAHRARTVPAEVLYMTQRDSP-VNRVYRNRTEERM-LVT
016 YP_001931967.1    --------------------------------------------------
017 YP_002455786.1    --------------------------------------------------
018 YP_009109694.1    --------------------------------------------------
019 NP_789739.1       --------------------------------------------------
020 NP_955619.1       PTLQQVLPNQDPTSILPVIPLDPARRPVIKAQIDTQTSHPKTIEA----L
021 YP_224289.1       LMDPEVELQRSMRERARTVPAEVLYMSRRDDV-HHRVYHFRSEERM-MVS
022 YP_009182100.1    --------------------------------------------------
023 NP_705927.1       --------------------------------------------------
024 YP_009508577.1    GAQITCIPEAFLKEEIPIGEAQIKTLHGTKLQSVYYLKFKVLGRKVEAEV
025 YP_004442830.1    VLDPEAELEKSMKRRAKAFPAEVVYAPRSDNK-LHKVFQGRTSQDI-MVV
026 NP_758887.1       ATAESGKQTGKEREGSIRGS----LQLPQFSLWNRPTTVVEIEGQKVEAL
027 YP_009508406.1    --------------------------------------------------
028 YP_610965.1       HIDPNEQLQLSQKKKAAIVPAEVLYDDGWHST-VHKVYQHYDEQRVLVLE
029 YP_001931961.1    --------------------------------------------------
030 YP_241114.1       --------------------------------------------------
031 YP_006732334.1    --------------------------------------------------
032 YP_009041481.1    ELDPNMELSASSRRRATMVPAEVLYSTGDTRV-RNRVYEHYSEQRG--LI
033 YP_001036293.1    QVDPERTLQLSAIKRASMIPAEVLYHARPDTV-NHKVYMHWSEEAD-LIV
034 YP_605811.1       VVDPNSELERSLAHRARTIPAEVLYMTQRGEP-TNRVYRNRTEERM-LVT
035 NP_955577.1       GAQHSVLTRPDGPLSDRTALVQGATG-SKNYRWTTDRRVQLATGKVTHSF
036 YP_003208050.1    ELDPEAEIQLSQRRRANMVPAEVLYEEGWSSTRLHRVYQHYSEERILST-
037 NP_789740.1       SPSEAG----ADRQGTVS------FNFPQVTLWQRPLVTIKIGGQLKEAL
038 YP_004442839.1    IVDPNSELERSLAHRARTIPAEVLYMTQRGEP-TNRVYRNRTEERM-LVT
039 YP_009507791.1    --------------------------------------------------
040 YP_004581513.1    --------------------------------------------------
041 NP_043933.1       --------------------------------------------------
042 YP_009507248.1    -MIPARSSKLVRIPINATDGEVLVEEQMFCNCIVHECVTMVKDGRGYVEL
043 YP_002117531.1    LMDPEAELTRSMQERARTVPAEVLYMTRRDDI-HHRVYYHRSEERL-LII
044 YP_004347415.1    --------------------------------------------------
045 NP_056891.1       PLIETDNKVQQPYRGQGSF-GSSDIYWVQPITCQKPSLTLWLDDKMFTGL
046 YP_009513242.1    GATITCIPEAFLEDEQPIQTMLIKTIHGEKQQNVYYLTFKIQGRKVEAEV
047 YP_009352866.1    EINPNRELSASSRRRASMVPAEVLYNSGDTNV-RNRVYEHYSEQRG--LI
048 YP_009165750.1    --------------------------------------------------
049 NP_040840.1       ----AEETAGKQQEGATCGAVRAPYV----VTEAPPKIEIKVGTRWKKLL
050 YP_004222728.1    --------------------------------------------------
051 YP_009506264.1    LMDPDVELQRSMSERARTVPAEVLYMTRRDDV-HHRVYHHRSEERM-LVL
052 YP_567050.1       --------------------------------------------------
053 YP_009508571.1    GASITCIPESFLEEETPIKKTIIKTIHGQKEQKVYYLTFKVNGRKVEAEV
054 NP_056803.1       GATITCIPESFLEDEQPIKQTLIKTIHGEKQQNVYYLTFKVKGRKVEAEV
055 NP_955564.1       -------------------------HWVQEISDSRPMLHIYLNGRRFLGL
056 YP_009508411.1    QVDPEKTLQVSMYQRASLVPAEVLYHARPDTI-HHKVYLHWSEEAV-LVI
057 YP_009140788.1    TIDPEEELSQSMQERANLVPAEVLYRTRRDNI-NHQVYNHRSEEAM-LCV
058 YP_009508410.1    --------------------------------------------------
059 NP_040333.1       GAQHSVLTQNPGPLSDKSAWVQGATG-GKRYRWTTDRRVHLATGKVTHSF
060 YP_008992013.1    TYPLISPYHLYKRPNSFTRS-IRTLISTKRPHPKEYIQSSRLDQCALKAT
061 NP_041734.1       EIDPQTTLRLSMQERARLVPAEVLYRSRRDTV-HHRVYTHRSEESV-LCV
062 NP_056895.1       PQTVTPITDPLSEAELECLLSIPLARSRPSVAVYLSGPWLQPSQNQALML
063 YP_009109689.1    GAQHSVLTKANGPLSSRTSWVQGATG-RKMHKWTNRRTVNLGQGMVTHSF
064 YP_002519387.1    --------------------------------------------------
065 YP_595725.1       LMQPDVELTRSMKKRARAFPAEVLYGPRSDDI-HHKVFQGSSSQDI-LLI
066 YP_009508551.1    GATITCIPESFLEDEQPIKKTLIKTIHGEKQQNVYYVTFKVKGRKVEAEV
067 YP_001497148.1    GAQHSVLTKANGPLSSRTSWVQGATG-RKIHKWTNRRTVDLGQGTVTHSF
068 NP_041186.1       --------------------------------------------------
069 NP_663784.1       IQRGDGGLAAPRAERDTSQRGDRGLAAPQFSLWKRPVVTAYIEDQPVEVL
070 YP_009508443.1    QVDPEKTLQLSSVQRASLVPAEILYHARTDTV-NHKVYMHWSEESD-LII
071 YP_003987465.1    VIDPEAELQRSMSERARTVPAEVLYMTRRDNI-HHRVYYFRCEERM-RVL
072 NP_861410.1       --------------------------------------------------
073 YP_009345071.1    QIDPQAELSMSLQRRASIVPAEVLYHSRQDDI-NHRVYQHWSEEAV-NCI
074 YP_009508582.1    GAQITCIPEAFLKEEIPIGEAQIKTLHGTKLQPVYYLKFKILGRKVEAEV
075 NP_569141.1       --------------------------------------------------
076 YP_002916057.1    --------------------------------------------------
077 NP_777384.2       PQTVTPITDPLSEAELECLLSIPLARSRPSVAVYLSGPWLQPSQNQALML
078 YP_233110.1       VVDPNSELERSLAHRARTIPAEVLYMTQRGEP-TNRVYRNRTEERM-LVT
079 NP_542258.1       --------------------------------------------------
080 YP_009130664.1    QIDPQVHLRNSMQERAAIVPAEVLYHSRCDDE-HHRVYVHRSEEAL-LCT
081 YP_009508566.1    GAQITCIPQVYLEQEKPIGKHVIETVNGKTQRDAYYIKLKINGKKIETEV
082 YP_006607892.1    --------------------------------------------------
083 YP_009506270.1    VVDPNSELSRSLAHRARTVPGEVLYMTQRDSP-VNRVYRNRTGERM-LVT
084 NP_042513.1       --------------------------------------------------
085 YP_006907834.1    --------------------------------------------------
086 YP_004442827.1    ILDPEAELEKSMKRRAKAFPAEVVYAPRYDNK-LHKVFQGRSSQDI-MVV
087 YP_009408594.1    PVDSHDLPEIPDDQDSEQFLAILTFTGDPQYLLPDPVYDTSSD-------
```

```
088 YP_009508561.1     GAQITCIPQSFLLDEQPIGTTDIETIHGKQKQKLYYLKFKVLGRKVEAEV
089 NP_056907.1        -PGGGDVISPQQISMLPVIPLEQQHQPLLDVQVSIAGAPPRPTQA----L
090 YP_002308474.1     --------------------------------------------------
091 YP_007761644.1     --------------------------------------------------
092 NP_040550.1        --------------------------------------------------
093 NP_758808.1        QINPEAEIRLSQQRRAAMVPAEVLYNTSPSTR-NQKVYQHYSEERILCTG
094 YP_009345075.1     EIDPEAQLRLSTQQRAGIVPAEVLYRNGWSER-AHRVYRHYSEERIMLTN
095 NP_068729.1        --------------------------------------------------
096 NP_047255.1        GAQHSVLTRPDGPLSDRTALVQGATG-SKNYRWTTDRRVQLATGKVTHSF
097 YP_006273075.1     IVDPEVELNQSMQERANLVPAEVLYRSRRDNI-NHQIYNHRSEEAM-LCV
098 YP_009508888.1     GTTITCIPTVFLTDEIPIKDVLIKTIHGERRQPAYYLTFKINGRKVQAEV
099 NP_395469.1        --------------------------------------------------
100 YP_009508546.1     GATITCVPESFLEDEQPIKQTLIKTIHGEKQQKVYYLTFKIKGRKVEAEV
101 NP_056762.1        VDVNQEVENFDWKKLSGIKPNKLYEKNWQEKVKLKQQSIVSAYKEEAISV
102 YP_009508556.1     GATITCIPEAFLEDEQPIQTMLIKTIHGEKRQNVYYLTFKIQGRKVEAEV
103 NP_569153.1        VVDPEVALQLSMQERARLVPAEVLYRSRTDDR-HHQVYIHKSEEAI-LCV
104 NP_043924.1        GGAWDPTRETGSARWKKIVKAAEAIGWGTGDVCQVVTAMSPSWADVPPEI
105 YP_009243641.1     --------------------------------------------------
106 YP_004300274.1     --------------------------------------------------
107 NP_056848.1        --------------------------------------------------
108 YP_006495799.1     QLSPNQELALSRRRRANLVPAEVLYTHNNNEV-VNRVYQHYEERSA--HV
109 YP_009508409.1     --------------------------------------------------
110 NP_687035.1        TAPERGERTDKETEGERSG-----GCFLELPLWRRPMKRVIIEGTPVQAL
111 NP_045937.2        TIKKDGTSVQYQHPIRNYPTGEGNLTAQVRNPFRPLELQQLRKDCPALPE
112 NP_612577.1        --------------------------------------------------
113 NP_619548.1        --------------------------------------------------
114 NP_056880.1        PYLKLPNPVIKEERGSEGFGSTSHVHWVQEISDSRPMLHIYLNGRRFLGL
115 YP_009121747.1     ELDPDQQLEVSRQRRAELVPAEVLYGSNSQTV-RHRVYQHYSEQRILCTE
116 YP_009113237.2     EVDPEATLRLSMQERARLVPAEVLYRSRSDDV-NHRVYIHRDEEAV-LCT
117 YP_009508408.1     QVEPEKTLQLSSVQRASLVPAEVLYHSRPDTV-NHKVYMHWSEESD-LII
118 NP_955579.1        --------------------------------------------------
119 YP_009229919.1     RIDPDAQLQISRRRRSDMVPAEVLYEAQRDNP-IHRVYQHYSERRIRVTE
120 YP_004442833.1     VMDVEAELEKSMKRRARAFPAEVVYSPRRDNK-LHKVFQGRASQDL-MVV
121 NP_862833.2        --------------------------------------------------
122 NP_040563.1        CPLWAEEPTTEQFSPEQHEFCDPICTPSYIRLDKQPFIKVFIGGRWVKGL
123 YP_443922.1        GCTYSAIKT-MQPLSMDSIQVVGVSGRPETQLKTQPLLFRWGPSTVRHQF
124 NP_057933.2        GAQHSVLTQNPGPLSDKSAWVQGATG-GKRYRWTTDRKVHLATGKVTHSF
125 YP_009116631.1     QLDPDAELSLSRRRRANLVPAEVLYAHNGQEP-VNRVYEHYSELSA--HV
126 YP_009513249.1     QADITCVPKDLLQGEEPVRQQNVTTIHGTQEGDVYYVNLKIDGRRINTEV
127 YP_223871.1        GATHSVVQKPVGPMSKESVAIIGATGNIRNYPKSEGRLVDLGRGLVTHSF
128 NP_127504.1        YLPLLNPYSAFAKRSVTPWSQIRSLVQSKPRHVKEYVAASKLDQHPVFAT
129 NP_044929.1        GAEITCVPAIYIIEEQPVGKKLITTIHNEKEHDVYYVEMKIEKRKVQCEV
130 NP_057860.1        PTLQQVLPNQDPTSILPVIPLDPARRPVIKAQIDTQTSHPKTIEA----L
131 NP_040973.1        --------------------------------------------------
132 NP_777317.1        IVNPDEELERSLTRRARTIPAEVLYLAQRREV-DNRVYRNMTEERM-LIT
133 NP_054716.1        GAEITCVPAIFLIEEEPIGERTIQTIHGITKEKVYYLTFKIQGRKLAAEV
134 YP_001856242.1     --------------------------------------------------
135 NP_954565.2        PLIETDNKVQQPYRGQGSF-GSSDIYWVQPITCQKPSLTLWLDDKMFTGL
136 NP_569150.1        VVDPNSELSRSLAHRARTVPGEVLYMTQRDSP-VNRIYRNRTEERM-LVT
137 NP_659397.1        --------------------------------------------------
138 YP_009513211.1     ----------MGKMGSKRTVVAGATG-SKVYPWTTKRLLKIGQKQVTHSF
139 YP_233107.1        LMDPEVELRRSMQERARTVPAEVLYMTRRDDI-HHRVYHYRSEEGM-LIT
140 YP_009109692.1     GAQHSVLTKANGPLSSRTSWVQGATG-RKMHKWTNRRTVNLGQGMVTHSF
141 NP_057849.4        SPSEAG----ADRQGTVS------FNFPQVTLWQRPLVTIKIGGQLKEAL
142 NP_049560.1        --------------------------------------------------
143 YP_009506267.1     QVDPEKTLQLSSMQRASLVPAEVLYHSRPDTV-NHKVYMHWSEESD-LVV
144 NP_039820.1        QMNPEVQLQRSMNHRAEAVPAEVLYRTFHGSV-NHRVYSHRSEERM-MVV
145 NP_056790.1        GAEHSVLTQPMGKVGSRRTVVEGATG-SKVYPWTTKRLLKIGHKQVTHSF
146 YP_002321513.1     PEQVSRRLQRSGSRRPEAVPAEVLYTAGQDSA-QTRVYESVAEESI-LVI
147 NP_955591.1        --------------------------------------------------
148 YP_001956722.2     GATITCVPQAFLEEEVPIKNIWIKTIHGEKEQPVYYLTFKIQGRKVEAEV
149 NP_056902.1        RSERNKKGIQCQGEGSSRGSQPGQFVGVTYNLEKRPTTIVLINDTPLNVL
150 NP_056886.1        LSSGPWPGPEPPAVSLAMTMEHKDRPLVRVILTNTGSHPVKQRSVYITAL
151 NP_041261.1        --------------------------------------------------


001 YP_004442836.1     NG-QQDRSFIYPESFEELVNAGFEYIHLGVLQVRLQIMHRTYAGTMALVV
002 NP_056728.1        --------------------------------------------------
003 YP_009508537.1     IGTALDYAIITPGDAPWILKKPLELT------------------------
004 YP_004732983.2     IDRQQDFRFIEEQSYRTLVQEGMQHIHPGMLMVRIQVLHRVDAGISAMIV
005 YP_009508407.1     --------------------------------------------------
006 NP_040939.1        FDTGADRTIVRWHEGSGNPAGRIKLQGIGGIV------------------
```

```
007 YP_003864102.1    --------------------------------------------------
008 YP_009002585.1    DGNQVDRTFIQPESMEALQRTGISFIHIGIMQVRLQILHRQNEGTMALVV
009 YP_003284237.1    RNDQQDRTFIQQESYEALAQAGLEYIHLGVLQVRFQILHRRYAGTVAFLV
010 YP_009268869.1    VDTGADRTIVRYHDNSGIPTGRIKLQGIGGII------------------
011 YP_009506251.1    GGNQVDRTFIQPSSLERLQRTGMSFIHIGILQVRIQILHRQEEGTMALVV
012 NP_955611.1       --------------------------------------------------
013 YP_008567619.1    EGNQVDRTFIQEESHKKLVKSGIKFIHLGVLQVRLQILHRKEEGTLALVV
014 NP_149413.1       DDNQIDMTYIKEESFEKLEQAGFRYIHLGAMAIRIQPLHAAWSGKLAFIV
015 YP_004442824.1    NG-QQDRTFIYPESFEELVNAGFEYIHLGVLQVRLQIMHRTYAGTIALVV
016 YP_001931967.1    --------------------------------------------------
017 YP_002455786.1    --------------------------------------------------
018 YP_009109694.1    --------------------------------------------------
019 NP_789739.1       --------------------------------------------------
020 NP_955619.1       LDTGADMTVLPIALFSSNTPLKNTSVLGAGGQ------------------
021 YP_224289.1       DADQQDRTFISEESFERLQQAGLTYIHLGVLQVRFQILHRTFAGTMALLV
022 YP_009182100.1    --------------------------------------------------
023 NP_705927.1       --------------------------------------------------
024 YP_009508577.1    TTSPFDYVIISPSDIPWYKPQPLELT------------------------
025 YP_004442830.1    DDNQLDMTFIREETFEQLERAGLRYIHIGTLQVRIQPLHQRWSGTMAFIA
026 NP_758887.1       LDTGADDTVIKDLDLKG----NWKPQIIGGIG------------------
027 YP_009508406.1    --------------------------------------------------
028 YP_610965.1       -GQQVELPFINEQSYRQLLSSGFQHIHVGMVLVRVHALHSRHAGTMALLC
029 YP_001931961.1    --------------------------------------------------
030 YP_241114.1       --------------------------------------------------
031 YP_006732334.1    --------------------------------------------------
032 YP_009041481.1    VNNQEDFRFIQESSYNTLRNSGYEHMHVGMMMMRVLTLHRTYTGVTALVT
033 YP_001036293.1    NN-QHDSTFIRPESYLELRKANMQFIHIGLMQVRIQVLHRHEAGTMALIV
034 YP_605811.1       HG-QQDRTFILPESYEELREAGFEYIHLGVLQVRIQIMHRSYAGTMALVV
035 NP_955577.1       LYVPECPYPLLGRDLLTKLKAQIHFTGEGANV------------------
036 YP_003208050.1    -GEQVELPFVTRRSYERLQRTGYQQIHLGLVLIRVHTLHRRDAGVKALVV
037 NP_789740.1       LDTGADDTVLEEMSLPG----RWKPKMIGGIG------------------
038 YP_004442839.1    QG-QQDRSFILQESYEALLEAGFEYIHLGVLQVRIQIMHRSYAGTMALVV
039 YP_009507791.1    ------------------------MQGVGGTN------------------
040 YP_004581513.1    --------------------------------------------------
041 NP_043933.1       --------------------------------------------------
042 YP_009507248.1    ENPTPNDVIFYLDQPASAELFNIKCTQVEQSQ------------------
043 YP_002117531.1    GNTQVDRTFIPQQSYEALSKEHMEYIHLGVIQVRLQILHRKFAGTLALIS
044 YP_004347415.1    --------------------------------------------------
045 NP_056891.1       IDTGADVTIIKLEDWPPNWPITDTLTNLRGIG------------------
046 YP_009513242.1    LASPYDYILLNPSDVPWLMKKPLQLT------------------------
047 YP_009352866.1    VNSQEDFRYIQESSYNTLRNSGYEHIHIGMMMVRVLTLHRTHAGVKALVT
048 YP_009165750.1    --------------------------------------------------
049 NP_040840.1       VDTGADKTIVTSHDMSGIPKGRIILQGIGGII------------------
050 YP_004222728.1    --------------------------------------------------
051 YP_009506264.1    ANDQQDRTFIVRESYEALERAGFEYVHLEVMQVRFQILHRRFAGTLAFIV
052 YP_567050.1       --------------------------------------------------
053 YP_009508571.1    IASPYDYILLSPMDVPWLVQKPLQLT------------------------
054 NP_056803.1       IASPYEYILLSPTDVPWLTQQPLQLT------------------------
055 NP_955564.1       LDTGADKTCIAGRDWPANWPIHQTESSLQGLG------------------
056 YP_009508411.1    QG-QYDSTFIKTESYEALQRAGMQFIHIGLMQVRIQVLHRLEAGTMALVA
057 YP_009140788.1    G-EQQDRSFIQPDSFQELQRSGMQFIHLGVLQVRIQILHRADEGTMALVV
058 YP_009508410.1    --------------------------------------------------
059 NP_040333.1       LHVPDCPYPLLGRDLLTKLKAQIHFEGSGAQV------------------
060 YP_008992013.1    SAEQYVTLEIPSELISNWKREGYTHLHLGGIRLILTLHGRKGLPVTARVA
061 NP_041734.1       GGSQVDRAFIQPESLEQLQRTGMSFIHIGILQVRIQILHRQEEGTMALVV
062 NP_056895.1       VDTGAENTVLPQNWLVRDYPRIPAAVLGAGGV------------------
063 YP_009109689.1    LVVPECPYPLLGRDLLTKLGAQIHFSEAGAQV------------------
064 YP_002519387.1    --------------------------------------------------
065 YP_595725.1       DDNQLDMTFIKEETFEQLEQAGLRYIHPGILAVRIQPLHPDWSGKLVFIV
066 YP_009508551.1    IASPYEYILLSPTDVPWLTQQPLQLT------------------------
067 YP_001497148.1    LVVPECPYPLLGRDLLTKLGAQIHFSETGAQV------------------
068 NP_041186.1       --------------------------------------------------
069 NP_663784.1       LDTGADDSIVAGIELGDN----YTPKIVGGIG------------------
070 YP_009508443.1    NG-QHDSTFIRPESFQVLQRSGMQFIHIGLMQVRIQVLHRLEAGTMAMVV
071 YP_003987465.1    DSDQQDRTFITPEAFEHLKEAGFEYIHLGILQVRFQILHRRYAGTMAFIA
072 NP_861410.1       --------------------------------------------------
073 YP_009345071.1    DGQQIDRTLVTMESYSTLNRAGFRYIHIGVIQVRLQILHRREMGTLAYVV
074 YP_009508582.1    TTSPFDYVIISPSDIPWYKPQPLELT------------------------
075 NP_569141.1       --------------------------------------------------
076 YP_002916057.1    --------------------------------------------------
077 NP_777384.2       VDTGAENTVLPQNWLVRDYPRIPAAVLGAGGV------------------
078 YP_233110.1       HG-QQDRTFILPESYEELREAGFEYIHLGVLQVRIQIMHRTYDGTMALVV
079 NP_542258.1       --------------------------------------------------
```

```
080 YP_009130664.1    DNQQQDRIFIQEESFKQLQRSRMQFIHIGVIQLRIQALHRHDEGTLALVV
081 YP_009508566.1    IPSPFSYALITPNDIPWFKPGGIELT------------------------
082 YP_006607892.1    --------------------------------------------------
083 YP_009506270.1    NG-QQDRSFIYPESFEELVAAGFEYIHLGVLQVRLQIMHRTYAGTMALVV
084 NP_042513.1       --------------------------------------------------
085 YP_006907834.1    --------------------------------------------------
086 YP_004442827.1    DDNQLDMTFIKEETFEQLERAGLRYIHIGALQVRIQPLHQKWYGTMAFLA
087 YP_009408594.1    --SDSDSDTESTTSDGPLPDLGPPDHVWATDPRPSYAVWEENRSGNCPKA
088 YP_009508561.1    TSSTLAYVILAPIDIPWYKPTPLELT------------------------
089 NP_056907.1       LDTGADLTVLPQALAPESVSVSDTTVLGAGGQ------------------
090 YP_002308474.1    --------------------------------------------------
091 YP_007761644.1    --------------------------------------------------
092 NP_040550.1       --------------------------------------------------
093 NP_758808.1       QNQQLNLPFINESSYRALRESGQQHLHIGLIMIRVHPLHRRNAGTTALIV
094 YP_009345075.1    PNQQRELRVITDQSYQKLREEGKQHVHCGLVMIRVHALHRKQAGVMVLVV
095 NP_068729.1       --------------------------------------------------
096 NP_047255.1       LYVPECPYPLLGRDLLTKLKAQIHFTGEGANV------------------
097 YP_006273075.1    G-EQQDRMFIQPNSFQELQRSGMQFIHLGVLQVRLQILHRADGGTMALVV
098 YP_009508888.1    IASPYDYILLCPADVPWLQQQPLQLT------------------------
099 NP_395469.1       --------------------------------------------------
100 YP_009508546.1    IASPYEYILLSPTDVPWLTQQPLQLT------------------------
101 NP_056762.1       THNAYTTTLFPQEVIKNVKNQGKLYYHIGMMAIGVKGLHRRKIGTKVMIM
102 YP_009508556.1    LASPYDYILLNPSDVPWLMKKPLQLT------------------------
103 NP_569153.1       DGDQVDRLLIQPESAEQLSRSGMSFIHMGIVQVRIQILHRQHEGTTALVV
104 NP_043924.1       RNRVATEKEIKAWLMKQGPGGGQGLLEFTKLRQGPTENPSNYLEKALELY
105 YP_009243641.1    --------------------------------------------------
106 YP_004300274.1    --------------------------------------------------
107 NP_056848.1       --------------------------------------------------
108 YP_006495799.1    VDRQMDLRFIEEESYRRLVDEGLQFIHLGMGMVRIHMLHRNSAGIEAMIV
109 YP_009508409.1    --------------------------------------------------
110 NP_687035.1       LDTGADDTIIQEKDLHFPPHKPWRSKVVGGIG------------------
111 NP_045937.2       GIPQLAEWLTQTMAIYNCDEADVEQLARVIFPTPVRQIAGVINGHAAANT
112 NP_612577.1       --------------------------------------------------
113 NP_619548.1       --------------------------------------------------
114 NP_056880.1       LDTGADKTCIAGRDWPANWPIHQTESSLQGLG------------------
115 YP_009121747.1    ENQ-TDLRLCNRQSYQSLRESGMQHIHLGMFMIRILTMHRRGAGTTALIV
116 YP_009113237.2    GDAQVDRALIQPQSAERLLQSGYSFIHIGIIQARIQILHRSQEGTMALVL
117 YP_009508408.1    NG-QHDSTFIRPESYQLLQRSGMQFIHIGLMQVRVQVLHRLEAGTMAMIV
118 NP_955579.1       --------------------------------------------------
119 YP_009229919.1    EGEQEDLRFITEPSYGTLRREGFQHIHLGLMMVRVHGLHARHAGTQVLVV
120 YP_004442833.1    DDNQMDMTFIKEETFEQLERAGLRYIHIGALQVRIQPLHQRWSGKMAFLA
121 NP_862833.2       --------------------------------------------------
122 NP_040563.1       VDTGADEVVLKNIHWDRIKGYPGTPIKQIGVN------------------
123 YP_443922.1       LYCPNCPINLLGRDLLCRLGCFINLTEDGVEVSINSPKSGRVLLLPSLPV
124 NP_057933.2       LHVPDCPYPLLGRDLLTKLKAQIHFEGSGAQV------------------
125 YP_009116631.1    VDRQQDFRFIEEASYQRLTREGMQFIHVGMAMVRIQMLHRTDAGISALVV
126 YP_009513249.1    IGTTLDYAIITPGDVPWILKKPLELT------------------------
127 YP_223871.1       LVIPECPDPLLGRDLLQKLRATISFTGGGPPE------------------
128 NP_127504.1       GEEQFVTLHIPEEFASHWKSHQFTHIHFGAVKIALTYHGRKGQPVVARLA
129 NP_044929.1       IATALDYVLVAPVDIPWYKPGPLELT------------------------
130 NP_057860.1       LDTGADMTVLPIALFSSNTPLKNTSVLGAGGQ------------------
131 NP_040973.1       --------------------------------------------------
132 NP_777317.1       DG-QQNRTFIRPESYSTLVASGYEYIHLGVLQVRIQTMHRAFAGTMALVV
133 NP_054716.1       IGTQLDYVIIAPSDIPWYKKYELELT------------------------
134 YP_001856242.1    --------------------------------------------------
135 NP_954565.2       IDTGADVTIIKLEDWPPNWPITDTLTNLRGIG------------------
136 NP_569150.1       NG-QQDRSFIYQESFEELASAGFEYIHLGVLQVRIQIMHRTYAGTMALIV
137 NP_659397.1       --------------------------------------------------
138 YP_009513211.1    LVIPECPAPLLGRDLLTKLKAQIQFSTEGPQV------------------
139 YP_233107.1       GSDQQDRTFITEESYEHLAQAEIEYIHLGILQVRFQILHRRYAGTMALLV
140 YP_009109692.1    LVVPECPYPLLGRDLLTKLGAQIHFSEAGAQV------------------
141 NP_057849.4       LDTGADDTVLEEMSLPG----RWKPKMIGGIG------------------
142 NP_049560.1       --------------------------------------------------
143 YP_009506267.1    NR-QYDSTFIRPESYTTLMKSGMQFIHIGLMQVRIQILHRAEAGTLAMIV
144 NP_039820.1       NGSQVDRSFIQESSFEVLSRTGIEFIHIGVMLVRIQILHRKFAGTMALIV
145 NP_056790.1       LVIPECPAPLLGRDLLTKLKAQIQFSAEGPQV------------------
146 YP_002321513.1    GETQRDLLFMEPQSLQEIRRQNFRHIHIGMVIIKIRPLHTDARGTSALLV
147 NP_955591.1       --------------------------------------------------
148 YP_001956722.2    ISSPYDYILVSPSDIPWLMKKPLQLT------------------------
149 NP_056902.1       LDTGADTSVLTTAHYNRLKYRGRKYQGTGIIG------------------
150 NP_056886.1       LDSGADITIISEEDWPTDWPVMEAANPQIHGI------------------
151 NP_041261.1       --------------------------------------------------
```

```
001 YP_004442836.1   FRDTRWTQEGD-EDRSIIAAMEADLSQG-HQLIYVIPDIMMTIKDFYQHV
002 NP_056728.1      --------------------------------------------------
003 YP_009508537.1   -------------------------------------IKLDLEEQQRTLL
004 YP_004732983.2   FRDTRWNDER-----QIISAMTVDMARG-AQLVYAIPDLMMSIHDFYHHL
005 YP_009508407.1   --------------------------------------------------
006 NP_040939.1      ------------------------------EGEKWNNVELEYKGE-----
007 YP_003864102.1   --------------------------------------------------
008 YP_009002585.1   FRDTRWNGDQ-----SIFATMEVDLTEG-RQIIYVVPDTMMTIGDFARNI
009 YP_003284237.1   FRDTRWNE-DD---RSIIAAMEVDLAEG-NQLVYVIPDIMMTIKDFYRHI
010 YP_009268869.1   ------------------------------EGEKWDKVVIQYKEK-----
011 YP_009506251.1   FRDNRWAGDQ-----AIFAQMEVDLTKG-SQLVFVVPDTMMTIGDFARNV
012 NP_955611.1      --------------------------------------------------
013 YP_008567619.1   FRDNRWKGDK-----GIIATMEVDLTKG-CQMVYIIPDIMMTVNDFYHNI
014 NP_149413.1      LRDVRNSP------PTTLGAMEVDLSKG-AQLIYVLPNFMCTIKDFYHGI
015 YP_004442824.1   FRDTRWTQEGE-EDRSIIAAMEADLSQG-HQLIYVIPDIMMTIRDFYQHV
016 YP_001931967.1   --------------------------------------------------
017 YP_002455786.1   --------------------------------------------------
018 YP_009109694.1   -----------------------------------------TVSLQDEHR
019 NP_789739.1      --------------------------------------------------
020 NP_955619.1      ------------------------------TQDHFKLTSLPVLIRLPFRT
021 YP_224289.1      FRDTRWTA-DD---RSIISAMEVDLAEG-NQLIYVIPNIMMTIGDFYRHI
022 YP_009182100.1   --------------------------------------------------
023 NP_705927.1      --------------------------------------------------
024 YP_009508577.1   -------------------------------------VKLPVQDFKKELI
025 YP_004442830.1   FQDTRSQP------ATVIAAMEVDLSKG-AQMIYIIPDFMTTIGDFYQGI
026 NP_758887.1      ------------------------------GSINVKQFFNCKVTIAG---
027 YP_009508406.1   --------------------------------------------------
028 YP_610965.1      LRDTRWKSSR-----GIIGSMEVDLTAG-SQLVYVVPDILLSVQDFYNHI
029 YP_001931961.1   --------------------------------------------------
030 YP_241114.1      --------------------------------------------------
031 YP_006732334.1   --------------------------------------------------
032 YP_009041481.1   FRDTRWHGEL-----SILGQMELDLSEG-SQLVFTTPNLLCSIHDFYHHI
033 YP_001036293.1   FRDCSWKGRR-----STIARMEVDLTKG-SQLVYIAPNITKKLTDFYNNI
034 YP_605811.1      FRDTRWTQENH-QGRSIIATMEADLSQG-HQLIYVIPDIMMTIRDFYQHI
035 NP_955577.1      ------------------------------VGPKGLPLQVLTLQLEEEYR
036 YP_003208050.1   FRDTRWRDDR-----SIIGSMEIDLSIG-TQLAYIAPDMMMSIHDFYNHV
037 NP_789740.1      ------------------------------GFIKVRQYDQILIEICG---
038 YP_004442839.1   FRDTRWTQEDA-EDRSIIAAMEADLSQG-HQLIYVIPDIMMTIRDFYQHI
039 YP_009507791.1   ------------------------------RGEKYINVHFEAYFDKEVRC
040 YP_004581513.1   --------------------------------------------------
041 NP_043933.1      --------------------------------------------------
042 YP_009507248.1   -----------------------------------------RVDDVLSRL
043 YP_002117531.1   FRDTRWAGDDD---RQLISVMEVDLSEG-CQLIYVIPDMMLTLKDFYRHI
044 YP_004347415.1   --------------------------------------------------
045 NP_056891.1      ------------------------------QSNNPKQSSKYLTWRDKENN
046 YP_009513242.1   -------------------------------------VLVPLQDYQERLL
047 YP_009352866.1   FRDTRWQGEL-----SILGQMELDLSEG-SQPVFTAPNLMCSIHDFFHHI
048 YP_009165750.1   --------------------------------------------------
049 NP_040840.1      ------------------------------EGEKWEQVHLQYKDK-----
050 YP_004222728.1   --------------------------------------------------
051 YP_009506264.1   FRDTRWHE-DD---TSIIAAMEVDLAEG-NQLVYVIPDIMMTIKDFYRHI
052 YP_567050.1      --------------------------------------------------
053 YP_009508571.1   -------------------------------------ILVPLQDYQSRIL
054 NP_056803.1      -------------------------------------ILVPLQEYQDRIL
055 NP_955564.1      ------------------------------MACGVARSSQPLRWQHEDK-
056 YP_009508411.1   FRDCSWKGRR-----SIIAKMEIDLSKG-SQLVYVAPDITKRVADFYRNI
057 YP_009140788.1   FRDNRWQGDQ-----SIFAIAEVDLTRG-SQIVYVIPDTMMTIGDFYRNV
058 YP_009508410.1   --------------------------------------------------
059 NP_040333.1      ------------------------------VGPMGQPLQVLTLNIEDEYR
060 YP_008992013.1   LLDTRFKQFQD----AVIGTVLTTLHAG-SVLLTFYPNFNLSLQDPNLPT
061 NP_041734.1      FRDNRWSGDQ-----SIFAQMEIDLTKG-SQLVFVIPDTMMTIGDFARNV
062 NP_056895.1      ------------------------------SRNRYNWLQGPLTLALKPEG
063 YP_009109689.1   ------------------------------LDRDGQPIQILTVSLQDEHR
064 YP_002519387.1   --------------------------------------------------
065 YP_595725.1      FRDIRDNP------PRVLGAMEIDLSKG-PQMVYVINSFMTTIKDFFHGI
066 YP_009508551.1   -------------------------------------ILVPLQEYQEKIL
067 YP_001497148.1   ------------------------------LNRDGQPIQVLTVSLHDEHR
068 NP_041186.1      --------------------------------------------------
069 NP_663784.1      ------------------------------GFINTKEYKNVEIKVLN---
070 YP_009508443.1   FRDCSWKGKR-----SIIARMEVDLSKG-SQLVYIAPNIIKKLADFYNNI
```

```
071 YP_003987465.1    YRDTRWD--NR---KSIIATMEVDLSEG-NQIVYIIPDMMTTIRDCYEHI
072 NP_861410.1       --------------------------------------------------
073 YP_009345071.1    FRDNRLLGDQ-----AILAQMEVSLADGGHQMIYVVPDIMMTIGDFYRNI
074 YP_009508582.1    -------------------------------------VKLPVQDFKKELI
075 NP_569141.1       --------------------------------------------------
076 YP_002916057.1    --------------------------------------------------
077 NP_777384.2       ------------------------------SRNRYNWLQGPLTLALKPEG
078 YP_233110.1       FRDTRWTQENH-QDRSIIATMEADLSQG-HQLIYVIPDIMMTIRDFYQHI
079 NP_542258.1       --------------------------------------------------
080 YP_009130664.1    FRDNRWPDDR-----SLFATMEVDLSQG-SQLVYVIPNTMMTIGDFYNNV
081 YP_009508566.1    -------------------------------------EKLPIQDYKDNIV
082 YP_006607892.1    --------------------------------------------------
083 YP_009506270.1    FRDTRWTQEGE-EDRSIIAAMEADLSQG-HQLIYVIPDIMMTIRDFYQHV
084 NP_042513.1       --------------------------------------------------
085 YP_006907834.1    --------------------------------------------------
086 YP_004442827.1    FQDTRSQP------AKVIAAMEIDLSKG-AQMVYITPDFMTTIGDFYQGI
087 YP_009408594.1    FWRS-YQPPA-----SYLDPVPDPSTAS-WGPDFDEP----LVPPEGGGE
088 YP_009508561.1    -------------------------------------IKLPVQDLKNTLV
089 NP_056907.1       ------------------------------TSSQFKLLQSPLCVYLPFRR
090 YP_002308474.1    --------------------------------------------------
091 YP_007761644.1    --------------------------------------------------
092 NP_040550.1       --------------------------------------------------
093 NP_758808.1       PRDIRWNDDR-----SIIGTMEIDLSAG-SQIVYIAPNIMLSVEDFYRNI
094 YP_009345075.1    IRDSRWGSDPR----GILGQMEIDLSTG-TELVFFVPDQMMTIKDFYDHF
095 NP_068729.1       --------------------------------------------------
096 NP_047255.1       ------------------------------VGPKGLPLQVLTLQLEEEYR
097 YP_006273075.1    FRDNRWQGDQ-----SIFAISEVDLTRG-SQIVYVIPDTMMIIGDFYRNV
098 YP_009508888.1    -------------------------------------VLVPLEQYKERIL
099 NP_395469.1       --------------------------------------------------
100 YP_009508546.1    -------------------------------------ILVPLQEYQDRIL
101 NP_056762.1       FYDDSFGKAR------EASIGSIEMDMNAGCGVFYSCPDFAKYIKDLSHL
102 YP_009508556.1    -------------------------------------VLVPLQDYQDRLL
103 NP_569153.1       FRDNRWQGDQ-----SIFATMELDLTKG-MQMVYIIPDTMMTVRDFCRNV
104 NP_043924.1       LDSQPGDRDGNKDDPAFLQQATQGLLPWLKKAVILGGKNTSWQEMTSFCQ
105 YP_009243641.1    --------------------------------------------------
106 YP_004300274.1    --------------------------------------------------
107 NP_056848.1       --------------------------------------------------
108 YP_006495799.1    FRDTRWTDDR-----QVIASMSVDMTHG-SQLVYVIPDAMLSIHDFYNHI
109 YP_009508409.1    --------------------------------------------------
110 NP_687035.1       ------------------------------GGIHVKEYQGVQVQLED---
111 NP_045937.2       AAKIQNYVTACRQHYPAVCDWGTIQAFTYKPPQTAHEYVKHAEIIFKNNS
112 NP_612577.1       --------------------------------------------------
113 NP_619548.1       --------------------------------------------------
114 NP_056880.1       ------------------------------MACGVARSSQPLRWQHEDK-
115 YP_009121747.1    LRDTRWGDDR-----QIIGTMEVDLSAG-TQLVYMVPDMVLSIDDFHNHI
116 YP_009113237.2    FRDTRWTGDR-----SIFATMEVDLTEG-SQLTYAIPNTMMTIRDFCNHV
117 YP_009508408.1    FRDCSWKGKR-----STIARMEVDLSKG-SQLVYVAPNIIKRLADFYNNI
118 NP_955579.1       -----------------------------------------TLQLEEEYR
119 YP_009229919.1    LRDTRWPDER-----QVIGTMEVDMSTG-TQLVYVVPDIMMSIEDFYEHI
120 YP_004442833.1    FQDTRSQP------ATIIAAMEVDLSKG-AQMIYITPDFMTTIGDFYQGI
121 NP_862833.2       --------------------------------------------------
122 NP_040563.1       ------------------------------GVNVAKRKTHVEWRFKD---
123 YP_443922.1       PPLSLTQEVYWLKCTQTGPSTPAIQFQFNTWRRLIYALHPYKTPLPEVHC
124 NP_057933.2       ------------------------------MGPMGQPLQVLTLNIEDEHR
125 YP_009116631.1    FRDTRWSDDR-----QVIGSMSVDMTRG-AQLVYIIPNAMMSIHDFYNRI
126 YP_009513249.1    -------------------------------------IKLDLEEQQGTLL
127 YP_223871.1       ------------------------------IRTEGKLLV--TAPLEEEYR
128 NP_127504.1       LLDTRYLEYQH----ANLGTAEITLNAG-TVFITLFPNFTMSLSDANLST
129 NP_044929.1       -------------------------------------IKIDVESQKHTLI
130 NP_057860.1       ------------------------------TQDHFKLTSLPVLIRLPFRT
131 NP_040973.1       --------------------------------------------------
132 NP_777317.1       FRDTRWTREVSGEDRSIIAAMEVDLSRG-HQLIYVIPNIIMTIRDFYQHI
133 NP_054716.1       -------------------------------------IKIDIQKQQEQLL
134 YP_001856242.1    --------------------------------------------------
135 NP_954565.2       ------------------------------QSNNPKQSSKYLTWRDKENN
136 NP_569150.1       FRDTRWTQEGE-EGRSIIGAMEADLSQG-HQLIYVIPDIMMTIRDFYQHV
137 NP_659397.1       --------------------------------------------------
138 YP_009513211.1    ------------------------------TWED-RPAMCLVLNLEEEYR
139 YP_233107.1       FRDTRWNSDDR----SIIAAMEVDLSEG-NQLIYIMPDMMMTIKDFYRHI
140 YP_009109692.1    ------------------------------LDRDGQPIQILTVSLQDEHR
141 NP_057849.4       ------------------------------GFIKVRQYDQILIEICG---
142 NP_049560.1       --------------------------------------------------
143 YP_009506267.1    FRDCSWRGRR-----SMIAKMEVDLTKG-SQMVYIAPNLIKRLTDFYNNI
```

144 NP_039820.1 FRDTRWSD-----DRAVLAAMEIDLSEG-NQIVYVLPDIMMTIKSFYRHI
145 NP_056790.1 ------------------------------TWGE-RPTMCLVLNLEEEYR
146 YP_002321513.1 LRDTRFNDSR-----EILGTMEVDFTNG-AQLVYVAPNYMSTISDFYNHF
147 NP_955591.1 --LNIEDEHR
148 YP_001956722.2 -------------------------------------TLVPLQEYEERLL
149 NP_056902.1 ------------------------------VGGNVETFSTPVTIKK----
150 NP_056886.1 ------------------------------GGGIPMRKSRDMIELGVINR
151 NP_041261.1 --

001 YP_004442836.1 QISILTKGYQG--FQ-GEANLLITRSCRCRLTNVPNVGFAYNIQKVVEYL
002 NP_056728.1 --
003 YP_009508537.1 NNSILS--
004 YP_004732983.2 QVSITTRGYGTG-WVGGESNLIVTRSLTGRITNTSQANFNYQIEGVADYL
005 YP_009508407.1 --
006 NP_040939.1 TRKGTIVVLPQS--------------------------------------
007 YP_003864102.1 --
008 YP_009002585.1 QISILTRGYSN--WQNGEANLLVTRGIVGRLSNTPNVAFAYEIGNVTDYL
009 YP_003284237.1 QISIKTKGYDT--WEGGEANLLITRSITSRLSNTPNVGFAYKIERVAEYL
010 YP_009268869.1 RIEGTIVVLPSS--------------------------------------
011 YP_009506251.1 QLSILTRGYDN--WQNGEANLLVTRSMTGRLSNTPNVAFACQIASATDYL
012 NP_955611.1 --
013 YP_008567619.1 HISVLTRGYEG--WQHGEANLLVTRGMIGRLSNTPNVGFAYDIQEVVDYF
014 NP_149413.1 QLSIKTKGYEG--WQ-GEANLHLERLITGRLSNTSNVAFKYKIDGVTAYL
015 YP_004442824.1 QISVLTKGYNG--FQ-GEANLLITRSCRCRLTNVPNVGFAYNIQKVVEYL
016 YP_001931967.1 --
017 YP_002455786.1 --
018 YP_009109694.1 LFDIPV--
019 NP_789739.1 --
020 NP_955619.1 TPIVLTSCLVDTKNNWA-----------------------------IIG-
021 YP_224289.1 QISIRTRGYDS--WEGGEANLLITRSVTARLSNTSNVGFAYRIDRVAEYL
022 YP_009182100.1 --
023 NP_705927.1 --
024 YP_009508577.1 NKANIN--
025 YP_004442830.1 QLSIKTKGYNN--WR-GEANLHLERMVTARLSSTSNTGFEIKIDKVVQFI
026 NP_758887.1 --KTTHASVLVGP-------------------------------------
027 YP_009508406.1 --
028 YP_610965.1 EVVVMTHGYQD--WVDGESNLLITKALVGRLTNTSYAHFKYNVEGVADYL
029 YP_001931961.1 --
030 YP_241114.1 --
031 YP_006732334.1 --
032 YP_009041481.1 QVAIITRGYDT--WVAGESNLLITRGLVARVTNSSVSGFRFGIEGVLNYF
033 YP_001036293.1 QVSILTKGYDD--YQNSEANLLITKGMIGRLSNTSNVGFAYTVDGVAEYF
034 YP_605811.1 QISILTKGYEG--FQ-GEANLLITRSCRCRLSNVPNVGFQYNIQNVVEFL
035 NP_955577.1 LFEPES--
036 YP_003208050.1 EVVVATRGYEE--WQGGESNLLITRGLVGRLTNTSHASFNYNVEQVAEHL
037 NP_789740.1 --HKAIGTVLVGP-------------------------------------
038 YP_004442839.1 QISILTKGYNG--FQ-GESNLLVTRSCRCRLSNVPNVGFQYNIQNVVEFL
039 YP_009507791.1 ICNGKICVIPQNN-------------------------------------
040 YP_004581513.1 --
041 NP_043933.1 --
042 YP_009507248.1 RTDHLN--
043 YP_002117531.1 QVSIQTKGYDT--WTNGEANLLVTRSCTARLSNTPNVGFNYKTERVAEYL
044 YP_004347415.1 --
045 NP_056891.1 SGLIKPFVIPNLPVNLWGRDLLSQMKIMMCSPNDIVTAQMLAQGYSPGKG
046 YP_009513242.1 KQTTLP--
047 YP_009352866.1 QVAVNTMEYDT--WIAGESNLLITRGLVARITNSSVSGFRFGIEGVLNYF
048 YP_009165750.1 --
049 NP_040840.1 IIRGTIVVLATS--------------------------------------
050 YP_004222728.1 --
051 YP_009506264.1 QISIRTKGYDS--WQGGEANILITRSITARLSNTPNVGFAYRIEQVAEYL
052 YP_567050.1 --
053 YP_009508571.1 EKTALS--
054 NP_056803.1 NKTALP--
055 NP_955564.1 SGIIHPFVIPTLPFTLWGRDIMKDIKVR----------------------
056 YP_009508411.1 QISVMTKGYED--YTNSEANLLITKSMVGRLSNTSNVGFQYSVSGIADYF
057 YP_009140788.1 QISIQTTGYES--WQNGEANLLITRGMTGRLSNTPNVGFAYRISHVTDYL
058 YP_009508410.1 --
059 NP_040333.1 LHETSK--
060 YP_008992013.1 TLKVQVQ-IQGAEQISTAKIATLHHQLVYRLQNHALDLPTPEHHSDTLMV
061 NP_041734.1 QLSILTRGYEN--WQNGEANLLITRGMTGRLSNTPNVAFAYQIASATDYL
062 NP_056895.1 PFITIPKILVDTFDKWQ-----------------------------ILG-

```
063 YP_009109689.1     LFDIPV--------------------------------------------
064 YP_002519387.1     --------------------------------------------------
065 YP_595725.1        QLTVKVKGYEG--WQ-GEANLHIERLITARLSNTTNVYFKYKVEGVASFI
066 YP_009508551.1     SKTALP--------------------------------------------
067 YP_001497148.1     LFETPV--------------------------------------------
068 NP_041186.1        --------------------------------------------------
069 NP_663784.1        --KRVRATIMTGD-------------------------------------
070 YP_009508443.1     QISILTKGYDD--YHNSEANLLITKGLVGRLSNTSNVGFVYSISGIADYF
071 YP_003987465.1     QISIRTIGYDD--DWNGESNLLITRGMTARLSNTPNVGFAYNISRVADYL
072 NP_861410.1        --------------------------------------------------
073 YP_009345071.1     QISIQTRGYGN--WRNAEANLLVTRGLVGRLSNTSNVGFEYSISNVTDYL
074 YP_009508582.1     NKANIN--------------------------------------------
075 NP_569141.1        --------------------------------------------------
076 YP_002916057.1     --------------------------------------------------
077 NP_777384.2        PFITIPKILVDTFDKWQ-----------------------------ILG-
078 YP_233110.1        QISILTKGYEG--FQ-GEANLLITRSCRCRLSNVPNVGFQYNIQNVVEFL
079 NP_542258.1        --------------------------------------------------
080 YP_009130664.1     QVSILTRGYEA--WR-GEANLLITRGLVGRLSNTPNVGFAYEVQSVVDYL
081 YP_009508566.1     KRADIT--------------------------------------------
082 YP_006607892.1     --------------------------------------------------
083 YP_009506270.1     QISVLTKGYQG--FQ-GEANLLITRSCRCRLTNVPNVGFAYNIQKVVEYL
084 NP_042513.1        --------------------------------------------------
085 YP_006907834.1     --------------------------------------------------
086 YP_004442827.1     QLSIKTKGYIN--WR-GEANLHLERMITARLSSTSNTGFEIKIDKVVQYM
087 YP_009408594.1     HCVAATRAENE--ILDDNDALFSEHDLFG-------HDLSYDPD--QDLF
088 YP_009508561.1     SQANIG--------------------------------------------
089 NP_056907.1        APVTLPSCLVDTNSKWA-----------------------------IIGR
090 YP_002308474.1     --------------------------------------------------
091 YP_007761644.1     --------------------------------------------------
092 NP_040550.1        --------------------------------------------------
093 NP_758808.1        QLAIQTQGYEN--WNSAESNLLISRALIGRLTNDSFTGFQYNISNVAEYL
094 YP_009345075.1     QLVVKVKGYDS--WIGGEANLLITKVFVGRLSNTSNTAFRYAVDDLGEYL
095 NP_068729.1        --------------------------------------------------
096 NP_047255.1        LFEPES--------------------------------------------
097 YP_006273075.1     QISIQTSGYEN--WQNGEANLLITRGMTGRLSNTPNVGFAYRISHVTDYL
098 YP_009508888.1     KETALE--------------------------------------------
099 NP_395469.1        --------------------------------------------------
100 YP_009508546.1     NKTALP--------------------------------------------
101 NP_056762.1        KIGIQTLGYEN----YEGKNLSVAIKTIGRLTTNIQSKYKINVKDIVEQI
102 YP_009508556.1     KQTALP--------------------------------------------
103 NP_569153.1        QISILTKGYGN--WQNGEANLLVTRGIVGRLSNTPNVAFAYQIQNVTDYL
104 NP_043924.1        RLWLVRDQFADKTGVSKARPIVRNEGPRPQQGHSKIVFGGNCRNCGKAGH
105 YP_009243641.1     --------------------------------------------------
106 YP_004300274.1     --------------------------------------------------
107 NP_056848.1        --------------------------------------------------
108 YP_006495799.1     QVSIQTRGYNGG-WTGGDSNMIVTRSLIGRLTNQSTTNFGYQIQGVTDYL
109 YP_009508409.1     --------------------------------------------------
110 NP_687035.1        --KIITGSILIGS-------------------------------------
111 NP_045937.2        GLEWQHATVPFINMVVQGLPPKVTRSLMSGNPDWSTKTIPQIIPLMQHYL
112 NP_612577.1        --------------------------------------------------
113 NP_619548.1        --------------------------------------------------
114 NP_056880.1        SGIIHPFVIPTLPFTLWGRDIMKDIKVR----------------------
115 YP_009121747.1     QIAIQTHGYSD--WQGGESNLLITVGLVGRISNTSYTGFQYNVENVVDHL
116 YP_009113237.2     EISVLTRGYEE--WN-GEANLLITRGLVGRLTNTSNAGFAYSIQNVTDYL
117 YP_009508408.1     QISILTKGYED--YQNSEANLLITKGLIGRLSNTSNIGFAYSIDGIADYF
118 NP_955579.1        LFEPES--------------------------------------------
119 YP_009229919.1     QLAIQTRGYEG--WQ-GESNLIVTTALVGRLTNTSYASFRYNVQNVAEHL
120 YP_004442833.1     QLSIKTKGYNN--WR-GEANLHLERMVTARLSSTSNTGFEIKIDKVIQYM
121 NP_862833.2        --------------------------------------------------
122 NP_040563.1        --KTGIIDVLFSD-------------------------------------
123 YP_443922.1        TMNVTD--------------------------------------------
124 NP_057933.2        LHETSK--------------------------------------------
125 YP_009116631.1     QVSIQTRGYGTG-WEGGDSNMIITRSLVGRLTNTSITSFEYRIDNVTDYL
126 YP_009513249.1     NNSILS--------------------------------------------
127 YP_223871.1        LFLEAP--------------------------------------------
128 NP_127504.1        ALKIQVQ-IQGAPLTKDSIQATLHYQIAWRVQNHAMDLTLPGGEEALFLK
129 NP_044929.1        TESTLS--------------------------------------------
130 NP_057860.1        TPIVLTSCLVDTKNNWA-----------------------------IIG-
131 NP_040973.1        -------VLEDNS-------------------------------------
132 NP_777317.1        QMSILTKGYTG--FQ-GEANLLVTRSCRCRLTSVPNAGFAFNIQRVVEYL
133 NP_054716.1        HTTNLS--------------------------------------------
134 YP_001856242.1     --------------------------------------------------
135 NP_954565.2        SGLIKPFVIPNLPVNLWGRDLLSQMKIMMCSPNDIVTAQMLAQGYSPGKG
```

```
136 NP_569150.1        QISILTKGYQG--FQ-GEANLLLITRSCRCRLTNVPNVGFAYNIQRVVEYL
137 NP_659397.1        ---------------------------------------------------
138 YP_009513211.1     LHEKPV---------------------------------------------
139 YP_233107.1        QMSIQTRGYDS--WTGAEANLLITRSITSRLSNTPNVGFAFQVNKVAEYL
140 YP_009109692.1     LFDIPV---------------------------------------------
141 NP_057849.4        --HKAIGTVLVGP--------------------------------------
142 NP_049560.1        ---------------------------------------------------
143 YP_009506267.1     QISILTKGYDD--YQNSEANLLITKGLVGRLSNTSNVGFAYSISGITDYF
144 NP_039820.1        QICVMTKGYDG--WQ-GEDNLLITRGLTGRLSNTSNVGFAYDVKAMVEHL
145 NP_056790.1        LHEKPV---------------------------------------------
146 YP_002321513.1     QLAIQTRGYESWVGQNAEANIGLSRQVIGRLSNDNVDRYSYNTTAVTDLL
147 NP_955591.1        LHETSK---------------------------------------------
148 YP_001956722.2     KQTMLT---------------------------------------------
149 NP_056902.1        KGRHIKTRMLVAD--------------------------------------
150 NP_056886.1        DGSLERPLLLFPAVAMVRGSILG----------------------------
151 NP_041261.1        ---------------------------------------------------
```

```
001 YP_004442836.1     NSKGVKAIQAQKLSTKKFQGTEWNIKPS-NVVVP-MQPASMITRVNYDSS
002 NP_056728.1        --------------------------------------------------
003 YP_009508537.1     -----KKGKEELKRLFEKYSALWQSWENQ---VGHRKIRPHKIATGTVK-
004 YP_004732983.2     ASHGVQSIPGQPWRDINQEG-SWNLRPSSIQAPT-QVPTGLVSRQSATGN
005 YP_009508407.1     --------------------------------------------------
006 NP_040939.1        -----------PVEVLGRDNMARFGIKIIMANLEEKRIPITKVKLKEGCT
007 YP_003864102.1     --------------------KGIWKFLTTVTDMLAPQKYADPIIWKSDT-
008 YP_009002585.1     TSQGVQAIAGRRYDSRELQNQQWILRPPQVERLP-QAPKNVDTRNLIDGS
009 YP_003284237.1     RSKGVKAINATKHSAKQFQGGEWTLKPS-RVVVP-MQPSSMNTSTKYDGS
010 YP_009268869.1     -----------PVEVLGRDNMAKLDIGIIMANLEEKKIPITQVKLKEGCK
011 YP_009506251.1     ASHGVKAIAGKKVNLQQLRNQQWVLRPPQADVLP-MQPRSVETRNLIDGS
012 NP_955611.1        ---------------------------------TVALHLAIPLKWKPDHT
013 YP_008567619.1     ITHGVNALPGAKYDTTRLQGQQWILKKP-ELIVP-TRPGHINSKDLVDGT
014 NP_149413.1        KTNGIKAIDAKKESTKKIRGGEWNIQPS-TVEVV-MEPRDMMQSQNFDGT
015 YP_004442824.1     NSKGVKAIQAPKLDTKKFRGTDWNIKPS-EVVVP-MEPTRMITRVNYDSS
016 YP_001931967.1     --------------------------------------------------
017 YP_002455786.1     -----------MPGGPLLTGGPPGPDPVIPG-----DHPCSHR-LRTSSR
018 YP_009109694.1     -----TTS--LPDVWLQDFPQAWAETGGL----GRAKCQAPIIIDLKPT-
019 NP_789739.1        ---------------------------------PISPIETVPVKLKPGMD
020 NP_955619.1        -----------RDALQQCQGVLYLPEAKRPPVILPIQAPAVLG-LEHLPR
021 YP_224289.1        RTKGVRAIDATKHDARRFQHGEWNLRPS-TAVVP-VQPTSITSAVNYDRS
022 YP_009182100.1     --------------------------------------------------
023 NP_705927.1        ---------------------------------PISPIETVPVKLKPGMD
024 YP_009508577.1     -----NEEKKQLAKLLDKYDVLWQQWENQ---VGHRKIPPHNIATGTVA-
025 YP_004442830.1     RTNGIKAIEAEKKSVKQFQGKEWNIQPI-KVEVA-MKPNKMTITDEYDNS
026 NP_758887.1        ----------TPVNIVGRNVLKKLGC-TLNF--PVSKVETVKVTLKPGTD
027 YP_009508406.1     --------------------------------------------------
028 YP_610965.1        ASNGIVALAGQRHTTTELQGLDWNLKPK-QSTAP-VQPQEVTYNNRLDGS
029 YP_001931961.1     --------------------------------------------------
030 YP_241114.1        -----------------SRSKRLGVFSLGTSD--SPVTHADPIDWKSEE-
031 YP_006732334.1     --------------------------------------------------
032 YP_009041481.1     NSRGVMAIKAQRRSTVPQRGIAWELRPSSIPRPI-QAPTNAVIRETTRGT
033 YP_001036293.1     ITHGVQALKGMPMTTDDLQGTLWNIKPS-RIIVP-SQPTQAEDRELADGS
034 YP_605811.1        KSKGVKALNATKLSTRRFQGSEWNIRPS-EVVVP-MQPTTMIVRVNYDSS
035 NP_955577.1        -----TQKQEMD-IWLKNFPQAWAETGGM----GTAHCQAPVLIQLKAT-
036 YP_003208050.1     SSHGIVALPGRRHSADAHNGEMWSIRPP-RSNSV-RNPREVQISNRLDGS
037 NP_789740.1        ----------TPVNIIGRNLLTQIGC-TLNF--PISPIETVPVKLKPGMD
038 YP_004442839.1     KSKGVKALNATKISTKKFQGGEWNIKPS-EVVVP-MQPTRMITRVNYDSS
039 YP_009507791.1     ----------LLEPLFGRDNMKLLDIKLVMANIS-TKIPIVKAKLVDPNK
040 YP_004581513.1     --------------------------------------------------
041 NP_043933.1        --------------------------------------------------
042 YP_009507248.1     --------EEEKANLLRLCSRYSDVFYIDGEALTFTNKIKHRIRTTDEVP
043 YP_002117531.1     HSRGVQAINATQHNALKLRNTEWHLQPS-QATAP-MQPQTVQTVGRYDGS
044 YP_004347415.1     --------------------------------------------------
045 NP_056891.1        LGKKENGILHPIPNQGQSNKKGFGNFLTAAIDILAPQQCAEPITWKSDE-
046 YP_009513242.1     -----KEQKDQLEKLFLKYDALWQHWENQ---VGHRRIKPHNIATGTLA-
047 YP_009352866.1     NSKGVMAIKARPRSTLAQRGIAWELRPTSIPRPI-QAPTSAVVRETSRGN
048 YP_009165750.1     --------------------------------------------------
049 NP_040840.1        -----------PVEVLGRDNMRELGIGLIMANLEEKKIPSTRVRLKEGCK
050 YP_004222728.1     ---------------------------------TVALHLAIPLKWKPDHT
051 YP_009506264.1     RSKGVKAINATKKSARQFQGGEWLLRPS-QVVVP-MQPSSLTTSTRYDGT
052 YP_567050.1        ---------------------------------------------EHFPP
053 YP_009508571.1     -----EEFKKQLQTLFLKYDNLWQHWENQ---VCHRKIRPHNIATGDYP-
054 NP_056803.1        -----EEQKQQLKALFTKYDNLWQHWENQ---VGHRKIRPHNIATGDYP-
```

055 NP_955564.1 -----------------LMTDSPDDSQDLMIGAIESNLFADQISWKSDQ-
056 YP_009508411.1 ATRGVQALKGTPFTAEEYQGELWNLKPS-RLTIA-MQPQESEERELPDHS
057 YP_009140788.1 ASRGVQALPGRRYNSDMLRGRNWIIRQPQLQVT--MMPRSVQTTNLLDGS
058 YP_009508410.1 --
059 NP_040333.1 -----GPDVPLGSTWLSDFPQAWAETGGM----GLAVRQAPLIIPLKAT-
060 YP_008992013.1 LAESDQIPTIIQIPRQIPRHELLKLMPLEWISNYEQFHNNTAPVQTSESM
061 NP_041734.1 ASHGVKAIAGKKMNLQHLRNQQWILRPPQTDITP-MQPRSVETRNLVDGS
062 NP_056895.1 -----------RDVLSRLQASISIPEEVRPPMVGVLDAPPSHIGLEHLPV
063 YP_009109689.1 -----TTS--LPDVWLQDFPQAWAETGGL----GRAKCQAPIIIDLKPT-
064 YP_002519387.1 --
065 YP_595725.1 KTKGIKAIEATKKSVKGIRGGEWNILPS-KLEVV-MQPTKVQTTENYDGT
066 YP_009508551.1 -----EDQKQQLKTLFVKYDNLWQHWENQ---VGHRKIRPHNIATGDYP-
067 YP_001497148.1 -----TTN--LLEAWLQDFPQAWAETGGL----GRAKCQAPIIIDLKPT-
068 NP_041186.1 --
069 NP_663784.1 ----------TPINIFGRNILTALGM-SLNL--PVAKIEPIKVTLKPGKD
070 YP_009508443.1 ITHGVQAIIGAPVSTEDIQGTLWNVKPT-TLVVP-VQPTAVDDRELADGS
071 YP_003987465.1 KSKGVKAIDASKEKTTQFKGREWVLKPSQASVTP-IMPTIADAHERYDGS
072 NP_861410.1 --
073 YP_009345071.1 TSHGVRAIPGKKVSESEHLGRSWILREP-EVSII-QQPTIADTRNLPNGS
074 YP_009508582.1 -----NEEKKQLAKLLDKYDILWQQWENQ---VGHRKIPPHNIATGTVA-
075 NP_569141.1 --
076 YP_002916057.1 --
077 NP_777384.2 -----------RDVLSRLQASISIPEEVRPPMVGVLDAPPSHIGLEHLPV
078 YP_233110.1 KSRGVKALNATKLSTRRFQGGEWNIRPS-EVVVP-MQPTTMIVRVNYDSS
079 NP_542258.1 ---------------PTLKCSP-------------CPNTQAYW-VRTSSP
080 YP_009130664.1 VSHGVQAIPGRRRTVQELQGYNWVVRPT-QASIP-RQPTEVDTRTNIDGT
081 YP_009508566.1 -----KEEKGMLYKLLDKYDPLWQQWENQ---VGNRQITPHIIATGTIN-
082 YP_006607892.1 --
083 YP_009506270.1 NSKGVKAIQAQKLSTKKFQGTDWNIKPS-NVVVP-MQPTTMITRVNYDSS
084 NP_042513.1 --
085 YP_006907834.1 --
086 YP_004442827.1 KTNGIQAIEAQKESIKKYQGAEWNIKPI-KVEVT-MKPKSMIVQDEYDNS
087 YP_009408594.1 LEHDLFTPP-----PDWDAWLDTDLSET-AESQG-PNPMMIQVTSINDHE
088 YP_009508561.1 -----KEDKIKLAKLLDKYDDLWQQWDNQ---VGNRKITPHNIATGTYP-
089 NP_056907.1 DILQKCQSVLYLPEDNLCKGTPRLPDGITPPRLLPVTTPNVIG-LEHFPP
090 YP_002308474.1 --CHW-AGTSPP
091 YP_007761644.1 --
092 NP_040550.1 ---------------------------------TVALHLAIPLKWKPDHT
093 NP_758808.1 HSHGVQAIEG--QAHPRTLGNRWILQAP-APPRS-LVPQNVETTTLLDGN
094 YP_009345075.1 ATKGIRAVPGQRYDASQYEAERWQLEPP-AGATV-RVPSRAHVWTDNQGS
095 NP_068729.1 --
096 NP_047255.1 -----TQKQEMD-IWLKNFPQAWAETGGM----GTAHCQAPVLIQLKAT-
097 YP_006273075.1 ASRGVQALPGRRYRSEMLRGRNWIIRQPQIQAA--MMPRNVETRNLIDGT
098 YP_009508888.1 -----GQFKQQLQNILSTFDTLWQHWENQ---VGHRKIPPHNIATGTHP-
099 NP_395469.1 --
100 YP_009508546.1 -----EEQKQRLKTLFAKYDTLWQHWENQ---VGHRRIRPHNIATGDYP-
101 NP_056762.1 SSQGIIMVAPMEIDSSHLDGNEWDLSKFLNHENTSRVPTKALIYQNLQGG
102 YP_009508556.1 -----KEQKDQLEKLFLKYDALWQHWENQ---VGHRRIKPHNIATGTLA-
103 NP_569153.1 VSHGIQALPGRRYSTADIQGQQWFLRPSNIPAVP-MAPTNVDTRNMIDGS
104 NP_043924.1 MARDCWAKGGGQEGKGPRQNTTWKPKSGAIASAPPAESPYADCAKQLADI
105 YP_009243641.1 -------------------------------------MEPIALSWKSNE-
106 YP_004300274.1 --
107 NP_056848.1 --
108 YP_006495799.1 TSHGVSCIPGEQWSVTNRSG-EWRLQQSSIAPPE-QVPTEARLRPGPRGE
109 YP_009508409.1 --
110 NP_687035.1 ----------TPINIIGRNILAQAGM-KLVMGVLSSQIEETKVQLKEGKD
111 NP_045937.2 NLQSRQDAKIKQTPLVLQLAMPAQTMNGNKGYVGSYPTNEPYYSFQQQQR
112 NP_612577.1 --
113 NP_619548.1 --
114 NP_056880.1 -----------------LMTDSPDDSQDLMIGAIESNLFADQISWKSDQ-
115 YP_009121747.1 ATNGITAIPGERRSIEDLEGMSWNLRPA-PLTMI-RIPARVEVNQRLDRS
116 YP_009113237.2 TSHGVKALPGRPRSTEDIRGLNWIVRPPAPRIQP-MQPQHLETHDLLGGG
117 YP_009508408.1 ITNGVQAIKGAPMSTEDIQGTLWNIKPT-TLVVP-VQPTAADDRELADGS
118 NP_955579.1 -----TQKQEMD-IWLKNFPQAWAETGGM----GTAHCQAPVLIQLKAT-
119 YP_009229919.1 ATNGIQAIPGQPRTIDQLKGERWELKTT-KVARS-QAPREVRLQQRSNGN
120 YP_004442833.1 KTNGIQAIEATKQSVKKYQGQDWNIRPA-KVEVA-MKPTAMEVKNEYDNS
121 NP_862833.2 -----------------SRTKRFGVFSLGTSD--SPVTHADPIDWKSGE-
122 NP_040563.1 ----------TPVNLFGRSLLRSIVT-CFTLLVHTEKIEPLPVKVRGP--
123 YP_443922.1 -----DEDNPYTEDWDENMSHQTPSIRCLSIVCGPEGVAAPVILPSHLKP
124 NP_057933.2 -----EPDVSLGSTWLSDFPQAWAETGGM----GLAVRQAPLIIPLKAT-
125 YP_009116631.1 ASNGVACIPGQKWSVANRSG-EWELQPSRIAAPL-AVPTDARLRQNPNGN
126 YP_009513249.1 -----KKGKEELKQLFEKYSALWQSWENQ---VGHRRIRPHKIATGTVK-
127 YP_223871.1 -----IQNVTLLEQWKRGIPKVWAEINPP----GLASTQAPIHVQLLST-

```
128 NP_127504.1       IDAGSGATQCTQVPRQLSKEDLIKILPDSWVTNYEKLREPEEPLRSTEVS
129 NP_044929.1       -----PQGQMRLKKLLDQYQALWQCWENQ---VGHRRIEPHKIATGALK-
130 NP_057860.1       -----------RDALQQCQGVLYLPEAKRPPVILPIQAPAVLG-LEHLPR
131 NP_040973.1       ----------LIQPLLGRDNMIKFNIRLVMAQIS-DKIPVVKVKMKDPNK
132 NP_777317.1       NSTGVRALEAQRVGNQQYQGTDWHIEPS-DIIVP-AQPSQLRTRINYDGT
133 NP_054716.1       -----SEGKKYLKDLFIKYDNLWQKWENQ---VGHRRITPHKIATGTLN-
134 YP_001856242.1    ---------------------------------PISPIETVPVKLKPGMD
135 NP_954565.2       LGKKENGILHPIPNQGQSNKKGFGNFLTAAIDILAPQQCAEPITWKSDE-
136 NP_569150.1       NSKGVKAIQAQKLSTTKYQGTEWNIKPS-NVVVP-MQPTNLITRVNYDNS
137 NP_659397.1       --------------------------------------------------
138 YP_009513211.1    -----PPS--IDPSWLQLFPMVWAEKAGM----GLANQVPPVVVELKSD-
139 YP_233107.1       RSKGIKAIDAMKYSTGQFQHSRWNLRPS-TVVIP-VLPSTLVTSTNYDGS
140 YP_009109692.1    -----TTS--LPDVWLQDFPQAWAETGGL----GRAKCQAPIIIDLKPT-
141 NP_057849.4       ----------TPVNIIGRNLLTQIGC-TLNF--PISPIETVPVKLKPGMD
142 NP_049560.1       --------------------------------------------------
143 YP_009506267.1    VTHGVQAIKGQPVSTEEFQETLWNIKPS-NLVVP-TQPTFAEERELADGS
144 NP_039820.1       QSNGVKAIKGEKWDAKRFHNGQWNIEPS-KVVVP-MQPTEMKAVSNYDGT
145 NP_056790.1       -----PSS--IDPSWLQLFPTVWAERAGM----GLANQVPPVVVELRSG-
146 YP_002321513.1    ASRGIRAIEGARSMPDRSNS--WNLRPSSVQNQQSMIPTAYTARRHINGD
147 NP_955591.1       -----EPDVSLGSTWLSDFPQAWAETGGM----GLAVRQAPLIIPLKAT-
148 YP_001956722.2    -----GSYKEKLQSLFLKYDALWQHWENQ---VGHRRIKPHHIATGTVN-
149 NP_056902.1       ----------IPVTILGRDILQDLGAKLVLAQLS-KEIKFRKIELKEGTM
150 NP_056886.1       ------------RDCLQGLGLRLTNLIGRATVLTVALHLAIPLKWKPDHT
151 NP_041261.1       --------------------------------------------------
```

```
001 YP_004442836.1    RSIRFGDYQASTST--APPRYNNDGDSDDEI-------------------
002 NP_056728.1       --------------------------------------------------
003 YP_009508537.1    --------------------------------------------------
004 YP_004732983.2    ISLRFTGFQDQVQTVETEEESGMTDTGERVTHYALVG-------------
005 YP_009508407.1    --------------------------------------------------
006 NP_040939.1       --------------------------------------------------
007 YP_003864102.1    --------------------------------------------------
008 YP_009002585.1    ISLRFRDYATTSTP--ELPSYNEQDEEVDPDE------------------
009 YP_003284237.1    ISIRFGDYEASTSS--RPPKYNTADDE--ID-------------------
010 YP_009268869.1    --------------------------------------------------
011 YP_009506251.1    ISIRFHDYEVAPST--SGPHYNENDEEVESES------------------
012 NP_955611.1       --------------------------------------------------
013 YP_008567619.1    ISLRFSNYTTQAS--TSKLKYNANDEEIQSDEEILAY-------------
014 NP_149413.1       TSFRFTNFQASASR--PAPAYNDEDEV--EM-------------------
015 YP_004442824.1    RTLKFTDYQASTST--APPKYNEDGDTDD---------------------
016 YP_001931967.1    --------------------------------------------------
017 YP_002455786.1    --------------------------------------------------
018 YP_009109694.1    --------------------------------------------------
019 NP_789739.1       --------------------------------------------------
020 NP_955619.1       --------------------------------------------------
021 YP_224289.1       ISLRFGDYQAASSS--RPPVYNKNDDE--VD-------------------
022 YP_009182100.1    --------------------------------------------------
023 NP_705927.1       --------------------------------------------------
024 YP_009508577.1    --------------------------------------------------
025 YP_004442830.1    KSFRFGHYQASSNQ--PAPEYNSEDEI-IQE-------------------
026 NP_758887.1       --------------------------------------------------
027 YP_009508406.1    --------------------------------------------------
028 YP_610965.1       VTLRFTGYAATRRPTLVEVNEDDLEEFAGMVNF-----------------
029 YP_001931961.1    --------------------------------------------------
030 YP_241114.1       --------------------------------------------------
031 YP_006732334.1    --------------------------------------------------
032 YP_009041481.1    LSTRFSGYDDIPARDSYQSPFDKTEYAEDGDTSLPKR-------------
033 YP_001036293.1    VSMRFGAYKAAPAL--MSS------EEFDSDENQVIN-------------
034 YP_605811.1       RSIRFGDYEASTSS--SAPKYEQDGDEDEAL-------------------
035 NP_955577.1       --------------------------------------------------
036 YP_003208050.1    VSLRFTGYEIA--PAVTEALQS----------------------------
037 NP_789740.1       --------------------------------------------------
038 YP_004442839.1    RSIRFGDYEASTSS--APPTYSTNDDEDEVL-------------------
039 YP_009507791.1    --------------------------------------------------
040 YP_004581513.1    --------------------------------------------------
041 NP_043933.1       --------------------------------------------------
042 YP_009507248.1    --------------------------------------------------
043 YP_002117531.1    LSLQFGNYTHAESS--RPPVYTAHDEERDID-------------------
044 YP_004347415.1    --------------------------------------------------
045 NP_056891.1       --------------------------------------------------
046 YP_009513242.1    --------------------------------------------------
```

```
047 YP_009352866.1   LSTRFFGYEDVPAKEPFKSPFDKTEYEEDGETSQPRR-------------
048 YP_009165750.1   --------------------------------------------------
049 NP_040840.1      --------------------------------------------------
050 YP_004222728.1   --------------------------------------------------
051 YP_009506264.1   LSIRFGDYEASSSS--KPPVYNEHDDE--ID-------------------
052 YP_567050.1      --------------------------------------------------
053 YP_009508571.1   --------------------------------------------------
054 NP_056803.1      --------------------------------------------------
055 NP_955564.1      --------------------------------------------------
056 YP_009508411.1   ISMRFTRYVAAREP--ARIAFNSQDEEVSEDEQHVLA-------------
057 YP_009140788.1   ISARFANYTQAPEP--QQPRYNGHDEEEASDEA-----------------
058 YP_009508410.1   --------------------------------------------------
059 NP_040333.1      --------------------------------------------------
060 YP_008992013.1   FERRPDGTVRMTFKPPPSAPQEPPRLSFTYSS----MITAVQT-------
061 NP_041734.1      ISIRFHDYEAATSA--SRPHYNEEDEEVESET------------------
062 NP_056895.1      --------------------------------------------------
063 YP_009109689.1   --------------------------------------------------
064 YP_002519387.1   --------------------------------------------------
065 YP_595725.1      TSFRFTNYEGASSS--KPVEHNSDD-------------------------
066 YP_009508551.1   --------------------------------------------------
067 YP_001497148.1   --------------------------------------------------
068 NP_041186.1      --------------------------------------------------
069 NP_663784.1      --------------------------------------------------
070 YP_009508443.1   ISVRFSNYKAARQP--AQITYNQDDEQIDEDEAHIIA-------------
071 YP_003987465.1   LGIKFGNYQEASTS--GPPQYNEHDDE--IV-------------------
072 NP_861410.1      --------------------------------------------------
073 YP_009345071.1   VSVRFGGYEAAPET--SRVRFDENDNEIMDDDE-----------------
074 YP_009508582.1   --------------------------------------------------
075 NP_569141.1      --------------------------------------------------
076 YP_002916057.1   --------------------------------------------------
077 NP_777384.2      --------------------------------------------------
078 YP_233110.1      RSIRFGDYEASTSS--SAPRYEQDGDEDEAL-------------------
079 NP_542258.1      --------------------------------------------------
080 YP_009130664.1   ISLRFGNYTSARTSTTRRIAYNLRDEEIQSDEEQIIA-------------
081 YP_009508566.1   --------------------------------------------------
082 YP_006607892.1   --------------------------------------------------
083 YP_009506270.1   RSIRFGDYQASTSS--APPRYNDDGDSGDEI-------------------
084 NP_042513.1      --------------------------------------------------
085 YP_006907834.1   --------------------------------------------------
086 YP_004442827.1   KSFKFGNYQATSNT--PAPEYNSEDEI-IQE-------------------
087 YP_009408594.1   IEEAYHLPTIP---------------------------------------
088 YP_009508561.1   --------------------------------------------------
089 NP_056907.1      --------------------------------------------------
090 YP_002308474.1   --------------------------------------------------
091 YP_007761644.1   --------------------------------------------------
092 NP_040550.1      --------------------------------------------------
093 NP_758808.1      VSIRFSNYHQAPVN-----DTQD-NSHPDIQ-------------------
094 YP_009345075.1   TSIRFTGYNTQAAPAPEYNSRDEEILEE----------------------
095 NP_068729.1      --------------------------------------------------
096 NP_047255.1      --------------------------------------------------
097 YP_006273075.1   ISARSADYTQAPEP--RQPHYNEQDEEVASDEE-----------------
098 YP_009508888.1   --------------------------------------------------
099 NP_395469.1      --------------------------------------------------
100 YP_009508546.1   --------------------------------------------------
101 NP_056762.1      ESLRFSNYKQTRMHDPTENNSDEDEDLKILGEQLN---------------
102 YP_009508556.1   --------------------------------------------------
103 NP_569153.1      ISLRFNSYQPAPDP--TPVAYNQHDEEVPPDED-----------------
104 NP_043924.1      EKRLKDLTTAGGGPKGPNPFHKPX--------------------------
105 YP_009243641.1   --------------------------------------------------
106 YP_004300274.1   --------------------------------------------------
107 NP_056848.1      --------------------------------------------------
108 YP_006495799.1   ISMRFTNFRDQAVRERALDE--DTDSGRPETSGRDET-------------
109 YP_009508409.1   --------------------------------------------------
110 NP_687035.1      --------------------------------------------------
111 NP_045937.2      PAPRAPPGNVPSNTCFFCKQPGHWKADCPNKTRN----------------
112 NP_612577.1      --------------------------------------------------
113 NP_619548.1      --------------------------------------------------
114 NP_056880.1      --------------------------------------------------
115 YP_009121747.1   TSLRFRSYRNNPQPPRFSVDSQDREVQANLS-------------------
116 YP_009113237.2   ISMRFTNYEAAPRE--PERRFNSQDEELASDE------------------
117 YP_009508408.1   ISVRFSKYKAIQEP--TTPVFNDQDEELDPNEQHILA-------------
118 NP_955579.1      --------------------------------------------------
119 YP_009229919.1   ISFRFTNYAGVPRQQPISVNQHDEEQFP----------------------
```

```
120 YP_004442833.1    MTFSFHKYQATSST--PAPQYNSEDEIQLEE-------------------
121 NP_862833.2       --------------------------------------------------
122 NP_040563.1       --------------------------------------------------
123 YP_443922.1       WYQLAPDSSPHVTLAVGNGFEARSLGPMIRRASKLDWVPTATPGIMKAST
124 NP_057933.2       --------------------------------------------------
125 YP_009116631.1    ISLRFTDFRDQRIVEEGETS--EPE-GRPETK-EDES-------------
126 YP_009513249.1    --------------------------------------------------
127 YP_223871.1       --------------------------------------------------
128 NP_127504.1       MSKRHDKSVAISFDHSHYKKLRNTHHFMGMISDDVIVLDDPET-------
129 NP_044929.1       --------------------------------------------------
130 NP_057860.1       --------------------------------------------------
131 NP_040973.1       --------------------------------------------------
132 NP_777317.1       RSIRFADYQASAST--TLPRGSSDTDDHDAT-------------------
133 NP_054716.1       --------------------------------------------------
134 YP_001856242.1    --------------------------------------------------
135 NP_954565.2       --------------------------------------------------
136 NP_569150.1       RSIRFGNYQASTSS--APPKYNEDGDSDDDI-------------------
137 NP_659397.1       --------------------------------------------------
138 YP_009513211.1    --------------------------------------------------
139 YP_233107.1       TTLQFGDYQAASSS--RPPIYN---NEDDEI-------------------
140 YP_009109692.1    --------------------------------------------------
141 NP_057849.4       --------------------------------------------------
142 NP_049560.1       --------------------------------------------------
143 YP_009506267.1    ISIRFSRYKPVPQL--PEVSYNANDEELGDDERHIIA-------------
144 NP_039820.1       TSLRFSNYAAASTS--KPPQYNEKDEEINED-------------------
145 NP_056790.1       --------------------------------------------------
146 YP_002321513.1    TSIRFHDHVANLAQRPQNSEELDEEEAIFSRLPNDQHGSSHFTHTVMMAI
147 NP_955591.1       --------------------------------------------------
148 YP_001956722.2    --------------------------------------------------
149 NP_056902.1       --------------------------------------------------
150 NP_056886.1       --------------------------------------------------
151 NP_041261.1       --------------------------------------------------
```

```
001 YP_004442836.1    ------------------------------------------------QE
002 NP_056728.1       --------------------------------------------------
003 YP_009508537.1    --------------------------------------------------
004 YP_004732983.2    ---------------------------------TFEWLEECPSYQQRRNQ
005 YP_009508407.1    --------------------------------------------------
006 NP_040939.1       --------------------------------------------------
007 YP_003864102.1    --------------------------------------------------
008 YP_009002585.1    ------------------------------------------------EQ
009 YP_003284237.1    ------------------------------------------------SD
010 YP_009268869.1    --------------------------------------------------
011 YP_009506251.1    -----------------------------------------------EAE
012 NP_955611.1       --------------------------------------------------
013 YP_008567619.1    ---------------------------------IHQERQEVTTPEVLGVI
014 NP_149413.1       ------------------------------------------------EA
015 YP_004442824.1    ------------------------------------------------EA
016 YP_001931967.1    --------------------------------------------------
017 YP_002455786.1    --------------------------------------------------
018 YP_009109694.1    --------------------------------------------------
019 NP_789739.1       --------------------------------------------------
020 NP_955619.1       --------------------------------------------------
021 YP_224289.1       ------------------------------------------------SD
022 YP_009182100.1    --------------------------------------------------
023 NP_705927.1       --------------------------------------------------
024 YP_009508577.1    --------------------------------------------------
025 YP_004442830.1    ------------------------------------------------EV
026 NP_758887.1       --------------------------------------------------
027 YP_009508406.1    --------------------------------------------------
028 YP_610965.1       -----------------------------------------------YDD
029 YP_001931961.1    --------------------------------------------------
030 YP_241114.1       --------------------------------------------------
031 YP_006732334.1    --------------------------------------------------
032 YP_009041481.1    -------------------------------------------FNNVTEG
033 YP_001036293.1    ---------------------------------VLT----TTVSS-LLIK
034 YP_605811.1       ------------------------------------------------GD
035 NP_955577.1       --------------------------------------------------
036 YP_003208050.1    ------------------------------------------------DD
037 NP_789740.1       --------------------------------------------------
038 YP_004442839.1    ------------------------------------------------GE
```

```
039 YP_009507791.1   --------------------------------------------------
040 YP_004581513.1   --------------------------------------------------
041 NP_043933.1      --------------------------------------------------
042 YP_009507248.1   --------------------------------------------------
043 YP_002117531.1   ------------------------------------------------EE
044 YP_004347415.1   --------------------------------------------------
045 NP_056891.1      --------------------------------------------------
046 YP_009513242.1   --------------------------------------------------
047 YP_009352866.1   -------------------------------------------FTRVNEE
048 YP_009165750.1   --------------------------------------------------
049 NP_040840.1      --------------------------------------------------
050 YP_004222728.1   --------------------------------------------------
051 YP_009506264.1   ------------------------------------------------DE
052 YP_567050.1      --------------------------------------------------
053 YP_009508571.1   --------------------------------------------------
054 NP_056803.1      --------------------------------------------------
055 NP_955564.1      --------------------------------------------------
056 YP_009508411.1   ---------------------------------VLH----TSEQF-LKVQ
057 YP_009140788.1   ----------------------------------------------ELAA
058 YP_009508410.1   --------------------------------------------------
059 NP_040333.1      --------------------------------------------------
060 YP_008992013.1   --------------------------------------------------
061 NP_041734.1      -----------------------------------------------ESE
062 NP_056895.1      --------------------------------------------------
063 YP_009109689.1   --------------------------------------------------
064 YP_002519387.1   --------------------------------------------------
065 YP_595725.1      ------------------------------------------------EA
066 YP_009508551.1   --------------------------------------------------
067 YP_001497148.1   --------------------------------------------------
068 NP_041186.1      --------------------------------------------------
069 NP_663784.1      --------------------------------------------------
070 YP_009508443.1   ---------------------------------YLH----MEEPC-LKVR
071 YP_003987465.1   ------------------------------------------------ED
072 NP_861410.1      --------------------------------------------------
073 YP_009345071.1   ------------------------------------------------SQ
074 YP_009508582.1   --------------------------------------------------
075 NP_569141.1      --------------------------------------------------
076 YP_002916057.1   --------------------------------------------------
077 NP_777384.2      --------------------------------------------------
078 YP_233110.1      ------------------------------------------------GD
079 NP_542258.1      --------------------------------------------------
080 YP_009130664.1   ---------------------------------VFKLDCYEAQPAIWDTL
081 YP_009508566.1   --------------------------------------------------
082 YP_006607892.1   --------------------------------------------------
083 YP_009506270.1   ------------------------------------------------QT
084 NP_042513.1      --------------------------------------------------
085 YP_006907834.1   --------------------------------------------------
086 YP_004442827.1   ------------------------------------------------EV
087 YP_009408594.1   ------------------------------------------------DD
088 YP_009508561.1   --------------------------------------------------
089 NP_056907.1      --------------------------------------------------
090 YP_002308474.1   --------------------------------------------------
091 YP_007761644.1   --------------------------------------------------
092 NP_040550.1      --------------------------------------------------
093 NP_758808.1      ------------------------------------------------ED
094 YP_009345075.1   ------------------------------------------------EE
095 NP_068729.1      --------------------------------------------------
096 NP_047255.1      --------------------------------------------------
097 YP_006273075.1   ----------------------------------------------ELAE
098 YP_009508888.1   --------------------------------------------------
099 NP_395469.1      --------------------------------------------------
100 YP_009508546.1   --------------------------------------------------
101 NP_056762.1      --------------------------------------------------
102 YP_009508556.1   --------------------------------------------------
103 NP_569153.1      -----------------------------------------------EEQ
104 NP_043924.1      --------------------------------------------------
105 YP_009243641.1   --------------------------------------------------
106 YP_004300274.1   --------------------------------------------------
107 NP_056848.1      --------------------------------------------------
108 YP_006495799.1   ---------------------------------TPYVGALRFDFKGRSKP
109 YP_009508409.1   --------------------------------------------------
110 NP_687035.1      --------------------------------------------------
111 NP_045937.2      --------------------------------------------------
```

```
112 NP_612577.1        --------------------------------------------------
113 NP_619548.1        --------------------------------------------------
114 NP_056880.1        --------------------------------------------------
115 YP_009121747.1     ------------------------------------------------ED
116 YP_009113237.2     -------------------------------------------------E
117 YP_009508408.1     ---------------------------------YLS----EASDMPLKIR
118 NP_955579.1        --------------------------------------------------
119 YP_009229919.1     ------------------------------------------------ED
120 YP_004442833.1     ------------------------------------------------EV
121 NP_862833.2        --------------------------------------------------
122 NP_040563.1        --------------------------------------------------
123 YP_443922.1        EQMWRIVLV-----------------------------------------
124 NP_057933.2        --------------------------------------------------
125 YP_009116631.1     ---------------------------------THYV----LMFKHSSPR
126 YP_009513249.1     --------------------------------------------------
127 YP_223871.1        --------------------------------------------------
128 NP_127504.1        --------------------------------------------------
129 NP_044929.1        --------------------------------------------------
130 NP_057860.1        --------------------------------------------------
131 NP_040973.1        --------------------------------------------------
132 NP_777317.1        ------------------------------------------------AV
133 NP_054716.1        --------------------------------------------------
134 YP_001856242.1     --------------------------------------------------
135 NP_954565.2        --------------------------------------------------
136 NP_569150.1        ------------------------------------------------QA
137 NP_659397.1        --------------------------------------------------
138 YP_009513211.1     --------------------------------------------------
139 YP_233107.1        ------------------------------------------------DE
140 YP_009109692.1     --------------------------------------------------
141 NP_057849.4        --------------------------------------------------
142 NP_049560.1        --------------------------------------------------
143 YP_009506267.1     ---------------------------------YLS----TPNLV---VR
144 NP_039820.1        ------------------------------------------------EQ
145 NP_056790.1        --------------------------------------------------
146 YP_002321513.1     QPSWEICQGIVPEDTFYTNGVFHGDMRNIDPYFNEYQVDTMPWIEETNEQ
147 NP_955591.1        --------------------------------------------------
148 YP_001956722.2     --------------------------------------------------
149 NP_056902.1        --------------------------------------------------
150 NP_056886.1        --------------------------------------------------
151 NP_041261.1        --------------------------------------------------
```

```
001 YP_004442836.1     TIN---MLYIEDDLED----------------------------------
002 NP_056728.1        --------------------------------------------------
003 YP_009508537.1     --------------------------------------------------
004 YP_004732983.2     ETEENGWVKHVEGDKGFNFKVRMTPPAWSHDPQPI---------------
005 YP_009508407.1     --------------------------------------------------
006 NP_040939.1        --------------------------------------------------
007 YP_003864102.1     --------------------------------------------------
008 YP_009002585.1     LMEHVVAAFVEEEPKEIWDT------------------------------
009 YP_003284237.1     EEHTIAMIT-EEIDE-----------------------------------
010 YP_009268869.1     --------------------------------------------------
011 YP_009506251.1     IREHTVAVWIIGEEEYPDDNG-----------------------------
012 NP_955611.1        --------------------------------------------------
013 YP_008567619.1     RLTPEARLPVRTSPSAAGYNLFGTERLVIQPGERQ---------------
014 NP_149413.1        HMALCE--ELDEEL------------------------------------
015 YP_004442824.1     EVHHVNMLFFE--AED----------------------------------
016 YP_001931967.1     --------------------------------------------------
017 YP_002455786.1     --------------------------------------------------
018 YP_009109694.1     --------------------------------------------------
019 NP_789739.1        --------------------------------------------------
020 NP_955619.1        --------------------------------------------------
021 YP_224289.1        EETFQINMVLLGDDMD----------------------------------
022 YP_009182100.1     -----------------MSGFKLSSPSDEDLNRWLTDELDRVQNKIIGKV
023 NP_705927.1        --------------------------------------------------
024 YP_009508577.1     --------------------------------------------------
025 YP_004442830.1     RMTLCESEDEDEGI------------------------------------
026 NP_758887.1        --------------------------------------------------
027 YP_009508406.1     --------------------------------------------------
028 YP_610965.1        SSPLLCTEILEEE-------------------------------------
029 YP_001931961.1     --------------------------------------------------
030 YP_241114.1        --------------------------------------------------
```

```
031 YP_006732334.1    --------------------------------------------------
032 YP_009041481.1    TSTNFALVILASCNPDFPEDDQLADGNFYT--------------------
033 YP_001036293.1    LMHRDAIIPQRITDGSVGYDLAACEDVVIQPRRPT---------------
034 YP_605811.1       IHQVNMITIIEDDAED----------------------------------
035 NP_955577.1       --------------------------------------------------
036 YP_003208050.1    ESELAAMAIQESG-------------------------------------
037 NP_789740.1       --------------------------------------------------
038 YP_004442839.1    IHQVNMITFLEDDDEF----------------------------------
039 YP_009507791.1    --------------------------------------------------
040 YP_004581513.1    --------------------------------------------------
041 NP_043933.1       --------------------------------------------------
042 YP_009507248.1    --------------------------------------------------
043 YP_002117531.1    SIAYLYSSDGEADDTDLEFLT-----------------------------
044 YP_004347415.1    --------------------------------------------------
045 NP_056891.1       --------------------------------------------------
046 YP_009513242.1    --------------------------------------------------
047 YP_009352866.1    TKDNYALVIIATESPDFPEEG-LAEGDYYT--------------------
048 YP_009165750.1    --------------------------------------------------
049 NP_040840.1       --------------------------------------------------
050 YP_004222728.1    --------------------------------------------------
051 YP_009506264.1    HLVAFLDVEEEEEDP-----------------------------------
052 YP_567050.1       --------------------------------------------------
053 YP_009508571.1    --------------------------------------------------
054 NP_056803.1       --------------------------------------------------
055 NP_955564.1       --------------------------------------------------
056 YP_009508411.1    VLHPDARIPQRMTAEAAGYDLTVIESAQLQPGEQK---------------
057 YP_009140788.1    TQHHVVAMFRLSGYTEVEEFKPKFKEQYERYQREQ---------------
058 YP_009508410.1    --------------------------------------------------
059 NP_040333.1       --------------------------------------------------
060 YP_008992013.1    --------------------------------------------------
061 NP_041734.1       IREHTIAVWIG-EEEIPDQTG-----------------------------
062 NP_056895.1       --------------------------------------------------
063 YP_009109689.1    --------------------------------------------------
064 YP_002519387.1    --------------------------------------------------
065 YP_595725.1       YMALFE--EEEEE-------------------------------------
066 YP_009508551.1    --------------------------------------------------
067 YP_001497148.1    --------------------------------------------------
068 NP_041186.1       --------------------------------------------------
069 NP_663784.1       --------------------------------------------------
070 YP_009508443.1    LLNEDAIIPQRLSHQAVGYDIAVVQAAEVPALGQT---------------
071 YP_003987465.1    VVLAIIDDLPEDDEEDYFQQILNDYQPDSQVALDYPVLIPTED----PFA
072 NP_861410.1       --------------------------------------------------
073 YP_009345071.1    IAEEVHVMIINEDDEEWEELN-----------------------------
074 YP_009508582.1    --------------------------------------------------
075 NP_569141.1       --------------------------------------------------
076 YP_002916057.1    --------------------------------------------------
077 NP_777384.2       --------------------------------------------------
078 YP_233110.1       IHQVNMITIIEDDAED----------------------------------
079 NP_542258.1       --------------------------------------------------
080 YP_009130664.1    GE-PSGKFGYYVRYDTHDDNLHIPLEHIIATG------------------
081 YP_009508566.1    --------------------------------------------------
082 YP_006607892.1    --------------------------------------------------
083 YP_009506270.1    TVEHVNMLYILDEAED----------------------------------
084 NP_042513.1       --------------------------------------------------
085 YP_006907834.1    --------------------------------------------------
086 YP_004442827.1    RMALCDSDSDSETDD-----------------------------------
087 YP_009408594.1    ES--TGTEVLDIN-------------------------------------
088 YP_009508561.1    --------------------------------------------------
089 NP_056907.1       --------------------------------------------------
090 YP_002308474.1    --------------------------------------------------
091 YP_007761644.1    --------------------------------------------------
092 NP_040550.1       --------------------------------------------------
093 NP_758808.1       ENQFIGFLS---DLGEE---------------------------------
094 YP_009345075.1    SEIETARMVLFVN-------------------------------------
095 NP_068729.1       --------------------------------------------------
096 NP_047255.1       --------------------------------------------------
097 YP_006273075.1    TQQHIVAMLRFPDYVEIQEFKPKYKEQYDKYKREQ---------------
098 YP_009508888.1    --------------------------------------------------
099 NP_395469.1       --------------------------------------------------
100 YP_009508546.1    --------------------------------------------------
101 NP_056762.1       --------------------------------------------------
102 YP_009508556.1    --------------------------------------------------
103 NP_569153.1       IRNHTIALWREDD--EVWDT------------------------------
```

```
104 NP_043924.1        --------------------------------------------------
105 YP_009243641.1     --------------------------------------------------
106 YP_004300274.1     --------------------------------------------------
107 NP_056848.1        --------------------------------------------------
108 YP_006495799.1     REEECFEVAAPDVLQSWMQQLSRSPPRQNR--------------------
109 YP_009508409.1     --------------------------------------------------
110 NP_687035.1        --------------------------------------------------
111 NP_045937.2        --------------------------------------------------
112 NP_612577.1        --------------------------------------------------
113 NP_619548.1        --------------------------------------------------
114 NP_056880.1        --------------------------------------------------
115 YP_009121747.1     EEQFVGICIQEREIHNP---------------------------------
116 YP_009113237.2     EVETILVLIAEEPEKEVWDT------------------------------
117 YP_009508408.1     LLNADAVLPQRMSYGAVGYDIAITTSEDIPPYRQV---------------
118 NP_955579.1        --------------------------------------------------
119 YP_009229919.1     EREFVGAILEEVEQPQENKPWWHDYDPDLPKHWGKQCDGCLMN-------
120 YP_004442833.1     RMAICSDDESEEPS------------------------------------
121 NP_862833.2        --------------------------------------------------
122 NP_040563.1        --------------------------------------------------
123 YP_443922.1        --------------------------------------------------
124 NP_057933.2        --------------------------------------------------
125 YP_009116631.1     WDTLGQPSGKYDYMVRYDAPEPTTWPTTNR--------------------
126 YP_009513249.1     --------------------------------------------------
127 YP_223871.1        --------------------------------------------------
128 NP_127504.1        --------------------------------------------------
129 NP_044929.1        --------------------------------------------------
130 NP_057860.1        --------------------------------------------------
131 NP_040973.1        --------------------------------------------------
132 NP_777317.1        QTEYINMLYTIE-GDD----------------------------------
133 NP_054716.1        --------------------------------------------------
134 YP_001856242.1     --------------------------------------------------
135 NP_954565.2        --------------------------------------------------
136 NP_569150.1        TIEHVNMLYIEDTSDT----------------------------------
137 NP_659397.1        --------------------------------------------------
138 YP_009513211.1     --------------------------------------------------
139 YP_233107.1        EQHIVAVITIIDDQED----------------------------------
140 YP_009109692.1     --------------------------------------------------
141 NP_057849.4        --------------------------------------------------
142 NP_049560.1        --------------------------------------------------
143 YP_009506267.1     LVNENAILPQRMSHGAVGYDIAITEAAEILPGQQK---------------
144 NP_039820.1        EINHSLNLILNDEESTDEDEEYYQYQRYAWSQVGDSTFYYDTDGVWEEID
145 NP_056790.1        --------------------------------------------------
146 YP_002321513.1     LADQAEVIIEEMDRDQHFIVITDGSNYPPGYFDFPDSDTEEPDDY-----
147 NP_955591.1        --------------------------------------------------
148 YP_001956722.2     --------------------------------------------------
149 NP_056902.1        --------------------------------------------------
150 NP_056886.1        --------------------------------------------------
151 NP_041261.1        --------------------------------------------------
```

```
001 YP_004442836.1     ----------------------------------------------DYP-
002 NP_056728.1        --------------------------------------------------
003 YP_009508537.1     --------------------------------------------------
004 YP_004732983.2     ------------------------IATGWGDDFNNPPPPPPPKTEEEEIL
005 YP_009508407.1     --------------------------------------------------
006 NP_040939.1        --------------------------------------------------
007 YP_003864102.1     --------------------------------------------------
008 YP_009002585.1     ---------------------LGQPSGKFDFLVKYSAPASSKIRIEDIVP
009 YP_003284237.1     -------------------------WLILAEELG----------YDLTEE
010 YP_009268869.1     --------------------------------------------------
011 YP_009506251.1     -----------------RIKVWEESSNGNGRFFRYYTPPSVSN--EPIIA
012 NP_955611.1        --------------------------------------------------
013 YP_008567619.1     -----------------LQRTGIAIRVPEGHYGRIAARSSAAWQLGIVVG
014 NP_149413.1        ------------------------------------------------TD
015 YP_004442824.1     ----------------------------------------------DYPS
016 YP_001931967.1     --------------------------------------------------
017 YP_002455786.1     --------------------------------------------------
018 YP_009109694.1     --------------------------------------------------
019 NP_789739.1        --------------------------------------------------
020 NP_955619.1        --------------------------------------------------
021 YP_224289.1        -------------------------YPMFRELEK---------LYITSES
022 YP_009182100.1     KDVDPDHVAMAEPGENHLRNGKVLIHQMNRMFATINTMFEKQLWFEQRQK
```

```
023 NP_705927.1       --------------------------------------------------
024 YP_009508577.1    --------------------------------------------------
025 YP_004442830.1    ------------------------------------------------SA
026 NP_758887.1       --------------------------------------------------
027 YP_009508406.1    --------------------------------------------------
028 YP_610965.1       ----------------------------DDEDLWVPVPPTCRASPYSDAE
029 YP_001931961.1    --------------------------------------------------
030 YP_241114.1       --------------------------------------------------
031 YP_006732334.1    --------------------------------------------------
032 YP_009041481.1    -----------------------------GQVLTD-WEDDY--LQDWLTD
033 YP_001036293.1    -----------------LVKTGIAIEVPAGTYAQLYLKSSFALKTGLQIT
034 YP_605811.1       ----------------------------------------------DYPR
035 NP_955577.1       --------------------------------------------------
036 YP_003208050.1    ----------------------------EDED-----------------D
037 NP_789740.1       --------------------------------------------------
038 YP_004442839.1    ----------------------------------------------DYPQ
039 YP_009507791.1    --------------------------------------------------
040 YP_004581513.1    --------------------------------------------------
041 NP_043933.1       --------------------------------------------------
042 YP_009507248.1    --------------------------------------------------
043 YP_002117531.1    --------------------QLDQVIPPVENVEGEEVSLFSLLDELDAEA
044 YP_004347415.1    --------------------------------------------------
045 NP_056891.1       --------------------------------------------------
046 YP_009513242.1    --------------------------------------------------
047 YP_009352866.1    -----------------------------GQVLSDNWEDDY--LEDWLEE
048 YP_009165750.1    --------------------------------------------------
049 NP_040840.1       --------------------------------------------------
050 YP_004222728.1    --------------------------------------------------
051 YP_009506264.1    -------------------------WLALERELG----------YDADYE
052 YP_567050.1       --------------------------------------------------
053 YP_009508571.1    --------------------------------------------------
054 NP_056803.1       --------------------------------------------------
055 NP_955564.1       --------------------------------------------------
056 YP_009508411.1    -----------------LFRTGVAIEVPTGTYAHLFPRSGIALKKGINIG
057 YP_009140788.1    -----------------QWDTLGQPSGKYDYYVQYTAPPAATIPLTEIQP
058 YP_009508410.1    --------------------------------------------------
059 NP_040333.1       --------------------------------------------------
060 YP_008992013.1    ---------------------------------AQENLPITGFTSNGFPV
061 NP_041734.1       -----------------RKKVWEESSNGNGRFFRYYTPPPTFE--GQIIA
062 NP_056895.1       --------------------------------------------------
063 YP_009109689.1    --------------------------------------------------
064 YP_002519387.1    --------------------------------------------------
065 YP_595725.1       ------------------------------------------------DD
066 YP_009508551.1    --------------------------------------------------
067 YP_001497148.1    --------------------------------------------------
068 NP_041186.1       --------------------------------------------------
069 NP_663784.1       --------------------------------------------------
070 YP_009508443.1    -----------------MFSTGIAVEVPNGTYAHLFIKSGVAKRKGLILN
071 YP_003987465.1    SSYAPLTPPALTPLPASGDNTPDGIRPVLQRIVVGEKDFTDFNTLDFTLR
072 NP_861410.1       --------------------------------------------------
073 YP_009345071.1    ----------------------RQMMADFAEEEQKHTEPIIAEEDIYLLP
074 YP_009508582.1    --------------------------------------------------
075 NP_569141.1       --------------------------------------------------
076 YP_002916057.1    --------------------------------------------------
077 NP_777384.2       --------------------------------------------------
078 YP_233110.1       ----------------------------------------------DYPR
079 NP_542258.1       --------------------------------------------------
080 YP_009130664.1    --------------------------WDDDDDDCTTSSSDEASYLQHLS-
081 YP_009508566.1    --------------------------------------------------
082 YP_006607892.1    --------------------------------------------------
083 YP_009506270.1    ----------------------------------------------EYPT
084 NP_042513.1       --------------------------------------------------
085 YP_006907834.1    --------------------------------------------------
086 YP_004442827.1    ----------------------------------------------PQSA
087 YP_009408594.1    --------------------------------------------------
088 YP_009508561.1    --------------------------------------------------
089 NP_056907.1       --------------------------------------------------
090 YP_002308474.1    --------------------------------------------------
091 YP_007761644.1    --------------------------------------------------
092 NP_040550.1       --------------------------------------------------
093 NP_758808.1       ----------------------------YELEYPSFTPVHADEFIFIIIN
094 YP_009345075.1    ----------------------------ATNKGTEIRRIERPTVPAVYPA
095 NP_068729.1       --------------------------------------------------
```

```
096 NP_047255.1          --------------------------------------------------
097 YP_006273075.1       -----------------GWDTLGEPSGKYDYYVRYTAPP-ATTPIEDIQP
098 YP_009508888.1       --------------------------------------------------
099 NP_395469.1          --------------------------------------------------
100 YP_009508546.1       --------------------------------------------------
101 NP_056762.1          ----------------------------IKMARFYTMQTPEEELREVIQQ
102 YP_009508556.1       --------------------------------------------------
103 NP_569153.1          ---------------------LGEPSGKFDFYVRYTRPAHALQDPAHIVA
104 NP_043924.1          ---------------------------GVAALAAPLCSLKGRPHVSVEIE
105 YP_009243641.1       --------------------------------------------------
106 YP_004300274.1       --------------------------------------------------
107 NP_056848.1          --------------------------------------------------
108 YP_006495799.1       ---------------------------YQGPEWDTLGEPSG-KYDYKVRY
109 YP_009508409.1       --------------------------------------------------
110 NP_687035.1          --------------------------------------------------
111 NP_045937.2          --------------------------------------------LRNMGN
112 NP_612577.1          --------------------------------------------------
113 NP_619548.1          --------------------------------------------------
114 NP_056880.1          --------------------------------------------------
115 YP_009121747.1       ----------------------------YDICFCSVCLSEAKRVEEEPLN
116 YP_009113237.2       ---------------------LGEPSGKFDYFVKYSAPEWAKNP-VKIEP
117 YP_009508408.1       -----------------KLSTGLAIEVPYGTYAQLYIKSGVAHRTGLMLN
118 NP_955579.1          --------------------------------------------------
119 YP_009229919.1       -----------------PIIGTRWKYTEDDYDLCASCMIKYKAQQGSYAA
120 YP_004442833.1       -----------------------------------------------TSA
121 NP_862833.2          --------------------------------------------------
122 NP_040563.1          --------------------------------------------------
123 YP_443922.1          --------------------------------------------------
124 NP_057933.2          --------------------------------------------------
125 YP_009116631.1       ---------------------------GWDDDPPKPPSPKG-SYEVSLRG
126 YP_009513249.1       --------------------------------------------------
127 YP_223871.1          --------------------------------------------------
128 NP_127504.1          ---------------------------------FSKTLPSLMQTHDWIHH
129 NP_044929.1          --------------------------------------------------
130 NP_057860.1          --------------------------------------------------
131 NP_040973.1          --------------------------------------------------
132 NP_777317.1          ----------------------------------------------TESA
133 NP_054716.1          --------------------------------------------------
134 YP_001856242.1       --------------------------------------------------
135 NP_954565.2          --------------------------------------------------
136 NP_569150.1          ----------------------------------------------DYPV
137 NP_659397.1          --------------------------------------------------
138 YP_009513211.1       --------------------------------------------------
139 YP_233107.1          ----------------------------------------------DYPA
140 YP_009109692.1       --------------------------------------------------
141 NP_057849.4          --------------------------------------------------
142 NP_049560.1          --------------------------------------------------
143 YP_009506267.1       -----------------LLPTGISLQVPNGFYAHLFIKSGVALRKGLILN
144 NP_039820.1          RCNDLPEYVPSETSTPTIDESEAIIDEFLEHAYEQRCDSDESLQSGDPRK
145 NP_056790.1          --------------------------------------------------
146 YP_002321513.1       -----------------AEFLQDLAEAPSQDRSSLYGSEDRSPEVSTKLD
147 NP_955591.1          --------------------------------------------------
148 YP_001956722.2       --------------------------------------------------
149 NP_056902.1          --------------------------------------------------
150 NP_056886.1          --------------------------------------------------
151 NP_041261.1          --------------------------------------------------
```

```
001 YP_004442836.1       -AAEEEQVLLEDMVGEED----------------IISKFLEELELSD---
002 NP_056728.1          --------------------------------------------------
003 YP_009508537.1       --------------------------------------------------
004 YP_004732983.2       ELYPVRRRPDPVQIARKEKAAVFSQAVNTIFEHEGKDVSRMKPSGEAPDS
005 YP_009508407.1       --------------------------------------------------
006 NP_040939.1          --------------------------------------------------
007 YP_003864102.1       --------------------------------------------------
008 YP_009002585.1       TGWGDEWEQPEQQQSPQQQQLLSCSQE------ESDDEDDWVSRFQFLAQ
009 YP_003284237.1       VEDVPPEQSSRSMVGEEK----------------IISQFLEKL-------
010 YP_009268869.1       --------------------------------------------------
011 YP_009506251.1       TGWDDDEDDDYDYDPRWDTNLESEGEEPDEPLRSEEDEEEEVQDEPVWHQ
012 NP_955611.1          --------------------------------------------------
013 YP_008567619.1       AGVIDADFQGEIKVLIFNLSNSPVEIEAGDAVAQLVLERISTPSLSVWRV
014 NP_149413.1          QQWIDRYLSEYSAQQKVVG---------------EIDEE-----------
```

```
015 YP_004442824.1    LAALEKIFSPEGMVGEEE----------------TINTFLQNLNLDT---
016 YP_001931967.1    --------------------------------------------------
017 YP_002455786.1    --------------------------------------------------
018 YP_009109694.1    --------------------------------------------------
019 NP_789739.1       --------------------------------------------------
020 NP_955619.1       --------------------------------------------------
021 YP_224289.1       MVGEDDEEEKSEDLDDEE----------------AHADFQKFLNYSDEVN
022 YP_009182100.1    VLFQNLWDFLNSYDENQKAQQKALKDSQDNIALATRNIARMLEQVKGIEE
023 NP_705927.1       --------------------------------------------------
024 YP_009508577.1    --------------------------------------------------
025 YP_004442830.1    LQEIENFLSALPP-EGGGS---------------NFSAARCSEN------
026 NP_758887.1       --------------------------------------------------
027 YP_009508406.1    --------------------------------------------------
028 YP_610965.1       EEFLQNLFSAGFTPATEEQDMEYPVSIPIPLSPIYDTYPEDVVHTDHGVL
029 YP_001931961.1    --------------------------------------------------
030 YP_241114.1       --------------------------------------------------
031 YP_006732334.1    --------------------------------------------------
032 YP_009041481.1    EPAPQPTAAPLPTLSLDEAEDIMNDFLDRYFSDEKDSTTVSSFSVEREVE
033 YP_001036293.1    GGVVDPDYRGELQIILINNTFTCVPILKGTFVAQIVLHQVATPAVQETTT
034 YP_605811.1       LSALERIIAPESMVGEED----------------TIAEFLGNLSLDSSTD
035 NP_955577.1       --------------------------------------------------
036 YP_003208050.1    DPFQDFLYMASMPRTAYES------------QPIWD--------------
037 NP_789740.1       --------------------------------------------------
038 YP_004442839.1    LAAFEQIFALESGVGEEDD---------------TIIEFLDNLRLSD---
039 YP_009507791.1    --------------------------------------------------
040 YP_004581513.1    --------------------------------------------------
041 NP_043933.1       --------------------------------------------------
042 YP_009507248.1    --------------------------------------------------
043 YP_002117531.1    AAAAAAAAVTEEPAVADP----------------IIEEFLQALDSAEQEL
044 YP_004347415.1    --------------------------------------------------
045 NP_056891.1       --------------------------------------------------
046 YP_009513242.1    --------------------------------------------------
047 YP_009352866.1    S-------QPQINISFSEEEDIISTFLG---NDEKESCYQSSFFGDRVEE
048 YP_009165750.1    --------------------------------------------------
049 NP_040840.1       --------------------------------------------------
050 YP_004222728.1    --------------------------------------------------
051 YP_009506264.1    VADT----TSEALVGDEE----------------VIQTFLSQVQD-----
052 YP_567050.1       --------------------------------------------------
053 YP_009508571.1    --------------------------------------------------
054 NP_056803.1       --------------------------------------------------
055 NP_955564.1       --------------------------------------------------
056 YP_009508411.1    AGVIDPDYTGEVKVLIQNISTQPCDIYAGEACAQIILEQHLTPEVQEVRA
057 YP_009140788.1    SGWDDQ--------PQWD-----------------DEPQ---ATTIRFEE
058 YP_009508410.1    --------------------------------------------------
059 NP_040333.1       --------------------------------------------------
060 YP_008992013.1    YPAKQNGHFLWDVPGSGHC--DPDCPCW------DDWEEDNDYVT-----
061 NP_041734.1       TGWGSD-DDNEKTPPKWD-----------------ESPDEEGPTEPIWDQ
062 NP_056895.1       --------------------------------------------------
063 YP_009109689.1    --------------------------------------------------
064 YP_002519387.1    --------------------------------------------------
065 YP_595725.1       ITFLNRILSKYSTQQKVVG---------------EEEFS-----------
066 YP_009508551.1    --------------------------------------------------
067 YP_001497148.1    --------------------------------------------------
068 NP_041186.1       --------------------------------------------------
069 NP_663784.1       --------------------------------------------------
070 YP_009508443.1    AGVIDPDYTGEVMLLVCNPTATPQFIQKGEFIAQIVLQQVLTPPVQDVAI
071 YP_003987465.1    GLDVELQTTDDFTAEEEE----------------LIGSFLNQLEEEENLY
072 NP_861410.1       --------------------------------------------------
073 YP_009345071.1    GMEDLLNISTDADLSNGNNLTSTVNGFIIVSVYHNGENMKILKVRNDTKE
074 YP_009508582.1    --------------------------------------------------
075 NP_569141.1       --------------------------------------------------
076 YP_002916057.1    --------------------------------------------------
077 NP_777384.2       --------------------------------------------------
078 YP_233110.1       LSALERIIAPESMVGEED----------------TIAEFLSNLSLDSSTD
079 NP_542258.1       --------------------------------------------------
080 YP_009130664.1    -----------------NLGGTPVPIEQDLPYPVPISDMINP--------
081 YP_009508566.1    --------------------------------------------------
082 YP_006607892.1    --------------------------------------------------
083 YP_009506270.1    LAAEEESFPLENMVGEEA----------------IISQFLESLNLSN---
084 NP_042513.1       --------------------------------------------------
085 YP_006907834.1    --------------------------------------------------
086 YP_004442827.1    IYQIEQYLATLSPQEEGGD---------------CVSAARSRNTECA---
087 YP_009408594.1    --------------------------------------------------
```

```
088 YP_009508561.1      --------------------------------------------------
089 NP_056907.1         --------------------------------------------------
090 YP_002308474.1      --------------------------------------------------
091 YP_007761644.1      --------------------------------------------------
092 NP_040550.1         --------------------------------------------------
093 NP_758808.1         GEEIPD-----------------------DFVSSFCSNFSPPPIPEPEPT
094 YP_009345075.1      NPXVHKQFEENMEERQSQGERWGTLGDDDSFLVKFSAPPTIPIEDIVPSG
095 NP_068729.1         --------------------------------------------------
096 NP_047255.1         --------------------------------------------------
097 YP_006273075.1      TGWEEE--------PKWD-----------------DSPDEKSLATIRFEE
098 YP_009508888.1      --------------------------------------------------
099 NP_395469.1         --------------------------------------------------
100 YP_009508546.1      --------------------------------------------------
101 NP_056762.1         LEREKQAMIAKLEAKMKESSKMAIVEDNFNPNNEYLEDTYSEYEDLEFEK
102 YP_009508556.1      --------------------------------------------------
103 NP_569153.1         TGWDDLDNDP-STSSPSNNILTYLTPS------SSSDEDDDMSYLQYLAQ
104 NP_043924.1         GHKIECLVDTGAEVSLTSLQLQAQRFEQVVGLGGKPVRVGIADHVDTTVG
105 YP_009243641.1      --------------------------------------------------
106 YP_004300274.1      --------------------------------------------------
107 NP_056848.1         --------------------------------------------------
108 YP_006495799.1      SAPPPTPWPNKPTGWGDEEEDLPPPRFDSLSESFS--GGGEIEPLEYHES
109 YP_009508409.1      --------------------------------------------------
110 NP_687035.1         --------------------------------------------------
111 NP_045937.2         MGRGGRMGGPPYRSQPYPAFIQPPQNHQNQYNGRMDRSQLQASAQEWLPG
112 NP_612577.1         --------------------------------------------------
113 NP_619548.1         --------------------------------------------------
114 NP_056880.1         --------------------------------------------------
115 YP_009121747.1      KAKRGNKPNKSRRRSYEKWSTLGEHSGKWDYYVRYDSPKDTTPIDQIIAT
116 YP_009113237.2      TGWESSDEGDEDDEDFRDINSFTAGVS-------LQEQEEQQQMPEVIPE
117 YP_009508408.1      AGVIDPDYRGEISLLLHNPTDSTVSVRKGDFLTQLVLHQILTPPVQEENA
118 NP_955579.1         --------------------------------------------------
119 YP_009229919.1      TYFIPIHGGNTLGQPSGKYDYQVKYTVPEDWKPAHKSQIIATGWDTESSL
120 YP_004442833.1      IQEIEEFLAALSPREEGSE---------------SVSTARCKN-------
121 NP_862833.2         --------------------------------------------------
122 NP_040563.1         --------------------------------------------------
123 YP_443922.1         --------------------------------------------------
124 NP_057933.2         --------------------------------------------------
125 YP_009116631.1      EKK----LKEKELAEFTPETDLVSQWLNQLSNSAHNSGASSSDDEPKFDE
126 YP_009513249.1      --------------------------------------------------
127 YP_223871.1         --------------------------------------------------
128 NP_127504.1         FQLDGRAVSWYKDPFDGHCPWDIDCQCYSCLYSEDEEDFEDGFPT-----
129 NP_044929.1         --------------------------------------------------
130 NP_057860.1         --------------------------------------------------
131 NP_040973.1         --------------------------------------------------
132 NP_777317.1         LAALERLLAPESMVGEHRQPIFDLDDPNPDIIEEDFLNFLDSLTLND---
133 NP_054716.1         --------------------------------------------------
134 YP_001856242.1      --------------------------------------------------
135 NP_954565.2         --------------------------------------------------
136 NP_569150.1         MAAEEEIFPLENMVGEDD----------------IISQFLENLDIT----
137 NP_659397.1         --------------------------------------------------
138 YP_009513211.1      --------------------------------------------------
139 YP_233107.1         LAAVEKQIFPENMVGEEE---------------AIISSFLQKLELSE---
140 YP_009109692.1      --------------------------------------------------
141 NP_057849.4         --------------------------------------------------
142 NP_049560.1         --------------------------------------------------
143 YP_009506267.1      AGVIDPDYTGELKLLLLNPTGAPVHVQAGEFIAQAVLQAIITPPVQEAQA
144 NP_039820.1         YEYPTPQSSPEHLDNESRSRSSSASSTSMQDDVEEIVRLMKEMRMKKQKK
145 NP_056790.1         --------------------------------------------------
146 YP_002321513.1      PNDPNVDFKIHEYCCLSPTHQSCGFFQASTSTAHQSAEQSVVGTSNNQNN
147 NP_955591.1         --------------------------------------------------
148 YP_001956722.2      --------------------------------------------------
149 NP_056902.1         --------------------------------------------------
150 NP_056886.1         --------------------------------------------------
151 NP_041261.1         --------------------------------------------------
```

```
001 YP_004442836.1      --------------------------------------------------
002 NP_056728.1         --------------------------------------------------
003 YP_009508537.1      --------------------------------------------------
004 YP_004732983.2      DPDSPVWKVKKSPYPHKPMKLKD---------------------------
005 YP_009508407.1      --------------------------------------------------
006 NP_040939.1         --------------------------------------------------
```

```
007 YP_003864102.1    --------------------------------------------------
008 YP_009002585.1    FDEEP---------------------------------------------
009 YP_003284237.1    --------------------------------------------------
010 YP_009268869.1    --------------------------------------------------
011 YP_009506251.1    EEEEEE--------------------------------------------
012 NP_955611.1       --------------------------------------------------
013 YP_008567619.1    FTEETVRGTASPLDEDLPYPQLR-------------------------QQ
014 NP_149413.1       --------------------------------------------------
015 YP_004442824.1    --------------------------------------------------
016 YP_001931967.1    --------------------------------------------------
017 YP_002455786.1    --------------------------------------------------
018 YP_009109694.1    --------------------------------------------------
019 NP_789739.1       --------------------------------------------------
020 NP_955619.1       --------------------------------------------------
021 YP_224289.1       --------------------------------------------------
022 YP_009182100.1    VINTVFQTIEGILNVNKQFMTAEKQREQILQSQLKQIKDQLAEPVVLSKP
023 NP_705927.1       --------------------------------------------------
024 YP_009508577.1    --------------------------------------------------
025 YP_004442830.1    --------------------------------------------------
026 NP_758887.1       --------------------------------------------------
027 YP_009508406.1    --------------------------------------------------
028 YP_610965.1       LSDHEVWVPPSHPAEAGGEPCPSTFCE-----------------------
029 YP_001931961.1    --------------------------------------------------
030 YP_241114.1       --------------------------------------------------
031 YP_006732334.1    --------------------------------------------------
032 YP_009041481.1    VPVE-------APEIRKTLEPEP---------------------------
033 YP_001036293.1    LTPTVRGTRGFGTLTAEASNMITKVRVEERPVNPVPTPTDHMEEDDEDAE
034 YP_605811.1       GEFYDADNSLF---------------------------------------
035 NP_955577.1       --------------------------------------------------
036 YP_003208050.1    --------------------------------------------------
037 NP_789740.1       --------------------------------------------------
038 YP_004442839.1    --------------------------------------------------
039 YP_009507791.1    --------------------------------------------------
040 YP_004581513.1    --------------------------------------------------
041 NP_043933.1       --------------------------------------------------
042 YP_009507248.1    --------------------------------------------------
043 YP_002117531.1    Q-------------------------------------------------
044 YP_004347415.1    --------------------------------------------------
045 NP_056891.1       --------------------------------------------------
046 YP_009513242.1    --------------------------------------------------
047 YP_009352866.1    E---------------DSLNPKL---------------------------
048 YP_009165750.1    --------------------------------------------------
049 NP_040840.1       --------------------------------------------------
050 YP_004222728.1    --------------------------------------------------
051 YP_009506264.1    --------------------------------------------------
052 YP_567050.1       --------------------------------------------------
053 YP_009508571.1    --------------------------------------------------
054 NP_056803.1       --------------------------------------------------
055 NP_955564.1       --------------------------------------------------
056 YP_009508411.1    MRPTLRGDQGFGSTSAAVFSIFE-----------------------DDDD
057 YP_009140788.1    FS------------------------------------------------
058 YP_009508410.1    --------------------------------------------------
059 NP_040333.1       --------------------------------------------------
060 YP_008992013.1    --------------------------------------------------
061 NP_041734.1       EEEE----------------------------------------------
062 NP_056895.1       --------------------------------------------------
063 YP_009109689.1    --------------------------------------------------
064 YP_002519387.1    --------------------------------------------------
065 YP_595725.1       --------------------------------------------------
066 YP_009508551.1    --------------------------------------------------
067 YP_001497148.1    --------------------------------------------------
068 NP_041186.1       --------------------------------------------------
069 NP_663784.1       --------------------------------------------------
070 YP_009508443.1    LHQTLRGDRAFGEVTSNYLAEA-----------STSYLADMCP--TFEED
071 YP_003987465.1    C-------------------------------------------------
072 NP_861410.1       --------------------------------------------------
073 YP_009345071.1    IKLIS---------------------------------------------
074 YP_009508582.1    --------------------------------------------------
075 NP_569141.1       --------------------------------------------------
076 YP_002916057.1    --------------------------------------------------
077 NP_777384.2       --------------------------------------------------
078 YP_233110.1       EEFYDADNSLF---------------------------------------
079 NP_542258.1       --------------------------------------------------
```

```
080 YP_009130664.1    FASTEGGGDKAPINN-----------------------------------
081 YP_009508566.1    --------------------------------------------------
082 YP_006607892.1    --------------------------------------------------
083 YP_009506270.1    --------------------------------------------------
084 NP_042513.1       --------------------------------------------------
085 YP_006907834.1    --------------------------------------------------
086 YP_004442827.1    --------------------------------------------------
087 YP_009408594.1    --------------------------------------------------
088 YP_009508561.1    --------------------------------------------------
089 NP_056907.1       --------------------------------------------------
090 YP_002308474.1    --------------------------------------------------
091 YP_007761644.1    --------------------------------------------------
092 NP_040550.1       --------------------------------------------------
093 NP_758808.1       --------------------------------------------------
094 YP_009345075.1    WDDK----------------------------------------------
095 NP_068729.1       --------------------------------------------------
096 NP_047255.1       --------------------------------------------------
097 YP_006273075.1    YKEEES--------------------------------------------
098 YP_009508888.1    --------------------------------------------------
099 NP_395469.1       --------------------------------------------------
100 YP_009508546.1    --------------------------------------------------
101 NP_056762.1       LGLTG---------------------------------------------
102 YP_009508556.1    --------------------------------------------------
103 NP_569153.1       QSPVPSPTQDFTNPFSEGGGESTYPYPSFQPPFDLQSDDSYGTLATWSEY
104 NP_043924.1       --------------------------------------------------
105 YP_009243641.1    --------------------------------------------------
106 YP_004300274.1    --------------------------------------------------
107 NP_056848.1       --------------------------------------------------
108 YP_006495799.1    SEDS------YEEVRRRDMENHN---------------------------
109 YP_009508409.1    --------------------------------------------------
110 NP_687035.1       --------------------------------------------------
111 NP_045937.2       TYPAX---------------------------------------------
112 NP_612577.1       --------------------------------------------------
113 NP_619548.1       --------------------------------------------------
114 NP_056880.1       --------------------------------------------------
115 YP_009121747.1    GWDE----------------------------------------------
116 YP_009113237.2    EEPETP--------------------------------------------
117 YP_009508408.1    LTPTLRGDRGFGALTAAAFIPT-----------SVPTEHNEADDEANEAD
118 NP_955579.1       --------------------------------------------------
119 YP_009229919.1    DDDD-----HPDDSQPRWDTSPEEDNE-----------------------
120 YP_004442833.1    --------------------------------------------------
121 NP_862833.2       --------------------------------------------------
122 NP_040563.1       --------------------------------------------------
123 YP_443922.1       --------------------------------------------------
124 NP_057933.2       --------------------------------------------------
125 YP_009116631.1    ADDE------DDVYNQKTWEKED---------------------------
126 YP_009513249.1    --------------------------------------------------
127 YP_223871.1       --------------------------------------------------
128 NP_127504.1       --------------------------------------------------
129 NP_044929.1       --------------------------------------------------
130 NP_057860.1       --------------------------------------------------
131 NP_040973.1       --------------------------------------------------
132 NP_777317.1       --------------------------------------------------
133 NP_054716.1       --------------------------------------------------
134 YP_001856242.1    --------------------------------------------------
135 NP_954565.2       --------------------------------------------------
136 NP_569150.1       --------------------------------------------------
137 NP_659397.1       --------------------------------------------------
138 YP_009513211.1    --------------------------------------------------
139 YP_233107.1       --------------------------------------------------
140 YP_009109692.1    --------------------------------------------------
141 NP_057849.4       --------------------------------------------------
142 NP_049560.1       --------------------------------------------------
143 YP_009506267.1    LQMTLRGDRGFGEMSRTRLPPVESDDG------SPSSPLSEEVRILFEDD
144 NP_039820.1       KKAQQALSSQAQEEP-----------------------------------
145 NP_056790.1       --------------------------------------------------
146 YP_002321513.1    FPNTTEWLDDTPLEFPEERLSYSQWFSEDPQVLAQPPWSQHSAKVCHVLD
147 NP_955591.1       --------------------------------------------------
148 YP_001956722.2    --------------------------------------------------
149 NP_056902.1       --------------------------------------------------
150 NP_056886.1       --------------------------------------------------
151 NP_041261.1       --------------------------------------------------
```

```
001 YP_004442836.1    --------------------------------------------------
002 NP_056728.1       --------------------------------------------------
003 YP_009508537.1    --------------------------------------------------
004 YP_004732983.2    --------------------------------------------------
005 YP_009508407.1    --------------------------------------------------
006 NP_040939.1       --------------------------------------------------
007 YP_003864102.1    --------------------------------------------------
008 YP_009002585.1    --ATQPQSPQSP-----------------------------------TWY
009 YP_003284237.1    --------------------------------------------------
010 YP_009268869.1    --------------------------------------------------
011 YP_009506251.1    --------------------------------------------------
012 NP_955611.1       --------------------------------------------------
013 YP_008567619.1    QDDTSPLLAETGGHLPN---------------------------------
014 NP_149413.1       --------------------------------------------------
015 YP_004442824.1    --------------------------------------------------
016 YP_001931967.1    --------------------------------------------------
017 YP_002455786.1    --------------------------------------------------
018 YP_009109694.1    --------------------------------------------------
019 NP_789739.1       --------------------------------------------------
020 NP_955619.1       --------------------------------------------------
021 YP_224289.1       --------------------------------------------------
022 YP_009182100.1    VSDPALEGHLKSINRGVKNLVERAELLIDYEELAAKIGKNISTQGIAVSE
023 NP_705927.1       --------------------------------------------------
024 YP_009508577.1    --------------------------------------------------
025 YP_004442830.1    --------------------------------------------------
026 NP_758887.1       --------------------------------------------------
027 YP_009508406.1    --------------------------------------------------
028 YP_610965.1       --------------------------------------------------
029 YP_001931961.1    --------------------------------------------------
030 YP_241114.1       --------------------------------------------------
031 YP_006732334.1    --------------------------------------------------
032 YP_009041481.1    --------------------------------------------------
033 YP_001036293.1    AYGDYLSYLSSRSFSREG------------------------------GE
034 YP_605811.1       --------------------------------------------------
035 NP_955577.1       --------------------------------------------------
036 YP_003208050.1    --------------------------------------------------
037 NP_789740.1       --------------------------------------------------
038 YP_004442839.1    --------------------------------------------------
039 YP_009507791.1    --------------------------------------------------
040 YP_004581513.1    --------------------------------------------------
041 NP_043933.1       --------------------------------------------------
042 YP_009507248.1    --------------------------------------------------
043 YP_002117531.1    --------------------------------------------------
044 YP_004347415.1    --------------------------------------------------
045 NP_056891.1       --------------------------------------------------
046 YP_009513242.1    --------------------------------------------------
047 YP_009352866.1    --------------------------------------------------
048 YP_009165750.1    --------------------------------------------------
049 NP_040840.1       --------------------------------------------------
050 YP_004222728.1    --------------------------------------------------
051 YP_009506264.1    --------------------------------------------------
052 YP_567050.1       --------------------------------------------------
053 YP_009508571.1    --------------------------------------------------
054 NP_056803.1       --------------------------------------------------
055 NP_955564.1       --------------------------------------------------
056 YP_009508411.1    DDDDFPSFAE----------------------------------------
057 YP_009140788.1    --------------------------------------------------
058 YP_009508410.1    --------------------------------------------------
059 NP_040333.1       --------------------------------------------------
060 YP_008992013.1    --------------------------------------------------
061 NP_041734.1       --------------------------------------------------
062 NP_056895.1       --------------------------------------------------
063 YP_009109689.1    --------------------------------------------------
064 YP_002519387.1    --------------------------------------------------
065 YP_595725.1       --------------------------------------------------
066 YP_009508551.1    --------------------------------------------------
067 YP_001497148.1    --------------------------------------------------
068 NP_041186.1       --------------------------------------------------
069 NP_663784.1       --------------------------------------------------
070 YP_009508443.1    PFYEDPTILTLQQVE-----------------------------------
```

071 YP_003987465.1 --
072 NP_861410.1 --
073 YP_009345071.1 --
074 YP_009508582.1 --
075 NP_569141.1 --
076 YP_002916057.1 --
077 NP_777384.2 --
078 YP_233110.1 --
079 NP_542258.1 --
080 YP_009130664.1 ----FPIFDKS---------------------------------------
081 YP_009508566.1 --
082 YP_006607892.1 --
083 YP_009506270.1 --
084 NP_042513.1 --
085 YP_006907834.1 --
086 YP_004442827.1 --
087 YP_009408594.1 --
088 YP_009508561.1 --
089 NP_056907.1 --
090 YP_002308474.1 --
091 YP_007761644.1 --
092 NP_040550.1 --
093 NP_758808.1 --
094 YP_009345075.1 --
095 NP_068729.1 --
096 NP_047255.1 --
097 YP_006273075.1 --
098 YP_009508888.1 --
099 NP_395469.1 --
100 YP_009508546.1 --
101 NP_056762.1 --
102 YP_009508556.1 --
103 NP_569153.1 DAMSQSNSPSSHSDAIQHLSFQHPSADTVLDFDRYSFTTSEDDVVQSAWI
104 NP_043924.1 --
105 YP_009243641.1 --
106 YP_004300274.1 --
107 NP_056848.1 --
108 YP_006495799.1 --
109 YP_009508409.1 --
110 NP_687035.1 --
111 NP_045937.2 --
112 NP_612577.1 --
113 NP_619548.1 --
114 NP_056880.1 --
115 YP_009121747.1 --
116 YP_009113237.2 --
117 YP_009508408.1 DEMEYLSYLSTAPFSG----------------------------------
118 NP_955579.1 --
119 YP_009229919.1 --
120 YP_004442833.1 --
121 NP_862833.2 --
122 NP_040563.1 --
123 YP_443922.1 --
124 NP_057933.2 --
125 YP_009116631.1 --
126 YP_009513249.1 --
127 YP_223871.1 --
128 NP_127504.1 --
129 NP_044929.1 --
130 NP_057860.1 --
131 NP_040973.1 --
132 NP_777317.1 --
133 NP_054716.1 --
134 YP_001856242.1 --
135 NP_954565.2 --
136 NP_569150.1 --
137 NP_659397.1 --
138 YP_009513211.1 --
139 YP_233107.1 --
140 YP_009109692.1 --
141 NP_057849.4 --
142 NP_049560.1 --
143 YP_009506267.1 PFLEDPTIYTIDSFNHS---------------------------------

```
144 NP_039820.1        --------------------------------------------------
145 NP_056790.1        --------------------------------------------------
146 YP_002321513.1     LNTGTSCKPVKPLKTEEQLRYERFERGKPTQADRDAVKYGIEKYWGLLGE
147 NP_955591.1        --------------------------------------------------
148 YP_001956722.2     --------------------------------------------------
149 NP_056902.1        --------------------------------------------------
150 NP_056886.1        --------------------------------------------------
151 NP_041261.1        --------------------------------------------------


001 YP_004442836.1     ------------------DDTKSQ-------VSQ---QVMNLEE-EDFPQ
002 NP_056728.1        --------------------------------------------------
003 YP_009508537.1     --------------------------------------------------
004 YP_004732983.2     ---------EKGKSPFEDLELKQDLVQSWIAQLGSGSGSRTEKPIFDTTS
005 YP_009508407.1     --------------------------------------------------
006 NP_040939.1        --------------------------------------------------
007 YP_003864102.1     --------------------------------------------------
008 YP_009002585.1     NP--FAEGGGSDEND-------ENPDPEESEEMGNEMGY-------ETEV
009 YP_003284237.1     -------------------ELEEE---------TTSMASLNIAMMSEYPE
010 YP_009268869.1     --------------------------------------------------
011 YP_009506251.1     --------------------EEEEEEEEEEEDEYDPNTY-------MAYL
012 NP_955611.1        --------------------------------------------------
013 YP_008567619.1     -------TNYEEEEAISPAVTTPEDIQDDLYQHDLAAIN------TEGPV
014 NP_149413.1        ------------------EEED-IITTFLSAIPDEEEAYPAEEIEEEYPA
015 YP_004442824.1     ------------------EDTRSH-------ISE---KVHHIED---YPS
016 YP_001931967.1     --------------------------------------------------
017 YP_002455786.1     --------------------------------------------------
018 YP_009109694.1     --------------------------------------------------
019 NP_789739.1        --------------------------------------------------
020 NP_955619.1        --------------------------------------------------
021 YP_224289.1        -----------TIQDFLNQYDADI---------SEDEANYDVGVLEEYPE
022 YP_009182100.1     RSKSFMSFDHPKPVESVLPKARMFSMFAGGKPKSSIVRSLELEVEEQPKV
023 NP_705927.1        --------------------------------------------------
024 YP_009508577.1     --------------------------------------------------
025 YP_004442830.1     ----------------CEDDEESIINDFLKLS--HEEVYMAEE-DSDYPA
026 NP_758887.1        --------------------------------------------------
027 YP_009508406.1     --------------------------------------------------
028 YP_610965.1        ----YDSNIEEQFLYDLECLYNAEQSDLASEEEPATVAVGDDACVLSPSQ
029 YP_001931961.1     --------------------------------------------------
030 YP_241114.1        --------------------------------------------------
031 YP_006732334.1     --------------------------------------------------
032 YP_009041481.1     ---------VSDEEVQWEVETIGTEVSDVTQQALP---------IYEEAA
033 YP_001036293.1     KSREVEAEADDDEDLYFRPSPARVTFSDDPFSEDPTIATIRESGDASQPI
034 YP_605811.1        ----------EEEEYDGDDDSEVS-------TPRSKYNIFALED--EYPK
035 NP_955577.1        --------------------------------------------------
036 YP_003208050.1     --------------------------DSDSEEEPDQGFINPFSEDGGGSK
037 NP_789740.1        --------------------------------------------------
038 YP_004442839.1     ----------EEEEYNGDNETETS-------TPRSKYNIFTFED--DYPQ
039 YP_009507791.1     --------------------------------------------------
040 YP_004581513.1     --------------------------------------------------
041 NP_043933.1        --------------------------------------------------
042 YP_009507248.1     --------------------------------------------------
043 YP_002117531.1     ----------VLIEDDRQRQAPARRAAHAEKRRRVEAAEQNRQNIEDFPD
044 YP_004347415.1     --------------------------------------------------
045 NP_056891.1        --------------------------------------------------
046 YP_009513242.1     --------------------------------------------------
047 YP_009352866.1     ---------ETDE--EWEVETIRTETTEVSQTVLP---------IYQEAQ
048 YP_009165750.1     --------------------------------------------------
049 NP_040840.1        --------------------------------------------------
050 YP_004222728.1     --------------------------------------------------
051 YP_009506264.1     ----------LTSPSNSDSETESL---------AESVRSLNIAAL-EYPD
052 YP_567050.1        --------------------------------------------------
053 YP_009508571.1     --------------------------------------------------
054 NP_056803.1        --------------------------------------------------
055 NP_955564.1        --------------------------------------------------
056 YP_009508411.1     ---------GGGEMDYFPFQIEESDEDPID---------------LAQLV
057 YP_009140788.1     ------------------------------SEAELELAY-------QQEV
058 YP_009508410.1     --------------------------------------------------
059 NP_040333.1        --------------------------------------------------
060 YP_008992013.1     -----------------KKKKKPK------KKSHASCHHSTSRPPQDPPP
061 NP_041734.1        -------------------------------DEYDPNVY-------RAYL
062 NP_056895.1        --------------------------------------------------
```

```
063 YP_009109689.1     --------------------------------------------------
064 YP_002519387.1     --------------------------------------------------
065 YP_595725.1        ------------------PEEDQIISDFLGKT---EEAYP-AEIEEEYPA
066 YP_009508551.1     --------------------------------------------------
067 YP_001497148.1     --------------------------------------------------
068 NP_041186.1        --------------------------------------------------
069 NP_663784.1        --------------------------------------------------
070 YP_009508443.1     --------EEDDDISYLTYLYDHHLFFGGEKEEEEAAAAKIFDEVAAAPI
071 YP_003987465.1     ----------KESPSCLFTDDNNG---------DDEGEHHVVAASLEYPE
072 NP_861410.1        --------------------------------------------------
073 YP_009345071.1     ----------------------------------------------KGDI
074 YP_009508582.1     --------------------------------------------------
075 NP_569141.1        --------------------------------------------------
076 YP_002916057.1     --------------------------------------------------
077 NP_777384.2        --------------------------------------------------
078 YP_233110.1        ----------EEEEYDG--DSEVS-------TPRSKYNIFALED--EYPK
079 NP_542258.1        --------------------------------------------------
080 YP_009130664.1     ----------DEEEEILPSSDS--------CRKEIASYI------AAAFP
081 YP_009508566.1     --------------------------------------------------
082 YP_006607892.1     --------------------------------------------------
083 YP_009506270.1     ------------------DDDEDS-------RSQ---HVMNLDE-EEFPE
084 NP_042513.1        --------------------------------------------------
085 YP_006907834.1     --------------------------------------------------
086 YP_004442827.1     ----------------EQDEEEAIINAFLKLTAEEEVLRIEDEEDCEYPA
087 YP_009408594.1     --------------------------------TDQTIAMLHDDDDWSNYP
088 YP_009508561.1     --------------------------------------------------
089 NP_056907.1        --------------------------------------------------
090 YP_002308474.1     --------------------------------------------------
091 YP_007761644.1     --------------------------------------------------
092 NP_040550.1        --------------------------------------------------
093 NP_758808.1        ------------------------------AIEETAFTLEEQFN------
094 YP_009345075.1     ------EEESQEQYDSEKEELAMATRETEIAIEEFSLEQVSTSSEEEEEE
095 NP_068729.1        --------------------------------------------------
096 NP_047255.1        --------------------------------------------------
097 YP_006273075.1     ----------------------QSDEEHYQPDAEAEFDY-------PLKR
098 YP_009508888.1     --------------------------------------------------
099 NP_395469.1        --------------------------------------------------
100 YP_009508546.1     --------------------------------------------------
101 NP_056762.1        ----------------WEDLDQDSIETEEITEWENPNQVLHREIRAYKSV
102 YP_009508556.1     --------------------------------------------------
103 NP_569153.1        SENLFRENTGNGEVHNLVPPRPDTPRGDEVKGTQESMAH-------TVAV
104 NP_043924.1        --------------------------------------------------
105 YP_009243641.1     --------------------------------------------------
106 YP_004300274.1     --------------------------------------------------
107 NP_056848.1        --------------------------------------------------
108 YP_006495799.1     ---------NLIAEGFTQEPRFPGLYAPARIEVIEEDHYEGDASEQSRSE
109 YP_009508409.1     --------------------------------------------------
110 NP_687035.1        --------------------------------------------------
111 NP_045937.2        -------------DPIDCPYEKSGTKTTQDVITTKNAEIMVTVNHTKIPM
112 NP_612577.1        --------------------------------------------------
113 NP_619548.1        --------------------------------------------------
114 NP_056880.1        --------------------------------------------------
115 YP_009121747.1     ----------------------YEDDPWRDDLEEGTVQIKEEDTEQSDKE
116 YP_009113237.2     -----------VEERLRRESYWNEEIDSDEEDPWGLFLN-------RNQY
117 YP_009508408.1     -------VEEGEDEGYFNLAAAEDYPTTIIDAAETSTIG-----FLSAPI
118 NP_955579.1        --------------------------------------------------
119 YP_009229919.1     ----EAESSDSFTEGGGSDNKSDNTPQKSQQEEDERVEVETASESESEKE
120 YP_004442833.1     ----------------KLEEEEEIINDFLKIS--EESVRMAEEEETDYPA
121 NP_862833.2        --------------------------------------------------
122 NP_040563.1        --------------------------------------------------
123 YP_443922.1        --------------------------------------------------
124 NP_057933.2        --------------------------------------------------
125 YP_009116631.1     ---------QEKRE--LELQGWKPTGRPGLYEMIPEQEEE--VYLRYEAE
126 YP_009513249.1     --------------------------------------------------
127 YP_223871.1        --------------------------------------------------
128 NP_127504.1        -----------------KYKGIPRPGSIAERKMQEEANLKKLYEEKDPFV
129 NP_044929.1        --------------------------------------------------
130 NP_057860.1        --------------------------------------------------
131 NP_040973.1        --------------------------------------------------
132 NP_777317.1        ------------DQNSNGQPDDDE-------VSTWSAQINHINDQSEPPA
133 NP_054716.1        --------------------------------------------------
134 YP_001856242.1     --------------------------------------------------
135 NP_954565.2        --------------------------------------------------
```

```
136 NP_569150.1         ------------------DDEEDS-------RSQ---YVMNLEDNEEFPQ
137 NP_659397.1         --------------------------------------------------
138 YP_009513211.1      --------------------------------------------------
139 YP_233107.1         -------------DEESYYGDLES-------EPRSHYEINHLDD--DYPE
140 YP_009109692.1      --------------------------------------------------
141 NP_057849.4         --------------------------------------------------
142 NP_049560.1         --------------------------------------------------
143 YP_009506267.1      -------FNDEEEMDYISYLYHLDDIPAN-----------------DAPI
144 NP_039820.1         -----IIEENIEENKQAQEEPTQEEIPTHKENQPEEIQNEEIHVFEEEPA
145 NP_056790.1         --------------------------------------------------
146 YP_002321513.1      ESGKFDYFVKYRTPPWATHSVCTINEDLPPAFNPDEWDDLPFEYTTDLHS
147 NP_955591.1         --------------------------------------------------
148 YP_001956722.2      --------------------------------------------------
149 NP_056902.1         --------------------------------------------------
150 NP_056886.1         --------------------------------------------------
151 NP_041261.1         --------------------------------------------------


001 YP_004442836.1      LQEIERLLST----------------------------------------
002 NP_056728.1         --------------------------------------------------
003 YP_009508537.1      --------------------------------------------------
004 YP_004732983.2      SDSDSDLSDVSSKVLAYAGVE------------------------EAVME
005 YP_009508407.1      --------------------------------------------------
006 NP_040939.1         --------------------------------------------------
007 YP_003864102.1      --------------------------------------------------
008 YP_009002585.1      EEQEEKVAMI----------------------------------------
009 YP_003284237.1      LKKISELVNA----------------------------------------
010 YP_009268869.1      --------------------------------------------------
011 YP_009506251.1      QKEEDDWTEI----------------------------------------
012 NP_955611.1         --------------------------------------------------
013 YP_008567619.1      WDNSEEEEE-----------------------------------------
014 NP_149413.1         IKRLEELMRTRVIVKEVPEKSE----------------------------
015 YP_004442824.1      LAKVEQVLS-----------------------------------------
016 YP_001931967.1      --------------------------------------------------
017 YP_002455786.1      --------------------------------------------------
018 YP_009109694.1      --------------------------------------------------
019 NP_789739.1         --------------------------------------------------
020 NP_955619.1         --------------------------------------------------
021 YP_224289.1         LLKLENNLAE----------------------------------------
022 YP_009182100.1      LKDYKVPKLSVKQLYEQGSMMQRNAYRIKQIERKIPYDPNTKVIHLVDKG
023 NP_705927.1         --------------------------------------------------
024 YP_009508577.1      --------------------------------------------------
025 YP_004442830.1      IRKLEELIKQ----------------------------------------
026 NP_758887.1         --------------------------------------------------
027 YP_009508406.1      --------------------------------------------------
028 YP_610965.1         SESKSGRELV----------------------------------------
029 YP_001931961.1      --------------------------------------------------
030 YP_241114.1         --------------------------------------------------
031 YP_006732334.1      --------------------------------------------------
032 YP_009041481.1      EQETIATQ-LADELSYKS--------------------------------
033 YP_001036293.1      FDESDDNED----------------------------------------H
034 YP_605811.1         LQQLESLVLS----------------------------------------
035 NP_955577.1         --------------------------------------------------
036 YP_003208050.1      DFEAKTQTAL----------------------------------------
037 NP_789740.1         --------------------------------------------------
038 YP_004442839.1      LQKLDNLVLS----------------------------------------
039 YP_009507791.1      --------------------------------------------------
040 YP_004581513.1      --------------------------------------------------
041 NP_043933.1         --------------------------------------------------
042 YP_009507248.1      --------------------------------------------------
043 YP_002117531.1      EEALMDLLRQ----------------------------------------
044 YP_004347415.1      --------------------------------------------------
045 NP_056891.1         --------------------------------------------------
046 YP_009513242.1      --------------------------------------------------
047 YP_009352866.1      TLKNEAVQGLAEEMSYKN--------------------------------
048 YP_009165750.1      --------------------------------------------------
049 NP_040840.1         --------------------------------------------------
050 YP_004222728.1      --------------------------------------------------
051 YP_009506264.1      AKAINEVIGM----------------------------------------
052 YP_567050.1         --------------------------------------------------
053 YP_009508571.1      --------------------------------------------------
054 NP_056803.1         --------------------------------------------------
```

```
055 NP_955564.1       --------------------------------------------------
056 YP_009508411.1    LEAEEEMLK-----------------------------------------
057 YP_009140788.1    FAAVEEEAE-----------------------------------------
058 YP_009508410.1    --------------------------------------------------
059 NP_040333.1       --------------------------------------------------
060 YP_008992013.1    -----PSAPLH---------------------------------------
061 NP_041734.1       QKEEDEWQEI----------------------------------------
062 NP_056895.1       --------------------------------------------------
063 YP_009109689.1    --------------------------------------------------
064 YP_002519387.1    --------------------------------------------------
065 YP_595725.1       LRRLEQLMKTKVVVQEIEEPS-----------------------------
066 YP_009508551.1    --------------------------------------------------
067 YP_001497148.1    --------------------------------------------------
068 NP_041186.1       --------------------------------------------------
069 NP_663784.1       --------------------------------------------------
070 YP_009508443.1    YDDYDDDADDE----------------------------------EEVQR
071 YP_003987465.1    LRKISEIINS----------------------------------------
072 NP_861410.1       --------------------------------------------------
073 YP_009345071.1    IGQGEVQQEE----------------------------------------
074 YP_009508582.1    --------------------------------------------------
075 NP_569141.1       --------------------------------------------------
076 YP_002916057.1    --------------------------------------------------
077 NP_777384.2       --------------------------------------------------
078 YP_233110.1       LQQLESLVLS----------------------------------------
079 NP_542258.1       --------------------------------------------------
080 YP_009130664.1    ENNAD---------------------------------------------
081 YP_009508566.1    --------------------------------------------------
082 YP_006607892.1    --------------------------------------------------
083 YP_009506270.1    MKEIERVLAS----------------------------------------
084 NP_042513.1       --------------------------------------------------
085 YP_006907834.1    --------------------------------------------------
086 YP_004442827.1    IKRLEELLKQ----------------------------------------
087 YP_009408594.1    KFRPIELIAA----------------------------------------
088 YP_009508561.1    --------------------------------------------------
089 NP_056907.1       --------------------------------------------------
090 YP_002308474.1    --------------------------------------------------
091 YP_007761644.1    --------------------------------------------------
092 NP_040550.1       --------------------------------------------------
093 NP_758808.1       --------------------------------------------------
094 YP_009345075.1    PDWENPFAVL----------------------------------------
095 NP_068729.1       --------------------------------------------------
096 NP_047255.1       --------------------------------------------------
097 YP_006273075.1    IHYLNPFSEG----------------------------------------
098 YP_009508888.1    --------------------------------------------------
099 NP_395469.1       --------------------------------------------------
100 YP_009508546.1    --------------------------------------------------
101 NP_056762.1       SEQIEDIFGELLKEHG----------------------------------
102 YP_009508556.1    --------------------------------------------------
103 NP_569153.1       TTEESKHEAE----------------------------------------
104 NP_043924.1       --------------------------------------------------
105 YP_009243641.1    --------------------------------------------------
106 YP_004300274.1    --------------------------------------------------
107 NP_056848.1       --------------------------------------------------
108 YP_006495799.1    EEPEFDYPQIAAVNQNN----------------------------EPELE
109 YP_009508409.1    --------------------------------------------------
110 NP_687035.1       --------------------------------------------------
111 NP_045937.2       LVDTGACLTAIGGAATVVPDLKLTN-------------------------
112 NP_612577.1       --------------------------------------------------
113 NP_619548.1       --------------------------------------------------
114 NP_056880.1       --------------------------------------------------
115 YP_009121747.1    FTEKIEMIQMIVSPTQEEEEHY------------------AFPTIESIGE
116 YP_009113237.2    WDPEPVHEEP----------------------------------------
117 YP_009508408.1    YDTDGEEGD------------------------------------EDNDD
118 NP_955579.1       --------------------------------------------------
119 YP_009229919.1    GESQAGYMAN----------------------------------------
120 YP_004442833.1    IKRLEELLQQ----------------------------------------
121 NP_862833.2       --------------------------------------------------
122 NP_040563.1       --------------------------------------------------
123 YP_443922.1       --------------------------------------------------
124 NP_057933.2       --------------------------------------------------
125 YP_009116631.1    DEEEDQELQVIGAATMD----------------------------EPEME
126 YP_009513249.1    --------------------------------------------------
127 YP_223871.1       --------------------------------------------------
```

128 NP_127504.1 G S L S R P G K Y E Y -
129 NP_044929.1 -
130 NP_057860.1 -
131 NP_040973.1 -
132 NP_777317.1 L L Q L E Q V L S -
133 NP_054716.1 -
134 YP_001856242.1 -
135 NP_954565.2 -
136 NP_569150.1 L R E I E K V L S S -
137 NP_659397.1 -
138 YP_009513211.1 -
139 YP_233107.1 L L N V E K I L S -
140 YP_009109692.1 -
141 NP_057849.4 -
142 NP_049560.1 -
143 YP_009506267.1 Y D D Y P E E G E - V D
144 NP_039820.1 F K H L A A Q L S E L V N -
145 NP_056790.1 -
146 YP_002321513.1 S D E E K P V P D Y L S M Q T P K P K Y Y Y G A S S S S S S S E S V A S R Y R P K K N E K V Y K P K
147 NP_955591.1 -
148 YP_001956722.2 -
149 NP_056902.1 -
150 NP_056886.1 -
151 NP_041261.1 -

001 YP_004442836.1 - - - - - - - T A E S A I S S -
002 NP_056728.1 -
003 YP_009508537.1 -
004 YP_004732983.2 Y P R R - V K T A T A K L A D M E K A F A G - - - - - - - - - - - - - - - - - E T T A A V G G D S E
005 YP_009508407.1 -
006 NP_040939.1 -
007 YP_003864102.1 -
008 YP_009002585.1 Q E D P D L L P Y P H L R E L L - K N E E K V F S T
009 YP_003284237.1 - - - - - - - S S E V T S S -
010 YP_009268869.1 -
011 YP_009506251.1 I A S - - - - - L N P E M E Y P - Q R R T E V A E T
012 NP_955611.1 -
013 YP_008567619.1 G Y A A E E E E E I D D M E Y P - H V R A L R L K M Q - E V
014 NP_149413.1 - - - - Q P Q E A K M A S S E G -
015 YP_004442824.1 - - - - - - - T G E S A I S S -
016 YP_001931967.1 -
017 YP_002455786.1 -
018 YP_009109694.1 -
019 NP_789739.1 -
020 NP_955619.1 -
021 YP_224289.1 - - - - - - - I P I L S E V T -
022 YP_009182100.1 E M D R M K A K G Y E Y V H F G A V Q V V F R L L A R R D L N C S V L S I C R D N R L K S L Q K G L
023 NP_705927.1 -
024 YP_009508577.1 -
025 YP_004442830.1 - - - - - - E E V R M S D T G -
026 NP_758887.1 -
027 YP_009508406.1 -
028 YP_610965.1 - - - - - - F M E L E A L P Y P T L - A R M E K - - - - - E
029 YP_001931961.1 -
030 YP_241114.1 -
031 YP_006732334.1 -
032 YP_009041481.1 - - - - - L H K M Q K K L V E N - M A L S G A E S -
033 YP_001036293.1 E L I Q E L V S L E A D F D Y P - Q L R Q L E D - - - - C V
034 YP_605811.1 - - - - - - - T T E S A I S R -
035 NP_955577.1 -
036 YP_003208050.1 - - - - - - M T S M E E L P Y P K L - K K L E A - - - - - A
037 NP_789740.1 -
038 YP_004442839.1 - - - - - - - T N E S A I S S -
039 YP_009507791.1 -
040 YP_004581513.1 -
041 NP_043933.1 -
042 YP_009507248.1 -
043 YP_002117531.1 - - - - - - - P C T E T L E H P R E N D D V S L N L A V L E E D N K D E L Y D Y P T A I R N L E N L
044 YP_004347415.1 -
045 NP_056891.1 -
046 YP_009513242.1 -

```
047 YP_009352866.1   -----LHKMQEKLAKN--------------------------MALSGVES-
048 YP_009165750.1   ---------------------------------------------------
049 NP_040840.1      ---------------------------------------------------
050 YP_004222728.1   ---------------------------------------------------
051 YP_009506264.1   -------SSVTSDYRPP----------------------------------
052 YP_567050.1      ---------------------------------------------------
053 YP_009508571.1   ---------------------------------------------------
054 NP_056803.1      ---------------------------------------------------
055 NP_955564.1      ---------------------------------------------------
056 YP_009508411.1   --EVKLTTATLDLDYP----------------------ELEELQR-----I
057 YP_009140788.1   ----------LELAYP--------------------------QRFKAIEKL
058 YP_009508410.1   ---------------------------------------------------
059 NP_040333.1      ---------------------------------------------------
060 YP_008992013.1   ---------------------------------------------------
061 NP_041734.1      TAS-----LREEMEYP--------------------------KRRPQ-TEM
062 NP_056895.1      ---------------------------------------------------
063 YP_009109689.1   ---------------------------------------------------
064 YP_002519387.1   ---------------------------------------------------
065 YP_595725.1      ----QPVEAKMSTSTG-----------------------------------
066 YP_009508551.1   ---------------------------------------------------
067 YP_001497148.1   ---------------------------------------------------
068 NP_041186.1      ---------------------------------------------------
069 NP_663784.1      ---------------------------------------------------
070 YP_009508443.1   LINPKLASISTEFEYP----------------------QLRELEQ-----V
071 YP_003987465.1   -------TVVDSNP-------------------------------------
072 NP_861410.1      ---------------------------------------------------
073 YP_009345071.1   ---VHFTEEEARAELR------------------------KLIAGEEVHME
074 YP_009508582.1   ---------------------------------------------------
075 NP_569141.1      ---------------------------------------------------
076 YP_002916057.1   ---------------------------------------------------
077 NP_777384.2      ---------------------------------------------------
078 YP_233110.1      -------TTESAISR------------------------------------
079 NP_542258.1      ---------------------------------------------------
080 YP_009130664.1   ----------DEIDYP----------------------QLKNL---QQ-HL
081 YP_009508566.1   ---------------------------------------------------
082 YP_006607892.1   ---------------------------------------------------
083 YP_009506270.1   -------VSESAISS------------------------------------
084 NP_042513.1      ---------------------------------------------------
085 YP_006907834.1   ---------------------------------------------------
086 YP_004442827.1   ------ETVQMSEAS------------------------------------
087 YP_009408594.1   ------ETALASS--------------------------------------
088 YP_009508561.1   ---------------------------------------------------
089 NP_056907.1      ---------------------------------------------------
090 YP_002308474.1   ---------------------------------------------------
091 YP_007761644.1   ---------------------------------------------------
092 NP_040550.1      ---------------------------------------------------
093 NP_758808.1      -----------DLDYP------------------------TLISMEKQLVQ
094 YP_009345075.1   ------AADDNDMDYPEL----------------------RALQEIVRSTG
095 NP_068729.1      ---------------------------------------------------
096 NP_047255.1      ---------------------------------------------------
097 YP_006273075.1   GGKDDHL-NSEEMDYP--------------------------QRMKKIEKL
098 YP_009508888.1   ---------------------------------------------------
099 NP_395469.1      ---------------------------------------------------
100 YP_009508546.1   ---------------------------------------------------
101 NP_056762.1      ---------------------------------------------------
102 YP_009508556.1   ---------------------------------------------------
103 NP_569153.1      FDYPAFARLQAHEESG--------------------------RPKPKTEKV
104 NP_043924.1      ---------------------------------------------------
105 YP_009243641.1   ---------------------------------------------------
106 YP_004300274.1   ---------------------------------------------------
107 NP_056848.1      ---------------------------------------------------
108 YP_006495799.1   YPTKKFQELMAKIKGFQNQK----------------------VEIAAMNAE
109 YP_009508409.1   ---------------------------------------------------
110 NP_687035.1      ---------------------------------------------------
111 NP_045937.2      -----------------------------------------TEIIAVGISA
112 NP_612577.1      ---------------------------------------------------
113 NP_619548.1      ---------------------------------------------------
114 NP_056880.1      ---------------------------------------------------
115 YP_009121747.1   EAIKQDEVDEEDLDYPRVREIKRMIKKAKKEG------YTPPIHQEQHIFP
116 YP_009113237.2   EAQEIVTVIQEELDYPHLKRLEE---------------ALKGKTVPVEKKE
117 YP_009508408.1   ILPKRIAVLSFEHDYP----------------------QLRGLEE---HVV
118 NP_955579.1      ---------------------------------------------------
119 YP_009229919.1   ------EEEELELEYPTL----------------------QKLKEKV--EH
```

```
120 YP_004442833.1     ------EQVKMSEVSS----------------------------------
121 NP_862833.2        --------------------------------------------------
122 NP_040563.1        --------------------------------------------------
123 YP_443922.1        --------------------------------------------------
124 NP_057933.2        --------------------------------------------------
125 YP_009116631.1     YPTR-LEKVMGKLKNVSMEKL--------------------FPVSGMDSE
126 YP_009513249.1     --------------------------------------------------
127 YP_223871.1        --------------------------------------------------
128 NP_127504.1        --------------------------------------------------
129 NP_044929.1        --------------------------------------------------
130 NP_057860.1        --------------------------------------------------
131 NP_040973.1        --------------------------------------------------
132 NP_777317.1        -------TNTSAISR-----------------------------------
133 NP_054716.1        --------------------------------------------------
134 YP_001856242.1     --------------------------------------------------
135 NP_954565.2        --------------------------------------------------
136 NP_569150.1        -------VAETAISS-----------------------------------
137 NP_659397.1        --------------------------------------------------
138 YP_009513211.1     --------------------------------------------------
139 YP_233107.1        -------TNESAISN-----------------------------------
140 YP_009109692.1     --------------------------------------------------
141 NP_057849.4        --------------------------------------------------
142 NP_049560.1        --------------------------------------------------
143 YP_009506267.1     VFNLATLDSGFDLDYP---------------------TLQKLEA------
144 NP_039820.1        -------MAESSGQSGVG--------------------------------
145 NP_056790.1        --------------------------------------------------
146 YP_002321513.1     PKPIQTQKAQYQPKKPKPQKEQKVTKEWKPKF-----QVHKLSQDISTEC
147 NP_955591.1        --------------------------------------------------
148 YP_001956722.2     --------------------------------------------------
149 NP_056902.1        --------------------------------------------------
150 NP_056886.1        --------------------------------------------------
151 NP_041261.1        --------------------------------------------------
```

```
001 YP_004442836.1     --------YRPPDVDMSGIAPGYAPATSTT-------------------G
002 NP_056728.1        --------------------------------------------------
003 YP_009508537.1     --------------------------------------------------
004 YP_004732983.2     MTTGQSSRSTLIPPNEGGGPIRYPPAERPSTSAS------------TYNT
005 YP_009508407.1     --------------------------------------------------
006 NP_040939.1        --------------------------------------------------
007 YP_003864102.1     --------------------------------------------------
008 YP_009002585.1     QQSEVSS--YRPPQDTAMTPAGYAPARGEAS-----------------SS
009 YP_003284237.1     --------YRPPEIDMTGPA-GYAPATSQQ-------------------G
010 YP_009268869.1     --------------------------------------------------
011 YP_009506251.1     VFSEIVD--YTPPGDTLMTPVGYPPASSSRS--------------TVTTP
012 NP_955611.1        --------------------------------------------------
013 YP_008567619.1     FSAQSEISQYRPPQDTAMGPSSYPPAVN-----------------ITNEA
014 NP_149413.1        ----SSAIWQPGTMDLDGNFPGYAPAQGAT-------------------G
015 YP_004442824.1     --------YRPPEVEMGGVAPGYAPASGTS-------------------G
016 YP_001931967.1     --------------------------------------------------
017 YP_002455786.1     --------------------------------------------------
018 YP_009109694.1     --------------------------------------------------
019 NP_789739.1        --------------------------------------------------
020 NP_955619.1        --------------------------------------------------
021 YP_224289.1        ------SQYRPADIDMAGPT-GYAPATSQQ-------------------G
022 YP_009182100.1     LGALQASLNNQVAYFVCIPNFSCTIEDAHEALVLCVQTHGTDFKEGYTDL
023 NP_705927.1        --------------------------------------------------
024 YP_009508577.1     --------------------------------------------------
025 YP_004442830.1     ----SSAMIP-ADIDMEGDLPRYAPAQGQT-------------------G
026 NP_758887.1        --------------------------------------------------
027 YP_009508406.1     --------------------------------------------------
028 YP_610965.1        SARSSVTSPYTPPTDSGMAPPGYPPASAGG----------AASSSQMPIL
029 YP_001931961.1     --------------------------------------------------
030 YP_241114.1        --------------------------------------------------
031 YP_006732334.1     --------------------------------------------------
032 YP_009041481.1     ---SGSGAGVYNPYSMREDNSNRPPGYAAAQGLP------------QNIP
033 YP_001036293.1     IAASSAVSNYTPPEDTTMNPPTYPPARID--------------QAGPSHS
034 YP_605811.1        --------FRPTDTDMTGIGPAYAPATGTA-------------------G
035 NP_955577.1        --------------------------------------------------
036 YP_003208050.1     FSSGSVVSNYAPPQDSNMGPPTYPPAPTNG----------ASSSRPMPTL
037 NP_789740.1        --------------------------------------------------
038 YP_004442839.1     --------YNPGDIDMEGVGPGYAPATGGT-------------------S
```

```
039 YP_009507791.1    --------------------------------------------------
040 YP_004581513.1    --------------------------------------------------
041 NP_043933.1       --------------------------------------------------
042 YP_009507248.1    --------------------------------------------------
043 YP_002117531.1    FSSEVTGSYSPPDTDMVGQT-GYAPATSTQ-------------------G
044 YP_004347415.1    --------------------------------------------------
045 NP_056891.1       --------------------------------------------------
046 YP_009513242.1    --------------------------------------------------
047 YP_009352866.1    ---SGSGAGVYNPYDMREDTSNRPPGFAAAQGLR------------QNIP
048 YP_009165750.1    --------------------------------------------------
049 NP_040840.1       --------------------------------------------------
050 YP_004222728.1    --------------------------------------------------
051 YP_009506264.1    -----ALDYRPADVDMTGPS-GYAPSTSAS-------------------G
052 YP_567050.1       --------------------------------------------------
053 YP_009508571.1    --------------------------------------------------
054 NP_056803.1       --------------------------------------------------
055 NP_955564.1       --------------------------------------------------
056 YP_009508411.1    VGASTAMSEYSPPQDTSMNPPTYPPARRE-----------SAASAPSTST
057 YP_009140788.1    LSTSEVTGPYRPPDDAAMGRPSYPPARSISEE-------------GVSSS
058 YP_009508410.1    --------------------------------------------------
059 NP_040333.1       --------------------------------------------------
060 YP_008992013.1    -----LYKKELQWLAKRCTSTISSPVPDPSPPLS-----------CMMFS
061 NP_041734.1       AFSETVD--YTPPGDTMMTPVGYPPASSSRS--------------TVTTP
062 NP_056895.1       --------------------------------------------------
063 YP_009109689.1    --------------------------------------------------
064 YP_002519387.1    --------------------------------------------------
065 YP_595725.1       ----SSAMIP-ANMDMDGNMPGYAPAQEAR-------------------G
066 YP_009508551.1    --------------------------------------------------
067 YP_001497148.1    --------------------------------------------------
068 NP_041186.1       --------------------------------------------------
069 NP_663784.1       --------------------------------------------------
070 YP_009508443.1    IAASSAVSAYNPPEDTTMNPPTYPPAQTP----SMPSTSYGGSTSYGGTF
071 YP_003987465.1    -----GNQYMIGDTDMDGPP-GYAPAQAQA-------------------A
072 NP_861410.1       --------------------------------------------------
073 YP_009345071.1    AESSINSGSYRLPTDATMGPPIYPPARQRFD----------------EPS
074 YP_009508582.1    --------------------------------------------------
075 NP_569141.1       --------------------------------------------------
076 YP_002916057.1    --------------------------------------------------
077 NP_777384.2       --------------------------------------------------
078 YP_233110.1       --------FRPADTDMTGVGPGYAPATGTA-------------------G
079 NP_542258.1       --------------------------------------------------
080 YP_009130664.1    YASPSAITGYKPPQDVAMGPPNYGPTPLR---------------VIRPEA
081 YP_009508566.1    --------------------------------------------------
082 YP_006607892.1    --------------------------------------------------
083 YP_009506270.1    --------FRPPEVEMGGIAPAYAPATSTT-------------------G
084 NP_042513.1       --------------------------------------------------
085 YP_006907834.1    --------------------------------------------------
086 YP_004442827.1    -----SAIIP-AEVDMEGNLPGYAPATTAT-------------------G
087 YP_009408594.1    -STTIAMSPYNPPEDTMMGPPQYPPATARPN---------MPNPWAVPDY
088 YP_009508561.1    --------------------------------------------------
089 NP_056907.1       --------------------------------------------------
090 YP_002308474.1    --------------------------------------------------
091 YP_007761644.1    --------------------------------------------------
092 NP_040550.1       --------------------------------------------------
093 NP_758808.1       SS---VTSAYNPPTEPLMGQVVYPPASAPRP---------QAETSS---T
094 YP_009345075.1    DSSSSIVTNLQPPMDSAMGPPVFPPAREGSR---------PTYLPGNLGF
095 NP_068729.1       --------------------------------------------------
096 NP_047255.1       --------------------------------------------------
097 YP_006273075.1    LSTSEVTSPYRPPEDAAMGRPSYPPARTIPGG-------------GASSS
098 YP_009508888.1    --------------------------------------------------
099 NP_395469.1       --------------------------------------------------
100 YP_009508546.1    --------------------------------------------------
101 NP_056762.1       -NYDMALKNLEEKYDLDKIEKAKSIEEIAKSSTSSEIRPTKRPKEEQTAY
102 YP_009508556.1    --------------------------------------------------
103 NP_569153.1       LSSAISS--YTPPTDTAMTPVAYPPAQNIASP-------------SYNPS
104 NP_043924.1       -QVKGKGCWRISQELAENILGNDLLRSLGLIVDQCNGVLWQASEGLGPDN
105 YP_009243641.1    --------------------------------------------------
106 YP_004300274.1    --------------------------------------------------
107 NP_056848.1       --------------------------------------------------
108 YP_006495799.1    SSGSGVTSGNFVPPIATTGPSVYPPAT--GTHGT------------NIGP
109 YP_009508409.1    --------------------------------------------------
110 NP_687035.1       --------------------------------------------------
111 NP_045937.2       EPVPHVLAKPTKIQIENTNIDISPWYNPDQTFHILGRDTLSKMRAIVSFE
```

```
112 NP_612577.1          --------------------------------------------------
113 NP_619548.1          --------------------------------------------------
114 NP_056880.1          --------------------------------------------------
115 YP_009121747.1       STSEGASGVYNPPPDQVMGPPVYPPARGNVP---------QNSFNLGYPK
116 YP_009113237.2       TVASAVSSAYNPPADTAMMPPAYPPGSADPG------------------P
117 YP_009508408.1       AATSTAVSAYRPPEDTTMNPPTYPPASNLPSAGSVPSTSYP-STSYPTTQ
118 NP_955579.1          --------------------------------------------------
119 YP_009229919.1       MAKSSASSPYRPPQEPYMGTYIYPPASGIT----------EASTSTASTM
120 YP_004442833.1       ----SSALIP-ADVDMEGNLPGYAPAQTKA-------------------G
121 NP_862833.2          --------------------------------------------------
122 NP_040563.1          --------------------------------------------------
123 YP_443922.1          -------------------------------------------------D
124 NP_057933.2          --------------------------------------------------
125 YP_009116631.1       SSITGGGGG-FIPPSPVPGAQGYPPAT--TSTMS------------TIGP
126 YP_009513249.1       --------------------------------------------------
127 YP_223871.1          --------------------------------------------------
128 NP_127504.1          -----LVRYDAPSWAKDPHLTVEPTGWDSDEPIPPKQPFTTRNTLPKIYM
129 NP_044929.1          --------------------------------------------------
130 NP_057860.1          --------------------------------------------------
131 NP_040973.1          --------------------------------------------------
132 NP_777317.1          --------YRPADEDMTGIGPAYTPATSTR-------------------G
133 NP_054716.1          --------------------------------------------------
134 YP_001856242.1       --------------------------------------------------
135 NP_954565.2          --------------------------------------------------
136 NP_569150.1          --------YRPPDAEMGEEAPAYAPATSAT-------------------G
137 NP_659397.1          --------------------------------------------------
138 YP_009513211.1       --------------------------------------------------
139 YP_233107.1          --------YRVP-QDEEMTVPGYAPAGSSR-------------------G
140 YP_009109692.1       --------------------------------------------------
141 NP_057849.4          --------------------------------------------------
142 NP_049560.1          --------------------------------------------------
143 YP_009506267.1       TIASTAVSEYRPPEDTTMNPPIYPPARIS---GQASTSTGPTSTSYTSGP
144 NP_039820.1          ---FQPPVNAQPDVNMEGPA-GYAPATSQA-------------------T
145 NP_056790.1          --------------------------------------------------
146 YP_002321513.1       PNLDKFLKTFEPKKKEPTPPVEKIFSASSSYHVPEDSSMGDTAYTPAPNM
147 NP_955591.1          --------------------------------------------------
148 YP_001956722.2       --------------------------------------------------
149 NP_056902.1          --------------------------------------------------
150 NP_056886.1          --------------------------------------------------
151 NP_041261.1          --------------------------------------------------
```

```
001 YP_004442836.1       WAG-NNQFPFTTRR-PRRWDSNNEFYMLPPAQSKQGAMFIMPLDFDV--K
002 NP_056728.1          --------------------------------------------------
003 YP_009508537.1       --------------------------------------------------
004 YP_004732983.2       TAPPLFEGTVR----PGRYGRPLAPWSLPSAQHSQGALLILPPEVASHAD
005 YP_009508407.1       --------------------------------------------------
006 NP_040939.1          --------------------------------------------------
007 YP_003864102.1       --------------------------------------------------
008 YP_009002585.1       THQPMFEGYMPRRP-QFKQDRSTEYWQFPTAQGQTGAMFVIPRQIGLFHD
009 YP_003284237.1       WAS---SHPPTSGRN-FRYKDPSGYFALPSAQQQNGSILLLSPTYDT--K
010 YP_009268869.1       --------------------------------------------------
011 YP_009506251.1       SRPPLYEGRIPSASRVLRRDDYTEWWQLPSSQGTTGALFVMPKQIGLFHD
012 NP_955611.1          --------------------------------------------------
013 YP_008567619.1       GPSRPMFEGYNKSGVRFKHKDLADNWTLPSAQQQQGAVFVIPTQLGLFDS
014 NP_149413.1          WDNSASSSKKNYGGYSRKFKNESEFFNLPSAMATSGAILVLTMNNYA--K
015 YP_004442824.1       WAG-TSSQLFPFPRRPKKWDDSSEWFALPPAQSRQGAMFVMPYDFDV--K
016 YP_001931967.1       --------------------------------------------------
017 YP_002455786.1       --------------------------------------------------
018 YP_009109694.1       --------------------------------------------------
019 NP_789739.1          --------------------------------------------------
020 NP_955619.1          --------------------------------------------------
021 YP_224289.1          LLG--STAPDRLGKGSFKWKSPTEYFNLPSAQQQAGAMFVMPANFDP--K
022 YP_009182100.1       AIEYIVTYKFMTSDMEAMSKLKATEGFNSMVISDRNNHTISHTKKIDWKE
023 NP_705927.1          --------------------------------------------------
024 YP_009508577.1       --------------------------------------------------
025 YP_004442830.1       WS-DYNPQQKHFGGTSKKWSNPVNHWNLPSANAANGSMLVLSIGKNN--D
026 NP_758887.1          --------------------------------------------------
027 YP_009508406.1       --------------------------------------------------
028 YP_610965.1          GAPFTAADVRANLKNLRGSRSNAGMFQLPSAQQLRGALLVLPEDIGLYQD
029 YP_001931961.1       --------------------------------------------------
030 YP_241114.1          --------------------------------------------------
```

```
031 YP_006732334.1   --------------------------------------------------
032 YP_009041481.1   DVSHFTASARPPPKSKQPYGAP-AMWNLPSAQQTQGVMLVLPYDIGKYAE
033 YP_001036293.1   GPSYS-----VPSGTRFKPMNYSANLQLPSAQQTTGAMFYMPLELDKFDE
034 YP_605811.1      YTG-ASSSDFPYPRRPRKWDNNSEWFNLPTANARQASIFVMPQDFDT--K
035 NP_955577.1      --------------------------------------------------
036 YP_003208050.1   G----LEGARPKVSDFR-RRETSNQWNLPSAQQVNGAMLVLPEDIGLYEE
037 NP_789740.1      --------------------------------------------------
038 YP_004442839.1   YTG-AGTSDFPYPRRPRKWENSSEWFNLPMANARQASVFIMPQDFDT--K
039 YP_009507791.1   --------------------------------------------------
040 YP_004581513.1   --------------------------------------------------
041 NP_043933.1      --------------------------------------------------
042 YP_009507248.1   --------------------------------------------------
043 YP_002117531.1   WVG-SHKFEGSTSKARFKWKDPSEMFTLPSAYQVEGAIFVMPPDYDP--K
044 YP_004347415.1   --------------------------------------------------
045 NP_056891.1      --------------------------------------------------
046 YP_009513242.1   --------------------------------------------------
047 YP_009352866.1   EVSNFVGGARPPPKSKVPYGAP-ANWNLPSAQQSQGVMLVLPYDIGMYAE
048 YP_009165750.1   --------------------------------------------------
049 NP_040840.1      --------------------------------------------------
050 YP_004222728.1   --------------------------------------------------
051 YP_009506264.1   YKE---GERSFGFRGGFRWKNPSENFQLPSAQQQTGAIFIMPPNFDP--K
052 YP_567050.1      --------------------------------------------------
053 YP_009508571.1   --------------------------------------------------
054 NP_056803.1      --------------------------------------------------
055 NP_955564.1      --------------------------------------------------
056 YP_009508411.1   GISSG--APTVSAGTRFRGKDYSGAWTLPSAQQTTGAIFNMPLELGKFDE
057 YP_009140788.1   NYPSKFN----AARTRFRGGYNDEMWTLPSAQQKNGAMFVLPEQLGLFND
058 YP_009508410.1   --------------------------------------------------
059 NP_040333.1      --------------------------------------------------
060 YP_008992013.1   SASSDYSSYFPPLDTHTDSQRNVVSKPFIPSPITSAGHLEPPKPFESVLN
061 NP_041734.1      SRPPLFEGRTTHVPRFLKRDEYTEWWQLPSSQGTTGALFVMPKQMGLFHE
062 NP_056895.1      --------------------------------------------------
063 YP_009109689.1   --------------------------------------------------
064 YP_002519387.1   --------------------------------------------------
065 YP_595725.1      WD-SGETSRRNYGGHSRKWKDESQFFNLPSAMATSGAMLVLTMGNYA--K
066 YP_009508551.1   --------------------------------------------------
067 YP_001497148.1   --------------------------------------------------
068 NP_041186.1      --------------------------------------------------
069 NP_663784.1      --------------------------------------------------
070 YP_009508443.1   GGTSG-----VGSGTRFRSKDYSGNWNLPSAQQQTGAMLYIPLELGKFDE
071 YP_003987465.1   AVPPTYGGTSPSVRGPFRWKNPHEHFELPSAHQLSGAIFVMPQNFDP--K
072 NP_861410.1      --------------------------------------------------
073 YP_009345071.1   TSTVPMYPPQYRAPLRAKVPDFQNWWNLPTAQINGGAMFIIPSDFSKFDD
074 YP_009508582.1   --------------------------------------------------
075 NP_569141.1      --------------------------------------------------
076 YP_002916057.1   --------------------------------------------------
077 NP_777384.2      --------------------------------------------------
078 YP_233110.1      YTG-ASSSDFPYPRRPRKWDNNSEWFNLPTANARQASIFVMPQDFDT--K
079 NP_542258.1      --------------------------------------------------
080 YP_009130664.1   GPLRPAFEGYKPGEVRYKAKDYSEWWNLPSAQHGTGAIFIIPTQLGMFND
081 YP_009508566.1   --------------------------------------------------
082 YP_006607892.1   --------------------------------------------------
083 YP_009506270.1   WAG-TTEFPFTTARRPRVWDSNNEFYMLPPAQSRQGAIFIMPMDFDV--K
084 NP_042513.1      --------------------------------------------------
085 YP_006907834.1   --------------------------------------------------
086 YP_004442827.1   WS-DYNPKINRIGGTSSKWNNQTNYWNLPNANAVNGSMLILCYGKNS--E
087 YP_009408594.1   AT-------RNAGRARFQGGIPGEQWQLPSAQQIAGAMLVLPDDIGMYND
088 YP_009508561.1   --------------------------------------------------
089 NP_056907.1      --------------------------------------------------
090 YP_002308474.1   --------------------------------------------------
091 YP_007761644.1   --------------------------------------------------
092 NP_040550.1      --------------------------------------------------
093 NP_758808.1      SERF-------KNFRAKP--YSTPTIFLPPAYNQQGAILVLPDDIGLYED
094 YP_009345075.1   GDG-----LAXPKRYPTARLPTQNFWELPSAQINRGALLILPNDIGKYDD
095 NP_068729.1      --------------------------------------------------
096 NP_047255.1      --------------------------------------------------
097 YP_006273075.1   AEPPRFD----AARTRFKGGYNDEMWTLPSAQQKNGAMFVIPEQLGLFND
098 YP_009508888.1   --------------------------------------------------
099 NP_395469.1      --------------------------------------------------
100 YP_009508546.1   --------------------------------------------------
101 NP_056762.1      EDDMRDDWKRKELTVNPIEASKDRNFERIGSSYKKNFYPSRSEILNLDNV
102 YP_009508556.1   --------------------------------------------------
103 NP_569153.1      PQMPMFEGYYPKRP-NFKRD-NHAFISLPSAQQNTGALFIMPQQIGLFHE
```

```
104 NP_043924.1       WMAAETLRIYSIKSPGHYNLPELLATKDEQLADILWNNVEAFATHRNDCG
105 YP_009243641.1    --------------------------------------------------
106 YP_004300274.1    --------------------------------------------------
107 NP_056848.1       --------------------------------------------------
108 YP_006495799.1    QDQGGWGGRMPRSRMPGGYGRPQRPWTLPSAQTENGVMLIIPEDLTLAAD
109 YP_009508409.1    --------------------------------------------------
110 NP_687035.1       --------------------------------------------------
111 NP_045937.2       KNGEMTVLLPPTYHKQLSCQTKNTLNIDEYLLQFPDQLWASLPTDIGRML
112 NP_612577.1       --------------------------------------------------
113 NP_619548.1       --------------------------------------------------
114 NP_056880.1       --------------------------------------------------
115 YP_009121747.1    YARE-------SDFNGDYGRYHSSQWTLPPALTDSGAMLVLPADPGLWSD
116 YP_009113237.2    STTPIFKG-YDGAKVKIKREEGSEYWQLPSAQNQTGALFVIPRQLGMFDE
117 YP_009508408.1    GATSG-----VGSGTRFRAKDYSGNWNLPSAQQTTGALFYIPLELGKFDE
118 NP_955579.1       --------------------------------------------------
119 YP_009229919.1    R--------LPRLRHQG-RDPTVNWWSLPPAQNQQGAMLILPQDIGLYTD
120 YP_004442833.1    YS-DYNPGETRFGGYSRKWSNPINNWSLPVANAATGSMLVLSIGKNS--E
121 NP_862833.2       --------------------------------------------------
122 NP_040563.1       --------------------------------------------------
123 YP_443922.1       TTEICKPERLSLPRHHGKPYSDHPSATALLDSIDEAIWTTSPFDVGRLAV
124 NP_057933.2       --------------------------------------------------
125 YP_009116631.1    ADMQGWGGRVPRSRSPLGYGRPQQPWSLPSAQSDNGCMLVLPQDFTLIPD
126 YP_009513249.1    --------------------------------------------------
127 YP_223871.1       --------------------------------------------------
128 NP_127504.1       FNPLNYENNFPPLSSFSKDGADHTPKIPKRNVVLPSGAKDPTGDLEATVN
129 NP_044929.1       --------------------------------------------------
130 NP_057860.1       --------------------------------------------------
131 NP_040973.1       --------------------------------------------------
132 NP_777317.1       WTG-ENS-NFPPVTAPRRWDNSSERFQLPSAQGTHGAIFVMPYDFDT--K
133 NP_054716.1       --------------------------------------------------
134 YP_001856242.1    --------------------------------------------------
135 NP_954565.2       --------------------------------------------------
136 NP_569150.1       WAG-SRPFPFMPKGGPRRWDSNNEFYSLPPAQSRQGAMFVMPMDFDI--K
137 NP_659397.1       --------------------------------------------------
138 YP_009513211.1    --------------------------------------------------
139 YP_233107.1       WAT-NIDDAMLHQRKPKRWDNSSEWFQLPSANARQGSIFVMPYDFDV--K
140 YP_009109692.1    --------------------------------------------------
141 NP_057849.4       --------------------------------------------------
142 NP_049560.1       --------------------------------------------------
143 YP_009506267.1    GPSSSGYSQRTPSGVHFRARDYSNNWSLPSAQQNTGAMFYLPLELVKFDE
144 NP_039820.1       WSN---GVNIPVKSANFRWKGPVGNFQLPSAQGKDGAMLVFGMNYSP--E
145 NP_056790.1       --------------------------------------------------
146 YP_002321513.1    ITPYNPPGVDARRGRARGRPYVPPTFNLPLAGNDTGLMLELS-DPSMYNE
147 NP_955591.1       --------------------------------------------------
148 YP_001956722.2    --------------------------------------------------
149 NP_056902.1       --------------------------------------------------
150 NP_056886.1       --------------------------------------------------
151 NP_041261.1       --------------------------------------------------
```

```
001 YP_004442836.1    VFERWESITLLHMTE--RSFDNADDKMRYMENLLGEDEKKHFIEWRM--K
002 NP_056728.1       --------------------------------------------------
003 YP_009508537.1    -------------PTPQKQYHINPKAKPDIQIVINDLLKQGVLIQK----
004 YP_004732983.2    AITTWETITLNHLMN--ISFDSLQDRVDYIENLLGPREREAWVTWRM--A
005 YP_009508407.1    --------------------------------------------------
006 NP_040939.1       -------------GPHVPQWPLTEEKLKGLTEIIDKLVEEGKLGKAP--P
007 YP_003864102.1    -------------PVWVDQWPLTKEKLTAAQQLVQEQLQAGHIVES----
008 YP_009002585.1    AFNRWESITKNYVAS--QGFTDSKDKAEFMENLLGETEKLTWVQWRM--N
009 YP_003284237.1    IFERWESTTLNHMAD--KSFATAEDKLIYLENLLGEMEKKTFQTWRM--A
010 YP_009268869.1    -------------GPHIAQWPLTQEKLEGLKEIVDKLEKEGKVGRAP--P
011 YP_009506251.1    VFSRWESITKNYVSA--QGITDPTEKMEFIENLLGETEKLTWIQWRM--R
012 NP_955611.1       -------------PVWIDQWPLPEGKLVALTQLVEKELQLGHIEPS----
013 YP_008567619.1    VFARWESITKNLVAA--QGFTDPADKVDFIENLLGEAEKLTWIQWRS--T
014 NP_149413.1       EFERWQSINTNLLAT--QSFETADDKITRIENLLGETEKLMFQTWRM--A
015 YP_004442824.1    VFERWESITLLYLSE--RSFDTADDKLRTVENLLGENEKKMFIAWRM--K
016 YP_001931967.1    --------------------------------------------------
017 YP_002455786.1    -------------APRGQPVSFKPERLQALIDLVSKALEAGHIEPY----
018 YP_009109694.1    -----------AVPVSIKQYPMSLEAHMGIRQHIIKFLELGVLRPC----
019 NP_789739.1       -------------GPKVKQWPLTEEKIKALVEICTEMEKEGKISKIG--P
020 NP_955619.1       -------------PPEISQFPLNPERLQALQHLVRKALEAGHIEPY----
021 YP_224289.1       VFERWESITLNHMAD--KVFSTAEDKLIYLENLLGEAEKIMFQSWRM--T
022 YP_009182100.1    LNQSEFPKEWSINSEEPPEAKSSSVITGIYQHENGNISMRFHQAGEG--S
```

```
023 NP_705927.1        -------------GPKVKQWPLTEEKIKALVEICTEMEKEGKISKIG--P
024 YP_009508577.1     -------------PRPQRQYHINTKAKPSIQQVIDDLLKQGVLIKQ----
025 YP_004442830.1     IFERWESSTLNYMAS--QNISGSEEKIARIENLLGETEKKTFIGWRM--A
026 NP_758887.1        -------------GPKIKQWPLSKEKILALQEICSQMEKEGQISKIG--P
027 YP_009508406.1     --------------------------------------------------
028 YP_610965.1        VISRWETITLNVLAD--KVFSDNRSKLMYVENLLGEDEKKAWIQWRM--A
029 YP_001931961.1     --------------------------------------------------
030 YP_241114.1        -------------PVWVDQWPLTQEKLSAAQQLVQEQLRLGHIEPS----
031 YP_006732334.1     --------------------------------------------------
032 YP_009041481.1     VINRWESINVNLVNS--MNWDSNQDKVDFMENLLGETEKQIFVSWRM--Q
033 YP_001036293.1     VFSRWESITKNITSQ--HSFISGREKADFIENLLGETEKLTWIQWRT--T
034 YP_605811.1        VFERWESSVLLHMSD--KVFDDPQDKLTYVENLLGESEKKMFITWRM--M
035 NP_955577.1        -----------ATPISIRQYPMPHEAYQGIKPHIRRMLDQGILKPC----
036 YP_003208050.1     VISRWESITINLLND--RTWDNNKARVIYVENLLGESEKKIWQQWRT--T
037 NP_789740.1        -------------GPKVKQWPLTEEKIKALVEICTEMEKEGKISKIG--P
038 YP_004442839.1     VFERWESSVLLHMTD--KIFDDPQDKLTYIENLLGESEKKMFITWRM--K
039 YP_009507791.1     -------------GPKIKQWPLTNEKIEALTEIVERLETEGKVKRAD--P
040 YP_004581513.1     --------------------------------------------------
041 NP_043933.1        --------------------------------------------------
042 YP_009507248.1     --------------VYTKSYRYPFIHRQEVRDQITKMLDQGIIRPSDSAW
043 YP_002117531.1     VFSRWESITLNHMAP--KAFANATDKITYIENLLGENEKITFQTWRM--S
044 YP_004347415.1     --------------------------------------------------
045 NP_056891.1        -------------PVWVDQWPLTNDKLAAAQQLVQEQLEAGHITES----
046 YP_009513242.1     -------------PRPQKQYPINPKAKPSIQIVIDDLLKQGVLIQQ----
047 YP_009352866.1     VINRWESINVNVVNS--MNWDSNQDKVDFIENLLGETEKQIVVSWRM--Q
048 YP_009165750.1     --------------------------------------------------
049 NP_040840.1        -------------GPHIAQWPLTQEKLEGLKEIVDRLEKEGKVGRAP--P
050 YP_004222728.1     -------------PVWIDQWPLPEGKLVALTQLVEKELQLGHIEPS----
051 YP_009506264.1     VFERWESVVLNFLAD--KNFPTAEDKLIYIENMLGEMEKITFQTWRM--A
052 YP_567050.1        -------------PPQIDQFPFKPERLQALTDLVSKALEASYIEPY----
053 YP_009508571.1     -------------PRPQKQYPINPKARSSIQVVIDDLLKQGVLVQQ----
054 NP_056803.1        -------------PRPQKQYPINPKAKPSIQIVIDDLLKQGVLTPQ----
055 NP_955564.1        -------------PVWLNQWPLKQEKLQALQQLVTEQLQLGHLEES----
056 YP_009508411.1     VFMRWESVTKNLTAQ--HVFTSGRDKAEFIENLLGEYEKLTWLQWRT--A
057 YP_009140788.1     AFSRWESVTKNHVAT--QSFTDVRDKMEYMENLLGEIEKLIWIQWRM--T
058 YP_009508410.1     --------------------------------------------------
059 NP_040333.1        -----------STPVSIKQYPMSQEARLGIKPHIQRLLDQGILVPC----
060 YP_008992013.1     WQTQNARAQNDTLLNINSKVEKISLRTEHLET---KVDSISAQLQQI--H
061 NP_041734.1        VFSRWESITKNYVAA--QGFTDPTEKMEFMENLLGETEKLTWIQWRM--N
062 NP_056895.1        -------------PPEVPQFPLNLERLQALQDLVHRSLEAGYISPW----
063 YP_009109689.1     -----------AVPVSIKQYPMSLEAHMGIRQHIIKFLELGVLRPC----
064 YP_002519387.1     --------------------------------------------------
065 YP_595725.1        EFDRWQSINTNLLAS--QTFENAEDKITRIENLLGETEKLMFQTWRM--A
066 YP_009508551.1     -------------PRPQKQYPINPKAKPSIQIVIDDLLKQGVLTPQ----
067 YP_001497148.1     -----------AMPVSIRQYPMSKEAHMGIQPHITRFLELGVLRPC----
068 NP_041186.1        --------------------------------------------------
069 NP_663784.1        -------------GPRLKQWPLTKEKIEALKEICEKMEKEGQLEEAP--P
070 YP_009508443.1     VFMRWESITKNIISQ--HTFISGKEKADYIENLLGEYEKLTWIQWRT--A
071 YP_003987465.1     VFGRWESIVLNHLAD--RNFPTALDKIIYVENLLGEMEKITFQTWRM--R
072 NP_861410.1        --------------------------------------------------
073 YP_009345071.1     VFMRWESITKNVVAL--QNFSDNKDKAEFIENLLGETEKLAWMQWRS--I
074 YP_009508582.1     -------------PRPQRQYHINTKAKPSIQQVIDDLLKQGVLVKQ----
075 NP_569141.1        --------------------------------------------------
076 YP_002916057.1     --------------------------------------------------
077 NP_777384.2        -------------PPEVPQFPLNLERLQALQDLVHRSLEAGYISPW----
078 YP_233110.1        VFERWESSVLLHMSD--KVFDDPQDKLTYVENLLGESEKKMFITWRM--M
079 NP_542258.1        -------------APGGLAVPVKPERLQALTDLVSRALEAKHIEPY----
080 YP_009130664.1     TFMRWESITKNLVSQ--QGFTDPNDKVEFIENLLGEAEKIAWIQWRM--T
081 YP_009508566.1     -------------PKPQKQYHINPKAKPSIQIVINDLLKQGVLKQQ----
082 YP_006607892.1     --------------------------------------------------
083 YP_009506270.1     VFERWESITLLHMAE--RSFDNADDKMRYIENLLGEDEKKHFVEWRM--K
084 NP_042513.1        --------------------------------------------------
085 YP_006907834.1     --------------------------------------------------
086 YP_004442827.1     IFERWESSTLNYMAA--QNISGGEEKISRIENLLGESEKRTFIGWRT--A
087 YP_009408594.1     VISRWESITTNLVDS--KVWGDVTTKINFIENLLGEIEKKLFVQWRM--M
088 YP_009508561.1     -------------PKPQKQYHINPKAKPSIQIVINDLLKQGVLRQS----
089 NP_056907.1        -------------PPQIDQFPFKPERLQALTDLVSKALEASYIEPY----
090 YP_002308474.1     -------------APRSSTISVKPERLQALTDLVSKALEAKYIEPY----
091 YP_007761644.1     --------------------------------------------------
092 NP_040550.1        -------------PVWIDQWPLPEGKLVALTQLVEKELQLGHIEPS----
093 NP_758808.1        TISRWESITLNMMNE--KVWPSNEAKAKYMENLLGEMEKKTWIQWRT--T
094 YP_009345075.1     VITRWSTINSNLLSQ--TDFSDNATKVQFVENLLGEDEKRLFQQWRS--N
095 NP_068729.1        --------------------------------------------------
```

096 NP_047255.1 -----------ATPISIRQYPMPHEAYQGIKPHIRRMLDQGILKPC----
097 YP_006273075.1 AFSRWESVTKNHVAT--QGFTDTRDKIKYMENLLGEIEKLIWIQWRM--T
098 YP_009508888.1 -------------PRPQKQYPINPKAKESIQIVINDLLKQGVLIQQ----
099 NP_395469.1 --
100 YP_009508546.1 -------------PRPQKQYPINPKAKPSIQIVIDDLLKQGVLTPQ----
101 NP_056762.1 PPQFYYDQLVTWEGIVKNEWEARKKDGMDMWSWMDGRITGLVLYLVQ--D
102 YP_009508556.1 -------------PRPQKQYPINPKAKPSIQIVIDDLLKQGVLIQQ----
103 NP_569153.1 VFTSWEAITKAYVAQ--QGITDPRDKAEFIENMLGPTEKIIWTQWRM--G
104 NP_043924.1 NLQGMTASFTADHPKMIKQYPVPDASHASIKETVEALLEQGVLRKCN---
105 YP_009243641.1 -------------PIWIEQWPLTREKLQAAEELVEEQLQKGHIETS----
106 YP_004300274.1 --
107 NP_056848.1 --
108 YP_006495799.1 AINRWESITINVVSK--LAFDNMQDKVDYVENLLGEREKEVWTTWRM--K
109 YP_009508409.1 --
110 NP_687035.1 -------------GPKLKQWPLSREKIEALTEICKQMEEEGKLSRIG--G
111 NP_045937.2 VPPITIKIKDNASLPSIRQYPLPKDKTEGLRPLISSLENQGILIKC----
112 NP_612577.1 --
113 NP_619548.1 --
114 NP_056880.1 -------------PVWLNQWPLKQEKLQALQQLVTEQLQLGHLEES----
115 YP_009121747.1 VLARWESITINRLND--TIWANNKAKLMFVENLLGENEKKMWQQWRT--A
116 YP_009113237.2 VFTRWESITKNYVSL--QMFSSGQEKMDFIENLLGEKEKLVWISWRM--A
117 YP_009508408.1 TFMRWESITKNLVSQ--HTFTSGRDKAEFIENLLGEYEKLAWIQWRT--S
118 NP_955579.1 -----------ATPISIRQYPMPHEAYQGIKPHIRRMLDQGILKPC----
119 YP_009229919.1 VVSRWESVTLNVIDDPMKSWSSNRQKISFIENLLGEDEKKIWQQWKA--A
120 YP_004442833.1 IFERWESTTLNYMAS--QNIIGAEEKISRIENLLGETEKKIFIGWRT--Q
121 NP_862833.2 -------------PVWVDQWPLTQEKLSAAQQLVQEQLRLGHIEPS----
122 NP_040563.1 -------------GPKVPQWPLTKEKYQALKEIVKDLLAEGKISEAA--W
123 YP_443922.1 PPVRVQLTHQGQLPVYRSQYRLKYEQIEGIRPTVEGLLGADCIYAT----
124 NP_057933.2 -----------STPVSIKQYPMSQEARLGIKPHIQRLLDQGILVPC----
125 YP_009116631.1 VINRWESITVNLINK--MMFDSLQDKADYVENLLGEREKETWMTWRM--Q
126 YP_009513249.1 -------------PTPQKQYHINPKAKPDIQIVINDLLKQGVLIQK----
127 YP_223871.1 -----------ALPVRVRQYPITLEAKRSLRETIHKFRAAGILRPV----
128 NP_127504.1 WQTENALAQNRMLTTIDRTLKETVTKVDRVTDQSSKNQGLIKVLEQQ--L
129 NP_044929.1 -------------PRPQKQYHINPRAKADIQIVIDDLLRQGVLRQQ----
130 NP_057860.1 -------------PPEISQFPLNPERLQALQHLVRKALEAGHIEPY----
131 NP_040973.1 -------------GPQIKQWPLTNEKIEALTEIVERLEREGKVKRAD--P
132 NP_777317.1 VFDRWESTTLLYLSE--RNFDTAEDKIRVIENLLGESEKKMFIAWRM--N
133 NP_054716.1 -------------PKPQKQYRINPKAKADIQIVIDDLLKQGVLKQQ----
134 YP_001856242.1 -------------GPKVKQWPLTEEKIKALVEICTEMEKEGKISKIG--P
135 NP_954565.2 -------------PVWVDQWPLTNDKLAAAQQLVQEQLEAGHITES----
136 NP_569150.1 VFERWESITLLHMTE--KIFDNADDKMRYMENLLGEDEKKHFIEWRM--K
137 NP_659397.1 --
138 YP_009513211.1 -----------ASPVAVRQYPMSKEAREGIRPHIQRFLDLGILVPC----
139 YP_233107.1 VFERWESSVLVHLAD--KNFDTPEDKVIYIENLLGESEKKAFMTWRM--K
140 YP_009109692.1 -----------AVPVSIKQYPMSLEAHMGIRQHIIKFLELGVLRPC----
141 NP_057849.4 -------------GPKVKQWPLTEEKIKALVEICTEMEKEGKISKIG--P
142 NP_049560.1 --
143 YP_009506267.1 VFSRWESITKNLVSQ--QSFITGKEKAEFIENLLGETEKLTWIQWRT--A
144 NP_039820.1 VFDRWASITRNYISS--FNFNDGGDKIAWMEDLLGETERKIFVSWRM--R
145 NP_056790.1 -----------ASPVAVRQYPMSKEAREGIRPHIQKFLDLGVLVPC----
146 YP_002321513.1 ILDQWESSTKNLLNG--QQFDSNRSKVEYVENLLGSTAKKTFLQWKV--A
147 NP_955591.1 -----------STPVSIKQYPMSQEARLGIKPHIQRLLDQGILVPC----
148 YP_001956722.2 -------------PRPQKQYPINPKAKASIQTVINDLLKQGVLIQQ----
149 NP_056902.1 -------------GPKIPQWPLTKEKLEGAKEIVQRLLSEGKISEAS--D
150 NP_056886.1 -------------PVWIDQWPLPEGKLVALTQLVEKELQLGHIEPS----
151 NP_041261.1 --

001 YP_004442836.1 YTTEYETMKAQALGDQGTQNIINQMRLIFFLENPQVGTTTSQDAAYKTLK
002 NP_056728.1 --
003 YP_009508537.1 ESTMNTPVYPVPKP-NGRWRMVLDYRAVNKVTPLIAVQNQHSYGILGSLF
004 YP_004732983.2 YDTEYRQLVEISGE---PRNVTSTIKRVLGINDPYTGTTHIQNQAYADLE
005 YP_009508407.1 --
006 NP_040939.1 HWTCNTPIFCIKKK-SGKWRMLIDFRELNKQTEDLTEAQLGLP-HPGGLQ
007 YP_003864102.1 NSPWNTPIFVIKKK-SGKWRLLQDLRAVNATMILMGALQPGLP-SPVAIP
008 YP_009002585.1 FPTEYQEMVNAADGREGTQNILSQMRRVFTLEDPTTGSTAVQDEAYRDLE
009 YP_003284237.1 FPTEFELMKTQALGANGTQNSISQIRRIFYLEDPKTGSTTSQDAAYKAIK
010 YP_009268869.1 HWTCNTPIFCIKKK-SGKWRMLIDFRELNKQTEDLAEAQLGLP-HPGGLQ
011 YP_009506251.1 YEAEYQQLLTIAEGRQGTQNILSQIKRVFSLEDPASGSTRIQDSAYRDLE
012 NP_955611.1 LSCWNTPVFVIRKA-SGSYRLLHDLRAVNAKLVPFGAVQQGAP-VLSALP
013 YP_008567619.1 YQAEYQQMMNSADGRAGTENILSQVRRIFALEDPSTGSTKIQDEAYRDLE
014 NP_149413.1 FPNAFDTLRGQALGTNGTANVFAQIKRVLLGEVPEQGTTGVQDAAYKKIK

015	YP_004442824.1	YTNEYETMKAQALGDNGSQNILNQLRLIFFLENPQVGTTDSQDAAYKTLK
016	YP_001931967.1	--
017	YP_002455786.1	SGPGNNPVFPVKKP-NGKWRFIHDLRATNAITTTLASPSPGPP-DLTSLP
018	YP_009109694.1	RSPWNTPLLPVKKPGTQDYRPVQDLREINKRTVDIHPTVPNPYNLLSTLK
019	NP_789739.1	ENPYNTPVFAIKKKDSTKWRKLVDFRELNKRTQDFWEVQLGIP-HPAGLK
020	NP_955619.1	TGPGNNPVFPVKKA-NGTWRFIHDLRATNSLTIDLSSSSPGPP-DLSSLP
021	YP_224289.1	YANEYEEMKGQALGNNGTQNLLSQIRRIFYLEDPKTGTTVSQDAAYKAIK
022	YP_009182100.1	SHSKAQTHPVHIFDDEPIQLDVSKSGRPKARMIRASTRKEVVVELHDINS
023	NP_705927.1	ENPYNTPVFAIKKKDSTKWRKLVDFRELNKRTQDFWEVQLGIP-HPAGLK
024	YP_009508577.1	TSVMNTPIYPVPKP-DGKWRMVLDYRAVNKTVPLIGAQNQHSLGILTNLV
025	YP_004442830.1	YTTEFELLKAQAIGVNGTQNVISQIRRILLGDSPKQGTTLTQDAAYKRIK
026	NP_758887.1	ENPYNTPVFCIKKKDGTSWRKLVDFRQLNKVTQDFFEVQLGIP-HPGGLK
027	YP_009508406.1	--
028	YP_610965.1	FEQEYESLIAVADD---SQAIISQVRRIITLEDPSQGSTEEQDRAYMDLG
029	YP_001931961.1	--
030	YP_241114.1	TSAWNSPIFVIKKK-SGKWRLLQDLRKVNETMMHMGALQPGLP-TPSAIP
031	YP_006732334.1	--
032	YP_009041481.1	FEDDYRKLVSVAGD---IRNVTSAIRVIFLTEDPAQGSTMEQDRAYADLE
033	YP_001036293.1	FNAEFDSLIAAADGREGTQNIISQIRTIFSLEDPFRGSTKIQEDAYRDLE
034	YP_605811.1	FTAEYEEMKNNALGSNGTQNILNQIRMIFFLENPQVGTTNTQDAAYKTLK
035	NP_955577.1	QSPWNTPLLPVKKPGTEDYRPVQDLREVNKRVEDIHPTVPNPYNLLSTLP
036	YP_003208050.1	YTKEYEELVAIADD---PQDITSQVRMVITLEDPYRGSTEEQSRAYLDLE
037	NP_789740.1	ENPYNTPVFAIKKKDSTKWRKLVDFRELNKRTQDFWEVQLGIP-HPAGLK
038	YP_004442839.1	FTTEYEEMRANALGSNGTQNILNQIRMIFFLENPQVGTTNSQDAAYKTLK
039	YP_009507791.1	NNPWNTPIFCIKKK-SGKWRMLIDFRELNKLTLKGAEVQLGLP-HPAGLS
040	YP_004581513.1	--
041	NP_043933.1	--
042	YP_009507248.1	SSPIWVVPKKIDASGKQKWRLVVDFRKLNEKTIDDKYPIPNISDVLDKLG
043	YP_002117531.1	YEQEYEALKTQAVGENGTQNVLSHIRKIFFLEDPKQGTTSSQDAAYRSLK
044	YP_004347415.1	--
045	NP_056891.1	SSPWNTPIFVIKKK-SGKWRLLQDLRAVNATMVLMGALQPGLP-SPVAIP
046	YP_009513242.1	NSTMNTPVYPVPKP-DGKWRMVLDYREVNKTIPLIAAQNQHSAGILSSIY
047	YP_009352866.1	FEKDYKRLVENAGD---IRNVTSAIRVIFLTEDPAQGSTVEQDRAYADLE
048	YP_009165750.1	--
049	NP_040840.1	HWTCNTPIFCIKKK-SGKWRMLIDFRELNKQTEDLAEAQLGLP-HPGGLQ
050	YP_004222728.1	LSCWNTPVFVIRKA-SGSYRLLHDLRAVNAKLVPFGAVQQGAP-VLSALP
051	YP_009506264.1	YEAEYTAMKGQALGNNGTQNILSQIRRIFYLEDPKSGTTLTQDAAYKAIK
052	YP_567050.1	SGPGNNPVFPVKKP-NGKWRFIHDLRATNAITTTLASPSPGPP-DLTSLS
053	YP_009508571.1	NSTMNTPVYPIPKP-DGRWGMVLDYREVNKTIPLIAAQNQHSAGILATIV
054	NP_056803.1	NSTMNTPVYPVPKP-DGRWRMVLDYREVNKTIPLTAAQNQHSAGILATIV
055	NP_955564.1	NSPWNTPVFVIKKK-SGKWRLLQDLRAVNATMHDMGALQPGLP-SPVAVP
056	YP_009508411.1	YPAEYDTLLASADGREGTQNILSQIRTIFTLEDPFRGSTKVQEDAYRDLE
057	YP_009140788.1	YVAEYESLIAIGDGRDGTQNILSQMRRVFSLEDPAQGSTVIQDEAYRELE
058	YP_009508410.1	--
059	NP_040333.1	QSPWNTPLLPVKKPGTNDYRPVQDLREVNKRVEDIHPTVPNPYNLLSGLP
060	YP_008992013.1	QNLQSRIAQLDSELRTMLAQRYHGPEFDQKEREIRHLKAELAQIESDKQK
061	NP_041734.1	YEAEYQQLLTQADGRQGTQNILSQIKRIFSLEDPASGSTRIQDAAYRDLE
062	NP_056895.1	DGPGNNPVFPVRKP-NGTWRFVHDLRATNALTKPIPALSPGPP-DLTAIP
063	YP_009109689.1	RSPWNTPLLPVKKPGTQDYRPVQDLREINKRTVDIHPTVPNPYNLLSTLK
064	YP_002519387.1	--
065	YP_595725.1	FPTAFEAMKTQATGTNGTQNVFSQMKRILLGEVPEQGTTNTQDAAYKRIK
066	YP_009508551.1	NSTMNTPVYPVPKP-DGRWRMVLDYREVNKTIPLTAAQNQHSAGILATIV
067	YP_001497148.1	RSPWNTPLLPVKKPGTRDYRPVQDLREVNKRTMDIHPTVPNPYNLLSTLS
068	NP_041186.1	-------------------------------MMHMGALQPGLP-TPSAIP
069	NP_663784.1	TNPYNTPTFAIKKKDKNKWRMLIDFRELNKVTQDFTEIQLGIP-HPAGLA
070	YP_009508443.1	FPEEYEKLLASADGREGTQNIISQLRTIFTLEDPFRGSTKVQEEAYRDLE
071	YP_003987465.1	YEEEFVSLQTEVLGNNGTQNLLAQIRKVFFLEDPKGGTTVSQDAAYKAIK
072	NP_861410.1	--
073	YP_009345071.1	YETEYQALIGTMEGRSGTQNMLSQIRRIFTLEDPYQGSTMSQDQAYKDLE
074	YP_009508582.1	TSVMNTPVYPVPKP-DGKWRMVLDYRAVNKTIPLIGAQNQHSLGILTNLV
075	NP_569141.1	--
076	YP_002916057.1	--
077	NP_777384.2	DGPGNNPVFPVRKP-NGTWRFVHDLRATNALTKPIPALSPGPP-DLTAIP
078	YP_233110.1	FTAEYEEMKNNALGSNGTQNILNQIRMIFFLENPQVGTTNTQDAAYKTLK
079	NP_542258.1	QGPGNNPIFPVKKP-NGKWRFIHDLRATNSLTRDLASPSPGPP-DLTSLP
080	YP_009130664.1	YPEAFQQMLESADGRQGTQNILSQIYRVFTLEDPYQGSTTMQEEALRDLE
081	YP_009508566.1	NSIMNTPIYPVPKT-EGKWRMVLDYRAVNKTIPLIAAQNQHSAGILTNLV
082	YP_006607892.1	--
083	YP_009506270.1	YTVEYETMKAQALGDQGTQNIINQIRLIFFLENPQVGTTTSQDAAYKTLK
084	NP_042513.1	--
085	YP_006907834.1	--
086	YP_004442827.1	FPTDFEALKNQAIGTNGTQNVISQVRRILLGDSPKQGTTATQDAAYKKIK
087	YP_009408594.1	YPDQYQRLIAIGDD---TQNILSQIRRIITLEDPATGSTAERDQAYLDLE

088 YP_009508561.1 TSPMNTPVYPVPKP-DGKWRMVLDYRAVNKTIPLIAAQNQHSLGILTNLI
089 NP_056907.1 SGPGNNPVFPVKKP-NGKWRFIHDLRATNAITTTLASPSPGPP-DLTSLS
090 YP_002308474.1 QGPGNNPIFPVKKP-NGKWRFIHDLRATNCLTKTLTSPSPGPP-DLTSLP
091 YP_007761644.1 --
092 NP_040550.1 LSCWNTPVFVIRKA-SGSYRLLHDLRAVNAKLVPFGAVQQGAP-VLSALP
093 NP_758808.1 YVSEYDALVQQSDE---TQNLLSQVRRIFLLQDPYQGSTAEQDQAYNDLE
094 YP_009345075.1 FPEEYDTLVEHADD---NINMFSQIRRMFTLEDPYSGSTMAQERAYYDLE
095 NP_068729.1 --
096 NP_047255.1 QSPWNTPLLPVKKPGTEDYRPVQDLREVNKRVEDIHPTVPNPYNLLSTLP
097 YP_006273075.1 YATEYEALVAIGEGRDGTQNILSQMRRVFSLEDPAQGSTVIQDEAYRELE
098 YP_009508888.1 NSIMNTPVYPVPKP-DGRWRMVLDYREVNKTIPLIAAQNQHSAGILASIY
099 NP_395469.1 --
100 YP_009508546.1 NSTMNTPVYPVPKP-DGRWRMVLDYREVNKTIPLTAAQNQHSAGILATIV
101 NP_056762.1 WISKNQAAYNDIKSRGDRPENFVKMVKDRFLIEDPTDERRTALQRLAQRE
102 YP_009508556.1 NSTMNTPVYPVPKP-DGKWRMVLDYREVNKTIPLIAAQNQHSARILSSFF
103 NP_569153.1 YADEYENLVTTADGREGTQNILSQMRRVFSLEDPTTGSTAVQDEAYRDLE
104 NP_043924.1 -STVNSAIWPVGKP-DGSWRLTIDYRPLNSAVSCPYPTVASTPELFAKLE
105 YP_009243641.1 TSPWNSPIFVIRKK-SGKWRLLTDLRAVNASMFPMGALQPGLP-APVMLP
106 YP_004300274.1 --
107 NP_056848.1 --
108 YP_006495799.1 WEDEYRRMVAISDD---TRNLTAAIKRVFGVHDPFTGSTFLQNQAYADLE
109 YP_009508409.1 --
110 NP_687035.1 ENPYNTPVFAIKKKDKTQWRMLVDFRELNKATQDFFEVQLGIP-HPAGLQ
111 NP_045937.2 HSPCNTPIFPIKKAGRDEYRMIHDLRAINNIVAPLTAVVASPTTVLSNLA
112 NP_612577.1 --
113 NP_619548.1 --
114 NP_056880.1 NSPWNTPVFVIKKK-SGKWRLLQDLRAVNATMHDMGALQPGLP-SPVAVP
115 YP_009121747.1 FPEAYEALETMADD---PQNITSQIRQITIMEDPYRGSTEEQDRAYRSLE
116 YP_009113237.2 YQDEYNQLIAKSDGYEGVQNILSQIRRVFTLQDPATGSTKAQDQAYRDIE
117 YP_009508408.1 YPDEYEKILESSDGREGTQNILSQIRTVFTLEDPFRGSTKVQEDAYRDLE
118 NP_955579.1 QSPWNTPLLPVKKPGTEDYRPVQDLREVNKRVEDIHPTVPNPYNLLSTLP
119 YP_009229919.1 YPEDYNSLEAIAEE---TQNVLSQIRKVFLLEVPYQGDLAEQNQAYIDLE
120 YP_004442833.1 YTTEFEILKTQAIGTNGTQNVISQIRRILLGDSPKQGTTLTQDAAYKRIK
121 NP_862833.2 TSAWNSPIFVIKKK-SGKWRLLQDLRKVNETMMHMGALQPGLP-TPSAIP
122 NP_040563.1 DNPYNTPVFVIKKKGTGRWRMLMDFRELNKITVKGQEFSTGLP-YPPGIK
123 YP_443922.1 SSPWNTPILPVPKADGKTYRMVQDFRAVNDVTTGVDLPVPDPHVSLSNLS
124 NP_057933.2 QSPWNTPLLPVKKPGTNDYRPVQDLREVNKRVEDIHPTVPNPYNLLSGLP
125 YP_009116631.1 YEEEYKQLLTMSGD---VRNITAAVKRVFGVHDPHTGSVHIQNQAYAELE
126 YP_009513249.1 ESTMNTPVYPVPKP-NGRWRMVLDYRAVNKVTPLIAVQNQHSYGILGSLF
127 YP_223871.1 HSPWNTPLLPVRKSGTSEYRMVQDLREVNKRVETIHPTVPNPYTLLSLLP
128 NP_127504.1 QDLNKRICPPGTSLFHFFDQ--QKSEMASLKEQIRLLKEQPQKNETDT--
129 NP_044929.1 NSEMNTPVYPVPKA-DGRWRMVLDYREVNKVTPLVATQNCHSASILNTLY
130 NP_057860.1 TGPGNNPVFPVKKA-NGTWRFIHDLRATNSLTIDLSSSSPGPP-DLSSLP
131 NP_040973.1 NNPWNTPVFAIKKK-SGKWRMLIDFRELNKLTEKGAEVQLGLP-HPAGLQ
132 NP_777317.1 FTEAYEELKAQAMGYNGSLNILNQIRLIFFLESPQAGTTDSQDAAFKTLK
133 NP_054716.1 TSPMNTPVYPVPKP-DGRWRMVLDYRAVNKVTPAIATQNCHSASLLNTLY
134 YP_001856242.1 ENPYNTPVFAIKKKDSTKWRKLVDFRELNKRTQDFWEVQLGIP-HPAGLK
135 NP_954565.2 SSPWNTPIFVIKKK-SGKWRLLQDLRAVNATMVLMGALQPGLP-SPVAIP
136 NP_569150.1 YTAEYETMKAQALGDQGTQNIINQIRLIFFLENPQVGTTTSQDAAYKTLK
137 NP_659397.1 --
138 YP_009513211.1 QSPWNTPLLPVKKPGTNDYRPVQDLREVNKRVQDIHPTVPNPYNLLSSLP
139 YP_233107.1 YLPEFEALKAAALGNNGTQNILNQIRMIFFLENPKVGTTDEQDAAYKTIK
140 YP_009109692.1 RSPWNTPLLPVKKPGTQDYRPVQDLREINKRTVDIHPTVPNPYNLLSTLK
141 NP_057849.4 ENPYNTPVFAIKKKDSTKWRKLVDFRELNKRTQDFWEVQLGIP-HPAGLK
142 NP_049560.1 --
143 YP_009506267.1 FPTEYETLLASADGREGTQNILSHIRTIFTLEDPYRGSTKVQEQAYLDLE
144 NP_039820.1 FKDEYQNLAKIANQDGGTQAILSQIRRIFLGEDPVLGQNTVQNIAFRKLK
145 NP_056790.1 RSPWNTPLLPVKKPGTNDYRPVQDLREINKRVQDIHPTVPNPYNLLSSLP
146 YP_002321513.1 YPTDYQQLITVADD---PQNITSMIRRIFTMEDPYTGSTLLQAVAYKDVE
147 NP_955591.1 QSPWNTPLLPVKKPGTNDYRPVQDLREVNKRVEDIHPTVPNPYNLLSGLP
148 YP_001956722.2 NSIMNTPVYPVPKP-DGKWRMVLDYREVNKTIPLIAAQNQHSAGILSSIF
149 NP_056902.1 NNPYNSPIFVIKKR-SGKWRLLQDLRELNKTVQVGTEISRGLP-HPGGLI
150 NP_056886.1 LSCWNTPVFVIRKA-SGSYRLLHDLRAVNAKLVPFGAVQQGAP-VLSALP
151 NP_041261.1 -------------------------------MVPMGALQPGLP-SPVAIP

001 YP_004442836.1 SLVCTEMTDTAIYRYMNDYFHLSAKTGRAWATEE---LSKEFFTKLPRGL
002 NP_056728.1 --
003 YP_009508537.1 K-GRYKTTIDLSNGFWAHPIVPEDYWITAFTWQGK-------QYCWTVLP
004 YP_004732983.2 RLQCKNIE--SVMPFLNSYFQLAAKSGKMWSSPE---LSEKLFRKLPPEI
005 YP_009508407.1 --
006 NP_040939.1 KKK-HVTILDIGDAYFTIPLYEPYREYTCFTLLSPNNLGPCKRYYWKVLP

007	YP_003864102.1	QNY-LKIIIDLKDCFFTIPLHPNDQKRFAFSLPSTNFKEPMKRYQWKVLP
008	YP_009002585.1	RLSCTNIK--DIVSFLNDYARLAAKSGRMFISPE---LSEKLWLKMPYDL
009	YP_003284237.1	SLVCTDMSGIAIKRYMNSYMDLAATSGRMWVSAE---LSDEFFTKLPNGL
010	YP_009268869.1	KKK-HVTILDIGDAYFTIPLYEPYRPYTCFTMLSPNNLGPCTRYYWKVLP
011	YP_009506251.1	RLTCHNIK--DIVQFLNDYGRLAAKSGRLFIGTE---LSEKLWMKMPPEL
012	NP_955611.1	RGW-PLMVLDLKDCFFSIPLAEQDREAFAFTLPSVNNQAPARRFQWKVLP
013	YP_008567619.1	QLTCSNIK--DLVPFLNDYLRLAAKTGRMFLGTE---LSEKLWMKLPGNL
014	NP_149413.1	SLVCTEMTYPALMRYMVGYRNLAARSGRAWANNE---LTNEFFTKLPGKL
015	YP_004442824.1	SLVCTEMTDTAIYRYMNDYFHLAAKSGRAWANEE---LSQEFFTKLPRGL
016	YP_001931967.1	--
017	YP_002455786.1	QALPHLQTIDLTDAFFQIPLPKRFQPYFAFTIPQPLNHGPGSRYAWTVLP
018	YP_009109694.1	PDYSWYTVLDLKDAFFCLPLAPQSQELFAFEWKDPE-RGISGQLTWTRLP
019	NP_789739.1	KKK-SVTVLDVGDAYFSVPLDEDFRKYTAFTIPSINNETPGIRYQYNVLP
020	NP_955619.1	TTLAHLQTIDLKDAFFQIPLPKQFQPYFAFTVPQQCNYGPGTRYAWRVLP
021	YP_224289.1	SLVCHEMTGTAIKRYMADYWTLAAKTGRIWQGPE---LSDEFFTKLPSGL
022	YP_009182100.1	IREISDEKLDEIYATTSDPQYFNYTVLPEMQRRINQGISVRAISQSVNRP
023	NP_705927.1	KKK-SVTVLDVGDAYFSVPLDEDFRKYTAFTIPSINNETPGIRYQYNVLP
024	YP_009508577.1	R-QKYKSTIDLSNGFWAHPITKDSQWITAFTWEGK-------QHVWTRLP
025	YP_004442830.1	SLVCTELSYPAIMRYLVGYFHLSARTGRMWSSKE---LSDEFFTKLPDEI
026	NP_758887.1	QCE-QITVLDIGDAYFSCPLDEDFRKYTAFTIPSVNNQGPGIRYQYNVLP
027	YP_009508406.1	--
028	YP_610965.1	RLSCTNMK--DLLDYMNDYKTLAAKSGRMYVNHE---LSDKFFDKMPQII
029	YP_001931961.1	--
030	YP_241114.1	DKS-YIIVIDLKDCFYTIPLAPQDCKRFAFSVPSVNFKEPMKRYQWKVLP
031	YP_006732334.1	--
032	YP_009041481.1	RLPTPEMK--NIFQFLNQYKKLAAASGRMWITPE---LSEKLFRKLPPVI
033	YP_001036293.1	RISCKDVQ--DIIPFMNEYMRIAAKTGRLFISSE---LSEKFWMKLPGDL
034	YP_605811.1	QLVCTEMSGPAIYRYLNDYFHLAAKSGRAWASDE---LSKEFFTKLPKGL
035	NP_955577.1	PSHPWYTVLDLKDAFFCLRLHSESQLLFAFEWRDPE-IGLSGQLTWTRLP
036	YP_003208050.1	RLVCDDIK--DILPYLNDFKVLAAKSGRMFTSPE---LSEKLFRKLPPLI
037	NP_789740.1	KKK-SVTVLDVGDAYFSVPLDEDFRKYTAFTIPSINNETPGIRYQYNVLP
038	YP_004442839.1	QLVCTEMSGPAIYRYLNDYFHLSAKSGRAWASEE---LSKEFFTKLPKGL
039	YP_009507791.1	MRK-QVTVLDIGDAYFTIPLDPDYQPYTAFTLPNKNNQGPGRRYVWCSLP
040	YP_004581513.1	--
041	NP_043933.1	--
042	YP_009507248.1	KCQ-YFTTLDLASGFYQVEMDPQDISKTAFNVEHG-------HFEFLRMP
043	YP_002117531.1	SLTCMEMTGTAIRRYMASYFDLAAKSGRMWSSEE---LSKEFFQKLPGRL
044	YP_004347415.1	--
045	NP_056891.1	QGY-LKIIIDLKDCFFSIPLHPSDQKRFAFSLPSTNFKEPMQRFQWKVLP
046	YP_009513242.1	R-GKYKTTLDLTNGFWAHPITPESYWLTAFTWQGK-------QYCWTRLP
047	YP_009352866.1	RLPTPEMK--NIFQFLNQYKKLAAASGKMWITSE---LSEKLFRKLPPII
048	YP_009165750.1	--
049	NP_040840.1	RKK-HVTILDIGDAYFTIPLYEPYRQYTCFTMLSPNNLGPCVRYYWKVLP
050	YP_004222728.1	RGW-PLMVLDLKDCFFSIPLAEQDREAFAFTLPSVNNQAPARRFQWKVLP
051	YP_009506264.1	SLVCNDENGEAVKRYMLSYFDLAARSGRMWTSQE---LSEEFFTKLPDGL
052	YP_567050.1	TALPYLQTIDLTDAFFQIPLPKQFQPYFAFTIPQPCNYGPGARYAWTVLP
053	YP_009508571.1	R-KKYKTTLVLANGFWAHPITPESYWLTAFIWQGK-------QYCWTRLP
054	NP_056803.1	R-QKYKTTLDLANGFWAHPITPDSYWLTAFTWQGK-------QYCWTRLP
055	NP_955564.1	KGW-EIIIIDLQDCFFNIKLHPEDCKRFAFSVPSPNFKRPYQRFQWKVLP
056	YP_009508411.1	RISCSDMK--NIIAFMNEYMRLASKSGRLFVSPE---LSEKFWLKLPGDL
057	YP_009140788.1	RLSCTDVK--YIIPFLNEYLRLAAKSGRIFLGGE---LSEKIWMKMPGDL
058	YP_009508410.1	--
059	NP_040333.1	PSHQWYTVLDLKDAFFCLRLHPTSQSLFAFEWRDPE-MGISGQLTWTRLP
060	YP_008992013.1	PSLFTSSPPIPSIGPTYHPFASMLSLIRQYEPSKLFGMTHTLFRDNPLPP
061	NP_041734.1	RLTCHNIK--DIVQFLNDYGRLAAKSGRLFLGTE---LSEKLWMKMPPEL
062	NP_056895.1	THLPHIICLDLKDAFFQIPVEDRFRSYFAFTLPTPGGLQPHRRFAWRVLP
063	YP_009109689.1	PDYSWYTVLDLKDAFFCLPLAPQSQELFAFEWKDPE-RGISGQLTWTRLP
064	YP_002519387.1	--
065	YP_595725.1	SLVCQEMTYPAIMRYLVGYRNLAARSGRAWVNNE---LTDEFFTKLPGKL
066	YP_009508551.1	R-QKYKTTLDLANGFWAHPITPESYWLTAFTWQGK-------QYCWTRLP
067	YP_001497148.1	PDRTWYTVLDLKDAFFCLPLAPQSQELFAFEWRDPE-RGISGQLTWTRLP
068	NP_041186.1	DKS-YIIVIDLKDCFYTIPLAPQDCKRFAFSLPSVNFKEPMQRYQWRVLP
069	NP_663784.1	KKK-RISILDVGDAYFSIPLHEDFRQYTAFTLPAVNNMEPGKRYIYKVLP
070	YP_009508443.1	RISCRDVK--DLIPFMNEYMRIASKTGRLFISSE---LSEKFWFKLPGDL
071	YP_003987465.1	SLTCQEMSGQAIRKYMSSYFDLAARSGRMWVNEE---LSDDFFTKLPMGL
072	NP_861410.1	--
073	YP_009345071.1	RLTCTDVK--HILEYLRQFMYLAAKTGRLFTSSE---LSSKLFVKMPGDL
074	YP_009508582.1	R-QKYKSTIDLSNGFWAHPITKDSQWITAFTWEGK-------QHVWTRLP
075	NP_569141.1	--
076	YP_002916057.1	--
077	NP_777384.2	THLPHIICLDLKDAFFQIPVEDRFRSYFAFTLPTPGGLQPHRRFAWRVLP
078	YP_233110.1	QLVCTEMSGPAIYRYLNDYFHLAAKSGRAWASDE---LSKEFFTKLPRGL
079	NP_542258.1	QDLPHLRTIDLTDAFFQIPLPAVFQPYFAFTLPQPNNHGPGTRYSWRVLP

080 YP_009130664.1 KLSCHNIK--DIVAYMNEYMRLACKSRRLFINAD---LSEKFWFKMPGEL
081 YP_009508566.1 R-QKYKSTIDLSNGFWAHPIDQDSQWITAFTWEGK-------QYVWTRLP
082 YP_006607892.1 --
083 YP_009506270.1 SLVCTEMTDTAIYRYMNDYFHLSAKTGRAWASEE---LSKEFFTKLPRGL
084 NP_042513.1 --
085 YP_006907834.1 --
086 YP_004442827.1 SLVCTELSYPAIMRYMVGYFHLASKSGRMWTSNE---LSDEFFTKLPDEI
087 YP_009408594.1 RLSCEHMK--DLIPYMNRYKVLSAKTGRLFINSE---LSDKFFRKMPTLI
088 YP_009508561.1 R-HKYKSTIDLSNGFWAHPITEDSQWITAFTWEGK-------QHVWTRLP
089 NP_056907.1 TALPYLQTIDLTDAFFQIPLPKQFQPYFAFTIPQPCNYGPGARYAWTVLP
090 YP_002308474.1 QGLPHLRTIDLTDAFFQIPLPVAFQPYFAFTLPQPNNHGPGARYSWKVLP
091 YP_007761644.1 --
092 NP_040550.1 RGW-PLMVLDLKDCFFSIPLAEQDREAFAFTLPSVNNQAPARRFQWKVLP
093 NP_758808.1 RISCDNIK--DLIPYLIQFRNLAAKSGRLFLGPE---LSEKLFRKMPPLI
094 YP_009345075.1 RLTCSNIN--NVIQFMNEYKKLAAASGRAFISTE---LSEKFFRKLPPLY
095 NP_068729.1 --
096 NP_047255.1 PSHPWYTVLDLKDAFFCLRLHSESQLLFAFEWRDPE-IGLSGQLTWTRLP
097 YP_006273075.1 RMSCTDVK--YIIPFLNEYMRLAAKTGRLFLGGE---LSEKIWMKMPGDL
098 YP_009508888.1 R-GTYKTTLDLANGFWAHPITPNSYWLTAFTWQGK-------QHCWTRLP
099 NP_395469.1 --
100 YP_009508546.1 R-QKYKTTLDLANGFWAHPITPESYWLTAFTWQGK-------QYCWTRLP
101 NP_056762.1 LEALNCEDPTKIQPFMAEYLKKASEAKKGFDVVY----VERLFDRLPEAV
102 YP_009508556.1 S-GKYKTTLDLTNGFWAHPITPESYWLTAFTWRGK-------QYCRTKLP
103 NP_569153.1 RLTCDSVK--HIVQYLNDFMRIAAKTGRMFIGPE---LSEKLWLKMPGDL
104 NP_043924.1 KKYQVYSSLDISNGFWSIRLEEECQYLFAFTFDT-------QQYTWTRLP
105 YP_009243641.1 RDW-PLIIIDLLDCFFTIPIHPQDKQRFAFSIPKINNAGPHTRYQWRVLP
106 YP_004300274.1 --
107 NP_056848.1 --
108 YP_006495799.1 RLSCKRME--DVMPFLFTYYQLAAKSGRMWTNEE---LSDKLFRKLPEAV
109 YP_009508409.1 --
110 NP_687035.1 KKK-QITVIDIGDAYYSIPLCKEFRKYTAFTIPSVNNTGPGIRYQFNCLP
111 NP_045937.2 PSLHWFTVIDLSNAFFSVPIHKDSQYLFAFTFEG-------HQYTWTVLP
112 NP_612577.1 --
113 NP_619548.1 --
114 NP_056880.1 KGW-EIIIIDLQDCFFNIKLHPEDCKRFAFSVPSPNFKRPYQRFQWKVLP
115 YP_009121747.1 RITCEDTR--DLWKFLNEFRILASKSGRLFF-PA---TTDKFFTKLPMTL
116 YP_009113237.2 KLNCHDVK--DIVSFLNDYLRLAAKTGRMYIGME---LSEKLWMKMPGEL
117 YP_009508408.1 RISCKDMK--DIIPFMNEYMRLASKTGRLFISSE---LSEKFWMKLPGDL
118 NP_955579.1 PSHPWYTVLDLKDAFFCLRLHSESQLLFAFEWRDPE-IGLSGQLTWTRLP
119 YP_009229919.1 RLTCESTR--DIWRYLNDFKTLAAKSGRIYF-PE---TSEKLFRKMPPLI
120 YP_004442833.1 SLVCDKETYPDIMRYMVGYLHLAARTGRMWISKE---LSDEFFLKLPSEI
121 NP_862833.2 DRS-YIIVIDLKDCFYTIPLAPQDCKRFAFSLPSVNFKEPMQRYQWKVLP
122 NP_040563.1 ECE-HLTAIDIKDAYFTIPLHEDFRPFTAFSVVPVNREGPIERFQWNVLP
123 YP_443922.1 PKHQYFTVVDLANAFFSIPLHVDSQPLFAFTYDNQ-------QYTYSVLP
124 NP_057933.2 PSHQWYTVLDLKDAFFCLRLHPTSQPLFAFEWRDPE-MGISGQLTWTRLP
125 YP_009116631.1 RLYCKRTD--DVIPFLYDYYQLAAKSGRMWLGPE---LSEKLFRKLPPEI
126 YP_009513249.1 K-GRYKTTIDLSNGFWAHPIVPEDYWITAFTWQGK-------QYCWTVLP
127 YP_223871.1 PDRIWYSVLDLKDAFFCIPLAPESQLIFAFEWADAE-EGESGQLTWTRLP
128 NP_127504.1 PSYQSSYQPFHSFSSPYMPSNPPNSPFTTFANTPQP--QPSLFSQYPIQP
129 NP_044929.1 R-GPYKSTLDLANGFWAHPIKPEDYWITAFTWGGK-------TYCWTVLP
130 NP_057860.1 TTLAHLQTIDLKDAFFQIPLPKQFQPYFAFTVPQQCNYGPGTRYAWRVLP
131 NP_040973.1 IKK-QVTVLDIGDAYFTIPLDPDYAPYTAFTLPRKNNAGPGRRFVWCSLP
132 NP_777317.1 SLVCTEMTDTSLYRYMNDYFHLAAKSGRAWANED---LSKEFFTKLPRGL
133 NP_054716.1 R-GQYKTTLDLANGFWAHPIQESDQWITSFTWNGK-------SYVWTTLP
134 YP_001856242.1 KKK-SVTVLDVGDAYFSVPLDEDFRKYTAFTIPSINNETPGIRYQYNVLP
135 NP_954565.2 QGY-LKIIIDLKDCFFSIPLHPSDQKRFAFSLPSTNFKEPMQRFQWKVLP
136 NP_569150.1 SLVCTEMTDTAIYRYMNDYFHLSAKTGRAWASEE---LSKEFFTKLPRGL
137 NP_659397.1 --
138 YP_009513211.1 PSHTWYSVLDLKDAFFCLKLHPNSQPLFAFEWRDPE-KGNTGQLTWTRLP
139 YP_233107.1 SLVCNEMTDKAVYRYMNDYFHLASKSGRMWANEE---LSTEFFTKLPRHL
140 YP_009109692.1 PDYSWYTVLDLKDAFFCLPLAPQSQELFAFEWKDPE-RGISGQLTWTRLP
141 NP_057849.4 KKK-SVTVLDVGDAYFSVPLDEDFRKYTAFTIPSINNETPGIRYQYNVLP
142 NP_049560.1 --
143 YP_009506267.1 RISCRDVK--DLIPFLNEYMRLAAKTGRLFISTE---LSDKIWMKLPGDL
144 NP_039820.1 QLVCPNY--QSIRRYLMDYLTLAAETGLMWSETEGPAISEELFTKMPAAI
145 NP_056790.1 PSYTWYSVLDLKDAFFCLRLHPNSQPLFAFEWKDPE-KGNTGQLTWTRLP
146 YP_002321513.1 RLQCDRIE--HIVPFMMEYFKISAETGRLYVDNE---MSDKFFRKLPPLY
147 NP_955591.1 PSHQWYTVLDLKDAFFCLRLHPTSQPLFAFEWRDPE-MGISGQLTWTRLP
148 YP_001956722.2 R-GKYKTTLDLSNGFWAHSITPESYWLTAFTWLGQ-------QYCWTRLP
149 NP_056902.1 KCK-HMTVLDIGDAYFTIPLDPEFRPYTAFTIPSINHQEPDKRYVWKCLP
150 NP_056886.1 RGW-PLMVLDLKDCFFSIPLAEQDREAFAFTLPSVNNQAPARRFQWKVLP
151 NP_041261.1 LNY-HKIVIDLKDCFFTIPLHPEDRPYFAFSVPQINFQSPMPRYQWKVLP

```
001 YP_004442836.1  GDEVEKAFKEKHPSNTIGVTARITFTK--RYLKELCEKVALQKSIGKMDF
002 NP_056728.1     --------------------------------------------------
003 YP_009508537.1  QGFLNSPGLFTGDVVDLLQGI--------PNVEVYVDDVYISHDSEKEHL
004 YP_004732983.2  GPTIAKEYAERYPGMLIGVNARIQFVS--EYLQDLCKQADLQRKLKNLNF
005 YP_009508407.1  --------------------------------------------------
006 NP_040939.1     QGWKLSPSVYQFTMQEILEDWIQQHPE--IQFGIYMDDIYIGSDLEIKKH
007 YP_003864102.1  QGMANSPTLCQKFVAMAIQTVRDTWKQ--IYIIHYMDDILLAGADGQQVL
008 YP_009002585.1  GNKIRTAYEAKHEGNTIGVFPRILFTY--KYLEQECKEAAFRRSLKNLSF
009 YP_003284237.1  GDRVAKAFKEKHPGNTVGVPARVTFTQ--NYLEEVCREAAYQRSLKNLDF
010 YP_009268869.1  QGWKLSPSVYQFTMQEILRDWIAKHPM--IQFGIYMDDIYIGSDLDIMKH
011 YP_009506251.1  GHRMREAFQKEYEGNEVGVFPRILFAY--RYLEQECKDAAFKRSLKSLSF
012 NP_955611.1     QGMTCSPTICQLVVGQVLEPLRLKHPS--LCMLHYMDDLLLAASSHDGLE
013 YP_008567619.1  GRRIKEAFDNKYKGNEAGVIPRILFTH--KYLEDQCKEAAFQRSLKGMTF
014 NP_149413.1     GDKVKEAFVAKYPSVERHVPAAIRFTY--DYLEEICTENNFQKQLRSLNF
015 YP_004442824.1  GDRIEKAFKEKHPSNTIGVAARITFTK--NYMKEICQEAVFQNQLKKLGF
016 YP_001931967.1  --------------------------------------------------
017 YP_002455786.1  QGFKNSPTLFEQQLASVLGPARKAFPT--SVIVQYMDDILLACPSQHELD
018 YP_009109694.1  QGFKNSPTLFDEALHRDLTDFRTQHPE--VTLLQYVDDLLLAAPTKKACT
019 NP_789739.1     QGWKGSPAIFQSSMTKILEPFRKQNPD--IVIYQYMDDLYVGSDLEIGQH
020 NP_955619.1     QGFKNSPTLFEMQLAHILQPIRQAFPQ--CTILQYMDDILLASPSHADLQ
021 YP_224289.1     GDRVAKAFKEKYPGNTVGVPARITFTQ--LYLEELCREAAYQRSLKNLDF
022 YP_009182100.1  STSGDSKEVPITSEVPSSSTLEGKGKT--KKFTPAGSSIKLPLITGVPTG
023 NP_705927.1     QGWKGSPAIFQSSMTKILEPFRKQNPD--IVIYQYMDDLYVGSDLEIGQH
024 YP_009508577.1  QGFLNSPALFTADVVDLLKNI--------PGISVYVDDIYFSTETVSEHL
025 YP_004442830.1  SDSAKEAFDKKFPGTNIHVPARIEFTY--NYLEEICTENNKQRRLGKLNF
026 NP_758887.1     QGWKGSPAIFQATADKILQPFRERHPD--VVIYQYMDDLFVGSDRVAPEH
027 YP_009508406.1  --------------------------------------------------
028 YP_610965.1     GPDMKKAFSEKYPGAQMGVLPRITFAY--KYLSDICKQAAVQKGVKDLAF
029 YP_001931961.1  --------------------------------------------------
030 YP_241114.1     QGMTNSPTLCQKLVATAIAPVRQRFPQ--IYLVHYMDDILLAHTDEHLLY
031 YP_006732334.1  --------------------------------------------------
032 YP_009041481.1  GPAIEKAYKQRYPGMDIGVPTRINFIY--QYLMEVCKQAALQRSLKDLSF
033 YP_001036293.1  GKTIKDSFDKTYVGNTVGVHPRILFTY--KYLQEECKKAALSRSLKNLQF
034 YP_605811.1     GDRVEKEFKKRYPNNTIGVAPRITFTR--NYMKEICQEAVFQSQLKKLDF
035 NP_955577.1     QGFKNSPTLFDEALHSDLADFRVRYPA--LVLLQYVDDLLLAAATRTECL
036 YP_003208050.1  GGEIEAAFKEKYPGNTVGVLPRIVFIH--HYLTDLCKKAAIQRGIKDLSF
037 NP_789740.1     QGWKGSPAIFQSSMTKILEPFRKQNPD--IVIYQYMDDLYVGSDLEIGQH
038 YP_004442839.1  GDRVEKSFKEKYPNNTIGVAARITFTR--AYMKEICQEAVFQNQLKRLDF
039 YP_009507791.1  QGWVLSPLIYQSTLDNILQPWRKKYPN--IDVYQYMDDIYIGSDFSRLEH
040 YP_004581513.1  --------------------------------------------------
041 NP_043933.1     --------------------------------------------------
042 YP_009507248.1  MGLKNSPSTFQRVMDNVLRGLQN------NICLVYIDDIIVYSTSLQEHL
043 YP_002117531.1  GERVEQAFNAKFQGNTIGVVPRITFTQ--RYLTEICEEASYQRSLKNLNF
044 YP_004347415.1  --------------------------------------------------
045 NP_056891.1     QGMANSPTLCQKYVATAIHKVRHAWKQ--MYIIHYMDDILIAGKDGQQVL
046 YP_009513242.1  QGFLNSPALFTADVVDLLKEV--------PNVQAYVDDIYMSHDDPQEHL
047 YP_009352866.1  GPAVETAFKLRYPGLDIGVPTRINFIY--HYLMEVCKQAALQRSMEDLSF
048 YP_009165750.1  --------------------------------------------------
049 NP_040840.1     QGWKLSPAVYQFTMQKILRGWIEEHPM--IQFGIYMDDIYIGSDLGLEEH
050 YP_004222728.1  QGMTCSPTICQLIVGQVLEPLRLKHPS--LCMLHYMDDLLLAASSHDRLE
051 YP_009506264.1  RDRAAEAFKKKFPGNTIGVPARITFLQ--NYLEEACKEDAYQRSIKNLNF
052 YP_567050.1     QGFKNSPTLFEQQLAAVLSPIRKTFPM--STIIQYMDDILLASPTQEELQ
053 YP_009508571.1  QGFLNSPALFTADVVDLLKEI--------SNVQAYVDDIYLSHDDPQEHL
054 NP_056803.1     QGFLNSPALFTADAVDLLKEV--------PNVQVYVDDIYLSHDNPHEHI
055 NP_955564.1     QGMKNSPTLCQKFVDKAILTVRDKYQD--SYIVHYMDDILLAHPSRSIVD
056 YP_009508411.1  GKRIKEKFDQAYAGNNVGVHPRILFTY--KFLEEECKAAAFSRSLKGLQF
057 YP_009140788.1  GKRIKEEFDRKYPGVISGVVPRVLFAY--KFLENECKEAAFKRSLKNLSF
058 YP_009508410.1  --------------------------------------------------
059 NP_040333.1     QGFKNSPTLFDEALHRDLADFRIQHPD--LILLQYVDDLLLAATSELDCQ
060 YP_008992013.1  --PPKSKPKPKPQPRPVTINPSSTIIP--YQQSPGYTPASPSPPEPSKSQ
061 NP_041734.1     GHRMKEAFQKEYSGNEVGVFPRILFAY--RYLEQECKDAAFKRSLKSLSF
062 NP_056895.1     QGFINSPALFERALQEPLRQVSAAFSQ--SLLVSYMDDILIASPTEEQRS
063 YP_009109689.1  QGFKNSPTLFDEALHRDLTDFRTQHPE--VTLLQYVDDLLLAAPTKKACT
064 YP_002519387.1  --------------------------------------------------
065 YP_595725.1     GDRVKEAFKKKYPGVERHVPAATRFTY--DYLEEICTENNFQKQLRSLNF
066 YP_009508551.1  QGFLNSPALFTADVVDLLKEI--------PNVQVYVDDIYLSHDDPKEHV
067 YP_001497148.1  QGFKNSPTLFDEALHRDLTDFRTQHPE--VTLLQYVDDLLLAAPTEEACT
068 NP_041186.1     QGMTNSPTLCQKFVATAIAPVRQRFPQ--LYLVHYMDDILLAHTDEHLLY
069 NP_663784.1     QGWKGSPAIFQYTMRQVLEPFRKANPD--VILIQYMDDILIASDRTGLEH
070 YP_009508443.1  GKRVKEAFDQTYSGNTVGVHPRILFTY--KFLEEECKKAAFARSLKGLQF
```

071	YP_003987465.1	GDRVRQAFLSRYPGNTIGVPARITFTQ--QYLEEVCREAAYQRSLKTLDF
072	NP_861410.1	--
073	YP_009345071.1	GKRIEDSFKAKYPGNTVGVFPRIVFSF--KYLENQCREASFQRSLKSLEF
074	YP_009508582.1	QGFLNSPALFTADVVDLLKDI--------PGISVYVDDIYFSTETVSEHL
075	NP_569141.1	--
076	YP_002916057.1	--
077	NP_777384.2	QGFINSPALFERALQEPLRQVSAAFSQ--SLLVSYMDDILIASPTEEQRS
078	YP_233110.1	GDRVEKKFKEKYPNNTIGVAPRITFTR--NYIKEICQEAVFQSQLKRLDF
079	NP_542258.1	QGFKNSPTLFEQQLSHILAPVRKAFPN--SLIIQYMDDILLASPALRELT
080	YP_009130664.1	GPRIKEAYEARYPGNTIGVFPRVLFAY--KFLENECRDAAYKRSLKNLSF
081	YP_009508566.1	QGFLNSPALFTADVVDLLKEI--------PNVNVYVDDIYVSTETINQHF
082	YP_006607892.1	--
083	YP_009506270.1	GDAVEKAFKEKHPSNTVGIAARITFTK--RYLKELCEKVALQKSIGKMDF
084	NP_042513.1	--
085	YP_006907834.1	--
086	YP_004442827.1	ADSAKTAFNKKFPGTAIHIPARIEFTY--NYLEEICTENNKQRKLGKLNF
087	YP_009408594.1	GKHLEQKYNAAFPGNTIGVFPRIHFAY--QELAEMCKQAQLQRGLKDLAF
088	YP_009508561.1	QGFLNSPALFTADVVDILKEV--------PGVSVYVDDIYISSPTMEEHF
089	NP_056907.1	QGFKNSPTLFEQQLAAVLSPIRKTFPM--STIIQYMDDILLASPTQEELQ
090	YP_002308474.1	QGFKNSPTLFEQQLSHILTPVRQAFPK--SIVIQYMDDILLASPTLEESI
091	YP_007761644.1	--
092	NP_040550.1	QGMTCSPTICQLVVGQVLEPLRLKHPS--LRMLHYMDDLLLAASSHDGLE
093	NP_758808.1	GKEIETAFIAKHGNANITVMPRIHFAY--HYLAELCKKAALQRSLKDLSF
094	YP_009345075.1	GKKIEAAFNEKHPGNNTGVMPRIHFTY--HWLAELCKSAAMQRGIKSLDF
095	NP_068729.1	--
096	NP_047255.1	QGFKNSPTLFDEALHSDLADFRVRYPA--LVLLQYVDDLLLAAATRTECL
097	YP_006273075.1	RKTIKEDFDNKYPGVVSGVVPRVLFAY--KFLEKECRDAAFKRSLKNLSF
098	YP_009508888.1	QGFLNSPALFTADVVDLMKHI--------PNVQVYVDDLYLSHDDPQEHL
099	NP_395469.1	--
100	YP_009508546.1	QGFLNSPALFTADVVDLLKEI--------PNVQVYVDDIYLSHDDPQEHI
101	NP_056762.1	GKVVKADFVKDGNSYEAGIGIAVSYIS--TWMRAKCIKETEAKTQKKASL
102	YP_009508556.1	QGFLNSPALFTAHVVDLLKEI--------PHVPAYVDDIYISHDDPHEHL
103	NP_569153.1	GQRMKKAYEEKHPGNIVGVCPRILFAY--KYLEGECKDAAFRRSLKNLSF
104	NP_043924.1	QGFHASPGIFHQALYNGLASCKTAIESQGCKLLQYVDDILLMSEDRDHHL
105	YP_009243641.1	QGMMNSPTICQLYVDAALKPIRKRYPF--LHIYHYMDDILFAGPKKEFLQ
106	YP_004300274.1	--
107	NP_056848.1	--
108	YP_006495799.1	GPTIEKAYKERYPGLVIGVMARINFII--EYLQNVCKQAALQRSLKNLNF
109	YP_009508409.1	--
110	NP_687035.1	QGWKGSPTIFQNTAANILEEIKRHTPG--LEIVQYMDDLWLASDHDETRH
111	NP_045937.2	QGFIHSPTLFSQALYQSLHKIKFKIS---SEICIYMDDVLIASKDRDTNL
112	NP_612577.1	--
113	NP_619548.1	--
114	NP_056880.1	QGMKNSPTLCQKFVDKAILTVRDKYQD--SYIVHYMDDILLAHPSRSIVD
115	YP_009121747.1	SKKIEEAFRNKYPGLTAGVLPAIKFTH--TYITEMCTEAALQKELRDLSL
116	YP_009113237.2	GVRIKEAFDREY-GEIIGVAPRVMFAY--GFLERQCREAAFARSLKGLDF
117	YP_009508408.1	GKRVKELYEASYSGNTIGVHPRILFTY--KFLEEECKKAAFSRSLKGLQF
118	NP_955579.1	QGFKNSPTLFDEALHSDLADFRVRYPA--LVLLQYVDDLLLAAATRTECL
119	YP_009229919.1	GEEVEKAWNEKYPGAAAAVIPRIYFTY--QYLSKMCKQAALQREIKDLSF
120	YP_004442833.1	GDQARKAFDAKFPGTHIHVPARIDFTY--NYLEEVCTENNRQRKLAKLDF
121	NP_862833.2	QGMTNSPTLCQKFVAAAIAPVRQCFPQ--LYLVHYMDDILLAHINEHLLY
122	NP_040563.1	QGWVCSPAIYQTTTQKIIENIKKSHPD--VMLYQYMDDLLIGSNRD--DH
123	YP_443922.1	QGYRCSPGIFNRVLKDHLKEIVIPD-G--VVLIQYVDDLLLAACSSDQCL
124	NP_057933.2	QGFKNSPTLFDEALHRDLADFRIQHPD--LILLQYVDDLLLAATSELDCQ
125	YP_009116631.1	GPTIEQAYKDRYPGLTIGVLARANFIL--EYLQNVCKQAALQRSLKSLSF
126	YP_009513249.1	QGFLNSPGLFTGDVVDLLQGI--------PNVEVYVDDVYISHDSEKEHL
127	YP_223871.1	QGFKNSPTLFDVALNRDLQGFRLDHPS--VSLLQYVDDLLIAADTQAACL
128	NP_127504.1	KSPNTFDLAKLVWEKKDAIAAEKRAKK--KLQKDEVKQKTSLPPESKRPD
129	NP_044929.1	QGFLNSPALFTADVVDILKDI--------PNVQVYVDDVYVSSATEQEHL
130	NP_057860.1	QGFKNSPTLFEMQLAHILQPIRQAFPQ--CTILQYMDDILLASPSHADLQ
131	NP_040973.1	QGWILSPLIYQSTLDNIIQPFIRQNPQ--LDIYQYMDDIYIGSNLSKKEH
132	NP_777317.1	GDRIEKAFTEKHPTNTIGVAARIAFTR--NYLREICQEAVFQSSLKRLGF
133	NP_054716.1	QGFLNSPALFTADVVDLLKDI--------PNVEVYVDDVYFSNDTEEEHL
134	YP_001856242.1	QGWKGSPAIFQSSMTKILEPFRKQNPD--IVIYQYMDDLYVGSDLEIGQH
135	NP_954565.2	QGMANSPTLCQKYVATAIHKVRHAWKQ--MYIIHYMDDILIAGKDGQQVL
136	NP_569150.1	GDEVEKAFMEKHPSNTVGITARITFTK--RYLKELCEKVALQKSIGKMDF
137	NP_659397.1	--
138	YP_009513211.1	QGFKNSPTLFDEALHRDLASFRALNPQ--VVMLQYVDDLLVAAPTYRDCK
139	YP_233107.1	GDKVEKAFKERHPTNSIGVTARIAFTR--NYLKDMCQEALFQSQLKKMNF
140	YP_009109692.1	QGFKNSPTLFDEALHRDLTDFRTQHPE--VTLLQYVDDLLLAAPTKKACT
141	NP_057849.4	QGWKGSPAIFQSSMTKILEPFRKQNPD--IVIYQYMDDLYVGSDLEIGQH
142	NP_049560.1	-----------MQLAHILQPIRQAFSQ--CTILQYMDDILLASPSHKDLQ
143	YP_009506267.1	GKRIKTKFEEQYAGNTIGVHPRIMFIY--KFLEEECKNAAFMRSMKNLQF

144	NP_039820.1	GERVAQAYKIMDPTSAVNLPSRVYFTI--NYLTEQCK[illegible]ASYMRSLKALDF
145	NP_056790.1	QGFKNSPTLFDEALHRDLAPFRALNPQ--VVLLQYVD[illegible]LLVAAPTYEDCK
146	YP_002321513.1	GEDIEKNYKQVH-GDLPGVAPRIYFTY--KYLQEMCK[illegible]AEKQRGLKSLNF
147	<u>NP_955591.1</u>	QGFKNSPTLFDEALHRDLADFRIQHPD--LILLQYVD[illegible]LLLAATSELDCQ
148	YP_001956722.2	QGFLNSPALFTADVVDLLKEV--------PNVQVYVD[illegible]IYISHDDPREHL
149	NP_056902.1	QGFVLSPYIYQKTLQEILQPFRERYPE--VQLYQYMD[illegible]LFMGSNGSKKQH
150	NP_056886.1	QGMTCSPTICQLVVGQVLEPLRLKHPS--LCMLHYMD[illegible]LLLAASSHDGLE
151	NP_041261.1	QGMANSPTLCQKFVAAAIAPVRSQWPE--AYILHYMD[illegible]ILLACDSAEAAK

001	YP_004442836.1	CRN---TPVHGLYGRERSH--KRYGARKSTSYKGK[illegible]HK-SHVRIGKKKHL
002	NP_056728.1	--
003	YP_009508537.1	EYL---EILFNRLNEAGYIVSLKKSNIANSIVDFL[illegible]FQITNEGRGLTDTF
004	YP_004732983.2	CKA---IPIPGYYDQGV---KKKYGLRKSKTYKGK[illegible]HD-SHVKVIKNKYK
005	YP_009508407.1	--
006	NP_040939.1	REI---VKDLANYIAQYGFTLPEEKRQKGYPAKWL[illegible]FELHPQTWKFQKHT
007	YP_003864102.1	QCF---AQLKEKLVIAGLHIAPEKIQLHD-PYTYL[illegible]FQLNGPKITTQKA-
008	YP_009002585.1	CSE---MPLPGYYGEK-----KRYGVRRSTTYKGK[illegible]HP-THARIEKQKHL
009	YP_003284237.1	CKE---FPVPGYY-KKPGY---KYGRRKTTTYKGK[illegible]HK-THVRIDKRKHL
010	YP_009268869.1	REI---VEELASYIAQYGFMLPEEKRQEGYPAKWL[illegible]FELHPEKWRFQKHT
011	YP_009506251.1	CKD---MPLTGYYDKS-----SRYGMRRSKTYKGK[illegible]HA-SHARIEKRKHL
012	NP_955611.1	AAG---EEVISTLERAGFTISPDKVQREP-GVQYL[illegible]YKLG-STYVAPVG-
013	YP_008567619.1	CNS---IPIPGYYS-KE----KKMGVRRSTTYKGK[illegible]HS-SHVRIEKRKHL
014	NP_149413.1	CKG---FPVVNPVGTKKYG--KRYGTRKARSYRGK[illegible]HS-SHVRIERTRYL
015	YP_004442824.1	CRN---TPVHGLYGKDKPY--KKYGARKSQNYHGK[illegible]HK-SHVRIAKRKHL
016	YP_001931967.1	--
017	YP_002455786.1	QLA---TLTAQLLSSHGLPVSQEKTQRTPGKIHFL[illegible]QIIHPDHITYETTP
018	YP_009109694.1	QGT---RHLLQELGEKGYRASAKKAQICQTKVTYL[illegible]YILSEGKRWLTPGR
019	NP_789739.1	RTK---IEELRQHLLRWGLTTPDKKHQKEPPFLWM[illegible]YELHPDKWTVQPIV
020	NP_955619.1	LLS---EATMASLISHGLPVSENKTQQTPGTIKFL[illegible]QIISPNHLTYDAVP
021	YP_224289.1	CRE---FPIPGYY-KKPGR---KFGVRKSTSYKGK[illegible]HK-THIKIDKRKYL
022	YP_009182100.1	KPN---EFNTERYKPRIEPNPAVSPNGVWLDIDNN[illegible]DPRGNIDEWVNTMN
023	NP_705927.1	RTK---IEELRQHLLRWGLTTPDKKHQKEPPFLWM[illegible]YELHPDKWTVQPIV
024	YP_009508577.1	KIL---EKVFKILLEAGYIVSLKKSALLRYEVTFL[illegible]FSITQTGRGLTSEF
025	YP_004442830.1	CRG---FPVINPL-SKKY---KSLGTRRTTTYKGK[illegible]HH-THARIDRRKYL
026	NP_758887.1	SRM---IQELRDHLLFWGLETPDKKFQKEPPFEWM[illegible]YILHPKKWTVQKVQ
027	YP_009508406.1	--
028	YP_610965.1	CRR---IPLPGYYKDGP---KKKLGLRKAKNYRGK[illegible]HD-THVRLVKNK--
029	YP_001931961.1	--
030	YP_241114.1	QAF---SILKQHLSLNGLVIADEKIQTHF-PYNYL[illegible]FSLYPRTYNTQLV-
031	YP_006732334.1	--
032	YP_009041481.1	CAQ---VPIPGDYNRE----GKKYGLRRASTYKGK[illegible]HA-THVRTFKRKDA
033	YP_001036293.1	CSK---IPIPGYYKGRE----KKYGVRKSTTYKGK[illegible]HD-SHVRIERRKHL
034	YP_605811.1	CKG---TPVHGLYGKDKAYG-RKYGVRKSTSYKGK[illegible]HK-SHVRIDKKKHL
035	NP_955577.1	EGT---KALLETLGNKGYRASAKKAQICLQEVTYL[illegible]YSLKDGQRWLTKAR
036	YP_003208050.1	CRK---FPIPGYYQK-S---QKKYGLRKSQSYKGK[illegible]HD-THVRVVKNK--
037	NP_789740.1	RTK---IEELRQHLLRWGLTTPDKKHQKEPPFLWM[illegible]YELHPDKWTVQPIV
038	YP_004442839.1	CKG---TPVHGLYGKDKSYG-RRYGVRKSTSYKGK[illegible]HK-SHVRIDKKKHL
039	YP_009507791.1	EKI---IQELRDLLIFWGFETPEDKLQQEPPYKWM[illegible]YTLYPNKWTIQKTK
040	YP_004581513.1	--
041	NP_043933.1	--
042	YP_009507248.1	ENL---ERVFQRLRESNFKIQMDKSEFLKLETAYL[illegible]HIISRDGIKPNPDK
043	YP_002117531.1	CSS---FPIPNYQ-KGAGR---KFGLRKSKSYKGK[illegible]HK-THVRIEKSKYL
044	YP_004347415.1	--
045	NP_056891.1	QCF---DQLKQELTAAGLHIAPEKVQLQD-PYTYL[illegible]FELNGPKITNQKA-
046	YP_009513242.1	EQL---EKVFSILLNAGYVVSLKKSEIAQREVEFL[illegible]FNITKEGRGLTETF
047	YP_009352866.1	CSQ---IPIPGDYNRE----GKKYGLRKAGTYKGK[illegible]HN-THVRAFKKKDQ
048	YP_009165750.1	--
049	NP_040840.1	RGI---VNELASYIAQYGFMLPEDKRQEGYPAKWL[illegible]FELHPEKWKFQKHT
050	YP_004222728.1	AAG---EEVISTLERAGFTISPDKVQREP-GVQYL[illegible]YKLG-STYVAPVG-
051	YP_009506264.1	CKE---FPIPGYYHKKPTK---KYGLRKTTTYRGK[illegible]HK-THVRIDKSKHL
052	YP_567050.1	QLS---KMTLQALVTHGLPVSQEKTQQTPGQIRFL[illegible]QVISPNHITYETTP
053	YP_009508571.1	DQL---EKVFQILLQAGYVVSLKKSEVAQKTVEFL[illegible]FNITKEGRGLTEAF
054	NP_056803.1	QQL---EKVFQILLQAGYVVSLKKSEIGQRTVEFL[illegible]FNITKEGRGLTDTF
055	NP_955564.1	EIL---TSMIQALNKHGLVVSTEKIQKYD-NLKYL[illegible]THIQGDSVSYQKL-
056	YP_009508411.1	CSQ---IPIPGYYKNSG----KKYGVRRSTTYKGK[illegible]HE-THVRIEKKKYL
057	YP_009140788.1	CSS---IPIPGYYKNTG----KKYGVRRSKTYKGK[illegible]HE-SHARIEKRKHL
058	YP_009508410.1	--
059	NP_040333.1	QGT---RALLQTLGDLGYRASAKKAQICQKQVKYL[illegible]YLLKEGQRWLTEAR
060	YP_008992013.1	PPT---ASKDKEPMHQFS-----AHTIDHPSSTDD[illegible]TSDSNLAVSDSHTE
061	NP_041734.1	CKD---MPLTGYYDKT-----SKYGMRKSRTYKGK[illegible]HA-SHARVEKRKHL
062	NP_056895.1	QCY---QALAARLRDLGFQVASEKTRQTPSPVPFL[illegible]QMVHNQIVTYQSLP

```
063 YP_009109689.1   Q G T - - - R H L L Q E L G E K G Y R A S A K K A Q I Q Q T K V T Y L G Y I L S E G K R W L T P G R
064 YP_002519387.1   - - - - - - - - - - - - - - - - - - - - - - - - - - - - - - - - - - - - - - - - - - - - - - - - - -
065 YP_595725.1      C K G - - - F P V V N P V G T R K Y G - - K K Y G T R K A R S Y R G K P H K - S H V R I E K K K Y L
066 YP_009508551.1   Q Q L - - - E K V F Q I L L Q A G Y V V S L K K S E I G Q K T V E F L G F N I T K E G R G L T D T F
067 YP_001497148.1   R G T - - - K H L L R E L G D K G Y R A S A K K A Q I Q Q T K V T Y L G Y I L S E G K R W L T P G R
068 NP_041186.1      Q A F - - - S I L K Q H L S L N G L V I A D E K I Q T H F - P Y N Y L G F S L Y P R V Y N T Q L V -
069 NP_663784.1      D K V - - - V L Q L K E L L N G L G F S T P D E K F Q K D P P F Q W M G C E L W P T K W K L Q K L Q
070 YP_009508443.1   C N Q - - - I P I P G Y Y K G K E - - - - K R Y G A R R S T T Y K G K P H D - S H V R I E K K K H L
071 YP_003987465.1   C K E - - - F P I P N F Y - K G P Q K - - - K Y G V R K S T T Y R G K P H K - S H I R I D K T K H L
072 NP_861410.1      - - - - - - - - - - - - - - - - - - - - - - - - - - - - - - - - - - - - - - - - - - - - - - - - - -
073 YP_009345071.1   C K Q - - - M P I P G Y Y G T D R - - - - K R L G I R K A T H Y K G K P H S - T H V K I E K H K H L
074 YP_009508582.1   K I L - - - E K V F K I L L E A G Y I V S L K K S A L L R H E V T F L G F S I T Q T G R G L T S E F
075 NP_569141.1      - - - - - - - - - - - - - - - - - - - - - - - - - - - - - - - - - - - - - - - - - - - - - - - - - -
076 YP_002916057.1   - - - - - - - - - - - - - - - - - - - - - - - - - - - - - - - - - - - - - - - - - - - - - - - - - -
077 NP_777384.2      Q C Y - - - Q A L A A R L R D L G F Q V A S E K T R Q T P S P V P F L G Q M V H N Q I V T Y Q S L P
078 YP_233110.1      C K G - - - T P V H G L Y G K E K A Y G - R K Y G V R K S T S Y K G K P H K - S H V R I D K K K H L
079 NP_542258.1      A L T - - - D K V T N A L T K E G L P M S L E K T Q A T P G S I H F L G Q V I S P D C I T Y E T L P
080 YP_009130664.1   C S S - - - I P I P G Y Y K - I E - - - - K K Y G V R R S R T Y K G K P H A - S H A R I E R R K H L
081 YP_009508566.1   Q V L - - - D K I F Q K L L Q A G Y V V S L K K S N L Q R Y E V T F L G F T I S K Y G R G L T E E F
082 YP_006607892.1   - - - - - - - - - - - - - - - - - - - - - - - - - - - - - - - - - - - - - - - - - - - - - - - - - -
083 YP_009506270.1   C R N - - - T P V H G L Y G K E K S Y - - R K Y G A R K S T S Y K G K P H K - S H V R I G K K K H L
084 NP_042513.1      - - - - - - - - - - - - - - - - - - - - - - - - - - - - - - - - - - - - - - - - - - - - - - - - - -
085 YP_006907834.1   - - - - - - - - - - - - - - - - - - - - - - - - - - - - - - - - - - - - - - - - - - - - - - - - - -
086 YP_004442827.1   C K G - - - F P V V N P M - T R K Y - - - K S L G T R R T T T Y K G K P H K - T H A R I D K K K Y L
087 YP_009408594.1   C S Q - - - I P L P G Y Y G P - - - - - R K K Y G L R K A K N Y S G K P H K - S H V R V I K K K Y Q
088 YP_009508561.1   Q V L - - - D S I F R K L L E T G Y I V S L K K S A L A R Y E V N F L G F V I S E T G R G L T S E F
089 NP_056907.1      Q L S - - - K M T L Q A L V T H G L P V S Q E K T Q Q T P G Q I R F L G Q V I S P N H I T Y E T T P
090 YP_002308474.1   V L A - - - Q E I T N A L A Q E G L P M S T E K T Q S T P G P I H F L G Q T I S K K Y I T Y E T L P
091 YP_007761644.1   - - - - - - - - - - - - - - - - - - - - - - - - - - - - - - - - - - - - - - - - - - - - - - - - - -
092 NP_040550.1      A A G - - - E E V I S T L E R A G F T I S P D K I Q R E P - G V Q Y L G Y K L G - S T Y V A P V G -
093 NP_758808.1      C N Q - - - I P L P G I Y T K - G - - - N K K F G L R K A R T Y K G K P H P - T H V R V F K - - - K
094 YP_009345075.1   C K D - - - I P I P G V Y P R - - - - - E K K Y S L R K A K N Y K G K P H D - T H V R V F K R K - -
095 NP_068729.1      - - - - - - - - - - - - - - - - - - - - - - - - - - - - - - - - - - - - - - - - - - - - - - - - - -
096 NP_047255.1      E G T - - - K A L L E T L G N K G Y R A S A K K A Q I Q L Q E V T Y L G Y S L K D G Q R W L T K A R
097 YP_006273075.1   C S S - - - I P I P G Y Y K N T G - - - - K K Y G V R R S K T Y K G K P H E - S H A R I E K R K H L
098 YP_009508888.1   Q V L - - - Q Q V L H I L H D A G Y V V S L K K S A I A Q K V V E F L G F N I T K T G R G L T D A F
099 NP_395469.1      - - - - - - - - - - - - - - - - - - - - - - - - - - - - - - - - - - - - - - - - - - - - - - - - - -
100 YP_009508546.1   Q Q L - - - E K V F Q I L L Q A G Y V V S L K K S E I G Q K T V E F L G F N I T K E G R G L T D T F
101 NP_056762.1      A F C R S I Y T I G D Y K K R K I L K R V T N Y N K N R R K N Y V R R P S I K K K C R C Y I C Q D E
102 YP_009508556.1   E Q L - - - E K V F S I L L N A G Y V V S L K K S E I A Q R E V E F L G F N I T K D G R G L T E T F
103 NP_569153.1      C S S - - - I P I P G Y Y G G K S G E - - K R Y G V R R T T T Y K G K P H S - T H A R I E K T K H L
104 NP_043924.1      R S L - - - A I L L Q G L K D L G V K I N P K K S H F Q K D Q V Q Y L G V N V G A D T R S L I D A R
105 YP_009243641.1   D L L - - - E Q L P L Y F K P Y G L I I A P E K I Q L E D - I S H Y L G F V V S R T T V H P Q K M -
106 YP_004300274.1   - - - - - - - - - - - - - - - - - - - - - - - - - - - - - - - - - - - - - - - - - - - - - - - - - -
107 NP_056848.1      - - - - - - - - - - - - - - - - - - - - - - - - - - - - - - - - - - - - - - - - - - - - - - - - - -
108 YP_006495799.1   C R T - - - M P V P G Y Y E K - - - - - - K K Y G V R K S T T Y K G K P H D - T H V K V I K N K D K
109 YP_009508409.1   - - - - - - - - - - - - - - - - - - - - - - - - - - - - - - - - - - - - - - - - - - - - - - - - - -
110 NP_687035.1      N Q Q - - - V D I V R K M L L E K G L E T P D K K V Q R E P P W E W M G Y K L H P N K W T I N K I E
111 NP_045937.2      K D T - - - A V M L Q H L A S E G H K V S K K K I Q L Q Q Q E V V Y L G Q L L T P E G R K I L P D R
112 NP_612577.1      - - - - - - - - - - - - - - - - - - - - - - - - - - - - - - - - - - - - - - - - - - - - - - - - - -
113 NP_619548.1      - - - - - - - - - - - - - - - - - - - - - - - - - - - - - - - - - - - - - - - - - - - - - - - - - -
114 NP_056880.1      E I L - - - T S M I Q A L N K H G L V V S T E K I Q K Y D - N L K Y L G T H I Q G D S V S Y Q K L -
115 YP_009121747.1   C S S - - - I P I P G Y Y D R - Q - - - R K K Y G I R K S R T Y K G K P H D K T H V K V F K R K Y K
116 YP_009113237.2   C K D - - - I P I P G Y Y Q S R R N T - G K R N S L R R S Q T Y K G K P H S - T H I R I D K A K Y L
117 YP_009508408.1   C N Q - - - I P I P G Y Y K G K E - - - - K R Y G V R R S T T Y K G K P H D - S H V R I E K K K H L
118 NP_955579.1      E G T - - - K A L L E T L G N K G Y R A S A K K A Q I Q L Q E V T Y L G Y S L K D G Q R W L T K A R
119 YP_009229919.1   C K E - - - V H I P G Y Y K T - - - - - G K R Y G L R K A K T Y K G K P H D - T H V R V F K K K - -
120 YP_004442833.1   C R G - - - F P V I N P L - Q R K Y - - - R S L G T R R T R T Y K G K P H S - S H A R I D K R K Y L
121 NP_862833.2      Q A F - - - S L L K Q H L S L N G L V X A D E K I Q T H F - P Y N Y L G F H L Y P R A Y T T Q L V -
122 NP_040563.1      K Q I - - - V Q E I R D K L G S Y G F K T P D E K V Q E E - R V K W I G F E L T P K K W R F Q P R Q
123 YP_443922.1      A A T - - - K I L L D F L A T K G Y K V K R S K V Q T Q R R T V L F L G R E V S A E G A G L S T A H
124 NP_057933.2      Q G T - - - R A L L Q T L G N L G Y R A S A K K A Q I Q Q K Q V K Y L G Y L L K E G Q R W L T E A R
125 YP_009116631.1   C R N - - - M P V P G Y Y E K - - - - - - K Q Y G I R K A K T Y K G K P H P - T H V K V I K N K Y K
126 YP_009513249.1   E Y L - - - D I L F N R L K E A G Y I I S L K K S N I A N S I V D F L G F Q I T N E G R G L T D T F
127 YP_223871.1      S A T - - - R D L L M T L A E L G Y R V S G K K A Q L Q Q E E V T Y L G F K I H K G S R S L S N S R
128 NP_127504.1      P Q S - - - S S H L G D Q F M I S D P A L P K V Y A L N E P S V P S E D T S - S Q S Y I S T E E S V
129 NP_044929.1      D I L - - - E T I F N R L S T A G Y I V S L K K S K L A K E T V E F L G F S I S Q N G R G L T D S Y
130 NP_057860.1      L L S - - - E A T M A S L I S H G L P V S E N K T Q Q T P G T I K F L G Q I I S P N H L T Y D A V P
131 NP_040973.1      K E K - - - V E E L R K L L L W W G F E T P E D K L Q E E P P Y T W M G Y E L H P L T W T I Q Q K Q
132 NP_777317.1      C K S - - - T P V H G V Y G E R K S Y K - G R F G A R K S T T Y N G K P H K - S H V R I A R K K H L
133 NP_054716.1      K T M - - - D L L F Q K L Q T A G Y I V S L K K S K L G Q H T V D F L G F Q I T Q T G R G L T D S Y
134 YP_001856242.1   R T K - - - I E E L R Q H L L R W G L T T P D K K H Q K E P P F L W M G Y E L H P D K W T V Q P I V
135 NP_954565.2      Q C F - - - D Q L K Q E L T A A G L H I A P E K V Q L Q D - P Y T Y L G F E L N G P K I T N Q K A -
```

```
136 NP_569150.1      CRS---TPVHGLY-RDKSY--RKYGARKSTSYKGKPHK-SHVRIGKKKYL
137 NP_659397.1      --------------------------------------------------
138 YP_009513211.1   EGT---RRLLQELSKLGYRVSAKKAQLCREEVTYLGYLLKGGKRWLTPAR
139 YP_233107.1      CGN---TPVHGIYGKNKEGGFRKYGVRKNTSYKGKPHK-SHIRIGKQKHL
140 YP_009109692.1   QGT---RHLLQELGEKGYRASAKKAQICQTKVTYLGYILSEGKRWLTPGR
141 NP_057849.4      RTK---IEELRQHLLRWGLTTPDKKHQKEPEFLWMGYELHPDKWTVQPIV
142 NP_049560.1      LLS---EVTMASLISHGLPVSENKTQQTPGTIKFLGQIISPNHLTYDAVP
143 YP_009506267.1   CSK---IPIPGYYGGAE----KKYGVRKSKTYKGKPHQ-SHVRIEKRKHL
144 NP_039820.1      CRD---FPIEGYYGRSGEK--KKYTARKATKYTGKAHD-NHIRVTKAKY-
145 NP_056790.1      KGT---QKLLQELSKLGYRVSAKKAQLCQREVTYLGYLLKEGKRWLTPAR
146 YP_002321513.1   CKD---IRLPGQYARDS---SKRYGMRKANTYKGKPHR-THARVIKNKEK
147 NP_955591.1      QGT---RALLQTLGNLGYRASAKKAQICQKQVKYLGYLLKEGQRWLTEAR
148 YP_001956722.2   EQL---EKVFSLLLNAGYVVSLKKSEIAQHEVEFLGFNITKEGRGLTETF
149 NP_056902.1      KEL---IIELRAILLEKGFETPDDKLQEVPEYSWLGYQLCPENWKVQKMQ
150 NP_056886.1      AAG---EEVISTLERAGFTISPDKVQREP-GVQYLGYKLG-STYVAPVG-
151 NP_041261.1      ACY---AHIISCLTSYGLKIAPDKVQVSE-EFSYLGFELHHQQVFTPRV-
```

```
001 YP_004442836.1   AAR-----KRNCRCYACGEEGHFASECT-NQKKIIDRVRVLDSLDLEDGL
002 NP_056728.1      --------------------------------------------------
003 YP_009508537.1   KEKLENVTAPTTLKQLQSILGLLNFARNFIPDFTELIAPLYALIPKSTK-
004 YP_004732983.2   GAQG-----RKCKCYLCGIEGHYARECPKKHVR-PERAAYFEGMGLDVNW
005 YP_009508407.1   --------------------------------------------------
006 NP_040939.1      LPELTKG--TITLNKLQKLVGELVWR---QSIIGKSIPNILKLMEGDREL
007 YP_003864102.1   VIRKDK---LQTLNDYQKLLGDINWLRPYLKLTTGDLRPLFDILKGDPNP
008 YP_009002585.1   VR------NKRCKCYLCGEMGHFARECP-NDKKNTRRVAMYEQLNIPEGF
009 YP_003284237.1   KNR-----K--CKCYACGEEGHYASECR-NKKQYTDRVNIIENIALKEDE
010 YP_009268869.1   LPEIKEG--TITLNKLQKLVGDLVWR---QSLIGKSIPNILKLMEGDRAL
011 YP_009506251.1   VR------NKRCKCYLRGDEGHFARECP-NNKRDVKRVAIFEGINLPDDY
012 NP_955611.1      LVAEPR---IATLWDVQKLVGSLQWLRPALGIPPRLMGPFYEQLRG-SDP
013 YP_008567619.1   LR------NKNCKCYLCGEEGHFAKDCN-RNKRDIRRVAIFEGLELPEDH
014 NP_149413.1      KQRKDGKGEKKCRCFVCGSTEHLMKDCK-SPMKRQDRVNLAREIDIPDNY
015 YP_004442824.1   AMR-----KKDCKCFACGETGHFASECK-NPRKLVERVAIMDSLELPEDV
016 YP_001931967.1   --------------------------------------------------
017 YP_002455786.1   TIPIKA---HWTLTELQTLLGELQWVSKGTPVLREHLHCLYSALRGLKDP
018 YP_009109694.1   IETVARIPPPRNPREVREFLGTAGFCRLWIPGFAELAAPLYALTKES---
019 NP_789739.1      LPEKD----SWTVNDIQKLVGKLNWAS--QIYPGIKVRQLCKLLRGTKAL
020 NP_955619.1      KVPIRS---RWALPELQALLGEIQWVSKGTPTLRQPLHSLYCALQRHTDP
021 YP_224289.1      RNK-----K--CKCYACGEEGHYASECR-NPRKLMERVSVLQDLELGKDM
022 YP_009182100.1   MLIVSETGKAWSPDEARTYYACTFKGIARKWFDKYKETDEYVLWLSTIER
023 NP_705927.1      LPEKD----SWTVNDIQKLVGKLNWAS--QIYPGIKVRQLCKLLRGTKAL
024 YP_009508577.1   KDKIQNITSPRTLKELQSILGLFNFARNFVPNFSEIIKPLYSLISTAEG-
025 YP_004442830.1   QAR-----KKNCRCYVCGEEGHFAKECK-SQKKIQERVNIVNELDIPEGY
026 NP_758887.1      LPEKE----EWTVNDIQKLVGKLNWAS--QIYSGIKTKELCRLIRGAKPL
027 YP_009508406.1   --------------------------------------------------
028 YP_610965.1      -D---KGRTTKCRCYICGQEGHFARECR-NGRGNIARAAIIKDLDLPSDF
029 YP_001931961.1   --------------------------------------------------
030 YP_241114.1      KLQTDH---LKTLNDFQKLLGDINWIRPYLKLPTYTLQPLFDILKGDSDP
031 YP_006732334.1   --------------------------------------------------
032 YP_009041481.1   GKQ------RKCACFICGEPDHFAKECPRNGKGNINRVHYYDNLNIPEDW
033 YP_001036293.1   IR------SKKCKCFLCGAEGHFARECP-NDKRNVKRIAIFEGMEIPEDC
034 YP_605811.1      LMK-----RKDCKCFACGDVGHFASECP-NPKKLMHRVQILQSLELDDGI
035 NP_955577.1      KEAILSIPVPKNSRQVREFLGTAGYCRLWIPGFAELAAPLYPLTRPG---
036 YP_003208050.1   -D---KARSVKCKCFICGEEGHFARECR-SKKGNIARAAVVDNLDLPPDF
037 NP_789740.1      LPEKD----SWTVNDIQKLVGKLNWAS--QIYPGIKVRQLCKLLRGTKAL
038 YP_004442839.1   TMK-----RKDCKCFACGDIGHFASECP-NPKKLMHRVQILQSLELEDGI
039 YP_009507791.1   L-DIPE---VPTLNQLQKLAGVINWAT--QNVGGIKIKALTELMKGNQQL
040 YP_004581513.1   --------------------------------------------------
041 NP_043933.1      --------------------------------------------------
042 YP_009507248.1   ISAIQKYLIPKTPKEIKQFLGLLGYYRKFIPDFARLTKPLTQCLKKGSKV
043 YP_002117531.1   RSK-----K--CKCYACGEEGHFAKDCK-NPRKLTERVAILEDLDLGDDY
044 YP_004347415.1   --------------------------------------------------
045 NP_056891.1      VIRKDK---LQTLNDFQKLLGDINWLRPYLKLTTGDLKPLFDTLKGDSDP
046 YP_009513242.1   KQKLLNVIPPKDLKQLQSILGLLNFARNFIPNYSELVKPLYTIVANANG-
047 YP_009352866.1   QKQ------RKCACFICGEPDHFARECPRSGKGNINRVHYYDNLNIPQDW
048 YP_009165750.1   --------------------------------------------------
049 NP_040840.1      LPEITEG--PITLNKLQKLVGDLVWR---QSLIGKSIPNILKLMEGDRAL
050 YP_004222728.1   LVAEPR---IATLWDVQKLVGSLQWLRPALGIPPRLMGPFYEQLRG-SDP
051 YP_009506264.1   KNR-----K--CKCYACGEEGHFARDCR-NPRKIMDRVNVLEDLELKDGM
052 YP_567050.1      TIPIKS---QWTLTELQTVLGEIQWVSKGTPILRKHLQCLYSALRGYQDP
053 YP_009508571.1   KAKLLDITPPKDLKQLQSILGLLNFARNFILNFAELVKPLYSLISSAKG-
054 NP_056803.1      KTKLLNVTPPKDLKQLQSILGLLNFARNFIPNFAELVQTLYNLIASSKG-
```

```
055 NP_955564.1       QIRTDK---LRTLNDFQKLLGNINWIRPFLKLTTGELKPLFEILNGDSNP
056 YP_009508411.1    ERGS-ALKNKKCKCFLCGEEGHFARECS-SDRRNIKRVAIFEGIELPEDC
057 YP_009140788.1    VR------NKRCKCFLCGQEGHFARECP-NDRKSSKRMAMFENLELPDDC
058 YP_009508410.1    --------------------------------------------------
059 NP_040333.1       KETVMGQPTPKTPRQLREFLGTAGFCRLWIPGFAEMAAPLYPLTKTG---
060 YP_008992013.1    IDTESSASTSDSEKSYADITRILMAQPD------PQTSRTEPYVNIPSDV
061 NP_041734.1       IR------NKKCKCYLCGDEGHFARECP-NQRRDVKRVAIFEGIDLPEGF
062 NP_056895.1       TLQISS---PISLHQLQAVLGDLQWVSRGTPTTRRPLQLLYSSLKGIDDP
063 YP_009109689.1    IETVARIPPPRNPREVREFLGTAGFCRLWIPGFAELAAPLYALTKES---
064 YP_002519387.1    --------------------------------------------------
065 YP_595725.1       QQR-----EKKCRCYVCGSPDHLMKDCK-SPMKRQERVNLANELDIPDGY
066 YP_009508551.1    KTKLLNITPPKDLKQLQSILGLLNFARNFIPNFAELVQPLYNLIASAKG-
067 YP_001497148.1    IETVARIPPPQSPREVREFLGTAGFCRLWIPGFAELAAPLYALTKES---
068 NP_041186.1       KLQTDH---LKTLNDFQKLLGDINWIRPYLKLPTYTLQPLFDILKGDSDP
069 NP_663784.1       LPQKD----IWTVNDIQKLVGVLNWAA--QIYSGIKTKHLCRLIRGKMTL
070 YP_009508443.1    VR------ERRCKCFLCGAEGHFARECP-NDKRNIKRVAVFEGLEIPEDC
071 YP_003987465.1    KNR-----K--CKCYICGEEGHFARDCK-NPKKIVDRVHVLEELDIQDGM
072 NP_861410.1       --------------------------------------------------
073 YP_009345071.1    RT-------KKCKCYLCGEEGHFARECK-NDNRNIHRQTMYQSLELPKEF
074 YP_009508582.1    KDKIQNITPPKTLKELQSILGLFNFARNFVPNFSEIIKPLYSLISTAEG-
075 NP_569141.1       --------------------------------------------------
076 YP_002916057.1    --------------------------------------------------
077 NP_777384.2       TLQISS---PISLHQLQAVLGDLQWVSRGTPTTRRPLQLLYSSLKGIDDP
078 YP_233110.1       LMK-----RKDCKCFACGDIGHFASECP-NPKKLMHRVQILQSLELDDGI
079 NP_542258.1       SIHVKS---IWSLAELQSMLGELQWVSKGTPVLRSSLHQLYLALRGHRDP
080 YP_009130664.1    QR------NKRCKCYLCGQEGHFARECP-NDRRSIKRVAIFENLDLPEDC
081 YP_009508566.1    QEKLRNISPPNSLKQLQSILGLLNFARNFIPNFSELIKPLYELISTAQG-
082 YP_006607892.1    --------------------------------------------------
083 YP_009506270.1    ALR-----KKNCRCYACGEEGHFASECR-NPRKIMDRVKVLDSLELEDGL
084 NP_042513.1       --------------------------------------------------
085 YP_006907834.1    --------------------------------------------------
086 YP_004442827.1    QGR-----KKDCRCYVCGEEGHFAKECR-SKKRIQERVNIVQELDIPDGY
087 YP_009408594.1    HD---RGRVKKCKCFICGKEGHFARECR-NNVGNKERVAMLNELDLPEDY
088 YP_009508561.1    RERLQEITPPTTLKQLQSILGFLNFARNFVPNFSELVQPLYQLISTASG-
089 NP_056907.1       TIPIKS---QWTLTELQTVLGEIQWVSKGTPILRKHLQCLYSALRGYQDP
090 YP_002308474.1    TIHVKP---NWTLTELQSTLGELQWVSKGTPTLRSSLHQLYTALRGHHDP
091 YP_007761644.1    --------------------------------------------------
092 NP_040550.1       LVAEPR---IATLWDVQKLVGSLQWLRPALGIPPRLMGPFYEQLRG-SDP
093 NP_758808.1       AK---YQRTKKCKCFICGEPGHFARECT-KQRGNIVRATVHQELAIPDNF
094 YP_009345075.1    ------KPPVKCKCYICGREGHFARDCN-NNNGNIARAAVIENLELPPDW
095 NP_068729.1       --------------------------------------------------
096 NP_047255.1       KEAILSIPVPKNSRQVREFLGTAGYCRLWIPGFAELAAPLYPLTRPG---
097 YP_006273075.1    VR------NKRCKCFLCGQEGHFARKCP-NDKRSSKRMAMFENLEIPEDC
098 YP_009508888.1    KEKLLNISPPQNLKQLQSILGLMNFARNFIPNYAERVKPFYSLISTAKS-
099 NP_395469.1       --------------------------------------------------
100 YP_009508546.1    KTKLLNVTPPKDLKQLQSILGLLNFARNFIPNFAELVQPLYNLIPSARG-
101 NP_056762.1       NHLANRCPRRYTNQARASLIDGLDEDIVSIASDDEDIENFLEIIELDEFI
102 YP_009508556.1    KLKLLDITPPKDLKQLQSILGLLNFARNFIPNYSELVKPLYTIVANANG-
103 NP_569153.1       R-------NKKCKCYLCGEEGHFARECP-NDRRNVKRVAMFEGLDLPDDC
104 NP_043924.1       SQLIRTLDIPLTVQGLRSALGLFNFCRAWIPEFSRKTQSLYDMLKGDCKS
105 YP_009243641.1    TIRRDN---LKTLNDFQKLLGDINWLRPSLGIPTYQLHNLFDTLKGDTDL
106 YP_004300274.1    --------------------------------------------------
107 NP_056848.1       --------------------------------------------------
108 YP_006495799.1    NAPG-----RKCKCYLCGIEGHYARECPKKTVQ-PQRMAYFEGLALADNW
109 YP_009508409.1    --------------------------------------------------
110 NP_687035.1       LPPLEG---EWTVNKIQKVVGVLNWAS--QIYPGIKTKHTCAMLRGKKNL
111 NP_045937.2       KVTVSQFQQPTTIRQIRAFLGLVGYCRHWIPEFSIHSKFLEKQLKKDTAE
112 NP_612577.1       --------------------------------------------------
113 NP_619548.1       --------------------------------------------------
114 NP_056880.1       QIRTDK---LRTLNDFQKLLGNINWIRPFLKLTTGELKPLFEILNGDSNP
115 YP_009121747.1    ED---RGRVSKCKCYICGREGHFARDCK-SKTGNMARRAVIDNLDLKEDW
116 YP_009113237.2    Q-------NRKCKCYACGEEGHFSKDCP-NDKKSSKRVALLQNLEIPEDY
117 YP_009508408.1    MR------EKRCKCFLCGNEGHFARECP-NDKRNIKRVAVFEGLEVPDDC
118 NP_955579.1       KEAILSIPVPKNSRQVREFLGTAGYCRLWIPGFAELAAPLYPLTRPG---
119 YP_009229919.1    -H---ADKVRKCKCFICGEEGHFARECK-RGKGNIARAAVLKDLDIPDDY
120 YP_004442833.1    QGR-----KKECRCYVCGEEGHFAKECK-SKKKIQERVNIVNELDIPEGY
121 NP_862833.2       KLQTDH---LKTLNDFQKLLGDINWIRPYLKLPTYILQPLFDILKGNSDP
122 NP_040563.1       LKIKN----PLTVNELQQLVGNCVWVQ--PEVK-IPLYPLTDLLRDKTNL
123 YP_443922.1       RNSILHHPRPTTVSGLLSFLGLTGYSRTHIPDYTCRTEPLREIVRQAGAR
124 NP_057933.2       KETVMGQPTPKTPRQLREFLGTAGFCRLWIPGFAEMAAPLYPLTKTG---
125 YP_009116631.1    HTQG-----KKCKCYLCGIEGHYARECPKKVVK-PQRAAYFNGMGLDDNW
126 YP_009513249.1    KEKLENITAPTTLKQLQSILGLLNFARNFIPDFTELIAPLYALIPKSTK-
127 YP_223871.1       TQAILQIPVPKTKRQVREFLGTIGYCRLWIPGFAELAQPLYAATRGGN--
```

```
128 NP_127504.1       EDTDSFSVVSEESTQLSQLSSSSNDSPENNENTLPQTFMVRPTEPEISEV
129 NP_044929.1       KQKLMDLQPPTTLRQLQSILGLINFARNFLPNFAELVAPLYQLIPKAKG-
130 NP_057860.1       KVPIRS---RWALPELQALLGEIQWVSKGTPTLRQPLHSLYCALQRHTDP
131 NP_040973.1       L-DIPE---QPTLNELQKLAGKINWAS--QAIPDLSIKALTNMMRGNQNL
132 NP_777317.1       ALR-----KSECKCYACGELGHFASDCN-NPRKLTQRVAILDSLELEKGI
133 NP_054716.1       KSKLLDITPPNTLKQLQSILGLLNFARNFIPNYSELITPLYQLIPLAKG-
134 YP_001856242.1    LPEKD----SWTVNDIQKLVGKLNWAS--QIYPGIKVRQLCKLLRGTKAL
135 NP_954565.2       VIRKDK---LQTLNDFQKLLGDINWLRPYLKLTTGDLKPLFDTLKGDSDP
136 NP_569150.1       SLR-----KKNCRCYACGEEGHFASECK-NPRKIMDRVKVLDSLDLEDGL
137 NP_659397.1       --------------------------------------------------
138 YP_009513211.1    KATVMKIPTPTTPRQVREFLGTAGFCRLWIPGFASLAAPLYPLTREK---
139 YP_233107.1       NLR-----KKDCKCFACGETGHYASECT-NPKKFTHRVAILESLDLQEGL
140 YP_009109692.1    IETVARIPPPRNPREVREFLGTAGFCRLWIPGFAELAAPLYALTKES---
141 NP_057849.4       LPEKD----SWTVNDIQKLVGKLNWAS--QIYPGIKVRQLCKLLRGTKAL
142 NP_049560.1       TVPIRS---RWALPELQALLGEIQWVSKGTPTLRQPLHSLYCALQRHTDP
143 YP_009506267.1    TR------EKRCKCFLCGKEGHYARECP-NDRRDIKRVAIFEGLSIPDDF
144 NP_039820.1       --------QRKCKCYICGQEGHYANQCR-NKHKDQQRVAILQSLDLKENE
145 NP_056790.1       KATVMKIPVPTTPRQVREFLGTAGFCRLWIPGFASLAAPLYPLTKES---
146 YP_002321513.1    SKK---GVIRKCKCYICGEDGHFARECR-SKMVNVQRAAIFNELELDDSL
147 NP_955591.1       KETVMGQPTPKTPRQLREFLGTAGFCRLWIPGFAEMAAPLYPLTKTG---
148 YP_001956722.2    KQKLLNITPPRDLKQLQSILGLLNFARNFIPNFSELVKPLYNIIATANG-
149 NP_056902.1       LDMVKN----PTLNDVQKLMGNITWMS--SGIPGLTVKHIAATTKGCLEL
150 NP_056886.1       LVAEPR---IATLWDVQKLVGSLQWLRPALGIPPRLMGPFYEQLRG-SDP
151 NP_041261.1       CLKTDH---LKTLNDFQKLLGDIQWLRPYLKLPTSALVPLNNILKGDPNP
```

```
001 YP_004442836.1    DVVSVGFDEDDVSDIYSIDEEAENNKFTNED-------------------
002 NP_056728.1       --------------------------------------------------
003 YP_009508537.1    ---NYVPWQTEHST------------------------------------
004 YP_004732983.2    DVISVDPGDQDGSDICSISEGEAQHGMEDLAAFKAQLPYPV---------
005 YP_009508407.1    --------------------------------------------------
006 NP_040939.1       QS--ERKIEEVHVK------------------------------------
007 YP_003864102.1    NS--PRSLSDEALI------------------------------------
008 YP_009002585.1    EVVSVEEGEEVSDAIFSISENEDEDALHTGGAFAETIF------------
009 YP_003284237.1    EIVSVGEDEDEVSDIWSLSEGEDGH-----G-------------------
010 YP_009268869.1    QS--ERRIELRHVK------------------------------------
011 YP_009506251.1    EIVSVEEGEEDSDAIYSLSENEE---ELQDEVLHEKVF------------
012 NP_955611.1       NE--AREWNLDMKM------------------------------------
013 YP_008567619.1    DVVSVSEGEDDSDAIYSVSEGE-------DGSIATMT-------------
014 NP_149413.1       DIVSVGFDEEDTEDIYSVSENEENQAHLGVSYEDPQLPTIP---------
015 YP_004442824.1    DVISVGMDEDDISEIYSVGEEIDEYQFQQEE-------------------
016 YP_001931967.1    --------------------------------------------------
017 YP_002455786.1    RD--TITLRHPHLH------------------------------------
018 YP_009109694.1    ---TPFTWQTEHQL------------------------------------
019 NP_789739.1       TE--VIPLTEEAEL------------------------------------
020 NP_955619.1       RD--QIYLNPSQVQ------------------------------------
021 YP_224289.1       EIVSVEENETELSDIYSISEGEESGPPKSEI-------------------
022 YP_009182100.1    SKSPSDFATCLYKQFCREQDPSKKEKDLEILKGKLVNLTISDLKYFLSYV
023 NP_705927.1       TE--VIPLTEEAEL------------------------------------
024 YP_009508577.1    ---NNIKWTSEHTR------------------------------------
025 YP_004442830.1    DIVSVGYDEKSVEDIFSISEGEDQQAHILKE-EDEQLPTTP---------
026 NP_758887.1       DE--KVEWTREAEL------------------------------------
027 YP_009508406.1    --------------------------------------------------
028 YP_610965.1       DVLSVDNNDPMSDAICSFSEGEAG-GSQTIKTALSDLP------------
029 YP_001931961.1    --------------------------------------------------
030 YP_241114.1       AS--PRTLSLEGQT------------------------------------
031 YP_006732334.1    --------------------------------------------------
032 YP_009041481.1    DIMSVDPEEPMSDAICSMSEGE-AGFTATAYNFEDDLPYG----------
033 YP_001036293.1    EIVSVQEGDELSDSIFSVSEGE---EVLQSLNPFIQQ-------------
034 YP_605811.1       DVISVGFDESDVSDIYSVSEGEDNYQFNNED-------------------
035 NP_955577.1       ---TLFQWGTEQQL------------------------------------
036 YP_003208050.1    DVLSLDLNESDSDAICSFSEGEIANGARYVQEELHKLP------------
037 NP_789740.1       TE--VIPLTEEAEL------------------------------------
038 YP_004442839.1    DVISVGFDESDVSDIYSVSEGEDNYQFKHED-------------------
039 YP_009507791.1    DS--YRQWTPEALGHLQKIKAGEPKAVTLRQGPKESYKDFIDR-------
040 YP_004581513.1    --------------------------------------------------
041 NP_043933.1       --------------------------------------------------
042 YP_009507248.1    TLSPEYVN------------------------------------------
043 YP_002117531.1    DVVSVGLDEGDQSDIYSVSEGEEGHS----D-------------------
044 YP_004347415.1    --------------------------------------------------
045 NP_056891.1       NS--HRSLSKEALA------------------------------------
046 YP_009513242.1    ---KFISWTEENSN------------------------------------
```

```
047 YP_009352866.1   DIMSVDPDEPMSDAICSMSEGE-AGFSATAFQFDDDLPYG----------
048 YP_009165750.1   --------------------------------------------------
049 NP_040840.1      QS--ERYIESIHVR------------------------------------
050 YP_004222728.1   NE--AREWNLDMKM------------------------------------
051 YP_009506264.1   EVVSVGENEDDLSDVYSLSEGETG------G-------------------
052 YP_567050.1      RA--HLLLQKQQLH------------------------------------
053 YP_009508571.1   ---KYIEWSNENTV------------------------------------
054 NP_056803.1      ---KYIEWTEDNTK------------------------------------
055 NP_955564.1      IS--TRKLTPEACK------------------------------------
056 YP_009508411.1   EIVSVKEGEAQSDAIYSISEGEDDNEVHN-LNSITHS-------------
057 YP_009140788.1   DIVSVQEGEELSDAIYSISEGEDN---------DEELQ------------
058 YP_009508410.1   --------------------------------------------------
059 NP_040333.1      ---TLFEWGPDQQK------------------------------------
060 YP_008992013.1   EEEMPESSATNQPPP-AQTTSSSQKSSNGPWFTYDDIPSYKWR-------
061 NP_041734.1      DIVSVEEGEEESDAIYSISENEDG--ELDTEVVHEKVF------------
062 NP_056895.1      RA--IIQLSPEQLQ------------------------------------
063 YP_009109689.1   ---TPFTWQTEHQL------------------------------------
064 YP_002519387.1   --------------------------------------------------
065 YP_595725.1      DLVSVGYDESDIDEIYSVSENEECQAHLGLN-EDEQLPKVP---------
066 YP_009508551.1   ---KYIEWSEENTK------------------------------------
067 YP_001497148.1   ---APFTWQEKHQS------------------------------------
068 NP_041186.1      AS--PRTLSLEGRT------------------------------------
069 NP_663784.1      TE--EVQWTELAEA------------------------------------
070 YP_009508443.1   EIVSVHEGDDLSDAIYSVSEGENNEEIDQGVNVFINR-------------
071 YP_003987465.1   DVVSVGENEDELSDIYSVSEGEES--------------------------
072 NP_861410.1      --------------------------------------------------
073 YP_009345071.1   ELVSAEENDS-GDEIYSMSEGEDPTTMFMNLTPLAQIEDCLTLDWEYKDV
074 YP_009508582.1   ---NNIKWTSEHTR------------------------------------
075 NP_569141.1      --------------------------------------------------
076 YP_002916057.1   --------------------------------------------------
077 NP_777384.2      RA--IIQLSPEQLQ------------------------------------
078 YP_233110.1      DVISVGFDESDVSDIYSVSEGEDSYQFNNED-------------------
079 NP_542258.1      RD--TIELTSTQVQ------------------------------------
080 YP_009130664.1   DIVSVQEGDNESDAIYNISEGEEMEDLTKSLNVMSVS-------------
081 YP_009508566.1   ---QSISWEPKHSQ------------------------------------
082 YP_006607892.1   --------------------------------------------------
083 YP_009506270.1   DVVSVGFDEDDVSDIYSIDEEADNYKFTNEE-------------------
084 NP_042513.1      --------------------------------------------------
085 YP_006907834.1   --------------------------------------------------
086 YP_004442827.1   DIISVGYDEKSIEDIYSISEGEDNQAHLGRD-EEQ-LPEVP---------
087 YP_009408594.1   DIVSVDLNEPDSDAICSVSEGETS-TEQQCQAATLEFP------------
088 YP_009508561.1   ---NFIQWTAEHTL------------------------------------
089 NP_056907.1      RA--HLLLQKQQLH------------------------------------
090 YP_002308474.1   RD--TIQLTPPQLQ------------------------------------
091 YP_007761644.1   --------------------------------------------------
092 NP_040550.1      NE--AREWNLDMKM------------------------------------
093 NP_758808.1      DVVSVDADESDSSGIYSYSENEAPLQEVNSFIHDENIF------------
094 YP_009345075.1   DVVSVDINDPDSDAICSVSDGEPEGQEEVLNGISPDP-------------
095 NP_068729.1      --------------------------------------------------
096 NP_047255.1      ---TLFQWGTEQQL------------------------------------
097 YP_006273075.1   DIVSVEEGEDMSDAIYSISEGEDG---------ADELQ------------
098 YP_009508888.1   ---NNILWNDELTS------------------------------------
099 NP_395469.1      --------------------------------------------------
100 YP_009508546.1   ---KYIEWSKENTK------------------------------------
101 NP_056762.1      AHSSQEHEHTWEIGG-----------------------------------
102 YP_009508556.1   ---KFISWTEENSN------------------------------------
103 NP_569153.1      EIVSIDEGDPDSDAIFSISEGEE-----AGTLEEQCF-------------
104 NP_043924.1      TDKLKWTEDNLNKFKLLKDEVASACVLGLPDPTLPFRHLIG---------
105 YP_009243641.1   NS--ARCLTPAAEE------------------------------------
106 YP_004300274.1   --------------------------------------------------
107 NP_056848.1      --------------------------------------------------
108 YP_006495799.1   DVLSVDVGESDSEGVCSISEGETAGRMDELAAFKTQLPYPV---------
109 YP_009508409.1   --------------------------------------------------
110 NP_687035.1      LE--EIVWTEEAEA------------------------------------
111 NP_045937.2      PFQLDDQQVEAFNK------------------------------------
112 NP_612577.1      --------------------------------------------------
113 NP_619548.1      --------------------------------------------------
114 NP_056880.1      IS--TRKLTPEACK------------------------------------
115 YP_009121747.1   DIVSADMSDHDT--VYSISEGEGGATEMNIGAMVEESP------------
116 YP_009113237.2   VAVSVDEGDPQSDAIYSISDNEDE--YQNELLQEESVF------------
117 YP_009508408.1   EIVSVQEGDNQSDAIYSVSEGEVADDVQQGVNVFIQK-------------
118 NP_955579.1      ---TLFQWGTEQQL------------------------------------
119 YP_009229919.1   DVVSVDVGDPDSDGICSFSEGEIK--NYATKAVLEDDF------------
```

```
120 YP_004442833.1     DIVSVGYDEKSLSDIFSISEGEDNQAHL-RD-EEG-LPEVP---------
121 NP_862833.2        AS--PRILSPEGRS------------------------------------
122 NP_040563.1        QE--KIQLTPEAIK------------------------------------
123 YP_443922.1        NLHASLIWTPEATC------------------------------------
124 NP_057933.2        ---TLFNWGPDQQK------------------------------------
125 YP_009116631.1     DVVSVEPGEEDDDEICSISEGENTGGMHELMAFKTQLPYPV---------
126 YP_009513249.1     ---NYVPWQIEHST------------------------------------
127 YP_223871.1        ---DPLVWGEKEEE------------------------------------
128 NP_127504.1        EDEVDGMTEEPIPERRPEITPPKMVGTGFHTFSLDDISITKWP-------
129 NP_044929.1        ---QCIPWTMDHTT------------------------------------
130 NP_057860.1        RD--QIYLNPSQVQ------------------------------------
131 NP_040973.1        NS--TRQWTKEAR-------------------------------------
132 NP_777317.1        DVVSVGFDEDDVSDIYSVGEEIDEYQFTHEN-------------------
133 NP_054716.1        ---IYIPWETKHTA------------------------------------
134 YP_001856242.1     TE--VIPLTEEAEL------------------------------------
135 NP_954565.2        NS--HRSLSKEALA------------------------------------
136 NP_569150.1        DVISVGFDEDDVSDIYSIDEEADNYRFTNEE-------------------
137 NP_659397.1        --------------------------------------------------
138 YP_009513211.1     ---VPFTWTEAHQE------------------------------------
139 YP_233107.1        EVVSVGMDESDVSDIYSISENEDNHQITEE--------------------
140 YP_009109692.1     ---TPFTWQTEHQL------------------------------------
141 NP_057849.4        TE--VIPLTEEAEL------------------------------------
142 NP_049560.1        RD--QICLNPSQVQ------------------------------------
143 YP_009506267.1     DIVSVHEGEEQSDAIYSVSEGE-NESIDQEVNTFISYGEGG---------
144 NP_039820.1        EVVSADDKEEEDDEIFSVLGEEDYQEETIMVLEED---------------
145 NP_056790.1        ---IPFIWTEEHQQ------------------------------------
146 YP_002321513.1     DVVSVDMGEPDSDHIVSMSDGVGPEATRKYLDSQFTLP------------
147 NP_955591.1        ---TLFNWGPDQQK------------------------------------
148 YP_001956722.2     ---KYITWTTDNSQ------------------------------------
149 NP_056902.1        NQ--KVIWTEEAQK------------------------------------
150 NP_056886.1        NE--AREWNLDMKM------------------------------------
151 NP_041261.1        LS--VRALTPEAKQ------------------------------------
```

```
001 YP_004442836.1     --------------------------------------------------
002 NP_056728.1        --------------------------------------------------
003 YP_009508537.1     --------------------------------------------------
004 YP_004732983.2     --------------------------------------------------
005 YP_009508407.1     --------------------------------------------------
006 NP_040939.1        --------------------------------------------------
007 YP_003864102.1     --------------------------------------------------
008 YP_009002585.1     --------------------------------------------------
009 YP_003284237.1     --------------------------------------------------
010 YP_009268869.1     --------------------------------------------------
011 YP_009506251.1     --------------------------------------------------
012 NP_955611.1        --------------------------------------------------
013 YP_008567619.1     --------------------------------------------------
014 NP_149413.1        --------------------------------------------------
015 YP_004442824.1     --------------------------------------------------
016 YP_001931967.1     --------------------------------------------------
017 YP_002455786.1     --------------------------------------------------
018 YP_009109694.1     --------------------------------------------------
019 NP_789739.1        --------------------------------------------------
020 NP_955619.1        --------------------------------------------------
021 YP_224289.1        --------------------------------------------------
022 YP_009182100.1     NEYNLAYYNLGIFNNNTYRDMFIQKLPDPWKEYLTNERAKEAGNEEWTLG
023 NP_705927.1        --------------------------------------------------
024 YP_009508577.1     --------------------------------------------------
025 YP_004442830.1     --------------------------------------------------
026 NP_758887.1        --------------------------------------------------
027 YP_009508406.1     --------------------------------------------------
028 YP_610965.1        --------------------------------------------------
029 YP_001931961.1     --------------------------------------------------
030 YP_241114.1        --------------------------------------------------
031 YP_006732334.1     --------------------------------------------------
032 YP_009041481.1     --------------------------------------------------
033 YP_001036293.1     --------------------------------------------------
034 YP_605811.1        --------------------------------------------------
035 NP_955577.1        --------------------------------------------------
036 YP_003208050.1     --------------------------------------------------
037 NP_789740.1        --------------------------------------------------
038 YP_004442839.1     --------------------------------------------------
```

```
039 YP_009507791.1    --------------------------------------------------
040 YP_004581513.1    --------------------------------------------------
041 NP_043933.1       --------------------------------------------------
042 YP_009507248.1    --------------------------------------------------
043 YP_002117531.1    --------------------------------------------------
044 YP_004347415.1    --------------------------------------------------
045 NP_056891.1       --------------------------------------------------
046 YP_009513242.1    --------------------------------------------------
047 YP_009352866.1    --------------------------------------------------
048 YP_009165750.1    --------------------------------------------------
049 NP_040840.1       --------------------------------------------------
050 YP_004222728.1    --------------------------------------------------
051 YP_009506264.1    --------------------------------------------------
052 YP_567050.1       --------------------------------------------------
053 YP_009508571.1    --------------------------------------------------
054 NP_056803.1       --------------------------------------------------
055 NP_955564.1       --------------------------------------------------
056 YP_009508411.1    --------------------------------------------------
057 YP_009140788.1    --------------------------------------------------
058 YP_009508410.1    --------------------------------------------------
059 NP_040333.1       --------------------------------------------------
060 YP_008992013.1    --------------------------------------------------
061 NP_041734.1       --------------------------------------------------
062 NP_056895.1       --------------------------------------------------
063 YP_009109689.1    --------------------------------------------------
064 YP_002519387.1    --------------------------------------------------
065 YP_595725.1       --------------------------------------------------
066 YP_009508551.1    --------------------------------------------------
067 YP_001497148.1    --------------------------------------------------
068 NP_041186.1       --------------------------------------------------
069 NP_663784.1       --------------------------------------------------
070 YP_009508443.1    --------------------------------------------------
071 YP_003987465.1    --------------------------------------------------
072 NP_861410.1       --------------------------------------------------
073 YP_009345071.1    YEYRHFGTCTSDIQQPQPWKQQTKVYEEGGNGHDRAFYRYYGPPKGRENW
074 YP_009508582.1    --------------------------------------------------
075 NP_569141.1       --------------------------------------------------
076 YP_002916057.1    --------------------------------------------------
077 NP_777384.2       --------------------------------------------------
078 YP_233110.1       --------------------------------------------------
079 NP_542258.1       --------------------------------------------------
080 YP_009130664.1    --------------------------------------------------
081 YP_009508566.1    --------------------------------------------------
082 YP_006607892.1    --------------------------------------------------
083 YP_009506270.1    --------------------------------------------------
084 NP_042513.1       --------------------------------------------------
085 YP_006907834.1    --------------------------------------------------
086 YP_004442827.1    --------------------------------------------------
087 YP_009408594.1    --------------------------------------------------
088 YP_009508561.1    --------------------------------------------------
089 NP_056907.1       --------------------------------------------------
090 YP_002308474.1    --------------------------------------------------
091 YP_007761644.1    --------------------------------------------------
092 NP_040550.1       --------------------------------------------------
093 NP_758808.1       --------------------------------------------------
094 YP_009345075.1    --------------------------------------------------
095 NP_068729.1       --------------------------------------------------
096 NP_047255.1       --------------------------------------------------
097 YP_006273075.1    --------------------------------------------------
098 YP_009508888.1    --------------------------------------------------
099 NP_395469.1       --------------------------------------------------
100 YP_009508546.1    --------------------------------------------------
101 NP_056762.1       --------------------------------------------------
102 YP_009508556.1    --------------------------------------------------
103 NP_569153.1       --------------------------------------------------
104 NP_043924.1       --------------------------------------------------
105 YP_009243641.1    --------------------------------------------------
106 YP_004300274.1    --------------------------------------------------
107 NP_056848.1       --------------------------------------------------
108 YP_006495799.1    --------------------------------------------------
109 YP_009508409.1    --------------------------------------------------
110 NP_687035.1       --------------------------------------------------
111 NP_045937.2       --------------------------------------------------
```

```
112 NP_612577.1         --------------------------------------------------
113 NP_619548.1         --------------------------------------------------
114 NP_056880.1         --------------------------------------------------
115 YP_009121747.1      --------------------------------------------------
116 YP_009113237.2      --------------------------------------------------
117 YP_009508408.1      --------------------------------------------------
118 NP_955579.1         --------------------------------------------------
119 YP_009229919.1      --------------------------------------------------
120 YP_004442833.1      --------------------------------------------------
121 NP_862833.2         --------------------------------------------------
122 NP_040563.1         --------------------------------------------------
123 YP_443922.1         --------------------------------------------------
124 NP_057933.2         --------------------------------------------------
125 YP_009116631.1      --------------------------------------------------
126 YP_009513249.1      --------------------------------------------------
127 YP_223871.1         --------------------------------------------------
128 NP_127504.1         --------------------------------------------------
129 NP_044929.1         --------------------------------------------------
130 NP_057860.1         --------------------------------------------------
131 NP_040973.1         --------------------------------------------------
132 NP_777317.1         --------------------------------------------------
133 NP_054716.1         --------------------------------------------------
134 YP_001856242.1      --------------------------------------------------
135 NP_954565.2         --------------------------------------------------
136 NP_569150.1         --------------------------------------------------
137 NP_659397.1         --------------------------------------------------
138 YP_009513211.1      --------------------------------------------------
139 YP_233107.1         --------------------------------------------------
140 YP_009109692.1      --------------------------------------------------
141 NP_057849.4         --------------------------------------------------
142 NP_049560.1         --------------------------------------------------
143 YP_009506267.1      --------------------------------------------------
144 NP_039820.1         --------------------------------------------------
145 NP_056790.1         --------------------------------------------------
146 YP_002321513.1      --------------------------------------------------
147 NP_955591.1         --------------------------------------------------
148 YP_001956722.2      --------------------------------------------------
149 NP_056902.1         --------------------------------------------------
150 NP_056886.1         --------------------------------------------------
151 NP_041261.1         --------------------------------------------------


001 YP_004442836.1      -------------------------------MEDFKNYEVYMMEAEELDE
002 NP_056728.1         --------------------------------------------------
003 YP_009508537.1      --------------------------------------------------
004 YP_004732983.2      --------------------------EAQYEQHQALVVIQTTFKKEDKPQ
005 YP_009508407.1      --------------------------------------------------
006 NP_040939.1         --------------------------------------------------
007 YP_003864102.1      --------------------------------------------------
008 YP_009002585.1      -------------------------------MFHEGTDNVYWAGKKDG--
009 YP_003284237.1      -------------------------------EEEVAAMVFALEDEEED--
010 YP_009268869.1      --------------------------------------------------
011 YP_009506251.1      -------------------------------MIREG-DQSYWMGKANH--
012 NP_955611.1         --------------------------------------------------
013 YP_008567619.1      --------------------------HYINVFFEED--RTYWVGKAGS--
014 NP_149413.1         --------------------------TSFEEWEEYYKEEFTLMAEVDDS-
015 YP_004442824.1      -------------------------------FEDFTNYEVYMIHVEEI--
016 YP_001931967.1      --------------------------------------------------
017 YP_002455786.1      --------------------------------------------------
018 YP_009109694.1      --------------------------------------------------
019 NP_789739.1         --------------------------------------------------
020 NP_955619.1         --------------------------------------------------
021 YP_224289.1         -------------------------------MSLELFMLHEIEDEPEA--
022 YP_009182100.1      NLIEFVKTKLADLAIQAWRAKRMQKLYSKSGLSFCQHLIDIPTQWGGRNK
023 NP_705927.1         --------------------------------------------------
024 YP_009508577.1      --------------------------------------------------
025 YP_004442830.1      --------------------------LSWEDWENYYKKEFLYMAEIEEVS
026 NP_758887.1         --------------------------------------------------
027 YP_009508406.1      --------------------------------------------------
028 YP_610965.1         -------------------------FLEGSFMLKETV-------------
029 YP_001931961.1      --------------------------------------------------
030 YP_241114.1         --------------------------------------------------
```

```
031 YP_006732334.1    -------------------------------------------------
032 YP_009041481.1    -------------------------EPTNSFNFGLVLIQGPVPDVNAS-
033 YP_001036293.1    --------------------------NLLYIREED--GKHFLGGGG---
034 YP_605811.1       ------------------------------FDVIG-HDVFMFTIEEQ--
035 NP_955577.1       -------------------------------------------------
036 YP_003208050.1    ------------------------YET-ALMLQSQN-------------
037 NP_789740.1       -------------------------------------------------
038 YP_004442839.1    ------------------------------FGVVD-HDVFMITIEE---
039 YP_009507791.1    -------------------------------------------------
040 YP_004581513.1    -------------------------------------------------
041 NP_043933.1       -------------------------------------------------
042 YP_009507248.1    -------------------------------------------------
043 YP_002117531.1    ------------------------------LHDETIFSLTEEDDEAA--
044 YP_004347415.1    -------------------------------------------------
045 NP_056891.1       -------------------------------------------------
046 YP_009513242.1    -------------------------------------------------
047 YP_009352866.1    -------------------------EGAG-INLGFVMIQGPIPNANEN-
048 YP_009165750.1    -------------------------------------------------
049 NP_040840.1       -------------------------------------------------
050 YP_004222728.1    -------------------------------------------------
051 YP_009506264.1    ------------------------------IEEIYALEDEMEPEQ----
052 YP_567050.1       -------------------------------------------------
053 YP_009508571.1    -------------------------------------------------
054 NP_056803.1       -------------------------------------------------
055 NP_955564.1       -------------------------------------------------
056 YP_009508411.1    --------------------------SLFYLRAED--NTYWLGGGG---
057 YP_009140788.1    ------------------------------RSLQTMEIVLMLGEVDGG-
058 YP_009508410.1    -------------------------------------------------
059 NP_040333.1       -------------------------------------------------
060 YP_008992013.1    -------------------------DRLNEMSAWIDLQMLRSGATTES-
061 NP_041734.1       ------------------------------MMREE-DQSYWLGKTNH--
062 NP_056895.1       -------------------------------------------------
063 YP_009109689.1    -------------------------------------------------
064 YP_002519387.1    -------------------------------------------------
065 YP_595725.1       -------------------------QTFEEWEEYYKDEFIMMADIEESE
066 YP_009508551.1    -------------------------------------------------
067 YP_001497148.1    -------------------------------------------------
068 NP_041186.1       -------------------------------------------------
069 NP_663784.1       -------------------------------------------------
070 YP_009508443.1    -------------------------ETLYYLREED--HTYFLGGGG---
071 YP_003987465.1    -------------------------------THEAYHVFSLVEDC----
072 NP_861410.1       -------------------------------------------------
073 YP_009345071.1    PETPIPTGWGSDFDHDPDKEIDDIPDDEEIYMMQDDQFDEHWIGKRG--
074 YP_009508582.1    -------------------------------------------------
075 NP_569141.1       -------------------------------------------------
076 YP_002916057.1    -------------------------------------------------
077 NP_777384.2       -------------------------------------------------
078 YP_233110.1       ------------------------------FDVIG-HDVFMFTIEEQ--
079 NP_542258.1       -------------------------------------------------
080 YP_009130664.1    -------------------------EHLYYFREED--QTYWIGGEG---
081 YP_009508566.1    -------------------------------------------------
082 YP_006607892.1    -------------------------------------------------
083 YP_009506270.1    ------------------------------MEDFKNYEVYMMNLEEIEE
084 NP_042513.1       -------------------------------------------------
085 YP_006907834.1    -------------------------------------------------
086 YP_004442827.1    -------------------------TTWEDWDSYYKKEFLMMAEVLEVS
087 YP_009408594.1    ------------------------GIEGLFMLGAPE-------------
088 YP_009508561.1    -------------------------------------------------
089 NP_056907.1       -------------------------------------------------
090 YP_002308474.1    -------------------------------------------------
091 YP_007761644.1    -------------------------------------------------
092 NP_040550.1       -------------------------------------------------
093 NP_758808.1       ------------------------FLSDADEFESPQQHLHETVNMLQSR
094 YP_009345075.1    --------------------------ESIFMLREELEHKPP--------
095 NP_068729.1       -------------------------------------------------
096 NP_047255.1       -------------------------------------------------
097 YP_006273075.1    ------------------------------KSLQTLEIVLMLGEIDGG-
098 YP_009508888.1    -------------------------------------------------
099 NP_395469.1       -------------------------------------------------
100 YP_009508546.1    -------------------------------------------------
101 NP_056762.1       -------------------------------------------------
102 YP_009508556.1    -------------------------------------------------
103 NP_569153.1       ------------------------------VFQEECNGTYWLGKRGG--
```

```
104 NP_043924.1         --------------------------------------------------
105 YP_009243641.1      --------------------------------------------------
106 YP_004300274.1      --------------------------------------------------
107 NP_056848.1         --------------------------------------------------
108 YP_006495799.1      --------------------------EFEQG-MFVLTIEHVTAPAVASG-
109 YP_009508409.1      --------------------------------------------------
110 NP_687035.1         --------------------------------------------------
111 NP_045937.2         --------------------------------------------------
112 NP_612577.1         --------------------------------------------------
113 NP_619548.1         --------------------------------------------------
114 NP_056880.1         --------------------------------------------------
115 YP_009121747.1      -------------------------LEEYCFMFHQSHEEIHKRVQEAVS-
116 YP_009113237.2      -------------------------------VIVEKTESRKYLLGKEGS-
117 YP_009508408.1      --------------------------ETLYYLRQED--NAYFLGGGG---
118 NP_955579.1         --------------------------------------------------
119 YP_009229919.1      -------------------------LDELAFVVTEKKEIDY---------
120 YP_004442833.1      --------------------------TTWDQWDEYYKKEFIYMADTKEVL
121 NP_862833.2         --------------------------------------------------
122 NP_040563.1         --------------------------------------------------
123 YP_443922.1         --------------------------------------------------
124 NP_057933.2         --------------------------------------------------
125 YP_009116631.1      --------------------------EYEAS-TPQFLMPWTQVPVEKSDK
126 YP_009513249.1      --------------------------------------------------
127 YP_223871.1         --------------------------------------------------
128 NP_127504.1         --------------------------ERIQDFHTWMLTKQLVE-REPFL-
129 NP_044929.1         --------------------------------------------------
130 NP_057860.1         --------------------------------------------------
131 NP_040973.1         --------------------------------------------------
132 NP_777317.1         -------------------------------LEDFKNYEVNFLEDHEEDE
133 NP_054716.1         --------------------------------------------------
134 YP_001856242.1      --------------------------------------------------
135 NP_954565.2         --------------------------------------------------
136 NP_569150.1         -------------------------------MEGFKNYEVYMLRMEEMDE
137 NP_659397.1         --------------------------------------------------
138 YP_009513211.1      --------------------------------------------------
139 YP_233107.1         -------------------------------------WDVLMLQEEEIPE
140 YP_009109692.1      --------------------------------------------------
141 NP_057849.4         --------------------------------------------------
142 NP_049560.1         --------------------------------------------------
143 YP_009506267.1      -----------------TGSTTHFSNQTLYYLKEEGTVTHYYLGGGG---
144 NP_039820.1         --------------------------DIQQIIKEFSKFGDLSRRNVGPNF
145 NP_056790.1         --------------------------------------------------
146 YP_002321513.1      ------------------------WATEFIGVFHVTTDTLSMNENLG---
147 NP_955591.1         --------------------------------------------------
148 YP_001956722.2      --------------------------------------------------
149 NP_056902.1         --------------------------------------------------
150 NP_056886.1         --------------------------------------------------
151 NP_041261.1         --------------------------------------------------


001 YP_004442836.1      --------------------------------------------------
002 NP_056728.1         --------------------------------------------------
003 YP_009508537.1      --------------------------------------------------
004 YP_004732983.2      --------------------------------------------------
005 YP_009508407.1      --------------------------------------------------
006 NP_040939.1         --------------------------------------------------
007 YP_003864102.1      --------------------------------------------------
008 YP_009002585.1      --------------------------------------------------
009 YP_003284237.1      --------------------------------------------------
010 YP_009268869.1      --------------------------------------------------
011 YP_009506251.1      --------------------------------------------------
012 NP_955611.1         --------------------------------------------------
013 YP_008567619.1      --------------------------------------------------
014 NP_149413.1         -SDEEK--------------------------------------------
015 YP_004442824.1      --------------------------------------------------
016 YP_001931967.1      --------------------------------------------------
017 YP_002455786.1      --------------------------------------------------
018 YP_009109694.1      --------------------------------------------------
019 NP_789739.1         --------------------------------------------------
020 NP_955619.1         --------------------------------------------------
021 YP_224289.1         --------------------------------------------------
022 YP_009182100.1      ELKHKKKGKKIKSFKRYKFRKSKKKGFEKSKINPQKNFKRRFKQKSGKEI
```

```
023 NP_705927.1       --------------------------------------------------
024 YP_009508577.1    --------------------------------------------------
025 YP_004442830.1    SEEEKED-------------------------------------------
026 NP_758887.1       --------------------------------------------------
027 YP_009508406.1    --------------------------------------------------
028 YP_610965.1       --------------------------------------------------
029 YP_001931961.1    --------------------------------------------------
030 YP_241114.1       --------------------------------------------------
031 YP_006732334.1    --------------------------------------------------
032 YP_009041481.1    --------------------------------------------------
033 YP_001036293.1    --------------------------------------------------
034 YP_605811.1       --------------------------------------------------
035 NP_955577.1       --------------------------------------------------
036 YP_003208050.1    --------------------------------------------------
037 NP_789740.1       --------------------------------------------------
038 YP_004442839.1    --------------------------------------------------
039 YP_009507791.1    --------------------------------------------------
040 YP_004581513.1    --------------------------------------------------
041 NP_043933.1       --------------------------------------------------
042 YP_009507248.1    --------------------------------------------------
043 YP_002117531.1    --------------------------------------------------
044 YP_004347415.1    --------------------------------------------------
045 NP_056891.1       --------------------------------------------------
046 YP_009513242.1    --------------------------------------------------
047 YP_009352866.1    --------------------------------------------------
048 YP_009165750.1    --------------------------------------------------
049 NP_040840.1       --------------------------------------------------
050 YP_004222728.1    --------------------------------------------------
051 YP_009506264.1    --------------------------------------------------
052 YP_567050.1       --------------------------------------------------
053 YP_009508571.1    --------------------------------------------------
054 NP_056803.1       --------------------------------------------------
055 NP_955564.1       --------------------------------------------------
056 YP_009508411.1    --------------------------------------------------
057 YP_009140788.1    --------------------------------------------------
058 YP_009508410.1    --------------------------------------------------
059 NP_040333.1       --------------------------------------------------
060 YP_008992013.1    --------------------------------------------------
061 NP_041734.1       --------------------------------------------------
062 NP_056895.1       --------------------------------------------------
063 YP_009109689.1    --------------------------------------------------
064 YP_002519387.1    --------------------------------------------------
065 YP_595725.1       NSDEEK--------------------------------------------
066 YP_009508551.1    --------------------------------------------------
067 YP_001497148.1    --------------------------------------------------
068 NP_041186.1       --------------------------------------------------
069 NP_663784.1       --------------------------------------------------
070 YP_009508443.1    --------------------------------------------------
071 YP_003987465.1    --------------------------------------------------
072 NP_861410.1       --------------------------------------------------
073 YP_009345071.1    --------------------------------------------------
074 YP_009508582.1    --------------------------------------------------
075 NP_569141.1       --------------------------------------------------
076 YP_002916057.1    --------------------------------------------------
077 NP_777384.2       --------------------------------------------------
078 YP_233110.1       --------------------------------------------------
079 NP_542258.1       --------------------------------------------------
080 YP_009130664.1    --------------------------------------------------
081 YP_009508566.1    --------------------------------------------------
082 YP_006607892.1    --------------------------------------------------
083 YP_009506270.1    --------------------------------------------------
084 NP_042513.1       --------------------------------------------------
085 YP_006907834.1    --------------------------------------------------
086 YP_004442827.1    SDEEKE--------------------------------------------
087 YP_009408594.1    --------------------------------------------------
088 YP_009508561.1    --------------------------------------------------
089 NP_056907.1       --------------------------------------------------
090 YP_002308474.1    --------------------------------------------------
091 YP_007761644.1    --------------------------------------------------
092 NP_040550.1       --------------------------------------------------
093 NP_758808.1       --------------------------------------------------
094 YP_009345075.1    --------------------------------------------------
095 NP_068729.1       --------------------------------------------------
```

096 NP_047255.1 --
097 YP_006273075.1 --
098 YP_009508888.1 --
099 NP_395469.1 --
100 YP_009508546.1 --
101 NP_056762.1 --
102 YP_009508556.1 --
103 NP_569153.1 --
104 NP_043924.1 --
105 YP_009243641.1 --
106 YP_004300274.1 --
107 NP_056848.1 --
108 YP_006495799.1 --
109 YP_009508409.1 --
110 NP_687035.1 --
111 NP_045937.2 --
112 NP_612577.1 --
113 NP_619548.1 --
114 NP_056880.1 --
115 YP_009121747.1 --
116 YP_009113237.2 --
117 YP_009508408.1 --
118 NP_955579.1 --
119 YP_009229919.1 --
120 YP_004442833.1 SEEENE--
121 NP_862833.2 --
122 NP_040563.1 --
123 YP_443922.1 --
124 NP_057933.2 --
125 YP_009116631.1 --
126 YP_009513249.1 --
127 YP_223871.1 --
128 NP_127504.1 --
129 NP_044929.1 --
130 NP_057860.1 --
131 NP_040973.1 --
132 NP_777317.1 RWDTLGEPSGKFDYYVKYSAPKHHQLMEEIIPSGWDTEEEEEEYPESVEE
133 NP_054716.1 --
134 YP_001856242.1 --
135 NP_954565.2 --
136 NP_569150.1 --
137 NP_659397.1 --
138 YP_009513211.1 --
139 YP_233107.1 --
140 YP_009109692.1 --
141 NP_057849.4 --
142 NP_049560.1 --
143 YP_009506267.1 --
144 NP_039820.1 PG--
145 NP_056790.1 --
146 YP_002321513.1 --
147 NP_955591.1 --
148 YP_001956722.2 --
149 NP_056902.1 --
150 NP_056886.1 --
151 NP_041261.1 --

001 YP_004442836.1 ----------PKEYLVGEPSEWRSKMRVSRRQYYCK-HDWKFEE----SH
002 NP_056728.1 --
003 YP_009508537.1 ---T
004 YP_004732983.2 -------------------GSWRMSKPIPETQQQCQ-HTWDDMYALAEGQ
005 YP_009508407.1 --
006 NP_040939.1 ---E
007 YP_003864102.1 ---A
008 YP_009002585.1 ---------------------YLPYKRITREQFQCE-HEWQMNQEVATVE
009 YP_003284237.1 -------------TLIGKPGTWRSQVRVTRKEYECI-HLWDFNS----EG
010 YP_009268869.1 ---E
011 YP_009506251.1 ---------------------WTAMVRVSYQQYHCP-HQWEHNKEIPIMA
012 NP_955611.1 ---A
013 YP_008567619.1 ---------------------WQPMIKVTQKVHDCQ-HCWMENTDVPNDG
014 NP_149413.1 -----------GPFLVGPKDGFRHQMIVSQKQYKCT-HDWEYQK----DR

```
015 YP_004442824.1    ----------PREFLVGEPSDWRSKMKVSINQYYCI-HNWKFDQ----ES
016 YP_001931967.1    --------------------------------------------------
017 YP_002455786.1    -------------------------------------------------A
018 YP_009109694.1    -------------------------------------------------A
019 NP_789739.1       -------------------------------------------------E
020 NP_955619.1       -------------------------------------------------S
021 YP_224289.1       -------------YLIGATDTWRNQMKVSRKEYYCQ-HEWDHTT----KV
022 YP_009182100.1    EDRKKQSKSKPTSSKGVANVCWNCNQQGHFSRECPRKNARLIELQEAADT
023 NP_705927.1       -------------------------------------------------E
024 YP_009508577.1    -------------------------------------------------Y
025 YP_004442830.1    ----------NGPWLVGKEGGFMYQMKVSRKQYHCQ-HEWFYNQ----PK
026 NP_758887.1       -------------------------------------------------E
027 YP_009508406.1    --------------------------------------------------
028 YP_610965.1       --------------------SWRPTVSLPANQANCQ-HLWEDNS-QSTGH
029 YP_001931961.1    --------------------------------------------------
030 YP_241114.1       -------------------------------------------------A
031 YP_006732334.1    --------------------------------------------------
032 YP_009041481.1    --------------------SWMPRHPLEGLQLTCI-HVWEENAPIQATE
033 YP_001036293.1    ---------------------WRAAVQVTPQEHDCH-HQWEYYI----LN
034 YP_605811.1       -----------RNCLVETTSAWRSAMKVTSEEKNCL-HTWSFEE----KT
035 NP_955577.1       -------------------------------------------------A
036 YP_003208050.1    --------------------SWRRTVKLPAYQDCCG-HQPTLGPNLTFEQ
037 NP_789740.1       -------------------------------------------------E
038 YP_004442839.1    -----------RNYLVPTTSEWRAAMKVTKEEYLCL-HQWEFSS----ER
039 YP_009507791.1    -------------------------------------LFQQIDQEQALEE
040 YP_004581513.1    --------------------------------------------------
041 NP_043933.1       --------------------------------------------------
042 YP_009507248.1    -------------------------------------------------A
043 YP_002117531.1    -------------LLVGKAGTWRPQMKVSKKEFSCE-HEWDYEV----SG
044 YP_004347415.1    --------------------------------------------------
045 NP_056891.1       -------------------------------------------------S
046 YP_009513242.1    -------------------------------------------------Q
047 YP_009352866.1    --------------------SWMVRYPLEGSQQSCD-HQWEEGAAIPQGE
048 YP_009165750.1    --------------------------------------------------
049 NP_040840.1       -------------------------------------------------E
050 YP_004222728.1    -------------------------------------------------A
051 YP_009506264.1    -------------LLIGKPNTWRTQIRVSRKEFHCL-HEWDFTK----TE
052 YP_567050.1       -------------------------------------------------A
053 YP_009508571.1    -------------------------------------------------Q
054 NP_056803.1       -------------------------------------------------Q
055 NP_955564.1       -------------------------------------------------A
056 YP_009508411.1    ---------------------WRFAVKVTPQEHNCA-HSWSYYE----EQ
057 YP_009140788.1    ---------------------YRPQIKLPDEQFNCA-HNWVHNGEILVQK
058 YP_009508410.1    --------------------------------------------------
059 NP_040333.1       -------------------------------------------------A
060 YP_008992013.1    ------------------------VLREFATRFTGALRDWFDSLGPYRQL
061 NP_041734.1       ---------------------WTAMVRVSSQQYHCM-HQWEHNKEILVVA
062 NP_056895.1       -------------------------------------------------G
063 YP_009109689.1    -------------------------------------------------A
064 YP_002519387.1    --------------------------------------------------
065 YP_595725.1       -----------GPFLVGPKGGFRHQMEVSYKQYKCE-HDWDFTR----TR
066 YP_009508551.1    -------------------------------------------------Q
067 YP_001497148.1    -------------------------------------------------A
068 NP_041186.1       -------------------------------------------------A
069 NP_663784.1       -------------------------------------------------E
070 YP_009508443.1    ---------------------WRCSVKVSQQEHDCK-HRWDYYT----NT
071 YP_003987465.1    --------------LIGKANTWRYQVRVSPKEYYCK-HEWGFNS----KI
072 NP_861410.1       --------------------------------------------------
073 YP_009345071.1    ---------------------WQPLIKLSDQEFNCV-HHWQHNIQVP-DC
074 YP_009508582.1    -------------------------------------------------H
075 NP_569141.1       --------------------------------------------------
076 YP_002916057.1    --------------------------------------------------
077 NP_777384.2       -------------------------------------------------G
078 YP_233110.1       -----------RNCLVETTSAWRSAMKVTPEEKNCL-HTWSFEE----KT
079 NP_542258.1       -------------------------------------------------A
080 YP_009130664.1    ---------------------YRSMIKATKTQHDCN-HVWEYN----GAG
081 YP_009508566.1    -------------------------------------------------A
082 YP_006607892.1    --------------------------------------------------
083 YP_009506270.1    ----------PNDYLVGEPSDWRSKMKVSRRQYFCK-HEWSFEE----AH
084 NP_042513.1       --------------------------------------------------
085 YP_006907834.1    --------------------------------------------------
086 YP_004442827.1    ----------EGPWLVGKEGGYMQQMYVTKKQFHCT-HDWYYDQ----PR
087 YP_009408594.1    --------------------GSRVQIKVSDEILNCV-HQWQMNEDVDVLY
```

```
088 YP_009508561.1  -------------------------------------------------R
089 NP_056907.1     -------------------------------------------------A
090 YP_002308474.1  -------------------------------------------------A
091 YP_007761644.1  --------------------------------------------------
092 NP_040550.1     -------------------------------------------------A
093 NP_758808.1     -------------------SAYLPQVAVGEEKLNCS-HIWLQDVDIPSDK
094 YP_009345075.1  --------------------SWFYKMPIPKEYQECL-HAWEANAPIVQGR
095 NP_068729.1     --------------------------------------------------
096 NP_047255.1     -------------------------------------------------A
097 YP_006273075.1  ---------------------YRPQIKLPEEQMNCV-HNWIHNGDIFVQR
098 YP_009508888.1  -------------------------------------------------Q
099 NP_395469.1     --------------------------------------------------
100 YP_009508546.1  -------------------------------------------------Q
101 NP_056762.1     ------------------------------------------------KK
102 YP_009508556.1  -------------------------------------------------Q
103 NP_569153.1     ---------------------YQDLVQISKEIYYCQ-HEWEENQPINDPA
104 NP_043924.1     ------------------------IRQGHFLCSLVQKDDNGMWHVLGFYS
105 YP_009243641.1  -------------------------------------------------E
106 YP_004300274.1  --------------------------------------------------
107 NP_056848.1     --------------------------------------------------
108 YP_006495799.1  ---------------------WRRRTELRGDQKVCP-HQWSDVAEVSLED
109 YP_009508409.1  --------------------------------------------------
110 NP_687035.1     -------------------------------------------------E
111 NP_045937.2     --------------------------------------------------
112 NP_612577.1     --------------------------------------------------
113 NP_619548.1     --------------------------------------------------
114 NP_056880.1     -------------------------------------------------A
115 YP_009121747.1  ---------------------WKPRMELPKGSKECE-HKWQENQ--VTCH
116 YP_009113237.2  ---------------------YLPWVAVKRKQYKCD-HEWTQGRDACLTE
117 YP_009508408.1  ---------------------WRCAVRVTQQEHDCR-HQWEYYS----PT
118 NP_955579.1     -------------------------------------------------A
119 YP_009229919.1  --------------------GWRYKVVLTKDQERCR-HTLEANCEVEAGK
120 YP_004442833.1  ----------NGPWLVGQPGGFEKQMYVTKKQYHCEGHEWFHNQ----PK
121 NP_862833.2     -------------------------------------------------A
122 NP_040563.1     -------------------------------------------------C
123 YP_443922.1     -------------------------------------------------A
124 NP_057933.2     -------------------------------------------------A
125 YP_009116631.1  -------------------PSWRRRKDISQVQKDCT-HTWSDTQEVPISD
126 YP_009513249.1  -------------------------------------------------T
127 YP_223871.1     -------------------------------------------------A
128 NP_127504.1     ------------------------ILSEFTARLSGTLREWWNSVGPDDKN
129 NP_044929.1     -------------------------------------------------Q
130 NP_057860.1     -------------------------------------------------S
131 NP_040973.1     ------------------------------------------------LE
132 NP_777317.1     HDVFMLREDEPDDYLVGNPPSWRSKLRVSRQQYYCQ-HNWQYDH----KE
133 NP_054716.1     -------------------------------------------------I
134 YP_001856242.1  -------------------------------------------------E
135 NP_954565.2     -------------------------------------------------S
136 NP_569150.1     ----------PREYLVGEPSEWRSKMKVSRRQYFCK-HEWKFEE----TH
137 NP_659397.1     --------------------------------------------------
138 YP_009513211.1  -------------------------------------------------A
139 YP_233107.1     ----------PSEYYIGEPG-WRSQMKVSKTEFHCQ-HEWKFDK----ED
140 YP_009109692.1  -------------------------------------------------A
141 NP_057849.4     -------------------------------------------------E
142 NP_049560.1     -------------------------------------------------S
143 YP_009506267.1  ---------------------TKVSVKVTQKEHDCT-HDWVLYG----EN
144 NP_039820.1     -------PAEVQMGVLKPKSSWRRPIQATLEEINCH-HNWTAIS----TG
145 NP_056790.1     -------------------------------------------------A
146 YP_002321513.1  ---------------------WRSKPKLSDDQLLCD-HEGFKICAKPAEA
147 NP_955591.1     -------------------------------------------------A
148 YP_001956722.2  -------------------------------------------------Q
149 NP_056902.1     -------------------------------------------------E
150 NP_056886.1     -------------------------------------------------A
151 NP_041261.1     -------------------------------------------------S


001 YP_004442836.1  RTICRACGSEAVHKHRIDCLKCDMTICLMCQPWFYKDINTEEVKK-----
002 NP_056728.1     --------------------------------------------------
003 YP_009508537.1  LETLITKLNEAEYLQGRRGDKTLIMKVNASY--TTGYIRYYNEGEK----
004 YP_004732983.2  -QACSTCQTITVLGRRATCTLCLLNLCSLCAGLDFGLKIVPKTA------
005 YP_009508407.1  --------------------------------------------------
006 NP_040939.1     WEACRKKLEEMEGN-YYNKDKDVYGQLAWG-DKAIEYIVYQEKGKP----
```

007	YP_003864102.1	LDKVETAIAE-QFVTHINYSQPLILLVFNTALTPTGLFWQSN--------
008	YP_009002585.1	LEKCSFCKQETRRRMRLHCPKCRITSCVLCSSNYCGIAIKPE-PV-----
009	YP_003284237.1	YEKCKACRLEARANERMKCKECKMVICCLCSNYCFDITIPRE-------R
010	YP_009268869.1	WEECRRKLAEMEGN-YYDEEKDVYGQIDWG-DKAIEYIVFQERGKP----
011	YP_009506251.1	LINCHFCKQPTNLRGRVHCPTCQLTSCYMCAPIYCDVQVQQQ-PK-----
012	NP_955611.1	WREIVRLSTT-AALERWDPALPLEGAVARCEQGAIGVLGQGLSTHP----
013	YP_008567619.1	PIQCLCCKRNSQKNFRIHCPICKVTSCGLCSTFCFDKSIQVKKEY-----
014	NP_149413.1	TRPCKRCLKTVSKGEYAYCKICKVGVCNDCALWCYQIKAPTCSQE-----
015	YP_004442824.1	MARCNACHHEALKGSRMDCLKCDLTICILCRPHYYPKIEIP-VEK-----
016	YP_001931967.1	--
017	YP_002455786.1	LHNIQQALHH-NCRGRLDSTLPLLGLIFLSPSGTTSVLFQTNHK------
018	YP_009109694.1	FEALKKALLSAPALGLPDTSKPFTLFLDERQGIAKGVLTQKLGPWK----
019	NP_789739.1	LAENREILKEPVHGVYYDPSKDLIAEIQKQGQGQWTYQIYQEPFKN----
020	NP_955619.1	LVQLRQALSQ-NCRSRLVQTLPLLGAIMLTLTGTTTVVFQSKQ-------
021	YP_224289.1	VERCRACTYYITSQPRIKCTKCKIPICNLCTNYCYGIKMADEK------E
022	YP_009182100.1	WEIPLMRIEEDDDYSDTSSIYSLETESDSESSDSDQESEDEDTVRMIKTQ
023	NP_705927.1	LAENREILKEPVHGVYYDPSKDLIAEIQKQGQGQWTYQIYQEPFKN----
024	YP_009508577.1	LEEIVSALNHAGNLEQRDNESPLVVKLNASP--KTGYIRYYNKGGQ----
025	YP_004442830.1	ARVCQRCFCKASKGQYIICGKCKVKVCNLCSDYCYGISLVKKEED-----
026	NP_758887.1	YEENKLIVQEEVHGVYYQPEKPLMAKVQKLTQGQWSYQIEQEDNKP----
027	YP_009508406.1	--
028	YP_610965.1	SW-CTYCKAPTSVLYRTKCTLCLLLCCPYCALHFH--QHRVRKER-----
029	YP_001931961.1	--
030	YP_241114.1	LQLIEEAIRR-QQITYCDYQRSWGLYILPTPRAPTGVLYQDK--------
031	YP_006732334.1	--
032	YP_009041481.1	NKRCGFCRNETRVTSRILCPACKLVACLLCASRRLGIEVRKEEE------
033	YP_001036293.1	TGLCKFCKRDITQRWHAKCMHCNLVTCGFCSQHYLDLEIPCQREE-----
034	YP_605811.1	SDYCRACKNRALQGSRADCTQCKMIICSLCKPYYFQDGSPIPAQG-----
035	NP_955577.1	FEDIKKALLSSPALGLPDITKPFELFIDENSGFAKGVLVQKLGPWK----
036	YP_003208050.1	AK-CAFCGVQTNPEMRMYCDICRLTACPMCSKYYL--DIPVRMNK-----
037	NP_789740.1	LAENREILKEPVHGVYYDPSKDLIAEIQKQGQGQWTYQIYQEPFKN----
038	YP_004442839.1	AGYCRACKHQALKDSRADCSTCKITICSLCKPHYYKDAAPIPVQK-----
039	YP_009507791.1	VRIAKEAIENLTDMSYFDNKKPVYCKFSIVGVHQVAYIVYQDQGQ-----
040	YP_004581513.1	--
041	NP_043933.1	--
042	YP_009507248.1	FEHCKTLLTNDPILQYPDFTREFNLTTDASNFAIGAVLSQGPIGSD----
043	YP_002117531.1	ELKCRCCFLPTRSKDRIHCKKCKITVCNMCSEFCYNIKIVRELPS----S
044	YP_004347415.1	--
045	NP_056891.1	LEKVETAIAE-QFVTHINYSLPLIFLIFNTALTPTGLFWQDN--------
046	YP_009513242.1	LQYIISVLNQADNLEERNPETRLILKVNSSP--SAGYIRYYNEGSK----
047	YP_009352866.1	PKNCGFCRLETRITSRISCPQCQLKACLLCAKRRLGIEVKKEEE------
048	YP_009165750.1	--
049	NP_040840.1	WEACRQKLKEMEGN-YYDEEKDIYGQLDWG-NKAIEYIVFQEKGKP----
050	YP_004222728.1	WREIVQLSTT-AALERWDPALPLEGAVARCEQGAIGVLGQGLSTHP----
051	YP_009506264.1	QADCRACGLKARSGGRMDCGKCGIIICCLCSQYCYDVTIPRE-------Q
052	YP_567050.1	LHAIQQALQH-NCRSRLNPALPILGLISLSSSGTTSVLFQARQR------
053	YP_009508571.1	LQTIIKALNNADNLEERIPEKRLIIKVNTSP--SAGYVRYYNETGK----
054	NP_056803.1	LNKVIEALNTASNLEERLPDQRLVIKVNTSP--SAGYVRYYNESGK----
055	NP_955564.1	LQLMNERLST-ARVKRLDLSQPWSLCILKTEYTPTACLWQDG--------
056	YP_009508411.1	SRTCRFCKREVMKRFNAHCSKCSITTCGMCSNHYLGFQIPCQQEE-----
057	YP_009140788.1	AIKCSFCRRESLKRARIHCPLCLLTACNLCGPYYLKIEVPVD-PA-----
058	YP_009508410.1	--
059	NP_040333.1	YQEIKQALLTAPALGLPDLTKPFELFVDEKQGYAKGVLTQKLGPWR----
060	YP_008992013.1	QFVQLPEVSSALAILHDQFLGDPAAAFEAARRDYLSMKCCSLDAKDLDFH
061	NP_041734.1	HINCHFCKQPTQLRSRIHCPTCQLTSCFMCAPIYCNMIVQQQ-PK-----
062	NP_056895.1	IAELRQALSH-NARSRYNEQEPLLAYVHLTRAGSTLVLFQKGAQ------
063	YP_009109689.1	FEALKKALLSAPALGLPDTSKPFTLFLDERQGIAKGVLTQKLGPWK----
064	YP_002519387.1	--
065	YP_595725.1	VKPCKRCLKTVTKGQYIYCRTCKITVCHECSEFCYNIKIEGAEAV-----
066	YP_009508551.1	LNMVIEALNTASNLEERLPEQRLVIKVNTSP--SAGYVRYYNETGK----
067	YP_001497148.1	FEALKEALLSAPALGLPDTSKPFTLFIDEKQGIAKGVLTQKLGPWK----
068	NP_041186.1	LQSIEEAIRQ-QQITYCDYQRSWGLYILPTPRAPTGVLYQDK--------
069	NP_663784.1	LEENKIILSQEQEGYYYQEEKELEATIQKSQGHQWTYKIHQEE-KI----
070	YP_009508443.1	ADKCRFCKREVMKRWNARCQHCNILTCGMCSKHYVDLELPCQQEE-----
071	YP_003987465.1	LKNCRACGATAQTGERMDCPLCNMTICSSCSNYCFDITIPKNEERVQAIV
072	NP_861410.1	--
073	YP_009345071.1	PEHCRCCNKETSDRCRIQCGYCKITACSLCAFSYFDIKVTQRKEF-----
074	YP_009508582.1	LEEIVSALNHAGNLEQRDDESPLVVKLNASP--KTGYIRYYNKGGQ----
075	NP_569141.1	--
076	YP_002916057.1	--
077	NP_777384.2	IAELRQALSH-NARSRYNEQEPLLAYVHLTRAGSTLVLFQKGAQ------
078	YP_233110.1	TDHCRACKNLALRGSRADCTQCKIIICSLCKPYYFQDGSPIPAQS-----
079	NP_542258.1	LKIIQKALAL-NCRSRLVSQLPILALIILRPTGTTAVLFQTKQK------

080 YP_009130664.1 SFKCFCCKEETSVNYRMHCPQCLITTCNLCALYYFGQKVPASRPN-----
081 YP_009508566.1 LNNLIIALNHADNLEQRNGEVPLVIKINASN--TTGYIRFYNKNGK----
082 YP_006607892.1 --
083 YP_009506270.1 VTTCKACGSEAVPKHRVDCKKCGMTVCLMCQPWYYKDINTEEVKK-----
084 NP_042513.1 --
085 YP_006907834.1 --
086 YP_004442827.1 VRICQRCFQKSPKGQYIICGICKVKVCHQCTEYCYGITMKGKEVQ-----
087 YP_009408594.1 IR-CLFCKYNTKKRMRIHCTQCKVTACPMCSDYYL--QVHITPG------
088 YP_009508561.1 LNELISALNHAGNLEQRRGDSPLVVKVNASD--KTGYIRYYNDNSL----
089 NP_056907.1 LHAIQQALQH-NCRSRLNPALPILGLISLSSSGTTSVLFQARQR------
090 YP_002308474.1 LNTLQKALTH-NCRSRIVSNLPILALIMLRPTGTTAVLFQTKQK------
091 YP_007761644.1 --
092 NP_040550.1 WREIVQLSTT-AALERWDPALPLEGAVARCEQGAIGVLGQGLSTHP----
093 NP_758808.1 HK-CHTCRRDTQKHYRLECQKCKFLVCSLCTIPYLGITMQFRQKQ-----
094 YP_009345075.1 NFNCLYCKNLTKQTRRWHCPKCQFTTCAYCAYLQLGKKMPPAPTK-----
095 NP_068729.1 --
096 NP_047255.1 FEDIKKALLSSPALGLPDITKPFELFIDENSGFAKGVLVQKLGPWK----
097 YP_006273075.1 AVHCSFCRRESIKRARIHCPLCLLTACNLCGPYYLKIEVPVE-PA-----
098 YP_009508888.1 LQELITLLNQADNLEERKPTTRLIIKVNSSS--HAGYIRYYNEGSK----
099 NP_395469.1 --
100 YP_009508546.1 LNTVIEALNTASNLEERLPNQRLVIKVNTSP--SAGYVRYYNESGK----
101 NP_056762.1 DKVCEICSYFTDYNKTVSCKTCETQYCKTCSDQLALEVTEVKKPTKEETM
102 YP_009508556.1 LQYIISVLNQADNLEERNPETRLILKVNSSP--SAGYIRYYNEGSR----
103 NP_569153.1 HVRCYPCKRETTQRARLHCKLCHITSCLMCGPTYFNKKITVQ-PM-----
104 NP_043924.1 RKMTPVESNLGICEQYAECAAWAISACNLVSGFGRKIIVTSHSPVKFILQ
105 YP_009243641.1 LQFFEHQLKS-KFLMRIDPLLPLTLYLINTFMYPTALLGQNE--------
106 YP_004300274.1 --
107 NP_056848.1 --
108 YP_006495799.1 -RFCSLCGDNTPVGRRVHCTTCKANLCPVCAWLEFGIRIVAAKT------
109 YP_009508409.1 --
110 NP_687035.1 YKNNQGIVQETQEGTYYDPLKELIATVQKQGEGQWTYQFTQEG-AV----
111 NP_045937.2 ---LKHAITTAPVLVVPDPAKPFQLYTSHSEHASIAVLTQKHAGRT----
112 NP_612577.1 --
113 NP_619548.1 --
114 NP_056880.1 LQLMNERLST-ARVKRLDLSQPWSLCILKTEYTPTACLWQDG--------
115 YP_009121747.1 SI-CHFCGIATTEISRLHCPECRLTACALCALHYLKIKIEVKRSK-----
116 YP_009113237.2 AK-CSFCKGERLQRQYVYCHKCDLLACGMCSWTYVNQKIEVK-PA-----
117 YP_009508408.1 VANCRFCKREVMQRWNAKCSKCSILTCGMCSEHYLGLNIPCQREE-----
118 NP_955579.1 FEDIKKALLSSPALGLPDITKPFELFIDENSGFAKGVLVQKLGPWK----
119 YP_009229919.1 EK-CSYCQYPTFKRMRCHCKGCGITACPMCSKFYL--EYLVTPEP-----
120 YP_004442833.1 VRSCQRCFGKFTKGQYVICGKCKVRVCHLCIQYCYGFSITKEEIP-----
121 NP_862833.2 LQFVEEAIRQ-QQITYCDYQRSWGLYILSTPRAPTGVLYQDK--------
122 NP_040563.1 VEEFNLKLKDPEWKDRIREGAELVIKIQMVPRGIVFDLLQDGNPIW----
123 YP_443922.1 FGLLKTDLSVAAALAAPDYSKPFHLDVSEKEGFASAILFQKQEGER----
124 NP_057933.2 YQEIKQALLTAPALGLPDLTKPFELFVDEKQGYAKGVLTQKLGPWR----
125 YP_009116631.1 -RVCSICSDETPHGRRVTCTTCNINLCPICARMDYGIMLIAAKDTK----
126 YP_009513249.1 LETLITKLNGAEYLQGRKGDKTLIMKVNASY--TTGYIRYYNEGEK----
127 YP_223871.1 FQSLKLALTQPPALALPSLDKPFQLFVEETSGAAKGVLTQALGPWK----
128 NP_127504.1 RFLTSQDFTWNIRILYSYFCGDQSQNKEELRRQIFEMKCLSYDRKKIDRH
129 NP_044929.1 LKTIIQALNSTENLEERRPDVDLIMKVHISN--TAGYIRFYNHGGQ----
130 NP_057860.1 LVQLRQALSQ-NCRSRLVQTLPLLGAIMLTLTGTTTVVFQSKQQ------
131 NP_040973.1 VQKAKKAIEEQVQLGYYDPSKELYAKLSLVGPHQISYQVYQKDPEKI---
132 NP_777317.1 PTTCKGCNIYAAPKNRMDCPQCKLTICVLCEPHYYKGAMALSKPE-----
133 NP_054716.1 LQKIIKELNASENLEQRKPDVELIVKVHVSP--TAGYIKFANKGSI----
134 YP_001856242.1 LAENREILKEPVHGVYYDPSKDLIAEIQKQGQGQWTYQIYQEPFKN----
135 NP_954565.2 LEKVETAIAE-QFVTHINYSLPLIFLIFNTALTPTGLFWQDN--------
136 NP_569150.1 VTICKACGSEAAPKHRIDCLKCEMTVCLMCQPWFYKNVNTEEVKK-----
137 NP_659397.1 --
138 YP_009513211.1 FGRIKEALLSAPALALPDLTKPFALYVDEKEGVARGVLTQTLGPWR----
139 YP_233107.1 SKHCRDCKFEARRDNRMDCSKCQLTICALCTYHCFK----ILIPR-----
140 YP_009109692.1 FEALKKALLSAPALGLPDTSKPFTLFLDERQGIAKGVLTQKLGPWK----
141 NP_057849.4 LAENREILKEPVHGVYYDPSKDLIAEIQKQGQGQWTYQIYQEPFKN----
142 NP_049560.1 LVQLRQALSQ-NCRSRLVQTVPLLGLVMLTLTGTTTVVFQSKQQ------
143 YP_009506267.1 NAHCRFCRREVMQRWNAKCNKCDITTCGMCSDHYVGLRVPCKRPE-----
144 NP_039820.1 QLACRSCKQFLAG---VQCHHCHAVYCFMCAEAYHDVQAEKILSKDYSFS
145 NP_056790.1 FDHIKKALLSAPALALPDLTKPFTLYIDERAGVARGVLTQTLGPWR----
146 YP_002321513.1 RIKCQECSIFTTPQARFLCDSCNLLLCAMCAQGYYGINIVEPVVPE----
147 NP_955591.1 YQEIKQALLTAPALGLPDLTKPFELFVDEKQGYAKGVLTQKLGPWR----
148 YP_001956722.2 LQNIISMLNSAENLEERNPEVRLIMKVNTSP--SAGYIRFYNEFAK----
149 NP_056902.1 LEENNEKIKNAQGLQYYNPEEEMLCEVEITKNYEATYVIKQSQGI-----
150 NP_056886.1 WREIVRLSTT-AALERWDPALPLEGAVARCEQGAIGVLGQGLSTHP----
151 NP_041261.1 LALINKAIQN-QSVQQISYNLPLVLLLPTPHTPTAVFWQPNGTDPTK---

```
001 YP_004442836.1   TRVRIERVIDWKEIALRQ--HEVLKTSIANEK-----------QLSEENE
002 NP_056728.1      --------------------------------------------------
003 YP_009508537.1   --------------------------------------------------
004 YP_004732983.2   -TRADWKFQDRDSLIASL--YEHNAFLLRQVEGLKQELQAAKEQLQLLHS
005 YP_009508407.1   --------------------------------------------------
006 NP_040939.1      --------------------------------------------------
007 YP_003864102.1   --------------------------------------------------
008 YP_009002585.1   --QTSFFNEKS--LLTQQ--QEYIAYQTAEIK-----------KLQEQLK
009 YP_003284237.1   SFSSQHHEPKWKEIATAL--GEINAKLEQEKK-----------ALIEELN
010 YP_009268869.1   --------------------------------------------------
011 YP_009506251.1   --TPIPFSPNA--LLQQQ--AAYIRWLESENK-----------RLNELVE
012 NP_955611.1      --------------------------------------------------
013 YP_008567619.1   --V-APYNPRN--LLEEQ--QRYIEWCELEMK-----------RLKESII
014 NP_149413.1      --KKPSSSINYEKLARQL--LLENSKLKEEKK-----------ILIEELN
015 YP_004442824.1   IKTRETRSVDWKKIVLEQ--HEALLSISAREK-----------SLRKELE
016 YP_001931967.1   --------------------------------------------------
017 YP_002455786.1   --------------------------------------------------
018 YP_009109694.1   --------------------------------------------------
019 NP_789739.1      --------------------------------------------------
020 NP_955619.1      --------------------------------------------------
021 YP_224289.1      KEKEKEKESDLLGLVNNC--REIMAKYRTEKE-----------VLLEELN
022 YP_009182100.1   FGIKTIRLNNWPHDTQEIGDVVSFEAILEYEKFECDFCKTSADQYTPVIQ
023 NP_705927.1      --------------------------------------------------
024 YP_009508577.1   --------------------------------------------------
025 YP_004442830.1   --PEVD----YKSLAASL--ISENERLKLEKK-----------VLLEELN
026 NP_758887.1      --------------------------------------------------
027 YP_009508406.1   --------------------------------------------------
028 YP_610965.1      --PPVEAP---EQLIQNL--IGYIDSLLKENE-----------RLKEER-
029 YP_001931961.1   --------------------------------------------------
030 YP_241114.1      --------------------------------------------------
031 YP_006732334.1   --------------------------------------------------
032 YP_009041481.1   ---VTWKFQNKDELINSL--YHHNAFLIKENS-----LLKEQLQLAQRRI
033 YP_001036293.1   --V-QVYNPQR--LLQEQ--QNYIQVCEVEIE-----------KLKESVK
034 YP_605811.1      STPSGYSHDDWMGSANRW--KAHYEFSQARRK-----------SLKADLE
035 NP_955577.1      --------------------------------------------------
036 YP_003208050.1   --PPPPPPENKDELIHEL--VVYTETLLKRVK-----------ELEEQL-
037 NP_789740.1      --------------------------------------------------
038 YP_004442839.1   KVVK----DDWMNMAKRF--MDSWKKAEDEKM-----------ELKEELN
039 YP_009507791.1   --------------------------------------------------
040 YP_004581513.1   --------------------------------------------------
041 NP_043933.1      --------------------------------------------------
042 YP_009507248.1   --------------------------------------------------
043 YP_002117531.1   SLLKDRKKVDYIGVCIEV--LEENKKLRTEKE-----------VLIQDLN
044 YP_004347415.1   --------------------------------------------------
045 NP_056891.1      --------------------------------------------------
046 YP_009513242.1   --------------------------------------------------
047 YP_009352866.1   ---VTWKFQNKDELINNL--YLHNAFLIKENS-----LLKEQLQLAQRRV
048 YP_009165750.1   --------------------------------------------------
049 NP_040840.1      --------------------------------------------------
050 YP_004222728.1   --------------------------------------------------
051 YP_009506264.1   NQ-VAYASPNWREIALRQ--QEIIRDLEAEKR-----------ALIEEVN
052 YP_567050.1      --------------------------------------------------
053 YP_009508571.1   --------------------------------------------------
054 NP_056803.1      --------------------------------------------------
055 NP_955564.1      --------------------------------------------------
056 YP_009508411.1   --VRPVYEPRQ--LMNEQ--MTYIKHCEEEIA-----------RLKQEVA
057 YP_009140788.1   --PSTPLNPRR--IIQEQ--HNYIGWCEAEIE-----------RLNKEVK
058 YP_009508410.1   --------------------------------------------------
059 NP_040333.1      --------------------------------------------------
060 YP_008992013.1   YKRMSLLFYKLNGFNEPTLKHVFLASLPEELQPDIQRQLTASNLVIDNIS
061 NP_041734.1      --PPVPFNTHT--LLQQQ--AAYIQWLEKENQ-----------RLTEAVE
062 NP_056895.1      --------------------------------------------------
063 YP_009109689.1   --------------------------------------------------
064 YP_002519387.1   --------------------------------------------------
065 YP_595725.1      --KPPEKKSNYELLAKQL--LIENSKLKMEKE-----------ILIEELN
066 YP_009508551.1   --------------------------------------------------
067 YP_001497148.1   --------------------------------------------------
068 NP_041186.1      --------------------------------------------------
069 NP_663784.1      --------------------------------------------------
070 YP_009508443.1   --IRQVYNPQQ--LMMEQ--QNYIQSCEMEIQ-----------RLKDELA
```

```
071 YP_003987465.1   SSSTASREESWKTIALEQ--RKLIKKLENEKE-----------ALIVELN
072 NP_861410.1      --------------------------------------------------
073 YP_009345071.1   --VSPYYATPS--LLKEN--QEYISWCQAELN-----------KTGEAMT
074 YP_009508582.1   --------------------------------------------------
075 NP_569141.1      --------------------------------------------------
076 YP_002916057.1   --------------------------------------------------
077 NP_777384.2      --------------------------------------------------
078 YP_233110.1      STPSGYSYDDWMGSANRW--KAHYEFSQARRK-----------SLKADLE
079 NP_542258.1      --------------------------------------------------
080 YP_009130664.1   --P-IQYNPLS--LMHEQ--QGYITWCEAEIT-----------RLKQEVE
081 YP_009508566.1   --------------------------------------------------
082 YP_006607892.1   --------------------------------------------------
083 YP_009506270.1   TRVRIERVIDWKDIALKQ--HEVLKTSIAREK-----------QLSEEVE
084 NP_042513.1      --------------------------------------------------
085 YP_006907834.1   --------------------------------------------------
086 YP_004442827.1   --PEED----YKELAKSL--LAENKKLKLERK-----------LLLEELN
087 YP_009408594.1   --PPVQKEFDEKPVLREL--LQYVTWLQTEND-----------RLKAELR
088 YP_009508561.1   --------------------------------------------------
089 NP_056907.1      --------------------------------------------------
090 YP_002308474.1   --------------------------------------------------
091 YP_007761644.1   --------------------------------------------------
092 NP_040550.1      --------------------------------------------------
093 NP_758808.1      --KSQPENPNLVRELLEH--AIFLEEKCKNQE-----------LLSETQI
094 YP_009345075.1   --EPAVRSKVQSSAVHAE--LSRLRNERDHLE-----------RIVDSFL
095 NP_068729.1      --------------------------------------------------
096 NP_047255.1      --------------------------------------------------
097 YP_006273075.1   --PSTPINPRR--IIQEQ--HNYIGWCEVEIE-----------RLNKEVK
098 YP_009508888.1   --------------------------------------------------
099 NP_395469.1      --------------------------------------------------
100 YP_009508546.1   --------------------------------------------------
101 NP_056762.1      IDDLKLNVKNLEFRVTILEHKVEMQNLQDKFETMQIR-------------
102 YP_009508556.1   --------------------------------------------------
103 NP_569153.1      --PQAPFNQKG--LLQQQ--QEYIAWCNNEIA-----------RLKEEVA
104 NP_043924.1      TTPNVSNQRLARWHRILTQEDITIETDASIQG------------------
105 YP_009243641.1   --------------------------------------------------
106 YP_004300274.1   --------------------------------------------------
107 NP_056848.1      --------------------------------------------------
108 YP_006495799.1   -DATKWNYLNKDELIAQL--YEHNAFLTHQNK----ALQGRIEELTKGRN
109 YP_009508409.1   --------------------------------------------------
110 NP_687035.1      --------------------------------------------------
111 NP_045937.2      --------------------------------------------------
112 NP_612577.1      --------------------------------------------------
113 NP_619548.1      --------------------------------------------------
114 NP_056880.1      --------------------------------------------------
115 YP_009121747.1   --GSSKGS-----EVIVP--DEETQQPNSIWK-----------LLAQKDN
116 YP_009113237.2   --AKPVPKPLSTDLVRLQ--SEHLLWQEKQID-----------KLKEEVL
117 YP_009508408.1   --IRQVYNPRQ--LMMEQ--QSYIQSCELEIQ-----------RLKEEIQ
118 NP_955579.1      --------------------------------------------------
119 YP_009229919.1   --PAPKQYSSKDRLIQEL--MSYNVYLLSENE-----------RLQEIIN
120 YP_004442833.1   --KEEEN---WKELASTL--MIENRKLKLEKK-----------LLLKELN
121 NP_862833.2      --------------------------------------------------
122 NP_040563.1      --------------------------------------------------
123 YP_443922.1      --------------------------------------------------
124 NP_057933.2      --------------------------------------------------
125 YP_009116631.1   -SAAHWQYQNKDELIQHL--YEHNAFLTRKVA----ELTSQLQEFHNRRP
126 YP_009513249.1   --------------------------------------------------
127 YP_223871.1      --------------------------------------------------
128 NP_127504.1      FQRMIKLFYHIG--GDISLKQAFISSLPPILSERISALIKERGTSVTQMH
129 NP_044929.1      --------------------------------------------------
130 NP_057860.1      --------------------------------------------------
131 NP_040973.1      --------------------------------------------------
132 NP_777317.1      PAPEEPKHLYWMRYAKEQ--LDKYKEAQKEKL-----------ELRKRVE
133 NP_054716.1      --------------------------------------------------
134 YP_001856242.1   --------------------------------------------------
135 NP_954565.2      --------------------------------------------------
136 NP_569150.1      TRVRIERVIDWKDIALKQ--HEVLKTSIANEK-----------QLSEEVE
137 NP_659397.1      --------------------------------------------------
138 YP_009513211.1   --------------------------------------------------
139 YP_233107.1      KQTTPSMKHDWRELAERQ--SELLRKFCQREK-----------ELEQEVQ
140 YP_009109692.1   --------------------------------------------------
141 NP_057849.4      --------------------------------------------------
142 NP_049560.1      --------------------------------------------------
143 YP_009506267.1   --VQQVYQPTN--LLVEQ--RNYIMSCEEEIA-----------RLKNEIE
```

144 NP_039820.1 ARGKKGKAVIIEEDEIEG--EFLISQLQQENQ-----------RLQKQVE
145 NP_056790.1 --
146 YP_002321513.1 -LQHHIERPKIDKGKKKVSWQEEIQDLEGMSEIPYARARQSLRMLSSDEE
147 NP_955591.1 --
148 YP_001956722.2 --
149 NP_056902.1 --
150 NP_056886.1 --
151 NP_041261.1 --

001 YP_004442836.1 ELKEKI-------------KELKGKELITVEEISEEEG------------
002 NP_056728.1 --
003 YP_009508537.1 --
004 YP_004732983.2 VDMINLSD-----------DGLENFSLEEKSFLRGGGTSSSS--------
005 YP_009508407.1 --
006 NP_040939.1 --
007 YP_003864102.1 --
008 YP_009002585.1 QTE----------EKLAEAERKIRGYENMEVLEKERKEMAL---------
009 YP_003284237.1 RALEQI-------------KDYKQKGLPTAIIQEVDE-------------
010 YP_009268869.1 --
011 YP_009506251.1 FYK----------RLLDGGSRLQQ------ELEADQKELEE---------
012 NP_955611.1 --
013 YP_008567619.1 QNDELRMENDRLQLENRDLKRQIEKLQDKQKMKALMEDLGEEF-------
014 NP_149413.1 KEIKAR-------------QEAEKNKAVFVEETSSEVN------------
015 YP_004442824.1 QALEQL-------------KHYKRESEIIIEEE----P------------
016 YP_001931967.1 --
017 YP_002455786.1 --
018 YP_009109694.1 --
019 NP_789739.1 --
020 NP_955619.1 --
021 YP_224289.1 NALKQA-------------KEYKEK--FEHLEGICEEQ------------
022 YP_009182100.1 RSDLSKEGTISYHTWCFTSLVMRETKNAEVPQLVQREVDLAKLRDAKKKF
023 NP_705927.1 --
024 YP_009508577.1 --
025 YP_004442830.1 KEILEK-------------NQLRN-ELPVIEETNSEAA------------
026 NP_758887.1 --
027 YP_009508406.1 --
028 YP_610965.1 DDLQQK-------LQRTEERLQSLLGDYLHEASEDMAA------------
029 YP_001931961.1 --
030 YP_241114.1 --
031 YP_006732334.1 --
032 YP_009041481.1 ANNAQLTG----ADLQAFMEDYRNLTLETAQKIATETPKPVGK-------
033 YP_001036293.1 AANKIA--------EDKIMAASIRHAVELEELRKQLQAIK----------
034 YP_605811.1 KAEEEL-------------KFYKQKEKERARLKNQIP-------------
035 NP_955577.1 --
036 YP_003208050.1 KEKELE-------IARLELMIN-------DEGKQETAE------------
037 NP_789740.1 --
038 YP_004442839.1 DIKEQI-------------KFYKGKEKE--VCTEEIP-------------
039 YP_009507791.1 --
040 YP_004581513.1 --
041 NP_043933.1 --
042 YP_009507248.1 --
043 YP_002117531.1 SALEQL-------------KNTKGK--EIIVEEESPE-------------
044 YP_004347415.1 --
045 NP_056891.1 --
046 YP_009513242.1 --
047 YP_009352866.1 ANNAMLTG----ADLQAFMEDYRNLTVETSQRIAEGTRGPLGQ-------
048 YP_009165750.1 --
049 NP_040840.1 --
050 YP_004222728.1 --
051 YP_009506264.1 MSRDEI-------------KQYKAKGLTEIIEETPEET------------
052 YP_567050.1 --
053 YP_009508571.1 --
054 NP_056803.1 --
055 NP_955564.1 --
056 YP_009508411.1 AAKILS--------ENEIVSMKIREAVETDTLRKENETLR----------
057 YP_009140788.1 HYK----------GLYEALLTEKS-------LKKDYEDLRE---------
058 YP_009508410.1 --
059 NP_040333.1 --
060 YP_008992013.1 LGKIFQLAKTCLDKLCEQKQFFKDLLKDKEPFRSACKKPYLQIKCKQKKD
061 NP_041734.1 FYK----------KEAEE-LRLER------DLEQDRRSLEP---------
062 NP_056895.1 --

```
063 YP_009109689.1    --------------------------------------------------
064 YP_002519387.1    --------------------------------------------------
065 YP_595725.1       KEIKAH-------------QETKKGKELYIEEASTEVE------------
066 YP_009508551.1    --------------------------------------------------
067 YP_001497148.1    --------------------------------------------------
068 NP_041186.1       --------------------------------------------------
069 NP_663784.1       --------------------------------------------------
070 YP_009508443.1    REKKVA--------EEMVVAARISEAVSQQESKKQLEEMK----------
071 YP_003987465.1    QALEQL-------------KQTKGKGIPEHQDDALEKELLKELLREEKEK
072 NP_861410.1       --------------------------------------------------
073 YP_009345071.1    HIKKEN------AAQSEVITAQEHVINKLKEEKLDRQKIVEELKQELQ--
074 YP_009508582.1    --------------------------------------------------
075 NP_569141.1       --------------------------------------------------
076 YP_002916057.1    --------------------------------------------------
077 NP_777384.2       --------------------------------------------------
078 YP_233110.1       RAEEEL-------------KFYKQKEKEKAKLKDQIP-------------
079 NP_542258.1       --------------------------------------------------
080 YP_009130664.1    TS-----------------KKQVEYW--KQKYLSTQEEV-----------
081 YP_009508566.1    --------------------------------------------------
082 YP_006607892.1    --------------------------------------------------
083 YP_009506270.1    FLRKQN-------------KELKSKEPIVFEEDTEET-------------
084 NP_042513.1       --------------------------------------------------
085 YP_006907834.1    --------------------------------------------------
086 YP_004442827.1    NQILEN-------------TKIKR-ELPVIEETSSEAA------------
087 YP_009408594.1    AATERR-------PREDVGDVEID-----AELLKDLED------------
088 YP_009508561.1    --------------------------------------------------
089 NP_056907.1       --------------------------------------------------
090 YP_002308474.1    --------------------------------------------------
091 YP_007761644.1    --------------------------------------------------
092 NP_040550.1       --------------------------------------------------
093 NP_758808.1       ERIVSS-------EKQVKFYGILPTKKSNKSAGYDLQSNIDIEIPPG---
094 YP_009345075.1    DQLPSE-------LQRSVVEPSQNQKLQQQEEPSSQKG------------
095 NP_068729.1       --------------------------------------------------
096 NP_047255.1       --------------------------------------------------
097 YP_006273075.1    HYK----------GLYEALLMEKS-------LRKDYEELKE---------
098 YP_009508888.1    --------------------------------------------------
099 NP_395469.1       --------------------------------------------------
100 YP_009508546.1    --------------------------------------------------
101 NP_056762.1       --------------------------------------------------
102 YP_009508556.1    --------------------------------------------------
103 NP_569153.1       FYK----------QLAQERELQLQ-------LEQSRKELAG---------
104 NP_043924.1       --------------------------------------------------
105 YP_009243641.1    --------------------------------------------------
106 YP_004300274.1    --------------------------------------------------
107 NP_056848.1       --------------------------------------------------
108 YP_006495799.1    NDLIDLCEGDPDLMAEICDELFRKPASGETSILRGKERVKLEI-------
109 YP_009508409.1    --------------------------------------------------
110 NP_687035.1       --------------------------------------------------
111 NP_045937.2       --------------------------------------------------
112 NP_612577.1       --------------------------------------------------
113 NP_619548.1       --------------------------------------------------
114 NP_056880.1       --------------------------------------------------
115 YP_009121747.1    DWKQAI-------RDQAKEEALWQIKEKELRAQINEKD------------
116 YP_009113237.2    YWKNKYLAS-IERDLEQEFQTLPVREEDREKKRKEDDEMEEDRVSSNQKP
117 YP_009508408.1    NVKKVA--------EEQVVAAKINEAVAVKEMQEQIQELK----------
118 NP_955579.1       --------------------------------------------------
119 YP_009229919.1    KKLIED-------LEKGRMEAER-----FDQAKERRKG------------
120 YP_004442833.1    DQIEAN-------------KKIKE-EFPVIEETNSDAA------------
121 NP_862833.2       --------------------------------------------------
122 NP_040563.1       --------------------------------------------------
123 YP_443922.1       --------------------------------------------------
124 NP_057933.2       --------------------------------------------------
125 YP_009116631.1    EDLISLAD---DLEDVSILDNASNRGKEEKELFQFGTTIPIDH-------
126 YP_009513249.1    --------------------------------------------------
127 YP_223871.1       --------------------------------------------------
128 NP_127504.1       VGDIRQTAFYVLDDLCSKRKFFNQMKKMSRDLEKACTKSDLIIKGDKGCS
129 NP_044929.1       --------------------------------------------------
130 NP_057860.1       --------------------------------------------------
131 NP_040973.1       --------------------------------------------------
132 NP_777317.1       ILETMI-------------PQHKELEEE----------------------
133 NP_054716.1       --------------------------------------------------
134 YP_001856242.1    --------------------------------------------------
135 NP_954565.2       --------------------------------------------------
```

```
136 NP_569150.1       ILRKQS-------------KELKEKEPIIFEEDTEET-------------
137 NP_659397.1       --------------------------------------------------
138 YP_009513211.1    --------------------------------------------------
139 YP_233107.1       ELRREI-------------STLKTDLANLSVIQETPE-------------
140 YP_009109692.1    --------------------------------------------------
141 NP_057849.4       --------------------------------------------------
142 NP_049560.1       --------------------------------------------------
143 YP_009506267.1    AMKISS--------ENQLVAEKIARAVEEEHLKKEVQALK----------
144 NP_039820.1       RLQEEL-------------MKLHREKDEALKHSEKASR------------
145 NP_056790.1       --------------------------------------------------
146 YP_002321513.1    QPNRPQRQAEKESDTIIKMASIMAEQTKEISYWRDKAVELKQALGDLQRD
147 NP_955591.1       --------------------------------------------------
148 YP_001956722.2    --------------------------------------------------
149 NP_056902.1       --------------------------------------------------
150 NP_056886.1       --------------------------------------------------
151 NP_041261.1       --------------------------------------------------
```

```
001 YP_004442836.1    ---------AAMIEKIENLEKENELLNAIIT-------------------
002 NP_056728.1       --------------------------------------------------
003 YP_009508537.1    --------------------------------------------------
004 YP_004732983.2    ---------IKISSTTTP--PGFPTTPNRFQP------------------
005 YP_009508407.1    --------------------------------------------------
006 NP_040939.1       --------------------------------------------------
007 YP_003864102.1    --------------------------------------------------
008 YP_009002585.1    ---------KDMQRKIDEEIKKAAMQELPTEK------------------
009 YP_003284237.1    -------------SKTEELERENELLNAVLE-------------------
010 YP_009268869.1    --------------------------------------------------
011 YP_009506251.1    ---------LEADRKELEAHDKGKRVQFTEEEK-----------------
012 NP_955611.1       --------------------------------------------------
013 YP_008567619.1    --------DYEEEVNVLVEDWDNKRVTKELD-------------------
014 NP_149413.1       ---------DEIEMWKSKAELYEALYHEALNK------------------
015 YP_004442824.1    ---------VELMEELENTKRENELLNALLER------------------
016 YP_001931967.1    --------------------------------------------------
017 YP_002455786.1    --------------------------------------------------
018 YP_009109694.1    --------------------------------------------------
019 NP_789739.1       --------------------------------------------------
020 NP_955619.1       --------------------------------------------------
021 YP_224289.1       ------------EAENKALAQENQDLKEKVE-------------------
022 YP_009182100.1    EQAQLKEEQERLRATRRPVILEVYDDAEELEEMSKIGGHLEELSIKLRRP
023 NP_705927.1       --------------------------------------------------
024 YP_009508577.1    --------------------------------------------------
025 YP_004442830.1    ---------LEIDMLQEVVTSLQRQLEQKDQI------------------
026 NP_758887.1       --------------------------------------------------
027 YP_009508406.1    --------------------------------------------------
028 YP_610965.1       ---------EAERRRKGKALATSVPRATTGGPVI----------------
029 YP_001931961.1    --------------------------------------------------
030 YP_241114.1       --------------------------------------------------
031 YP_006732334.1    --------------------------------------------------
032 YP_009041481.1    ---------AALTWREEAEAAYKEELATRKNW------------------
033 YP_001036293.1    ---------LDKERLELENPDLKKILE-----------------------
034 YP_605811.1       ---------EAIQAKLDDLEREKELNNILRIE------------------
035 NP_955577.1       --------------------------------------------------
036 YP_003208050.1    ---------EGEE-------------------------------------
037 NP_789740.1       --------------------------------------------------
038 YP_004442839.1    ---------QIYTQKLELLENE----NSLLKD------------------
039 YP_009507791.1    --------------------------------------------------
040 YP_004581513.1    --------------------------------------------------
041 NP_043933.1       ----------------------MENNLQLLEELN----HLEEG-------
042 YP_009507248.1    --------------------------------------------------
043 YP_002117531.1    -----------LLARISYLEQRELFMQELYE-------------------
044 YP_004347415.1    --------------------------------------------------
045 NP_056891.1       --------------------------------------------------
046 YP_009513242.1    --------------------------------------------------
047 YP_009352866.1    ---------AARNWRKEADVEWTEEEED----------------------
048 YP_009165750.1    --------------------------------------------------
049 NP_040840.1       --------------------------------------------------
050 YP_004222728.1    --------------------------------------------------
051 YP_009506264.1    ------------AKEIERLEAEKALLAELLE-------------------
052 YP_567050.1       --------------------------------------------------
053 YP_009508571.1    --------------------------------------------------
054 NP_056803.1       --------------------------------------------------
```

```
055 NP_955564.1        --------------------------------------------------
056 YP_009508411.1     ---------LENERLQLEVADLNKIIKGKQPIIPEDEELE----------
057 YP_009140788.1     ---------GDIKRGKGVMIQEPEDINYLGEN------------------
058 YP_009508410.1     --------------------------------------------------
059 NP_040333.1        --------------------------------------------------
060 YP_008992013.1     CDCSPKKKRHFRKFKSPEFSSRP-RRSRKPYRF-----------------
061 NP_041734.1        ---------TLLD--------KGKKVQILDPD------------------
062 NP_056895.1        --------------------------------------------------
063 YP_009109689.1     --------------------------------------------------
064 YP_002519387.1     --------------------------------------------------
065 YP_595725.1        ---------NEIETWKSRAELFEALYNEEVKKN-----------------
066 YP_009508551.1     --------------------------------------------------
067 YP_001497148.1     --------------------------------------------------
068 NP_041186.1        --------------------------------------------------
069 NP_663784.1        --------------------------------------------------
070 YP_009508443.1     ---------LENDRLTLENEDLKKEIRS----------------------
071 YP_003987465.1     AKKAEESLQELLKEEKEKAKKAEESLQELLR-------------------
072 NP_861410.1        --------------------------------------------------
073 YP_009345071.1     ---------QEKEKFLKEIEEQKELFMRQIAEEK----------------
074 YP_009508582.1     --------------------------------------------------
075 NP_569141.1        --------------------------------------------------
076 YP_002916057.1     --------------------------------------------------
077 NP_777384.2        --------------------------------------------------
078 YP_233110.1        ---------EAVQAKLDDLEKEKELNNILRIE------------------
079 NP_542258.1        --------------------------------------------------
080 YP_009130664.1     ---------FEKEFDELYKEKGKGVVIQEAT-------------------
081 YP_009508566.1     --------------------------------------------------
082 YP_006607892.1     --------------------------------------------------
083 YP_009506270.1     ---------AQLVQKLEDMEKENELMKILIS-------------------
084 NP_042513.1        --------------------------------------------------
085 YP_006907834.1     --------------------------------------------------
086 YP_004442827.1     ---------IEIDTLQEALERANELNKEKDAM------------------
087 YP_009408594.1     ---------LEIH-------------------------------------
088 YP_009508561.1     --------------------------------------------------
089 NP_056907.1        --------------------------------------------------
090 YP_002308474.1     --------------------------------------------------
091 YP_007761644.1     --------------------------------------------------
092 NP_040550.1        --------------------------------------------------
093 NP_758808.1        -------KCTVISTGTFLQMPDNMYGRLVERTSLAIQG------------
094 YP_009345075.1     ---------VCIQEPPPQKPAITFTRNEGSTVTLPTKKTRRRAGWDFYSP
095 NP_068729.1        --------------------------------------------------
096 NP_047255.1        --------------------------------------------------
097 YP_006273075.1     ---------GDKERRKGIMIQEPEEVNYLGED------------------
098 YP_009508888.1     --------------------------------------------------
099 NP_395469.1        --------------------------------------------------
100 YP_009508546.1     --------------------------------------------------
101 NP_056762.1        --------------------------------------------------
102 YP_009508556.1     --------------------------------------------------
103 NP_569153.1        ---------VDSRRRKDKGIVIDEGSCYFNPE------------------
104 NP_043924.1        --------------------------------------------------
105 YP_009243641.1     --------------------------------------------------
106 YP_004300274.1     --------------------------------------------------
107 NP_056848.1        --------------------------------------------------
108 YP_006495799.1     ---------LEEEVRGYKEEQELRTPLQKLSEGASTS-------------
109 YP_009508409.1     --------------------------------------------------
110 NP_687035.1        --------------------------------------------------
111 NP_045937.2        --------------------------------------------------
112 NP_612577.1        --------------------------------------------------
113 NP_619548.1        --------------------------------------------------
114 NP_056880.1        --------------------------------------------------
115 YP_009121747.1     -------KKAQEESQKWAEERQSLYKDIDDLLKIQQQ-------------
116 YP_009113237.2     YKS----STPAKERDRRTVIIQEPHQETVQKTV-----------------
117 YP_009508408.1     ---------LENDRLTLENHDLKELLK-----------------------
118 NP_955579.1        --------------------------------------------------
119 YP_009229919.1     ---------VVIRTPSGS--------------------------------
120 YP_004442833.1     ---------IEIEMLQELLNEANKSNNQ----------------------
121 NP_862833.2        --------------------------------------------------
122 NP_040563.1        --------------------------------------------------
123 YP_443922.1        --------------------------------------------------
124 NP_057933.2        --------------------------------------------------
125 YP_009116631.1     ---------IQNLENVAKIIEKWKDTPRVVIK------------------
126 YP_009513249.1     --------------------------------------------------
127 YP_223871.1        --------------------------------------------------
```

128 NP_127504.1 GYCNPSRRRKYKRFKLPSFKERDGRQYRKRRRF-----------------
129 NP_044929.1 --
130 NP_057860.1 --
131 NP_040973.1 --
132 NP_777317.1 ---------NELKIRLEDEKRENSLLQRLYE-------------------
133 NP_054716.1 --
134 YP_001856242.1 --
135 NP_954565.2 --
136 NP_569150.1 ---------AQLIQKLEDVERENELLNILIK-------------------
137 NP_659397.1 --
138 YP_009513211.1 --
139 YP_233107.1 ----------EDEEELQRLRQENTLLELLNQ-------------------
140 YP_009109692.1 --
141 NP_057849.4 --
142 NP_049560.1 --
143 YP_009506267.1 ---------LENDRLTLENEDQRKELEKK---------------------
144 NP_039820.1 ----------VFSTIQESDEAELNLIKEELR-------------------
145 NP_056790.1 --
146 YP_002321513.1 YAQ----LQKEVELLRWHNVVKNSQPKEADESLETNEDSIN---------
147 NP_955591.1 --
148 YP_001956722.2 --
149 NP_056902.1 --
150 NP_056886.1 --
151 NP_041261.1 --

001 YP_004442836.1 -----------------------------------QKEEEEVGYLNKIQE
002 NP_056728.1 --
003 YP_009508537.1 --
004 YP_004732983.2 ---LAQEKLKGI
005 YP_009508407.1 --
006 NP_040939.1 --
007 YP_003864102.1 --
008 YP_009002585.1 ---ELWEQ
009 YP_003284237.1 -----------------------------------KKDKE----------
010 YP_009268869.1 --
011 YP_009506251.1 --------------------------------------VQLTEELEEIHM
012 NP_955611.1 --
013 YP_008567619.1 ---DLE
014 NP_149413.1 ---------------------------------PSSSMIKEGLYQTQITH
015 YP_004442824.1 --------------------------------EKTEREELAIRASEEKEA
016 YP_001931967.1 --
017 YP_002455786.1 --
018 YP_009109694.1 --
019 NP_789739.1 --
020 NP_955619.1 --
021 YP_224289.1 -----------------------------------ELKEENEILKDQNTT
022 YP_009182100.1 VAASPGATSSNPLEENVDTPKDLKGQILLCPIFKGPIPKRTEADWKREEE
023 NP_705927.1 --
024 YP_009508577.1 --
025 YP_004442830.1 ---IIQ
026 NP_758887.1 --
027 YP_009508406.1 --
028 YP_610965.1 --REPLEG
029 YP_001931961.1 --MSTSQR
030 YP_241114.1 --
031 YP_006732334.1 --------------------------------------MLSQQMMNHQEQ
032 YP_009041481.1 --RQKKKIGE
033 YP_001036293.1 --
034 YP_605811.1 --------------------------------AEAELKALKESFKEREEA
035 NP_955577.1 --
036 YP_003208050.1 --AN
037 NP_789740.1 --
038 YP_004442839.1 --------------------------------LEEERKIQDIQVFKEIQA
039 YP_009507791.1 --
040 YP_004581513.1 --
041 NP_043933.1 ------------------------------------WEMLTDEDFKLQQN
042 YP_009507248.1 --
043 YP_002117531.1 -----------------------------------KEKFEKDLLQAQMRS
044 YP_004347415.1 --
045 NP_056891.1 --
046 YP_009513242.1 --

```
047 YP_009352866.1 -------------------------------------------------E
048 YP_009165750.1 --------------------------------------------------
049 NP_040840.1 --------------------------------------------------
050 YP_004222728.1 --------------------------------------------------
051 YP_009506264.1 -----------------------------------KEQAKNKMKIGELEA
052 YP_567050.1 --------------------------------------------------
053 YP_009508571.1 --------------------------------------------------
054 NP_056803.1 --------------------------------------------------
055 NP_955564.1 --------------------------------------------------
056 YP_009508411.1 --------------------------------------ELKQQVQQLKKS
057 YP_009140788.1 --------------------------------------------------
058 YP_009508410.1 --------------------------------------------------
059 NP_040333.1 --------------------------------------------------
060 YP_008992013.1 ---------------------------------------FRKRSSSSKDS
061 NP_041734.1 ---------------------------------------------EDQHT
062 NP_056895.1 --------------------------------------------------
063 YP_009109689.1 --------------------------------------------------
064 YP_002519387.1 --------------------------------------------MTKSKI
065 YP_595725.1 --------------------------------KASTSSVTEGMYQVQIDH
066 YP_009508551.1 --------------------------------------------------
067 YP_001497148.1 --------------------------------------------------
068 NP_041186.1 --------------------------------------------------
069 NP_663784.1 --------------------------------------------------
070 YP_009508443.1 ----------------------------------------------LKIQ
071 YP_003987465.1 -----------------------------------KEKEKVRKAEESQKE
072 NP_861410.1 --------------------------------------------------
073 YP_009345071.1 ------------------------------------------AKFLSLKQ
074 YP_009508582.1 --------------------------------------------------
075 NP_569141.1 --------------------------------------------------
076 YP_002916057.1 --------------------------------------------------
077 NP_777384.2 --------------------------------------------------
078 YP_233110.1 --------------------------------AETELKALKESFKEKEEA
079 NP_542258.1 --------------------------------------------------
080 YP_009130664.1 -----------------------------------------------NVF
081 YP_009508566.1 --------------------------------------------------
082 YP_006607892.1 --------------------------------------------MNMNTM
083 YP_009506270.1 -----------------------------------QKEAEEIQYLNKITE
084 NP_042513.1 --------------------------------------------------
085 YP_006907834.1 --------------------------------------------------
086 YP_004442827.1 -----------------------------------------------ILK
087 YP_009408594.1 -------------------------------------------------D
088 YP_009508561.1 --------------------------------------------------
089 NP_056907.1 --------------------------------------------------
090 YP_002308474.1 --------------------------------------------------
091 YP_007761644.1 --------------MNQTRFEDDPIEHDLNAQSWIKWFHLMRRQDTGYQW
092 NP_040550.1 --------------------------------------------------
093 NP_758808.1 ---------------------------------------ITVQGGVIDPD
094 YP_009345075.1 GDIALPIGAILTIQTQLKVQLPSGTYMQLAAKSSLAKLGVQVLGGVIDED
095 NP_068729.1 --------------------------------------------------
096 NP_047255.1 --------------------------------------------------
097 YP_006273075.1 --------------------------------------------------
098 YP_009508888.1 --------------------------------------------------
099 NP_395469.1 --------------------------------------------------
100 YP_009508546.1 --------------------------------------------------
101 NP_056762.1 --------------------------------------------------
102 YP_009508556.1 --------------------------------------------------
103 NP_569153.1 ---------------------------------------------ETTR-
104 NP_043924.1 --------------------------------------------------
105 YP_009243641.1 --------------------------------------------------
106 YP_004300274.1 --------------------------------------------------
107 NP_056848.1 --------------------------------------------------
108 YP_006495799.1 ---------------------------------------YKAPEESDEER
109 YP_009508409.1 --------------------------------------------------
110 NP_687035.1 --------------------------------------------------
111 NP_045937.2 --------------------------------------------------
112 NP_612577.1 --------------------------------------------MSLRNR
113 NP_619548.1 --------------------------------------MTSSSLFREGEL
114 NP_056880.1 --------------------------------------------------
115 YP_009121747.1 ----------------------------------------------IKDQ
116 YP_009113237.2 --------------------------------------EVEGKGKEKAEE
117 YP_009508408.1 -------------------------------------------------N
118 NP_955579.1 --------------------------------------------------
119 YP_009229919.1 ---------------------------------------------VHEAS
```

```
120 YP_004442833.1     --------------------------------------------------
121 NP_862833.2        --------------------------------------------------
122 NP_040563.1        --------------------------------------------------
123 YP_443922.1        --------------------------------------------------
124 NP_057933.2        --------------------------------------------------
125 YP_009116631.1     ----------------------------------------ETPESSTSNT
126 YP_009513249.1     --------------------------------------------------
127 YP_223871.1        --------------------------------------------------
128 NP_127504.1        ---------------------------------------FRR--SKTSKA
129 NP_044929.1        --------------------------------------------------
130 NP_057860.1        --------------------------------------------------
131 NP_040973.1        --------------------------------------------------
132 NP_777317.1        -----------------------------------DERTKNTELETKIKE
133 NP_054716.1        --------------------------------------------------
134 YP_001856242.1     --------------------------------------------------
135 NP_954565.2        --------------------------------------------------
136 NP_569150.1        -----------------------------------QKEKDEIQYLNEIIE
137 NP_659397.1        --------------------------------------MKRNTLFKWGEF
138 YP_009513211.1     --------------------------------------------------
139 YP_233107.1        ------------------------------------KQQKDIEALQQQNE
140 YP_009109692.1     --------------------------------------------------
141 NP_057849.4        --------------------------------------------------
142 NP_049560.1        --------------------------------------------------
143 YP_009506267.1     ----------------------------------------NKEVLDLQSY
144 NP_039820.1        --------------------------------------QFKEETRMAIAQ
145 NP_056790.1        --------------------------------------------------
146 YP_002321513.1     ----------------------------------------DGQEDASGAE
147 NP_955591.1        --------------------------------------------------
148 YP_001956722.2     --------------------------------------------------
149 NP_056902.1        --------------------------------------------------
150 NP_056886.1        --------------------------------------------------
151 NP_041261.1        --------------------------------------------------
```

```
001 YP_004442836.1     LEERIRKLEQQMKYRDEEMQVILK--------------------------
002 NP_056728.1        --------------------------------------------------
003 YP_009508537.1     --------------------------------------------------
004 YP_004732983.2     QEDLSLAVQFDDVR------------------------------------
005 YP_009508407.1     --------------------------------------------------
006 NP_040939.1        --------------------------------------------------
007 YP_003864102.1     --------------------------------------------------
008 YP_009002585.1     REGKAHIAGAVIAA------------------------------------
009 YP_003284237.1     ----IKDLKER---------------LQWLSQ------------------
010 YP_009268869.1     --------------------------------------------------
011 YP_009506251.1     AYLEEDTITRVVGH------------------------------------
012 NP_955611.1        --------------------------------------------------
013 YP_008567619.1     AELNKVGVIIPEEK------------------------------------
014 NP_149413.1        LTKELRQLEAGMEVNQ----------------------------------
015 YP_004442824.1     LKTEIRELRQENEALKTG--------------------------------
016 YP_001931967.1     --------------------------------------------------
017 YP_002455786.1     --------------------------------------------------
018 YP_009109694.1     --------------------------------------------------
019 NP_789739.1        --------------------------------------------------
020 NP_955619.1        --------------------------------------------------
021 YP_224289.1        MGQEMVKIQEKQQEAEKFLRREIKRREQANTE------------------
022 YP_009182100.1     ERNRKLVESYRKLKLFERYKEVRNEVLQQIPQHQIPINTIITVKSSPTEM
023 NP_705927.1        --------------------------------------------------
024 YP_009508577.1     --------------------------------------------------
025 YP_004442830.1     LTNKLKGYEQASSSSE----------------------------------
026 NP_758887.1        --------------------------------------------------
027 YP_009508406.1     --------------------------------------------------
028 YP_610965.1        CVEECSDEDTEEAA------------------------------------
029 YP_001931961.1     SLSSTQIYPIRKALMN----------------------------------
030 YP_241114.1        --------------------------------------------------
031 YP_006732334.1     NKKEILFKAIMAKIRQR---------------------------------
032 YP_009041481.1     EEDEEEEEEEIIVG------------------------------------
033 YP_001036293.1     -EFEQVNVLICDG-------------------------------------
034 YP_605811.1        LMGEITALEEGVKVHKEEAEELQEENQKLKEKILAFEKEA----------
035 NP_955577.1        --------------------------------------------------
036 YP_003208050.1     FIEESSTIHCEEDE------------------------------------
037 NP_789740.1        --------------------------------------------------
038 YP_004442839.1     MRKEIESLKAAK---EEENKAHEEEKKALKEEILALKRSI----------
```

```
039 YP_009507791.1   --------------------------------------------------
040 YP_004581513.1   ----------MIQG------------------------------------
041 NP_043933.1      E-------------------------------------------------
042 YP_009507248.1   --------------------------------------------------
043 YP_002117531.1   MAAEMERLQQQVS---------QLEEQIAQKA------------------
044 YP_004347415.1   --------------------------------------------------
045 NP_056891.1      --------------------------------------------------
046 YP_009513242.1   --------------------------------------------------
047 YP_009352866.1   DEGEQVGGVVFPEG------------------------------------
048 YP_009165750.1   -----------MDLL-----------------------------------
049 NP_040840.1      --------------------------------------------------
050 YP_004222728.1   --------------------------------------------------
051 YP_009506264.1   EKALLAELLEREQ-----AKCE-ALEEAQQTT------------------
052 YP_567050.1      --------------------------------------------------
053 YP_009508571.1   --------------------------------------------------
054 NP_056803.1      --------------------------------------------------
055 NP_955564.1      --------------------------------------------------
056 YP_009508411.1   LEEKRLGVIICEG-------------------------------------
057 YP_009140788.1   -----------EKV------------------------------------
058 YP_009508410.1   --------------------------------------------------
059 NP_040333.1      --------------------------------------------------
060 YP_008992013.1   KRRKSSRCFICRKKGHFAK-------------------------------
061 NP_041734.1      AYLEEDTISRVIGH------------------------------------
062 NP_056895.1      --------------------------------------------------
063 YP_009109689.1   --------------------------------------------------
064 YP_002519387.1   T-------------------------------------------------
065 YP_595725.1      LRKELREVEATLEVNK----------------------------------
066 YP_009508551.1   --------------------------------------------------
067 YP_001497148.1   --------------------------------------------------
068 NP_041186.1      --------------------------------------------------
069 NP_663784.1      --------------------------------------------------
070 YP_009508443.1   IEDMAVNVLICDG-------------------------------------
071 YP_003987465.1   TALLVEKLQEQLQ-----QALSNKTTCNEEVR------------------
072 NP_861410.1      --------------------------------------------------
073 YP_009345071.1   KQEKEEECLFIKEE------------------------------------
074 YP_009508582.1   --------------------------------------------------
075 NP_569141.1      --------------------------------------------------
076 YP_002916057.1   --------------------------------------------------
077 NP_777384.2      --------------------------------------------------
078 YP_233110.1      LKEEITALEEEVRIHKEEAEELQEENQKLKEKIIAFEKDV----------
079 NP_542258.1      --------------------------------------------------
080 YP_009130664.1   RQIETQRILS----------------------------------------
081 YP_009508566.1   --------------------------------------------------
082 YP_006607892.1   FLSSLKNPQIQMEQLSI---------------------------------
083 YP_009506270.1   LSERIKRLEQQQKY------------------------------------
084 NP_042513.1      --------------------------------------------------
085 YP_006907834.1   -----------MKPQPL---------------------------------
086 YP_004442827.1   LTEKLKGLEEASTS------------------------------------
087 YP_009408594.1   KGRESIQYLGEYVV------------------------------------
088 YP_009508561.1   --------------------------------------------------
089 NP_056907.1      --------------------------------------------------
090 YP_002308474.1   --------------------------------------------------
091 YP_007761644.1   HKSLSEQEIINEARIRAFITVIPRPIVTEPINNGQTSNQPQSPTETIEEI
092 NP_040550.1      --------------------------------------------------
093 NP_758808.1      FTGEIQIVLFNHNTAPYPVKKTYRLAQIIFEKFYTPIFIQEPFTSTQQGS
094 YP_009345075.1   YKEEIFITLYNASKEDLVFPAQSQIAQG----------------------
095 NP_068729.1      --------------------------------------------------
096 NP_047255.1      --------------------------------------------------
097 YP_006273075.1   -----------EKI------------------------------------
098 YP_009508888.1   --------------------------------------------------
099 NP_395469.1      --------------------------------------------------
100 YP_009508546.1   --------------------------------------------------
101 NP_056762.1      --------------------------------------------------
102 YP_009508556.1   --------------------------------------------------
103 NP_569153.1      -----------IIA------------------------------------
104 NP_043924.1      --------------------------------------------------
105 YP_009243641.1   --------------------------------------------------
106 YP_004300274.1   --------------------------------------------------
107 NP_056848.1      --------------------------------------------------
108 YP_006495799.1   VGALLAEGELEKLA------------------------------------
109 YP_009508409.1   --------------------------------------------------
110 NP_687035.1      --------------------------------------------------
111 NP_045937.2      --------------------------------------------------
```

```
112 NP_612577.1         --------------------------------------------------
113 NP_619548.1         GHFCLN--------------------------------------------
114 NP_056880.1         --------------------------------------------------
115 YP_009121747.1      WNEEKAKLEAQN---EYLQQENKRLREEGFQPYPP---------------
116 YP_009113237.2      ISEESSEEDAEIIA------------------------------------
117 YP_009508408.1      KEPEQLEVLICEG-------------------------------------
118 NP_955579.1         --------------------------------------------------
119 YP_009229919.1      SDEETAMAITEETS------------------------------------
120 YP_004442833.1      LRKRIAELEGASTS------------------------------------
121 NP_862833.2         --------------------------------------------------
122 NP_040563.1         --------------------------------------------------
123 YP_443922.1         --------------------------------------------------
124 NP_057933.2         --------------------------------------------------
125 YP_009116631.1      IGALLAEEGIEELA------------------------------------
126 YP_009513249.1      --------------------------------------------------
127 YP_223871.1         --------------------------------------------------
128 NP_127504.1         MRQKPRSCFTCGKIGHFSR-------------------------------
129 NP_044929.1         --------------------------------------------------
130 NP_057860.1         --------------------------------------------------
131 NP_040973.1         --------------------------------------------------
132 NP_777317.1         LQKELEQAKHQNTVLTAQAKGKTEE-------------------------
133 NP_054716.1         --------------------------------------------------
134 YP_001856242.1      --------------------------------------------------
135 NP_954565.2         --------------------------------------------------
136 NP_569150.1         LKERIKDLEQQQKD------------------------------------
137 NP_659397.1         SP------------------------------------------------
138 YP_009513211.1      --------------------------------------------------
139 YP_233107.1         LLLQLAKIDKKEIESLRS--------------------------------
140 YP_009109692.1      --------------------------------------------------
141 NP_057849.4         --------------------------------------------------
142 NP_049560.1         --------------------------------------------------
143 YP_009506267.1      IEEHDVNVLICEG-------------------------------------
144 NP_039820.1         LKEAIIVQEEDTIEER----------------------------------
145 NP_056790.1         --------------------------------------------------
146 YP_002321513.1      ATNDMDEILSVHSQHS----------------------------------
147 NP_955591.1         --------------------------------------------------
148 YP_001956722.2      --------------------------------------------------
149 NP_056902.1         --------------------------------------------------
150 NP_056886.1         --------------------------------------------------
151 NP_041261.1         --------------------------------------------------
```

```
001 YP_004442836.1      ------------QQREEKVNALEEVSVNALR-PRNNHLNIRCEVEIKEKK
002 NP_056728.1         ----MDHLLLKTQTQTEQVMNVTNP----------NSIYIKGRLYFKGYK
003 YP_009508537.1      ---------------------KPISYVSIVFSKTELKFTELEKLLTTVHK
004 YP_004732983.2      -------------------QQE-QAYTEMPRGAHNKLYHVVVTFRIPDAK
005 YP_009508407.1      --------------------------------------------------
006 NP_040939.1         ------------------------LWVNVVHNIKNLSI--PQQVIKAAQK
007 YP_003864102.1      ----------------------PLMWIHSHASPKRVLLPYYDAIADLIIL
008 YP_009002585.1      -------------------STETGREERRPVKG-NMLYNMDVEIDIPSVP
009 YP_003284237.1      --------EKVQMLTEEDEKVFSAS-GRSGP-RYNGLYNVKVGIEVEKEI
010 YP_009268869.1      ------------------------LWVNVVHNIKNLSQ--SQQIIKAAQK
011 YP_009506251.1      -------------------TTEGPSEAKKVVKKSNMLYNLEVILIIPEVG
012 NP_955611.1         ---------------------RPCLWLFS-TQPTKAFTAWLEVLTLLITK
013 YP_008567619.1      -------------------ICISKEGEKIKPISKSMLIKFQVKLEIPDVP
014 NP_149413.1         -----------AVESEEEDEVEEVTLMASAV--KDQMFRFPVVIEVPEVG
015 YP_004442824.1      ------------RPIIEDVNALEECIVSTLG-PRNNLLNMKVSFEVEERR
016 YP_001931967.1      ----------------MSKRNLTNP----------NSIYILGTFQFPGYK
017 YP_002455786.1      ---------------------WPLVWLHAPHPPT-SLCPWGHILACTVLT
018 YP_009109694.1      ---------------------RPVAYLSKKLDPVAAGWPPCLRIMAATAM
019 NP_789739.1         ------------------------LKTGKYARMRGAHTNDVKQLTEAVQK
020 NP_955619.1         ---------------------WPLVWLHAPLPHT-SQCPWGQLLASAVLL
021 YP_224289.1         --------KLALMMNEVCTEREYEYQINATT-VQNNLYNIKIGLEVDGEK
022 YP_009182100.1      LADIEKLMEEVKKLQQKESTPEDKPDATPEFRHKDNSIYISAAFRFKGYE
023 NP_705927.1         ------------------------LKTGKYARMRGAHTNDVKQLTEAVQK
024 YP_009508577.1      ---------------------KPIAYASHVFTNTELKFTPLEKLLVTMHK
025 YP_004442830.1      -----------KILMAKEKL--QETVLISAR--KDPMYRFDVHVEVEGK-
026 NP_758887.1         ------------------------LKVGKYARTKNAHTNELRVLAGLVQK
027 YP_009508406.1      --------------------------------------------------
028 YP_610965.1         ----------------ALLAEAAKIGQQHQKKLINRLYNTVVKFTIPGAE
029 YP_001931961.1      ----RVKNLINNQIYSGDVTNITNP----------HSIYIKGRLKFPGYK
030 YP_241114.1         ----------------------PLRWIYLSATPTKHLLPYYELVAKIVAK
```

```
031 YP_006732334.1   --KHQCLSLQTVDNGLCLIANRTSP----------NSIYIKAKLSFKGYK
032 YP_009041481.1   -------------------AMHESILSSTVKGATRRLYNIRVTFEIPDVP
033 YP_001036293.1   -------------------REK-SERIASIKERRNGLYNLMVQLEIPGAA
034 YP_605811.1      --------VQESEEVIELVNNVEEHLVLTGQ-QKNNLLNIKITLEIKEKK
035 NP_955577.1      ---------------------RPVAYLSKKLDTVASGWPPCLRMVAAIAI
036 YP_003208050.1   ----------------FGASAAVTG-----RKVINRLYNVKILFTIPNVK
037 NP_789740.1      ------------------------LKTGKYARMRGAHTNDVKQLTEAVQK
038 YP_004442839.1   --------T-EIQKPVELVNNVEEHLVLTGK-QRNNLLNIKITIEIKEKK
039 YP_009507791.1   ------------------------LWMGRMNRQKKKAENTCDIVLRAINK
040 YP_004581513.1   -------------------AVSPQIVSSASGELNNRLYNMKVCIRIRGCP
041 NP_043933.1      --DEEQLLESVDSEERFSTLTKTNP----------NSIYIRGNFYFKGYK
042 YP_009507248.1   ---------------------KPVCYASRTLNESELNYSTIEKELLAIVW
043 YP_002117531.1   --------ESVGMLMEENEQVFSASQALQPKGQYNGLYNLQIEICCDSWT
044 YP_004347415.1   ---------------------------------MNRMTYMTVKITIPTYM
045 NP_056891.1      ----------------------PIMWIHLPASPKKVLLPYYDAIADLIIL
046 YP_009513242.1   ---------------------RPIMYVNYVFSKAEVKFTQTEKMLTTMHK
047 YP_009352866.1   -------------------INSEECYSTSVKKASKKLYNVRVTFEIPGVE
048 YP_009165750.1   ------QKVQIQNNHQQLSLNATNT----------SSIYISGKLKFQGYK
049 NP_040840.1      ------------------------LWVNVVHSIKNLSQ--AQQIIKAAQK
050 YP_004222728.1   ---------------------KPCLWLFS-TQPTKAFTAWLEVLTLLITK
051 YP_009506264.1   --------ENALFLEEEESKVFSYERLRAGP-RYNGLYNLKIALEINGVI
052 YP_567050.1      ---------------------WPLVWLHTPHPPT-SLCPWGHLLACTILT
053 YP_009508571.1   ---------------------KPIMYLNYVFSKAELKFTLLEKLLTTMHK
054 NP_056803.1      ---------------------KPIMYLNYVFSKAELKFSMLEKLLTTMHK
055 NP_955564.1      ----------------------VVEWIHLPHISPKVITPYDIFCTQLIIK
056 YP_009508411.1   -------------------KGEQVLATTSS---RNGLYNLVVELSIPGIL
057 YP_009140788.1   -------------------LIAGAEERKRLVK--NMLYNFDIEFEIPDVP
058 YP_009508410.1   --------------------------------------------------
059 NP_040333.1      ---------------------RPVAYLSKKLDPVAAGWPPCLRMVAAIAV
060 YP_008992013.1   -----------DCPNKRAKSIRLVEHLQATTDYSPQQDELEFYFSEQDEP
061 NP_041734.1      -------------------TVE-QQEVRKPVKKGNMLYNLDVVLHIPEVG
062 NP_056895.1      ---------------------FPLAYFQTPLTDN-QASPWGLLLLLGCQY
063 YP_009109689.1   ---------------------RPVAYLSKKLDPVAAGWPPCLRIMAATAM
064 YP_002519387.1   -------LLMNLNLKTSGKSTKTNP----------YSTYVTVGFYFNGYK
065 YP_595725.1      -----------VEESEEE---AEEVMMASAV--KDEMYRFPVIIEVPEVG
066 YP_009508551.1   ---------------------KPIMYLNYVFSKAELKFSMLEKLLTTMHK
067 YP_001497148.1   ---------------------RPVAYLSKKLDPVAAGWPPCLRIMAATAM
068 NP_041186.1      ----------------------PLRWIYLSATPTKHLLPYYELVAKIIAK
069 NP_663784.1      ------------------------LKVGKYAKIKNTHTNGVRLLAQVVQK
070 YP_009508443.1   -------------------KDKHVAATLPLKERRNGLYNLIVELDIPGST
071 YP_003987465.1   --------EEVAVITEEEN--LSYSAIKSPT-RYNGLYNLKAKIEVEGET
072 NP_861410.1      ------------------MKSKGNP----------NATFITVKINDV---
073 YP_009345071.1   -------------------PEEEEQRVEETLAISGKLIIIEGRIEIEGVK
074 YP_009508582.1   ---------------------KPIAYASHVFTNTESKFTPLEKLLVTMHK
075 NP_569141.1      -----------------------------------MKIYILAKIIVEGYY
076 YP_002916057.1   ---------------------------------------MRVSIRIRGCP
077 NP_777384.2      ---------------------FPLAYFQTPLTDN-QASPWGLLLLLGCQY
078 YP_233110.1      --------TQGPEEVIELVNNVEEHLVLTGQ-QKNNLLNIKITLEVKEKR
079 NP_542258.1      ---------------------WPLVWLHTPHPAT-SLRPWGQLLANAIIT
080 YP_009130664.1   ---------------------IEKNELSPPKKTKNMLYNLILEINIPDVA
081 YP_009508566.1   ---------------------RPIAYASHVFNHTEQKFTPVEKLLTTMHK
082 YP_006607892.1   --HLQNHQVQIQNNSEMNYLNSTNP----------NSIYVKGILKFSGYK
083 YP_009506270.1   -------------REEEQVNALEEVSVNALR-PRNNHLNIKCEVEVKERK
084 NP_042513.1      ---------------------MSSK----------NSSFIKVKLFNK---
085 YP_006907834.1   --KTMEHLLQKTQIQTEQVMNITNP----------NSIYIRGRLYFSGYK
086 YP_004442827.1   -----------VLMAQEGQ----ETVMLTAR--KDPMYRFDVHVEVDGI-
087 YP_009408594.1   ----------------CRASTDQKE--YHPKRMINRLFNMKVTFHIPTMD
088 YP_009508561.1   ---------------------IPIAYASHVFSTAELKFTPLEKLLVTMHR
089 NP_056907.1      ---------------------WPLVWLHTPHPPT-SLCPWGHLLACTILT
090 YP_002308474.1   ---------------------WPLVWLHTPHPAT-SLRLWGQLLANAIIT
091 YP_007761644.1   DLQIQLLELKLKKVKLQKKLKSIPP----------PPLPNPDRIETVEES
092 NP_040550.1      ---------------------RPCLWLFS-TQPTKAFTAWLEVLTLLITK
093 NP_758808.1      SNFGSTAKPLQITENIEVMSETVANQVAKSSVLP-RLYSIQAHIHIAPDI
094 YP_009345075.1   -----------IVIPLAAQDTAMVIKEESARAVNNQLYNFEVEIMIPGVK
095 NP_068729.1      --------MNTEIVQKHRVLTKGNP----------NVTFIKVSIGKR---
096 NP_047255.1      ---------------------RPVAYLSKKLDTVASGWPPCLRMVAAIAI
097 YP_006273075.1   -------------------LAAGTEEKKKFVK--NMLYNFDIEFEIPDVS
098 YP_009508888.1   ---------------------KPILYINYVFSKAEEKFSMLEKLLTTLHK
099 NP_395469.1      ----------------------MNQ----------NNTFIKITISQK---
100 YP_009508546.1   ---------------------KPIMYLNYVFSKAELKFSILEKLLTTMHK
101 NP_056762.1      ----------NKSEITEIPTTSLAMRANESNYIKTSINKTAGCYVETKIS
102 YP_009508556.1   ---------------------RPIMYVNYVFSKAEVKFTQTEKILTTMHK
103 NP_569153.1      -------------------HGDTQVTKTRPVK--NMLYNMDVRMEIPGIP
```

```
104 NP_043924.1         -----------------WFVPEPYEGEQHQCRPHDDTITWRVSTRAMPTG
105 YP_009243641.1      ---------------------EPCEWLYTRHRFHRSIMSYEQQIAILIQQ
106 YP_004300274.1      -----------------------------------MKIHILVKVICEGYK
107 NP_056848.1         ---------------------------------MNKITYMTIKISIPKYM
108 YP_006495799.1      -------------------AVAGESYSRMPKGGLNKLYHLNVQFVIPKAE
109 YP_009508409.1      --------------------------------------------------
110 NP_687035.1         ------------------------LKVGRYAKQRETHTNDLRTLAHLVQK
111 NP_045937.2         ---------------------RPIAFLSSKFDAIESGLPPCLKACASIHR
112 NP_612577.1         ----------------------TNP----------NSIYVKGILKFPGYQ
113 NP_619548.1         -------------KQEMLHLNVTNP----------NSIYIEGKLSFEGYK
114 NP_056880.1         ----------------------VVEWIHLPHISPKVITPYDIFCTQLIIK
115 YP_009121747.1      ------LGPHQINTLIEVAMRADGSEQPKVRKINNQLYNVEVEFDIPGIK
116 YP_009113237.2      -------------------VQKEVHHAEGSSKRRNNLYNIDVILEVIDEA
117 YP_009508408.1      -------------------KERQYIAAASLKERRNGLYNLMVEIDIPGSL
118 NP_955579.1         ---------------------RPVAYLSKKLDTVASGWPPCLRMVAAIAI
119 YP_009229919.1      ----------------LSSTTEAPS---HGRKVINRLYNMVVNINIPGVK
120 YP_004442833.1      -----------IILTAQEQY--NETVLVTQR--KDCMYRFDVFVEVEGK-
121 NP_862833.2         ----------------------PLRWIYLSATPAKHLLPYYELIAKIVAK
122 NP_040563.1         ---------------------------GGVKGLNYDHSNKIKKILRTMNE
123 YP_443922.1         ---------------------RVLMYHSSKLDHIETGQTTCSRYVAAVAK
124 NP_057933.2         ---------------------RPVAYLSKKLDPVAAGWPPCLRMVAAIAV
125 YP_009116631.1      -------------------AAVDTAYTEMPKGGLNKLYNTIVEFVIPQEK
126 YP_009513249.1      ---------------------KPISYVSIVFSKTELKFTELEKLLTTVHK
127 YP_223871.1         ---------------------RPVAYLSKRLDPVAAGWPRCLRAIAAAAL
128 NP_127504.1         -----------NCP-QNKKSIKLISEIQKYTGID-IEDDLESVFSIEDEP
129 NP_044929.1         ---------------------KPIAYNNALFTSTELKFTPTEKIMATIHK
130 NP_057860.1         ---------------------WPLVWLHAPLPHT-SQCPWGQLLASAVLL
131 NP_040973.1         ------------------------LWYGKMSRQKKKAENTCDIALRACYK
132 NP_777317.1         ------------GEALMHYADEEDCVVNLLR-PRNNLLNIKVAVEVGSTK
133 NP_054716.1         ---------------------KPIAYHNVVFSKTELKFTITEKVMTTIHK
134 YP_001856242.1      ------------------------LKTGKYARMRGAHTNDVKQLTEAVQK
135 NP_954565.2         ----------------------PIMWIHLPASPKKVLLPYYDAIADLIIL
136 NP_569150.1         -------------KEE-QVNVLEEVSINALR-PRNNHLNIKCEIEVKNKK
137 NP_659397.1         -------------NKFCNLANKTNP----------NSIYIEAKVKFKGYQ
138 YP_009513211.1      ---------------------RPVAYLSKKLDPVASGWPTCLKAIAAVAL
139 YP_233107.1         -------------QHPLPVLHLDDFAVTVVQ-PSNNLLNIKVIVEIDGKQ
140 YP_009109692.1      ---------------------RPVAYLSKKLDPVAAGWPPCLRIMAATAM
141 NP_057849.4         ------------------------LKTGKYARMRGAHTNDVKQLTEAVQK
142 NP_049560.1         ---------------------WPLVWLHAPLPHT-SQCPWGQLLASAVLL
143 YP_009506267.1      -------------------NDKQVAASIPVQDRRNGLYNLIVELEIPGCA
144 NP_039820.1         ------------CAMILEEKHTENIYSATAKAEYNGLYNVKVGIKPDNME
145 NP_056790.1         ---------------------RPVAYLSKKLDPVASGWPTCLKAVAAVAL
146 YP_002321513.1      ----------------MKHFEFDALVNIERPGQLNKLYNFGCEIQCNGGS
147 NP_955591.1         ---------------------RPVAYLSKKLDPVAAGWPPCLRMVAAIAV
148 YP_001956722.2      ---------------------RPIMYLNYVYTKAEVKFTNTEKLLTTIHK
149 NP_056902.1         ------------------------LWAGKKIMKANKGWSTVKNLMLLLQH
150 NP_056886.1         ---------------------RPCLWLFS-TQPTKAFTAWLEVLTLLITK
151 NP_041261.1         -------------------NGSPLLWLHLPASPSKVLLTYPSLLAMLIIK
```

```
001 YP_004442836.1      V----ILN-AILDTGATVCVADSRMIPAEMR--EQAKNRIIIRGVNGVTE
002 NP_056728.1         K----IRLHCFVDTGASLCIASKFVIPEEHWVNAER--PIMVKIADGSSI
003 YP_009508537.1      G-----LLKALDLSMGQNIHVYSPIVSMQNIQKTP-----QTAKKALASR
004 YP_004732983.2      GQLLEFIINAIIDTGCTCCCINLTKVPDGAI--ENASIIQEVSGINSKTV
005 YP_009508407.1      --------------------------------------------------
006 NP_040939.1         L-----TQEVIIRTGKIPWILLP------------------GKEEDWRLE
007 YP_003864102.1      G-----RGHSRTYFGIEPSTIIQ------------------PYSKSQIDW
008 YP_009002585.1      R----FTVKAILDTGATSCCISMDKIPKAAI--EPNTFEVTFRGINSVQK
009 YP_003284237.1      K----YLN-AIVDTGATTCVVREARLTEKML--EESPVNVTLRGMNSISR
010 YP_009268869.1      L-----TQEVIIRIGKIPWILLP------------------GKEEDWILE
011 YP_009506251.1      KP---LRVKAILDTGATTCCININSVPKMAI--EQNTFLVQFRGINSTQS
012 NP_955611.1         L-----RASAVRTFGKEVDILLL------------------PACFRED--
013 YP_008567619.1      E----FTVNAILDTGATTCCIDERVIPQEAL--EANPYLVHFRGINSKTT
014 NP_149413.1         K----VQLTALLDTGATRSCINKPFIEDKML--QDTAFKVKISGVNSVTY
015 YP_004442824.1      I----MLN-AILDTGATVSVCSEEIVPQEFR--TNAAVKALIKGVNGTTA
016 YP_001931967.1      I----IRIHCLVDTGASMCIASRYIIPPELWKPTKK--VHKVKTANG-QV
017 YP_002455786.1      L-----IKYALQHYGQLCKSFHH------------------NMSTQALHD
018 YP_009109694.1      L-----VKDSAKLTLGQPLTVITPHTLEAIVRQPPDRWITNARLTHYQAL
019 NP_789739.1         I-----TTESIVIWGKTPKFKLP------------------IQKETWETW
020 NP_955619.1         L-----IKYTLQSYGLLCQTIHH------------------NISTQTFNQ
021 YP_224289.1         K----FLN-AILDTGAATCVVSQAKVSERFL--EPSAMKVELRGVNGTSK
022 YP_009182100.1      E----YRLHCLVDTGASITLATQWAIPEEKWITIPHGNEIKITVANGETC
```

```
023 NP_705927.1      I-----TTESIVIWGKTPKFKLP------------------IQKETWETW
024 YP_009508577.1   A-----IIKAIDLALGQPIEVYSPIISMQKLQKTP-----LPERKALSTR
025 YP_004442830.1   K----T[illegible]LKALLDTGATKSCINIDHLDQSYL--TDSKFAVDIEGVNSSTR
026 NP_758887.1      I-----AKEALVIWGKLPKFYLP------------------IEREVWDQW
027 YP_009508406.1   --------------------------------------------------
028 YP_610965.1      P----F[illegible]AIAIFDTGATSCVLDSKIVPEKAL--TALTYNVQFSGVNSLDT
029 YP_001931961.1   I----MNLDCFVDTGASICLCSKYVIPEEHWKDAER--SIKVKIADDSVI
030 YP_241114.1      G-----[illegible]HEAIQYFGMEPPFICV------------------PYALEQQDW
031 YP_006732334.1   K----FILHCYVDTGAGLCLASKHVIPDELWENAPR--TIEVTVANQEKV
032 YP_009041481.1   ----KFTAMAILDTGATCCCIDQRVVPAGAT--EDAAMQSTFLGVNSKTI
033 YP_001036293.1   P----IQVNAILDTGATTCCINEEGLPKAIL--EANTFEVKFTGANSEMV
034 YP_605811.1      I----TMN-AILDTGAAICVCDGNMVDEYFR--RPSMMNAFIKGVNGITN
035 NP_955577.1      L-----VKDAGKLTLGQPLTILTSHPVEALVRQPPNKWLSNARMTHYQAM
036 YP_003208050.1   P----FTLNAIVDTGATSCCADIRAIPEEAL--EELNYTVNFFGVNSESQ
037 NP_789740.1      I-----TTESIVIWGKTPKFKLP------------------IQKETWETW
038 YP_004442839.1   I----TMN-AILDTGAAICVCDGQIVDNYFR--RPSMMNAFIKGVNGITN
039 YP_009507791.1   V-----[illegible]QEAIVRLGIEPVYQIP------------------CSREIWESY
040 YP_004581513.1   E----FSVNAILDTGATVCCIEEERVPKEGL--EESKMTAQFTGLNSTQQ
041 NP_043933.1      K----YSLDLYVDTGASMCTANKHVIPEEFWVNAKN--PIRARIANDSIM
042 YP_009507248.1   AT----[illegible]YFRPYLFGRKFKILTDHKPLQWMMN--------LKDPNSRMTR
043 YP_002117531.1   A----FIVNAILDTGATTSCIRTGKAPRETV--EDSKTQAVLKGVNSVSI
044 YP_004347415.1   S----RIYHGLFDTGANTCVCKTKVLPPEKWIEIDN---KVLTGFSEEKN
045 NP_056891.1      G-----[illegible]DHSKKYFGIEPSTIIQ------------------PYSKSQIDW
046 YP_009513242.1   G-----IIKAMDLAMGQEILVYSPIVSMTKIQKTP-----LPERKALPVR
047 YP_009352866.1   ----KFTAMAIIDTGATCCCIDQRVVPEGAT--EEAAVPSVFHGINSQSL
048 YP_009165750.1   Q----LILHCFVDTGASMCVASKHVIPEEHWENSSR--QLLVKTANG-MI
049 NP_040840.1      L-----TQEVIIRTGKIPWILLP------------------GREEDWILE
050 YP_004222728.1   L-----[illegible]ASAVRTFGKEVDTLLL------------------PACFRED--
051 YP_009506264.1   T----RIN-AILDTGATICVVRSSRIPANCL--EEASMNYTIKGVNSITK
052 YP_567050.1      L-----IKYSLQHYGQLCQSFHH------------------NMSNTALHD
053 YP_009508571.1   A-----IIKAMDLAMGQEILVYSPVVSMTKIQKTP-----IPERKALPIR
054 NP_056803.1      A-----IIKAMDLAMGQEILVYSPIVSMTKIQKTP-----LPERKALPIR
055 NP_955564.1      G-----[illegible]HRSKELFSKDPDYIVV------------------PYTKVQFDL
056 YP_009508411.1   P----FSVNAILDTGATNCCINEEGLPKGAL--EDNTFEVTFTGANSVMK
057 YP_009140788.1   K----F[illegible]VRAILDTGASTCCVNEGAVPKEAI--EDSPYEVQLNGVNSVQK
058 YP_009508410.1   --------------------------------------------------
059 NP_040333.1      L-----TKDAGKLTMGQPLVILAPHAVEALVKQPPDRWLSNARMTHYQAL
060 YP_008992013.1   NDETVFALQQSSDDS------DSDQSQVIFHQQLLSLDTTVPIPSIKLQI
061 NP_041734.1      RP---I[illegible]VKAILDTGATTCCININSVPQTAI--EQNTFLVQFRGINSTQS
062 NP_056895.1      L-----QTQALSSYAKPILKYYH------------------NLPKTSLDN
063 YP_009109689.1   L-----VKDSAKLTLGQPLTVITPHTLEAIVRQPPDRWITNARLTHYQAL
064 YP_002519387.1   G----F[illegible]LHAYVDTGATSCTASPHIIPTELWEELPK--PILVNIANDTNI
065 YP_595725.1      K----VQLTALLDTGATRSCINQVFIEEKFL--QPTKFKVKIHGVNSVTK
066 YP_009508551.1   A-----IIKAMDLAMGQEILVYSPIVSMTKIQKTP-----LPERKALPIR
067 YP_001497148.1   L-----VKDSAKLTLGQPLTVITPHALEAIVRQPPDRWITNARLTHYQAL
068 NP_041186.1      G-----[illegible]HEAIQYFGMEPPFICV------------------PYALEQQDW
069 NP_663784.1      I-----GKEALVIWGRIPKFHLP------------------VERETWEQW
070 YP_009508443.1   P----L[illegible]VNAILDTGATTCCINEEGLPKEIL--EDNTFEVKFTGANSVMT
071 YP_003987465.1   V----VIN-AILDTGATACIIQEAKVPDRPC--EQAAVSYTLHGVNSTTS
072 NP_861410.1      ------[illegible]INAYVDTGATICLADPK--IKLKWVKMEK--PIKISIADKSVQ
073 YP_009345071.1   P----FTVKALLDTGASCCNIKKEFVPVDAV--EQCNYPVLIHGVNSTLE
074 YP_009508582.1   A-----IIKAIDLALGQPIEVYSPIVSMQKLQKTP-----LPERKALSTR
075 NP_569141.1      N----RYYTPMIDTGAEANICKYNCLPTDKWEKLKTP--MVVTGFNNEGS
076 YP_002916057.1   E----F[illegible]VNAILDTGATVCCIEEERVPKEGL--EESRMTAQFTGLNSTQQ
077 NP_777384.2      L-----QTQALSSYAKPILKYYH------------------NLPKTSLDN
078 YP_233110.1      I----TMN-AILDTGAAICVCDGQMVNEYFR--RPSMMNAFIKGVNGITN
079 NP_542258.1      L-----IKYSLQHYGQICKSFHH------------------NISNQALTY
080 YP_009130664.1   N----FSVHAILDTGASTCCINAGAIPENAV--EESPFMASSSGINSKVS
081 YP_009508566.1   A-----IIKGIDLAIGQPIEIYSPIVSMQKLQKIT-----LPERKALSTR
082 YP_006607892.1   Q----L[illegible]LHCFVDTGASMCVASKHVIPEEHWENSSR--HISVKTANG-LV
083 YP_009506270.1   V----TIN-AILDTGATVCVADERMIPSSMR--EQAKNRIIIRGVNGVTE
084 NP_042513.1      ------YLYAYIDTGATICLAQAKILPIKYWKKMIK--PIKVRIANNKVI
085 YP_006907834.1   I----V[illegible]LDCFVDTGASLCIASKHVIPEERWEAAPR--KINVKIANESTI
086 YP_004442827.1   K----T[illegible]LKALLDTGATKSCINAKFIDERFL--TEAKFMVNINGVNSSTK
087 YP_009408594.1   P----FTVSAILDTGATACCIDKGSVPEDAL--EENSYTVYFNGVNSRQA
088 YP_009508561.1   A-----ILKGIDLALGQPIKVYSPIASMQKLQKTP-----IPERKALSTR
089 NP_056907.1      L-----IKYSLQHYGQLCQSFHH------------------NMSNTALHD
090 YP_002308474.1   L-----IKYSLQHYGQVCKSFHH------------------NISNQALTH
091 YP_007761644.1   SE---E[illegible]IEETEASSSSSEQEESSDESIAETLSSNESFEIIPEDKNEENN
092 NP_040550.1      L-----[illegible]ASAVRTFGKEVDILLL------------------PACFRED--
093 NP_758808.1      V----ISTTAIIDTGATVCCISEKIVPEAAK--EQLNYKVNISGISSQQQ
094 YP_009345075.1   P----FTAKAILDTGATKCCIDPKVVPKEAI--TGTLYPVTVYGVNSETI
095 NP_068729.1      ------[illegible]FLAYIDTGATLCFGKRK--ISNNWEILKQ--PKEIIIADKSKH
```

096	NP_047255.1	L-----VKDAGKLTLGQPLTILTSHPVEALVRQPPNKWLSNARMTHYQAM
097	YP_006273075.1	R----FIVRAILDTGASTCCINEGAVPKEAL--EESPYEVQLNGVNSVQK
098	YP_009508888.1	A-----IIKAVDLAMGTEIMVYSPIVSMTKIQKTP----LPERKALPVR
099	NP_395469.1	------TILAYIDTGASLCLLPEYNLPKQLWKELKK--PITIRVADKREL
100	YP_009508546.1	A-----IIKAMDLAMGQEILVYSPIVSMTKIQKTP----LPERKALPIR
101	NP_056762.1	FNNENRIITALIDSGSTHNIICPTLIPASWINNTHREIIMFAVDNSKYNL
102	YP_009508556.1	G-----IIKAMDLAMGQEILVYSPIVSMTKIQKTP----LPERKALPVR
103	NP_569153.1	A----FTVKAILDTGATTCCIDSRSVPKDAL--EENSFVVNFSGINSKQQ
104	NP_043924.1	EKWWIDGSRYWDHDKGGYVTGWAALREDKKNQLGGALEGHVSAQVAELVA
105	YP_009243641.1	G-----EQRCISLSGYDVSTIVL------------------PLTKSQFFF
106	YP_004300274.1	S----RYYTPMIDTGAEISICKMNCLPDEYWKETRND--LIVRGFNNEGS
107	NP_056848.1	S----RIYHGLFDTGANICICKKKVLPDELWHKTEN---LVLRGFNDEKH
108	YP_006495799.1	GGHVSFTVAAIIDTGCTCCCINGAKVPEEAQ--EEASYAMTFAGVNSRGT
109	YP_009508409.1	--
110	NP_687035.1	I-----GKEALTIWGRLPRVQLP------------------VDKKTWDMW
111	NP_045937.2	S-----ITQADSFILGAPLIIYTTHAICTLLQRDRSQLVTASRFSKWEAD
112	NP_612577.1	TN---LILHCYVDTGSSLCMASKYVIPEEYWQTAEK--PLNIKIANGKII
113	NP_619548.1	S----FNIHCYVDTGASLCIASRYIIPEELWENSPK--DIQVKIANQELI
114	NP_056880.1	G-----EHRSKELFSKDPDYIVV------------------PYTKVQFDL
115	YP_009121747.1	L----FEVKAIIDTGATSCCINKVIVPREAL--EEMNKTVFFNGLNSRQP
116	YP_009113237.2	P----VTVKAIIDTGATVCCIDERTVPAQIL--EENTYRVEFSGVNSKSI
117	YP_009508408.1	P----INVNAILDTGATTCCINEKGLPKEIL--EDNTYEVKFVGANSEMT
118	NP_955579.1	L-----VKDAGKLTLGQPLTILTSHPVEALVRQPPNKWLSNARMTHYQAM
119	YP_009229919.1	P----FSVRAILDTGATTCCVDKGAVPAEAL--EKSPYAVHFSGINSRQL
120	YP_004442833.1	K----EELKALLDTGATKSCISERYIPKEFL--TDSKFTVNIAGVNSMTR
121	NP_862833.2	G-----EYEAIQYFGLEPPFICV------------------PYALEQQDW
122	NP_040563.1	L-----NRTVVIMTGREASFLLP------------------GSSEDWEAA
123	YP_443922.1	A-----IEKTAHLVMCHKMEIHTHHGVAAYLTAKEFTFSADR-----KNK
124	NP_057933.2	L-----TKDAGKLTMGQPLVILAPHAVEALVKQPPDRWLSNARMTHYQAL
125	YP_009116631.1	GAPTKFEVRAVIDTGCTCTCINSKKVPKEAL--EEAKYQMNFAGVNSTGE
126	YP_009513249.1	G-----ILKALDLSMGQNIHVYSPIVSMQNIQKTP----QTAKKALASR
127	YP_223871.1	L-----TREASKLTFGQDIEITSSHNLESLLRSPPDKWLTNARITQYQVL
128	NP_127504.1	SEDTLFSLEFYEEYAGEQYQITSYEAPKTENPPLPKIHTIVEIPQTEVKI
129	NP_044929.1	G-----ILKALDLSLGKEIHVYSAIASMTKLQKTP----LSERKALSIR
130	NP_057860.1	L-----IKYTLQSYGLLCQTIHH------------------NISTQTFNQ
131	NP_040973.1	I-----EEESIIRIGKEPRYEIP------------------TSREAWESN
132	NP_777317.1	F----NIN-AILDTGATVCVCSEEMIPKEIR--EPSKTNTVIRGVNGANK
133	NP_054716.1	A-----ILKAFDLAMGQPIWVYSPIHSMTRIQKTP----LTERKALSIR
134	YP_001856242.1	I-----TTESIVIWGKTPKFKLP------------------IQKETWETW
135	NP_954565.2	G-----EDHSKKYFGIEPSTIIQ------------------PYSKSQIDW
136	NP_569150.1	V----VIN-AILDTGATVCVADERMIPSGMK--EQAKNKIIIRGVNGVTE
137	NP_659397.1	T----LSLHCYVDTGASLCLASKHVIPDDFWENAPR--SIPVKIANQSSI
138	YP_009513211.1	L-----IKDADKLTLGQNVLVIAPHNLESIVRQPPDRWMTNARMTHYQSL
139	YP_233107.1	L----PIN-AILDTGAAICVCNGEVIPQEYR--RSSLTDSLIQGVNGATR
140	YP_009109692.1	L-----VKDSAKLTLGQPLTVITPHTLEAIVRQPPDRWITNARLTHYQAL
141	NP_057849.4	I-----TTESIVIWGKTPKFKLP------------------IQKETWETW
142	NP_049560.1	L-----IKYTLQSYGLLCQTIHH------------------NISTQTFDQ
143	YP_009506267.1	P----LEVNAILDTGATTCCINSEGLPKEMM--EDNTFTVKFTGANSVMK
144	NP_039820.1	P----YYINAIVDTGATACLIQISAIPENYY--EDAKVTVNFRSVLGIGT
145	NP_056790.1	L-----IKDADKLTLGQNVTVIASHSLESIVRQPPDRWMTNARMTHYQSL
146	YP_002321513.1	P----FTARAILDTGASICCICTSITPRNCL--VET-KEASFNGITSSSK
147	NP_955591.1	L-----TKDAGKLTMGQPLVILAPHAVEALVKQPPDRWLSNARMTHYQAL
148	YP_001956722.2	G-----IIKALDLGMGQEILVYSPIVSMTKIQKTP----LPERKALPIR
149	NP_056902.1	V-----ATESITRVGKCPTFKVP------------------FTKEQVMWE
150	NP_056886.1	L-----EASAVRTFGKEVDILLL------------------PACFRED--
151	NP_041261.1	G-----EYTGRQLFGRDPHSIII------------------PYTQDQLTW

001	YP_004442836.1	VNEVTAI-GRLWVGKQ-----WFTLPQTFIMP---TLGDGVHMIIGMNFI
002	NP_056728.1	TISKVCKDIDLIIAGE-----IFRIPTVYQQ------ESGIDFIIGNNFC
003	YP_009508537.1	WLSWLSYLEDPRIRFFYDPQMPALKDL--PAVDIGENN-KKHP-SNFQHV
004	YP_004732983.2	VTKKLRQ-GKMILAGN-----DFYIPYVSAFE---MNMPGIDMLIGCNFI
005	YP_009508407.1	--
006	NP_040939.1	LQLG-NITWMP--------------------------KFWSCYRGHTRWR
007	YP_003864102.1	LMQNTETWPIACASYAGKIDNHYPPDK----------LLQFCRLHTFIFP
008	YP_009002585.1	SNKKLKY-GQMRIGGH-----MFRIPYTYCFES-LTLGDDVQYVLGCNFI
009	YP_003284237.1	ASKVVKQ-GKIWIGEQ-----YFRTPRTFALDI--SLSGGIDMILGCNFI
010	YP_009268869.1	LQIG-NITWMP--------------------------SFWSCYRGSIRWK
011	YP_009506251.1	VDKKLRY-GRMTISGH-----QFRFPYCYAFP--LSLGDGIEMILGCNFI
012	NP_955611.1	-LPLPEGILLALKGFAGKIRSSDTPS-----------IFDIARPLHVSLK
013	YP_008567619.1	ANKKLKY-GRMIIGDQ-----QFKVPYTYAFP--MTISDDIQMILGCNFI
014	NP_149413.1	LDKQVKDGAKLWAGSS-----WFRLPLTYVGNM--HMGEKTQMLIGCNFL

```
015 YP_004442824.1   VTEVLKE-GKLWVGNQ-----YFRMPRTFVMP---KLSEGLHLIIGLNFI
016 YP_001931967.1   TIDKVCLSQVITVAGK-----EFLIPTIYQL------NTGIDMVLGNNFL
017 YP_002455786.1   FVKNSSHPSVAILIHHMHRFCDLGRQPPGPWRTLLQLPALLREPQLLRPA
018 YP_009109694.1   LLDTDRVQFGPPVTLNPATLLPVPENQPSPHDCRQVLAETHGTREDLKDQ
019 NP_789739.1      WTEYWQATWIP-------------------------EWEFVNTPPLVKLW
020 NP_955619.1      FIQTSDHPSVPILLHHSHRFKNLGAQTGELWNTFLKTTAPLAPVKALMPV
021 YP_224289.1      VNKVLKE-GKIWIGNQ-----YFRLPRTYALEI--ELSGGIDMIIGTNFL
022 YP_009182100.1   KIDKYATNVPIYISGI-----KFTIEKVLQFN-----KQGCDFLIGNDFF
023 NP_705927.1      WTEYWQATWIP-------------------------EWEFVNTPPLVKLW
024 YP_009508577.1   WITWLSYLEDPRITFYYDKTLPDLKNV--PETVTDKKP-KMXPIIEYAAV
025 YP_004442830.1   VTKQLKE-AKLWVGGT-----FFTLPITYVGKL--DLGERTQMVIGCNFI
026 NP_758887.1      WPEYWQATWIP-------------------------EWEFVSTPHLIGLW
027 YP_009508406.1   --------------------------------------------------
028 YP_610965.1      SRQRLKE-GRMTIGDH-----DFPIPLVYCFP-LDSSRDGVQMLIGCNFI
029 YP_001931961.1   EINKVCENLNMYIAGE-----CFHIPRVFMQ------ESGIDFILGNNFC
030 YP_241114.1      LFQFSDNWAIAFANYPGRIIHHYPPDK----------LLQFASSHAFIFP
031 YP_006732334.1   QINKVCKNLILQIVGE-----EFVIPTIYQQ------ETGIDILLGNNFC
032 YP_009041481.1   AKKRIKA-GKMFLGDN-----YFKIPYIYCFQ---MQVKNIDMLIGCNFI
033 YP_001036293.1   ARKKLKG-GIMKIGDN-----QFRIPYTFAFP--LKLGGGEQLIIGCNFI
034 YP_605811.1      VKEILEE-GKIWIGNQ-----WFRIPRTYIMP---QLSEGLHFIIGMNFI
035 NP_955577.1      LLDAERVHFGPTVSLNPATLLPLPSGG-NHHDCLQILAETHGTRPDLTDQ
036 YP_003208050.1   ARHKIKG-GQISMGES-----NFRLPFIYAFK-MDS-KDEIQMLLGCNFL
037 NP_789740.1      WTEYWQATWIP-------------------------EWEFVNTPPLVKLW
038 YP_004442839.1   VQEILEE-GRIWIGNQ-----WFRIPRTYVMP---QLSEGLHFIIGMNFI
039 YP_009507791.1   LITS-IYLKNPPP----------------------EVQFIHASLMMARML
040 YP_004581513.1   TRKKLKE-GYMLIGEH-----MFPLPFVYALN-PMRIGRGIQFIIGCNFI
041 NP_043933.1      TFNKVAELMQVQIADE-----TFIIPTLYQAT-----TKG-DITLGNNFC
042 YP_009507248.1   WRLRLSEYDFSVVYKK---------------------GKSNTNADALSRV
043 YP_002117531.1   GTKSLRP-GLMKIQGG-----TYRIPKTYVIDI--QLSEGIDMILGCNFI
044 YP_004347415.1   VIKYRADNIKIMIAKK-----EFIIPYIYAMD-----NINADIIIGATFY
045 NP_056891.1      LMQNTEMWPIACASFVGILDNHYPPNK----------LIQFCKLHTFVFP
046 YP_009513242.1   WITWMTYLEDPRIQFHYDKTLPELQQT--PSVTEDVIA-KTKHPSEFAMV
047 YP_009352866.1   AKKRLKA-GKIFLGDN-----YFKIPYIYCFQ---MQVKGIDMLIGCNFI
048 YP_009165750.1   TINKVCKNIDIMIADT-----IFHIPTIYQQ------EIGIDLLLGNNFL
049 NP_040840.1      LQMG-NINWMP--------------------------SFWSCYKGSVRWK
050 YP_004222728.1   -LPLPEGILLALKGFAGKIRSSDTPS-----------IFDIARPLHVSLK
051 YP_009506264.1   TNQVLKG-GKIWIGEQ-----YYRIPRTMALEV--ALSEGIDMIIGCNFI
052 YP_567050.1      FVKNSPHPSVGILIHHMGRFHNLGSQPSGPWRTLLHLPALLQEPRLLRPL
053 YP_009508571.1   WITWMTYLEDPRIQFHYDKTLPELKNI--PDVLTENSSKIMIHPSQYNSV
054 NP_056803.1      WITWMTYLEDPRIQFHYDKTLPELKHI--PDVYTSSIP-PLKHPSQYEGV
055 NP_955564.1      LLQEKEDWPISLLGFLGEVHFHLPKDP----------LLTFTLQTAIIFP
056 YP_009508411.1   ANKKLKG-GMMKIGDH-----QFRIPYTFAFP--LRLGGGEQLILGCNFI
057 YP_009140788.1   TKKKLKY-GRMMIGMN-----SFRIPFTYSLP--LVIGDNIQMIVGCNFI
058 YP_009508410.1   --------------------------------------------------
059 NP_040333.1      LLDTDRVQFGPIVALNPATLLPLPEEG-LQHDCLDILAEAHGTRPDLTDQ
060 YP_008992013.1   LPSKFQRPIPAIGLIDTGAQRSMINPHILPPDSWTQSEEHFKAVNGKLFT
061 NP_041734.1      VDKKLKY-GRMTISNH-----QFRIPYCYAFP--LSLGDGIEMILGCNFI
062 NP_056895.1      WIQSSEDPRVQELLQLWPQISSQGIQPPGPWKTLITRAEVFLTP------
063 YP_009109689.1   LLDTDRVQFGPPVTLNPATLLPVPENQPSPHDCRQVLAETHGTREDLKDQ
064 YP_002519387.1   EIRYVTRNLKVSMTDTNGNKHTFIIPTVYQQ------NTGMDFIFGQNFL
065 YP_595725.1      LDRQVKDGAKLWAGEN-----WFRLPITYVGPM--YMGEKTQMLIGCNFM
066 YP_009508551.1   WITWMTYLEDPRIQFHYDKTLPELKHI--PDVYTSSQS-PVKHPSQYEGV
067 YP_001497148.1   LLDTDRVQFGPPVTLNPATLLPAPKDQQSAHDCRQVLAETHGTREDLKDQ
068 NP_041186.1      LFQFSDNWSIAFANYPGQITHHYPSDK----------LLQFASSHAFIFP
069 NP_663784.1      WDNYWQVTWIP-------------------------EWDFVSTPPLVRLT
070 YP_009508443.1   AIKKLKN-GTMRIGDH-----QFRIPYTYAFP--LKLGGGEQLILGCNFI
071 YP_003987465.1   GRKVLKG-GKLWLGEQ-----FFRLPRTLVADM--VLTEGVEMIVGCNFL
072 NP_861410.1      EIWHRAEMVEIWIRNY-----KFVAATVCQK------SSGMDFVIGNNFL
073 YP_009345071.1   VNQKLKQ-GKLIIGSQ-----EFRLPYTYVLPMNKNLVTDMQMIIGCNFI
074 YP_009508582.1   WITWLSYLEDPRIIFHYDKTLPDLKNV--PETITEKQP-KILPIIEYAAV
075 NP_569141.1      MINYKARNVKIQIWDK-----ILTIEEIYNFE-----FTTKDMLLGMPFL
076 YP_002916057.1   TRKKLKE-GYMIIADH-----TFPLPFVYALN-PMRIGRGIQFIIGCNFI
077 NP_777384.2      WIQSSEDPRVQELLQLWPQISSQGIQPPGPWKTLITRAEVFLTP------
078 YP_233110.1      VKEILEE-GKIWIGNQ-----WFRIPRTYIMP---QLSEGLHFIIGMNFI
079 NP_542258.1      YLHTSDQSSVAILLQHSHRFHNLGAQPSGPWRSLLQVPQIFQNIDVLRPP
080 YP_009130664.1   TNKKLRY-GKMIIGDN-----AFRIPYTYVFP--MQLGDDIQMILGCNFI
081 YP_009508566.1   WLSWLSYIEDPRFLFIYDKTLPDLKEM--PPTQTDDYN-PMLPLHQYLAV
082 YP_006607892.1   TLTKVCKNVKIMLAGE-----LFHIPTIYQQ------EVEIDLILGNNIL
083 YP_009506270.1   VNEVTSA-GKLWVGKQ-----WFYLPQTYIMP---TLADGVHMIIGMNFI
084 NP_042513.1      HIWYKAVDLVLLLEGK-----RFPLPSVYQQ------DAGLPLILGNNFL
085 YP_006907834.1   TLDKICRNLDIIIAGE-----RFHIDVVFQQ------ESGIDFIIGNNFC
086 YP_004442827.1   VTKQLKE-AKLWVKDT-----FFTLPITYVGNL--DLGRNTQMIIGCNFI
087 YP_009408594.1   ANWKLKG-GQMQIGEN-----KFKIPFTYSFN-MNL-GDGIQMIIGCNFV
```

```
088 YP_009508561.1    WVTWISYLEDPRITFYYDKTLPDLKHV--PASTDNNII-TLLPITEYEAV
089 NP_056907.1       FVKNSPHPSVGILIHHMGRFHNLGSQPSGPWRTLLHLPALLQEPRLLRPL
090 YP_002308474.1    YLHTSDQSSVAILLQHSHRFHNLGAQPSGPWKGLLQVPQIFQNVATLSPP
091 YP_007761644.1    AVEIIQSLSTLRYIDLEFQLEGYSEYRIRSFY----DTGASLMLARYHI
092 NP_040550.1       -LPLEEGILLALRGFAGKIRSSDTPS----------IFDIARPLHVSLK
093 NP_758808.1       IQHRIKR-GTLEIASN-----KYALPLCYIIE-LND-KDDFSMILGCNFF
094 YP_009345075.1    VNEKIKD-GQMRIAGN-----SFRIPFTYCFP-LGA-HDNVDMLIGCNFI
095 NP_068729.1       YIREAISNVFLKIENK-----EFLIPIIYLH-----DSGLDLIIGNNFL
096 NP_047255.1       LLDAERVHFGPTVSLNPATLLPLPSGG-NHHDCLQILAETHGTRPDLTDQ
097 YP_006273075.1    TKKKIKY-GRMMIGMN-----TFRIPFTYSLP--LVIGDNIQMIVGCNFI
098 YP_009508888.1    WITWMTYLEDPRITFHYDKTLPELKDV--PSVYQNDIP-IVPHPSQYSMV
099 NP_395469.1       QINKVALMITILIEKR-----KFLVPTIYQF-----DSGVPMIIGNNFL
100 YP_009508546.1    WITWMTYLEDPRIQFHYDKTLPELKHI--PDVYTSEQP-PSKHPSQYEGV
101 NP_056762.1       NQELIDDIKLQFQEVDETFGIKYKLGQTYVAP----KPTKTFIIGHRFL
102 YP_009508556.1    WITWMTYLEDPRIQFYYDKTLPELQQI--PSVTEDVVA-KTKHPSEFAMV
103 NP_569153.1       VKQKIKT-GKMFINEH-----YFRIPYCYSFE--MQIGDGIQLILGCNFI
104 NP_043924.1       LREAIRLQRPLTLYTDSTYVLGICTKYLAVWKRRGMVNADGSQISNQNIL
105 YP_009243641.1    LLQRSEALQIALAQFVGNIEYTLPKGK---------LWDFFKRHNFVIN
106 YP_004300274.1    VIDRKASNISIQIWDK-----IVKIDHIYQFE----IQGKDIILGMNFI
107 NP_056848.1       VAEYFADNITIMIAKE-----KFIIPYIYAMD----EMSPDIIIGATFY
108 YP_006495799.1    TRKKMRA-GKMVISGN-----DFYTPYISVFD---MDLPDIDMLIGCNFI
109 YP_009508409.1    --------------------------------------------------
110 NP_687035.1       WQDYWQVSWIP-----------------------EWEFVSTPLLVKLW
111 NP_045937.2       LLRPELTFVACSAVSPAHLYMQSCENNIPPHDCVLLTHTISRPRPDLSDL
112 NP_612577.1       QLTKVCSKLPIRLGGE-----RFLIPTLFQQ-----ESGIDLLLGNNFC
113 NP_619548.1       KITKVCKNLKVKFAGK-----SFEIPTVYQQ-----ETGIDFLIGNNFC
114 NP_056880.1       LLQEKEDWPISLLGFLGEVHFHLPKDP---------LLTFTLQTAIIFP
115 YP_009121747.1    SSQKIKA-GNFRIGGN-----KFRIPLIYAMD-MMT-GDGIDMLIGTNFT
116 YP_009113237.2    ASKKIKY-GRMKIGEN-----TFRIPYTYAFQ--MNVGKEIQFIIGCNFI
117 YP_009508408.1    ATKKIKA-GNMKIGDH-----TFRIPYTFAFP--LELGGGEQLILGCNFI
118 NP_955579.1       LLDAERVHFGPTVSLNPATLLPLPSGG-NHHDCLQILAETHGTRPDLTDQ
119 YP_009229919.1    AEYKEKQ-GQMEIGEN-----RFRIPFTYSFP-MGM-KDGIEMLLGCNFI
120 YP_004442833.1    VTKQIKE-AKLWIKET-----FFSLPITYVGNL--DLGTNTQMIIGCNFI
121 NP_862833.2       LFRYSDNWAIAFANYPGRIIHHYPSDK---------LLQFASSHAFIFP
122 NP_040563.1       LQKEESLTQIFP-------------------------VKFYRHSCRW
123 YP_443922.1       IQNKCTQSHITFVNTDKNMADALNADEGSPHVCAERAAQELKLRPDLGND
124 NP_057933.2       LLDTDRVQFGPVVALNPATLLPLPEEG-LQHNCLDILAEAHGTRPDLTDQ
125 YP_009116631.1    TKLKMKN-GKMIVSGS-----DFYTPYIAAFP---MELPDVDMLIGCNFL
126 YP_009513249.1    WLSWISYLEDPRIRFFYDPQMPALKDL--PAVDTGKDN-KKHP-SNFQHI
127 YP_223871.1       LLDPERVRFKQTAALNPATLLPETDDTLPIHHCLDTLDSLTSTRPDLTDQ
128 NP_127504.1       YTSKWDKPISVIAFYDTGAAYSIMDPAILPSEYWIPHFRHFGTADDGILT
129 NP_044929.1       WLKWQTYFEDPRIKFHHDATLPDLQNLPVPQQDTGKEM-TILPLLHYEAI
130 NP_057860.1       FIQTSDHPSVPILLHHSHRFKNLGAQTGELWNTFLKTTAPLAPVKALMPV
131 NP_040973.1       LINS-PYLKAPPP---------------EVEYIHAALNIKRAL
132 NP_777317.1       VTEVIKD-GRLWVGDQ-----YFRLPRTFVMP---KLSDGLHIILGMNFI
133 NP_054716.1       WLKWQTYFEDPRLIFHYDDTLPDLQNL--PQTTLGNEV-DILPLSEYEVV
134 YP_001856242.1    WTEYWQATWIP-----------------------EWEFVNTPPLVKLW
135 NP_954565.2       LMQNTEMWPIACASFVGILDNHYPPNK---------LIQFCKLHTFVFP
136 NP_569150.1       VNEVTSA-GKLWVGKQ-----WFYLPQTFIMP---SLADGVHMIIGMNFI
137 NP_659397.1       TLNKVCRGLKINIAGN-----EFFIPTIYQQ-----ETGIDILLGNNFC
138 YP_009513211.1    LLN-ERVSFAPPAILNPATLLPVESDDTPIHICSEILAEETGTRPDLRDQ
139 YP_233107.1       VNEIIKD-GKLWVGDQ-----YFRIPRTYIMP---TMHKGLEFIIGMNFI
140 YP_009109692.1    LLDTDRVQFGPPVTLNPATLLPVPENQPSPHDCRQVLAETHGTREDLKDQ
141 NP_057849.4       WTEYWQATWIP-----------------------EWEFVNTPPLVKLW
142 NP_049560.1       FIQTSDHPSVPILYHHSHRFKNLGAQTGELWNTFLKTAAPLAPVKALMPM
143 YP_009506267.1    ADKKIKY-GMMKIEGH-----VFRIPYTFAFP--LRLGGGEQLVIGCNFI
144 NP_039820.1       STQMIKA-GRILIGEQ-----YFRMPVTYVMNM--GLSPGIQMIIGCSFI
145 NP_056790.1       LLN-ERVSFAPPAVLNPATLLPVESEATPVHRCSEILAEETGTRRDLEDQ
146 YP_002321513.1    CNQRIAR-GTFKIGDH-----MFQIPLVYAFQ--MNPRDDVKFIIGCNFI
147 NP_955591.1       LLDTDRVQFGPVVALNPATLLPLPEEG-LQHNCLDILAEAHGTRPDLTDQ
148 YP_001956722.2    WITWMSYLEDPRIQFHYDKTLPELQQV--PTVTDDIIA-KIKHPSEFSMV
149 NP_056902.1       MQKGWYYSWLP-----------------------EIVYTHQVVHDDW
150 NP_056886.1       -LPLEEGILLALKGFAGKIRSSDTPS----------IFDIARPLHVSLK
151 NP_041261.1       LLQTSDEWAIALSSFTGDIDNHYPSDP---------VIQFAKLHQFIFP
```

```
001 YP_004442836.1    RTVG--LRIENGEVTIYKIMTTVPAPPIAHDL-----------------
002 NP_056728.1       QLYEPFIQFTDRVIFTK---NKSYPVHIAKLT----------------
003 YP_009508537.1    FYTDGSAITSPTKEGHLNAGMGIVYFMNKDG----------------
004 YP_004732983.2    RAMKGGIRLEGTEVTFYKTITRIQTTLEPQ-----------------
005 YP_009508407.1    --------------------------------------------------
006 NP_040939.1       KRNIIEEVVEGPTYYTDGGK-KNKVGSLG-FI----------------
```

```
007 YP_003864102.1  KIISKSPLDNALLVFTDGSSTG-IAAYTFAD-------------------
008 YP_009002585.1  RNMHGGVRLEGTEVTFYKNITTIQTRLMAPIVE-----------------
009 YP_003284237.1  RSLEGGVRIEGNDITFYKLVTSVRTQREAHQV------------------
010 YP_009268869.1  KRNVITEVVEGPTYYTDGGK-KNGKGSLG-FI------------------
011 YP_009506251.1  RGMYGGLRIEGSTITFYKNVTTIQTKLAAGMVGGTTSFYPGEGGGSHFEF
012 NP_955611.1     VRVTDHPVPGPTVFTDASSSTHKGVVVWREGP------------------
013 YP_008567619.1  RAMHGGVRIEGNEVTFYKYVTKIMAQQGN-AMVRT---------------
014 NP_149413.1     QSLAGGVRLEGRTVTFYKYISSIQAN------------------------
015 YP_004442824.1  HAMEGGVRIEKGEVTFYKIVTTTPTSPIVHDI------------------
016 YP_001931967.1  NLYGPFIQFTNCIILHLRD-NPKTSVRVTKVT------------------
017 YP_002455786.1  FSLSPVVIDQAPCLFSDGSPQKAAYVIWDKVI------------------
018 YP_009109694.1  ELPDADHTWYTDGSSYLDSGTRRAGAAVVD--------------------
019 NP_789739.1     YQLEKEPIVGAETF------------------------------------
020 NP_955619.1     FTLSPVIINTAPCLFSDGSTSQAAYILWDKHI------------------
021 YP_224289.1     RAMSGGVRIEGETVTFYKIVTTTDAPKVPHQI------------------
022 YP_009182100.1  MKYAPYTQYPDRISIAK---NGISYITNVVERPVSVAY------------
023 NP_705927.1     YQLEKEPIVGAETFYVDGAANRETKLGKAGYV------------------
024 YP_009508577.1  FYTDGSAIRSPDKNKSHSSGMGIVHAVFKP--------------------
025 YP_004442830.1  QSLRGGLRLEGRTVTFYKLTTHVQAD------------------------
026 NP_758887.1     YNLLREPVPGEDVYYVDGAANRNSKEGKAGYV------------------
027 YP_009508406.1  --------------------------------------------------
028 YP_610965.1     RSKQGGVRFEGTTVTFYKQLTTVQTEIEAIKS------------------
029 YP_001931961.1  QNYEPLKQYTDRIILTL---ENR-EIIIGKIR------------------
030 YP_241114.1     KIVRRQPIPNATLIFTDGSSNG-TAALIINH-------------------
031 YP_006732334.1  QLYNPFIQWVDRIAFHK---NEE-IILVPKVR------------------
032 YP_009041481.1  KSMGGGLRIEGPTITFYKNVTSIQTQAEELGVISMEKGE-----------
033 YP_001036293.1  RAMNGGVRIEGDTVTFYKNVTTIQTQQQVPKLLAL---------------
034 YP_605811.1     RAMEGGIRIEQGMVTFYKMVTQAQAPPMVHDI------------------
035 NP_955577.1     PLPDADLTWYTDGSSFIRNGEREAGAAVTT--------------------
036 YP_003208050.1  RSMAGGVRLEGTTITFYKQITTINTTLAVESAKKA---------------
037 NP_789740.1     YQLEKEPIVGAETFYVDGAANRETKLGKAGYV------------------
038 YP_004442839.1  RAMEGGIRIEQGIVTFYKMITQAQAPPITHDI------------------
039 YP_009507791.1  TMLTSEPIEGIETWFIDGGR-RLGKKAISAYW------------------
040 YP_004581513.1  RRMKGGLRIEGPTVTFYRNVSTIETQEKSTVAAT----------------
041 NP_043933.1     RLYEPFVQYKDMITFHK----DGRAVSTKKVT------------------
042 YP_009507248.1  EIHTTEIDEIDSVIENIKELSSMINNPSETSR------------------
043 YP_002117531.1  RAMEGGLRIEGDTLSFYKLLTTVQTSRHAQQI------------------
044 YP_004347415.1  NKYSPITIDMEKGIIKFTNKGEVYPNYLVKYPRKKV--------------
045 NP_056891.1     QIISKTPLNNALLVFTDGSSTG-MAAYTLTD-------------------
046 YP_009513242.1  FYTDGSAIKHPDINKSHSAGMGIAQVQFQP--------------------
047 YP_009352866.1  KSMGGGLRIEGTTVTFYKNVTTVQTQAEDLGAPNAGDQD-----------
048 YP_009165750.1  LLYGPFTQYTDRIILFK---ENQ-PVIIGKVS------------------
049 NP_040840.1     KRNVIAEVVPGPTYYTDGGK-KNGRGSLG-YI------------------
050 YP_004222728.1  VRVTDHPVPGPTVFTDASSSTHKGVVVWREGP------------------
051 YP_009506264.1  RSLEGGVRIEGDKITFYKLTTHIDTSKEAFTV------------------
052 YP_567050.1     FTLSPVVLTTAPCLFSDGSSQKAAYVLWDQTI------------------
053 YP_009508571.1  FYTDGSAIRSPDPTKSHNAGMGIVQVKFSP--------------------
054 NP_056803.1     FCTDGSAIKSPDPTKSNNAGMGIVHAIYNP--------------------
055 NP_955564.1     HMTSTTPLEKGIVIFTDGSANGRSVTYIQGR-------------------
056 YP_009508411.1  RAMQGGLRIEGDEVTFYKNVTTIKTQQMVSKVLAL---------------
057 YP_009140788.1  RAMYGGVRIEGNEVTFYKNLTKISTSPEVSVNV-----------------
058 YP_009508410.1  --------------------------------------------------
059 NP_040333.1     PLPDADHTWYTDGSSFLQEGQRKAGAAVTT--------------------
060 YP_008992013.1  TSLITKKPIGIQIFPNCVIWTKVIGSTLPNKDILLG--------------
061 NP_041734.1     RGMYGGLRIEGHTITFYKNVTTIQTRLAAVMVGGTTASELGGGEESKSDS
062 NP_056895.1     -QFSPEPIPAALCLFSDGATGRGAYCLWKDHL------------------
063 YP_009109689.1  ELPDADHTWYTDGSSYLDSGTRRAGAAVVD--------------------
064 YP_002519387.1  KLYRPFTQDLYEIYLTP-------KLLKFKIQQ-----------------
065 YP_595725.1     QSLAGGVRLEGRTVTFYKYIASIKAN------------------------
066 YP_009508551.1  FYTDGSAIKSPDPTKSNNAGMGIVHATYKP--------------------
067 YP_001497148.1  ELPDADHSWYTDGSSYIDSGTRRAGAAVVD--------------------
068 NP_041186.1     KIVRRQPIPEATLIFTDGSSNG-TAALIINH-------------------
069 NP_663784.1     FNLVGDPIPGAETFYTDGSCNRQSKEGKAGYV------------------
070 YP_009508443.1  RSMNGGVRIEGNEVTFYKNVTTIQTQQEVPKVLAL---------------
071 YP_003987465.1  RSLEGGLRIEGEVVTFYKLVTNVQSCRSTHAFG-----------------
072 NP_861410.1     RLYQPFIQGLNYIKLRAP------LDKDINQPS-----------------
073 YP_009345071.1  RSLHGGVRIEGNEITFYKYLTKLESINLIEEDN-----------------
074 YP_009508582.1  FYTDGSAIRSPDKNKSHSSGMGIVQAIFKP--------------------
075 NP_569141.1     EKLYPHIIT--KTHWWFTTPCKNKVGAKRVNNKQRK--------------
076 YP_002916057.1  RRMKGGLRIEGPTVTFYRNVSTIETQEKSTVAAX----------------
077 NP_777384.2     -QFSPEPIPAALCLFSDGATGRGAYCLWKDHL------------------
078 YP_233110.1     RAMEGGIRIEQGTVTFYKMVTQAQAPPMVHDI------------------
079 NP_542258.1     FIISPVVIDHAPCLFSDGATSKAAFILWDKQV------------------
```

```
080 YP_009130664.1   RSMQGGVRIEGDTVTFYKNVTQIQTQQTVPIIAAI---------------
081 YP_009508566.1   FYTDGSSIKSPDPTKTHSSGMGIVQAIYEP--------------------
082 YP_006607892.1   QLHEPFVQFTDRIIIHK---DGE-PVTIGKVF------------------
083 YP_009506270.1   RTVG--LRIENGEVTIYKIMTTVQAPPIVHEL------------------
084 NP_042513.1      KLYNPFIQTLETISLRCP------QLE--KQPS-----------------
085 YP_006907834.1   QEYSPFIQFTGHIVFTM---KDQYQVPITKLR------------------
086 YP_004442827.1   QSLRGAVRLEGRSVTFYKLISTVEAD------------------------
087 YP_009408594.1   RAMQGGIRIEGNQVTFYKQLTTIETTMSAGPCIQE---------------
088 YP_009508561.1   FYTDGSAIKSPKTEQTHSAGMGIVMVVYTP--------------------
089 NP_056907.1      FTLSPVVLTTAPCLFSDGSSQKAAYVLWDQTI------------------
090 YP_002308474.1   FTISPVVINHAPCLFSDGSNSQAAFTIWDKKI------------------
091 YP_007761644.1   FPPELWIKQTPKLVSYANQSTGSIMYKAVQVPFKLG--------------
092 NP_040550.1      VRVTDHPVPGPTAFTDASSSTHKGVVVWREGP------------------
093 NP_758808.1      KHMGGGMRFEGPHVTFYKGITTLST--------S----------------
094 YP_009345075.1   KANYGGMRIEGSKITFYKNVGSIETSPVVSSAHFA---------------
095 NP_068729.1      KLYQPFIQRLETIELRWK------NLNNPKES------------------
096 NP_047255.1      PLPDADLTWYTDGSSFIRNGEREAGAAVTT--------------------
097 YP_006273075.1   RAMYGGVRIEGNEVTFYKNLTRISTSPEVSVNS-----------------
098 YP_009508888.1   FYTDGSAIKNPNPTKTHSAGMGVVQGKFNP--------------------
099 NP_395469.1      RLYYPFCQYLSYITLRCP------KMINQKQ-------------------
100 YP_009508546.1   FYTDGSAIKSPDPTKSNNAGMGIVHVTYKP--------------------
101 NP_056762.1      TNENGSVTIHKDYITIQKTTGIYPTARHELKSEFAR--------------
102 YP_009508556.1   FYTDGSAIKHPDINKSHSAGMGIAQVQFQP--------------------
103 NP_569153.1      RSMYGGVRLEGNTITFYKQITSINTRLAAPLLK-----------------
104 NP_043924.1      QEIWQLIEHDSTQTLGIVKVKAHTQRKCSTHEQQLNNDVDQPAKQYAKEE
105 YP_009243641.1   QIISDIPLE-GPNVFTDGSRQR--SAYWAEK-------------------
106 YP_004300274.1   RMYLPHKIS--KDFWYLTTPCGKMIGAKIVENKERF--------------
107 NP_056848.1      NKYSPIELDIGKGIIKFTKNNEKYPNYLVKYPKKRK--------------
108 YP_006495799.1   KAMQGGIRFEGTEVTIYKKVTTIQTTLEPI--------------------
109 YP_009508409.1   --------------------------------------------------
110 NP_687035.1      YSLVKEPIKGEDVYYVDGAASKVTKLGKAGYL------------------
111 NP_045937.2      PIPDPDMTLFSDGSYTTGRGGAAVVMHRPVTD------------------
112 NP_612577.1      QLYSPFIQYTDRIYFHL---NKQ-SVIIGKIT------------------
113 NP_619548.1      RLYNPFIQWEDRIAFHL----KNEMVLIKKVT------------------
114 NP_056880.1      HMTSTTPLEKGIVIFTDGSANGRSVTYIQGR-------------------
115 YP_009121747.1   RSMNGGIRIEGDEVTLYKKVTKIKTNPTVEITA-A---------------
116 YP_009113237.2   RAMNGGLRIEGPTLTFYKLLTSIETKTVAALIK-----------------
117 YP_009508408.1   RSMSGGVRIEGDNVTFYKNVTTIKTQQEVSKVLSL---------------
118 NP_955579.1      PLPDADLTWYTDGSSFIRNGEREAGAAVTT--------------------
119 YP_009229919.1   RSMYGGLRIEGNLLTFYKNVTTVTTVQNTEASKLA---------------
120 YP_004442833.1   QSLKGAVRLEGRSVTFYKLVSTIEAD------------------------
121 NP_862833.2      KIVRQQPIPEATLIFTDGSSNG-TAALIINQ-------------------
122 NP_040563.1      TSICGPVRENLTTYYTDGGK--KGKTAAAVYW------------------
123 YP_443922.1      PLPQADLWLYTDGCCYRGKDGNIAAYAVVQQDSNG---------------
124 NP_057933.2      PLPDADHTWYTDGSSLLQEGQRKAGAAVTT--------------------
125 YP_009116631.1   RAMKGGVRLEGTEVTIYKKVTTIQTTLEPQ--------------------
126 YP_009513249.1   FYTDGSAITSPTKEGHLNAGMGIVYFINKDG-------------------
127 YP_223871.1      PLAQAEATLFTDGSSYVRDGKRYAGAAVVT--------------------
128 NP_127504.1      TTVKTKHPITIEFFPGFKYTTKLLGSDIPGKDLLIG--------------
129 NP_044929.1      FYTDGSAIRSPKPNKTHSAGMGIIQAKFEP--------------------
130 NP_057860.1      FTLSPVIINTAPCLFSDGSTSQAAYILWDKHI------------------
131 NP_040973.1      SMIKDAPIPGAETWYIDGGR-KLGKAAKAAYW------------------
132 NP_777317.1      SSMEGGIRIEQGMVTFYKLVTQAETSPIVHEL------------------
133 NP_054716.1      FYTDGSSIKSPKKDKQHSAGMGIIAVRYQP--------------------
134 YP_001856242.1   YQLEKEPIVGAETFYVDGAANRETKLGKAGYV------------------
135 NP_954565.2      QIISKTPLNNALLVFTDGSSTG-MAAYTLTD-------------------
136 NP_569150.1      RTVG--LRIENGEVTIYKIMTTVQAPPIVHEL------------------
137 NP_659397.1      QTYQPFIQWVDHIAFHIKENNEEKRVLIPKVR------------------
138 YP_009513211.1   PLPGVP-AWYTDGSSFIMDGRRQAGAAIVD--------------------
139 YP_233107.1      KAMEGGLRIEKGEVTFYKLVTTVNTSPKPHEV------------------
140 YP_009109692.1   ELPDADHTWYTDGSSYLDSGTRRAGAAVVD--------------------
141 NP_057849.4      YQLEKEPIVGAETFYVDGAANRETKLGKAGYV------------------
142 NP_049560.1      FTLSPVIINAAPCLFSDGSTSRAAYILWDKHI------------------
143 YP_009506267.1   RSMNGGVRIEGNDVTFYKNITTVKTQTEVPKILAL---------------
144 NP_039820.1      RSLEGGLRIEKDIITFYKLVTSIETSRTTQVA------------------
145 NP_056790.1      PLPGVP-TWYTDGSSFITEGKRRAGAPIVD--------------------
146 YP_002321513.1   RSMKGGIKIEGNTITFYERSYSIQTCEESGEIAAK---------------
147 NP_955591.1      PLPDADHTWYTDGSSLLQEGQRKAGAAVTT--------------------
148 YP_001956722.2   FYTDGSAIKHPNVNKSHNAGMGIAQVQFKP--------------------
149 NP_056902.1      RMKLVEEPTSGITIYTDGGK-QNGE-GIAAYV------------------
150 NP_056886.1      VRVTDHPVPGPTVFTDASSSTHKGVVVWREGP------------------
151 NP_041261.1      KITKCAPIPQATLVFTDGSSNG-IAAYVIDN-------------------
```

001	YP_004442836.1	--NYIEELELELYEYYDICATQ----------------------------
002	NP_056728.1	---RAVRVG--TEGFLESMKKRS---------------------------
003	YP_009508537.1	--
004	YP_004732983.2	-----KIAYLEELVEA-EDLHYE---------------------------
005	YP_009508407.1	--
006	NP_040939.1	--
007	YP_003864102.1	--
008	YP_009002585.1	EDDEDDEVEQ---ETNWIRTMAS---------------------------
009	YP_003284237.1	--AAIEELDLNEDEYYDIAISD----------------------------
010	YP_009268869.1	--
011	YP_009506251.1	KASEESDDDAEIDFEIHQQIISH---------------------------
012	NP_955611.1	--
013	YP_008567619.1	-----EVISEEEEEYSAVEEICT---------------------------
014	NP_149413.1	--------EFLQAEVEEINIAT----------------------------
015	YP_004442824.1	--NYIEELELEIPEYYDICSTVG---------------------------
016	YP_001931967.1	---KAYAYG--KPGFIQSMKKGS---------------------------
017	YP_002455786.1	--
018	YP_009109694.1	--
019	NP_789739.1	--
020	NP_955619.1	--
021	YP_224289.1	--NLIEELEMSEEEFIEVQIST----------------------------
022	YP_009182100.1	--RKEFLERRKIPDHQQSHKVKCGENYKVQKQAQENDFTIEDYGTTDSEA
023	NP_705927.1	--
024	YP_009508577.1	--
025	YP_004442830.1	--------EYLKMAEEQLMIAQ----------------------------
026	NP_758887.1	--
027	YP_009508406.1	--
028	YP_610965.1	-----AQFDEPEELMEIICPQWH---------------------------
029	YP_001931961.1	---RAHRVG--VPGFLESLKKKS---------------------------
030	YP_241114.1	--
031	YP_006732334.1	---KAMRKG--YEGFLKTMERDS---------------------------
032	YP_009041481.1	GAHQQQIAELTTTESVLDVQEAY---------------------------
033	YP_001036293.1	-----EELEMDEDEYIELQQSIS---------------------------
034	YP_605811.1	--SYLEELELELPIYYDICATN----------------------------
035	NP_955577.1	--
036	YP_003208050.1	----IPELDLDAEMLAEIQELVY---------------------------
037	NP_789740.1	--
038	YP_004442839.1	--SYIEELELELPLYYDICAAN----------------------------
039	YP_009507791.1	--
040	YP_004581513.1	----------------------I---------------------------
041	NP_043933.1	---KAYFHG--LPGFLESKKVGS---------------------------
042	YP_009507248.1	--
043	YP_002117531.1	--AAIQELELDEIEYQNLGALD----------------------------
044	YP_004347415.1	--LIPWEKGN--
045	NP_056891.1	--
046	YP_009513242.1	--
047	YP_009352866.1	GLPQQQVAELLTAEAVLDHQEAY---------------------------
048	YP_009165750.1	---KAYLHG--MPGYLDSMKKNS---------------------------
049	NP_040840.1	--
050	YP_004222728.1	--
051	YP_009506264.1	--ASIEELDLNEDEYYDIALMD----------------------------
052	YP_567050.1	--
053	YP_009508571.1	--
054	NP_056803.1	--
055	NP_955564.1	--
056	YP_009508411.1	-----EELEMTEDEFIEVNETLS---------------------------
057	YP_009140788.1	-----LEEEVCEEEYLQIQDMVV---------------------------
058	YP_009508410.1	--
059	NP_040333.1	--
060	YP_008992013.1	-FDILHQIK-HLQIIPHGIRVKS---------------------------
061	NP_041734.1	ESMFDLSETEEFDSETHQQIVSH---------------------------
062	NP_056895.1	--
063	YP_009109689.1	--
064	YP_002519387.1	---RAFKQA--SPGFLDSIRKQKRGE------------------------
065	YP_595725.1	--------EYLQAEAEEILVAT----------------------------
066	YP_009508551.1	--
067	YP_001497148.1	--
068	NP_041186.1	--
069	NP_663784.1	--
070	YP_009508443.1	-----EELEMGEEEYIAIQESCS---------------------------

071 YP_003987465.1 --AIIEELEMAEDEYIDLNLML----------------------------
072 NP_861410.1 ---KMIYIPVTTPSKILQFAILE---------------------------
073 YP_009345071.1 -------FSKNFSEYMLLNDTGT---------------------------
074 YP_009508582.1 --
075 NP_569141.1 --TTEWIRGS--
076 YP_002916057.1 ----------------------I---------------------------
077 NP_777384.2 --
078 YP_233110.1 --SYLEELELELPIYYDICATN----------------------------
079 NP_542258.1 --
080 YP_009130664.1 -----EELELEEEEYISIATICA---------------------------
081 YP_009508566.1 --
082 YP_006607892.1 ---KAYYHG--KPGYLESMKKNS---------------------------
083 YP_009506270.1 --NYIEELELELQEYYNICSAE----------------------------
084 NP_042513.1 ---SLITTKIYNTFSLFGGVIVN---------------------------
085 YP_006907834.1 ---RAFKRG--IPGFLESMKKRS---------------------------
086 YP_004442827.1 --------EYIQKIEEQVLIVQ----------------------------
087 YP_009408594.1 ----EEDLD-----AAVMTELSL---------------------------
088 YP_009508561.1 --
089 NP_056907.1 --
090 YP_002308474.1 --
091 YP_007761644.1 --YKTYSFDFWCHDAMSHDIIVG---------------------------
092 NP_040550.1 --
093 NP_758808.1 ----YANTGIDTEHEQITSTT-----------------------------
094 YP_009345075.1 ----IEELEMDELDYLKIHESVF---------------------------
095 NP_068729.1 ---QMISTKILTKNEVLKLSFEK---------------------------
096 NP_047255.1 --
097 YP_006273075.1 -----LDEEVCEEEYLQVQEMVI---------------------------
098 YP_009508888.1 --
099 NP_395469.1 ---EVIKIPIHHSSQLIKAKLLN---------------------------
100 YP_009508546.1 --
101 NP_056762.1 ------KHGGRPPLFSNIPETYN---------------------------
102 YP_009508556.1 --
103 NP_569153.1 QEEEEKEEELNLEEHRLIQEMVA---------------------------
104 NP_043924.1 PNMSVIAPLQVYPLWIGLVPCKE---------------------------
105 YP_009243641.1 --
106 YP_004300274.1 --KCEWEKGD--
107 NP_056848.1 --LVPWTKGN--
108 YP_006495799.1 -----KLGYAEFDPDIQVELERA---------------------------
109 YP_009508409.1 --
110 NP_687035.1 --
111 NP_045937.2 --
112 NP_612577.1 ---KAYQYG--VKGFLESMKKKS---------------------------
113 NP_619548.1 ---KAFSVS--NPSFLENMKKDS---------------------------
114 NP_056880.1 --
115 YP_009121747.1 ----IQELEIDEEFYQELQGTXM---------------------------
116 YP_009113237.2 ------EEEVNYEELDQIKEIVA---------------------------
117 YP_009508408.1 -----EELEMNEDEYISIQQSVS---------------------------
118 NP_955579.1 --
119 YP_009229919.1 ----VEELGLEAEEYYSIREMVY---------------------------
120 YP_004442833.1 --------EYIRRAEEQICIAH----------------------------
121 NP_862833.2 --
122 NP_040563.1 --
123 YP_443922.1 --
124 NP_057933.2 --
125 YP_009116631.1 -----KISLLRAEAEVGEELERM---------------------------
126 YP_009513249.1 --
127 YP_223871.1 --
128 NP_127504.1 -FDIYRQLNNKLRIGADGIRWKN---------------------------
129 NP_044929.1 --
130 NP_057860.1 --
131 NP_040973.1 --
132 NP_777317.1 --NYIEELELELPEYYNICAAA----------------------------
133 NP_054716.1 --
134 YP_001856242.1 --
135 NP_954565.2 --
136 NP_569150.1 --NYIDELELELHEYYNICAAE----------------------------
137 NP_659397.1 ---IAMRKG--HPGFLEAMRKGS---------------------------
138 YP_009513211.1 --
139 YP_233107.1 --CLLDELDLELPEYYDICAAI----------------------------
140 YP_009109692.1 --
141 NP_057849.4 --
142 NP_049560.1 --
143 YP_009506267.1 -----EELEMEEEEYLALQSKYS---------------------------

```
144 NP_039820.1         --NSIEELELSEDEYLNIAASV----------------------------
145 NP_056790.1         --------------------------------------------------
146 YP_002321513.1      --YAIPELELNENEYQCLSESIY---------------------------
147 NP_955591.1         --------------------------------------------------
148 YP_001956722.2      --------------------------------------------------
149 NP_056902.1         --------------------------------------------------
150 NP_056886.1         --------------------------------------------------
151 NP_041261.1         --------------------------------------------------


001 YP_004442836.1      --------------------------------------------------
002 NP_056728.1         --------------------------------------------------
003 YP_009508537.1      --------------------------------------------------
004 YP_004732983.2      --------------------------------------------------
005 YP_009508407.1      --------------------------------------------------
006 NP_040939.1         --------------------------------------------------
007 YP_003864102.1      --------------------------------------------------
008 YP_009002585.1      --------------------------------------------------
009 YP_003284237.1      --------------------------------------------------
010 YP_009268869.1      --------------------------------------------------
011 YP_009506251.1      --------------------------------------------------
012 NP_955611.1         --------------------------------------------------
013 YP_008567619.1      --------------------------------------------------
014 NP_149413.1         --------------------------------------------------
015 YP_004442824.1      --------------------------------------------------
016 YP_001931967.1      --------------------------------------------------
017 YP_002455786.1      --------------------------------------------------
018 YP_009109694.1      --------------------------------------------------
019 NP_789739.1         --------------------------------------------------
020 NP_955619.1         --------------------------------------------------
021 YP_224289.1         --------------------------------------------------
022 YP_009182100.1      EEPIYDAIYDKGFQPIESDDEPNDEIQPINDEPNEEVRPGFQPIESNDEP
023 NP_705927.1         --------------------------------------------------
024 YP_009508577.1      --------------------------------------------------
025 YP_004442830.1      --------------------------------------------------
026 NP_758887.1         --------------------------------------------------
027 YP_009508406.1      --------------------------------------------------
028 YP_610965.1         --------------------------------------------------
029 YP_001931961.1      --------------------------------------------------
030 YP_241114.1         --------------------------------------------------
031 YP_006732334.1      --------------------------------------------------
032 YP_009041481.1      --------------------------------------------------
033 YP_001036293.1      --------------------------------------------------
034 YP_605811.1         --------------------------------------------------
035 NP_955577.1         --------------------------------------------------
036 YP_003208050.1      --------------------------------------------------
037 NP_789740.1         --------------------------------------------------
038 YP_004442839.1      --------------------------------------------------
039 YP_009507791.1      --------------------------------------------------
040 YP_004581513.1      --------------------------------------------------
041 NP_043933.1         --------------------------------------------------
042 YP_009507248.1      --------------------------------------------------
043 YP_002117531.1      --------------------------------------------------
044 YP_004347415.1      --------------------------------------------------
045 NP_056891.1         --------------------------------------------------
046 YP_009513242.1      --------------------------------------------------
047 YP_009352866.1      --------------------------------------------------
048 YP_009165750.1      --------------------------------------------------
049 NP_040840.1         --------------------------------------------------
050 YP_004222728.1      --------------------------------------------------
051 YP_009506264.1      --------------------------------------------------
052 YP_567050.1         --------------------------------------------------
053 YP_009508571.1      --------------------------------------------------
054 NP_056803.1         --------------------------------------------------
055 NP_955564.1         --------------------------------------------------
056 YP_009508411.1      --------------------------------------------------
057 YP_009140788.1      --------------------------------------------------
058 YP_009508410.1      --------------------------------------------------
059 NP_040333.1         --------------------------------------------------
060 YP_008992013.1      --------------------------------------------------
061 NP_041734.1         --------------------------------------------------
062 NP_056895.1         --------------------------------------------------
```

063 YP_009109689.1 --
064 YP_002519387.1 --
065 YP_595725.1 --
066 YP_009508551.1 --
067 YP_001497148.1 --
068 NP_041186.1 --
069 NP_663784.1 --
070 YP_009508443.1 --
071 YP_003987465.1 --
072 NP_861410.1 --
073 YP_009345071.1 --
074 YP_009508582.1 --
075 NP_569141.1 --
076 YP_002916057.1 --
077 NP_777384.2 --
078 YP_233110.1 --
079 NP_542258.1 --
080 YP_009130664.1 --
081 YP_009508566.1 --
082 YP_006607892.1 --
083 YP_009506270.1 --
084 NP_042513.1 --
085 YP_006907834.1 --
086 YP_004442827.1 --
087 YP_009408594.1 --
088 YP_009508561.1 --
089 NP_056907.1 --
090 YP_002308474.1 --
091 YP_007761644.1 --
092 NP_040550.1 --
093 NP_758808.1 --
094 YP_009345075.1 --
095 NP_068729.1 --
096 NP_047255.1 --
097 YP_006273075.1 --
098 YP_009508888.1 --
099 NP_395469.1 --
100 YP_009508546.1 --
101 NP_056762.1 --
102 YP_009508556.1 --
103 NP_569153.1 --
104 NP_043924.1 --
105 YP_009243641.1 --
106 YP_004300274.1 --
107 NP_056848.1 --
108 YP_006495799.1 --
109 YP_009508409.1 --
110 NP_687035.1 --
111 NP_045937.2 --
112 NP_612577.1 --
113 NP_619548.1 --
114 NP_056880.1 --
115 YP_009121747.1 --
116 YP_009113237.2 --
117 YP_009508408.1 --
118 NP_955579.1 --
119 YP_009229919.1 --
120 YP_004442833.1 --
121 NP_862833.2 --
122 NP_040563.1 --
123 YP_443922.1 --
124 NP_057933.2 --
125 YP_009116631.1 --
126 YP_009513249.1 --
127 YP_223871.1 --
128 NP_127504.1 --
129 NP_044929.1 --
130 NP_057860.1 --
131 NP_040973.1 --
132 NP_777317.1 --
133 NP_054716.1 --
134 YP_001856242.1 --
135 NP_954565.2 --

```
136 NP_569150.1          --------------------------------------------------
137 NP_659397.1          --------------------------------------------------
138 YP_009513211.1       --------------------------------------------------
139 YP_233107.1          --------------------------------------------------
140 YP_009109692.1       --------------------------------------------------
141 NP_057849.4          --------------------------------------------------
142 NP_049560.1          --------------------------------------------------
143 YP_009506267.1       --------------------------------------------------
144 NP_039820.1          --------------------------------------------------
145 NP_056790.1          --------------------------------------------------
146 YP_002321513.1       --------------------------------------------------
147 NP_955591.1          --------------------------------------------------
148 YP_001956722.2       --------------------------------------------------
149 NP_056902.1          --------------------------------------------------
150 NP_056886.1          --------------------------------------------------
151 NP_041261.1          --------------------------------------------------


001 YP_004442836.1       --------------------NQRGEIAEEFIEP-----------------
002 NP_056728.1          ------KTQQPE-------PVNIS--TNKIENPL----EEIAILSEGRRL
003 YP_009508537.1       --------------------NLQKQQEWSISLGN----------------
004 YP_004732983.2       --------------------LAAASMPEPTAEGLR---------------
005 YP_009508407.1       --------------------------------------------------
006 NP_040939.1          ---------------------VSTGEKFRKHEE-----------------
007 YP_003864102.1       -----------------------SIIKFQTK--F----------------
008 YP_009002585.1       --------------------STMDAPYEKLRSS-----------------
009 YP_003284237.1       --------------------QEKAYINKEIVDS-----------------
010 YP_009268869.1       ---------------------ASTGVKFRKHEE-----------------
011 YP_009506251.1       --------------------AVAQATTPKLSPK-----------------
012 NP_955611.1          ---------------------RWEIKEIADL--G----------------
013 YP_008567619.1       ----------------------GTVIHPEFGNK-----------------
014 NP_149413.1          --------------------SEGGFIDKPFMSKN----------------
015 YP_004442824.1       --------------------AIQGGISEEFISP-----------------
016 YP_001931967.1       ------KVKPPT-------PENISRLTDKIVSFSGGESSEDTTKTNFEND
017 YP_002455786.1       ---------------------LSQRSVPLPPHAN----------------
018 YP_009109694.1       --------------------GHNTIWAQSLPPG-----------------
019 NP_789739.1          --------------------------------------------------
020 NP_955619.1          ---------------------LSQRSFPLPP-PH----------------
021 YP_224289.1          --------------------STEGYINKEIMDS-----------------
022 YP_009182100.1       NNEIQPSNDEPNKEVRPKPTKDIR--PKPITEEDFQAELRLVEAHYEKQA
023 NP_705927.1          ---------------------TNRGRQKVVTLTD----------------
024 YP_009508577.1       --------------------ELTIEHQWSIPLGD----------------
025 YP_004442830.1       --------------------QPSPFIEKSFMTRN----------------
026 NP_758887.1          ---------------------TARNKSRVIALEN----------------
027 YP_009508406.1       --------------------------------------------------
028 YP_610965.1          --------------------IEHPTDAIGVGLR-----------------
029 YP_001931961.1       ------KLTMQK-------GTNIA--PSKT--------SFDKRGELGYTL
030 YP_241114.1          -----------------------QTYYTQTS--F----------------
031 YP_006732334.1       ------KSQPTP-------GTNIT--QEVIDE------------------
032 YP_009041481.1       --------------------FARPEQPVDLKFKHV---------------
033 YP_001036293.1       --------------------SIEP--NNYFSNK-----------------
034 YP_605811.1          --------------------PSGGEINSDLIPP-----------------
035 NP_955577.1          --------------------ESEVIWAAPLPPG-----------------
036 YP_003208050.1       --------------------YQSPARNPRFESR-----------------
037 NP_789740.1          ---------------------TNRGRQKVVTLTD----------------
038 YP_004442839.1       --------------------PIGGMFNNDLISP-----------------
039 YP_009507791.1       ---------------------TSEGKEKYEYIE-----------------
040 YP_004581513.1       --------------------GSINEGRTLIFPR-----------------
041 NP_043933.1          ------STSTPN-------PENIT--PVTINQDNISKIFKGEEIGESEQ-
042 YP_009507248.1       --------------------PQRQTENTDEIRQ-----------------
043 YP_002117531.1       ---------------------DQAFFNKKFMEQN----------------
044 YP_004347415.1       --------------------PSIIQPLQNEQIN-----------------
045 NP_056891.1          -----------------------TTIKFQTN--L----------------
046 YP_009513242.1       --------------------EYKVIHQWSIPLGD----------------
047 YP_009352866.1       --------------------FAQPEKPIDQKFKHV---------------
048 YP_009165750.1       ------RNPVPP-------PINIT--SNKIEDSKRFSVLKLQLLERGREI
049 NP_040840.1          ---------------------TSTGEKFRIHEE-----------------
050 YP_004222728.1       ---------------------RWEIKEIADL--G----------------
051 YP_009506264.1       --------------------EEKGYINREIVDS-----------------
052 YP_567050.1          ---------------------LHHDSVTLPPHGS----------------
053 YP_009508571.1       --------------------ELQVINQWSIPLGN----------------
054 NP_056803.1          --------------------EYKILNQWSIPLGH----------------
```

```
055 NP_955564.1      -----------------------EPIIKENT--Q----------------
056 YP_009508411.1   --------------------VVGR----QPQPK-----------------
057 YP_009140788.1   --------------------CNIGESRESFLKK-----------------
058 YP_009508410.1   --------------------------------------------------
059 NP_040333.1      --------------------ETEVVWAKALPAG-----------------
060 YP_008992013.1   ------------------MFKPFTDVLKLYNLSETP--------------
061 NP_041734.1      --------------------VAAQAQQQKLDPK-----------------
062 NP_056895.1      ---------------------LDFQAVPAP--------------------
063 YP_009109689.1   --------------------GHNTIWAQSLPPG-----------------
064 YP_002519387.1   ----IISKHATN-------PINITKLEDNLRIVETILEDSNSKTLSEVSD
065 YP_595725.1      --------------------SEQEFINRSFMSKN----------------
066 YP_009508551.1   --------------------EYQVLNQWSIPLGN----------------
067 YP_001497148.1   --------------------GHHIIWAQSLPPG-----------------
068 NP_041186.1      -----------------------QTYYAQTS--F----------------
069 NP_663784.1      ---------------------TDRGKDKVKVLEQ----------------
070 YP_009508443.1   --------------------TICPVVNINFAQR-----------------
071 YP_003987465.1   --------------------ATEPAVGEEFAKL-----------------
072 NP_861410.1      ------------------KLQDILFELHVQEN------------------
073 YP_009345071.1   --------------------DELQNEQENLRIKKA---------------
074 YP_009508582.1   --------------------ELTIEHQWTIPLGD----------------
075 NP_569141.1      --------------------EKITQKLENINKNTTT--------------
076 YP_002916057.1   --------------------GNINEERTMVFPR-----------------
077 NP_777384.2      ---------------------LDFQAVPAP--------------------
078 YP_233110.1      --------------------PSGGEINSDLISP-----------------
079 NP_542258.1      ---------------------IHQQVLPLP--ST----------------
080 YP_009130664.1   --------------------YGGMEISSPFQFS-----------------
081 YP_009508566.1   --------------------NFQIKHQWSIPLGD----------------
082 YP_006607892.1   ------KKPVPS-------PLNIT--PVKIEEQE---TLRLQLLRRGREI
083 YP_009506270.1   --------------------TSKGEIAEEFISP-----------------
084 NP_042513.1      ------------------ILKQQIYIAIEDE-------------------
085 YP_006907834.1   ------TAQQPE-------PLNIS--TNKTVS-----------LSRGRRF
086 YP_004442827.1   --------------------EPAPYIDRPFMTKN----------------
087 YP_009408594.1   --------------------FSAVPSNQKFLQQ-----------------
088 YP_009508561.1   --------------------EPNITQQWSIPLGD----------------
089 NP_056907.1      ---------------------LHHDSVTLPPHGS----------------
090 YP_002308474.1   ---------------------IHQQVLPLP--TA----------------
091 YP_007761644.1   ----NPFWIYLQQSFMSITGNTVHTKDNSFVFYEHKNPARETPWQSEFTH
092 NP_040550.1      ---------------------RWEIKEIADL--G----------------
093 NP_758808.1      --------------------------SQSFKER-----------------
094 YP_009345075.1   --------------------YNVGQKLGNHGPE-----------------
095 NP_068729.1      ------------------IHICLEKYLFFKT-------------------
096 NP_047255.1      --------------------ESEVIWAAPLPPG-----------------
097 YP_006273075.1   --------------------CNIGESRKSFLKK-----------------
098 YP_009508888.1   --------------------EFQVVNQWSIPLGN----------------
099 NP_395469.1      ------------------LVTNIEEQLLMEQ-------------------
100 YP_009508546.1   --------------------EYQILNQWSIPLGN----------------
101 NP_056762.1      ------------------KIPHLHSYQPQPILGYKN--------------
102 YP_009508556.1   --------------------EYKVIHQWSIPLGD----------------
103 NP_569153.1      --------------------YSTERPFVQFQQK-----------------
104 NP_043924.1      -------------------PKLWENIQHHITKVDLP--------------
105 YP_009243641.1   -----------------------KYWSQKQN--T----------------
106 YP_004300274.1   --------------------KTLNQKLRNVYD------------------
107 NP_056848.1      --------------------PSVTETMENIGIN-----------------
108 YP_006495799.1   --------------------YYAAPVSEAELIKLR---------------
109 YP_009508409.1   --------------------------------------------------
110 NP_687035.1      ---------------------SERGKSRIRELEN----------------
111 NP_045937.2      -----------------------DFIIIHQQPGG----------------
112 NP_612577.1      ------KVNRPE-------PINIT--SNQH--------LFLEEG--GNHV
113 NP_619548.1      ------KTEQIP-------GTNIS--KNIINP------------------
114 NP_056880.1      -----------------------EPIIKENT--Q----------------
115 YP_009121747.1   --------------------FSKE-DAESLKER-----------------
116 YP_009113237.2   --------------------MNVHEPYPGFAQR-----------------
117 YP_009508408.1   --------------------AISPGIHSSFTTR-----------------
118 NP_955579.1      --------------------ESEVIWAAPLPPG-----------------
119 YP_009229919.1   --------------------YSLGHTSPKFQER-----------------
120 YP_004442833.1   --------------------DPASFVQETFMKKN----------------
121 NP_862833.2      -----------------------QTYYAHTN--F----------------
122 NP_040563.1      ---------------------CEG--RTKSKVFP----------------
123 YP_443922.1      --------------------NHSTLESGVIPQP-----------------
124 NP_057933.2      --------------------ETEVIWAKALPAG-----------------
125 YP_009116631.1   --------------------YYANDYSEEGISRLK---------------
126 YP_009513249.1   --------------------NLQKQQEWSISLGN----------------
127 YP_223871.1      --------------------LDSVIWAEPLPIG-----------------
```

```
128 NP_127504.1      ------------------QFKRYTEIPRLFQLTTSN--------------
129 NP_044929.1      --------------------DFRIVHLWSFPLGD----------------
130 NP_057860.1      ---------------------LSQRSFPLPP-PH----------------
131 NP_040973.1      ---------------------TDTGKWQVMELE-----------------
132 NP_777317.1      --------------------PESGVISEDFLDK-----------------
133 NP_054716.1      --------------------QMNIIQEWSIPLGD----------------
134 YP_001856242.1   ---------------------TNRGRQKVVTLTD----------------
135 NP_954565.2      -----------------------TTIKFQTN--L----------------
136 NP_569150.1      --------------------SSRGEISEEFISP-----------------
137 NP_659397.1      ------GKKPVP-------GTNIT--QEVIDD------------------
138 YP_009513211.1   --------------------NKRTVWASNLPEG-----------------
139 YP_233107.1      --------------------PRQGSINEEFISP-----------------
140 YP_009109692.1   --------------------GHNTIWAQSLPPG-----------------
141 NP_057849.4      ---------------------TNRGRQKVVTLTD----------------
142 NP_049560.1      ---------------------LSQKSFPLPP-PH----------------
143 YP_009506267.1   --------------------SICPVIQENFKAR-----------------
144 NP_039820.1      --------------------ETPSFLDQEFARKN----------------
145 NP_056790.1      --------------------GKRTVWASSLPEG-----------------
146 YP_002321513.1   --------------------LNIGTTSDGFKSR-----------------
147 NP_955591.1      --------------------ETEVIWAKALPAG-----------------
148 YP_001956722.2   --------------------EFTVINTWSIPLGD----------------
149 NP_056902.1      ---------------------TSNGRTKQKRLGP----------------
150 NP_056886.1      ---------------------RWEIKEIADL--G----------------
151 NP_041261.1      -----------------------QPISIKSP--Y----------------
```

```
001 YP_004442836.1   -------------------AMMTRMKKLGYIGEEPLKHWTKNQV------
002 NP_056728.1      SE-------EKLFITQQR-MQKIEELLEKVCSENPLDPNKTKQW------
003 YP_009508537.1   --------------------HTAQFAEIAAFEFALKKCLPLGGN------
004 YP_004732983.2   -----------------NTKLLAELKDQGYIGEEPLKHWSKNRV------
005 YP_009508407.1   ------------------------------MN-HTEEYKRALQA------
006 NP_040939.1      --------------------GTNQQLELRAIEEALKQGPQT---------
007 YP_003864102.1   --------------------SSAQLVELQAFIAVLSAFPNQP--------
008 YP_009002585.1   -----------------FGPLMKELKDQGYIGENPMKHWARNKV------
009 YP_003284237.1   -------------------SIFRRLKELGYIGEEPLKHWRKNQV------
010 YP_009268869.1   --------------------GTNQQLELRAIEEACKQGPEK---------
011 YP_009506251.1   -----------------LKHLMGQLKDQGFIGENPMKHWASNKI------
012 NP_955611.1      --------------------ASVQQLEARAVAMALLLWPTTP--------
013 YP_008567619.1   -----------------YQKLIEDMKNQGYIGEDPMKYWQKNQV------
014 NP_149413.1      ------------------KQLIEEMKEQGYMGEDTLKHWEKNKV------
015 YP_004442824.1   -------------------EFIQKMKKLGYIGEEPLKHWEKNQV------
016 YP_001931967.1   FL-------KTENIFEIQSTSEIEELLELACSENPLDPEKSKGL------
017 YP_002455786.1   --------------------NSAQKGELVGLLLGLQAAQPWPS-------
018 YP_009109694.1   --------------------TSAQKAELIALTKALELSKGKK--------
019 NP_789739.1      --------------------------------------------------
020 NP_955619.1      --------------------KSAQRAELLGLLHGLSSARSWRC-------
021 YP_224289.1      -------------------KLFKELKDQGYIGEEPLKHWQKNMV------
022 YP_009182100.1   RPKQRPNLNVLEEQISRCSIEELTKQLEPNISDNPIH-KWDVTQ------
023 NP_705927.1      --------------------TTNQKTELQAIYLALQDSGLE---------
024 YP_009508577.1   --------------------HTAQYAEISAVEFACKKANNISGP------
025 YP_004442830.1   ------------------KEIINEMKELGYIGDEPLRYWANNKV------
026 NP_758887.1      --------------------TTNQKAELEAIKMALQDSGPK---------
027 YP_009508406.1   --------------------------------------------------
028 YP_610965.1      -----------------AQKIVQRLLQQN-ISDDPLKFWAKNKV------
029 YP_001931961.1   GL-------KKLFEEDLKKKSIIEKLLDQVCSENPLDPLKTKKW------
030 YP_241114.1      --------------------SSAQVVELFAVHQALLTVPTS---------
031 YP_006732334.1   ---------ENNFIIDISRYQEIQELLKRVCSENPIDPQKSKGW------
032 YP_009041481.1   -----------------LGPILKDLMAQGYIGEDPVRHWVKNQV------
033 YP_001036293.1   -----------------LSQVFQELKAAGYIGDDPLKFWSKNQV------
034 YP_605811.1      -------------------SEIQKLKDLGYIGEEPLKHWAKNQV------
035 NP_955577.1      --------------------TSAQRAELIALTQALKMAEGKK--------
036 YP_003208050.1   -----------------FEGLIGRLRNLGFIGENPVKHWARNQV------
037 NP_789740.1      --------------------TTNQKTELQAIYLALQDSGLE---------
038 YP_004442839.1   -------------------VEIQKLKDLGYIGEEPLKHWAKNQV------
039 YP_009507791.1   --------------------GSNQQAEVNALRMALVDGPSE---------
040 YP_004581513.1   -----------------FRKEVAALIKEGFIGNNPLLHWTKNRV------
041 NP_043933.1      ----------LFSTIS--AYTEVEKLLDSICSEHPLDSRINKGK------
042 YP_009507248.1   --------------------NSTTDTVHTSDEHPILEVPITNEP------
043 YP_002117531.1   ------------------RALMERLKKIGIIGEDPLKFWERNKV------
044 YP_004347415.1   ----------NIN----EVEELNNILGEDIYGDNPLKHWEKHKT------
045 NP_056891.1      --------------------NSAQLVELQALIAVLSAFPNQP--------
046 YP_009513242.1   --------------------HTAQLAEIAAVEFACKKALKISGP------
```

```
047 YP_009352866.1  -----------------LGPILADLKAQGFIGEDPVKHWKKNQV------
048 YP_009165750.1  IN-------RRLFLTQQDQLSTIEKLLDEVCSENPIDPHKSKKW------
049 NP_040840.1     --------------------GTNQQLELRAIEEACKQGPEK---------
050 YP_004222728.1  --------------------ASVQQLEARAVAMALLLWPTTP--------
051 YP_009506264.1  -------------------QLFRNLKEAGYIGEEPLKHWSQNQI------
052 YP_567050.1     --------------------NSAQKGELLALLSGIRAAKSWPS-------
053 YP_009508571.1  --------------------HTAQMAEIAAVEFACKKALKITGP------
054 NP_056803.1     --------------------HTAQMAEIAAVEFACKKALKVPGP------
055 NP_955564.1     --------------------NTAQQAEIVAVITAFEEVSQP---------
056 YP_009508411.1  -----------------YEAILSRMKQQGYIGDKPMRHWSKNKV------
057 YP_009140788.1  -----------------FKPLIEEFQEAGYIGENPLQHWEKNGV------
058 YP_009508410.1  ------------------------------MNSESLEYKRALQA------
059 NP_040333.1     --------------------TSAQRAELIALTQALKMAEGKK--------
060 YP_008992013.1  ---------PSYQDVSTKLLSFCPESHSEFT--HPNPLWKNQSF------
061 NP_041734.1     -----------------LQQLMVQLQDQGFIGENPMQHWAKNKI------
062 NP_056895.1     --------------------ESAQKGELAGLLAGLAAAPPEP--------
063 YP_009109689.1  --------------------TSAQKAELIALTKALELSKGKK--------
064 YP_002519387.1  SKTLSEDSDDYFVYSIQRHNTIVNDLLTQACSENPLDENKNHNG------
065 YP_595725.1     ------------------KRLLEEMKEQGYMGEDTLAHWNKNQI------
066 YP_009508551.1  --------------------HTAQMAEIAAVEFACKKALKIPGP------
067 YP_001497148.1  --------------------TSAQKAELIALTKALELSEGKK--------
068 NP_041186.1     --------------------SSAQVVELFAVHQALLTVPTS---------
069 NP_663784.1     --------------------TTNQQAELEVFRMALADSGPK---------
070 YP_009508443.1  -----------------FEDLILELKSAGYIGEEPMKFWSHNKV------
071 YP_003987465.1  -------------------PLYQQLKAEGFIGENPLLHWQRNQV------
072 NP_861410.1     --------------SKTPLELKVSSTLEEVCDENPLDV-KNTNT------
073 YP_009345071.1  -----------------VAHKLLQLSDMGYIGEEPLKHWKKNQV------
074 YP_009508582.1  --------------------HTAQYAEISAVEFACKKANNISGP------
075 NP_569141.1     --------QLEIIIFTIDKVKIIQKKLEKLYNDNPLQGWEKHKT------
076 YP_002916057.1  -----------------FRKEVARLIQEGYIGENPLRHWSKNKV------
077 NP_777384.2     --------------------ESAQKGELAGLLAGLAAAPPEP--------
078 YP_233110.1     -------------------SEIRKLKDLGYIGEEPLKHWAKNQV------
079 NP_542258.1     --------------------CSAQAGELFGLLAGLQKSKPWPA-------
080 YP_009130664.1  -----------------YQKLIQELKDMGYIGDDPMKYWSSNKI------
081 YP_009508566.1  --------------------HTAQYAEIAAVEFACKKALQVTGP------
082 YP_006607892.1  VT-------QKLFSIQQDHLSKIHNLLDRVCSENPIDPEKSKRW------
083 YP_009506270.1  -------------------NLINRMKRLGYIGEEPLKHWEKNQV------
084 NP_042513.1     ----------------------VTQLLEAICSQNPLDPQKNRNQ------
085 YP_006907834.1  G--------EKLRITQER-MVKIEELLEKVCSENPLDPEKSKGW------
086 YP_004442827.1  ------------------RKLMEEMKELGYIGDEPLKHWNNNKI------
087 YP_009408594.1  -----------------YRGTLDRLKEQGYIGDNPLQHWEKNQL------
088 YP_009508561.1  --------------------HTAQYAEISAVEFACKKASLLQGP------
089 NP_056907.1     --------------------NSAQKGELLALLSGIRAAKSWPS-------
090 YP_002308474.1  --------------------SSAQAGELFALLAALRECKPWSS-------
091 YP_007761644.1  AERGGHITQVSENSETKHWKMQVEEILLDTFSDNPLALYNPRHPKCQIEL
092 NP_040550.1     --------------------ASVQQLEARAVAMALLLWPTTP--------
093 NP_758808.1     -----------------FSPLMNELKAAGYIGEDPLKHWSKNKV------
094 YP_009345075.1  -----------------IQKALQQMEELNFIGPNPQIHWAKNRV------
095 NP_068729.1     ----------------------IEEQLEEVCSEHPLDETKNKNG------
096 NP_047255.1     --------------------TSAQRAELIALTQALKMAEGKK--------
097 YP_006273075.1  -----------------FRPLLEELKEAGFIGENPLQHWKKNGI------
098 YP_009508888.1  --------------------HTAQLAEVAAVEFACKQALKITGP------
099 NP_395469.1     ----------------------VNKILQERFSLDLLGE-KNKNK------
100 YP_009508546.1  --------------------HTAQMAEIAAVEFACKKALKIPGP------
101 NP_056762.1     -----------EIGNQSLITMVKELEALGFIGDDITKNRTTWVCD-----
102 YP_009508556.1  --------------------HTAQLAEIAAVEFACKKALKISGP------
103 NP_569153.1     -----------------FAGLIQDLKAQGYIGEEPMKYWAKNQV------
104 NP_043924.1     ----------------DFQKQLAEIMPQQDISHCTLAYFDKPSPEATKYH
105 YP_009243641.1  --------------------NSIQKNELLAIIQVLKDFKQD---------
106 YP_004300274.1  -------------------FSLVRNQIKTLFSENPLEFWDKHRT------
107 NP_056848.1     ----------QIESRNPIEEEINQILGTDIYGENPLEKWEKHKT------
108 YP_006495799.1  -----------------DHRLLAELKEQGFIGNDPMLHWAKNQV------
109 YP_009508409.1  --------------------------------------------------
110 NP_687035.1     --------------------TTNQQAELTAVKMALEDSGEN---------
111 NP_045937.2     --------------------ASAQTAELLALAAACHLATDKTVN------
112 NP_612577.1     D--------EMLYEIQISKFSAIEEMLERVSSENPIDPEKSKQW------
113 NP_619548.1     ---------EERYFLITEKYQKIEQLLDKVCSENPIDPIKSKQW------
114 NP_056880.1     --------------------NTAQQAEIVAVITAFEEVSQP---------
115 YP_009121747.1  -----------------FIPILKELKQQGXIGEEPLKHWRKNGE------
116 YP_009113237.2  -----------------IQPLIEELKEQGTVGENPLQHWERNRV------
117 YP_009508408.1  -----------------FGDLIQKLKEAGYVGEEPMKFWQQNKV------
118 NP_955579.1     --------------------TSAQRAELIALTQALKMAEGKK--------
119 YP_009229919.1  -----------------FKPLLERLKEQGYVGEEPMRHWSKNQV------
```

```
120 YP_004442833.1     ------------------MKIINDMKELGFIGEEPLKHWANNKI------
121 NP_862833.2        --------------------SSAQVVELFAVHQALLTVSTS---------
122 NP_040563.1        --------------------GTNQQAELKAICMALLDGPPK---------
123 YP_443922.1        --------------------ASAQLAEIIGLTRALTLAEGKT--------
124 NP_057933.2        --------------------TSAQRAELIALTQALKMAEGKK--------
125 YP_009116631.1     -----------------NHRLLQELREQGYIGEEPMRHWAKNGI------
126 YP_009513249.1     --------------------HTAQFAEIAAFEFALKKCLPLGGN------
127 YP_223871.1        --------------------TSAQKAELIALTKALEWSKDKS--------
128 NP_127504.1        ---------ELQQLEDVIKNQLCADSHVDFLSKCSHPLWLNQDF------
129 NP_044929.1        --------------------HTAQYAEIAAFEFAIRRATGIRGP------
130 NP_057860.1        --------------------KSAQRAELLGLLHGLSSARSWRC-------
131 NP_040973.1        --------------------GSNQKAEIQALLLALKAGSEE---------
132 NP_777317.1        -------------------RLIEEMKRLGFIGDEPLKHWRQNKV------
133 NP_054716.1        --------------------HTAQFAEIAAFEFALKQAIRKMGP------
134 YP_001856242.1     --------------------TTNQKTELQAIYLALQDSGLE---------
135 NP_954565.2        --------------------NSAQLVELQALIAVLSAFPNQP--------
136 NP_569150.1        -------------------DIIGKMKKLGYIGEEPLKHWEKNQV------
137 NP_659397.1        ---------EEKGFIEISKFREIEQLLEKVCSENPIDPEKSKGW------
138 YP_009513211.1     --------------------TSAQKAELIALTQALRLAEGKS--------
139 YP_233107.1        -------------------SEIDRLKRLGFIGEEPLRHWKRNQI------
140 YP_009109692.1     --------------------TSAQKAELIALTKALELSKGKK--------
141 NP_057849.4        --------------------TTNQKTELQAIYLALQDSGLE---------
142 NP_049560.1        --------------------KSAQRAELLGLLHGLSSAHSWRC-------
143 YP_009506267.1     -----------------YEELIVQLKNAGYIGENPMKFWAKNKV------
144 NP_039820.1        ------------------KDLLKEMKEMKYIGENPMEFWKNNKI------
145 NP_056790.1        --------------------TSAQKAELVALTQALRLAEGKN--------
146 YP_002321513.1     -----------------FTSTIEDLKAQGVIGDAPLQLWERNQV------
147 NP_955591.1        --------------------TSAQRAELIALTQALKMAEGKK--------
148 YP_001956722.2     --------------------HTAQLAEVAAVEFACKKALKIDGP------
149 NP_056902.1        --------------------VTHQVAERMAIQMALEDTRDKQ--------
150 NP_056886.1        --------------------ASVQQLEARAVAMALLLWPTTP--------
151 NP_041261.1        --------------------LSAQLVELYAILQVFTVLAHQP--------
```

```
001 YP_004442836.1     --KCRIEVKNPDMIIEDKPLKHVTPAMKETMAKHVKKLLELKVIRPS---
002 NP_056728.1        -MKASIKLSDPSKAIKVKP-MKYSPMDREEFDKQIKELLDLKVIKPS---
003 YP_009508537.1     -----ILVVTDSNYVAKAYNEELDVWASNGFVNNRKKPLKHISKWKS---
004 YP_004732983.2     --RCKLDIINPDITIEAKPPGHLTLEDKVKYQKHIDALLDLGVIRPS---
005 YP_009508407.1     -----TEAIDPPAVGYIKP-------------TEK---LALAVINLQ---
006 NP_040939.1        -----MNLVTDSRYAFEFLLRNWDEEVIKNPIQARIMEIAHKKDRIG---
007 YP_003864102.1     -----LNIYTDSAYLAHSIPLLETVAQIKHIS-DTAQLFLQCQQLIH---
008 YP_009002585.1     --LCYLDIKNPDMVIEDKPIKHVTPQMEESFRKHIKGLLELKVIRPS---
009 YP_003284237.1     --KCSLEIKNPDMIIEDRPLKHVTPKMKEQMKKHVDKLLELKVIRPS---
010 YP_009268869.1     -----MNIVTDSRYAYEFMRRNWDEEVIKNPIQARIMKLVHDKEQIG---
011 YP_009506251.1     --HCKLDIKNPDLIIEDKPIKHLTPAMEKQFAKHIKALLDIGVIRPS---
012 NP_955611.1        -----TNVVTDSAFVAKMLLKMGQEGVPSTAA------AFILEDALS---
013 YP_008567619.1     --TCKLEIKNPDLIIEDRPLKHVTPAMHASFLKHIDALLKIKVIRPS---
014 NP_149413.1        --KCKIELINPDLIIKDKPITMLTIQTKEAMRKHVDALLARGVIRKS---
015 YP_004442824.1     --RCRIEVKNPDLIIEDRPLKHVTPKMKEAMAVHIKKLLELKVIRPS---
016 YP_001931967.1     -LTASIKLIDPNKIIRVKP-IPYPPNIRQEFDIQIKELLAMNLIVPS---
017 YP_002455786.1     -----LNIFLDSKFLIRYLQSLASGAFQGSST----HHRLQASLPTL---
018 YP_009109694.1     -----ANIYTDSRYAFATAHTHGSIYERRGLLTSEGKEIKNKAEIIA---
019 NP_789739.1        --------------------------------------------------
020 NP_955619.1        -----LNIFLDSKYLYHYLRTLALGTFQGRSS----QAPFQALLPRL---
021 YP_224289.1        --KCKLELKNPDITIQDKPLKHVTAKMRETMRAHIDKLLQLKVIRPS---
022 YP_009182100.1     -TYADIQLKDPYALIRVKP-MPYSAEDEAEFERQLEEQLKLELIQPS---
023 NP_705927.1        -----VNIVTDSQYALGIIQAQ--PDQSESELVNQIIEQLIKKEKVY---
024 YP_009508577.1     -----VLIVTDSDYVARSVNEELPFWRSNGFVNNKKKPLKHISKWKN---
025 YP_004442830.1     --KCHIRIKNPELTIQDKPHKFVTPQMKEQMQKHMDELLKRKVIRPS---
026 NP_758887.1        -----VNIVTDSQYAMGILSAA--PDQSDNPIVREIIELMIHKEAVY---
027 YP_009508406.1     --------------------------------------------------
028 YP_610965.1        --TCQLEIINPDLTIQDKPLKHVTPLMEQQFKRHVEALLQLKVIRPS---
029 YP_001931961.1     -MKASIKLIDPKTVVKVKP-MRYNPQDVEEFAKQIKELLELKIIIPS---
030 YP_241114.1        -----FNLFTDSSYVVGALQMIETVPIIGTTSPEVFNLFTLIQQVLH---
031 YP_006732334.1     -MTASIKLADPKSVVKVKP-MVYSPQDRKEFEIQIKELLNLKVIIPS---
032 YP_009041481.1     --ICRLDIINPDITIQSQPLKHVTVEMERSFQTHVDGLLKLKVIRPS---
033 YP_001036293.1     --VCELNIINPDLTIQDKPLKHVTPAMEETFRKHIDALLKLKVIRAS---
034 YP_605811.1        --KCKIEIKNPDLIIEDRPLKHVTPAMKESMKKHIDKLLELKVIRPL---
035 NP_955577.1        -----LTVYTDSRYAFATTHVHGEIYRRRGLLTSEGKEIKNKNEILA---
036 YP_003208050.1     --KCRLEIINPDLTIQDKPLKHVTPQMEAQFKRHTDALLQLGVIRPS---
037 NP_789740.1        -----VNIVTDSQYALGIIQAQ--PDQSESELVNQIIEQLIKKEKVY---
038 YP_004442839.1     --RCRIEVKNPDLIIEDRPLKHVTPAMKESMKRHVDKLLELKVIRPS---
```

```
039 YP_009507791.1   -----MNIITDSQYIMNVLKQ--YPDSLSG-LWQKIIELLQSKIKIF---
040 YP_004581513.1   --YCKLQIKNTDLIIQDPPLKHVTPAAREFFKSQISDLLKAKLIRPS---
041 NP_043933.1      -FEAQIALLDTNKIIKCKP-MQYSPQDREEFKTQIEELLKLGIIRPS---
042 YP_009507248.1   -----LNRFHRQIHLTVVGDIKRRPIVTKPFESHTRIAIQLSESNLEQDV
043 YP_002117531.1   --KCKLDIINPNLTIEDKPLKHVTPAMKEQMRKHIDKLLELKVIRPS---
044 YP_004347415.1   --YAKIELKNPDDHIYKPP-INYQESDYKEFKMHIDEMVKEGFIEECKNL
045 NP_056891.1      -----LNIYTDSAYLAHSIPLLETVAQIKHIS-ETAKLFLQCQQLIY---
046 YP_009513242.1   -----VLIVTDSFYVAESANKELSYWKSNGFLNNKKKPLKHVSKWKS---
047 YP_009352866.1   --ICKLEIINPDITIQAQPLKHVTAEMEKSFKTQVDGLLKLRVIRPS---
048 YP_009165750.1   -MTASIKLKDPNTLVQVKP-MQYCPEDRKEFAVQIKELLDLKIIIPS---
049 NP_040840.1      -----MNIVTDSRYAYEFMLRNWDEEVIRNPIQARIMELVHNKEKIG---
050 YP_004222728.1   -----TNVVTDSAFVAKMLLKMGQKGVPSTAA------AFILEDALS---
051 YP_009506264.1   --KCRLEIKNPDMIIEDRPLKHVTPKLKEDMQKHIDQLLKLKVIRPS---
052 YP_567050.1      -----LNIFLDSKYLIKYLHSLAIGAFLGTST----HQSLYAHLPTL---
053 YP_009508571.1   -----VLIITDSFYVAESTNKELPYWKSNGFVNNKKKPLKHVSKWKS---
054 NP_056803.1      -----VLVITDSFYVAESANKELPYWKSNGFVNNKKEPLKHISKWKS---
055 NP_955564.1      -----FNLYTDSKYVTGLFPEIETATLSPRTK--IYTELKHLQRLIH---
056 YP_009508411.1   --MCKLDIINPNLIIQDKPLKHVTPAMTETFKKHIDALLELKVIRPS---
057 YP_009140788.1   --LCQLDIKNPDFIIEDRPLKSVTPQMKESFKRHVKALLDLKVIRPS---
058 YP_009508410.1   -----VESLEPPALGFIKP-------------ADFRAGLATASAAVQ---
059 NP_040333.1      -----LNVYTDSRYAFATAHIHGEIYRRRGLLTSEGKEIKNKDEILA---
060 YP_008992013.1   -FVKLPFKLNEDINPTKATHPGMSPSDLLLAQQECSQLLAQGLIEPT---
061 NP_041734.1      --LCRLDIKNPDLIIEDKPIKHLTPAMEKQFQKHIKALLDIGVIRPS---
062 NP_056895.1      -----LNIWVDSKYLYSLLRTLVLGAWLQPDP----VPSYALLYKSL---
063 YP_009109689.1   -----ANIYTDSRYAFATAHTHGSIYERRGLLTSEGKEIKNKAEIIA---
064 YP_002519387.1   -LLAEIKLINPATTVNVKS-MAYSPDDAVEINKQIQELLEIKVIRPS---
065 YP_595725.1      --KCKIELRNPDLIIKDKPQTLLNIQKKEAMRKHIDALLERKVIRPS---
066 YP_009508551.1   -----VLVITDSFYVAESANKELPYWKSNGFVNNKKKPLKHISKWKS---
067 YP_001497148.1   -----ANIYTDSRYAFATAHTHGSIYERRGLLTSEGKEIKNKAEIIA---
068 NP_041186.1      -----FNLFTDSSYVVGALQMIETVPIIGTTSPEVLNLFTLIQQVLH---
069 NP_663784.1      -----VNIIVDSQYVMGIVAGQ--PTESENRIVNQIIEEMIKKEAVY---
070 YP_009508443.1   --TCKLEIINPELTIQDKPLKHVTPSMEEAFRKHVQALLKLKVIRPS---
071 YP_003987465.1   --ICELQIKNPELTIEDRPLKHVTPALKEAMQKHVDKLLELKVIRPS---
072 NP_861410.1      -ELVKIELINPEKEVNVPNNIPYSLRDINEFSQECADLVRKGIIEES---
073 YP_009345071.1   --ECKLEIINPQLKIEDKPLKHITPQMQESFNKQVESLLKLGVIRPS---
074 YP_009508582.1   -----VLIVTDSDYVARSVNEELPFWRSNGFVNNKKKPLKHISKWKN---
075 NP_569141.1      --KVKIELIEENSIITQKP-LKYNFNDLTEFKIHIKDLLDNKYIQES---
076 YP_002916057.1   --ECTLRIKNPDLVIQDPPLKHVTPAAREFFQNQVSSLIKAQLIRPS---
077 NP_777384.2      -----LNIWVDSKYLYSLLRTLVLGAWLQPDP----VPSYALLYKSL---
078 YP_233110.1      --KCRIEIKNPDLIIEDRPLKHVTPAMKESMKKHVDKLLELKVIRPS---
079 NP_542258.1      -----LNIFLDSKFLIGHLRRMALGAFLGPST----QCDLHARLFPL---
080 YP_009130664.1   --TCQLEIKNPDLTIEDRPLKHISPQMEASYRRHTEAVLKLGTIRPS---
081 YP_009508566.1   -----VLIVTDSDYVARSVNNELNFWRSNGFVNNKKKPLKHISKWKS---
082 YP_006607892.1   -MTASIKLKDPNTIVKVKP-MQYNPDDRMEFSKQIKELLDLKIIIPS---
083 YP_009506270.1   --ECRIEVKNPDMIIEDEPLKHVTPAMKETMAKHVKKLLELKVIRPS---
084 NP_042513.1      -IIVHIDLIDPTKEVNVPNRIPYTQKDIDEFREETSKQIELGILRQS---
085 YP_006907834.1   -MQASIKLSDPTKVIKVKP-MKYSPMDREEFEKQIQELLDLKVIRPS---
086 YP_004442827.1   --KCHIRIKNPELTIQDKPQKMVTPQIKEQMKKHMDELLLRRVIRPS---
087 YP_009408594.1   --KCHIDIINPDITIQDPPLKHVTPALKETFQKHIDALLKLGVIRES---
088 YP_009508561.1   -----VLIVTDSDYVARSANKELPFWRSNGFLNNKKKPLKHISKWKN---
089 NP_056907.1      -----LNIFLDSKYLIKYLHSLAIGAFLGTST----HQSLYAHLPTL---
090 YP_002308474.1   -----LNIFLDSKFLVGQLRRLALGAFIGPST----QCDLHSQLLPL---
091 YP_007761644.1   LPQDQLDRLKLKCPVRCKP-IGANPIDMEEFHNQINELLRLKLIRKT---
092 NP_040550.1      -----TNVVTDSAFVAKMLLKMGQEGVPSTAA------AFILEDALS---
093 NP_758808.1      --TCKLDLKNTEITIQDKPLRHITPALEQSYGRHVNALLMLKVIQPS---
094 YP_009345075.1   --VCKIDIINPDLTIEDRPLKHITPEMKAQFSRHTEALLKLGVIRKS---
095 NP_068729.1      -LLIEIRLKDPLQEINVTNRIPYTIRDVQEFKEECEDLLKKGLIRES---
096 NP_047255.1      -----LTVYTDSRYAFATTHVHGEIYRRRGLLTSEGKEIKNKNEILA---
097 YP_006273075.1   --LCQLDIKNPDFIIEDRPLKNLTPQMKESFKKHIKVLLDLGVIRAS---
098 YP_009508888.1   -----VLIITDSFYVAESANKELPYWKSNGFVNNKKKPLKHVSKWKS---
099 NP_395469.1      -ELIEIKLKDPNAEIFVPNNIPYTQRDIEEFKEDMEDLINKGLIRPS---
100 YP_009508546.1   -----VLIITDSFYVAESANKELPYWKSNGFVNNKKKPLKHISKWKS---
101 NP_056762.1      -----FKIINPDINITCAT-IPYTPADKEVFEKQIKELLDNKLIKKADP-
102 YP_009508556.1   -----VLIVTDSFYVAESANKELSYWKSNGFLNNKKKPLRHVSKWKS---
103 NP_569153.1      --VCHLDIKNPDMVIEDRPLKHVTPQMEESFRKHVEALLKIGAIRPS---
104 NP_043924.1      DKIKPYLGKGQQLTLCDTYIGKEGAAILGQLRPDMQALHQAEGEVHVSLG
105 YP_009243641.1   -----INIIADSAYVVGVVKNIIG-AVINSLDKELTTLFKTLQVLLT---
106 YP_004300274.1   --EVKIELINPDSIVYQKP-LRWNFEDIEEFKLHIDELLKGGFIRPS---
107 NP_056848.1      --LAKIELKNETDNIYKPP-MLYQETDLPEFKMHIEEMIKEGFIEEKTNF
108 YP_006495799.1   --KCKLDIINPDITIQGKPPSTATPEIKDRYQRHIDALLSIGVIRPS---
109 YP_009508409.1   --------------------------------------------------
110 NP_687035.1      -----VNIVTDSQYVMNILTAC--PQESNSPLVEQIIQALMKKRQVY---
111 NP_045937.2      -------IYTDSRYAYGVVHDFGHLWMHRGFVTSAGTPIKNHKEIEYLLK
```

```
112 NP_612577.1        -MTATIELIDPKTVVKVKP-MSYSPSDREEFDRQIKELLELKVIKPS---
113 NP_619548.1        -MKASIKLIDPLKVIRVKP-MSYSPQDREGFAKQIKELLDLGLIIPS---
114 NP_056880.1        -----FNLYTDSKYVTGLFPEIETATLSPRTK--IYTELKHLQRLIH---
115 YP_009121747.1     --TCKLDIINPDITVQDKPLKHVTPALEASFKKXIEALLKLKVIRPS---
116 YP_009113237.2     --HCYLDIKNPDLTVQDKPLDQITPVQKEMYKKHIDALLQIGVIRRS---
117 YP_009508408.1     --TCKLDIINPELRIQDKPLKHVTPAMEETFRKHVQALLKLKVIRPS---
118 NP_955579.1        -----LTVYTDSRYAFATTHVHGEIYRRRGLLTSEGKEIKNKNEILA---
119 YP_009229919.1     --KCKLEIINPDITIQDKPMKHVTPAMKDQFQKHTQALLKLGVIRPS---
120 YP_004442833.1     --KCHIRIRNPELTIQDKPHKFVTPQMKEQMQFHMNELLKRKVIRPS---
121 NP_862833.2        -----FNLFTDSSYVVGALQMIETVPIIGTTSPEVLNLFTLIQQALH---
122 NP_040563.1        -----MNIITDSRYAYEGMREE-PETWAREGIWLEIAKILPFKQYVG---
123 YP_443922.1        -----VNIYTDSAYAHGAVHIDGPQWLRRNFTTTGNLPIKHKTQMEV---
124 NP_057933.2        -----LNVYTDSRYAFATAHIHGEIYRRRGLLTSEGKEIKNKDEILA---
125 YP_009116631.1     --KCKLDIKNPDIVISSKPPDSVSKETKAQYQRHIDALLKIGVIQPS---
126 YP_009513249.1     -----ILVVTDSNYVAKAYNEELDVWASNGFVNNRKKPLKHISKWKS---
127 YP_223871.1        -----VNIYTDSRYAFATLHVHGMIYRERGLLTAGGKAIKNAPEILA---
128 NP_127504.1        -FIQLPFKKNENINPTKASHSGMNPEHLQLAIKECDELQQFDLIEPS---
129 NP_044929.1        -----VLIVTDSNYVAKSYNEELPYWESNGFVNNKKKTLKHISKWKA---
130 NP_057860.1        -----LNIFLDSKYLYHYLRTLALGTFQGRSS----QAPFQALLPRL---
131 NP_040973.1        -----MNIITDSQYVINIILQ--QPDMMEG-IWQEVLEELEKKTAIF---
132 NP_777317.1        --TCKLEIKNPDLIIEDKPLKHVTPKMKEVMARHVTALLQSKVIRPS---
133 NP_054716.1        -----VLIVTDSDYVAKSYNQELDFWVSNGFVNNKKKPLKHVSKWKS---
134 YP_001856242.1     -----VNIVTDSQYALGIIQAQ--PDQSESELVNQIIEQLIKKEKVY---
135 NP_954565.2        -----LNIYTDSAYLAHSIPLLETVAQIKHIS-ETAKLFLQCQQLIY---
136 NP_569150.1        --KCRIEVKNPDMIIEDRPLKHVTPTMKETMAKHVQKLLELKVIRPS---
137 NP_659397.1        -MKAEIKLIKPETVVRVKP-MVYSPEDRKEFEIQIKELLDLKVIIPS---
138 YP_009513211.1     -----INIYTDSRYAFATAHVHGAIYKQRGLLTSAGKDIKNKEEILA---
139 YP_233107.1        --KCKLEIKNPDLIIEDRPLKHVTPAMKETMTKHVQRLLDIKVIRPS---
140 YP_009109692.1     -----ANIYTDSRYAFATAHTHGSIYERRGLLTSEGKEIKNKAEIIA---
141 NP_057849.4        -----VNIVTDSQYALGIIQAQ--PDQSESELVNQIIEQLIKKEKVY---
142 NP_049560.1        -----LNIFLDSKYLYHYLRTLALGTFQGRSS----QAPFQDPLPRL---
143 YP_009506267.1     --QCKLEIINPNLTIQDKPLKHVTPAMEATFKKHVDALLELKVIRPS---
144 NP_039820.1        --KCKLNIINPDIKIMGRPIKHVTPGDEEAMTRQINLLLQMKVIRPS---
145 NP_056790.1        -----INIYTDSRYAFATAHIHGAIYKQRGLLTSAGKDIKNKEEILA---
146 YP_002321513.1     --KCKLEVINPDITISDKPLKHVSIGLKQQFQNQLDPLLKMGLIRPS---
147 NP_955591.1        -----LNVYTDSRYAFATAHIHGEIYRRRGLLTSEGKEIKNKDEILA---
148 YP_001956722.2     -----VLIVTDSFYVAESVNKELPYWQSNGFFNNKKKPLKHVSKWKS---
149 NP_056902.1        -----VNIVTDSYYCWKNITEGLGLEGPQS-PWWPIIQNIREKEIVY---
150 NP_056886.1        -----TNVVTDSAFVAKMLLKMGQEGVPSTAA------AFILEDALS---
151 NP_041261.1        -----FNLYTDSAYIAQSVPLLETVPFIKSST-NATPLFSKLQQLIL---


001 YP_004442836.1     -QSKHRTTAMIVESGTEIDPVTGK--------------------------
002 NP_056728.1        -KSPHMAPAFLVNNEAEKR-------------------------------
003 YP_009508537.1     -VADFKKLRPDVVVTHEPG-------------------------------
004 YP_004732983.2     -KSRHRSAAFIVASGTSVDPKTGK--------------------------
005 YP_009508407.1     -KQNNTLTNLITQIFEKLT-------------------------------
006 NP_040939.1        -VHWVPGHKGIPQN---EEIDKYISEIFLAKEGEGILPKREEDAGYDLIC
007 YP_003864102.1     -NRSTPIYIGHVRA------------------------------------
008 YP_009002585.1     -TSKHRTTAFIVNSGTSVDPVTGK--------------------------
009 YP_003284237.1     -TSKHRTTAMIVESGTEIDPKTGQ--------------------------
010 YP_009268869.1     -VHWVPGHKGIPQN---EEIDKYISEIFLAREGSGILPKRAEDAGYDLIC
011 YP_009506251.1     -KSRHRTTAFIVESGTTIDPKTKK--------------------------
012 NP_955611.1        -QRSAMAAVLHVRS------------------------------------
013 YP_008567619.1     -KSRHRTCAFIVNSGTSIDPVTGK--------------------------
014 NP_149413.1        -NSPHRTNAFIVESGTSVDPVTKK--------------------------
015 YP_004442824.1     -TSKHRTTAMIVESGTEVDPVTGI--------------------------
016 YP_001931967.1     -KSPHMSPAFMVNKGAEQR-------------------------------
017 YP_002455786.1     -LQGKVVYLHHTRS------------------------------------
018 YP_009109694.1     -LLKALFLPQEVAIIHCPG-------------------------------
019 NP_789739.1        --------------------------------------------------
020 NP_955619.1        -LSRKVVYLHHVRS------------------------------------
021 YP_224289.1        -SSRHRTTAMIVESGTEVDPKTGQ--------------------------
022 YP_009182100.1     -KSPHSSPAFCVRNHAEIK-------------------------------
023 NP_705927.1        -LAWVPAHKGIGGN------------------------------------
024 YP_009508577.1     -ISDSLLLKRDIIIVHEPG-------------------------------
025 YP_004442830.1     -SSRHRTNAFIVNSGTTVDPLTKK--------------------------
026 NP_758887.1        -LAWVPAHKGIGGN------------------------------------
027 YP_009508406.1     --------------------------------------------------
028 YP_610965.1        -KSRHRTMAMIGNSGTSVDPTTGK--------------------------
029 YP_001931961.1     -KSPHQSPAFLVENEAERR-------------------------------
030 YP_241114.1        -CRQYPCFFGHIRA------------------------------------
```

```
031 YP_006732334.1  -KSQHMSPAFLVEKEAEKR-------------------------------
032 YP_009041481.1  -KSRHRTLAILVKSGTSIDPLTGK--------------------------
033 YP_001036293.1  -KSRHRTTAFIVYSGTTVDPVTGK--------------------------
034 YP_605811.1     -TSKHRTTAIIVQSGTEIDPLTGK--------------------------
035 NP_955577.1     -LLEALFLPKRLSIIHCPG-------------------------------
036 YP_003208050.1  -KSRHRTMAIMVQSGTTVDPATGK--------------------------
037 NP_789740.1     -LAWVPAHKGIGGN------------------------------------
038 YP_004442839.1  -ESKHRTTAIIVQSGTEIDPLTGK--------------------------
039 YP_009507791.1  -LDWVPGHKNIPGN---VEVDR-ICQENMIIEGDGIIDKREEDAGYDLIA
040 YP_004581513.1  -KSKHRTTAFMVESGTIVDPKTGK--------------------------
041 NP_043933.1     -KSPHSSPAFMVRNHAEIK-------------------------------
042 YP_009507248.1  ISAIKEYVNPKVKTALIIN-------------------------------
043 YP_002117531.1  -KSRHRTTAMIVQSGTEMVLQKDKNGKEKMV-------------------
044 YP_004347415.1  ENKKYSSPAFIVNKHSEIK-------------------------------
045 NP_056891.1     -NRSIPFYIGHVRA------------------------------------
046 YP_009513242.1  -IAECLQLKPDITIIHEKG-------------------------------
047 YP_009352866.1  -KSRHRTLALLVKSGTSIDPLTGK--------------------------
048 YP_009165750.1  -KSPHRSPAFLVENEAERR-------------------------------
049 NP_040840.1     -VHWVPGHKGIPQN---EEIDRYISEIFLAKEGRGILQKRAEDAGYDLIC
050 YP_004222728.1  -QRSAMAAVLHVRS------------------------------------
051 YP_009506264.1  -ASKHRTTAMLVESGTEVDPKTGL--------------------------
052 YP_567050.1     -LHNKVIYLHHIRS------------------------------------
053 YP_009508571.1  -IAECLSLKPDITIQHERG-------------------------------
054 NP_056803.1     -IAECLSIKPDITIQHEKG-------------------------------
055 NP_955564.1     -KRQEKFYIGHIRG------------------------------------
056 YP_009508411.1  -TSKHRTTAFIVHSGTTVDPETGL--------------------------
057 YP_009140788.1  -KSRHRTTAMLVNSGTTVDPKTGK--------------------------
058 YP_009508410.1  -KQNNTIIQLLLQLSEKVN-------------------------------
059 NP_040333.1     -LLKALFLPKRLSIIHCPG-------------------------------
060 YP_008992013.1  -TSQWACQAFYVEKHSEIV-------------------------------
061 NP_041734.1     -KSKHRTTAFIVESGTVIDPVTKK--------------------------
062 NP_056895.1     -LRHPAIFVGHVRS------------------------------------
063 YP_009109689.1  -LLKALFLPQEVAIIHCPG-------------------------------
064 YP_002519387.1  -RSPHSSPCFLVQNHNEIK-------------------------------
065 YP_595725.1     -KSPHRTNAFIVESGTSIDPKTGK--------------------------
066 YP_009508551.1  -IAECLSMKPDITIQHEKG-------------------------------
067 YP_001497148.1  -LLKALFLPRRVAIIHCPG-------------------------------
068 NP_041186.1     -CRQHPCFFGHIRA------------------------------------
069 NP_663784.1     -VAWVPAHKGIGGN------------------------------------
070 YP_009508443.1  -TSRHRTTAFIVHSGTTVDPKTGV--------------------------
071 YP_003987465.1  -TSRHRTTAMIVYSGTEVDPVTKK--------------------------
072 NP_861410.1     -KSPHSAPAFYVENHNEIK-------------------------------
073 YP_009345071.1  -KSQHRTTAFLVKSGTTVDPKTGK--------------------------
074 YP_009508582.1  -ISDSLLLKRDITIVHEPG-------------------------------
075 NP_569141.1     -NSKHTSPAFIVNKHSEQK-------------------------------
076 YP_002916057.1  -RSRHRTTAFMVESGTSVDPKTGK--------------------------
077 NP_777384.2     -LRHPAIFVGHVRS------------------------------------
078 YP_233110.1     -TSKHRTTAIIVQSGTEIDPLTGK--------------------------
079 NP_542258.1     -LQGKTVYVHHVRS------------------------------------
080 YP_009130664.1  -KSKHRTTAIIVNSGTTIDPITGK--------------------------
081 YP_009508566.1  -ISESLLLHKNITIVHEPG-------------------------------
082 YP_006607892.1  -KSPHQSPAFLVENEAERR-------------------------------
083 YP_009506270.1  -QSKHRTTAIIVQSGTEIDPVTGK--------------------------
084 NP_042513.1     -KSPHSAPAFYVENHNEIK-------------------------------
085 YP_006907834.1  -KSPHMAPAFLVNNEAEKR-------------------------------
086 YP_004442827.1  -TSRHRTNAFIVNSGTSIDPITMK--------------------------
087 YP_009408594.1  -SSRHRTMAMIVKSGTTVDPITGQ--------------------------
088 YP_009508561.1  -ISDSLLLKRNITIVHEPG-------------------------------
089 NP_056907.1     -LHNKVIYLHHIRS------------------------------------
090 YP_002308474.1  -LYNKTIYVHHVRS------------------------------------
091 YP_007761644.1  -NSPWSFQAFMVRNHAEIV-------------------------------
092 NP_040550.1     -QRSAMAAVLHVRS------------------------------------
093 NP_758808.1     -KSRHRTMAFLVNSGTTVT-ADGK--------------------------
094 YP_009345075.1  -SSRHRTNAMIVRSGTSVDPKTGE--------------------------
095 NP_068729.1     -QSPHSAPAFYVENHNEIK-------------------------------
096 NP_047255.1     -LLEALFLPKRLSIIHCPG-------------------------------
097 YP_006273075.1  -KSRHRTTAMLVNSGTTVDPKTGK--------------------------
098 YP_009508888.1  -IADCLSLKTGITIKHEKG-------------------------------
099 NP_395469.1     -KSPHSAPAFYVENHSEIK-------------------------------
100 YP_009508546.1  -IAECLSLKPDIVIQHEKG-------------------------------
101 NP_056762.1     -TCRHRTAAFIVRNHSEEVAQ-----------------------------
102 YP_009508556.1  -IAECLQLKPDITIIHEKG-------------------------------
103 NP_569153.1     -KSRHRTTAIIVNSGTSIDPITGK--------------------------
```

```
104 NP_043924.1        TRAGHCPQELGTMLTNLLKSTQER--------------------------
105 YP_009243641.1     -SRNARIFIVHIRS------------------------------------
106 YP_004300274.1     -NSKHSSPAFIVNKHSEQK-------------------------------
107 NP_056848.1        EDKKYSSPAFIVNKHSEQK-------------------------------
108 YP_006495799.1     -KSRHRTAAFITYSGTSVDPKTGE--------------------------
109 YP_009508409.1     --------------------------------------------------
110 NP_687035.1        -LQWVPAHKGIGGN------------------------------------
111 NP_045937.2        QIMKPKQVSVIKIEAHTKGVSMEVRG------------------------
112 NP_612577.1        -KSTHMSPAFLVENEAERR-------------------------------
113 NP_619548.1        -KSQHMSPAFLVENEAERR-------------------------------
114 NP_056880.1        -KRQEKFYIGHIRG------------------------------------
115 YP_009121747.1     -KSRHRTMAMIVNSGTTVDPATGK--------------------------
116 YP_009113237.2     -NSRHRTNAFIVHSGTTVDPRTGE--------------------------
117 YP_009508408.1     -TSKHRTTAFIVHSGTTIDPKTGV--------------------------
118 NP_955579.1        -LLEALFLPKRLSIIHCPG-------------------------------
119 YP_009229919.1     -KSRHRTMAMIVYSGTSVDEKTGK--------------------------
120 YP_004442833.1     -TSRHRTNAFIVNSGTTVDPITRK--------------------------
121 NP_862833.2        -CRQHPCFFGHIRA------------------------------------
122 NP_040563.1        -VGWVPAHKGIGGN--------------------------TEADEGVKKA
123 YP_443922.1        -LISAVALPKKVAIMKCKG-------------------------------
124 NP_057933.2        -LLKALFLPKRLSIIHCPG-------------------------------
125 YP_009116631.1     -KSKHRTAAFITHSGTSIDPITKK--------------------------
126 YP_009513249.1     -VADLKRLRPDVVVTHEPG-------------------------------
127 YP_223871.1        -LLTAVWLPKRVAVMHCKG-------------------------------
128 NP_127504.1        -DSQWACEAFYVNKRSEQV-------------------------------
129 NP_044929.1        -IAECKNLKADIHVIHEPG-------------------------------
130 NP_057860.1        -LSRKVVYLHHVRS------------------------------------
131 NP_040973.1        -IDWVPGHKGIPGN---EEVDK-LCQTMMIIEGDGILDKRSEDAGYDLLA
132 NP_777317.1        -TSKHRTTAIIVESGTEVDPITGK--------------------------
133 NP_054716.1        -IADCKKHKADIHVIHEPG-------------------------------
134 YP_001856242.1     -LAWVPAHKGIGGN------------------------------------
135 NP_954565.2        -NRSIPFYIGHVRA------------------------------------
136 NP_569150.1        -SSKHRTTAMIVESGTEVDPMTGK--------------------------
137 NP_659397.1        -KSQHMSPAFLVEKEAEKR-------------------------------
138 YP_009513211.1     -LLEAIHLPKRVAIIHCPG-------------------------------
139 YP_233107.1        -TSKHRTTAIMVNSGTEIDPITGA--------------------------
140 YP_009109692.1     -LLKALFLPQEVAIIHCPG-------------------------------
141 NP_057849.4        -LAWVPAHKGIGGN------------------------------------
142 NP_049560.1        -LSRKVVYLHHVRS------------------------------------
143 YP_009506267.1     -TSQHRTTAFIVYSGTTVDPATGK--------------------------
144 NP_039820.1        -ESKHRSTAFIVRSGTEIDPITGK--------------------------
145 NP_056790.1        -LLEAIHLPRRVAIIHCPG-------------------------------
146 YP_002321513.1     -TSRHRTMAMIINSGTTVDPVTGE--------------------------
147 NP_955591.1        -LLKALFLPKRLSIIHCPG-------------------------------
148 YP_001956722.2     -IADCIQLKPDIIIIHEKG-------------------------------
149 NP_056902.1        -FAWVPGHKGIYGNQLADEAAKIKEEIMLAYQGTQIKEKRDEDAGFDLCV
150 NP_056886.1        -QRSAMAAVLHVRS------------------------------------
151 NP_041261.1        -NRQHPFFIGHLRA------------------------------------
```

```
001 YP_004442836.1     ----------------------------------EKRGKERLVFNYKRLN
002 NP_056728.1        ------------------------------------RGKKRMVVNYKAMN
003 YP_009508537.1     ---------------------------------------HQKLDSSPHAY
004 YP_004732983.2     ----------------------------------ETRGKERMVIDYRMLN
005 YP_009508407.1     ----------------------------------------QIQTEVRSLK
006 NP_040939.1        PEEVTIEPGQVKCIPIELRLNLKKSQWAMIATKSSMAAKGVFTQGGIIDS
007 YP_003864102.1     ----------------------------------------HSGLPGPIAQ
008 YP_009002585.1     ----------------------------------ETKGKERMVFNYKRLN
009 YP_003284237.1     ----------------------------------EKRGKERLVFNYKRLN
010 YP_009268869.1     PQEVCIPAGQVRKIPINLRINLKEDQWAMVGTKSSFASKGVFVQGGIIDS
011 YP_009506251.1     ----------------------------------TIHGKERMVFNYKRLN
012 NP_955611.1        ----------------------------------------HSEVPGFFTE
013 YP_008567619.1     ----------------------------------EVKGKERLVFNYKRLN
014 NP_149413.1        ----------------------------------EIRGKPRLVFNYKRLN
015 YP_004442824.1     ----------------------------------EKRGKERLVFNYKRLN
016 YP_001931967.1     ------------------------------------RGKMRMVVNYKALN
017 YP_002455786.1     ----------------------------------------HTQLPDPIST
018 YP_009109694.1     ---------------------------------------HQKGQDP-VAV
019 NP_789739.1        --------------------------------------------------
020 NP_955619.1        ----------------------------------------HTNLPDPISR
021 YP_224289.1        ----------------------------------EKRGKERLVFNFKRLN
022 YP_009182100.1     ------------------------------------RQKARIVINYKELN
```

```
023 NP_705927.1      ---------------------------------------------EQVDK
024 YP_009508577.1   ---------------------------------------HKPSYTSIHTQ
025 YP_004442830.1   ----------------------------------EIKGKPRLVFNYKRLN
026 NP_758887.1      ---------------------------------------------EQVDK
027 YP_009508406.1   --------------------------------------------------
028 YP_610965.1      ----------------------------------EVKGKERMVFNYRSLN
029 YP_001931961.1   ------------------------------------RGKKRMVVNYKAIN
030 YP_241114.1      ----------------------------------------HSTLPGALVR
031 YP_006732334.1   ------------------------------------RGKKRMVVNYKKLN
032 YP_009041481.1   ----------------------------------EVKGKERMVYDYRQLN
033 YP_001036293.1   ----------------------------------ENKGKERMVFNYKRLN
034 YP_605811.1      ----------------------------------ERRGKERLVFNYKRLN
035 NP_955577.1      ---------------------------------------HQKGDSP-QAK
036 YP_003208050.1   ----------------------------------ETRGKERMVYNYRSLN
037 NP_789740.1      ---------------------------------------------EQVDK
038 YP_004442839.1   ----------------------------------EKRGKERLVFNYKRLN
039 YP_009507791.1   QEDVFLMKGEVRIVPINAKIMLPEGTWGLIIGKSSIGKLGLDVLGGVIDQ
040 YP_004581513.1   ----------------------------------EIRGKQRMVYNYKRLN
041 NP_043933.1      ------------------------------------RGKARMVINYKKLN
042 YP_009507248.1   ----------------------------------------PPLKMYSIIP
043 YP_002117531.1   ----------------------------------EKRGKERLVFNYKRLN
044 YP_004347415.1   ------------------------------------RGKSRMVIDYKDLN
045 NP_056891.1      ----------------------------------------HSGLPGPIAQ
046 YP_009513242.1   ---------------------------------------HQQPMTTLHTE
047 YP_009352866.1   ----------------------------------EVKGKERMVYDYRQLN
048 YP_009165750.1   ------------------------------------RGKKRMVVNYKKLN
049 NP_040840.1      PQEISIPAGQVKRIAIDLKINLKKDQWAMIGTKSSFANKGVFVQGGIIDS
050 YP_004222728.1   ----------------------------------------HSEVPGFFTE
051 YP_009506264.1   ----------------------------------EKKGKQRLVFNYKRLN
052 YP_567050.1      ----------------------------------------HTNLPDPIST
053 YP_009508571.1   ---------------------------------------HQPIYTSIHTE
054 NP_056803.1      ---------------------------------------HQPINTSIHTE
055 NP_955564.1      ----------------------------------------HTGLPGPLAQ
056 YP_009508411.1   ----------------------------------EKKGKERLVFNYKRLN
057 YP_009140788.1   ----------------------------------ETKGKERMVFNYKRLN
058 YP_009508410.1   ----------------------------------------SLQAEIRTLR
059 NP_040333.1      ---------------------------------------HQKGNRA-EAR
060 YP_008992013.1   ------------------------------------RGKKRLVIDYQPLN
061 NP_041734.1      ----------------------------------TIHGKERLVFNYKRLN
062 NP_056895.1      ----------------------------------------HSSASHPIAS
063 YP_009109689.1   ---------------------------------------HQKGQDP-VAV
064 YP_002519387.1   ------------------------------------RGKKRLVINYKALN
065 YP_595725.1      ----------------------------------EIRGKPRLVFNYKRLN
066 YP_009508551.1   ---------------------------------------HQPTNTSIHTE
067 YP_001497148.1   ---------------------------------------HQKGQDP-IAT
068 NP_041186.1      ----------------------------------------HSTLPGALVQ
069 NP_663784.1      ---------------------------------------------QEVDH
070 YP_009508443.1   ----------------------------------ETKGKERMVFNYRRLN
071 YP_003987465.1   ----------------------------------EKRGKERLVFNYKRLN
072 NP_861410.1      ------------------------------------RKKRRMVINYKALN
073 YP_009345071.1   ----------------------------------ETKGKERMVFNYQRLN
074 YP_009508582.1   ---------------------------------------HQPSHTSIHTQ
075 NP_569141.1      ------------------------------------RGKSRMVIDYRNLN
076 YP_002916057.1   ----------------------------------EVRGKQRMVLNYKRLN
077 NP_777384.2      ----------------------------------------HSSASHPIAS
078 YP_233110.1      ----------------------------------EKRGKERLVFNYKRLN
079 NP_542258.1      ----------------------------------------HTLLQDPISR
080 YP_009130664.1   ----------------------------------EVRGKERMVFNYKRLN
081 YP_009508566.1   ---------------------------------------HQPSSTSVHTQ
082 YP_006607892.1   ------------------------------------RGKKRMVVNYKKIN
083 YP_009506270.1   ----------------------------------EKRGKERLVFNYKRLN
084 NP_042513.1      ------------------------------------RGKRRLVINYK-MN
085 YP_006907834.1   ------------------------------------RGKKRMVVNYKAMN
086 YP_004442827.1   ----------------------------------EVKGKPRLVFNYKRLN
087 YP_009408594.1   ----------------------------------EQKGKERMVFNYRTLN
088 YP_009508561.1   ---------------------------------------HQPSKTSIHTL
089 NP_056907.1      ----------------------------------------HTNLPDPIST
090 YP_002308474.1   ----------------------------------------HTLLQDPISR
091 YP_007761644.1   ------------------------------------RGKARMVINYKPLN
092 NP_040550.1      ----------------------------------------HSEVPGFFTE
093 NP_758808.1      ----------------------------------EIKGKERMVFNYKALN
094 YP_009345075.1   ----------------------------------FRHGKERMVFDYRKLN
095 NP_068729.1      ------------------------------------RGKRRMVINYKKMN
```

```
096 NP_047255.1       ---------------------------------------HQKGDSP-QAK
097 YP_006273075.1    ----------------------------------EIRKKERMVFNYKRLN
098 YP_009508888.1    ---------------------------------------HQPSHTSVHTE
099 NP_395469.1       ------------------------------------RAKRRIVINYKAMN
100 YP_009508546.1    ---------------------------------------HQPMNTSIHTE
101 NP_056762.1       --------------------------------------KPRIVYNYKRLN
102 YP_009508556.1    ---------------------------------------HQQPMTTLHTE
103 NP_569153.1       ----------------------------------EVKGKERMVFNYKRLN
104 NP_043924.1       --------------------------------------IWQDPPVFKLHD
105 YP_009243641.1    ----------------------------------------HSKLPGPLVY
106 YP_004300274.1    ------------------------------------RGKSRMVIDYRNLN
107 NP_056848.1       ------------------------------------RGKTRMVIDYKDLN
108 YP_006495799.1    ----------------------------------EIRGKERMVFDYRALN
109 YP_009508409.1    --------------------------------------------------
110 NP_687035.1       ---------------------------------------------TEIDK
111 NP_045937.2       -----------------------------------NAAADEAAKNAVFLV
112 NP_612577.1       ------------------------------------RGKKRMVVNYKAMN
113 NP_619548.1       ------------------------------------RGKKRMVVNYKAIN
114 NP_056880.1       ----------------------------------------HTGLPGPLAQ
115 YP_009121747.1    ----------------------------------ETKGXXRMVFNYRTLN
116 YP_009113237.2    ----------------------------------ETKGKERMVFNYKRLN
117 YP_009508408.1    ----------------------------------EVKGKERLVFNYKRLN
118 NP_955579.1       ---------------------------------------HQKGDSP-QAK
119 YP_009229919.1    ----------------------------------EVKGKERMVFNYKTLN
120 YP_004442833.1    ----------------------------------ETKGKPRLVFNYKRLN
121 NP_862833.2       ----------------------------------------HSTLPGALVR
122 NP_040563.1       LEQMAPCSPPEAILLKPGEKQNLETGIYMQGLRPQSFLPRADLPVAITGT
123 YP_443922.1       ---------------------------------------HQVLNSR-ISK
124 NP_057933.2       ---------------------------------------HQKGHSA-EAR
125 YP_009116631.1    ----------------------------------EVRGKERMVFDYRSLN
126 YP_009513249.1    ---------------------------------------HQKLDSSPHAY
127 YP_223871.1       ---------------------------------------HQKDDAP-TST
128 NP_127504.1       ------------------------------------RGKLRLVINYQPLN
129 NP_044929.1       ---------------------------------------HQPAEASPHAQ
130 NP_057860.1       ----------------------------------------HTNLPDPISR
131 NP_040973.1       AKEIHLLPGEVKVIPTGVKLMLPKGYWGLIIGKSSIGSKGLDVLGGVIDE
132 NP_777317.1       ----------------------------------EKRGKERLVFNYKRLN
133 NP_054716.1       ---------------------------------------HQNDLQSPYAM
134 YP_001856242.1    ---------------------------------------------EQVDK
135 NP_954565.2       ----------------------------------------HSGLPGPIAQ
136 NP_569150.1       ----------------------------------ERRGKERLVFNYKRLN
137 NP_659397.1       ------------------------------------RGKKRMVVNYKKLN
138 YP_009513211.1    ---------------------------------------HQRGTDP-VAT
139 YP_233107.1       ----------------------------------EKKGKERLVFNYKRLN
140 YP_009109692.1    ---------------------------------------HQKGQDP-VAV
141 NP_057849.4       ---------------------------------------------EQVDK
142 NP_049560.1       ----------------------------------------HTNLPDPITR
143 YP_009506267.1    ----------------------------------EKKGKERMVFNYKRLN
144 NP_039820.1       ----------------------------------EKKGKERMVFNYKLLN
145 NP_056790.1       ---------------------------------------HQRGSNP-VAT
146 YP_002321513.1    ----------------------------------EKRGKERMVFNYKTLN
147 NP_955591.1       ---------------------------------------HQKGHSA-EAR
148 YP_001956722.2    ---------------------------------------HQPTASTFHTE
149 NP_056902.1       PYDIMIPVSDTKIIPTDVKIQVPPNSFGWVTGKSSMAKQGLLINGGIIDE
150 NP_056886.1       ----------------------------------------HSEVPGFFTE
151 NP_041261.1       ----------------------------------------HLNLPGPLAE
```

```
001 YP_004442836.1    DNTEKDQYSLPGINTIIKRIGNAKIYSKF---------------------
002 NP_056728.1       KATVGDAYNLPNKDELLTLIRGKKIFSSF---------------------
003 YP_009508537.1    GNNLADQLATQASFKVHTTK-NPKLDIEQIKAIQACQNNEKLPVGYPKQY
004 YP_004732983.2    DNCHKDQYSLPGITSIIKSLGQAKIFSKF---------------------
005 YP_009508407.1    SQASPVAASDELLDKVITKLGRLSIADKL---------------------
006 NP_040939.1       GYQGQIQVIMYNSNKIAVVIPQGRKFAQLILMDKKHGKLEPWG-------
007 YP_003864102.1    GNQLADLATKTIAF------------------------------------
008 YP_009002585.1    DLTEKDQYSLPGINTIMKRVGHAKIYSKF---------------------
009 YP_003284237.1    DNTEKDQYSLPGINTIIQRIGRSKIYSKF---------------------
010 YP_009268869.1    GYQGIIQVVVYNSNDKEVVIIPQGRKFAQLILMPLIHEDLEAWG------
011 YP_009506251.1    DNTEKDQYSLPGIHTILKRVGNKKIFSKF---------------------
012 NP_955611.1       GNDVADSQATFQA-------------------------------------
013 YP_008567619.1    DNTNKDQYSLPGINTIISKVGNSKVFSKF---------------------
014 NP_149413.1       DNTWPDQYSLPGINALLKNVARAKIFSKF---------------------
```

```
015 YP_004442824.1  DNTEKDQYSLPGINTIIQRIGNAKIYSKF---------------------
016 YP_001931967.1  DATIGDAHNIPNRDSLMALISGKRIFSSF---------------------
017 YP_002455786.1  LNEYTDSLIVAPV-------------------------------------
018 YP_009109694.1  GNRQADRVARQAAMAEVLTLATEPDNTS--HIT-----------------
019 NP_789739.1     --------------------------------------------------
020 NP_955619.1     LNALTDALLITPV-------------------------------------
021 YP_224289.1     DNTEKDQYSLPGINTIISRIGNAKVYSKF---------------------
022 YP_009182100.1  RVTKDDGYFLPNLETLVYQVAEARVFSKF---------------------
023 NP_705927.1     LVSAGIRKVL----------------------------------------
024 YP_009508577.1  GNNLADKLATQGSYTVNNIVKNPSLDAELEQLINGHSVK-----GYPSRY
025 YP_004442830.1  DNTEKDQYSLPGINGLLKSVGNAKIYSKF---------------------
026 NP_758887.1     LVSRGVRQVLF---------------------------------------
027 YP_009508406.1  --------------------------------------------------
028 YP_610965.1     DNTHKDQYSLPGINTIIQKIGRATVYSKF---------------------
029 YP_001931961.1  TATIGDAHNLPNKDELLTLIRGKSIFSSF---------------------
030 YP_241114.1     GNHTADALTKQVF-------------------------------------
031 YP_006732334.1  EVTIGDSHNLPNMQELITLLRGKSIFSSF---------------------
032 YP_009041481.1  NNTHKDQYSLPGINTIIQKVGRAKVYSKF---------------------
033 YP_001036293.1  DNTEKDQYSLPGINTILKRVGQSKIYSKF---------------------
034 YP_605811.1     DNTEKDQYSLPGINTIISRIGKSKIYSKF---------------------
035 NP_955577.1     GNRLADDTAKKAATETHSSLTVLPTELIEGPKRPPWEYDDSDL-----DL
036 YP_003208050.1  DNTHKDQYSLPGINTILKKIGTSKVYSKF---------------------
037 NP_789740.1     LVSAGIRKVLF---------------------------------------
038 YP_004442839.1  DNTEKDQYSLPGINTIISRIGNSKIYSKF---------------------
039 YP_009507791.1  GYRGALNVILSNLTRHGVKISKGQKIAQLIILPYVTESLEKGK-------
040 YP_004581513.1  DNTEKDQYSLPGINTIVSRISGKKIFSKF---------------------
041 NP_043933.1     DHTKGDGYLLPNKEQLLQRIGGKTFYSSF---------------------
042 YP_009507248.1  IIQKTFRSSSLNLVLTKVELENVKEYLRQ---------------------
043 YP_002117531.1  DNTEKDQYSLPGINTIIQRIGRSKIYSKF---------------------
044 YP_004347415.1  KKAKVIKHPIPNKDILINRGIKANYFSKF---------------------
045 NP_056891.1     GNQRADLATKIVAS------------------------------------
046 YP_009513242.1  GNNLADKLATQGSYVVHCNTT-PSLDAELDQLLQGHNPP-----GYPKQY
047 YP_009352866.1  NNTHKDQYSLPGINTIIQKVGRAKVYSKF---------------------
048 YP_009165750.1  EVTLGDSHNLPNKDELLTLIRGKTIYSSF---------------------
049 NP_040840.1     GYQGTIQVVIYNSNNKEVVIPQGRKFAQLILMPLIHEELEPWG-------
050 YP_004222728.1  GNDVADSQATFQA-------------------------------------
051 YP_009506264.1  DNTEKDQYSLPGINTIIQRIGRSKIYSKF---------------------
052 YP_567050.1     LNEYTDSLIIAPL-------------------------------------
053 YP_009508571.1  GNALADKLATQGSYVVNNNDKKPNLDAELDHLIQGKYPK-----GYPKQY
054 NP_056803.1     GNALADKLATQGSYVVNCNTKKPNLDAELDQLLQGNNVK-----GYPKQY
055 NP_955564.1     GNAYADSLTRILT-------------------------------------
056 YP_009508411.1  DNTEKDQYSLPGINTILKKIGHSKVYSKF---------------------
057 YP_009140788.1  DITHKDQYSLPGINTILKRIGNSKIFSKF---------------------
058 YP_009508410.1  KEPE---LPDEILDQITSRFNKLSIAEAP---------------------
059 NP_040333.1     GNRMADQAAREVATRETPETSTLLIENSAPYTHEHFHYTVTDI-----KD
060 YP_008992013.1  MFLQDDKFPLPRRQSMFTFLKNAQIFSKF---------------------
061 NP_041734.1     DNTEKDQYSLPGIQTILKRVGNKKVFSKF---------------------
062 NP_056895.1     LNNYVDQLLP----------------------------------------
063 YP_009109689.1  GNRQADRVARQAAMAEVLTLATEPDNTS--HITIEHTYTSEDQ-----EE
064 YP_002519387.1  AATISDGYLLPNKETILTAIRGRKYFSTL---------------------
065 YP_595725.1     DNTWPDQYSLPGINALLKNVARAKIFSKF---------------------
066 YP_009508551.1  GNALADKLATQGSYVVNCNTKKPNLDAELDQLLQGHYIK-----GYPKQY
067 YP_001497148.1  GNRQADQVARQVAVAETLTLTK-LEETN--LTTNKYAYTPEDQ-----EE
068 NP_041186.1     GNHTADVLTKQVF-------------------------------------
069 NP_663784.1     LVSQGIRQVLF---------------------------------------
070 YP_009508443.1  DNTEKDQYSLPGINTILKRIGQSVIYSKF---------------------
071 YP_003987465.1  DNTEKDQYSLPGISTILQKIGHSKIYSKF---------------------
072 NP_861410.1     KATIGNAHKLPRIDSILTKVKGSNWFSTL---------------------
073 YP_009345071.1  DNTEKDQYPLPGINTILQRIGKTKIYSKF---------------------
074 YP_009508582.1  GNNLADKLATQGSYNVNSIVKNPSLDAELEQLINGHSMK-----GYPSKY
075 NP_569141.1     AKTKTYNYPIPNKILKIRQIQGYNYFSKF---------------------
076 YP_002916057.1  DNTEKDQYSLPGINTIISRVAGKKVFSKF---------------------
077 NP_777384.2     LNNYVDQLLP----------------------------------------
078 YP_233110.1     DNTEKDQYSLPGINTIISRIGKSKIYSKF---------------------
079 NP_542258.1     LNEATDALMLAPL-------------------------------------
080 YP_009130664.1  DNTHKDQYSLPGINTIIQKVGNSKIYSKF---------------------
081 YP_009508566.1  GNALADKLAVQGSYTINNITIKPSLDTELRAVLEGKLPK-----GYPKNL
082 YP_006607892.1  EATIGDSHNLPNKDELLTLIRGKTIYSSF---------------------
083 YP_009506270.1  DNTEKDQYSLPGINTIIRRIGNAKIYSKF---------------------
084 NP_042513.1     KATKGDAYNLDR---LYLTDRESNWFSTL---------------------
085 YP_006907834.1  DATVGDAYNLPNKDELLTLIRGKKIFSSF---------------------
086 YP_004442827.1  DNTEKDQYSLPGINALLKSVGNAKIYSKF---------------------
087 YP_009408594.1  DNTYKDQYSLPGINTILKKVGNSKIYSKF---------------------
```

```
088 YP_009508561.1   GNSLADKLAVQGSYSVNTINKIPSLDAELNQILEGNLPK-----GYPKQY
089 NP_056907.1      LNEYTDSLIIAPL-------------------------------------
090 YP_002308474.1   LNEATDALMLAPL-------------------------------------
091 YP_007761644.1   LRIRKNAYRIPNKDSLFLAIRESQFYSKF---------------------
092 NP_040550.1      GNDVADSQATFQA-------------------------------------
093 NP_758808.1      DNTYKDQYSLPNIQLILKKVINSTIYSKF---------------------
094 YP_009345075.1   QNTHKDQYSLPGINTIIKLVGNSKIYSKF---------------------
095 NP_068729.1      EATIGDSYKLPRKDFILEKIKGSLWFSSL---------------------
096 NP_047255.1      GNRLADDTAKKAATETHSSLTVLPTELIEGPKRPPWEYDDSDL-----DL
097 YP_006273075.1   DITHKDQYSLPGINTILKKVGNSKIFSKF---------------------
098 YP_009508888.1   GNALADKLATQGSYVVN-NIIKPSLDAELDQVLQGNLPK-----GYPKHF
099 NP_395469.1      EATIGTPKTLPRADYIMNRLKGKIWFSTL---------------------
100 YP_009508546.1   GNALADKLATQGSYVVNCNTKKPNLDAELDQLLQGQNVK-----GYPKQY
101 NP_056762.1      DNMHTDPFNIPHKISMINLIQKANIFSKF---------------------
102 YP_009508556.1   GNNLADKLATQGSYVVHCNTT-PSLDAELDQLLQGHNPP-----GYPKQY
103 NP_569153.1      DLTNKDQYSLPGIQTILQRLKGSTIFSKF---------------------
104 NP_043924.1      ETGKVQGYVIKTTLGMKTWIMNDHLVTQS---------------------
105 YP_009243641.1   GNEQVDKLAAFAS-------------------------------------
106 YP_004300274.1   AKTKTYNYPLPNKILRVRQVQGYNWFSKF---------------------
107 NP_056848.1      KKAKVVKYPIPNKDTLIHRSIQARYYSKF---------------------
108 YP_006495799.1   NNTHKDQYTLPGINSIVAAVGNAKIYSKF---------------------
109 YP_009508409.1   --------------------------------------------------
110 NP_687035.1      LVSKGIRQILF---------------------------------------
111 NP_045937.2      QRVLKKGDALASTDLVMEYSETDEKFTAG---------------------
112 NP_612577.1      KATKGDAHNLPNKDELLTLVRGKKIYSSF---------------------
113 NP_619548.1      QATIGDSHNLPNMQELLTLLRGKSIFSSF---------------------
114 NP_056880.1      GNAYADSLTRILT-------------------------------------
115 YP_009121747.1   DNTYKDXYSLPGINTLLKRIGNAKIFSKF---------------------
116 YP_009113237.2   DLTHKDQYSLPGIQGIIARVGRAKIFSKF---------------------
117 YP_009508408.1   DNTEKDQYSLPGINTILKRVGQSEIYSKF---------------------
118 NP_955579.1      GNRLADDTAKKAATETHSSLTVL---------------------------
119 YP_009229919.1   DNTFKDQYSLPGINTILQRIGKSKIYSKF---------------------
120 YP_004442833.1   DNTEKDQYSLPGINSLLNSVGNAKIYSKF---------------------
121 NP_862833.2      GNHTADVLTKQVF-------------------------------------
122 NP_040563.1      MVDSELQLQLLNIGTEHIRIQKDEVFMT----------------------
123 YP_443922.1      GNDAADQAAKKAGGYSPGQMVLRVDPPSTELTVEHIKELQQTAGPYEHSV
124 NP_057933.2      GNRMADQAARKAAITETPDTSTLLIENSSPYTSEHFHYTVTDI-----KD
125 YP_009116631.1   DNTHKDQYTLPGINTIISAIGNAKIFSKF---------------------
126 YP_009513249.1   GNNLADQLATQASFKVHMTK-NPKLDIEQIKAIQACQNNERLPVGYPKQY
127 YP_223871.1      GNRRADEVAREVAIRPLSTQATISDAPDMPDTETP-QYSNVEE-----AL
128 NP_127504.1      HFLQDDKFPIPNKLTLFSHLSKAKLFSKF---------------------
129 NP_044929.1      GNALADKQAVSGSYKVFSNELKPSLDAELEQVLSTGRPN---PQGYPNKY
130 NP_057860.1      LNALTDALLITPV-------------------------------------
131 NP_040973.1      GYRGEIGVIMINVSRKSITLMERQKIAQLIIILPCKHEVLEQGK------
132 NP_777317.1      DNTEKDQYSLPGINTIIKRIGTSKVYSKF---------------------
133 NP_054716.1      GNNAADKLAVKASYTVFSVQTLPSLDAELHQLLDKQTPN---PKGYPSKY
134 YP_001856242.1   LVSAGIRKVL----------------------------------------
135 NP_954565.2      GNQRADLATKIVAS------------------------------------
136 NP_569150.1      DNTEKDQYSLPGINTIIKRIGNAKIYSKF---------------------
137 NP_659397.1      EVTIGDSHNLPNMQELITLLRGKNIFSSF---------------------
138 YP_009513211.1   GNRKADEAAKQAAQSTRILTETTKNQEHFEPTRGKIKPRE----------
139 YP_233107.1      DNTEKDQYSLPGINTIIARISHSKIYSKF---------------------
140 YP_009109692.1   GNRQADRVARQAAMAEVLTLATEPDNTS--HITIEHTYTSEDQ-----EE
141 NP_057849.4      LVSAGIRKVLF---------------------------------------
142 NP_049560.1      LNALTDALLITPV-------------------------------------
143 YP_009506267.1   DNTEKDQYSLPGINTILKKVGNSKIYSKF---------------------
144 NP_039820.1      ENTESDQYSLPGINTIISKVGRSKIYSKF---------------------
145 NP_056790.1      GNRRADEAAKQAALSTRVLAGTTKPQEPIEPAQEKTRPRE----------
146 YP_002321513.1   DNTYRDPYSLPGINTIIQKVGRSKIYSKF---------------------
147 NP_955591.1      GNRMADQAARKAAITETPDTSTLLI-------------------------
148 YP_001956722.2   GNNLADKLATQGSYVVNINTT-PSLDAELDQLLQGQYPK-----GFPKHY
149 NP_056902.1      GYTGEIQVICTNIGKSNIKLIEGQKFAQLIIILQHHSNSRQPWD------
150 NP_056886.1      GNDVADSQATFQA-------------------------------------
151 NP_041261.1      GNALADAATQIFP-------------------------------------


001 YP_004442836.1   ----------------DLKSGFHQVAMDPESIPWTAFWAI-DGLYEWLVM
002 NP_056728.1      ----------------DCKSGFWQVLLDQESRPLTAFTCP-QGHYEWNVV
003 YP_009508537.1   TYELRNDKCMVLR--KDGWREIPPSRERYKLIKEAHD---ISHAGREAVL
004 YP_004732983.2   ----------------DLKSGFHQVMMEEESIPWTAFISP-AGLYEWLVM
005 YP_009508407.1   ----------------PEKQGK----------------------------
006 NP_040939.1      -------ESRKTERGEKGFGSTGMYW------IENIPLAEEDHTKWHQDA
```

```
007 YP_003864102.1   ------------------------------NLNTNLQNAQDTHALHHLNA
008 YP_009002585.1   ----------------DLKSGFHQVAMHPESIKWTAFWVP-DGLYEWLVM
009 YP_003284237.1   ----------------DLKSGFHQVAMEEASIPWTAFWAI-DGLYEWLVM
010 YP_009268869.1   -------ETRRTERGNQGFGSTGAYW------IENIPLAEEDHSKWHQDA
011 YP_009506251.1   ----------------DLKSGFHQVAMAEESIPWTAFWVP-QGLYEWLAM
012 NP_955611.1      ---------------------------------Y----------------
013 YP_008567619.1   ----------------DLKSGFHQVAMDPESIEWTAFSTP-NGLYEWLVM
014 NP_149413.1      ----------------DLKSGFHQVAMDPESIPLTAFTAY-NELYEWLVM
015 YP_004442824.1   ----------------DLKSGFHQVAMDPESIPWTAFWAI-DGLYEWLVM
016 YP_001931967.1   ----------------DCKSGFWQVLLDKPSQELTAFTCP-QGHYQWLVM
017 YP_002455786.1   ----------------------------------TPLKPEGLHALTHCNQ
018 YP_009109694.1   --------------------------------------------------
019 NP_789739.1      --------------------------------------------------
020 NP_955619.1      ----------------------------------LQLSPADLHSFTHCGQ
021 YP_224289.1      ----------------DLKSGFHQVAMDPESIPWTAFLAN-NELYEWLVM
022 YP_009182100.1   ----------------DCKSGYWQIKLTPESIPLTAFSTP-KGQYEWKVL
023 NP_705927.1      --------------------------------------------------
024 YP_009508577.1   KYILKEGQVFVLR--PEGEKIIPPKSDRLALVKIAHE---FSHAGREATV
025 YP_004442830.1   ----------------DLKSGFHQVAMEEESIEWTAFWAF-TGLYEWLVM
026 NP_758887.1      --------------------------------LEGIDKAQEEHDKYHNNW
027 YP_009508406.1   --------------------------------------------------
028 YP_610965.1      ----------------DLKSGFHQVAMSPESIEWTAFIVL-GGLYEWLVM
029 YP_001931961.1   ----------------DCKSGFWQVLLDEDSQLLTAFTCP-QGHYQWIVV
030 YP_241114.1      -------------------------------FQSAIDAARKSHDLHHQNS
031 YP_006732334.1   ----------------DCKSGFWQVFLDQESQKLTAFTCP-QGHFQWRVV
032 YP_009041481.1   ----------------DLKSGFHQVAMDEASIPWTAFLVP-GGLYEWLVM
033 YP_001036293.1   ----------------DLKSGFHQVAMAPQSVEWTAFLAP-GGLYEWLVM
034 YP_605811.1      ----------------DLKSGFHQVAMDPESIPWTAFWAI-DGLYEWLVM
035 NP_955577.1      VQKLEAHYEPKRGTWEYRGKTIMPEKYAKELISHLHKLTHLSARKMKTLL
036 YP_003208050.1   ----------------DLKSGFHQVAMDEESIPWTAFCVP-GGLYEWLVM
037 NP_789740.1      --------------------------------LDGIDKAQDEHEKYHSNW
038 YP_004442839.1   ----------------DLKSGFHQVAMDPESIPWTAFWAI-DGLYEWLVM
039 YP_009507791.1   -------MIMDSQRGEGGFGSTGAYVTQISSWMDNIEKAEDDHDKFHSDV
040 YP_004581513.1   ----------------DLKAGFHQIRMEEKSKPWTAFWTP-EGLYEFEVM
041 NP_043933.1      ----------------DCKSGFWQVRLAPETIQLTAFSCP-QGHYEWLVM
042 YP_009507248.1   -----------------------------QDIIRHYHDGKTNHRGINECY
043 YP_002117531.1   ----------------DLKSGFHQVAMDPASIEWTAFWAI-DGLYEWLVM
044 YP_004347415.1   ----------------DCKSGFYHIKLEEDSKKYTAFTVP-QGYYVWIVL
045 NP_056891.1      ------------------------------NINTNLESAQNAHTLHHLNA
046 YP_009513242.1   KYTLEDNKIIVER--PNGQRIVPPKSDREKIISMAHN---IAHTGRDATF
047 YP_009352866.1   ----------------DLKSGFHQVAMDEDSIPWTAFLVP-GGLYEWLVM
048 YP_009165750.1   ----------------DCKSGFWQVLLDQESQLLTAFTCP-TGHYQWKVV
049 NP_040840.1      -------ETRKTERGEQGFGSTGMYW------IENIPLAEEEHNKWHQDA
050 YP_004222728.1   ---------------------------------YPLREAKDLHTALHIGP
051 YP_009506264.1   ----------------DLKSGFHQVAMEAESIPWTAFWAI-DGLYEWLVM
052 YP_567050.1      ----------------------------------IPLTPQDLHKLTHCNS
053 YP_009508571.1   TYYMEDGKVKVNR--PEGTKIIPPSLERAGIVQKAHN---LAHTGREATL
054 NP_056803.1      TYYLEDGKVKVSR--PEGVKIIPPQSDRQKIVLQAHN---LAHTGREATL
055 NP_955564.1      ----------------------------------ALESAQESHALHHQNA
056 YP_009508411.1   ----------------DLKSGFHQVAMHPESIEWTAFLTT-AGLFEWLVM
057 YP_009140788.1   ----------------DLKSGFHQVAMHPDSIEWTAFWVP-DGLYEWLVM
058 YP_009508410.1   ----------------SEPRGR----------------------------
059 NP_040333.1      LTKLGATYDDAKKCWVYQGKPVMPDQFTFELLDFLHQLTHLSFSKTKALL
060 YP_008992013.1   ----------------DLKSGFWQLGIEPSERYKTAFCIP-NAHFQWTVL
061 NP_041734.1      ----------------DLKSGFHQVAMAEESIPWTAFWVP-QGLYEWLVM
062 NP_056895.1      -----------------------------------LETPEQWHKLTHCNS
063 YP_009109689.1   ARAIGATENKDTRNWEKEGKIVLPQKEALAMIQQMHAWTHLGNRKLKLLI
064 YP_002519387.1   ----------------DCKSGFWQIRLNENSKPLTAFSCP-MGQYEWNVV
065 YP_595725.1      ----------------DLKSGFHQVAMDEESIPLTAFSAY-NELYEWLVM
066 YP_009508551.1   TYFLEDGKVKVSR--PEGVKIIPPQSDRQKIVLQAHN---LAHTGREATL
067 YP_001497148.1   AKAIGAILNQDTKDWEKEGKIVLPRKEALAMIQQMHAWTHLSNRKLKSLI
068 NP_041186.1      -------------------------------FQSAIDAARKSHDLHHQNS
069 NP_663784.1      --------------------------------LEKIEPAQEEHEKYHSII
070 YP_009508443.1   ----------------DLKSGFHQVAMDPDSVQWTAFWVP-DGLYEWLVM
071 YP_003987465.1   ----------------DPKSGFHQVAMHPDSVPWTAFWAI-NGLYEWLVM
072 NP_861410.1      ----------------DAKSGYWQLRLHPQSKPLTAFSCPPQKHYQWNVL
073 YP_009345071.1   ----------------DLKSGFHQVAMAEESIPWTAFAIPGKGLFEWMVM
074 YP_009508582.1   KYILKEGQVFVLR--PEGEKIIPPKSDRPALVKVAHE---FSHAGREATV
075 NP_569141.1      ----------------DCKSGFYHLKLEDESKKLTAFTVP-QGFYEWNVL
076 YP_002916057.1   ----------------DLKSGFHQIRMSKESIPWTAFWTP-DGLYEFLVM
077 NP_777384.2      -----------------------------------LETPEQWHKLTHCNS
078 YP_233110.1      ----------------DLKSGFHQVAMDPESIPWTAFWAI-DGLYEWLVM
079 NP_542258.1      ----------------------------------LPLNPTTLHQITHCNP
```

```
080 YP_009130664.1   ----------------DLKSGFHQVAMHPYSIEWTAFWVP-QGLYEWLAM
081 YP_009508566.1   KYEYNSPNLIVIR--KEGQRIIPPLSDRPKLVKQAHE---LAHTGREATL
082 YP_006607892.1   ----------------DCKSGFWQVFLDQESQLLTAFTCP-SGHYQWRVV
083 YP_009506270.1   ----------------DLKSGFHQVAMDPESIPWTAFWAI-DGLYEWLVM
084 NP_042513.1      ----------------DAKSGFLQLRLDEETKPLTAFSCPPQMHLEYNVM
085 YP_006907834.1   ----------------DCKSGFWQVRLDEESKSLTAFTCP-QGHYEWNVV
086 YP_004442827.1   ----------------DLKSGFHQVAMEEESIEWTAFWAI-TGLYEWLVM
087 YP_009408594.1   ----------------DLKSGFHQIAMEKESIPWTAFIVP-QGLYEWLVM
088 YP_009508561.1   KYVLKNNELIVQR--PEGDKIIPPKADRLPLVKTAHE---LAHTGREATL
089 NP_056907.1      ----------------------------------IPLTPQDLHKLTHCNS
090 YP_002308474.1   ----------------------------------LPLSPATLHEITHCNP
091 YP_007761644.1   ----------------DCKSGFYQVPMEQDSIQLTAFSTP-IGSYEWLVM
092 NP_040550.1      ---------------------------------YPLREAKDLHTALHIGP
093 NP_758808.1      ----------------DLKSGFHQVAMDPDSVEWTAFLVP-QGLYEWLAM
094 YP_009345075.1   ----------------DLKSGFHQVAMEESSIPWTAFWVP-DGLYEWLVM
095 NP_068729.1      ----------------DAKSGYYQLRLHENTKPLTAFSCPPQKHYEWNVL
096 NP_047255.1      VQKLEAHYEPKRGTWEYRGKTIMPEKYAKELISHLHKLTHLSARKMKTLL
097 YP_006273075.1   ----------------DLKSGFHQVAMHPDSIEWTAFWVP-DGLYEWLVM
098 YP_009508888.1   VYTLEEGKVIVKR--PEGTKIIPPLADRKLLASQAHK---LSHSGREATL
099 NP_395469.1      ----------------DVKSAYWQLRLTEESKPLTAFSYPPPKHYEWNVL
100 YP_009508546.1   TYFLEDGKVKVSR--PEGVKVIPPYSDRQKIVLQAHN---LAHTGREATL
101 NP_056762.1      ----------------DLKAGFHHMKLKDDFKDWTTFTCS-EGLYTWNVC
102 YP_009508556.1   KYTLEDNKIIVER--PAGKRIVPPKADREKIISTAHN---IAHTGRDATF
103 NP_569153.1      ----------------DLKSGFHQVAMHPDSIEWTAFWVP-SGLYEWLVM
104 NP_043924.1      ----------------EQTEGRAKLSSTEGYALAQQYHHLYGHPSEESLR
105 YP_009243641.1   --------------------------------------PEEEHRYFHNNA
106 YP_004300274.1   ----------------DCKSGFYHLKLTEESKHLSAFNVP-QGFYEFNVL
107 NP_056848.1      ----------------DCKSGFYHIKLEEDSKKYTAFTVP-QGYYQWKVL
108 YP_006495799.1   ----------------DLKAGFHQVLMEESSIPWTAFITP-VGFYEWLVM
109 YP_009508409.1   --------------------------------------------------
110 NP_687035.1      --------------------------------LDRIEEAQDDHAKYHNNW
111 NP_045937.2      --------------AELHDGVFMRGDLIVPPLEMLHAILLAIHGVSHTHK
112 NP_612577.1      ----------------DCKSGLWQVLLDKESQLLTAFTCP-QGHYQWNVV
113 NP_619548.1      ----------------DCKSGFWQVVLDEESQKLTAFTCP-QGHFQWKVV
114 NP_056880.1      ----------------------------------ALESAQESHALHHQNA
115 YP_009121747.1   ----------------DLKSGFHQVAMEEESIPWTAFLIP-GGLYEWLVM
116 YP_009113237.2   ----------------DLKSGFHQVAMHPESIPWTAFWVP-QGLYEWLVM
117 YP_009508408.1   ----------------DLKSGFHQVAMDEGSIEWTAFWVP-DGLFEWLVM
118 NP_955579.1      --------------------------------------------------
119 YP_009229919.1   ----------------DLKSGFHQVAMDEESIPWTAFITP-EGLYEWLVM
120 YP_004442833.1   ----------------DLKSGFHQVAMEEESIEWTAFWAF-TGLYEWLVM
121 NP_862833.2      -------------------------------FQSAIDAARKSHNLHHQNS
122 NP_040563.1      ------------------------------CFLENIPSATEDHERWHTSP
123 YP_443922.1      WKQKGASQGPEGLWRCHDGRLVAPANLCPELIREAHGLTHEGKLKTLQRV
124 NP_057933.2      LTKLGAIYDKTKKYWVYQGKPVMPDQFTFELLDFLHQLTHLSFSKMKALL
125 YP_009116631.1   ----------------DLKSGFHQVLMDEESIPWTAFVTP-VGFYEWKVM
126 YP_009513249.1   TYELQNNKCMVLR--KDGWREIPPSRERYKLIKEAHN---ISHAGREAVL
127 YP_223871.1      GHRLRGTKDPAGWWHLPDGRLLLPRAVGRKVLEQTHRATHLGESKLTELV
128 NP_127504.1      ----------------DLKSGFWQLGIHPNERPKTGFCIP-DRHFQWKVM
129 NP_044929.1      EYKLVNGLCYVDRRGEEGLKIIPPKADRVKLCQLAHDGPGSAHLGRSALL
130 NP_057860.1      ----------------------------------LQLSPADLHSFTHCGQ
131 NP_040973.1      -------VVMDSERGDNGYGSTGVFS----SWVDRIEEAEINHEKFHSDP
132 NP_777317.1      ----------------DLKSGFHQVAMAEESIPWTAFWAI-DGLYEWLVM
133 NP_054716.1      EYTLRDGQVYVKR--TDGEKIIPSKDDRVKILELAHKGPGSGHLGKNTMY
134 YP_001856242.1   --------------------------------------------------
135 NP_954565.2      ------------------------------NINTNLESAQNAHTLHHLNA
136 NP_569150.1      ----------------DLKSGFHQVAMDPESIPWTAFWAI-DGLYEWLVM
137 NP_659397.1      ----------------DCKSGFWQVLLDDESQKLTAFTCP-QGHYQWRVV
138 YP_009513211.1   ----------------------LTPDQGREFIQRLHQLTHLGPDKLLQLV
139 YP_233107.1      ----------------DLKSGFHQVAMEEESIPWTAFWAI-NGLYEWLVM
140 YP_009109692.1   ARAIGATENKDTRNWEKEGKIVLPQKEALAMIQQMHAWTHLGNRKLKLLI
141 NP_057849.4      --------------------------------LDGIDKAQDEHEKYHSNW
142 NP_049560.1      ----------------------------------LQLSPAELHSLTHCGQ
143 YP_009506267.1   ----------------DLKSGFHQVAMHPDSIQWTAFCVP-GGLYEWLVM
144 NP_039820.1      ----------------DLKSGFWQVAMEEESVPWTAFLAG-NKLYEWLVM
145 NP_056790.1      ----------------------LTPDRGKEFIKRLHQLTHLGPEKLLQLV
146 YP_002321513.1   ----------------DLKSGFHQVAMDPESIPWTAFLTP-QGLFEWLVM
147 NP_955591.1      --------------------------------------------------
148 YP_001956722.2   QYQLENGQVMVTR--PNGKRIIPPKSDRPQIILQAHN---IAHTGRDSTF
149 NP_056902.1      -------ENKISQRGDKGFGSTGVFW------VENIQEAQDEHENWHTSP
150 NP_056886.1      ---------------------------------YPLREAKDLHTALHIGP
151 NP_041261.1      ------------------------------IISDPIHEATQAHTLHHLNA
```

```
001 YP_004442836.1    PFGLKNAPAIFQRKMDNCFRG-TEKFIAVYIDDILVFSET-VQQHKEHLK
002 NP_056728.1       PFGLKQAPSIFQRHMDEAFR-VFRKFCCVYVDDILVFSNN-EEDHLLHVA
003 YP_009508537.1    LKIQENYWWPKMKKDISSFLSTCNVCKMVNPLNLKPISPQA-IVHPTKPF
004 YP_004732983.2    PFGIQNAPAIFQRKMDECFKG-TEDFIAVYIDDILVFSNS-IREHEKHLQ
005 YP_009508407.1    --------------------------LLVFKDPVLIYQEE----------
006 NP_040939.1       RSLHLEFE--IPRTAAEDIVNQCEICKEARTPAVIR---GGNKRG----V
007 YP_003864102.1    QTLRLMFK--IPREQARQIIKQCPTC--VTYSPVPH---LGVNPRGLLPN
008 YP_009002585.1    PFGLKNAPAVFQRKMDNVFKG-TEAFIAVYIDDILVFSQS-EEEHIKHIR
009 YP_003284237.1    PFGLKNAPAVFQRKMDNCFRG-TEEFIAVYIDDILIFSES-PQQHVQHLK
010 YP_009268869.1    GSLHLDFG--IPRTAAEDIVQQCEVCQENKMPSTIR---GSNRRG----I
011 YP_009506251.1    PFGLKNAPAIFQRKMDVCFKG-TEDFIAVYIDDILVFSET-MEEHENHIS
012 NP_955611.1       --------------------------------------------------
013 YP_008567619.1    PFGLKNAPAVFQRKMDNCFKG-MEDFIAVYIDDILVFSEN-MRDHAQHLV
014 NP_149413.1       PFGLKNAPAVFQRKMDDCFRG-TERFIAVYIDDILVFSET-EEKHAEHLW
015 YP_004442824.1    PFGLKNAPAIFQRKMDHCFH---EDFIAVYIDDILVFSET-VQQHRQHLK
016 YP_001931967.1    PFGLKQAPAIFQRHMQIALN-EHSAYSCVYIDDILVFSEN-EKDHEIHVS
017 YP_002455786.1    QALVSHG---ATPAQAKQLVQACRTCQIINPQHHMP---RGHIRRGHFPN
018 YP_009109694.1    --------------------------------------------------
019 NP_789739.1       --------------------------------------------------
020 NP_955619.1       TALTLQG---ATTTEASNILRSCHACRKNNPQHQMP---QGHIRRGLLPN
021 YP_224289.1       PFGLKNAPAIFQRKMDTCFKG-TEAFIAVYIDDILVFSET-EQLHRDHLR
022 YP_009182100.1    PFGLKNAPNIFQRRMDKIFKDCY-QFCGVYVDDILVFSKD-KEEHLLHLD
023 NP_705927.1       --------------------------------------------------
024 YP_009508577.1    LRLQDKYWWPNMRKDVISHIRTCKPCLTTDGSNLTPIPPKP-QLRPEKPF
025 YP_004442830.1    PFGLKNAPAIFQRKMDNCFRG-TEKFITVYIDDILVFSET-EEEHAIHLT
026 NP_758887.1       RALAQDFC--IPNIVAKEIVAQCPKCQTK--GEPIH---GQVDTS----P
027 YP_009508406.1    ------MSAIWENSLKAYFE------------------------------
028 YP_610965.1       PFGLKNAPAVFQRKMDHCFAG-TEKFIAVYIDDILIFSAN-DEEHVEHLK
029 YP_001931961.1    PFGLKQAPSIFQRHMNNAFR-DFASYCCVYVDDILVFSNN-IKDHYAHVA
030 YP_241114.1       HSLRLQFK--ISREAARQIVKSCSTC--PQFFVLPQ---YGVNPRGLRPN
031 YP_006732334.1    PFGLKQAPSIFQRHMQNALR-GLEDYCLVYVDDIIVFSNS-EKEHYDHVL
032 YP_009041481.1    PFGLRNAPAIFQRKMDEVFAD-LKEFVSVYIDDILVFSET-YEEHAAHLK
033 YP_001036293.1    PFGLKNAPAVFQRKMDNVFRG-TEEFIAVYIDDILVFSET-EEEHLKHLR
034 YP_605811.1       PFGLKNAPAIFQRKMDNCFRG-TEEYIAVYIDDILIFSDN-VSDHRKHLS
035 NP_955577.1       EREETGFYLPNRDLHLRQVTESCRACAQINAGKIKFGPDV--RARGRRPG
036 YP_003208050.1    PFGLKNAPSVFQRKMDDCFKG-TEAFIAVYIDDILVFSKN-EEEHQKHLQ
037 NP_789740.1       RAMASDFN--LPPVVAKEIVASCDKCQLK--GEAMH---GQVDCS----P
038 YP_004442839.1    PFGLKNAPAIFQRKMDNCFRG-TEEFIAVYIDDILIFSDN-ISDHRKHLK
039 YP_009507791.1    VYLKQRFG--IPRQVAEEIIRKCPLCMIK--GEVSY---GQLKIG----E
040 YP_004581513.1    PFGLMNAPADFQRKMDNAFRG-TDAFIAVYIDDILVFSEN-EEEHEDHLL
041 NP_043933.1       PFGLKQAPAIFQRHMDESLSNMYPQFCAVYVDDIIVFSKT-EEEHLGHVK
042 YP_009507248.1    LALSKRYYWPRMKDQITKFINECTICGQAKYDRNPIR-----PQFNIVPP
043 YP_002117531.1    PFGLKNAPATFQRKMDMCFAG-TEHFIAVYIDDILVFSET-EEDHVKHLH
044 YP_004347415.1    PFGYHNSPSIYQQFMDGIFR-PYYDFILVYIDDILIFSKT-YEEHKIHLE
045 NP_056891.1       QTLRLMFN--IPREQARQIVKQCPIC--VTYLPVPH---LGVNPRGLFPN
046 YP_009513242.1    LKVSSKYWWPNLRKDVVKVIRQCKQCLVTNAANLTSPPILR-PEKPLKPF
047 YP_009352866.1    PFGLRNAPAIFQRKMDEVFGD-LKDFIAVYIDDILVFSET-HEQHAQHIK
048 YP_009165750.1    PFGLKQAPSIFQRHMNNAFR-EFEEFCCVYVDDILVFSKN-ETEHRKHVI
049 NP_040840.1       VSLHLEFG--IPRTAAEDIVQQCDVCQENKMPSTLR---GSNKRG----I
050 YP_004222728.1    RALSKACN--ISMQQAREVVQTCPHC---NSAPALE---AGVNPRGLGPL
051 YP_009506264.1    PFGLKNAPAVFQRKMDGCFKG-TEEFIAVYIDDILVFSET-PQQHVQHLR
052 YP_567050.1       RALVSSG---ATPQQAKSLLQTCYTCNIINSQHHMP---QGHIRRGLMPN
053 YP_009508571.1    LKIANLYWWPNMRKDVVRQLGRCQQCLVTNAFNQTSGPILR-PTRPLKPF
054 NP_056803.1       LKIANLYWWPNMRKDVVKQLGRCKQCLITNASNKTSGPILR-PDRPQKPF
055 NP_955564.1       AALRFQFH--ITREQAREIVKLCPNC--PDWGHAPQ---LGVNPRGLKPR
056 YP_009508411.1    PFGLKNAPAVFQRKMDNCFRG-MEDFLAVYIDDILVFSNS-EEEHMRHLE
057 YP_009140788.1    PFGLKNAPSIFQRKMDECFNG-TEEFIAVYIDDILVFSED-EKSHAKHLR
058 YP_009508410.1    --------------------------ILVHTDPVKIFEEE----------
059 NP_040333.1       ERNYCPYYMLNRDRTLKDITETCQACAQVNASKSAVKQGT--RVRGHRPG
060 YP_008992013.1    PFGLKTAPSIFQKSMIKIFQP-LLSNALIYIDDILLFSGS-HDDHHRLLN
061 NP_041734.1       PFGLKNAPAVFQRKMDQCFKG-TEEFIAVYIDDILVFSEN-MAEHTKHIG
062 NP_056895.1       RALSRWPNPRISAWDPRSPATLCETCQRLNPTGGGK---MRTIQRGWAPN
063 YP_009109689.1    EK--TDFLIPRASTLIEQVTSACKVCQQVNAGATRVPAGK--RTRGNRPG
064 YP_002519387.1    PFGLKQAPGLFQRFMDNSFK-EYSAFCAVYVDDILVFSKT-LDEHYDHLE
065 YP_595725.1       PFGLKNAPAIFQRKMDQCFRG-TEGFIAVYIDDILVFSED-EEQHAEHLW
066 YP_009508551.1    LKIANLYWWPNMRKDVVKQLGRCQQCLITNASNKASGPILR-PDRPQKPF
067 YP_001497148.1    EK--TDFLIPKAGTLIEQVTSACKVCQQVNAGATRVPEGK--RTRGNRPG
068 NP_041186.1       HSLRLQFK--ISREAARQIVKSCSTC--PQFFVLPQ---YGVNPRGLRPN
069 NP_663784.1       KELTHKFG--IPLLVARQIVNSCAQCQQK--GEAIH---GQVNAE----I
070 YP_009508443.1    PFGLKNAPAVFQRKMDNCFRG-TEDFIAVYIDDILVFSRS-QEEHAQHLK
```

```
071 YP_003987465.1   PFGLKNAPAVFQRKMDHCFRG-TEDFIAVYIDDILVFSET-PEQHKKHLE
072 NP_861410.1      PFGLKQAPGIYQNFMDKNLE-GLENFCLAYIDDILVFTNSSREEHLSKLL
073 YP_009345071.1   PFGLKNAPSVFQRKMDLCFTG-LEKFVAVYIDDVLIFSNS-EQEHLEHLD
074 YP_009508582.1   LRLQDKYWWPNMRKDVISHIRMCKPCLTTDSSNLTPIPPKP-QQRPKKPF
075 NP_569141.1      PFGYKNAPGRYQHFMDNYFN--QLENCIVYIDDILLYSRT-QDEHIKLLE
076 YP_002916057.1   PFGLVNAPADFQRKMDNAFRG-TEAFIAVYIDDILIFSET-EEDHEQHLL
077 NP_777384.2      RALSRWPNPRISAWDPRSPATLCETCQRLNPTGGGK---MRTIQRGWAPN
078 YP_233110.1      PFGLKNAPAIFQRKMDNCFRG-TEEFIAVYIDDILIFSDN-ISDHRKHLS
079 NP_542258.1      HALRNHG---ATASEAHAIVQACHTCKVINPQGRLP---QGYIRRGHAPN
080 YP_009130664.1   PFGLKNAPAVFQRKMDNCFKG-TENFIAVYIDDILVFSET-EREYARHLE
081 YP_009508566.1   LRLQNQYWWPKMRKDVSHCLRTCMPCLQTNSTNLTTTRPFQ-QIRPSKPF
082 YP_006607892.1   PFGLKQAPSIFQRHMQNAFR-SFEKFCCVYVDDILVFSDN-EQDHHRHVM
083 YP_009506270.1   PFGLKNAPAVFQRKMDSCFRG-TEEFIAIYIDDILVFSET-VQQHKEHLK
084 NP_042513.1      PMGLKQAPSQFQRFMDNNLR-GLEDISLAYIDDIIVFTKGTKDYHLKQVA
085 YP_006907834.1   PFGMKQAPSIFQRHMDEAFK-VFRKFCCIYVDDILVFSDN-EQNHQLHVA
086 YP_004442827.1   PFGLKNAPAIFQRKMDLCFQG-TESFIAVYIDDILIFSQN-EEEHAEHLY
087 YP_009408594.1   PFGLKNAPALFQRKMDNCFKG-TEDFIAVYIDDVLVFSNN-EQDHHRHLH
088 YP_009508561.1   LKLQTTHWWPNMRKDIITVLRQCKPCLQTDSTNLTPIPPVS-QPRPVKPF
089 NP_056907.1      RALVSSG---ATPQQAKSLLQTCYTCNIINSQHHMP---QGHIRRGLMPN
090 YP_002308474.1   PALCNHG---ATATETKAIVRACHTCKITNPQGRLP---QGHIRRGHAPN
091 YP_007761644.1   PFGLATAPSIFQAKMDNVLE-DHHDYCLVYIDDIIVFSRT-LEEHKIHVI
092 NP_040550.1      RALSKACN--ISMQQAREVVQTCPHC---NSAPALE---AGVNPRGLGPL
093 NP_758808.1      PFGLKNAPAVFQRKMDAVFKG-CEKFLAVYIDDILVFSNN-EEDHAKHLV
094 YP_009345075.1   PFGLKNAPAIFQRKMDNCFKG-TEEFIAVYIDDILVFSQT-EEEHVRHLQ
095 NP_068729.1      SFGLKQAPSIYQRFMDQSLK-GLEHICLAYIDDILIFTKGSKEQHVNDVR
096 NP_047255.1      EREETGFYLPNRDLHLRQVTESCRACAQINAGKIKFGPDV--RARGRRPG
097 YP_006273075.1   PFGLKNAPSVFQRKMDECFKG-TEDFIAVYIDDILVFSEN-EKDHAKHLK
098 YP_009508888.1   LKLSNTYWWPNMRKDVVKVIGQCQQCLVTNPSNLTSGPILR-PERPTKPF
099 NP_395469.1      PMGLKQAPGIFQEFMNRSLH-NLEHICLVYVDDIIIFSEKDKNDHLSKVL
100 YP_009508546.1   LKIANLYWWPNMRKDVVKQLGRCQQCLITNASNKASGPILR-PDRPQKPF
101 NP_056762.1      PFGIANAPCAFQRFMQESFG--DLKFALLYIDDILIASNN-EKEHIEHLK
102 YP_009508556.1   LKVSSKYWWPNLRKDVVKVIRQCKQCLVTNAANLTSPPILR-PEKPLKPF
103 NP_569153.1      PFGLKNAPAIFQRKMDHCFKG-TEAFIAVYIDDILVFSKT-EQDHEKHLQ
104 NP_043924.1      KVLTKRFVWEDMGQHCKEITNTCLTCAKYKVLRAGP--PMGVGRSAEGPC
105 YP_009243641.1   GSLHHRWR--ISWKQAKDILSNCQICK-PLHLRRTS---IGVNPRGLAPD
106 YP_004300274.1   MFGYKNAPGRYQCYMDSYFS--KLENCIVYIDDILLYSKT-KDEHETLLK
107 NP_056848.1      PFGYHNSPSIFQQFMDRIFR-PYYDFIIVYIDDILVFSKT-IEEHKIHIA
108 YP_006495799.1   PFGIANAPAVFQRKMDNCFHK-LREFVAVYIDDILVFSNS-LQEHESHLR
109 YP_009508409.1   ------MSVKWEKSVQAYFE------------------------------
110 NP_687035.1      RSMVQEFG--LPNIVAKEIVAACPKCQIR--GEPKH---GQVDAS----I
111 NP_045937.2      GGIMSYFSKFWTHPKASQTIDLILGHCQICLKHNPKYKSRLQGHRPLPSR
112 NP_612577.1      PFGLKQAPSIFPKTYANSHSNQYSKYCCVYVDDILVFSNTGRKEHYIHVL
113 NP_619548.1      PFGLKQAPSIFQRHMQTALN-GADKFCMVYVDDIIVFSNS-ELDHYNHVY
114 NP_056880.1      AALRFQFH--ITREQAREIVKLCPNC--PDWGHAPQ---LGVNPRGLKPR
115 YP_009121747.1   PFGLKNAPAIFQRKMDKCFKD-TEEFIAVYIDDILVYSNS-EADHERHLK
116 YP_009113237.2   PFGLKNAPAIFQRKMDNCFMG-TEEFIVVYIDDILVFSQN-EQDHERHIR
117 YP_009508408.1   PFGLKNAPAVFQRKMDNCFRG-MEDFVAVYIDDILVFSKT-PEEHAVHLE
118 NP_955579.1      --------------------------------------------------
119 YP_009229919.1   PFGLKNAPAIFQRKMDNCFRG-TEEFIAVYIDDILVFSET-EEQHAKHLQ
120 YP_004442833.1   PFGLKNAPAIFQRKMDNCFRG-TEAFIAVYIDDILIFSKD-PEEHAKHLE
121 NP_862833.2      HSLRLQFK--ISREAARQIVKSCSTC--PQFFVLPQ---YGVNPRGLRPN
122 NP_040563.1      DILVRQFH--LPKRIAKEIVARCQECKRT-TTSPVR---GTNPRG----R
123 YP_443922.1      SYTWWHPYMKEMTDLFCDNCTICGNYNPKKPYQTPMGHYP--VPNACFQD
124 NP_057933.2      ERSHSPYYMLNRDRTLKNITETCKACAQVNASKSAVKQGT--RVRGHRPG
125 YP_009116631.1   PFGLANAPAVFQRKMDQCFAG-TSEFIAVYIDDILVFSKT-LKEHEKHLS
126 YP_009513249.1   LKIQENYWWPKMKKDISSFLSTCNVCKMVNPLNLKPISPQA-IVHPTKPF
127 YP_223871.1      RK---HYLICGIYRAARDITTRCVACAQVNPGAAPVEKGLNSRIRGAAPG
128 NP_127504.1      PFGLKTAPSLFQKAMIKIFQP-ILFSALVYIDDILLFSET-LEDHIKLLN
129 NP_044929.1      LKLQQKYWWPRMHIDASRIVLNCTVCAQTNSTNQKPRPPLV-IPHDTKPF
130 NP_057860.1      TALTLQG---ATTTEASNILRSCHACRKNNPQHQMP---QGHIRRGLLPN
131 NP_040973.1      QYLRTEFN--LPKMVAEEIRRKCPVCRIR--GEQVG---GQLKIG----P
132 NP_777317.1      PFGLKNAPAIFQRKMDECFRG-MEEFIAVYVDDILIFSNS-VQDHKRHLQ
133 NP_054716.1      IKILNKYWWPNLIKDISKYIRTCTNCIITNTDNVPNKSYIV-QEKTGLPF
134 YP_001856242.1   --------------------------------------------------
135 NP_954565.2      QTLRLMFN--IPREQARQIVKQCPIC--VTYLPVPH---LGVNPRGLFPN
136 NP_569150.1      PFGLKNAPAIFQRKMDNCFRG-TEDFIAVYIDDILVFSET-IHQHKEHLK
137 NP_659397.1      PFGLKQAPSIFQRHMQDALR-GLEEFSLVYVDDIIVFSDN-KNDHQDHVM
138 YP_009513211.1   GR--TSFHIPNLQSVVREITSKCQVCAVTNAVTTYREPGR--RQRGDRPG
139 YP_233107.1      PFGLKNAPAIFQRKMDNCFRG-TEKFIAVYIDDILIFSDS-KEAHRTHLR
140 YP_009109692.1   EK--TDFLIPRASTLIEQVTSACKVCQQVNAGATRVPAGK--RTRGNRPG
141 NP_057849.4      RAMASDFN--LPPVVAKEIVASCDKCQLK--GEAMH---GQVDCS----P
142 NP_049560.1      AALTLQG---ATTTEASNILRSCHACRKNNPQHQMP---RGHIRRGLLPN
143 YP_009506267.1   PFGLKNAPSVFQRKMDNCFRG-TENFIAVYIDDILVFSRT-PEEHAEHLL
```

```
144 NP_039820.1       PFGLKNAPAIFQRKMDNVFKG-TEKFIAVYIDDILVFSET-AEQHSQHLY
145 NP_056790.1       NR--TSLLIPNLQSAVREVTSQCQACAMTNAVTTYRETGK--RQRGDRPG
146 YP_002321513.1    PFGLKNAPAIFQRKMDNCFSR-YSDFIAVYIDDILVFSES-ERDHEKHLR
147 NP_955591.1       --------------------------------------------------
148 YP_001956722.2    LKVSSKYWWPNLRKDVVKVIRQCKQCLVTNAATLAAPPILR-PERPVKPF
149 NP_056902.1       KILARNYK--IPLTVAKQITQECPHCTKQ--GSGPA---GCVMRS----P
150 NP_056886.1       RALSKACN--ISMQQAREVVQTCPHC---NSAPALE---AGVNPRGLGPL
151 NP_041261.1       HTLRLLYK--ITREQARDIVKACKQC--VVATPVPH---LGVNPRGLVPN
```

```
001 YP_004442836.1    KFMEICERNGLVLSPTKMKIGTRQVDFLGATIG-NSKIKLQPHIIQKIAE
002 NP_056728.1       MILQKCNQHGIILSKKKAQLFKKKINFLGLEID-EGTHKPQGHILEHINK
003 YP_009508537.1    DKFYMDYIGPLPPSEGYVHVLVVVDAATGFTWLYPTKAQTSKATIKVLT-
004 YP_004732983.2    RMLSICKEHGLVLSPTKMKIAVPGIDFLGAHIR-NSRVSLQPHIIKKIAD
005 YP_009508407.1    ----------------AEKLKRR---------------------------
006 NP_040939.1       NHWQVDYT-HYEN----IILLVWVETNSGLIYAEKVKGESGQEFRIKVMH
007 YP_003864102.1    MLWQMDIT-HCSEFNNLKYIHVSIDTFSGFILATLQTGEATKHVIAHLLH
008 YP_009002585.1    VMLEKCRENGLVLSPTKMKIAQRKVEFLGAILE-AGRIQLQPHIIKKVVN
009 YP_003284237.1    KFMEICEKNGLVLSPTKMKIGVSQVDFLGATIG-QSKIRLQPHIIKKIAD
010 YP_009268869.1    DHWQVDYT-HYED----KIILVWVETNSGLIYAERVKGETGQEFRIMTIR
011 YP_009506251.1    RMLEICKRHGLVLSPNKMSIAQEEIEFLGTIIS-KGRMKLQAHVIKKIVS
012 NP_955611.1       --------------------------------------------------
013 YP_008567619.1    AMLEVCKKNGLILSPTKMKIGLGTIDFLGATIG-NSKVKLQEHIVKKILD
014 NP_149413.1       KMLQICKKNGLILSPSKYKIGVKRVDFLGSTIG-DNQLAVQEHIVSKIAD
015 YP_004442824.1    VFFDICEKNGLVLSPTKMKIGTRQIDFLGATIG-DSRIKLQPHIIKKIAD
016 YP_001931967.1    KVLNRCINLGIILSKKKSQLFKETIDFLGISID-KGTHSPKPHILENIHN
017 YP_002455786.1    HTWQGDVT-HLKHKRTRYCLHVWVDTFSGAVSCVCKKKETSSDLIKTLLH
018 YP_009109694.1    --------------------------------------------------
019 NP_789739.1       --------------------------------------------------
020 NP_955619.1       HIWQGDIT-HFKYKNTLYRLHVWVDTFSGAISATQKRKETSSEAISSLLQ
021 YP_224289.1       KFLEISKANGLILSPTKMKIGVKTIDFLGASIG-NSKIKLQPHIIKKIAD
022 YP_009182100.1    IIINKIVQNGIIIGRTKYELVRESIDFLGVTIC-KGKIKLQPHILTHLME
023 NP_705927.1       --------------------------------------------------
024 YP_009508577.1    DKFFIDYIGPLPPSHGFVYVLVVVDAATGFTWLYPTKAPSTNATITSLN-
025 YP_004442830.1    KMLEICKRNGLILSPTKMKIGVKVIDFLGSTIG-QNQLDLQPHIVQKIVD
026 NP_758887.1       GTWQMDCT-HMEG----KVIIAAVHVASRYLEAEVIPTETGKETAHFLLK
027 YP_009508406.1    -------------------GRSTEPEYLNLYTV-DKPTNKHLATNLTYLN
028 YP_610965.1       VFCAIVEKHGLILSSNKMQLGKREIDFLGATLG-NRKIKLQAHIIKKIAA
029 YP_001931961.1    QVLRKCAELGIILSKKKAQLFKCRINFLGLDID-EGTHRPQNHILEHIHK
030 YP_241114.1       HLWQTDVT-HIPQFGRLKYVHVSIDTFSNFLMASLHTGESTRHCIQHLLF
031 YP_006732334.1    SVLRRVESLGIILSEKKANLFKEKINFLGLEID-RGTHTPQNHILEHLHG
032 YP_009041481.1    RMLQRCKKFGLVLSPTKMKIATREIDFLGATIK-DGRIKLQDHIIKKIST
033 YP_001036293.1    ILLQICQQHGLVLSPTKMKIGTKTIEFLGAVIG-NRKIKLQEHIIKKIAD
034 YP_605811.1       KFLEICKANGLVLSPTKMKIGAREIDFLGATIG-NSRIKLQPHIIKKIIE
035 NP_955577.1       THWEVDFTEIKPGMYGYKYLLVFIDTFSGWAEAYPAKHETAKVVAKKLLE
036 YP_003208050.1    KFLEIVEKEGLVLSPTKMKIAVPEVEFLGAIIG-NSTIKLQPHIIRKIAD
037 NP_789740.1       GIWQLDCT-HLEG----KVILVAVHVASGYIEAEVIPAETGQETAYFLLK
038 YP_004442839.1    KFLEICKANGLVLSPTKMKIGTPEIDFLGATIG-NSKIKLQPHIIKKVIE
039 YP_009507791.1    GIWQIDCT-HLEK----LILLVCIQTTSGFVVVYNLKREDAKETGLAFLN
040 YP_004581513.1    NLAQIVRREGLILSPTKMKIGVKEVDFLGIKIQ-GNKIQLQEHILKKIGD
041 NP_043933.1       IVLNRCKALGIVLSKKKAQLCKTTINFLGLVIE-RGNLKVQSHIGLHLVA
042 YP_009507248.1    ATKPLETVHMDLFTVQNEKYITFIDVFTKYGQAYHLRDGTAISILQALLR
043 YP_002117531.1    QMLHICEKEGLVLSPTKMKIGATSIDFLGATIG-RSKIRLQPHIIKKITE
044 YP_004347415.1    IFRNIIIKHGIVLSKKKAEIGKQKIEFLGVKIE-QGGIELQPHIIDKILE
045 NP_056891.1       MIWQMDVT-HYSEFGNLKYIHVSIDTFSGFLLATLQTGETTKHVITHLLH
046 YP_009513242.1    DKFYIDYIGPLPPSNGYLHVLVVVDGMTGFVWLYPTKAPSTSATVKALN-
047 YP_009352866.1    RMLLRCKKHGLVLSPSKMKIAQKEIEFLGATLT-GGQIKLQDHIVKKIAA
048 YP_009165750.1    AILEQCKNLGIILSKKKAHLFKTKINFLGLEID-QGSHKPQNHILEHIHK
049 NP_040840.1       DHWQVDYT-HYED----KIILVWVETNSGLIYAERVKGETGQEFRVQTMK
050 YP_004222728.1    QIWQTDFT-LEPRMAPRSWLAVTVDTASSAIVVTQHGRVTSVAAQHHWAT
051 YP_009506264.1    KFLEIVKKNGLVLSPTKMKIGVSQIDFLGATIG-QSRIKLQPHIIKKVTE
052 YP_567050.1       HIWQGDVT-HYKYKRNRYCLHVWVDTFSNAVSITCKTKETSSETVSALLH
053 YP_009508571.1    DKFFIDYIGPLPPSNGYLHVLVVVDSMTGFTWLYPTKAPTTNATVKALN-
054 NP_056803.1       DKFFIDYIGPLPPSQGYLYVLVIVDGMTGFTWLYPTKAPSTSATVKSLN-
055 NP_955564.1       VLWQMDVT-HVSEFGKLKYVHVTVDTYSHFTFATARTGEATKDVLQHLAQ
056 YP_009508411.1    QMLSVCEANGLVLSPTKMKIGTSNIEFLGARIG-NHKIQLQEHIIKKIAD
057 YP_009140788.1    IMLGICKRNGLVLSPTKMKIAVQEVEFLGAQIG-NQKIKLQPHVIKKIVE
058 YP_009508410.1    ----------------KLKL------------------------------
059 NP_040333.1       THWEIDFTEVKPGLYGYKYLLVFIDTFSGWVEAFPTKKETAKVVTKKLLE
060 YP_008992013.1    QFYDIVHSHGIMLSAKKSTIATDTIEFLGMIIK-DGHYQPGKHVAQELLH
061 NP_041734.1       IMLKICQENGLVLSPSKICLAQREIEFLGTVIS-QGQMKLQAHVIKKIVN
062 NP_056895.1       HIWQADIT-HYKYKQFTYALHVFVDTYSGATHASAKRGLTTQMTIEGLLE
```

063 YP_009109689.1	VYWEIDFTEVKPHYAGYKYLLVFVDTFSGWVEAFPTRQETAHIVAKKILE
064 YP_002519387.1	TVLRKCIETGIILSKKKAEVAKTKINYLGFTIS-NGEIELQSHILENIKL
065 YP_595725.1	KMLQICKRNGLILSPSKYKIGVKKVDFLGSTIG-DNQLAVQEHIIKKIAE
066 YP_009508551.1	DKFFIDYIGPLPPSQGYLYVLVVVDGMTGFTWLYPTKAPSTSATVKSLN-
067 YP_001497148.1	VYWEIDFTEVKPHYAGYKYLLVFVDTFSGWVEAYPTRQETAHVVAKKILE
068 NP_041186.1	HLWQTDVT-HIPQFGRLKYVHVSIDTFSNFLMASLHTGESTRHCIQHLLF
069 NP_663784.1	GVWQMDYT-HLEG----KIIIVAVHVASGFIEAEVIPQESGRQTALFLLK
070 YP_009508443.1	QMLEICRRNGLVLSPTKMKIGTQVVEFLGAVIG-NRKIQLQSHIIKKIAD
071 YP_003987465.1	IFLQIARKNGLVLSPTKMKVGVQQVDFLGATIG-NSRIRLQPHIIQKVVQ
072 NP_861410.1	VVLERCKEKGLILSKKKAIIARQTIDFLGLTLQENGEIKLQPNVLEKLEL
073 YP_009345071.1	RFFERVQQHGLVLSPTKMKIGVRKVDFLGTVIS-NGLLHMQEHILKKIAA
074 YP_009508582.1	DKFFIDYIGPLPPSHGFAYVLVVVDAATGFTWLYPTKAPSTNATITSLN-
075 NP_569141.1	KFAHIIENSGISLSKTKAEIMKNQIEFLGIQID-KNGIKMQTHIVQKIIN
076 YP_002916057.1	KFAQIVEKNGLILSPTKMKIGVKSVDFLGVKIH-QNKVQLQEHILKKIGD
077 NP_777384.2	HIWQADIT-HYKYKQFTYALHVFVDTYSGATHASAKRGLTTQMTIEGLLE
078 YP_233110.1	KFLEICKANGLVLSPTKMKIGAKEIDFLGATIG-NSKIKLQPHIIKKIIE
079 NP_542258.1	VIWQGDVT-HLHYKRYKYCLLVWVDTYSGVVSVSCRRKETGSDCVASLLA
080 YP_009130664.1	IMLEICKKNGLILSNSKMKIAVSTIDFLGATIG-NRKIKLQEHIIKKIAD
081 YP_009508566.1	DKYYIDYIGPLPPSEGYSYVLVVVDSATGFCWLYPTKAPSTRATVKSLN-
082 YP_006607892.1	ALLQRCNQLGIILSKKKAQLFQEKINFLGLEID-QGSHKPQNHILEHIHK
083 YP_009506270.1	KFMEICERNGLVLSPTKMKIGTRQVDFLGATIG-NSKIKLQPHIIQKIID
084 NP_042513.1	RVLIQLGNHGVILSKEKAKIAFEEIEFLGLKILKNGFIEPQKHLLEKIAE
085 YP_006907834.1	MILQKCYQHGIILSKKKAQLFKERINFLGLEID-QGTHRPQSHILEHIQK
086 YP_004442827.1	KMMEICKKNGLILSPTKMKIGVKVVDFLGSTVG-ENHLDLQPHIVQKIVD
087 YP_009408594.1	VMLNICKQHGLILSPSKMMIAVPEVYFLGAVLG-KQKLKLQPHIIQKIAN
088 YP_009508561.1	DKFFIDYIGPLPPSHGFSYVLVIVDARTGFTWLYPTKAPSTNATINSLN-
089 NP_056907.1	HIWQGDVT-HYKYKRNRYCLHVWVDTFSNAVSITCKTKETSSETVSALLH
090 YP_002308474.1	TIWQGDVT-HLQYKKYKYCLLVWVDTYSGAVAVSCRRKETSSECVASLLA
091 YP_007761644.1	TIAKTLKKNGIVISKKKMELGLTKINFLGCEIE-NGRIILQNHVLENLSK
092 NP_040550.1	QIWQTDFT-LEPRMAPRSWLAVTVDTASSAIVVTQHGRVTSVAAQHHWAT
093 NP_758808.1	IMLQRCKEHGLVLSPTKMNIAVREVNFLGATIG-SRKVKLQENIIKKILD
094 YP_009345075.1	RFFQICQKHGLVLSKDKMAIAVPQIEFLGAIIG-NRKIKLQPHIIRKIAN
095 NP_068729.1	IVLQRIKEKGIIISKKKSKLIQQEIEYLGLKIQGNGEIDLSPHTQEKILQ
096 NP_047255.1	THWEVDFTEIKPGMYGYKYLLVFIDTFSGWAEAYPAKHETAKVVAKKLLE
097 YP_006273075.1	AMLEICKRNGLVLSPSKMKIAVQEVEFLGAQIG-NQRIRLQPHVIKKIVE
098 YP_009508888.1	DKFFIDYIGPLPPSNGYLHVLVVVDAMTGFVWLYPTKAPSANATVKALN-
099 NP_395469.1	QVLKRCEDEGIILSQPKAKIAHKEIDFFGLHIS-EGEIILQPHILEKLVL
100 YP_009508546.1	DKFFIDYIGPLPPSQGYLYVLVVVDGMTGFIWLYPTKAPSTSATVKSLN-
101 NP_056762.1	IFFNRVKEVGCVLSKKKSKMFLKEVEYLGVEIK-EGKISLQPHIVDKIKK
102 YP_009508556.1	DKFYIDYIGPLPPSNGYLHVLVVVDGMTGFVWLYPTKAPSTSATVKALN-
103 NP_569153.1	IMLAICQKNGLILSPTKMKIAQAEIEFLGAIIH-KGLIKLQPHIVQKLLT
104 NP_043924.1	QKLQVDHVGPLGPGTHGYRYLTTMVDVYTGWFWAKPCRGPTTGATIAALE
105 YP_009243641.1	ALWQMDVT-HIPEFGHLKYVHVVIDTYSRFIWATPQGGETVKHVIAHLLE
106 YP_004300274.1	KFYHIVKEAGVSLSEKKAIIGVNQIEFLGIEID-KSGVKMQNHIVTKIVQ
107 NP_056848.1	KFRDITLANGLIISKKKTELCKEKIDFLGVQIE-QGGIELQPHIINKILE
108 YP_006495799.1	QMLEVCRKNGLVLSPTKMKVAVTTVEFLGAIIG-NGKIKLQPHIVKKISE
109 YP_009508409.1	-------------------SHKPELEYLDLSSL-EKPPLKSIAHNLAYLN
110 NP_687035.1	ETWQMDCT-HLEG----KVIIVAVHVASGFIEAEVIPRETGKETAHFLLK
111 NP_045937.2	PFAHLQIDFVQMCVKKPMYALVIIDVFSKWPEIIPCNKEDAKTVCDILMK
112 NP_612577.1	NILRRCEKLGIILSKKKAQLFKEKINFLGLEID-QGTHCPQNHILEHIHK
113 NP_619548.1	AVLKIVEKYGIILSKKKANLFKEKINFLGLEID-KGTHCPQNHILENIHK
114 NP_056880.1	VLWQMDVT-HVSEFGKLKYVHVTVDTYSHFTFATARTGEATKDVLQHLAQ
115 YP_009121747.1	IMLGKCQENGLVLSPTKMKIAVPEVEFLGAIIG-RNKIKLQPHIIKKICD
116 YP_009113237.2	AMLKICKENGLILSPSKMKIGQTKVEFLGAIID-KGKIRLQPNVIKKVCD
117 YP_009508408.1	KMLAVCQRNGLVLSPTKMKIGTPTIEFLGATIG-NRKIKLQEHIIKKIAD
118 NP_955579.1	--
119 YP_009229919.1	IMLEICEKNGLVLSANKMKIAVKEVEFLGAVIC-DRKIKLQPHIIKKITS
120 YP_004442833.1	EMLRICKANGLILSPTKYKIGVKVVDFLGSTIG-ENQLHLQPHIVQKIID
121 NP_862833.2	NLWQTDVT-HIPHFGRLKYVHASIDTFSNFLMASLHTGESTRHCIQHLLF
122 NP_040563.1	FLWQMDNT-HWNK----TIIWVAVETNSGLVEAQVIPEETALQVALCILQ
123 YP_443922.1	ISIDYTDMGADQVRGGKRYLLVMVDRFSRWVEAIPTAKEDAKSVIKWLQT
124 NP_057933.2	THWEIDFTEIKPGLYGYKYLLVFIDTFSGWIEAFPTKKETAKVVTKKLLE
125 YP_009116631.1	IMLGICRDNGLVLSPSKMKLAATEIDFLGATIG-DGRIKLQPHIIKKIAE
126 YP_009513249.1	DKFYMDYIGPLPPSEGYVHVLVVVDAATGFTWLYPTKAQTSKATIKVLN-
127 YP_223871.1	EHWEADFTEMITAKGGYKYLLVLVDTFSGWVEAYPAKRETSQVVIKHLIH
128 NP_127504.1	QFISLVKKFGVMLSAKKMILAQNKIQFLGMDFA-DGTFSPAGHISLELQK
129 NP_044929.1	QVWYMDYIGPLPPSNGYQHALVIVDAGTGFTWIYPTKAQTANATVKALT-
130 NP_057860.1	HIWQGDIT-HFKYKNTLYRLHVWVDTFSGAISATQKRKETSSEAISSLLQ
131 NP_040973.1	GIWQMDCT-HFDG----KIILVGIHVESGYIWAQIISQETADCTVKAVLQ
132 NP_777317.1	RFFEVCTKEGLVLSPTKMKIGVREVDFLGATIG-NSKIKLQPHIITKIID
133 NP_054716.1	QKYYMDYIGPLPPSDGYYHVLVIVDEGTGYTWLYPTKAQTANATVKALN-
134 YP_001856242.1	--
135 NP_954565.2	MIWQMDVT-HYSEFGNLKYIHVSIDTFSGFLLATLQTGETTKHVITHLLH

```
136 NP_569150.1          KFMTICEKNGLVLSPTKMKIGTRQIDFLGATIG-NSKIKLQPHIIKKIIE
137 NP_659397.1          KVLRRIESLGIILSKKKANLFKEKINFLGLEID-RGTHTPQNHILDHIHT
138 YP_009513211.1       VYWEVDFTEVKPGRYGNRYLLVFIDTFSGWVEAFPTKTETALTVCKKILE
139 YP_233107.1          QFITICEENGLVLSPTKMKIGVQQVDFLGATIG-DSKVRLQPHIVKKVLE
140 YP_009109692.1       VYWEIDFTEVKPHYAGYKYLLVFVDTFSGWVEAFPTRQETAHIVAKKILE
141 NP_057849.4          GIWQLDCT-HLEG----KVILVAVHVASGYIEAEVIPAETGQETAYFLLK
142 NP_049560.1          HIWQGDIT-HFKYKNTLYRLHVWVDTFSGAISATQKKKETSSEAISSLLQ
143 YP_009506267.1       KMLSICKEHGLVLSPTKMKIGTPTVEFLGATIG-NSKIKLQEHIIKKIAD
144 NP_039820.1          TMLQLCKENGLIILSPTKMKIGTPEIDFLGASLG-CTKIKLQPHIISKICD
145 NP_056790.1          VYWEVDFTEIKPGRYGNKYLLVFIDTFSGWVEAFPTKTETALIVCKKILE
146 YP_002321513.1       VMLQVCQENGLVLSPTKMKVAVKTIEFLGAILG-NQCVQLQPHIIQKIVD
147 NP_955591.1          --------------------------------------------------
148 YP_001956722.2       DKFFIDYIGPLPPSNGYLHVLVVVDSMTGFVWLYPTKAPSTSATVKALN-
149 NP_056902.1          NHWQADCT-HLDN----KIILTFVESNSGYIHATLLSKENALCTSLAILE
150 NP_056886.1          QIWQTDFT-LEPRMAPRSWLAVTVDTASSAIVVTQHGRVTSVAVQHHWAT
151 NP_041261.1          AIWQMDVT-HFTPFGKQRFVHVTVDTFSGFILATPQTGEASKNVISHVIH
```

```
001 YP_004442836.1       TKDEELKEFRGLRRWLGILNYARS--YIPNLGKILGPLYSKTNPNGE--R
002 NP_056728.1          FP-DTLEDKKQLQRFLGILTYASD--YIPKLAQIRKPLQAKLKENVP--W
003 YP_009508537.1       -HLTGLAIPKVLHSDQGSAFTSEE------FAQWAKERNIQLEFSTP---
004 YP_004732983.2       KKDDELMTLKGLRSWLGVINYVRQ--YIPKCGTLLGPLYAKTSEHGD--R
005 YP_009508407.1       --------------------------------------------------
006 NP_040939.1          -WYALFG-PESLQSDNGPAFAAEP------TQLLMQYLGVKHTTGIP---
007 YP_003864102.1       -CFSILGPPRQLKTDNGPGYTSRN------FHDFCSKLNIKHTTGIP---
008 YP_009002585.1       FKDEDLQETKGLRSFLGLLNYARA--YIPQMGRLLSPLYAKVSPKGE--K
009 YP_003284237.1       FEDEKLKETKGLRSWLGILNYARN--YIPNLGKTLGPLYSKVSPTGE--K
010 YP_009268869.1       -WYGLFA-PKSLQSDNGPAFVAEP------TQLLMKYLGITHTTGIP---
011 YP_009506251.1       KAQMELSTTKGLRSFLGLLNYARI--YIPNLGRKLSPLYAKVSPTGE--K
012 NP_955611.1          --------------------------------------------------
013 YP_008567619.1       FNTDGLEDKKNLRSWLGILNYARA--YIPNLGRILGPLYAKVSPTGE--R
014 NP_149413.1          FDEERLKTKEGLKSWLATLNYARN--HIKDMGKLLGPLYPKTSEKGA--K
015 YP_004442824.1       IKDEELKEVRGLRKWLGILNYARN--YIPNLGKTLGPLYSKTSPNGE--R
016 YP_001931967.1       FP-ERFKDVNQCRKFLGIITYAMR--YIPELSRKRMFLQDKLKKNVP--W
017 YP_002455786.1       -AISVLGKPFSVNTDNGPAYLSQE------FHEFCTTLCIKHSTHIP---
018 YP_009109694.1       --------------------------------------------------
019 NP_789739.1          --------------------------------------------------
020 NP_955619.1          -AIAYLGKPSYINTDNGPAYISQD------FLNMCTSLAIRHTTHVP---
021 YP_224289.1          FDDHRLKETKGLRAWLGILNYARN--YIPNLGKTLGPLYSKISPNGE--K
022 YP_009182100.1       FP-DKLKDRLQVQQFLGCVNYAGK--FIQRLAELCSVLHTKLKKDHK--W
023 NP_705927.1          --------------------------------------------------
024 YP_009508577.1       -ILLGTAVPRVLHSDQGSAFTSST------FADWAKEKGIQLEFSTP---
025 YP_004442830.1       FSEEKLKTKEGLKSWLAILNYARG--HIKNLGRTLGPLYPKTSEKGE--R
026 NP_758887.1          -LAGRWP-VKHLHTDNGPNFTSEK------VATVCWWAQIEHTTGIP---
027 YP_009508406.1       HQ-EKLFSKVSLKHFYQLHEEIQQ--LKAENLKLRKDLVALTREVVE---
028 YP_610965.1          FPEAQLAEKKGLRSWLGILNYARS--YIPRLGILLGPLYQKTSPHGD--K
029 YP_001931961.1       FP-NKIEDKKQLQRFLGILTYASD--YIPQLASMRAPLQEKLKEDVP--W
030 YP_241114.1          -CFSTSGIPQTLKTDNGPGYTSRS------FQRFCLSFQIQHKTGIP---
031 YP_006732334.1       FP-DRLEDKKQLQRFLGVLTYADS--YIPKLAEKRKPLQVKLKKDQV--W
032 YP_009041481.1       VDEKSLETTKGLRSWLGIINYARG--YIPNCGTLLGPLYSKVGLHGD--K
033 YP_001036293.1       FSDDELKTTKGLRSWLGILNYARA--YMPNMGRILGPLYSKVSPNGE--K
034 YP_605811.1          TKDEELKETKGLRKWLGVLNYARA--YIPNLGKTLGPLYSKTSVNGE--K
035 NP_955577.1          EIFPRYGIPQVLGSDNGPAFISQV------SQSVATLLGINWKLHCA---
036 YP_003208050.1       IPEEQLKEKKGLRGWLGILNYART--YIPNLSTLLGPLYQKTSPHGD--K
037 NP_789740.1          -LAGRWP-VKTIHTDNGSNFTGAT------VRAACWWAGIKQEFGIP---
038 YP_004442839.1       TRDEELKEVKGLRKWLGVLNYARA--YIPNLGKTLGPLYSKTSGNGE--R
039 YP_009507791.1       -LFSQYY-VTEIQTDNGPNFANER------ITGICFHFGIKHKKGIP---
040 YP_004581513.1       FKEKDLLTKKGLRSWLGILNYARQ--YIPNLGKLLGPLYGKTSPTGE--I
041 NP_043933.1          FP-DQLSDRNALQRFLGLLNYISA--YFPKIANLRSPLQVKLKKEIT--W
042 YP_009507248.1       -FCTHHGLPITIVTDNGTEFSNQL------FSEFVRIHKIIHHKTLP---
043 YP_002117531.1       AKEEELQTTKGLRAWLGILNYARS--YIPNLGKTLGPLYSKVSPTGE--K
044 YP_004347415.1       KH-IKIKSKKELQSILGLVNQIRN--FLPNLSKILLPIQKKLKIKNEEVW
045 NP_056891.1          -CFSIIGLPKQIKTDNGPGYTSKN------FQEFCSTLQIKHITGIP---
046 YP_009513242.1       -MLTSIAIPKVLHSDQGAAFTSST------FADWAKEKGIQLEFSTP---
047 YP_009352866.1       VDDKSLQTTKGLRSWLGIINYTRA--YIPNCGTLLGPLYSKVGLHGD--K
048 YP_009165750.1       FP-DRLEDKKQLQRFLGILTYASD--YIPKLAQIRKPFQAKLKKDVT--W
049 NP_040840.1          -WYAMFA-PKSLQSDNGPAFVAES------TQLLMKYLGIEHTTGIP---
050 YP_004222728.1       -AIAVLGRPKAIKTDNGSCFTSKS------TREWLARWGIAHTTGIP---
051 YP_009506264.1       FQEEKLNNTKGLRQFLGILNYARN--YIPNLGKTLGPLYSKISPKGE--K
052 YP_567050.1          -AITILGKPLSINTDNGSAFLSQE------FQAFCASWHIKHSTHVP---
053 YP_009508571.1       -VLTSIAVPKVIHSDQGAAFTSST------FADWAKERGIQLEFSTP---
054 NP_056803.1          -VLTSIAIPKVIHSDQGAAFTSST------FAEWAKERGIHLEFSTP---
```

055 NP_955564.1 -SFAYMGIPQKIKTDNAPAYVSRS------IQEFLARWKISHVTGIP---
056 YP_009508411.1 FPESELMTTKGMRAWLGILNYARS--YIPNLGKTLGPLYSKVSPNGE--K
057 YP_009140788.1 FNEAELKEKKGMRSWLGILNYARA--YIPNLGRLLSPLYAKTSPTGD--K
058 YP_009508410.1 --
059 NP_040333.1 EIFPRFGMPQVLGTDNGPAFVSKV------SQTVADLLGVDWKLHCA---
060 YP_008992013.1 FP-DQQLSKKQVQQFLGIINYIRD--FIPHVDHYTRHLSALLKKKPP---
061 NP_041734.1 KANIELETTKGLRSFLGLLNYARI--YIPNLGRKLSPLYAKTSPTGE--K
062 NP_056895.1 -AIVHLGRPKKLNTDQGANYTSKT------FVRFCQQFGISLSHHVP---
063 YP_009109689.1 EIFPRFGLPKVIGSDNGPAFVSQV------SQGLARILGINWKLHCA---
064 YP_002519387.1 FP-SRIPDKKSLQRFLGILTYADQ--YIRKLAEWRKPLQRKLKKDTV--W
065 YP_595725.1 FDDEKLKTKEGLKSWLATLNYARN--HIKDMGKLLGPLYPKTSEKGE--R
066 YP_009508551.1 -VLTSIAIPKVIHSDQGAAFTSST------FAEWAKERGIHLEFSTP---
067 YP_001497148.1 EIFPRFGLPKVIGSDNGPAFVSQV------SQGLARTLGINWKLHCA---
068 NP_041186.1 -CFSTSGIPQTLKTDNGPGYTSRS------FQRFCLSFQIHHKTGIP---
069 NP_663784.1 -LASRWP-ITHLHTDNGPNFTSQE------VKMVAWWVGIEQSFGVP---
070 YP_009508443.1 FSDEEIKTTKGLRSWLGILNYARN--YIPNLGKILGPLYSKTSPSGE--K
071 YP_003987465.1 FDNKDLQTTKGLRSFLGILNYARS--YIPQMGKLLGPLYSKVSPTGE--K
072 NP_861410.1 FP-DAIEDRKQLQRFLGCLNYIADKGFLKEIAKETKNLYPKVSITNP--W
073 YP_009345071.1 FGPEQYKSKKDLRSWLGLINYARN--HIPNVGRMLGPIYAKTSPQGE--P
074 YP_009508582.1 -ILLGTAVPRVLHSDQGSAFTSSA------FADWAKEKGIQLEFSTP---
075 NP_569141.1 LD-ENIDTKKKLQSFLGIVNQVRE--YIPKLAENLKPLQKKLKKDVE--Y
076 YP_002916057.1 FREEDLLTKKGLRSWLGILNYARQ--HIPNLGKMLGPLYGKTSPTGE--I
077 NP_777384.2 -AIVHLGRPKKLNTDQGANYTSKT------FVRFCQQFGISLSHHVP---
078 YP_233110.1 TKDEELKETKGLRKWLGVLNYARA--YIPNLGKTLGPLYSKTSINGE--K
079 NP_542258.1 -AISILGKPHSINTDNGTAYLSQE------FQQFCSSLSIKHSTHIP---
080 YP_009130664.1 YPEKELMNAKGLRSWLGILNYARS--YIPYLGKQLGPLYSKVSPTGE--R
081 YP_009508566.1 -FLLGIAVPKILHSDQGSAFTSSD------FANWAKEKEITLEFSTP---
082 YP_006607892.1 FP-DKIEDKKQLQRFLGILTYASD--YIPKLAEIRKPLQSKLKKDVP--W
083 YP_009506270.1 IKDEELKEVKGLRKWLGILNYARS--YIPKLGKILGPLYSKTSPNGE--R
084 NP_042513.1 FP-DQLQDRKQIQKFLGCLNYIGEKGFFKELAKERKVLQKMLSEKLP--W
085 YP_006907834.1 FP-DIIESKLQLQRFLGVLTYASD--YIPKLAQIRKPLQAKLKENVQ--W
086 YP_004442827.1 FDEEKLKTKKGLKSWLAILNYARG--HIKNMGKILGPLYPKTSEKGE--K
087 YP_009408594.1 FKDADLQEKKGLRSWLGILNYARS--YIPNLGSKLGPLYEKTSPHGD--K
088 YP_009508561.1 -LLLGTAIPKVLHSDQGSAFTSAT------FAEWAKDRSIQLEFSTP---
089 NP_056907.1 -AITILGKPLSINTDNGSAFLSQE------FQAFCASWHIKHSTHVP---
090 YP_002308474.1 -AISILGKPHTINTDNGAAYLSQE------FQQFCTSLSIKHTTHVP---
091 YP_007761644.1 FP-SEIKDKKELQSFLGIINYAAS-HYSIEVTKLRVPLQKKLKKNYI--W
092 NP_040550.1 -AIAVLGRPKAIKTDNGSCFTSKS------TREWLARWGIAHTTGIP---
093 NP_758808.1 FDTEKLQSKKGLRSFLGILNYARN--HIPNLGKIAGPLYSKTSIYGD--I
094 YP_009345075.1 FPDDQLKEKKGLRSWLGILNYARN--YIPKLGQLLGPLYNKTSPMAD--K
095 NP_068729.1 FP-DELEDRKQIQRFLGCINYIANEGFFKNLALERKHLQKKISVKNP--W
096 NP_047255.1 EIFPRYGIPQVLGSDNGPAFISQV------SQSVATLLGINWKLHCA---
097 YP_006273075.1 FNEAELKEKKGMRSWLGILNYARA--YIPNLGRLLSPLYAKTSPTGD--K
098 YP_009508888.1 -MLTSIAVPKVIHSDQGAAFTSST------FADWAKEKGIHLEYSTP---
099 NP_395469.1 FP-DEIENRKQLQRFLGNLNYISEKGFFKDFAKYRKELQKKVSEKVP--W
100 YP_009508546.1 -VLTSIAIPKVIHSDQGAAFTSST------FAEWAKERGIHLEFSTP---
101 NP_056762.1 FDKNKLNTLKGLQAYLGLLNYARG--YIKDLSKLVGPLYKKTGKNGQ--R
102 YP_009508556.1 -MLTSIAIPKVLHSDQGAAFTSST------FADWAKEKGIQLEFSTP---
103 NP_569153.1 FTNKQLEEVKGLRSWLGLLNYARS--YIPHMGRLLSPLYAKVSPTGE--R
104 NP_043924.1 EHISIWGVPYSIQSDNGTAFTSKA------MQEWANTYGIEWKVGAI---
105 YP_009243641.1 -SFAVLGIPQTIKTDNGPAYSSNK------FKRFLTQYKIHHITGIE---
106 YP_004300274.1 CE-EVLDTKKKLQSFLGLINQVRE--YVPNIAKELLFLQKKLKKDVE--Y
107 NP_056848.1 KH-TKIKNKTELQSILGLLNQIRH--FIPHLAQILLPIQKKLKIKDEEIW
108 YP_006495799.1 VDDESLRTLKGLRSWLGIINYARN--YIPNCGTLLGPLYSKTSENGD--R
109 YP_009508409.1 YQ-EKLQSKVLLKHLYTIQEKISE--LEAENKRLRKDLVSLTKEVVE---
110 NP_687035.1 -LLARWP-VKHLHTDNGPNFTSQN------VAAVCWWGNIEHTTGIP---
111 NP_045937.2 DIIPRWGLPDQIDSDQGTHFTAKIS------QELTHSIGVAWKLHCPG--
112 NP_612577.1 FP-DRIEDKKQLQRFLGILTYASD--YIPKLASIRKPLQSKLKEDST--W
113 NP_619548.1 FP-DRLEDKKHLQRFLGVLTYAET--YIPKLAEIRKPLQVKLKKDVT--W
114 NP_056880.1 -SFAYMGIPQKIKTDNAPAYVSRS------IQEFLARWKISHVTGIP---
115 YP_009121747.1 FDEEKLKTKAGLRSFLGILNYARN--YIPRLSILLGPLYEKTNPHGD--K
116 YP_009113237.2 FKMEHLETKTGLRSWLGLLNYARP--YMPDLGKMLGPLYAKVSPNGE--R
117 YP_009508408.1 FNDEELKTTKGLRSWLGILNYARN--YIPNLGKILGPLYSKVSPSGE--K
118 NP_955579.1 --
119 YP_009229919.1 VPEDSLKEKKGLRSWLGILNYART--YIPKLGTLLGPLYEKTSPHGD--K
120 YP_004442833.1 FDEEKLKTKKGLKSWLAILNYARG--HIKNMGRILGPLYPKTSEKGE--R
121 NP_862833.2 -CFSISGIPQTLKTDNGPGYTSRS------FQRFCLSFQIHHKTGIP---
122 NP_040563.1 -LIQRYT-VLHLHSDNGPCFTAHR------IENLCKYLGITKTTGIP---
123 YP_443922.1 ELIPRYGVPRQIRSDNGSHFSNQH------LRQVEERFGIVHKFGSV---
124 NP_057933.2 EIFPRFGMPQVLGTDNGPAFVSKV------SQTVADLLGIDWKLHCA---
125 YP_009116631.1 VDDESLKTLKGLRSWLGVLNYARN--YIPKCGTLLGPLYSKTSEHGD--R
126 YP_009513249.1 -HLTGLAIPKVLHSDQGSAFTSEE------FAQWAKERNIQLEFSTP---
127 YP_223871.1 DIIPRFGLPVQIGSDNGPAFVAKV------TQQLCEALNVSWKLHCA---

128 NP_127504.1 FP-DTNLSVKQIQQFLGIVNYIRD--FIPEVTEHISPLSDMLKKKPP---
129 NP_044929.1 -HLTGTAVPKVLHSDQGPAFTSSI------LADWAKDRGIQLEHSAP---
130 NP_057860.1 -AIAYLGKPSYINTDNGPAYISQD------FLNMCTSLAIRHTTHVP---
131 NP_040973.1 -LLSAHN-VTELQTDNGPNFKNQK------MEGVLNYMGVKHKFGIP---
132 NP_777317.1 MRDEEIKETRGLRKWLGILNYARS--YIPRLGQTLGPLYSKVSPNGE--K
133 NP_054716.1 -HLTGTAIPKVLHSDQGSAFTSAT------LVAWAKDKGIQMEYSSP---
134 YP_001856242.1 --
135 NP_954565.2 -CFSIIGLPKQIKTDNGPGYTSKN------FQEFCSTLQIKHITGIP---
136 NP_569150.1 MKDEELKEVKGLRKWLGILNYARS--YIPKLGKILGPLYAKTSPNGE--R
137 NP_659397.1 FP-DRIEDKKQLQRFLGVLTYADS--YIPKLAEKRKPLQVKLKKDQV--W
138 YP_009513211.1 EILPRFGIPKVLGSDNGPAFVAQV------SQGLATQLGIDWKLHCA---
139 YP_233107.1 TKEESLSETKALRRWLGILNYARA--YIPDLGKILGPLYSKTSGKGE--R
140 YP_009109692.1 EIFPRFGLPKVIGSDNGPAFVSQV------SQGLARILGINWKLHCA---
141 NP_057849.4 -LAGRWP-VKTIHTDNGSNFTGAT------VRAACWWAGIKQEFGIP---
142 NP_049560.1 -AIAYLGKPNYINTDNGPAYISQD------FLDMCTPLAIRHTTHVP---
143 YP_009506267.1 FKEEELKTTKGLRSWLGILNYARS--YIPNLGKTLGPLYSKISPTGE--R
144 NP_039820.1 FSDEKLATPEGMRSWLGILSYARN--YIQDIGKLVQPLRQKMAPTGD--K
145 NP_056790.1 EILPRFGIPKVLGSDNGPAFVAQV------SQGLATQLGINWKLHCA---
146 YP_002321513.1 FDEEHLQTKQGIRSWLGILNYART--YIPNIGTLLAPFYEKTSPHGD--K
147 NP_955591.1 --
148 YP_001956722.2 -MLTSIAVPKVIHSDQGAAFTSAT------FADWAKNKGIQLEFSTP---
149 NP_056902.1 -WARLFS-PKSLHTDNGTNFVAEP------VVNLLKFLKIAHTTGIP---
150 NP_056886.1 -AIAVLGRPKAIKTDNGSCFTSKS------TREWLARWGIAHTTGIP---
151 NP_041261.1 -CLATIGKPHTIKTDNGPGYTGKN------FQDFCQKLQIKHVTGIP---

001 YP_004442836.1 RMNRQDWVIVDKIKDLVKN-LPELELPPNNAMM----IIETDGCMEGWGG
002 NP_056728.1 RWTKEDTLYMQKVKKNLQG-FPPLHHPLPEEKL----IIETDASDDYWGG
003 YP_009508537.1 -YHPQSSGKVERKNSEIKKLLTKLLVGRPLKWYNL--ISSVQLALNNTHV
004 YP_004732983.2 RWHPKDWEIVRQIKKMVQS-LPDLELPPPHAVI----IIESDGCMEGWGG
005 YP_009508407.1 --
006 NP_040939.1 -WNPQSQAIVERAHQLLKSTLKKFQPQFV---------------AVESAI
007 YP_003864102.1 -YNPQGQGIVERAHLSLKTTIDKIKKGEW----------YPTKGTPRNIL
008 YP_009002585.1 KLNKEDWKLVRQIKEQVQQ-LPDLELPPQECFI----ILETDGCMEGWGG
009 YP_003284237.1 RMNHQDWVLINKVKRQVKE-LEDMELPPVEACI----VLETDGCMEGWGG
010 YP_009268869.1 -WNPQSQALVERTHQTLKNTIEKFVSMFA---------------SFDSAI
011 YP_009506251.1 KLNKQDWNLINEIKQMVQK-LPDLDIPPIESCI----VIESDGCMEGWGG
012 NP_955611.1 --
013 YP_008567619.1 KMNQQDWGIVAQIKKIIQE-LPELELPPEDCCI----VIETDGCMSGWGG
014 NP_149413.1 GLNSEDWKLISRIKTMVRS-LPSLTIPPEDAYI----IIETDACATGWGA
015 YP_004442824.1 RMNSQDWAIVRKIKEQVLN-LPDMELPPKEAVM----IIETDGCMEGWGG
016 YP_001931967.1 TWTSEDTRLLQKLKLSLKE-FPKLHIPQPGQQL----ILETDASQKYWGG
017 YP_002455786.1 -YNPTSSGLVERTNGILKTLLYKYFLDHP-------------DLPLESAV
018 YP_009109694.1 --
019 NP_789739.1 --
020 NP_955619.1 -YNPTSSGLVERSNGILKTLLYKYFTDKP-------------DLPMDNAL
021 YP_224289.1 RMNAQDWALVTQVKRQVQN-LPELELPPEKCKM----VIETDGCMEGWGG
022 YP_009182100.1 KFTEDDVKRVQLIKKVCAN-LPELTLPPPTWKI----VLQTDASDHHWAG
023 NP_705927.1 --
024 YP_009508577.1 -YHPQSSGMVERKNREIKRLITKLLVGRPTKWYPL--LPTIQLALNNTYS
025 YP_004442830.1 SLNSEDWRIIRRMKQEVQK-LPKLSIPPQDAYI----VIETDGSMNGWGG
026 NP_758887.1 -YNPQSQGVIEAKNHHLKQIIGQVRDQAE---------------KLETAV
027 YP_009508406.1 NRPLTEKKVQDLILKITEQ--PKEIEQQAVRLS------EDLKIKLERVE
028 YP_610965.1 RMKPHDWELVAQIKQMVQS-LPDLEVPPKDSFI----VLETDGCMEGWGG
029 YP_001931961.1 NWKHSDTEYVEEIKKSLTD-FPKLHHPATDEKL----IIECDASGKYWGG
030 YP_241114.1 -YNPQGQGIVERAHQRLKHQLLKQKKGNE----------LYSP-SPHNVL
031 YP_006732334.1 SWSQSDTDYIKKIKKGLIN-FPKLYLPKPEDSL----IIETDASDNFWGG
032 YP_009041481.1 RWKSSDWQLVRKVKAAVEN-LPELEIPPKNCHI----IIETDGCMEGWGG
033 YP_001036293.1 RLNAEDWRLIREVRTTIRA-LPDLELPPADSFI----VIEADGCMEGWGG
034 YP_605811.1 RMNSQDWKVVQLIKNQVQN-LPDLDIPPAGATM----VLETDGCMEGWGG
035 NP_955577.1 -YRPQSSGQVERMNRSIKETLTKLTLETGSKDWVL--LLPLVLYRVRNTP
036 YP_003208050.1 RLKAQDYALIRQIKALVQN-LPDLKIPPADSYI----VLETDGCMEGWGG
037 NP_789740.1 -YNPQSQGVVESMNKELKKIIGQVRDQAE---------------HLKTAV
038 YP_004442839.1 RMNSQDWKIVQNIKTQVQR-LPDLEIPPTSAII----ILETDGCMEGWGG
039 YP_009507791.1 -INPQSQALVENFNRTLKVWVDKFKEVTE---------------TLEAAV
040 YP_004581513.1 RFNAQDWKLVREIKRKVQQ-LPPLEIPPKDCCI----VLEADGCMEGWGA
041 NP_043933.1 SWTEKDTETVRKIKSLVKT-LPDLYNPSPEDKP----IIECDASDDHWGA
042 YP_009507248.1 -HSPSDNGNIERFHSTILEHIRILKLQHKDEPIVNLMPYAIIGYNSSIHS
043 YP_002117531.1 RMNHQDWALVKKVKDSVQN-LPDMELPPKQCCI----VIETDGCMDGWGG
044 YP_004347415.1 EWTKEDEQNIIKLKDYCKDNVIKMTYPVEEKDMNW--IIEVDASKEYYGN
045 NP_056891.1 -YNPQGQGIVERAHLSLKTTIEKIKKGEW----------YPRKGTPRNIL
046 YP_009513242.1 -YHPQSSGKVERKNSDIKRLLTKLLIGRPAKWYDL--LPVVQLALNNSYS

```
047 YP_009352866.1    RWKASDWQLVKQIKRLVQI-LPTLEIPPKDCHI----IIETDGCMEGWGG
048 YP_009165750.1    SWTDSDTSLMIKIKKGLKS-FPTLYHPKEEDNL----IIECDASDSFWGG
049 NP_040840.1       -WNPQSQALVERTHQTLKNTLEKLIPMFN---------------AFESAL
050 YP_004222728.1    -GNSQGQAMVERANRLLKDKIRVLAEGDG-----F--MKRIPASKQGELL
051 YP_009506264.1    RMNKQDWALVQQIKKQVAN-LPEMELPPRDAVM----ILETDGCMDGWGG
052 YP_567050.1       -YNSTSSGLVERTNGIVKALLNKYLLDSP-------------NLPLDNAI
053 YP_009508571.1    -YHPQSSGKVERKNSDIKRLLTKLLVGRPTKWYDL--LPVVQLALNNSYS
054 NP_056803.1       -YHPQSSGKVERKNSDIKRLLTKLLVGRPTKWYDL--LPVVQLALNNTYS
055 NP_955564.1       -YNPQGQAIVERTHQNIKAQLNKLQKAGK----------YYTP---HHLL
056 YP_009508411.1    RMNAQDWALVRKVKISIQN-LPELEIPPKNCVI----IIETDGCMEGWGG
057 YP_009140788.1    RMNSQDWKLVSQIKEEVQK-LPDLEIPPEDSFI----ILETDGCMTGWGG
058 YP_009508410.1    --------------------------------------------------
059 NP_040333.1       -YRPQSSGQVERMNRTIKETLTKLTLATGSRDWVL--LLPLALYRARNTP
060 YP_008992013.1    EWNDDHTNAVTTLKKIAQNPPPKHQTHPTKKIL------QTDASDQYWSA
061 NP_041734.1       RFNRQDWHLIKEIKDMVQK-LPNLAIPPARCYI----IIESDGCMEGWGA
062 NP_056895.1       -YNPTSSGLVERTNGLLKLLLSKYHLDEP-------------HLPMTQAL
063 YP_009109689.1    -YRPQSSGQVERMNRTIKETLTKLTLETGLKDWRR--LLSLALLRARNTP
064 YP_002519387.1    EWNDSDTQYVEKFKRNLKE-FPKLHHPLPDEYL----IIETDASHEHWAG
065 YP_595725.1       RLNSEDWKLINRIKTMVRT-LPNLTIPPEDAYI----IIETDACATGWGA
066 YP_009508551.1    -YHPQSSGKVERKNSDIKRLLTKLLVGRPTKWYDL--LPVVQLALNNTYS
067 YP_001497148.1    -YRPQSSGQVERMNRTIKETLTKLTLETGLKDWRR--LLSLALLRARNTP
068 NP_041186.1       -YNPQGQGIVERAHQRIKHQLLKQKKGNE----------LYSP-SPHNAL
069 NP_663784.1       -YNPQSQGVVEAMNHHLKNQISRIREQAN---------------TIETIV
070 YP_009508443.1    RMNAQDWELVRKVKKIVNE-LPDLIIPPEKCCI----IIETDGCMSGWGG
071 YP_003987465.1    RMNKQDWAIIEKIKQMVEQ-LPELELPPNGSVI----VIETDGCMEGWGG
072 NP_861410.1       HWSDLDSKLVNQIKKKCKD-LSPLYFPKPEDYL----IIETDASGDTWAG
073 YP_009345071.1    KMNAQDWKIIQDIQAQIHQ-LPTLEIPDKDAVI----IIETDGCMTGWGA
074 YP_009508582.1    -YHPQSSGMVERKNREIKRLITKLLVGRPTKWYPL--IPTIQLALNNTYS
075 NP_569141.1       SFDEKDKEQIKKIKILCKK-LPKLYFPDENKKFTY--IVETDSSNYSYGG
076 YP_002916057.1    RFNAQDWKLVREIKRKIQQ-LPPLEIPPKDCCI----VLEADGCMDGWGA
077 NP_777384.2       -YNPTSSGLVERTNGLLKLLLSKYHLDEP-------------HLPMTQAL
078 YP_233110.1       KMNSQDWKVVQLIKNQVQN-LPDLDIPPAEATM----VLETDGCMEGWGG
079 NP_542258.1       -YNPTSSGLVERTNGILKTLISKYLLDNH-------------HLPLETAI
080 YP_009130664.1    KMNQQDWAIVKQIKEKVQN-LPDLELPPPDCHI----VLETDGCMDGWGG
081 YP_009508566.1    -YHPQSSGKVERKNQEIKKLLTKLLVGRPAKWYPL--IPSVQLALNNTYS
082 YP_006607892.1    TWTHDDINYMSKIKKNLKS-FPTLYHPKLTDTL----IIETDASGSFWGG
083 YP_009506270.1    RMNSQDWKIVKEVKEIVAN-LPDLELPPEKAIM----IIETDGCMDGWGG
084 NP_042513.1       KWNDLATLAVKRLKQVCKN-LPRLYVAKPSDLL----ILTTDASDTTWGA
085 YP_006907834.1    RWTPEDTLYMKKVKKNLNG-FPPLHHPLPEEKL----IIETDASDNYWGG
086 YP_004442827.1    RLNSEDWKIIRKMKEEVRR-LPKMAIPPEDAYI----VIETDGSMNGWGG
087 YP_009408594.1    RFKPSDWALVKELKAQIQN-LPDLEIAPASAYI----VLETDGSMTGWGG
088 YP_009508561.1    -YHPQSSGMVERKNKEIKRLITKLLVGRPTKWYPL--LPTVQLALNNTYS
089 NP_056907.1       -YNSTSSGLVERTNGIVKALLNKYLLDSP-------------NLPLDNAI
090 YP_002308474.1    -YNPTSSGLVERTNGILKTLISKYLLDDH-------------HLPLDTAI
091 YP_007761644.1    SWTEQDKQIVEQIKTICQN-LPALELPKNGDKL----VLTTDASDKHWAG
092 NP_040550.1       -GNSQGQAMVERANRLLKDKIRVLAEGDG-----F--MKRIPTSKQGELL
093 NP_758808.1       RFSASDWKLINEIKAIVEK-LPPLDYPPEQAYI----IIESDGCMEGWGA
094 YP_009345075.1    RMTASDWNLVKKIKEKVQA-LPDMELPPKDAFV----IIETDGCTEGWGA
095 NP_068729.1       KWDTIDTKMVQSIKGKIQS-LPKLYNASIQDFL----IVETDASQHSWSG
096 NP_047255.1       -YRPQSSGQVERMNRSIKETLTKLTLETGSKDWVL--LLPLVLYRVRNTP
097 YP_006273075.1    RMNAQDWKLVAQIKEEVQK-LPDLELPPEDCFI----ILETDGCMTGWGG
098 YP_009508888.1    -YHPQSSGKVERKNSDIKRLLTKLLVGRPTKWYDL--LSTVQLALNNAYS
099 NP_395469.1       KWTSYDTTQVQALKTLCQR-LPKLYNAKESDLL----IIATDASNGHWGA
100 YP_009508546.1    -YHPQSSGKVERKNSDIKRLLTKLLVGRPTKWYDL--LPVVQLALNNTYS
101 NP_056762.1       IFNKEDWNIIFKIEREVSK-IKPLERPKETDYI----IIETDASEEGWGA
102 YP_009508556.1    -YHPQSSGKVERKNSDIKRLLTKLLIGRPAKWYDL--LPVVQLALNNSYS
103 NP_569153.1       RMNRQDWALIDKIRAQVQN-LPALELPPADCFI----IIETDGCMDGWGG
104 NP_043924.1       -YHPQSQGKVERKHRLLKDRLKRATHEGKNWVQALPSILLFINSMHPRDQ
105 YP_009243641.1    -YNPQGQAIIERAHGTLKLYIKKLKKRGERELELP--PLIALQNNPRAVL
106 YP_004300274.1    HFDSQDQEKLKKIKEKCSN-LPKLLFPDETKQFDW--IVETDASEISYGG
107 NP_056848.1       TWTKEDEEKIKLIQDYSKNLVIKMKYPINKEDMNW--IIEVDASNNAYGS
108 YP_006495799.1    RMSPQDWKLVRKVKELVKS-LPDLELPPAGAYV----VIETDGCMEGWGG
109 YP_009508409.1    NRPLTERKVQELIVNITSQ--PKEIEQQAVKLS------KDLQLKLDRVE
110 NP_687035.1       -YNPQSQGSVESMNRQLKEIISQIRDDCE---------------RLETAV
111 NP_045937.2       --HPRSSGIVERTNRTLKSKIIKAQEQLQLSKWTE--VLPYVLLEMRATP
112 NP_612577.1       TWNDTDSQYMAKIKKNLKS-FPKLYHPEPNDKL----VIETDASEEFWGG
113 NP_619548.1       NWTQSDSDYVKKIKKNLGS-FPKLYLPKPEDHL----IIETDASDSFWGG
114 NP_056880.1       -YNPQGQAIVERTHQNIKAQLNKLQKAGK----------YYTP---HHLL
115 YP_009121747.1    RMKPSDYEIVRKIKAKVQQ-LPDLQIPPDDAYI----TIETDGCMDGWGG
116 YP_009113237.2    RFNREDRELLRKLTEKIRN-LPDLELPPPNAYI----IIESDGCMTGWGG
117 YP_009508408.1    RMNGQDWELVRKVKQLVQK-LPDLEIPPKDCCI----LIETDGCMDGWGG
118 NP_955579.1       --------------------------------------------------
119 YP_009229919.1    RMKPSDWELVKKIKAQVQN-LPDMEIPPEKAHI----VIETDGSMEGWGA
```

120 YP_004442833.1 RLNSEDWKIIRGMKQEVKN-LPKLAIPPENAYI----VIETDGSMNGWGG
121 NP_862833.2 -YNPQGQGIVERAHQRLKHQLLKQKKGSD----------LYSP-SPHNAL
122 NP_040563.1 -YNPQSQGVVERAHRDLKDRLAAYQGDCE---------------TVEAAL
123 YP_443922.1 -YKPQSQGLVERCNQTLKAKIAKVCAGTKLTWVEA--LPLALMAMRSSPG
124 NP_057933.2 -YRPQSSGQVERMNRTIKETLTKLTLATGSRDWVL--LLPLALYRARNTP
125 YP_009116631.1 RWHASDWALVKRIKGLVQN-LPDLKLPTEEAYM----IIETDGCMEGWGG
126 YP_009513249.1 -YHPQSSGKVERKNSEIKKLLTKLLVGRPLKWYNL--ISSVQLALNNTHV
127 YP_223871.1 -YRPQSSGQVERMNRTLKETIAKLRKETGG-DWVS--LLPQALLRARCTP
128 NP_127504.1 AWGKCQDNAVKQLKQLAQQVKSLHIPSEGKKIL------QTDASDQYWSA
129 NP_044929.1 -YHPQSSGKVERKNSEIKRLLTKLLAGRPTKWYPL--IPIVQLALNNTPN
130 NP_057860.1 -YNPTSSGLVERSNGILKTLLYKYFTDKP-------------DLPMDNAL
131 NP_040973.1 -GNPQSQALVENVNHTLKVWIRKFLPETT---------------SLDNAL
132 NP_777317.1 RMNSQDWAIVKKIKQQVQN-LPDLELPPQEAIM----VIEADGCMEGWGG
133 NP_054716.1 -YHPQSSGKVERKNSEIKRLLTKLLVGRPTKWYPL--IPTVQLALNNTPN
134 YP_001856242.1 --
135 NP_954565.2 -YNPQGQGIVERAHLSLKTTIEKIKKGEW----------YPRKGTPRNIL
136 NP_569150.1 RMNTQDWKIVKEVKEVVAN-LPELELPPEKAIM----IIETDGCMEGWGG
137 NP_659397.1 IWTQSDTDYVKKIKKGLIN-FPKLYLPKKEDSL----IIETDASDHFWGG
138 YP_009513211.1 -YRPQSSGQVERMNRTIKETLTKLALETGGKDWVT--LLPLALLRARNTP
139 YP_233107.1 KLNHQDMKIIHQIKEKVKN-LPELEVPPPESII----LIETDGCMDGWGG
140 YP_009109692.1 -YRPQSSGQVERMNRTIKETLTKLTLETGLKDWRR--LLSLALLRARNTP
141 NP_057849.4 -YNPQSQGVVESMNKELKKIIGQVRDQAE---------------HLKTAV
142 NP_049560.1 -YNPTSSGLVERSNGILKTLLYKYFTDKP-------------DLPMDNAL
143 YP_009506267.1 RMNDQDWDLIRKIKRQIHN-LPDLEIPPEDAFI----ILEVDGCMEGWGG
144 NP_039820.1 RMNPETWKMVRQIKEKVKN-LPDLQLPPKDSFI----IIETDGCMTGWGA
145 NP_056790.1 -YRPQSSGQVERMNRTIKETLTKLALETGGKDWVT--LLPLALLRARNTP
146 YP_002321513.1 RLKPSDRELVKKIKEMVQH-LPPLELPPPDAYI----ILETDGSMEGWGG
147 NP_955591.1 --
148 YP_001956722.2 -YHPQSSGKVERKNSDIKRLLTKLLVGRPAKWYDL--LPVVQLALNNSYS
149 NP_056902.1 -YHPESQGIVERANRTLKEKIQSHRDNTQ---------------TLEAAL
150 NP_056886.1 -GNSQGQAMVERANRLLKDRIRVLAEGDG-----F--MKRIPTSKQGELL
151 NP_041261.1 -YNPQGQGVVERAHQTLKNALNRLARSPL----------GFSMQQPRNLL

001 YP_004442836.1 VCKWKEQSGQPRWSEK----------------------------------
002 NP_056728.1 MLKAIKINEGTNT-------------------------------------
003 YP_009508537.1 VSTKYTPHQLMFG-------------------------------------
004 YP_004732983.2 ICKWKNSKGESKGKER----------------------------------
005 YP_009508407.1 --
006 NP_040939.1 A---
007 YP_003864102.1 N---
008 YP_009002585.1 VCKWKKKEYDPKNTER----------------------------------
009 YP_003284237.1 VCKWKLPGQN-KASEK----------------------------------
010 YP_009268869.1 A---
011 YP_009506251.1 ICKWKKAKEDSRTTGR----------------------------------
012 NP_955611.1 --
013 YP_008567619.1 VCKWKPMACDPKNTER----------------------------------
014 NP_149413.1 VCKWKKSKADPRSSEL----------------------------------
015 YP_004442824.1 VCKWKKNISQPRLEER----------------------------------
016 YP_001931967.1 ILKAEVIHSNN-EI------------------------------------
017 YP_002455786.1 S---
018 YP_009109694.1 --
019 NP_789739.1 --
020 NP_955619.1 S---
021 YP_224289.1 VCKWTTVG---KAQEK----------------------------------
022 YP_009182100.1 TIGAISPDKKEE--------------------------------------
023 NP_705927.1 --
024 YP_009508577.1 VHYKKTPHQLLFG-------------------------------------
025 YP_004442830.1 ICCWKKSKNDPRSTEQ----------------------------------
026 NP_758887.1 Q---
027 YP_009508406.1 TLLKKIEGSVL---------------------------------------
028 YP_610965.1 ICKWKQAKKDPRGKEK----------------------------------
029 YP_001931961.1 ILKAIHQSE---
030 YP_241114.1 N---
031 YP_006732334.1 VLKAQTTEGQ--
032 YP_009041481.1 VCKWCPPNKRTKGEER----------------------------------
033 YP_001036293.1 VCKWKKLKKDFRRNER----------------------------------
034 YP_605811.1 VCKWKLHPSDTRLAEK----------------------------------
035 NP_955577.1 GPHGLTPFEILYGAPPPMAHFF----------------------------
036 YP_003208050.1 VCKWKPNKHSPKSQEK----------------------------------
037 NP_789740.1 Q---
038 YP_004442839.1 ICKWKAKPSDTRLNEK----------------------------------

```
039 YP_009507791.1   Q-------------------------------------------------
040 YP_004581513.1   VCKWKQSAYDPRSKER----------------------------------
041 NP_043933.1      ILKAKLPEGKE---------------------------------------
042 YP_009507248.1   FTKCRPFDLLNGHFDPRD--------------------------------
043 YP_002117531.1   VCKWKLTRHDSKTTEK----------------------------------
044 YP_004347415.1   CLKYK--KDKIEY-------------------------------------
045 NP_056891.1      N-------------------------------------------------
046 YP_009513242.1   PSSKYTPHQLLFG-------------------------------------
047 YP_009352866.1   ICKWCPIRKKAKKEER----------------------------------
048 YP_009165750.1   ILKAKTIDEDK---------------------------------------
049 NP_040840.1      A-------------------------------------------------
050 YP_004222728.1   A-------------------------------------------------
051 YP_009506264.1   ICKWKMPGAT-KASEK----------------------------------
052 YP_567050.1      S-------------------------------------------------
053 YP_009508571.1   PSLKHTPHQLLFG-------------------------------------
054 NP_056803.1      PVLKYTPHQLLFG-------------------------------------
055 NP_955564.1      A-------------------------------------------------
056 YP_009508411.1   VCKWKLFAKDPKATEK----------------------------------
057 YP_009140788.1   VCKWKPKRNDPRRSER----------------------------------
058 YP_009508410.1   --------------------------------------------------
059 NP_040333.1      GPHGLTPYEILYGAPPPLVNFP----------------------------
060 YP_008992013.1   VLLEEHNGKRH---------------------------------------
061 NP_041734.1      VCKWKLAKEDSRTTEK----------------------------------
062 NP_056895.1      S-------------------------------------------------
063 YP_009109689.1   NRFGLTPYEILYGGPPPLSTLL----------------------------
064 YP_002519387.1   VLKSRGTDN-----------------------------------------
065 YP_595725.1      VCKWKKNKADPRNTEQ----------------------------------
066 YP_009508551.1   PVLKYTPHQLLFG-------------------------------------
067 YP_001497148.1   NRFGLTPYEILYGGPPPLSTLL----------------------------
068 NP_041186.1      N-------------------------------------------------
069 NP_663784.1      L-------------------------------------------------
070 YP_009508443.1   VCKWKKFLKDPRVTEQ----------------------------------
071 YP_003987465.1   ICKWKFPGAP-RNQEK----------------------------------
072 NP_861410.1      CLKAAEL-------------------------------------------
073 YP_009345071.1   ICKWKKKEKDPKSTEK----------------------------------
074 YP_009508582.1   VHYKKTPHQLLFG-------------------------------------
075 NP_569141.1      VLKYRYNKEKIEH-------------------------------------
076 YP_002916057.1   ICKWKQFAYDPRSKER----------------------------------
077 NP_777384.2      S-------------------------------------------------
078 YP_233110.1      VCKWKLHPSDTRLAEK----------------------------------
079 NP_542258.1      S-------------------------------------------------
080 YP_009130664.1   VCKWKPQKCDGRQMEK----------------------------------
081 YP_009508566.1   PKIKLTPHQLLFG-------------------------------------
082 YP_006607892.1   ILKARTENSDS---------------------------------------
083 YP_009506270.1   VCKWKDNSSQPRWSEK----------------------------------
084 NP_042513.1      VLMAVPNAYEEFLSFVTNNRPSDLKTFLKQKEGSSRYAEKSVPEKLNSMH
085 YP_006907834.1   ILKAIHIDLSTNES------------------------------------
086 YP_004442827.1   VCYWKKSKADPRSTEQ----------------------------------
087 YP_009408594.1   VCKWKKAKGDPRSTER----------------------------------
088 YP_009508561.1   PRTKLTPHKLLFG-------------------------------------
089 NP_056907.1      S-------------------------------------------------
090 YP_002308474.1   S-------------------------------------------------
091 YP_007761644.1   VLQFYRKIEQEVFEK-----------------------------------
092 NP_040550.1      A-------------------------------------------------
093 NP_758808.1      ICKWKLAEYDPKSSEQ----------------------------------
094 YP_009345075.1   VAKWRPFKGASRGTEK----------------------------------
095 NP_068729.1      CLRALPKGKQKIGLDEFGIPTADLCTGS-------------------SSA
096 NP_047255.1      GPHGLTPFEILYGAPPPMAHFF----------------------------
097 YP_006273075.1   ICKWKPKRNDPRRMEK----------------------------------
098 YP_009508888.1   PILKHTPHQLLFG-------------------------------------
099 NP_395469.1      VMTAVTP----VHITNYGISLEDLF-------------------------
100 YP_009508546.1   PVLKHTPHQLLFG-------------------------------------
101 NP_056762.1      VLVCKPDKYSGKDTEK----------------------------------
102 YP_009508556.1   PSSKYTPHQLLFG-------------------------------------
103 NP_569153.1      VCKWKVAQYDPRSSER----------------------------------
104 NP_043924.1      FSAYELMTGRVPHLGG----------------------------------
105 YP_009243641.1   S-------------------------------------------------
106 YP_004300274.1   VLKYKYHQDKIEY-------------------------------------
107 NP_056848.1      CLKYKPKNSKIEY-------------------------------------
108 YP_006495799.1   VCKWKHKKGESASAEK----------------------------------
109 YP_009508409.1   DILRRLEGAVT---------------------------------------
110 NP_687035.1      Q-------------------------------------------------
111 NP_045937.2      KKHGLSPHEIVMG-------------------------------------
```

```
112 NP_612577.1       ILKAIHNSH-----------------------------------------
113 NP_619548.1       VLKARALDGV----------------------------------------
114 NP_056880.1       A-------------------------------------------------
115 YP_009121747.1    ICKWKKKKEDPRRDEK----------------------------------
116 YP_009113237.2    VCKWKTGESDPRTTER----------------------------------
117 YP_009508408.1    VCKWKAFTHDPRKDER----------------------------------
118 NP_955579.1       --------------------------------------------------
119 YP_009229919.1    VCKWKPNQSDPKSTER----------------------------------
120 YP_004442833.1    VCLWKKSKNDPKSTEQ----------------------------------
121 NP_862833.2       N-------------------------------------------------
122 NP_040563.1       S-------------------------------------------------
123 YP_443922.1       AGTHLSPHEIMTGRVMPGPPRE----------------------------
124 NP_057933.2       GPHGLTPYEILYGAPPPLVNFP----------------------------
125 YP_009116631.1    VCKWKPMKADSASKEE----------------------------------
126 YP_009513249.1    VSTKYTPHQLMFG-------------------------------------
127 YP_223871.1       GREGLSPFEILYGLKPPVVPRV----------------------------
128 NP_127504.1       VLLEEHNGKRK---------------------------------------
129 NP_044929.1       TRQKYTPHQLMYG-------------------------------------
130 NP_057860.1       S-------------------------------------------------
131 NP_040973.1       S-------------------------------------------------
132 NP_777317.1       ICKWKIFG--PRTSEK----------------------------------
133 NP_054716.1       AKIGKTPHQLMYG-------------------------------------
134 YP_001856242.1    --------------------------------------------------
135 NP_954565.2       N-------------------------------------------------
136 NP_569150.1       VCKWKTDSLQPRWSEK----------------------------------
137 NP_659397.1       VLKAQTTEGE----------------------------------------
138 YP_009513211.1    GQFGLTPYEILHGGPPPVLASG----------------------------
139 YP_233107.1       ICKWKLNKGEPRSAEK----------------------------------
140 YP_009109692.1    NRFGLTPYEILYGGPPPLSTLL----------------------------
141 NP_057849.4       Q-------------------------------------------------
142 NP_049560.1       S-------------------------------------------------
143 YP_009506267.1    VCKWRPKKNDPKSTEK----------------------------------
144 NP_039820.1       VCKWKMSKHDPRSTER----------------------------------
145 NP_056790.1       GRFGLTPYEILYGGPPPILESG----------------------------
146 YP_002321513.1    ICKWKHKKNDPRSSER----------------------------------
147 NP_955591.1       --------------------------------------------------
148 YP_001956722.2    PSSKYTPHQLLFG-------------------------------------
149 NP_056902.1       Q-------------------------------------------------
150 NP_056886.1       A-------------------------------------------------
151 NP_041261.1       S-------------------------------------------------
```

```
001 YP_004442836.1    ----------------------------------ICAYASGKFNPIKSTI
002 NP_056728.1       --------------------------------ELICRYASGSFKAAEKNY
003 YP_009508537.1    ---------------------------------IDCNLPFANKDTLDWTR
004 YP_004732983.2    ----------------------------------ICAYASGKFPTVKSTI
005 YP_009508407.1    --------------------------------------------------
006 NP_040939.1       ------------------------------------------AALVAINI
007 YP_003864102.1    ------------------------------------------HALFILNF
008 YP_009002585.1    ----------------------------------ICAYASGKFHPIKSTI
009 YP_003284237.1    ----------------------------------ICAYASGKFSPIKSTI
010 YP_009268869.1    ------------------------------------------AALITLNI
011 YP_009506251.1    ----------------------------------ICAYASGKFGVIKSTI
012 NP_955611.1       --------------------------------------------------
013 YP_008567619.1    ----------------------------------VCAYCSGKFDPPKSTI
014 NP_149413.1       ----------------------------------ICRYASGKFDKPKGTC
015 YP_004442824.1    ----------------------------------VCAYASGKFTPIKSTI
016 YP_001931967.1    -------------------------------TEEICCYASGTFKQAELNY
017 YP_002455786.1    ------------------------------------------KALWTINH
018 YP_009109694.1    --------------------------------------------------
019 NP_789739.1       --------------------------------------------------
020 NP_955619.1       ------------------------------------------IALWTINH
021 YP_224289.1       ----------------------------------VCAYASGKFTPIKSTI
022 YP_009182100.1    ---------------------------------VICAYRSGTFKPAEQNY
023 NP_705927.1       --------------------------------------------------
024 YP_009508577.1    ---------------------------------VDGNVPFANQDTLDLTR
025 YP_004442830.1    ----------------------------------ICRYASGKFAKPKSTI
026 NP_758887.1       ------------------------------------------MAVLIHNF
027 YP_009508406.1    ----------------------------------L---------------
028 YP_610965.1       ----------------------------------VAAYASGRFPVPKSTI
029 YP_001931961.1    --------------------------------ERICRYTSGSFKKAELNY
030 YP_241114.1       ------------------------------------------HALYVLNF
```

```
031 YP_006732334.1  --------------------------------ELIC---------STVQE
032 YP_009041481.1  ----------------------------------VCAYVSGKFPTVKSTI
033 YP_001036293.1  ----------------------------------VCAYASGKFDPPKSTI
034 YP_605811.1     ----------------------------------VCAYASGRYHPIKSTI
035 NP_955577.1     --------------------------------DTDISSFATSPT-MQAHL
036 YP_003208050.1  ----------------------------------VAAYASGKFPVVKSTI
037 NP_789740.1     ------------------------------------------MAVFIHNF
038 YP_004442839.1  ----------------------------------ICAYASGSYNPIKSTI
039 YP_009507791.1  ------------------------------------------LAVHALNH
040 YP_004581513.1  ----------------------------------IAAYASGKFQPIKSTI
041 NP_043933.1     ---------------------------------VICRYASGTFKPAEKNY
042 YP_009507248.1  --------------------------------PLDIDLTEHILQQYAQNH
043 YP_002117531.1  ----------------------------------TCAYASGKFSPVKSTI
044 YP_004347415.1  ----------------------------------ICRYNSGTFKEHEKNY
045 NP_056891.1     ------------------------------------------HALFILNF
046 YP_009513242.1  ---------------------------------VDSNTPFANSDTLDLSR
047 YP_009352866.1  ----------------------------------ICAYVSGKFLTVKSTI
048 YP_009165750.1  --------------------------------EYICRYTSGSFKQAELNY
049 NP_040840.1     ------------------------------------------GTLITLNI
050 YP_004222728.1  ------------------------------------------KAMYALNH
051 YP_009506264.1  ----------------------------------VCAYASGRFPAVKSTI
052 YP_567050.1     ------------------------------------------KALWTLNQ
053 YP_009508571.1  ---------------------------------IDSNTPFANQDTLDLTR
054 NP_056803.1     ---------------------------------IDSNTPFANQDTLDLTR
055 NP_955564.1     ------------------------------------------HALFVLNH
056 YP_009508411.1  ----------------------------------VCAYASGKFNPPKSTI
057 YP_009140788.1  ----------------------------------ICAYASGKFNPVKSTI
058 YP_009508410.1  --------------------------------------------------
059 NP_040333.1     --------------------------------DPDMAKVTHNPS-LQAHL
060 YP_008992013.1  ----------------------------------FCAHASGQFKHSEKNY
061 NP_041734.1     ----------------------------------ICAYASGKFGVVKSTI
062 NP_056895.1     ------------------------------------------RALWTHNQ
063 YP_009109689.1  --------------------------------N-SFSPSNSKTD-LQARL
064 YP_002519387.1  -------------------------------LERLCRYTSGSFKPAEINY
065 YP_595725.1     ----------------------------------ICRYASGKFDKPKGTC
066 YP_009508551.1  ---------------------------------IDSNTPFANQDTLDLTR
067 YP_001497148.1  --------------------------------N-SFSPSDPKTD-LQARL
068 NP_041186.1     ------------------------------------------HALYVLNF
069 NP_663784.1     ------------------------------------------MAVHCMNF
070 YP_009508443.1  ----------------------------------VCAYASGRFDPPKSTI
071 YP_003987465.1  ----------------------------------VCAYASGRFQPIKSTI
072 NP_861410.1     --------------------LFPKGTKNKVVER-LCKYTSGIFSSAEQKY
073 YP_009345071.1  ----------------------------------ICAYASGPYNPLKSTI
074 YP_009508582.1  ---------------------------------VDGNVPFANQDTLDLTR
075 NP_569141.1     ----------------------------------HCRYYSGSYTEPQEKW
076 YP_002916057.1  ----------------------------------ITAYASGKFQPIKSTI
077 NP_777384.2     ------------------------------------------RALWTHNQ
078 YP_233110.1     ----------------------------------VCAYASGRYHPIKSTI
079 NP_542258.1     ------------------------------------------KSLWTINH
080 YP_009130664.1  ----------------------------------ICAYASGKFNPPKSTI
081 YP_009508566.1  ---------------------------------VDGNIPFANSDTLDLKR
082 YP_006607892.1  --------------------------------ELICRYTSGSFKAAELNY
083 YP_009506270.1  ----------------------------------ICAYASGKFTPIKSTI
084 NP_042513.1     DKSHGEKAEKSVSRQTSQGRSSSQSQQSYIGSFMITKWSSGTFKPAEENY
085 YP_006907834.1  -------------------------------IELVCRYASGSFKPAEQNY
086 YP_004442827.1  ----------------------------------VCRYASGKFNKPKSTI
087 YP_009408594.1  ----------------------------------VCAYANGKFPTVKSSI
088 YP_009508561.1  ---------------------------------VDGNVPFANQDTLDLTR
089 NP_056907.1     ------------------------------------------KALWTLNQ
090 YP_002308474.1  ------------------------------------------KTLWTINH
091 YP_007761644.1  -------------------------------DLRVSRYCSGTWNQTEQNW
092 NP_040550.1     ------------------------------------------KAMYALNH
093 NP_758808.1     ----------------------------------ICAYASGKFSPIKSTI
094 YP_009345075.1  ----------------------------------AFAYASGRFPALQSTI
095 NP_068729.1     SSDNSPAEIDKCHSASKQDTHVASKIKKLENELLLCKYVSGTFTDTETRY
096 NP_047255.1     --------------------------------DTDISSFATSPT-MQAHL
097 YP_006273075.1  ----------------------------------VCAYASGKFSPIKSTI
098 YP_009508888.1  ---------------------------------VDANIPFANQDTLDLTR
099 NP_395469.1     -----PKEQHTAQALSSQYQFFG--TKDFVQKELLTKYASGTFTDTEKRY
100 YP_009508546.1  ---------------------------------IDSNTPFANQDTLDLTR
101 NP_056762.1     -----------------------------------IAGYASGNFGEKKTW
102 YP_009508556.1  ---------------------------------VDSNTPFANSDTLDLSR
103 NP_569153.1     ----------------------------------VCAYASGKFNPPKSTI
```

```
104 NP_043924.1        ------------------------------YHPHPLETAKEEEVRIFLRA
105 YP_009243641.1     ------------------------------------------QVLFTLNF
106 YP_004300274.1     ----------------------------------HCRYYSGTFKDNEKNW
107 NP_056848.1        ----------------------------------LCRYNSGTFKENEQKY
108 YP_006495799.1     ----------------------------------VCAYASGKFPAIKSTI
109 YP_009508409.1     ----------------------------------V---------------
110 NP_687035.1        ------------------------------------------MATHIHNF
111 NP_045937.2        ---------------------------------RPMKTTYLSDMSPLWAT
112 NP_612577.1        --------------------------------EYICRYASGSFKAAERNY
113 NP_619548.1        --------------------------------ELICRYSSGSFKQAEKNY
114 NP_056880.1        ------------------------------------------HALFVLNH
115 YP_009121747.1     ----------------------------------ICAYASGKFKVVQSTI
116 YP_009113237.2     ----------------------------------VCAFASGTFSPIKSTI
117 YP_009508408.1     ----------------------------------ICAYASGKFDPPKSTI
118 NP_955579.1        --------------------------------------------------
119 YP_009229919.1     ----------------------------------PCAYASGKFPAVKSTI
120 YP_004442833.1     ----------------------------------ICRYASGKFAKPKSTI
121 NP_862833.2        ------------------------------------------HALYVLNF
122 NP_040563.1        ------------------------------------------LALVSLN-
123 YP_443922.1        --------------------------------GGHMPALDVHQIGMTDYV
124 NP_057933.2        --------------------------------DPDMTRVTNSPS-LQAHL
125 YP_009116631.1     ----------------------------------ICAYASGKFPTVKSTI
126 YP_009513249.1     ---------------------------------IDCNLPFANKDTLDWTR
127 YP_223871.1        --------------------------------GCDKLASITNQT-LLKSL
128 NP_127504.1        ----------------------------------ICGFASGKFKVSEQHY
129 NP_044929.1        ---------------------------------ADCNLPFENLDTLDLTR
130 NP_057860.1        ------------------------------------------IALWTINH
131 NP_040973.1        ------------------------------------------LAVHSLNF
132 NP_777317.1        ----------------------------------VCAYASGKFNPIKSTI
133 NP_054716.1        ---------------------------------VDCNLPFQDLSTLDLTR
134 YP_001856242.1     --------------------------------------------------
135 NP_954565.2        ------------------------------------------HALFILNF
136 NP_569150.1        ----------------------------------ICAYASGKFTPIKSTI
137 NP_659397.1        --------------------------------ELICRYSSGTFKPAELNY
138 YP_009513211.1     --------------------------------EVVGSNGDFFPV-LFTHL
139 YP_233107.1        ----------------------------------ICAYASGRFNPIKGAI
140 YP_009109692.1     --------------------------------N-SFSPSNSKTD-LQARL
141 NP_057849.4        ------------------------------------------MAVFIHNF
142 NP_049560.1        ------------------------------------------IALWTINH
143 YP_009506267.1     ----------------------------------ICAYASGKFDIPKSTI
144 NP_039820.1        ----------------------------------ICAYASGSFNPIKSTI
145 NP_056790.1        --------------------------------ETLGPDDRFLPV-LFTHL
146 YP_002321513.1     ----------------------------------VCDYASGKFPVVKSSI
147 NP_955591.1        --------------------------------------------------
148 YP_001956722.2     ---------------------------------IDSNTPFANSDTLDLSR
149 NP_056902.1        ------------------------------------------LALITCNK
150 NP_056886.1        ------------------------------------------KAMYALNH
151 NP_041261.1        ------------------------------------------HALFQLNF
```

```
001 YP_004442836.1     D---AEIQAVINSLDKFKIYYLDKKELIIRTDSQAIVSFYKKSSDHKPSR
002 NP_056728.1        HSNDKETLAVINTIKKFSIYLTPVHFLIRTDNTHFKS-FVNLNYKGDSKL
003 YP_009508537.1     EEELALLQEIRESLQHPVRPP---TCSGWSPYVGQLVQERVYRPSQLRPK
004 YP_004732983.2     D---AEIYAVMASLENFKIYYLDKREITIRTDCQAIISFYDKTAIKKPSR
005 YP_009508407.1     --------------------------------------------------
006 NP_040939.1        KRKGGLG-TSPMDIFIYNKEQKRINNKYNKNSQKIQFCYYRIRKRGHQES
007 YP_003864102.1     LNLDD-QGKSAADRFWHS--------------DPKKQLAMVKWKDPLDNS
008 YP_009002585.1     D---AEIHAVMNSLEAFKIHYLDKPSLTIRTDCQAIIAFFDTISNHKPSR
009 YP_003284237.1     D---AEIQAVINSLDKFKIYYLDKKKIIIRTDCQAIVAFYSKIAQNKPSR
010 YP_009268869.1     KRKGGLG-TSPMDIFIFNKEQQRIQQQSTRNQSKFRFCYYRVRKRGHPGE
011 YP_009506251.1     D---AEIYALIKALDAFKIFYLDKGHLIVRTDCQAIVTFYNKTNTHKPSR
012 NP_955611.1        --------------------------------------------------
013 YP_008567619.1     D---AEIHAVMNSLEKLKIYYLDKREIIIRTDCQAIISFFNKSSVNKPSR
014 NP_149413.1        D---AEIYGVMNGLEKMRLFYLDKREITVRTDSAAIERFYNKSVEHKPSE
015 YP_004442824.1     D---AEVQAVINSLEKFKIYYLDKKELIIRTDSQAIVTFYKKISDHKPSR
016 YP_001931967.1     HSNEKEILAVIRSIQSFPVYLTPVEFIVRTDNKTMEH-FLTSKFELGTKS
017 YP_002455786.1     LNVMRPCGKTRWQLHHTPPLPPISESIQT--TPTRLHWYYYKTPGLTNQR
018 YP_009109694.1     --------------------------------------------------
019 NP_789739.1        --------------------------------------------------
020 NP_955619.1        LNVLTNCHKTRWQLHHSPRLQPIPETHSL--SNKQTHWYYFKLPGLNSRQ
021 YP_224289.1        D---AEVQAVINSLDKFKIYYLDKKELLIRTDCEAIVRFYKSTAQNKPSR
022 YP_009182100.1     STQEKEVLAIIRTIQKAKIFVLKP-FLVRTDSKFAAL-FLDKKINESLAR
```

```
023 NP_705927.1         --------------------------------------------------
024 YP_009508577.1      EEELSLLSEVRTSLLPPSTP--PASKRAWLPSVGLLVQERVARPSQLRPK
025 YP_004442830.1      E---AEITGVLQSLEKFRLYYIDKAEITVRTDSSAIERFYNKSTEHKPSE
026 NP_758887.1         KRKGGIGEYSPGERIVDIIATD-LLTTKLQHNIQKIQNFRVYYREGRDQQ
027 YP_009508406.1      --------------------------------------------------
028 YP_610965.1         D---AEIYACTETLSALKIHYLDRKEIILRTDCQAIISFYNKSSVNKPSR
029 YP_001931961.1      HSNEKEILAVIRVIAKFTIYLTPLEFLIRTDNKNFTF-FMNTNVKGDYKQ
030 YP_241114.1         LTLDA-EGNSAAQRFWGE--------------RSSCKKPLVRWKDPLTNL
031 YP_006732334.1      HSNQQ----------------KPITIVMKKNY------------------
032 YP_009041481.1      D---AEIFAVMESLSALKIFFLDKEEITVRTDCQAIIAFYNKQAQNKPSR
033 YP_001036293.1      D---AELFAVMNSMDKFKIHYLDRKELLIRSDCQALISFYNKSSENKPSR
034 YP_605811.1         D---AEVHAVINSLEKFKIYYLDKKELIIRTDSQAIVAFYKKQADHKPSR
035 NP_955577.1         RALQLVQEEIQRPLAAAYREKLETPVVPHPFKPGDSVWVRRHQTKNLEPR
036 YP_003208050.1      D---AEIHACMGTLSALKIHYLDRKEITLRTDCQAIVSFHNKMAQNKPSR
037 NP_789740.1         KRKGGIGGYSAGERIVDIIATD-IQTKELQKQITKIQNFRVYYRDSRNPL
038 YP_004442839.1      D---AEVHAVINSLEKFKIYYLDKKELIIRTDSQAIVAFYKKQADHKPSR
039 YP_009507791.1      KRKGGLGGITPYELYVQ-QESIRIQELYSSIPHKFLKDSWIYYKDRKDKL
040 YP_004581513.1      D---AEIFAIMNAMEAFKIYYLDKKEMIIRTDCEAIVSFFNKSASNKPSR
041 NP_043933.1         HSNEKEILSIIKAIKAFRAYILPYKFLVRTDNTNAAY-FVRTNIAGDYKQ
042 YP_009507248.1      RQQMKQVYEIINETSLANRTALIESRNKTRESEVEYIPQQQVFIKNPLAS
043 YP_002117531.1      D---AEIQAVICSLDKFKIFYLDKQEVVIRTDCQAIISFFDKTSQNKPSR
044 YP_004347415.1      DINRKELIAIYKGLEHYAIFTTQGKKLVRTDNSQAYYWIKNSKIKNSIDM
045 NP_056891.1         LNLDD-QNKSAADRFWHN--------------NPKKQFAMVKWKDPLDNT
046 YP_009513242.1      EEELSLLQEIRSSLHQPTSP--PASSRSWSPSVGQLVQERVARPASLRPR
047 YP_009352866.1      D---AEIFAVMESLSALKIYFLDKEEITVRTDCQAIISFYNKQAQNKPSR
048 YP_009165750.1      HSNEKEILAAMNTIKKFSGYLTPVKFLIRTDNKNFTF-FLNTNHKGDYKQ
049 NP_040840.1         KRKGGLG-TSPMDIFIFNKEQQRIQQQSKSKQEKIRFCYYRTRKRGHPGE
050 YP_004222728.1      FERGE-NTKTPIQKHWRP--------------TILTEGPPVKIRIETG-E
051 YP_009506264.1      D---AEIQAVINSLDKFKIYYLDKKALTIRTDCQAIVSFYAKIAQNKPSR
052 YP_567050.1         LNVMSSQWKTRWQLHHGPRLRPIPETPRP--SRAPTNWYYYKLPGLTNQR
053 YP_009508571.1      EEELSLLQEIRSSLHQPSTP--PASTRSWSPIGGQIVQERVPRPASLRPR
054 NP_056803.1         EEELSLLQEIRASLYQPSTP--PASSRSWSPVVGQLVQERVARPASLRPR
055 NP_955564.1         VNMDN-QGHTAAERHWGP--------------ISADPKPMVMWKDLLTGS
056 YP_009508411.1      D---AEIFAVMNSLEKFKIHYLDKEHIVIRTDCQAIISFYNKSVDNKPSR
057 YP_009140788.1      D---AEIHAVMNSLEKLKIYYLDKRELIIRTDCQAIISFFNKSAMNKPSR
058 YP_009508410.1      --------------------------------------------------
059 NP_040333.1         QALYLVQHEVWRPLAAAYQEQLDRPVVPHPFRVGDTVWVRRHQTKNLEPR
060 YP_008992013.1      HVIYKEILTVKYGIKKFEFHLISHTFLINMDNSSFPRIFDFKNKLLLDKQ
061 NP_041734.1         D---AEIYALIKALESFKIFYLDKKHLVVRTDCQAIVTFYNKTSTHKPSR
062 NP_056895.1         INLLP-ILKTRWELHHSPPLAVISEGGET--PKGSDKLFLYKLPGQNNRR
063 YP_009109689.1      KGLQAVQAQIWAPLAELYRP--GHSQTSHPFQVGDSVYVRRHRSQGLEPR
064 YP_002519387.1      HSNEKEVLAVKRTITKFKGYLASSEFLVRTDNKYFTY-FLRTSIKDDYKQ
065 YP_595725.1         D---AEIYGVMNGLEKMRLFYLDKREITVRTDSSAIERFYNKSAEHKPSE
066 YP_009508551.1      EEELSLLQEIRTSLYHPSTP--PASSRSWSPVVGQLVQERVARPASLRPR
067 YP_001497148.1      KGLQAVQAQIWTPLAELYRP--GHPQTSHPFQVGDSVYVRRHRSQGLEPR
068 NP_041186.1         LTLDT-EGNSAAQRFWGE--------------RSSCKKPLVRWKDPLTNL
069 NP_663784.1         KRRGGIGDMTPAERLINMITTE-QEIQFLQRKNSNFKNFQVYYREGRDQL
070 YP_009508443.1      D---AEIHAVMNSLDKFKIHYLDKEEIIIRTDCQAIISFYNKSAENKPSR
071 YP_003987465.1      D---AEIQAVINSLDKFKIYYLNQKELVVRTDCQAIVSFYEKMANNKPSR
072 NP_861410.1         TVHEKETLAALKTMRKWKAELLPKEFTLRTDSSYVTG-FARHNLKANYNQ
073 YP_009345071.1      D---AEISAVLHALETFKIFYLSQPTIIIRTDCQAIISFYNKSFNHKPSR
074 YP_009508582.1      EEELSLLSEVRTSLLPPSTP--PASRRSWLPSVGLLVQERVARPSQLRPK
075 NP_569141.1         EINRKELFALYKCLLAFEPYIVYTRFIVRTDNTQVKWWITR-KVQDSVTT
076 YP_002916057.1      D---AEIFAIMNAMEAFKIYYLDKKEMVIRTDCQAIVSFFNKSASNKPSR
077 NP_777384.2         INLLP-ILKTRWELHHSPPLAVISEGGET--PKGSDKLFLYKLPGQNNRR
078 YP_233110.1         D---AEVHAVINSLEKFKIYYLDKKELIIRTDSQAIVAFYKKQADHKPSR
079 NP_542258.1         LNVLPSCQKTRWQLHQAQPLPSISENTLP--PRASPKWYYYKIPGLTNPR
080 YP_009130664.1      D---AEIHAVMNSLNSFKIYYLDKKELLIRTDCQAIISFFNKSAKNKPSR
081 YP_009508566.1      EEELALLSEIRTTLSTVSPEPFPSTAKTWTPSVGLLVQERVYRPSQLRPK
082 YP_006607892.1      HSNEKEILAVMNTIKKFTGYLTPVKFLIRTDNKNFTH-FLKINLKGDYKQ
083 YP_009506270.1      D---AEIQAVINSLDKFKIYYLDKKELVIRTDSQAIVSFYKKSSDHKPSR
084 NP_042513.1         TVHEKELLAFVNALKKFQIDLRPVKFIFQTDNSWVQG-FLVNKLKETYNR
085 YP_006907834.1      HSNDKETLAVIRTIQKFSIYLTPVRFLVRTDNTHFKY-FLNINYKGDSKM
086 YP_004442827.1      E---AEIFGVLNSLEKFRLYYIDKAEITVRTDSSAIEKFYNKSTEHKPSE
087 YP_009408594.1      D---AEIFACMETLDALKIHYLDKEEITLRTDCQAIISFYNKSATNKPSR
088 YP_009508561.1      EEELSLLAELRSSFLPSASP--PASSRSWRPSVGLLVQERVARPSQLRPK
089 NP_056907.1         LNVMSSQWKTRWQLHHGPRLRPIPETPRP--SRAPTNWYYYKLPGLTNQR
090 YP_002308474.1      LNVLSSCQKTRWQLHQAQPLPPVPENLPL--PEPVPKWYYYKIPGLTSSR
091 YP_007761644.1      STFGKELRAIKLALQKFKLFLFEP-FTLYSDNLAVIN-FLKKDLNEKRSQ
092 NP_040550.1         FERGE-NTKTPIQKHWRP--------------TVLTEGPPVKIRIETG-E
093 NP_758808.1         D---AEITAAMEGLEAFKIHYLDKQKITLRTDCQAIISFCNKTSVNKPSR
094 YP_009345075.1      D---AEISALINGLESFKIHYLDKGEIMVRTDCHAIVSFYNKLHANKPSR
095 NP_068729.1         PIAELEVLAGVKVLEKWRIDLLQTRFLLRTDSKYFAG-FCRYNIKTDYRN
```

```
096 NP_047255.1        RALQLVQEEIQRPLAAAYREKLETPVVPHPFKPGDSVWVRRHQTKNLEPR
097 YP_006273075.1     D---AEIHAVMNTLEKLKIYYLDKKELIIRTDCQAIISFFNKSALNKPSR
098 YP_009508888.1     EEELSLLQEIRESLIQPPSP--PASSRSWTPAVGLLVQERVARPASLRPR
099 NP_395469.1        PIHELETLAVLQTFRKWKVDLLSKPFILKTDSKYVTG-FLRYKIKANYNQ
100 YP_009508546.1     EEELSLLQEIRTSLYQPSTP--PASVRSWSPVVGQLVQERVARPASLRPR
101 NP_056762.1        TSLDYEIEAINEALNKFQIY--LDKDFTIRTDCEAIVKGIKTEDYKKRSK
102 YP_009508556.1     EEELSLLQEIRSSLHQPSSP--PASSRSWSPSVGQLVQERVARPASLRPR
103 NP_569153.1        D---AEIHAVMNSLNNFKIYYLDKSSLCLRTDCQAIISFFNKSNVNKPSR
104 NP_043924.1        THDCMQNKKWEEQLEKVTKAEAHSHWTQRSPNLEPGCIVLVRKFTGDAFS
105 YP_009243641.1     MNLPQGDIQTTAERHFEE--------------KSEPLIHEDVWVRPLMET
106 YP_004300274.1     EINRKELLSVFKCLYAFEPYIVYNKFILRTDNTQVKYWLTG-KLDNSVTT
107 NP_056848.1        DINRKELIAVYQGLQSYSLFTCEGNKLVRTDNSQVYYWIKNDTNKKSIEF
108 YP_006495799.1     D---AEMHGVMNSLEKFQIYFMDKGEVTIRTDCQAIVAFYEKLNANKPSR
109 YP_009508409.1     --------------------------------------------------
110 NP_687035.1        KRKGGIGGISSAERLVNMLTTQ-LELNTLQNQIQKILNFKVYYREGRDPV
111 NP_045937.2        DTLVTYMNKLTRQLSAYHQQVVDQWPSTSLPPGPEPGSWCMLRNPKKSSN
112 NP_612577.1        HSNEKELLAVIRVIKKFSIYLTPSRFLIRTDNKNFTH-FVNINLKGDRKQ
113 NP_619548.1        HSNDKELLAVKQVITKFSAYLTPVRFTVRTDNKNFTY-FLRINLKGDSKQ
114 NP_056880.1        VNMDN-QGHTAAERHWGP--------------ISADPKPMVMWKDLLTGS
115 YP_009121747.1     D---AEINACINSLEKLKIYYLDKKEITLRTDCQAIISFYNKTNSNKASR
116 YP_009113237.2     D---AEIHAVMNSLVKFKLYYIGRQKITIRTDCQAIISFVNKSANSKPSR
117 YP_009508408.1     D---AEIHAVMNSLDKFKIHYLDKREILIRTDCQAIISFYNKSMENKPSR
118 NP_955579.1        --------------------------------------------------
119 YP_009229919.1     D---AEIFAVMEGLKNFKIYYLDRGAVTIRTDCQAIISFYNKSAQNKPSR
120 YP_004442833.1     E---AEISGVLCSLEKFRLYYLDKAEITVRTDSGAIERFYNKSTEHKPSE
121 NP_862833.2        LTLDA-EGNSAAQRFWGE--------------RSSCKKPLVRWKDPLTNL
122 NP_040563.1        KKRGGIGGHTPYEIYLESEHTKYQDQLEQQFSKQKIEKWCYVRNRRKE--
123 YP_443922.1        RKLTELSAALSIQIQRVAEGELTDDQDQPRVKVGDWVRIKVHKRKWADPR
124 NP_057933.2        QALYLVQHEVWRPLAAAYQEQLDRPVVPHPYRVGDTVWVRRHQTKNLEPR
125 YP_009116631.1     D---AEIFAVMESLEKFKIFYMNKDEVTIRTDCQAIITFYEKLNAKKPSR
126 YP_009513249.1     EEELALLQEIRESLQHPVQPP---TCSGWSPYVGQLVQERVYRPSQLRPK
127 YP_223871.1        QALQATRSLARATLRDQLPQKEAQQDRTPLFQPGDLVFVKKHDFQQLGPR
128 NP_127504.1        HSTFKEILAVKNGIKKFNFFLIHTNFLVEMDMRAFPKMIRLNPKIVPNSQ
129 NP_044929.1        EEQLAVLKEVRDGLLDLYPSPSQTTARSWTPSPGLLVQERVARPAQLRPK
130 NP_057860.1        LNVLTNCHKTRWQLHHSPRLQPIPETHSL--SNKQTHWYYFKLPGLNSRQ
131 NP_040973.1        KRRGRIGGMAPYELLAQ-QESLRIQDYFSAIPQK-LQAQWIYYKDQKDKK
132 NP_777317.1        D---AEIQAVINSLDKFKIYYLDKKELVIRTDSQAIVTFYKRMADHKPSR
133 NP_054716.1        EEQLAVLQEIRTALEQPAQHP---TLPKWTPCPGLLVQERVNRPAQLRPK
134 YP_001856242.1     --------------------------------------------------
135 NP_954565.2        LNLDD-QNKSAADRFWHN--------------NPKKQFAMVKWKDPLDNT
136 NP_569150.1        D---AEIQAVINSLDKFKIYYLDKKELIIRTDSQAIVSFYKKSSDHKPSR
137 NP_659397.1        HSNEKELLAVKQVITKFSIYLTPVCFTVRTDNVNLLKGFMNKKITGDSKQ
138 YP_009513211.1     KALEVVRTQIWDQIKEAYRP--GTVAIPHPFQVGDRVLVRRHRSGSLEPR
139 YP_233107.1        D---AEIQAVIYSLEKFKIYYLDKRELILRTDSKAIVRFYEKCSEHKPSR
140 YP_009109692.1     KGLQAVQAQIWAPLAELYRP--GHSQTSHPFQVGDSVYVRRHRSQGLEPR
141 NP_057849.4        KRKGGIGGYSAGERIVDIIATD-IQTKELQKQITKIQNFRVYYRDSRNPL
142 NP_049560.1        LNVLTHCHKTRWQLHHSPRLQPIPETRSL--SNKQTHWYYFKLPGLNSRQ
143 YP_009506267.1     D---AEIHAVMNCMDKFKIHYLDKKELLIRTDCHAIMAFYNKSSCNKPSR
144 NP_039820.1        D---AEIQAAIHGLDKFKIYYLDKKELIIRSDCEAIIKFYNKTNENKPSR
145 NP_056790.1        KALEIVRTQIWDQIKEVYKP--GTVTIPHPFQVGDQVLVRRHRPSSLEPR
146 YP_002321513.1     D---AEIHAAMFTLEKLKIYYLDKEEIILRTDCQAIISYYNKSNSNKPSR
147 NP_955591.1        --------------------------------------------------
148 YP_001956722.2     EEELSLLQEIRSSLYLPSTP--PASIRAWSPSVGQLVQERVARPASLRPR
149 NP_056902.1        GRE-SMGGQTPWEVFITNQAQVIHEKLLLQQAQS--SKKFCFYKIPGEHD
150 NP_056886.1        FERGE-NTKTPIQKHWRP--------------TVLTEGPPVKIRIETG-E
151 NP_041261.1        LQLDS-QGRSAADRLWHP--------------QTSQQHATVMWRDPLTSV
```

```
001 YP_004442836.1     VRWLAFTDYITGTGLDVKFEHIDGKDNVLADTLSRLVK------------
002 NP_056728.1        GRNIRWQAWLSHYS--FDVEHIKGTDNHFADFLSREFNKVNS--------
003 YP_009508537.1     WRKPTKVLEILNPRTVIIVDHLGQRKPVSIDNLKPTAHQHNG--------
004 YP_004732983.2     VRWINFCDYITNTGIKVQFEHIKGQDNQLADQLSR---------------
005 YP_009508407.1     --------------------------------------------------
006 NP_040939.1        GKDQPRYCGKGKEPIVVKDIESEKYLVIPYKDAKFIPPPTKEKE------
007 YP_003864102.1     WNGPDPVLIWGRGSVCVYSQAHDAARWLPERLVRQINNENQ---SRESYS
008 YP_009002585.1     LRWINFTDYVTGLGIPVNFQHVKGEDNRLADALSR---------------
009 YP_003284237.1     VRWLTFSDFISGLGVQVSFEHIDGKDNTIANTLSRLVH------------
010 YP_009268869.1     WLGPTQVLWEGEGAIVIKDKNLEKYLVIAKKDVKFIPQPKEIQTE-----
011 YP_009506251.1     IRWITFLDYITGLGVSVTIEHIDGKDNHLADTLSR---------------
012 NP_955611.1        --------------------------------------------------
013 YP_008567619.1     VRWIGFTDYITGLGIDVKFQHIDGKENSLADSLSR---------------
014 NP_149413.1        IRWIRFMDYITGAGPTIVIEHIKGKHNGLADILSRLKA------------
```

015	YP_004442824.1	VRWLAFVDYITGTGLNIRFEHIDGKDNILADTLSRLVQ------------
016	YP_001931967.1	GRLVRWQMWFKHYN--FKVEHIKGTSNFLADYLSREYNEI----------
017	YP_002455786.1	WKGPVQSLQEAAG-AALLQVSDGSPQWIPWRLLKKTVCPKPDDPEPAGHV
018	YP_009109694.1	--
019	NP_789739.1	--
020	NP_955619.1	WKGPQEALQEAAG-AALIPVSASSAQWIPWRLLKRAACPRPVG-GPADPK
021	YP_224289.1	VRWLMLTDFISGTGLEIKFEHINGCENILADSLSRLVQ------------
022	YP_009182100.1	GRLIRWQIMLRQYY--LDIEHIPGVSNYLPNALTREMAGCNIIYQIKMVN
023	NP_705927.1	--
024	YP_009508577.1	WKKPTPILEVVNDRTVVILDNQGQRRTVSIDNLKLTPHQD----------
025	YP_004442830.1	VRWIHFMDVISGAGPEIKFEHIKGKENTLADLLSRLN-------------
026	NP_758887.1	WKGPAELIWKGEGAVVIKEGTDLKVVPRRKAKIIRDYG--KTVDSDPNVE
027	YP_009508406.1	--
028	YP_610965.1	VRWLAFTDFITGLGIDVQFEHIKGEQNVLADTLSRLTFVLL---------
029	YP_001931961.1	GRLVRWQQWLSRYS--FKVEHITGVKNIFADFLTREFQSKNSIEL-----
030	YP_241114.1	WYGPDPVLIWGRGHVCVFPQDAEAPRWIPERLVRAAEELPDISNATHDIE
031	YP_006732334.1	--
032	YP_009041481.1	VRWLAFCDYINGNGLSVKFEHIKGEENVLADQLSR---------------
033	YP_001036293.1	VRWIAFNDFVTGLGIDVTFEHIDGKDNQLADSLSR---------------
034	YP_605811.1	TRWLMLIDYITGLGINVKFEHIDGKENVLADTLSRLVQ------------
035	NP_955577.1	WKGP--HIVLLTTPTALKVDG--VAAWIHASHVKAAGPTTNQDLSDSPSS
036	YP_003208050.1	VRWISFTDFITGLGIKVNIEHIDGKDNVLADTLSRLIFKIQ---------
037	NP_789740.1	WKGPAKLLWKGEGAVVIQDNSDIKVVPRRKAKIIRDYG--KQMAGDDCVA
038	YP_004442839.1	TRWLMLIDYITGLGINVKFEHIDGKENVLADTLSRLVQ------------
039	YP_009507791.1	WKGPTQVYYWGEGAVLIKDEN-NKYLLIPRRRIRRVPAPKDLTQEDGFNQ
040	YP_004581513.1	ARWISFTDYITGTGIKIRIEHIDGKDNILADYLSR---------------
041	NP_043933.1	SRLVRWQMALREYS--FDIEHVSGQKNVLADIMTRELAGKT---------
042	YP_009507248.1	RQKVAPRYTQDTVLADLPIHIYTSKKRGPVAKARLKRVPKGN--------
043	YP_002117531.1	VRWLTFTDFITGIGIPVRFEHISGKDNLLADTLSRLVL------------
044	YP_004347415.1	KNVKGILAKIIMYD--FDIEIIDGKTNIVADFLSRNGTDDATITG-----
045	NP_056891.1	WHGPDPVLIWGRGSVCVYSQTYDAARWLPERLVRQVSNNNQ---SRE---
046	YP_009513242.1	WHKPTSILEVVNPRTVVILDHLGNRRTVSVDNLKLTAHQN----------
047	YP_009352866.1	VRWLAFCDYINGNGLCVKFEHIKGEENFLADHLSR---------------
048	YP_009165750.1	GRLIRWQMWFSRYS--FTVEHLPGNKNVFADFLTREFNN-----------
049	NP_040840.1	WQGPTQVLWGGDGAIVVKDRGTDRYLVIANKDVKFIPPPKEIQKE-----
050	YP_004222728.1	WEKGWNVLVWGRGYAAVKNRDTDKVIWVPSRKVKPDITQKDEVTKKDEAS
051	YP_009506264.1	VRWLTFSDFITGLGVVVTFEHIDGKDNVLADTLSRLVV------------
052	YP_567050.1	WKGPLQSLQEAAG-AALLSID-GSPQWIPWRLLKKTVCPRPDGSELVAHA
053	YP_009508571.1	WHKPSRIVDILNERTVVIVDHLGNNRTVSIDNLKLTPHQ-----------
054	NP_056803.1	WHKPSTVLEVLNPRTVVILDHLGNNRTVSIDNLKPTSHQ-----------
055	NP_955564.1	WKGPDVLITAGRGYACVFPQDAETPIWVPDRFIRPFTERKEATPTPGTAE
056	YP_009508411.1	VRWVAFNDYLTGLGIEVKIEHIQGVHNTLADALSR---------------
057	YP_009140788.1	VRWISFTDFITGLGINVEFEHIDGKDNVLADSLSR---------------
058	YP_009508410.1	--
059	NP_040333.1	WKGP--YTVLLTTPTALKVDG--IAAWIHAAHVKAADTRI--------EP
060	YP_008992013.1	LLNLKTWFAK----YDFTVQHIKGNQNLIPDFLTRPAINKPSLISSIQTI
061	NP_041734.1	IRWITFSDYITGLGVPVTIEHIDGKENQLADTLSR---------------
062	NP_056895.1	WLGPLPALVEASG-GALLATN--PPVWVPWRLLKAFKCPKNDGPEDAHNR
063	YP_009109689.1	WKGP--YIVLLTTPTAIKVDG--IATWIHASHAKAAPGTP--------GP
064	YP_002519387.1	GRLIRWQQWFSHYK--FNVEHLAGTHNFTADSLTREFANKPP--------
065	YP_595725.1	IRWIRFMDYITGAGPEIVIEHIKGKSNGLADILSRLKA------------
066	YP_009508551.1	WHKPSTVLKVLNPRTVVILDHLGNNRTVSIDNLKPTSHQ-----------
067	YP_001497148.1	WKGP--YIVLLTTPTAIKVDG--IAAWIHASHAKAAPKTP--------GP
068	NP_041186.1	WYGPDPVLIWGRGHVCVFPQDAEAPRWIPERLVRAAEELPDASDATHDPE
069	NP_663784.1	WKGPGELLWKGEGAVIVKVGTDIKVVPRRKAKIIRDYGGRQELDSSPHLE
070	YP_009508443.1	VRWIAFNDFITGLGISVHFEHIDGKHNQLADALSR---------------
071	YP_003987465.1	VRWLTFSDFITGIGVPVKFEHIDGEDNLLADTLSRLVV------------
072	NP_861410.1	GRLVRWQLEFLQYP--ARVEYIKGEKNSLADTLTREWKQQ----------
073	YP_009345071.1	LRWIRLSDWLTGTGVAYKFEHIKGELNNLADHLSRNPEIALT--------
074	YP_009508582.1	WRKPTPILEVVNDRTVVILDNQGQRRTVSIDNLKLTPHQD----------
075	NP_569141.1	KEIRRLVLNILNFT--FTIEIINTNKNVVADYLSRQSYPN----------
076	YP_002916057.1	ARWISFTDYITGTGIKIRIEHIDGKDNTLADYLSR---------------
077	NP_777384.2	WLGPLPALVEASG-GALLATN--PPVWVPWRLLKAFKCPKNDGPEDAHNR
078	YP_233110.1	TRWLMLIDYITGLGINVKFEHIDGKENVLADTLSRLVQ------------
079	NP_542258.1	WSGPVQSLKEAAG-AALIPVG-GSHLWIPWRLLKRGICPRPESNAVADPE
080	YP_009130664.1	VRWVSFTDFITGTGLEIRFQHIDEKDNALADALSR---------------
081	YP_009508566.1	WKKPTPILEVLNERTVVIDNN-GQRRTVSVDNLKYTPHQKD---------
082	YP_006607892.1	GRLVRWQMWFSRYV--FEVEHLSGDKNVFADFLTREFHY-----------
083	YP_009506270.1	VRWLAFTDYITGTGLEVKFEHIDGKDNVLADTLSRLVK------------
084	NP_042513.1	GRLARWSMFIQQFD--FISLHIAGKENYLADTLTREWKTSSR--------
085	YP_006907834.1	GRNIRWQGWLQNYV--FDVDHIKGTNNCLADFLSREFNGA----------
086	YP_004442827.1	IRWIKFMDFISGAGPEIKFEHIKGKDNTLADLLSRLN-------------
087	YP_009408594.1	VRWLNFADFITGTGVKIVFEHIDGRQNVLADSLSRLITYMS---------

```
088 YP_009508561.1    WKKPVIICELINDRTVVIVDKAGNKRTVSIDNLKLTPHQKNS--------
089 NP_056907.1       WKGPLQSLQEAAG-AALLSID-GSPQWIPWRLLKKTVCPRPDGSELVAHA
090 YP_002308474.1    WSGPVQSVKEAAG-AALIPVG-TRHIWIPWRLLKRGACPRPGDSVTTESK
091 YP_007761644.1    -REIRDKLDILQYQGWMTLKHIPGTKNVLADALTRGLSN-----------
092 NP_040550.1       WEKGWNVLVWGRGYAAVKNRDTDKVIWVPSRKVKPDVTQKDEVTKKDEAS
093 NP_758808.1       VRWLKFIDYITNTGIDVKFEHIDAKNNVLADTLSRLVN-TLQDLP--WLD
094 YP_009345075.1    LRWAKLLDYLTGTGVQITLEHIDGRDNILADQLSRLSSYIS---------
095 NP_068729.1       GRLIRWQLRLQAYQ--PYVELIKSENNPFADTLTREWSKPSSS-------
096 NP_047255.1       WKGP--HIVLLTTPTALKVDG--VAAWIHASHVKAAGPTTNQDLSDSPSS
097 YP_006273075.1    VRWVAFTDFITGIGINVEFEHIDGRDNVLADSLSR---------------
098 YP_009508888.1    WHKPVKILEVLNPRTVVILDHLGNNRTVSVDNLKLTANQ-----------
099 NP_395469.1       GRFIRWQLELSQFN--YRTFYIKGSENYGPDTLTREWKEL----------
100 YP_009508546.1    WHKPSTVLEVLNPRTVVILDHLGNNRTVSIDNLKPTSHQ-----------
101 NP_056762.1       TRWIKLRDNLLKDGYKPTFEHIKGNKNFLPNFLSREGDFILKCLQNPDST
102 YP_009508556.1    WHRPTTVLEVVNPRTVIILDHLGNRRTVSVDNLKLTAHQD----------
103 NP_569153.1       VRWIAFTDFLTGLGIPVNIEHIDGKNNHLADALSR---------------
104 NP_043924.1       PKWEGPYVITETTKYAAKVQAMSDKVTQHSGWIHRTHLVLFPSQNKRWAD
105 YP_009243641.1    EWVPAQLLVKGKGYGLVLLQNGK-QQWLPACRIQVRRTTGQETIATASAV
106 YP_004300274.1    KEIRRLVVKINCYN--FDVVVIKSKDNCFADYLSREVKK-----------
107 NP_056848.1       RNIKYLLAKIAVYN--FEIQLIDGKTNIIADYLSRYNSSDTDGRYDEANT
108 YP_006495799.1    VRWLNFCDYITNTGVKVVFEHIKGKDNVLADTLSR---------------
109 YP_009508409.1    --------------------------------------------------
110 NP_687035.1       WKGPARLIWKGEGAVVIKEGEDIKVVPRRKAKIIKDYGERKTMDSEGSME
111 NP_045937.2       WEGPFLILLSTPTAVKVEGRPTWIHLDHCKLLRSSLSSSLG---------
112 NP_612577.1       GRLVRWQMWLSQYD--FDVEHIAGTKNVFADFLQENTLTNYV--------
113 NP_619548.1       GRLVRWQNWFSKYQ--FDVEHLEGVKNVLADCLTRDFNA-----------
114 NP_056880.1       WKGPDVLITAGRGYACVFPQDAETPIWVPDRFIRPFTERKEATPTPGTAE
115 YP_009121747.1    VRWLKFSDIITGTGVTINMEHIDGKNNVLADSLSRMVKLCFTGCT--EQE
116 YP_009113237.2    VRWISFTDFITGCGSEVRFEHIEGKENQLADHLSR---------------
117 YP_009508408.1    IRWIAFSDFISGLGISVHFEHIEGRHNQLADALSR---------------
118 NP_955579.1       --------------------------------------------------
119 YP_009229919.1    VRWLGFLDFITGSGVDVTFEHVEGKNNMLADALSRLTSNLCYAECPENQK
120 YP_004442833.1    VRWLKFMDFISGAGPEIKFEHIKGKDNTLADLLSRLN-------------
121 NP_862833.2       WYGPDPVLIWGRGHVCVFPQDAEAPRWIPERLVRAAEEFPDTSNASNDTE
122 NP_040563.1       WKGPYKVLWDGDGAAVIEEEGKTALYPHRHMRFIPPPDSDIQDGSS----
123 YP_443922.1       WTGP--YEVKEVTSHSVQVKGKSGAPWHHLTHCTPAPAPDR---------
124 NP_057933.2       WKGP--YTVLLTTPTALKVDG--IAAWIHAAHVKAADPGG--------GP
125 YP_009116631.1    VRWLAFCDYITNSGVRMKFEHIKGKDNQLADNLSR---------------
126 YP_009513249.1    WRKPTKVLEILNPRTVIIVDHLGQRKSVSIDNLKPTAHQHNG--------
127 YP_223871.1       WDGP--YTVVLSTPTAVKVAG--KTPWIHYSRLKKAPDNQ----------
128 NP_127504.1       LLRWAQWFSP----YQFEVKHLKGKDNILADFLSRPHEFSQRLKNSPKVL
129 NP_044929.1       WRKPTPIKKVLNERTVIIDHLG-QDKVVSIDNLKPAAHQKLA--------
130 NP_057860.1       WKGPQEALQEAAG-AALIPVSASSAQWIPWRLLKRAACPRPVG-GPADPK
131 NP_040973.1       WKGPMRVEYWGQGSVLLKDEE-KGYFLIPRRHIRRVPEPCALPEGDE---
132 NP_777317.1       VRWLAFTDYITGTGLDIKFEHIDGKENILADTLSRLVN------------
133 NP_054716.1       WKKPTPILKVLNPKTVVIAGPGGQERIVSIDNLKKTPHHDTS--------
134 YP_001856242.1    --------------------------------------------------
135 NP_954565.2       WHGPDPVLIWGRGSVCVYSQTYDAARWLPERLVRQVSNNNQ---SRE---
136 NP_569150.1       VRWLAFTDYITGTGLEIKFEHIDGKDNVLADTLSRLVK------------
137 NP_659397.1       GRLIRWQMWFSHYT--FKVDHLKGEQNVLADYLTREFHNGNVIHDD----
138 YP_009513211.1    WKGP--YLVLLTTPTAVKVDG--IAAWVHASHLKPAPPGAP---------
139 YP_233107.1       VRWMTLTDYISGCGVKVYFEHIDGKDNTLADELSRLVQA-----------
140 YP_009109692.1    WKGP--YIVLLTTPTAIKVDG--IATWIHASHAKAAPGTP--------GP
141 NP_057849.4       WKGPAKLLWKGEGAVVIQDNSDIKVVPRRKAKIIRDYG--KQMAGDDCVA
142 NP_049560.1       WKGPQEALQEAAG-PALIPVIASSAQWIPWRLLKRAACPRPVG-APPIPK
143 YP_009506267.1    VRWIAFNDFLTGLGIPVTIEHIDGKNNVLADALSR---------------
144 NP_039820.1       VRWLTFSDFLTGLGITVTFEHIDGKHNGLADALSRMIN------------
145 NP_056790.1       WKGP--YLVLLTTPTAVKVDG--IAAWVHASHLKPAPPSAP---------
146 YP_002321513.1    ARWIAFTDYITNTGTNIKFEHIEGKLNVLADALSR---------------
147 NP_955591.1       --------------------------------------------------
148 YP_001956722.2    WHKPTPVLEVINPRAVVILDHLGNRRTVSVDNLKLTAYQK----------
149 NP_056902.1       WKGPTRVLWKGDGAVVVNDEG-KGIIAVPLTRTKLLIKPN----------
150 NP_056886.1       WEKGWNVLVWGRGYAAVKNRDTDKVIWVPSRKVKPDITQKDEVTKKDEAS
151 NP_041261.1       WKGPDPVLIWGRGSACIYDQKEDGPRWLPERLIRHINNQTAPLCDRPSNP
```

```
001 YP_004442836.1    ---IICHKEKHPSETILINVAEEILQKGSIGAKRKLGEMISGYEAWMTR-
002 NP_056728.1       --------------------------------------------------
003 YP_009508537.1    --TRTCDDPEGMDGMECSQTTT----------------------------
004 YP_004732983.2    -----LAQNVCAIQVIPESA--------HEALSIILEQD-----------
005 YP_009508407.1    --------------------------------------------------
006 NP_040939.1       --------------------------------------------------
```

```
007 YP_003864102.1    P-------------------------------------------------
008 YP_009002585.1    -----LISVIFEAGQQWQE---------ESTRMQHLTQLIGE--------
009 YP_003284237.1    ---ILIKEEDQELINKIMVLWEKKTE------------------------
010 YP_009268869.1    --------------------------------------------------
011 YP_009506251.1    -----LVWSQFP------------------IEPPEEEPLKSQ--------
012 NP_955611.1       --------------------------------------------------
013 YP_008567619.1    -----LTCSLIR-QWHHLEP---------VITTMEAALVQEQ--------
014 NP_149413.1       ---KLAEA-PSEEVVLLAKALKEIAYYPDHPQVPKLIEWGKQILDPFPKF
015 YP_004442824.1    ---IIIHKEAHPAMSILIDAAEEVVRKYNPHSAARLGRMIEAVKDWQDNQ
016 YP_001931967.1    --------------------------------------------------
017 YP_002455786.1    ETDHQHHG------------------------------------------
018 YP_009109694.1    --------------------------------------------------
019 NP_789739.1       --------------------------------------------------
020 NP_955619.1       EKDHQHHG------------------------------------------
021 YP_224289.1       ---TLLQGWQHQHLNGILLALEELYQKPNPEVAKKIGQIIMKVLE-----
022 YP_009182100.1    HGKAPRMDLRDVITSKARARENQQLENEDIHWKQHLASTSRSFAKRKDVP
023 NP_705927.1       --------------------------------------------------
024 YP_009508577.1    ---GTSNEPDGMDPLEQKKKYDNHDI------------------------
025 YP_004442830.1    ---KVLKVEASTEMITLARALREIDYDQDHPAFKKIHEYSQKVKWP----
026 NP_758887.1       A-------------------------------------------------
027 YP_009508406.1    --------------------------------------------------
028 YP_610965.1       -QVPDPFSPPITELCQALKEIQATSTTPVGVWN---RFQTQICKT-----
029 YP_001931961.1    --------------------------------------------------
030 YP_241114.1       --------------------------------------------------
031 YP_006732334.1    --------------------------------------------------
032 YP_009041481.1    -----ITALLFVQEWPTQEE--------VEELGTFLTAIDDT--------
033 YP_001036293.1    -----LVTSLVRHEGKWQQQ---------RKALVIIEEMMQK--------
034 YP_605811.1       ---VLITRVHHPAETQLVEAVMEVLSNPKKEALDKVNHFIFLTQQWIAEQ
035 NP_955577.1       DDPSRWKVQRTQNPLKIRLSRGT---------------------------
036 YP_003208050.1    -EEGALMALERRPEWEPRPPPRVDNNSPMNIIYGLIHQDQQELIN-----
037 NP_789740.1       SRQDED--------------------------------------------
038 YP_004442839.1    ---VLVTKIHHPAEEQLVDAVLEVLKNPKEVAIKKVNHFILLTERWIAGF
039 YP_009507791.1    TEQESNNA------------------------------------------
040 YP_004581513.1    -----LVFSLIIAEWKTQ-EKSIAPLQAPRITLTKXSCSKQQEPLL----
041 NP_043933.1       --------------------------------------------------
042 YP_009507248.1    ---TLLQDSAATDNTCDASSRDKT--------------------------
043 YP_002117531.1    ---CMVQGWTHDGIKAITPAITAIKDDSDSARVAAIIMKVLP--------
044 YP_004347415.1    --------------------------------------------------
045 NP_056891.1       --------------------------------------------------
046 YP_009513242.1    --NGTTNDSGTMAPVEEDESGSPSS-------------------------
047 YP_009352866.1    -----ITALLVTGPWPTEEE--------AEELGTFLAVADDT--------
048 YP_009165750.1    --------------------------------------------------
049 NP_040840.1       --------------------------------------------------
050 YP_004222728.1    PLFAGISDWAPWKSEQEGL-------------------------------
051 YP_009506264.1    ---MFLQED------KYDDLIQKALK------------------------
052 YP_567050.1       ATDHQHHG------------------------------------------
053 YP_009508571.1    --DGTSNVSSTMDHLE----------------------------------
054 NP_056803.1       --NGTTNDTATMDHLEQNEQSS----------------------------
055 NP_955564.1       KTPPRDEKDQQESPKNESSPHQREDGLATSAGVDLRSGGGP---------
056 YP_009508411.1    -----LVGSLVFHTEAWNSR---------KEALMVAEACINK--------
057 YP_009140788.1    -----LINSFFLTEWN--------------QLKEQSMMELEE--------
058 YP_009508410.1    --------------------------------------------------
059 NP_040333.1       PSESTWRVQRSQNPLKIRLTRGTS--------------------------
060 YP_008992013.1    PVIALNRQLPFKALTQRHFPMNISFQSAYQLQ------------------
061 NP_041734.1       -----LVYTTWNQS--------------QTHQPEEEELEKSQ--------
062 NP_056895.1       SSDG----------------------------------------------
063 YP_009109689.1    TSSGTWRLRRSEDPLKIRLSRT----------------------------
064 YP_002519387.1    --------------------------------------------------
065 YP_595725.1       ---KLAQNEPTEEMILLTQAIREVIPYPDHPYTEQLREWGNKILDPFPTF
066 YP_009508551.1    --NGTTNDTATMDHLEQNE-------------------------------
067 YP_001497148.1    ETPKTWKLRRSENPLKIRLSRV----------------------------
068 NP_041186.1       --------------------------------------------------
069 NP_663784.1       GAREDGEMACPCQVPEIQNKRPRGGALCSPPQGGMGMVDLQQGNIPTTRK
070 YP_009508443.1    -----LVGALIQNSEKWSQR---------THIIRAMGLAIEQ--------
071 YP_003987465.1    ---MMLHEEAYTEPLQRILPLLSKKE------------------------
072 NP_861410.1       --------------------------------------------------
073 YP_009345071.1    ----KLVSTLISEWQDLPEP--------IYHLLSETLSMGEE--------
074 YP_009508582.1    ---GTSNEPNGVDPLEQEKEHDNHDI------------------------
075 NP_569141.1       --------------------------------------------------
076 YP_002916057.1    -----LVFSLIIAEWKTQGRRSTAHHQAQSMAQTIGSCSKQQEPLL----
077 NP_777384.2       SSDG----------------------------------------------
078 YP_233110.1       ---VLITKVHHPAETQLVEAVMEVISNPKKEALDKVNHFIFLTQQWIAER
079 NP_542258.1       TKDHQLHG------------------------------------------
```

```
080 YP_009130664.1    -----LVHGLVSKPDFHSDQ---------TILLVELAIAEAQ--------
081 YP_009508566.1    ---GETYDSS----------------------------------------
082 YP_006607892.1    --------------------------------------------------
083 YP_009506270.1    ---IIFHQEKHQSEEILINAVEKTLKKGDASTRQKINDLVKRYESWMNTG
084 NP_042513.1       --------------------------------------------------
085 YP_006907834.1    --------------------------------------------------
086 YP_004442827.1    ---RALKAEATIELVTLAQALKEIDYDQDHIAFEKIKRYAENIKWP----
087 YP_009408594.1    --------TEWQTLEEADQAAQAQLSIQTNLLL-----------------
088 YP_009508561.1    --HGSTPDITGMDVMEQEEEPGNMD-------------------------
089 NP_056907.1       ATDHQHHG------------------------------------------
090 YP_002308474.1    HKDLQLHG------------------------------------------
091 YP_007761644.1    --------------------------------------------------
092 NP_040550.1       PLFAGISDWIPWEDEQEGLQGETASNKQERPGEDTLAANES---------
093 NP_758808.1       EPHQDQTVSLMQEIEDAPLEIKQR----SLTCLQRLICRSFMEDST--EE
094 YP_009345075.1    -KEGKLVTSQQQQALKVLDQVYQAKDHQRWKDPALIACLHQLFPX-----
095 NP_068729.1       --------------------------------------------------
096 NP_047255.1       DDPSRWKVQRTQNPLKIRLSRGT---------------------------
097 YP_006273075.1    -----LINSFFLTEWS--------------LKKEEAVAELEK--------
098 YP_009508888.1    --NGSTNDSTTMAGLGQNAESQ----------------------------
099 NP_395469.1       --------------------------------------------------
100 YP_009508546.1    --NGTTNDITTMDHLEQNE-------------------------------
101 NP_056762.1       ESYSIDSSESIPLYIDSKESHSIESDDSIPLYRDKLLPLVERLKEKSA--
102 YP_009508556.1    --NGTTNDSGTMAPVEKDESGPSSS-------------------------
103 NP_569153.1       -----LVTGFVFAEPQCQDKFQDDLGKLEAALQEKKEAPQAM--------
104 NP_043924.1       PGNPGNQPDEDCTGKKGTVSTADMSPTTSTTRDRGINMGTEQTHRRRSPR
105 YP_009243641.1    --------------------------------------------------
106 YP_004300274.1    --------------------------------------------------
107 NP_056848.1       --------------------------------------------------
108 YP_006495799.1    -----LTQTLAAVREMPAEQ--------EEILRQALNNTEVQ--------
109 YP_009508409.1    --------------------------------------------------
110 NP_687035.1       GVREANKQMEGDSDLQDQE-------------------------------
111 NP_045937.2       ---GPVNQLLS---------------------------------------
112 NP_612577.1       --------------------------------------------------
113 NP_619548.1       --------------------------------------------------
114 NP_056880.1       KTPPRDEKDQQESPKNESSPHQREDGLATSAGVDLRSGGGP---------
115 YP_009121747.1    TEVLEKGLAAMGEIFEENVKLSNEKNGYEEIIQISSYCQKFIEDNSRLKN
116 YP_009113237.2    -----LINTLILYDEHCTQR--------QELNLLIPAIEENK--------
117 YP_009508408.1    -----LVLSLVQVPDEWHHQ---------QGLTSKIEDALEQ--------
118 NP_955579.1       --------------------------------------------------
119 YP_009229919.1    EELATLAEKAWNELSDGTKAGYNTAKLTMELAHLMMTWMDELSMSSREST
120 YP_004442833.1    ---AALKAEPTQELITLAKALKEIEYDEDNPVFKKIKEYSEKIKWP----
121 NP_862833.2       --------------------------------------------------
122 NP_040563.1       --------------------------------------------------
123 YP_443922.1       -TLTEVRSDLIASNLAINSESPDN--------------------------
124 NP_057933.2       SSRLTWRVQRSQNPLKIRLTREAP--------------------------
125 YP_009116631.1    -----LTQLITFVKWLPTEL--------KDLAAELTRKDDGT--------
126 YP_009513249.1    --TRTCDDPEGMDGMECSQTTT----------------------------
127 YP_223871.1       ---EEWTVSPTSDPLRVKLTRRAKP-------------------------
128 NP_127504.1       MFQRRTRSSSTKSKADSSQSTGSSYKLSHNLPENPPEVFNLDYPWDTSVF
129 NP_044929.1       --QTPDSAEICPSATPCPPNTSLWYDLDTGTWTCQRCGYQCPDKYHQPQC
130 NP_057860.1       EKDHQHHG------------------------------------------
131 NP_040973.1       --------------------------------------------------
132 NP_777317.1       ---FLCFAGTNEEVKELAVQAITQEQK-NPMGLKRLGQILQHKEERVHYL
133 NP_054716.1       --NDSTRMDAVEVPTECQ--------------------------------
134 YP_001856242.1    --------------------------------------------------
135 NP_954565.2       --------------------------------------------------
136 NP_569150.1       ---IILHPEKHQSEGVLINAVEEVFHKGNTDAKQRVNDVVKRYEDWLSKG
137 NP_659397.1       --------------------------------------------------
138 YP_009513211.1    --DESWELEKTDHPLKLRVRRRRNESTA----------------------
139 YP_233107.1       ---ILINKEESPIILSLIKATTEVLQKENPISRSRLALCISRALG-----
140 YP_009109692.1    TSSGTWRLRRSEDPLKIRLSRT----------------------------
141 NP_057849.4       SRQDED--------------------------------------------
142 NP_049560.1       KKTTNTMGKFLTTLILFFQFCPLILGDYSPSCCTLTIGVSSYHSKPCNPA
143 YP_009506267.1    -----LVCSIIKNPVYWQSK---------EELLKQTEEALEQ--------
144 NP_039820.1       ----FIVEKNDESPYRFTSSVEDALKVCN---------------------
145 NP_056790.1       --DESWELEKTDHPLKLRIRRRRDESAK----------------------
146 YP_002321513.1    -----ITCYLCTAGSQGCLAPEE-----DDHMKVMETLPEEIN-------
147 NP_955591.1       --------------------------------------------------
148 YP_001956722.2    --DGTPNESAAVVAMEKDE-------------------------------
149 NP_056902.1       --------------------------------------------------
150 NP_056886.1       PLFAGISDWIPWEDEQEGLQGETASNKQERPGEDTLAANES---------
151 NP_041261.1       NTAPGPKGSP----------------------------------------
```

001 YP_004442836.1 IQEHKIKTLTLIEK------------------------PIFKCGCR----
002 NP_056728.1 --
003 YP_009508537.1 ----ETSVDSS---------------------------------------
004 YP_004732983.2 ----CTAQELMAQ-----------------------FNSMLQANLR----
005 YP_009508407.1 --
006 NP_040939.1 --
007 YP_003864102.1 --
008 YP_009002585.1 ---HYKFEASGKK-------------------HHQLKETLTALITS----
009 YP_003284237.1 ------DVMHINDN------------------------PLLVCGCG----
010 YP_009268869.1 --
011 YP_009506251.1 ---QLNYWE--------------------------QVTHTAWPMMAS----
012 NP_955611.1 --
013 YP_008567619.1 ---QNPTPGSTKA-------------------LKQALHQANQWLSS----
014 NP_149413.1 KKDMFERTEHIMMA---------------------TQEPTLLCGCR----
015 YP_004442824.1 MH-PKVFMYRYIKE------------------------PKFKCGCG----
016 YP_001931967.1 --
017 YP_002455786.1 --
018 YP_009109694.1 --
019 NP_789739.1 --
020 NP_955619.1 --
021 YP_224289.1 -KPAGIQINMITEG------------------------PKLRCACG----
022 YP_009182100.1 MPSPIPPPFEYKTYRRLTEEQKSQLDWLTSSWHTVDQDTFFDVLKAMAKE
023 NP_705927.1 --
024 YP_009508577.1 --
025 YP_004442830.1 QVTAKEVICMADVK---------------------EIAP--LCNCS----
026 NP_758887.1 --
027 YP_009508406.1 --
028 YP_610965.1 ---YASMASSRSS-----------------------PTLDDDLWCQ----
029 YP_001931961.1 --
030 YP_241114.1 --
031 YP_006732334.1 --
032 YP_009041481.1 ---SSSSTLKTED-----------------------KESLIQLSGI----
033 YP_001036293.1 ---ALSKEIRS---------------------------QVAHQISA----
034 YP_605811.1 RKEHMVNTLLQLEE------------------------PQLHCGCKDHTT
035 NP_955577.1 --
036 YP_003208050.1 ---RHKEALWQST-----------------------PKYEGPTGLM----
037 NP_789740.1 --
038 YP_004442839.1 KDEHPVNVLTLDEE------------------------TRLRCSCQDPIT
039 YP_009507791.1 --
040 YP_004581513.1 ---LRELSMKRPL-------------------EDKEDQGPWSILLL----
041 NP_043933.1 --
042 YP_009507248.1 --
043 YP_002117531.1 --QERIFHLTEVEG------------------------PALKCACN----
044 YP_004347415.1 --
045 NP_056891.1 --
046 YP_009513242.1 --
047 YP_009352866.1 ---SISSTLTRET-----------------------KESLIQLSGT----
048 YP_009165750.1 --
049 NP_040840.1 --
050 YP_004222728.1 --
051 YP_009506264.1 ------HIKVEEEA------------------------YMLAWPC-----
052 YP_567050.1 --
053 YP_009508571.1 --
054 NP_056803.1 --
055 NP_955564.1 --
056 YP_009508411.1 ---LLTHPSHE---------------------------ATMKGVKL----
057 YP_009140788.1 ---LMKEEQFKEK-------------------LPSLINKMIGCFSN----
058 YP_009508410.1 --
059 NP_040333.1 --
060 YP_008992013.1 --DFTKKFL---
061 NP_041734.1 ---HLSFAG--------------------------LAIPIAWPMMGS----
062 NP_056895.1 --
063 YP_009109689.1 --
064 YP_002519387.1 --
065 YP_595725.1 KKDMFERTEQAFML---------------------TEEPVLLCACR----
066 YP_009508551.1 --
067 YP_001497148.1 --
068 NP_041186.1 --
069 NP_663784.1 KSSRNTGILEPNTRKRMALLSCSKINLVYRKVLDRCYPRLCRHPNT----
070 YP_009508443.1 ---VTSRPHAE---------------------------AMKKLATM----

```
071 YP_003987465.1   ------AVHVLTHK------------------------PILKCGCG----
072 NP_861410.1      --------------------------------------------------
073 YP_009345071.1   ---TQALMTSYLN-------------------TRSRSKTISKHAPN----
074 YP_009508582.1   --------------------------------------------------
075 NP_569141.1      --------------------------------------------------
076 YP_002916057.1   ---LREPPLKQEQ-------------------EG-TGEGPWGIEQL----
077 NP_777384.2      --------------------------------------------------
078 YP_233110.1      KEEHTVNTLLQLEE------------------------PQLHCGCRNYET
079 NP_542258.1      --------------------------------------------------
080 YP_009130664.1   ---AKPQPGKN--------------------------FQLTKLISS----
081 YP_009508566.1   --------------------------------------------------
082 YP_006607892.1   --------------------------------------------------
083 YP_009506270.1   FN-HHVNVLTIKEE------------------------PVFKCGCN----
084 NP_042513.1      --------------------------------------------------
085 YP_006907834.1   --------------------------------------------------
086 YP_004442827.1   SYEKIEICCMTETN---------------------ELAP--KCNCG----
087 YP_009408594.1   --------YCGLQ-----------------------PIIETGLKTS----
088 YP_009508561.1   --------------------------------------------------
089 NP_056907.1      --------------------------------------------------
090 YP_002308474.1   --------------------------------------------------
091 YP_007761644.1   --------------------------------------------------
092 NP_040550.1      --------------------------------------------------
093 NP_758808.1      AIHFLEDDKIEPT------------------AESSTPITLDEFSRK----
094 YP_009345075.1   ICLKTTSGICNKP-----------------------ATYHRTCNST----
095 NP_068729.1      --------------------------------------------------
096 NP_047255.1      --------------------------------------------------
097 YP_006273075.1   ---LMEDKQCRKQ-------------------LLPLINKMTNCFNN----
098 YP_009508888.1   --------------------------------------------------
099 NP_395469.1      --------------------------------------------------
100 YP_009508546.1   --------------------------------------------------
101 NP_056762.1      --------------------------------------------------
102 YP_009508556.1   --------------------------------------------------
103 NP_569153.1      ---HVEYVSLLIR-------------------SADRITRSLCFMRD----
104 NP_043924.1      FQRGSDSPSGGQ--------------------------------------
105 YP_009243641.1   --------------------------------------------------
106 YP_004300274.1   --------------------------------------------------
107 NP_056848.1      --------------------------------------------------
108 YP_006495799.1   ---PKERRILMDH-----------------------ICGMLEAQAQ----
109 YP_009508409.1   --------------------------------------------------
110 NP_687035.1      --------------------------------------------------
111 NP_045937.2      --------------------------------------------------
112 NP_612577.1      --------------------------------------------------
113 NP_619548.1      --------------------------------------------------
114 NP_056880.1      --------------------------------------------------
115 YP_009121747.1   QVLCIDTIKSEPTPPQYALKNQVSTQELPNNTKEKPPNKPSELCAT----
116 YP_009113237.2   ---KLKSKWSTEK-------------------IKELINLLIYNIKN----
117 YP_009508408.1   ---VMSRPNQA---------------------------AMTKLAEL----
118 NP_955579.1      --------------------------------------------------
119 YP_009229919.1   LAYHCIQGPSHKS-----------------------SSTESSLASS----
120 YP_004442833.1   EYKKLEVVCMAQTA---------------------DEVSPLLCNCE----
121 NP_862833.2      --------------------------------------------------
122 NP_040563.1      --------------------------------------------------
123 YP_443922.1      --------------------------------------------------
124 NP_057933.2      --------------------------------------------------
125 YP_009116631.1   ---P-AKKEVQEE-----------------------ISCFLEAALR----
126 YP_009513249.1   ----ETSVDSS---------------------------------------
127 YP_223871.1      --------------------------------------------------
128 NP_127504.1      LERRTFYELQVFKKYGGSILRPFGVDPEYPFAHIFIPNPTDFSEDLLWMF
129 NP_044929.1      TWSCEDRCGHRWKECGNCIPQDGSSDDASAVAAVEI--------------
130 NP_057860.1      --------------------------------------------------
131 NP_040973.1      --------------------------------------------------
132 NP_777317.1      EDENTTRVGQTYSVGTED-----------------LIQPTLNCACK----
133 NP_054716.1      ----QDQRGHT---------------------------------------
134 YP_001856242.1   --------------------------------------------------
135 NP_954565.2      --------------------------------------------------
136 NP_569150.1      YRLHQINVLTLSEE------------------------PVFKCGCN----
137 NP_659397.1      --------------------------------------------------
138 YP_009513211.1   --------------------------------------------------
139 YP_233107.1      -NKYQVNFMTWEQP-------------------------QLKCACG----
140 YP_009109692.1   --------------------------------------------------
141 NP_057849.4      --------------------------------------------------
142 NP_049560.1      QPVCSWTLDLLALSADQALQPPCPNLVSYSSYHATYSLYLFPHWIKKPNR
143 YP_009506267.1   ---IMKKPHQG---------------------------AIARIATL----
```

```
144 NP_039820.1       --------------------------------------------------
145 NP_056790.1       --------------------------------------------------
146 YP_002321513.1    ---SLKDTSLQIE----------------------LQESFLKKIYY----
147 NP_955591.1       --------------------------------------------------
148 YP_001956722.2    --------------------------------------------------
149 NP_056902.1       --------------------------------------------------
150 NP_056886.1       --------------------------------------------------
151 NP_041261.1       --------------------------------------------------


001 YP_004442836.1    ---KPARLHTSRTSRNPGREFYSCENK--ACFTWVWKDQIDE---YVQEV
002 NP_056728.1       --------------------------------------------------
003 YP_009508537.1    --------------------------------------------------
004 YP_004732983.2    ---LNHGR-----------PNTTWYSRTKPKKSKARKQAQVQLRFDVSND
005 YP_009508407.1    --------------------------------------------------
006 NP_040939.1       --------------------------------------------------
007 YP_003864102.1    --------------------------------------------------
008 YP_009002585.1    ---TMNSWTEKSLSSTQKEALLSDTRITSTNKWPLLKDKPVHKQNKHSN-
009 YP_003284237.1    ---IPAVQRESRTSRNPNRKFVTCRDNR--CRCWWWSDNIDDYTRRMIDL
010 YP_009268869.1    --------------------------------------------------
011 YP_009506251.1    ---CSNEGIPLHMAPSPWQLNMPSPDSSIRSRKKLPRKRSTHYET-----
012 NP_955611.1       --------------------------------------------------
013 YP_008567619.1    ---ISSTKMPLKDSQGLTAPAHENGGTTCASSKSLKEKPPKKPKKRWKSS
014 NP_149413.1       ---KPAIMLTSGTRLNPRRRFYKCAMNI--CHCWYWADLLEE---YVQER
015 YP_004442824.1    ---RKAEEVVSHTQKNPDRKFYRCKRS--NCHTWVWKDQIDN---YVQNY
016 YP_001931967.1    --------------------------------------------------
017 YP_002455786.1    --------------------------------------------------
018 YP_009109694.1    --------------------------------------------------
019 NP_789739.1       --------------------------------------------------
020 NP_955619.1       --------------------------------------------------
021 YP_224289.1       ---KDAEIAVSHTSRNPDRPFYKCQRNL--CHIWIWKDLVDD---YFQNL
022 YP_009182100.1    FEGLNKLKNGNPAKRVKTSREAKLARWKAQPKKFTFFEEWRTILLRRLRN
023 NP_705927.1       --------------------------------------------------
024 YP_009508577.1    --------------------------------------------------
025 YP_004442830.1    ---KPAMLRTSMTSRSPNRQFWNCAEKR--CHAWWWYDNLEG---YIKNE
026 NP_758887.1       --------------------------------------------------
027 YP_009508406.1    --------------------------------------------------
028 YP_610965.1       ---EQSKKAPLSTHCRGTGEHSTSLRPTNGEPCGPSSALQSSS--RNSRW
029 YP_001931961.1    --------------------------------------------------
030 YP_241114.1       --------------------------------------------------
031 YP_006732334.1    --------------------------------------------------
032 YP_009041481.1    ---VMDNWCALK-----SRHSSTALDSPGAQLKGKACPSKNMKSTDAAWP
033 YP_001036293.1    ---LLK-------IIDKGKSI-----------------------------
034 YP_605811.1       GERRNAVLLQSHTSANPYRWFYKCAQD--RCHTWIWKDILDQ---YAEDY
035 NP_955577.1       --------------------------------------------------
036 YP_003208050.1    ---IPLNEAQEWRAFARR-MRIAQAQKVDKELLGLLQMAQDKA--QYIED
037 NP_789740.1       --------------------------------------------------
038 YP_004442839.1    KQGMFAALLQSHTATNPGRWFYKCERN--RCHLWIWKDLLDQ---YANEY
039 YP_009507791.1    --------------------------------------------------
040 YP_004581513.1    ---TQPIEHLLKGSRNGQDRSKPITETGSMTSLKIALMILDLSPEVIIST
041 NP_043933.1       --------------------------------------------------
042 YP_009507248.1    --------------------------------------------------
043 YP_002117531.1    ---KEVLIKTSHTWRNPDRKFCVCPDGR--CHIWYWWDLLED---YVQQR
044 YP_004347415.1    --------------------------------------------------
045 NP_056891.1       --------------------------------------------------
046 YP_009513242.1    --------------------------------------------------
047 YP_009352866.1    ---VMETWCSQR-----LQLNSTAQNSQGQQQK-KVYPSKQQLNIGQGCP
048 YP_009165750.1    --------------------------------------------------
049 NP_040840.1       --------------------------------------------------
050 YP_004222728.1    --------------------------------------------------
051 YP_009506264.1    ----------------PKPNLLCSQDS--------------------LDI
052 YP_567050.1       --------------------------------------------------
053 YP_009508571.1    --------------------------------------------------
054 NP_056803.1       --------------------------------------------------
055 NP_955564.1       --------------------------------------------------
056 YP_009508411.1    ---LNQLMNITDSAIASEESEGLQLSRKKTKREDFSPC------------
057 YP_009140788.1    ---TRISQMKDE-PFIMKEYLSPMNKKSESSLELLKLSQQRKQSKLLRNI
058 YP_009508410.1    --------------------------------------------------
059 NP_040333.1       --------------------------------------------------
060 YP_008992013.1    --------------------------------------------------
061 NP_041734.1       ---YNKRRTPLLTGQSLWQRNKPSQHSSTASKSRQPRKHYWPYVT-----
062 NP_056895.1       --------------------------------------------------
```

```
063 YP_009109689.1   --------------------------------------------------
064 YP_002519387.1   --------------------------------------------------
065 YP_595725.1      ---KPAIQLVSRTSANPGRKFFKCAMNK--CHCWYWADLIEE---HIQDR
066 YP_009508551.1   --------------------------------------------------
067 YP_001497148.1   --------------------------------------------------
068 NP_041186.1      --------------------------------------------------
069 NP_663784.1      --------------------------------------------------
070 YP_009508443.1   ---IQG-------VLDSTHPRE----------------------------
071 YP_003987465.1   ---KTAIRKMSRTSRNPNRHYYCCEQEK--CHCWWWED-------HLLQF
072 NP_861410.1      --------------------------------------------------
073 YP_009345071.1   ---QGQWTNSSNLGPKSRTRSTNCTPKCVKNSQKYTQSLNSTGTQLDPRQ
074 YP_009508582.1   --------------------------------------------------
075 NP_569141.1      --------------------------------------------------
076 YP_002916057.1   ---TLPLERLLKGLKTGPYKNKLIIGTGLMNSLKIALRILDLSPEVIIST
077 NP_777384.2      --------------------------------------------------
078 YP_233110.1      GERRNAILLQSHTSANPNRWFYKCAEN--KCHIWIWKDILDQ---YAEDY
079 NP_542258.1      --------------------------------------------------
080 YP_009130664.1   ---LAG-----DGKRGRQLLQNTKGNGTASKTHDIVA-------------
081 YP_009508566.1   --------------------------------------------------
082 YP_006607892.1   --------------------------------------------------
083 YP_009506270.1   ---KPARLKTSRTSRNPNRQFYSCETK--SCFTWVWKDQIDT---FV---
084 NP_042513.1      --------------------------------------------------
085 YP_006907834.1   --------------------------------------------------
086 YP_004442827.1   ---QPAVLKISQTSKNPGRRFWTCEQRK--CHAWWWDDHLED---YIHAR
087 YP_009408594.1   ---LRGP------------LRQINMTRSPNGLEQLTRSPKMLSWQHWKKC
088 YP_009508561.1   --------------------------------------------------
089 NP_056907.1      --------------------------------------------------
090 YP_002308474.1   --------------------------------------------------
091 YP_007761644.1   --------------------------------------------------
092 NP_040550.1      --------------------------------------------------
093 NP_758808.1      ---RFQEHTDLLEEFQLTLLQINLLEAS---LHERLMKCQ-SYATRDNFW
094 YP_009345075.1   ---ASMKK-----------EQXTACRTNSXRYQNSCRNSEPWSTDHWPTI
095 NP_068729.1      --------------------------------------------------
096 NP_047255.1      --------------------------------------------------
097 YP_006273075.1   ---TRTLLRKEKGSFDQEEYLLPETMNSENNSGRLKKNLQEEPLKPWNNT
098 YP_009508888.1   --------------------------------------------------
099 NP_395469.1      --------------------------------------------------
100 YP_009508546.1   --------------------------------------------------
101 NP_056762.1      --------------------------------------------------
102 YP_009508556.1   --------------------------------------------------
103 NP_569153.1      ---SSHSRIYSCRPGKEPMKALICEQKSCQSKGDLGNTRTVHSKSAFNQQ
104 NP_043924.1      --------------------------------------------------
105 YP_009243641.1   --------------------------------------------------
106 YP_004300274.1   --------------------------------------------------
107 NP_056848.1      --------------------------------------------------
108 YP_006495799.1   ---KAS-------------LHQHGLSP-----------------------
109 YP_009508409.1   --------------------------------------------------
110 NP_687035.1      --------------------------------------------------
111 NP_045937.2      --------------------------------------------------
112 NP_612577.1      --------------------------------------------------
113 NP_619548.1      --------------------------------------------------
114 NP_056880.1      --------------------------------------------------
115 YP_009121747.1   ---SRQFWTSKPKSVLQSHQQITIGETIGPMLRNRISKPENSYQNLRPYA
116 YP_009113237.2   ---SSTRSRPRWPQEECHLASMTNCEETEMSWNHEQKLKQSGHCRPIALS
117 YP_009508408.1   ---IYCSE--NIIKLQISSKEDTLRLQNKMKEDAYSPY------------
118 NP_955579.1      --------------------------------------------------
119 YP_009229919.1   ---MKTNWNEPRTAWPHCYQWPISIWSEFNEKRPRTTGPQIVSPRRRKRS
120 YP_004442833.1   ---QPAGRQMSKTSRNPGRWFWSCVQRK--CHAWWWDDHLED---YIETQ
121 NP_862833.2      --------------------------------------------------
122 NP_040563.1      --------------------------------------------------
123 YP_443922.1      --------------------------------------------------
124 NP_057933.2      --------------------------------------------------
125 YP_009116631.1   ---RAKRSVTTHQSEPRHVLWQKWQNPEGWLYCDERRSSTALPNTSATRS
126 YP_009513249.1   --------------------------------------------------
127 YP_223871.1      --------------------------------------------------
128 NP_127504.1      WYLLNHFHILMKFRCSKFSKIDQVNPWMLKFLLWFNNHNYWASLFKCMKG
129 NP_044929.1      --------------------------------------------------
130 NP_057860.1      --------------------------------------------------
131 NP_040973.1      --------------------------------------------------
132 NP_777317.1      ---IKAQKLISRTTRNPDRAYYRCGLSPPKCYTWIWEDILEA---YVTER
133 NP_054716.1      --------------------------------------------------
134 YP_001856242.1   --------------------------------------------------
135 NP_954565.2      --------------------------------------------------
```

136 NP_569150.1 ---KPAKLKISRTSRNPDREFYSCETN--TCFTWVWKDQIDS---FVQEK
137 NP_659397.1 --
138 YP_009513211.1 --
139 YP_233107.1 ---ENAVLLTSHTSRNPGRRFYRCGTN--TCHVWYWADLIED---YIAQL
140 YP_009109692.1 --
141 NP_057849.4 --
142 NP_049560.1 NGGGYYSASYSDPCSLKCPYLGCQSWTCPYTGAISSPYWKFQQDVNFTQE
143 YP_009506267.1 ---VKK----MLEDVPTFPPRDA---------------------------
144 NP_039820.1 --------------DDHGRNLISAVIN---------------------DI
145 NP_056790.1 --
146 YP_002321513.1 ---LTPALECIDIPYIGGTMEMLSQPMLQQGDGYNTPGMSCLEETSLKET
147 NP_955591.1 --
148 YP_001956722.2 --
149 NP_056902.1 --
150 NP_056886.1 --
151 NP_041261.1 --

001 YP_004442836.1 MTWNDQVSQ--L
002 NP_056728.1 --
003 YP_009508537.1 --
004 YP_004732983.2 D---
005 YP_009508407.1 --
006 NP_040939.1 --
007 YP_003864102.1 --
008 YP_009002585.1 -NWKEYKDK---
009 YP_003284237.1 AQEEADYDEAM--------------------------------------R
010 YP_009268869.1 --
011 YP_009506251.1 --YKGSYIT---
012 NP_955611.1 --
013 YP_008567619.1 STSTN---
014 NP_149413.1 IEDFMVENFDKKA--------------------------KLDEPSSSNVH
015 YP_004442824.1 INWLKESEEPEDEGYDPINRPRSLDSSPWSFDEPGSSQHQKEEEDERLLC
016 YP_001931967.1 --
017 YP_002455786.1 --
018 YP_009109694.1 --
019 NP_789739.1 --
020 NP_955619.1 --
021 YP_224289.1 TAWNRISEEHR--------------------------------------R
022 YP_009182100.1 WTNGATIMDEELYQRIINTPCRAQLQWWSQLNKLWQYQPPEQSFICKATC
023 NP_705927.1 --
024 YP_009508577.1 --
025 YP_004442830.1 VDKRLQR---IL
026 NP_758887.1 --
027 YP_009508406.1 --
028 YP_610965.1 RHVTGSERV---
029 YP_001931961.1 --
030 YP_241114.1 --
031 YP_006732334.1 --
032 YP_009041481.1 ASKTRNTKSSWYK-----------------------------------PP
033 YP_001036293.1 --
034 YP_605811.1 AAYTRIGLE---
035 NP_955577.1 --
036 YP_003208050.1 -QRAGNNWA---
037 NP_789740.1 --
038 YP_004442839.1 --YINMGLN---
039 YP_009507791.1 --
040 YP_004581513.1 YGLKEGSLE---
041 NP_043933.1 --
042 YP_009507248.1 --
043 YP_002117531.1 VTAAQLAECKN--------------------------------------F
044 YP_004347415.1 --
045 NP_056891.1 --
046 YP_009513242.1 --
047 YP_009352866.1 PLRTRNTRNSLPK-----------------------------------LQ
048 YP_009165750.1 --
049 NP_040840.1 --
050 YP_004222728.1 --
051 YP_009506264.1 ANDD--
052 YP_567050.1 --
053 YP_009508571.1 --
054 NP_056803.1 --

```
055 NP_955564.1       --------------------------------------------------
056 YP_009508411.1    --------------------------------------------------
057 YP_009140788.1    SESMQSKHW-----------------------------------------
058 YP_009508410.1    --------------------------------------------------
059 NP_040333.1       --------------------------------------------------
060 YP_008992013.1    --------------------------------------------------
061 NP_041734.1       --YRAYSTS-----------------------------------------
062 NP_056895.1       --------------------------------------------------
063 YP_009109689.1    --------------------------------------------------
064 YP_002519387.1    --------------------------------------------------
065 YP_595725.1       IDEFLKN---------------------------------LEVLKTGGVQ
066 YP_009508551.1    --------------------------------------------------
067 YP_001497148.1    --------------------------------------------------
068 NP_041186.1       --------------------------------------------------
069 NP_663784.1       --------------------------------------------------
070 YP_009508443.1    --------------------------------------------------
071 YP_003987465.1    AAERGALEQEA--------------------------------------L
072 NP_861410.1       --------------------------------------------------
073 YP_009345071.1    QGIMLGETI-----------------------------------------
074 YP_009508582.1    --------------------------------------------------
075 NP_569141.1       --------------------------------------------------
076 YP_002916057.1    YKLKEGYSE-----------------------------------------
077 NP_777384.2       --------------------------------------------------
078 YP_233110.1       ATYTRIGLE-----------------------------------------
079 NP_542258.1       --------------------------------------------------
080 YP_009130664.1    --------------------------------------------------
081 YP_009508566.1    --------------------------------------------------
082 YP_006607892.1    --------------------------------------------------
083 YP_009506270.1    --------------------------------------------------
084 NP_042513.1       --------------------------------------------------
085 YP_006907834.1    --------------------------------------------------
086 YP_004442827.1    IKMKLED-----------------------------------------IQ
087 YP_009408594.1    PKYWRPRKT-----------------------------------------
088 YP_009508561.1    --------------------------------------------------
089 NP_056907.1       --------------------------------------------------
090 YP_002308474.1    --------------------------------------------------
091 YP_007761644.1    --------------------------------------------------
092 NP_040550.1       --------------------------------------------------
093 NP_758808.1       GDWLPEARR-----------------------------------------
094 YP_009345075.1    PASRQKTTM-----------------------------------------
095 NP_068729.1       --------------------------------------------------
096 NP_047255.1       --------------------------------------------------
097 YP_006273075.1    GRFTHLKQP-----------------------------------------
098 YP_009508888.1    --------------------------------------------------
099 NP_395469.1       --------------------------------------------------
100 YP_009508546.1    --------------------------------------------------
101 NP_056762.1       --------------------------------------------------
102 YP_009508556.1    --------------------------------------------------
103 NP_569153.1       DNWWPSTST-----------------------------------------
104 NP_043924.1       --------------------------------------------------
105 YP_009243641.1    --------------------------------------------------
106 YP_004300274.1    --------------------------------------------------
107 NP_056848.1       --------------------------------------------------
108 YP_006495799.1    --------------------------------------------------
109 YP_009508409.1    --------------------------------------------------
110 NP_687035.1       --------------------------------------------------
111 NP_045937.2       --------------------------------------------------
112 NP_612577.1       --------------------------------------------------
113 NP_619548.1       --------------------------------------------------
114 NP_056880.1       --------------------------------------------------
115 YP_009121747.1    SSWAKSQSKPKES-----------------------------------KC
116 YP_009113237.2    --DKQSRTG-----------------------------------------
117 YP_009508408.1    --------------------------------------------------
118 NP_955579.1       --------------------------------------------------
119 YP_009229919.1    KGWSRPTRR-----------------------------------------
120 YP_004442833.1    VELRLAK-----------------------------------------IF
121 NP_862833.2       --------------------------------------------------
122 NP_040563.1       --------------------------------------------------
123 YP_443922.1       --------------------------------------------------
124 NP_057933.2       --------------------------------------------------
125 YP_009116631.1    SSPESTLRQQRPE-----------------------------------QP
126 YP_009513249.1    --------------------------------------------------
127 YP_223871.1       --------------------------------------------------
```

```
128 NP_127504.1        IKKYVVIWFYRPVNYYQGKLCALPHSSIVKWNHVSVLNDEDEYSELQRFI
129 NP_044929.1        --------------------------------------------------
130 NP_057860.1        --------------------------------------------------
131 NP_040973.1        --------------------------------------------------
132 NP_777317.1        IIRTQEQE--------------------------------------IDIL
133 NP_054716.1        --------------------------------------------------
134 YP_001856242.1     --------------------------------------------------
135 NP_954565.2        --------------------------------------------------
136 NP_569150.1        IRWEKKLEE----------------------------------------I
137 NP_659397.1        --------------------------------------------------
138 YP_009513211.1     --------------------------------------------------
139 YP_233107.1        SNLQNLDSG----------------------------------------Q
140 YP_009109692.1     --------------------------------------------------
141 NP_057849.4        --------------------------------------------------
142 NP_049560.1        VSRLTINLHFSKCGFPFSLLIDAPGYDPIWFLNTEPSQLPPTAPPLLPHS
143 YP_009506267.1     --------------------------------------------------
144 NP_039820.1        ITVLRR--------------------------------------------
145 NP_056790.1        --------------------------------------------------
146 YP_002321513.1     SQPALSTFTLG---------------------------------------
147 NP_955591.1        --------------------------------------------------
148 YP_001956722.2     --------------------------------------------------
149 NP_056902.1        --------------------------------------------------
150 NP_056886.1        --------------------------------------------------
151 NP_041261.1        --------------------------------------------------


001 YP_004442836.1     SE-----------EPEGHNEGC-------------------------TIE
002 NP_056728.1        --------------------------------------------------
003 YP_009508537.1     --------------------------------------------------
004 YP_004732983.2     --------------------------------------------------
005 YP_009508407.1     --------------------------------------------------
006 NP_040939.1        --------------------------------------------------
007 YP_003864102.1     --------------------------------------------------
008 YP_009002585.1     --------RRTTSGSQRQETTG-------------------------PVT
009 YP_003284237.1     RYHEELQEKKLDLMQINEALEA-------------------------AED
010 YP_009268869.1     --------------------------------------------------
011 YP_009506251.1     --------SATTCQALQQGTTG-------------------------RLT
012 NP_955611.1        --------------------------------------------------
013 YP_008567619.1     --------------------------------------------------
014 NP_149413.1        HDDYEEHRSSVIDRPRPTDDHFRPWGDVTYWLNKEAEECHTRGDNVEGAE
015 YP_004442824.1     KETMGEDYNESEQEVIRNNQAT-------------------------AIE
016 YP_001931967.1     --------------------------------------------------
017 YP_002455786.1     --------------------------------------------------
018 YP_009109694.1     --------------------------------------------------
019 NP_789739.1        --------------------------------------------------
020 NP_955619.1        --------------------------------------------------
021 YP_224289.1        EMAREEGQNLEEEDYWENVFNE-------------------------VFD
022 YP_009182100.1     FDNNHTMLKLKCPPINKLTPEFMDWFENGFIQSFALKRTSDYSLLPTDFI
023 NP_705927.1        --------------------------------------------------
024 YP_009508577.1     --------------------------------------------------
025 YP_004442830.1     AEQD-EEFEELCEIYKELEQQE--------------------ILRNQATD
026 NP_758887.1        --------------------------------------------------
027 YP_009508406.1     --------------------------------------------------
028 YP_610965.1        --------IMHGATHTPTCGTS-------------------------MNS
029 YP_001931961.1     --------------------------------------------------
030 YP_241114.1        --------------------------------------------------
031 YP_006732334.1     --------------------------------------------------
032 YP_009041481.1     QLTRSSARNWRPPGDAPVDETM-------------------------PTG
033 YP_001036293.1     --------------------------------------------------
034 YP_605811.1        ALSLEDWFEEPEPDPPDPVDRQ-------------------------KIE
035 NP_955577.1        --------------------------------------------------
036 YP_003208050.1     --------TDAWPNYLEDVNVI-------------------------HDV
037 NP_789740.1        --------------------------------------------------
038 YP_004442839.1     SLLLNMDWPDEESDPPDSPSPA-------------------------RTE
039 YP_009507791.1     --------------------------------------------------
040 YP_004581513.1     --------KACQEMWVHNSKQA-------------------------DRP
041 NP_043933.1        --------------------------------------------------
042 YP_009507248.1     --------------------------------------------------
043 YP_002117531.1     RQQLDNIKRQLDSDKRNRAHSI----------------------------
044 YP_004347415.1     --------------------------------------------------
045 NP_056891.1        --------------------------------------------------
046 YP_009513242.1     --------------------------------------------------
```

```
047 YP_009352866.1   QLTPSSVKSWKPPKAVQVAGII-------------------------TTE
048 YP_009165750.1   --------------------------------------------------
049 NP_040840.1      --------------------------------------------------
050 YP_004222728.1   --------------------------------------------------
051 YP_009506264.1   --------------------------------------------------
052 YP_567050.1      --------------------------------------------------
053 YP_009508571.1   --------------------------------------------------
054 NP_056803.1      --------------------------------------------------
055 NP_955564.1      --------------------------------------------------
056 YP_009508411.1   --------------------------------------------------
057 YP_009140788.1   --------NANHGVHQDETEII-------------------------GPT
058 YP_009508410.1   --------------------------------------------------
059 NP_040333.1      --------------------------------------------------
060 YP_008992013.1   --------------------------------------------------
061 NP_041734.1      --------RETIWPLLPLETTG-------------------------LAT
062 NP_056895.1      --------------------------------------------------
063 YP_009109689.1   --------------------------------------------------
064 YP_002519387.1   --------------------------------------------------
065 YP_595725.1      T--MEEELMKEVTKLKIEEQEFEEY------------QATPRAMSPVAAE
066 YP_009508551.1   --------------------------------------------------
067 YP_001497148.1   --------------------------------------------------
068 NP_041186.1      --------------------------------------------------
069 NP_663784.1      --------------------------------------------------
070 YP_009508443.1   --------------------------------------------------
071 YP_003987465.1   RMEELFDRLQFN---TNEPHGG-------------------------PLE
072 NP_861410.1      --------------------------------------------------
073 YP_009345071.1   --------SPTSKEKPKPPTIT-------------------------SVS
074 YP_009508582.1   --------------------------------------------------
075 NP_569141.1      --------------------------------------------------
076 YP_002916057.1   --------MGSQEMSVHNLQLI-------------------------DSP
077 NP_777384.2      --------------------------------------------------
078 YP_233110.1      ALNLEDWFEEPEPDPPNPVDRQ-------------------------RIE
079 NP_542258.1      --------------------------------------------------
080 YP_009130664.1   --------------------------------------------------
081 YP_009508566.1   --------------------------------------------------
082 YP_006607892.1   --------------------------------------------------
083 YP_009506270.1   --------------------------------------------------
084 NP_042513.1      --------------------------------------------------
085 YP_006907834.1   --------------------------------------------------
086 YP_004442827.1   KEQNPYDFEDWDDLLKET-------------------------QEEYKLE
087 YP_009408594.1   --------IFKCRQ------------------------------------
088 YP_009508561.1   --------------------------------------------------
089 NP_056907.1      --------------------------------------------------
090 YP_002308474.1   --------------------------------------------------
091 YP_007761644.1   --------------------------------------------------
092 NP_040550.1      --------------------------------------------------
093 NP_758808.1      --------DLLQIQLAKEIIEK-------------------------VRE
094 YP_009345075.1   --------LGTTKKHSNMHRVR-------------------------F--
095 NP_068729.1      --------------------------------------------------
096 NP_047255.1      --------------------------------------------------
097 YP_006273075.1   --------NVHHGVHPEGMGII-------------------------GLI
098 YP_009508888.1   --------------------------------------------------
099 NP_395469.1      --------------------------------------------------
100 YP_009508546.1   --------------------------------------------------
101 NP_056762.1      --------------------------------------------------
102 YP_009508556.1   --------------------------------------------------
103 NP_569153.1      --------NSLTSEAKLQGTTH-------------------------MPI
104 NP_043924.1      --------------------------------------------------
105 YP_009243641.1   --------------------------------------------------
106 YP_004300274.1   --------------------------------------------------
107 NP_056848.1      --------------------------------------------------
108 YP_006495799.1   --------------------------------------------------
109 YP_009508409.1   --------------------------------------------------
110 NP_687035.1      --------------------------------------------------
111 NP_045937.2      --------------------------------------------------
112 NP_612577.1      --------------------------------------------------
113 NP_619548.1      --------------------------------------------------
114 NP_056880.1      --------------------------------------------------
115 YP_009121747.1   PGAHAWDEEIARRQQLHNRSMK-------------------------YNQ
116 YP_009113237.2   --------VQGTHPGITSMVTH-------------------------YPP
117 YP_009508408.1   --------------------------------------------------
118 NP_955579.1      --------------------------------------------------
119 YP_009229919.1   --------SQGTSPSSEEKDVR-------------------------DDV
```

```
120 YP_004442833.1    KEQGTQEYEESMEKLVIKDNSPLPYQE-----NPEDAYQADFIQEDYQPE
121 NP_862833.2       --------------------------------------------------
122 NP_040563.1       --------------------------------------------------
123 YP_443922.1       --------------------------------------------------
124 NP_057933.2       --------------------------------------------------
125 YP_009116631.1    GTTGMLTSHQHWNDEPPQHGSS-------------------------WPL
126 YP_009513249.1    --------------------------------------------------
127 YP_223871.1       --------------------------------------------------
128 NP_127504.1       FQENKCIPKEIWPGSSGSWNYGNSDHPHGQWIRDALREYREMNDYFQDAQ
129 NP_044929.1       --------------------------------------------------
130 NP_057860.1       --------------------------------------------------
131 NP_040973.1       --------------------------------------------------
132 NP_777317.1       SDDNEAGINDLQNTTYDPGDDN-------------------------NWE
133 NP_054716.1       --------------------------------------------------
134 YP_001856242.1    --------------------------------------------------
135 NP_954565.2       --------------------------------------------------
136 NP_569150.1       SEDSLWEELLREQENLRAEQEY-------------------------LIE
137 NP_659397.1       --------------------------------------------------
138 YP_009513211.1    --------------------------------------------------
139 YP_233107.1       ADDEGWAYQTEDLINPEDLANS-------------------------DID
140 YP_009109692.1    --------------------------------------------------
141 NP_057849.4       --------------------------------------------------
142 NP_049560.1       NLDHILEPSIPWKSKLLTPVQLTLQSTNYTCIVCIDRASLSTWHVLYFPN
143 YP_009506267.1    --------------------------------------------------
144 NP_039820.1       --------------------------------------------------
145 NP_056790.1       --------------------------------------------------
146 YP_002321513.1    ---------KTEFVKPEKPSSS-------------------------YVT
147 NP_955591.1       --------------------------------------------------
148 YP_001956722.2    --------------------------------------------------
149 NP_056902.1       --------------------------------------------------
150 NP_056886.1       --------------------------------------------------
151 NP_041261.1       --------------------------------------------------

001 YP_004442836.1    DAFDLLDVSNDDQWARS---------------------------------
002 NP_056728.1       --------------------------------------------------
003 YP_009508537.1    --------------------------------------------------
004 YP_004732983.2    --------------------------------------------------
005 YP_009508407.1    --------------------------------------------------
006 NP_040939.1       --------------------------------------------------
007 YP_003864102.1    --------------------------------------------------
008 YP_009002585.1    GYHPP---------------------------------------------
009 YP_003284237.1    QDRREGVISDADFEPNVIRDD-----------------------------
010 YP_009268869.1    --------------------------------------------------
011 YP_009506251.1    DSH-----------------------------------------------
012 NP_955611.1       --------------------------------------------------
013 YP_008567619.1    --------------------------------------------------
014 NP_149413.1       DAVDLTDVSNDDQWRRS---------------------------------
015 YP_004442824.1    DALDLLDVSNDDQWGRS---------------------------------
016 YP_001931967.1    --------------------------------------------------
017 YP_002455786.1    --------------------------------------------------
018 YP_009109694.1    --------------------------------------------------
019 NP_789739.1       --------------------------------------------------
020 NP_955619.1       --------------------------------------------------
021 YP_224289.1       HEEITEFYPDGGDPG-----------------------------------
022 YP_009182100.1    KVIEQIWAIKDIGNTLFIMMYSAPGEWNYKDCSYYYPYHLVRIYDRVPSV
023 NP_705927.1       --------------------------------------------------
024 YP_009508577.1    --------------------------------------------------
025 YP_004442830.1    DPLDWNEVSNDD--------------------------------------
026 NP_758887.1       --------------------------------------------------
027 YP_009508406.1    --------------------------------------------------
028 YP_610965.1       SY-ASLDNSKS---------------------------------------
029 YP_001931961.1    --------------------------------------------------
030 YP_241114.1       --------------------------------------------------
031 YP_006732334.1    --------------------------------------------------
032 YP_009041481.1    TSSL----------------------------------------------
033 YP_001036293.1    --------------------------------------------------
034 YP_605811.1       DILDLQDVSNDD--------------------------------------
035 NP_955577.1       --------------------------------------------------
036 YP_003208050.1    RR-KIQNITRH---------------------------------------
037 NP_789740.1       --------------------------------------------------
038 YP_004442839.1    DIFDLANVSNDD--------------------------------------
```

039 YP_009507791.1 --
040 YP_004581513.1 YCMRRPRRPGEA--------------------------------------
041 NP_043933.1 --
042 YP_009507248.1 --
043 YP_002117531.1 --
044 YP_004347415.1 --
045 NP_056891.1 --
046 YP_009513242.1 --
047 YP_009352866.1 ICSQPWKKGKG---------------------------------------
048 YP_009165750.1 --
049 NP_040840.1 --
050 YP_004222728.1 --
051 YP_009506264.1 --
052 YP_567050.1 --
053 YP_009508571.1 --
054 NP_056803.1 --
055 NP_955564.1 --
056 YP_009508411.1 --
057 YP_009140788.1 TFQMSRDQTRISKGSFTISEKNCLMP------------------------
058 YP_009508410.1 --
059 NP_040333.1 --
060 YP_008992013.1 --
061 NP_041734.1 DCQLPNKTQPP---------------------------------------
062 NP_056895.1 --
063 YP_009109689.1 --
064 YP_002519387.1 --
065 YP_595725.1 DVLDLQDVSNDD--------------------------------------
066 YP_009508551.1 --
067 YP_001497148.1 --
068 NP_041186.1 --
069 NP_663784.1 --
070 YP_009508443.1 --
071 YP_003987465.1 DDDQASLLDAVGL-------------------------------------
072 NP_861410.1 --
073 YP_009345071.1 --
074 YP_009508582.1 --
075 NP_569141.1 --
076 YP_002916057.1 YCMKRPRRPGEAS-------------------------------------
077 NP_777384.2 --
078 YP_233110.1 DILDLLNVSNDD--------------------------------------
079 NP_542258.1 --
080 YP_009130664.1 --
081 YP_009508566.1 --
082 YP_006607892.1 --
083 YP_009506270.1 --
084 NP_042513.1 --
085 YP_006907834.1 --
086 YP_004442827.1 DALDLDDFMNDDQWRRA---------------------------------
087 YP_009408594.1 --
088 YP_009508561.1 --
089 NP_056907.1 --
090 YP_002308474.1 --
091 YP_007761644.1 --
092 NP_040550.1 --
093 NP_758808.1 KLHSI---
094 YP_009345075.1 --
095 NP_068729.1 --
096 NP_047255.1 --
097 YP_006273075.1 ACQALGDMTQD---------------------------------------
098 YP_009508888.1 --
099 NP_395469.1 --
100 YP_009508546.1 --
101 NP_056762.1 --
102 YP_009508556.1 --
103 NP_569153.1 GYPHAIGTTSNCVKWSSY--------------------------------
104 NP_043924.1 --
105 YP_009243641.1 --
106 YP_004300274.1 --
107 NP_056848.1 --
108 YP_006495799.1 --
109 YP_009508409.1 --
110 NP_687035.1 --
111 NP_045937.2 --

```
112 NP_612577.1         --------------------------------------------------
113 NP_619548.1         --------------------------------------------------
114 NP_056880.1         --------------------------------------------------
115 YP_009121747.1      ALWLIDESWGPPLSMPRYCKL-----------------------------
116 YP_009113237.2      CCTGMNNSPPTRML------------------------------------
117 YP_009508408.1      --------------------------------------------------
118 NP_955579.1         --------------------------------------------------
119 YP_009229919.1      KYPKTSGMTKNI--------------------------------------
120 YP_004442833.1      DVLDLDDFTNDDQWRRS---------------------------------
121 NP_862833.2         --------------------------------------------------
122 NP_040563.1         --------------------------------------------------
123 YP_443922.1         --------------------------------------------------
124 NP_057933.2         --------------------------------------------------
125 YP_009116631.1      TRNSPRVRM-----------------------------------------
126 YP_009513249.1      --------------------------------------------------
127 YP_223871.1         --------------------------------------------------
128 NP_127504.1         DPYPAYSKVDLTQEELNTLRITRSYGSSSEDADMVKRSIYTVQSNIVKDS
129 NP_044929.1         --------------------------------------------------
130 NP_057860.1         --------------------------------------------------
131 NP_040973.1         --------------------------------------------------
132 NP_777317.1         DAVDISAYDSIDAFWDAHT-------------------------------
133 NP_054716.1         --------------------------------------------------
134 YP_001856242.1      --------------------------------------------------
135 NP_954565.2         --------------------------------------------------
136 NP_569150.1         DALDLLDISNDD--------------------------------------
137 NP_659397.1         --------------------------------------------------
138 YP_009513211.1      --------------------------------------------------
139 YP_233107.1         DPPEDSGLFHRHDD------------------------------------
140 YP_009109692.1      --------------------------------------------------
141 NP_057849.4         --------------------------------------------------
142 NP_049560.1         VSVPSSPSTPLLYPSLALPAPHLTLPFNWTHCFNPQIQAIVSSPCHNSLI
143 YP_009506267.1      --------------------------------------------------
144 NP_039820.1         --------------------------------------------------
145 NP_056790.1         --------------------------------------------------
146 YP_002321513.1      TMRKK---------------------------------------------
147 NP_955591.1         --------------------------------------------------
148 YP_001956722.2      --------------------------------------------------
149 NP_056902.1         --------------------------------------------------
150 NP_056886.1         --------------------------------------------------
151 NP_041261.1         --------------------------------------------------
```

```
001 YP_004442836.1      --------------------------------------------------
002 NP_056728.1         --------------------------------------------------
003 YP_009508537.1      --------------------------------------------------
004 YP_004732983.2      --------------------------------------------------
005 YP_009508407.1      --------------------------------------------------
006 NP_040939.1         --------------------------------------------------
007 YP_003864102.1      --------------------------------------------------
008 YP_009002585.1      --------------------------------------------------
009 YP_003284237.1      --------------------------------------------------
010 YP_009268869.1      --------------------------------------------------
011 YP_009506251.1      --------------------------------------------------
012 NP_955611.1         --------------------------------------------------
013 YP_008567619.1      --------------------------------------------------
014 NP_149413.1         --------------------------------------------------
015 YP_004442824.1      --------------------------------------------------
016 YP_001931967.1      --------------------------------------------------
017 YP_002455786.1      --------------------------------------------------
018 YP_009109694.1      --------------------------------------------------
019 NP_789739.1         --------------------------------------------------
020 NP_955619.1         --------------------------------------------------
021 YP_224289.1         --------------------------------------------------
022 YP_009182100.1      TRDTILEGEIFPHLRSQEQIQRDEEMGMYEPLYCPIDDWLSRDPRHVSKW
023 NP_705927.1         --------------------------------------------------
024 YP_009508577.1      --------------------------------------------------
025 YP_004442830.1      --------------------------------------------------
026 NP_758887.1         --------------------------------------------------
027 YP_009508406.1      --------------------------------------------------
028 YP_610965.1         --------------------------------------------------
029 YP_001931961.1      --------------------------------------------------
030 YP_241114.1         --------------------------------------------------
```

```
031 YP_006732334.1    --------------------------------------------------
032 YP_009041481.1    --------------------------------------------------
033 YP_001036293.1    --------------------------------------------------
034 YP_605811.1       --------------------------------------------------
035 NP_955577.1       --------------------------------------------------
036 YP_003208050.1    --------------------------------------------------
037 NP_789740.1       --------------------------------------------------
038 YP_004442839.1    --------------------------------------------------
039 YP_009507791.1    --------------------------------------------------
040 YP_004581513.1    --------------------------------------------------
041 NP_043933.1       --------------------------------------------------
042 YP_009507248.1    --------------------------------------------------
043 YP_002117531.1    --------------------------------------------------
044 YP_004347415.1    --------------------------------------------------
045 NP_056891.1       --------------------------------------------------
046 YP_009513242.1    --------------------------------------------------
047 YP_009352866.1    --------------------------------------------------
048 YP_009165750.1    --------------------------------------------------
049 NP_040840.1       --------------------------------------------------
050 YP_004222728.1    --------------------------------------------------
051 YP_009506264.1    --------------------------------------------------
052 YP_567050.1       --------------------------------------------------
053 YP_009508571.1    --------------------------------------------------
054 NP_056803.1       --------------------------------------------------
055 NP_955564.1       --------------------------------------------------
056 YP_009508411.1    --------------------------------------------------
057 YP_009140788.1    --------------------------------------------------
058 YP_009508410.1    --------------------------------------------------
059 NP_040333.1       --------------------------------------------------
060 YP_008992013.1    --------------------------------------------------
061 NP_041734.1       --------------------------------------------------
062 NP_056895.1       --------------------------------------------------
063 YP_009109689.1    --------------------------------------------------
064 YP_002519387.1    --------------------------------------------------
065 YP_595725.1       --------------------------------------------------
066 YP_009508551.1    --------------------------------------------------
067 YP_001497148.1    --------------------------------------------------
068 NP_041186.1       --------------------------------------------------
069 NP_663784.1       --------------------------------------------------
070 YP_009508443.1    --------------------------------------------------
071 YP_003987465.1    --------------------------------------------------
072 NP_861410.1       --------------------------------------------------
073 YP_009345071.1    --------------------------------------------------
074 YP_009508582.1    --------------------------------------------------
075 NP_569141.1       --------------------------------------------------
076 YP_002916057.1    --------------------------------------------------
077 NP_777384.2       --------------------------------------------------
078 YP_233110.1       --------------------------------------------------
079 NP_542258.1       --------------------------------------------------
080 YP_009130664.1    --------------------------------------------------
081 YP_009508566.1    --------------------------------------------------
082 YP_006607892.1    --------------------------------------------------
083 YP_009506270.1    --------------------------------------------------
084 NP_042513.1       --------------------------------------------------
085 YP_006907834.1    --------------------------------------------------
086 YP_004442827.1    --------------------------------------------------
087 YP_009408594.1    --------------------------------------------------
088 YP_009508561.1    --------------------------------------------------
089 NP_056907.1       --------------------------------------------------
090 YP_002308474.1    --------------------------------------------------
091 YP_007761644.1    --------------------------------------------------
092 NP_040550.1       --------------------------------------------------
093 NP_758808.1       --------------------------------------------------
094 YP_009345075.1    --------------------------------------------------
095 NP_068729.1       --------------------------------------------------
096 NP_047255.1       --------------------------------------------------
097 YP_006273075.1    --------------------------------------------------
098 YP_009508888.1    --------------------------------------------------
099 NP_395469.1       --------------------------------------------------
100 YP_009508546.1    --------------------------------------------------
101 NP_056762.1       --------------------------------------------------
102 YP_009508556.1    --------------------------------------------------
103 NP_569153.1       --------------------------------------------------
```

```
104 NP_043924.1        --------------------------------------------------
105 YP_009243641.1     --------------------------------------------------
106 YP_004300274.1     --------------------------------------------------
107 NP_056848.1        --------------------------------------------------
108 YP_006495799.1     --------------------------------------------------
109 YP_009508409.1     --------------------------------------------------
110 NP_687035.1        --------------------------------------------------
111 NP_045937.2        --------------------------------------------------
112 NP_612577.1        --------------------------------------------------
113 NP_619548.1        --------------------------------------------------
114 NP_056880.1        --------------------------------------------------
115 YP_009121747.1     --------------------------------------------------
116 YP_009113237.2     --------------------------------------------------
117 YP_009508408.1     --------------------------------------------------
118 NP_955579.1        --------------------------------------------------
119 YP_009229919.1     --------------------------------------------------
120 YP_004442833.1     --------------------------------------------------
121 NP_862833.2        --------------------------------------------------
122 NP_040563.1        --------------------------------------------------
123 YP_443922.1        --------------------------------------------------
124 NP_057933.2        --------------------------------------------------
125 YP_009116631.1     --------------------------------------------------
126 YP_009513249.1     --------------------------------------------------
127 YP_223871.1        --------------------------------------------------
128 NP_127504.1        PRKRKGKAKSRSSTRSEKRRAKNKCKYRSLHGEDWWIELGYSTKPSTPSW
129 NP_044929.1        --------------------------------------------------
130 NP_057860.1        --------------------------------------------------
131 NP_040973.1        --------------------------------------------------
132 NP_777317.1        --------------------------------------------------
133 NP_054716.1        --------------------------------------------------
134 YP_001856242.1     --------------------------------------------------
135 NP_954565.2        --------------------------------------------------
136 NP_569150.1        --------------------------------------------------
137 NP_659397.1        --------------------------------------------------
138 YP_009513211.1     --------------------------------------------------
139 YP_233107.1        --------------------------------------------------
140 YP_009109692.1     --------------------------------------------------
141 NP_057849.4        --------------------------------------------------
142 NP_049560.1        LPPFSLSPVPTLGSRSRRAVPVAVWLVSALAMGAGMAGGITGSMSLASGR
143 YP_009506267.1     --------------------------------------------------
144 NP_039820.1        --------------------------------------------------
145 NP_056790.1        --------------------------------------------------
146 YP_002321513.1     --------------------------------------------------
147 NP_955591.1        --------------------------------------------------
148 YP_001956722.2     --------------------------------------------------
149 NP_056902.1        --------------------------------------------------
150 NP_056886.1        --------------------------------------------------
151 NP_041261.1        --------------------------------------------------


001 YP_004442836.1     --------------------------------------------------
002 NP_056728.1        --------------------------------------------------
003 YP_009508537.1     --------------------------------------------------
004 YP_004732983.2     --------------------------------------------------
005 YP_009508407.1     --------------------------------------------------
006 NP_040939.1        --------------------------------------------------
007 YP_003864102.1     --------------------------------------------------
008 YP_009002585.1     --------------------------------------------------
009 YP_003284237.1     --------------------------------------------------
010 YP_009268869.1     --------------------------------------------------
011 YP_009506251.1     --------------------------------------------------
012 NP_955611.1        --------------------------------------------------
013 YP_008567619.1     --------------------------------------------------
014 NP_149413.1        --------------------------------------------------
015 YP_004442824.1     --------------------------------------------------
016 YP_001931967.1     --------------------------------------------------
017 YP_002455786.1     --------------------------------------------------
018 YP_009109694.1     --------------------------------------------------
019 NP_789739.1        --------------------------------------------------
020 NP_955619.1        --------------------------------------------------
021 YP_224289.1        --------------------------------------------------
022 YP_009182100.1     RAFALMEIQDLINEDKGFLPRWHYIHSNQRFIFEVEGISMTRACESYSLF
```

```
023 NP_705927.1       --------------------------------------------------
024 YP_009508577.1    --------------------------------------------------
025 YP_004442830.1    --------------------------------------------------
026 NP_758887.1       --------------------------------------------------
027 YP_009508406.1    --------------------------------------------------
028 YP_610965.1       --------------------------------------------------
029 YP_001931961.1    --------------------------------------------------
030 YP_241114.1       --------------------------------------------------
031 YP_006732334.1    --------------------------------------------------
032 YP_009041481.1    --------------------------------------------------
033 YP_001036293.1    --------------------------------------------------
034 YP_605811.1       --------------------------------------------------
035 NP_955577.1       --------------------------------------------------
036 YP_003208050.1    --------------------------------------------------
037 NP_789740.1       --------------------------------------------------
038 YP_004442839.1    --------------------------------------------------
039 YP_009507791.1    --------------------------------------------------
040 YP_004581513.1    --------------------------------------------------
041 NP_043933.1       --------------------------------------------------
042 YP_009507248.1    --------------------------------------------------
043 YP_002117531.1    --------------------------------------------------
044 YP_004347415.1    --------------------------------------------------
045 NP_056891.1       --------------------------------------------------
046 YP_009513242.1    --------------------------------------------------
047 YP_009352866.1    --------------------------------------------------
048 YP_009165750.1    --------------------------------------------------
049 NP_040840.1       --------------------------------------------------
050 YP_004222728.1    --------------------------------------------------
051 YP_009506264.1    --------------------------------------------------
052 YP_567050.1       --------------------------------------------------
053 YP_009508571.1    --------------------------------------------------
054 NP_056803.1       --------------------------------------------------
055 NP_955564.1       --------------------------------------------------
056 YP_009508411.1    --------------------------------------------------
057 YP_009140788.1    --------------------------------------------------
058 YP_009508410.1    --------------------------------------------------
059 NP_040333.1       --------------------------------------------------
060 YP_008992013.1    --------------------------------------------------
061 NP_041734.1       --------------------------------------------------
062 NP_056895.1       --------------------------------------------------
063 YP_009109689.1    --------------------------------------------------
064 YP_002519387.1    --------------------------------------------------
065 YP_595725.1       --------------------------------------------------
066 YP_009508551.1    --------------------------------------------------
067 YP_001497148.1    --------------------------------------------------
068 NP_041186.1       --------------------------------------------------
069 NP_663784.1       --------------------------------------------------
070 YP_009508443.1    --------------------------------------------------
071 YP_003987465.1    --------------------------------------------------
072 NP_861410.1       --------------------------------------------------
073 YP_009345071.1    --------------------------------------------------
074 YP_009508582.1    --------------------------------------------------
075 NP_569141.1       --------------------------------------------------
076 YP_002916057.1    --------------------------------------------------
077 NP_777384.2       --------------------------------------------------
078 YP_233110.1       --------------------------------------------------
079 NP_542258.1       --------------------------------------------------
080 YP_009130664.1    --------------------------------------------------
081 YP_009508566.1    --------------------------------------------------
082 YP_006607892.1    --------------------------------------------------
083 YP_009506270.1    --------------------------------------------------
084 NP_042513.1       --------------------------------------------------
085 YP_006907834.1    --------------------------------------------------
086 YP_004442827.1    --------------------------------------------------
087 YP_009408594.1    --------------------------------------------------
088 YP_009508561.1    --------------------------------------------------
089 NP_056907.1       --------------------------------------------------
090 YP_002308474.1    --------------------------------------------------
091 YP_007761644.1    --------------------------------------------------
092 NP_040550.1       --------------------------------------------------
093 NP_758808.1       --------------------------------------------------
094 YP_009345075.1    --------------------------------------------------
095 NP_068729.1       --------------------------------------------------
```

```
096 NP_047255.1       --------------------------------------------------
097 YP_006273075.1    --------------------------------------------------
098 YP_009508888.1    --------------------------------------------------
099 NP_395469.1       --------------------------------------------------
100 YP_009508546.1    --------------------------------------------------
101 NP_056762.1       --------------------------------------------------
102 YP_009508556.1    --------------------------------------------------
103 NP_569153.1       --------------------------------------------------
104 NP_043924.1       --------------------------------------------------
105 YP_009243641.1    --------------------------------------------------
106 YP_004300274.1    --------------------------------------------------
107 NP_056848.1       --------------------------------------------------
108 YP_006495799.1    --------------------------------------------------
109 YP_009508409.1    --------------------------------------------------
110 NP_687035.1       --------------------------------------------------
111 NP_045937.2       --------------------------------------------------
112 NP_612577.1       --------------------------------------------------
113 NP_619548.1       --------------------------------------------------
114 NP_056880.1       --------------------------------------------------
115 YP_009121747.1    --------------------------------------------------
116 YP_009113237.2    --------------------------------------------------
117 YP_009508408.1    --------------------------------------------------
118 NP_955579.1       --------------------------------------------------
119 YP_009229919.1    --------------------------------------------------
120 YP_004442833.1    --------------------------------------------------
121 NP_862833.2       --------------------------------------------------
122 NP_040563.1       --------------------------------------------------
123 YP_443922.1       --------------------------------------------------
124 NP_057933.2       --------------------------------------------------
125 YP_009116631.1    --------------------------------------------------
126 YP_009513249.1    --------------------------------------------------
127 YP_223871.1       --------------------------------------------------
128 NP_127504.1       TQDSSSEPCV----------------------------------------
129 NP_044929.1       --------------------------------------------------
130 NP_057860.1       --------------------------------------------------
131 NP_040973.1       --------------------------------------------------
132 NP_777317.1       --------------------------------------------------
133 NP_054716.1       --------------------------------------------------
134 YP_001856242.1    --------------------------------------------------
135 NP_954565.2       --------------------------------------------------
136 NP_569150.1       --------------------------------------------------
137 NP_659397.1       --------------------------------------------------
138 YP_009513211.1    --------------------------------------------------
139 YP_233107.1       --------------------------------------------------
140 YP_009109692.1    --------------------------------------------------
141 NP_057849.4       --------------------------------------------------
142 NP_049560.1       SLLHEVDKDISQLTQAIVKNHKNLLKIAQYAAQNRRGLDLLFWEQGGLCK
143 YP_009506267.1    --------------------------------------------------
144 NP_039820.1       --------------------------------------------------
145 NP_056790.1       --------------------------------------------------
146 YP_002321513.1    --------------------------------------------------
147 NP_955591.1       --------------------------------------------------
148 YP_001956722.2    --------------------------------------------------
149 NP_056902.1       --------------------------------------------------
150 NP_056886.1       --------------------------------------------------
151 NP_041261.1       --------------------------------------------------


001 YP_004442836.1    --------------------------------------------------
002 NP_056728.1       --------------------------------------------------
003 YP_009508537.1    --------------------------------------------------
004 YP_004732983.2    --------------------------------------------------
005 YP_009508407.1    --------------------------------------------------
006 NP_040939.1       --------------------------------------------------
007 YP_003864102.1    --------------------------------------------------
008 YP_009002585.1    --------------------------------------------------
009 YP_003284237.1    --------------------------------------------------
010 YP_009268869.1    --------------------------------------------------
011 YP_009506251.1    --------------------------------------------------
012 NP_955611.1       --------------------------------------------------
013 YP_008567619.1    --------------------------------------------------
014 NP_149413.1       --------------------------------------------------
```

```
015 YP_004442824.1    --------------------------------------------------
016 YP_001931967.1    --------------------------------------------------
017 YP_002455786.1    --------------------------------------------------
018 YP_009109694.1    --------------------------------------------------
019 NP_789739.1       --------------------------------------------------
020 NP_955619.1       --------------------------------------------------
021 YP_224289.1       --------------------------------------------------
022 YP_009182100.1    QSLSLKVPMNYDTQLEIDCIQTLDAAQSEDPCDEPYDESDSYWQDMDDET
023 NP_705927.1       --------------------------------------------------
024 YP_009508577.1    --------------------------------------------------
025 YP_004442830.1    --------------------------------------------------
026 NP_758887.1       --------------------------------------------------
027 YP_009508406.1    --------------------------------------------------
028 YP_610965.1       --------------------------------------------------
029 YP_001931961.1    --------------------------------------------------
030 YP_241114.1       --------------------------------------------------
031 YP_006732334.1    --------------------------------------------------
032 YP_009041481.1    --------------------------------------------------
033 YP_001036293.1    --------------------------------------------------
034 YP_605811.1       --------------------------------------------------
035 NP_955577.1       --------------------------------------------------
036 YP_003208050.1    --------------------------------------------------
037 NP_789740.1       --------------------------------------------------
038 YP_004442839.1    --------------------------------------------------
039 YP_009507791.1    --------------------------------------------------
040 YP_004581513.1    --------------------------------------------------
041 NP_043933.1       --------------------------------------------------
042 YP_009507248.1    --------------------------------------------------
043 YP_002117531.1    --------------------------------------------------
044 YP_004347415.1    --------------------------------------------------
045 NP_056891.1       --------------------------------------------------
046 YP_009513242.1    --------------------------------------------------
047 YP_009352866.1    --------------------------------------------------
048 YP_009165750.1    --------------------------------------------------
049 NP_040840.1       --------------------------------------------------
050 YP_004222728.1    --------------------------------------------------
051 YP_009506264.1    --------------------------------------------------
052 YP_567050.1       --------------------------------------------------
053 YP_009508571.1    --------------------------------------------------
054 NP_056803.1       --------------------------------------------------
055 NP_955564.1       --------------------------------------------------
056 YP_009508411.1    --------------------------------------------------
057 YP_009140788.1    --------------------------------------------------
058 YP_009508410.1    --------------------------------------------------
059 NP_040333.1       --------------------------------------------------
060 YP_008992013.1    --------------------------------------------------
061 NP_041734.1       --------------------------------------------------
062 NP_056895.1       --------------------------------------------------
063 YP_009109689.1    --------------------------------------------------
064 YP_002519387.1    --------------------------------------------------
065 YP_595725.1       --------------------------------------------------
066 YP_009508551.1    --------------------------------------------------
067 YP_001497148.1    --------------------------------------------------
068 NP_041186.1       --------------------------------------------------
069 NP_663784.1       --------------------------------------------------
070 YP_009508443.1    --------------------------------------------------
071 YP_003987465.1    --------------------------------------------------
072 NP_861410.1       --------------------------------------------------
073 YP_009345071.1    --------------------------------------------------
074 YP_009508582.1    --------------------------------------------------
075 NP_569141.1       --------------------------------------------------
076 YP_002916057.1    --------------------------------------------------
077 NP_777384.2       --------------------------------------------------
078 YP_233110.1       --------------------------------------------------
079 NP_542258.1       --------------------------------------------------
080 YP_009130664.1    --------------------------------------------------
081 YP_009508566.1    --------------------------------------------------
082 YP_006607892.1    --------------------------------------------------
083 YP_009506270.1    --------------------------------------------------
084 NP_042513.1       --------------------------------------------------
085 YP_006907834.1    --------------------------------------------------
086 YP_004442827.1    --------------------------------------------------
087 YP_009408594.1    --------------------------------------------------
```

```
088 YP_009508561.1   --------------------------------------------------
089 NP_056907.1      --------------------------------------------------
090 YP_002308474.1   --------------------------------------------------
091 YP_007761644.1   --------------------------------------------------
092 NP_040550.1      --------------------------------------------------
093 NP_758808.1      --------------------------------------------------
094 YP_009345075.1   --------------------------------------------------
095 NP_068729.1      --------------------------------------------------
096 NP_047255.1      --------------------------------------------------
097 YP_006273075.1   --------------------------------------------------
098 YP_009508888.1   --------------------------------------------------
099 NP_395469.1      --------------------------------------------------
100 YP_009508546.1   --------------------------------------------------
101 NP_056762.1      --------------------------------------------------
102 YP_009508556.1   --------------------------------------------------
103 NP_569153.1      --------------------------------------------------
104 NP_043924.1      --------------------------------------------------
105 YP_009243641.1   --------------------------------------------------
106 YP_004300274.1   --------------------------------------------------
107 NP_056848.1      --------------------------------------------------
108 YP_006495799.1   --------------------------------------------------
109 YP_009508409.1   --------------------------------------------------
110 NP_687035.1      --------------------------------------------------
111 NP_045937.2      --------------------------------------------------
112 NP_612577.1      --------------------------------------------------
113 NP_619548.1      --------------------------------------------------
114 NP_056880.1      --------------------------------------------------
115 YP_009121747.1   --------------------------------------------------
116 YP_009113237.2   --------------------------------------------------
117 YP_009508408.1   --------------------------------------------------
118 NP_955579.1      --------------------------------------------------
119 YP_009229919.1   --------------------------------------------------
120 YP_004442833.1   --------------------------------------------------
121 NP_862833.2      --------------------------------------------------
122 NP_040563.1      --------------------------------------------------
123 YP_443922.1      --------------------------------------------------
124 NP_057933.2      --------------------------------------------------
125 YP_009116631.1   --------------------------------------------------
126 YP_009513249.1   --------------------------------------------------
127 YP_223871.1      --------------------------------------------------
128 NP_127504.1      --------------------------------------------------
129 NP_044929.1      --------------------------------------------------
130 NP_057860.1      --------------------------------------------------
131 NP_040973.1      --------------------------------------------------
132 NP_777317.1      --------------------------------------------------
133 NP_054716.1      --------------------------------------------------
134 YP_001856242.1   --------------------------------------------------
135 NP_954565.2      --------------------------------------------------
136 NP_569150.1      --------------------------------------------------
137 NP_659397.1      --------------------------------------------------
138 YP_009513211.1   --------------------------------------------------
139 YP_233107.1      --------------------------------------------------
140 YP_009109692.1   --------------------------------------------------
141 NP_057849.4      --------------------------------------------------
142 NP_049560.1      ALQEQCCFLNITNSHVSILQERPPLENRVLTGWGLNWDLGLSQWAREALQ
143 YP_009506267.1   --------------------------------------------------
144 NP_039820.1      --------------------------------------------------
145 NP_056790.1      --------------------------------------------------
146 YP_002321513.1   --------------------------------------------------
147 NP_955591.1      --------------------------------------------------
148 YP_001956722.2   --------------------------------------------------
149 NP_056902.1      --------------------------------------------------
150 NP_056886.1      --------------------------------------------------
151 NP_041261.1      --------------------------------------------------
```

```
001 YP_004442836.1     ------------------------------------------------
002 NP_056728.1        ------------------------------------------------
003 YP_009508537.1     ------------------------------------------------
004 YP_004732983.2     ------------------------------------------------
005 YP_009508407.1     ------------------------------------------------
006 NP_040939.1        ------------------------------------------------
```

```
007 YP_003864102.1    ----------------------------------------------
008 YP_009002585.1    ----------------------------------------------
009 YP_003284237.1    ----------------------------------------------
010 YP_009268869.1    ----------------------------------------------
011 YP_009506251.1    ----------------------------------------------
012 NP_955611.1       ----------------------------------------------
013 YP_008567619.1    ----------------------------------------------
014 NP_149413.1       ----------------------------------------------
015 YP_004442824.1    ----------------------------------------------
016 YP_001931967.1    ----------------------------------------------
017 YP_002455786.1    ----------------------------------------------
018 YP_009109694.1    ----------------------------------------------
019 NP_789739.1       ----------------------------------------------
020 NP_955619.1       ----------------------------------------------
021 YP_224289.1       ----------------------------------------------
022 YP_009182100.1    WHNIENMMEG------------------------------------
023 NP_705927.1       ----------------------------------------------
024 YP_009508577.1    ----------------------------------------------
025 YP_004442830.1    ----------------------------------------------
026 NP_758887.1       ----------------------------------------------
027 YP_009508406.1    ----------------------------------------------
028 YP_610965.1       ----------------------------------------------
029 YP_001931961.1    ----------------------------------------------
030 YP_241114.1       ----------------------------------------------
031 YP_006732334.1    ----------------------------------------------
032 YP_009041481.1    ----------------------------------------------
033 YP_001036293.1    ----------------------------------------------
034 YP_605811.1       ----------------------------------------------
035 NP_955577.1       ----------------------------------------------
036 YP_003208050.1    ----------------------------------------------
037 NP_789740.1       ----------------------------------------------
038 YP_004442839.1    ----------------------------------------------
039 YP_009507791.1    ----------------------------------------------
040 YP_004581513.1    ----------------------------------------------
041 NP_043933.1       ----------------------------------------------
042 YP_009507248.1    ----------------------------------------------
043 YP_002117531.1    ----------------------------------------------
044 YP_004347415.1    ----------------------------------------------
045 NP_056891.1       ----------------------------------------------
046 YP_009513242.1    ----------------------------------------------
047 YP_009352866.1    ----------------------------------------------
048 YP_009165750.1    ----------------------------------------------
049 NP_040840.1       ----------------------------------------------
050 YP_004222728.1    ----------------------------------------------
051 YP_009506264.1    ----------------------------------------------
052 YP_567050.1       ----------------------------------------------
053 YP_009508571.1    ----------------------------------------------
054 NP_056803.1       ----------------------------------------------
055 NP_955564.1       ----------------------------------------------
056 YP_009508411.1    ----------------------------------------------
057 YP_009140788.1    ----------------------------------------------
058 YP_009508410.1    ----------------------------------------------
059 NP_040333.1       ----------------------------------------------
060 YP_008992013.1    ----------------------------------------------
061 NP_041734.1       ----------------------------------------------
062 NP_056895.1       ----------------------------------------------
063 YP_009109689.1    ----------------------------------------------
064 YP_002519387.1    ----------------------------------------------
065 YP_595725.1       ----------------------------------------------
066 YP_009508551.1    ----------------------------------------------
067 YP_001497148.1    ----------------------------------------------
068 NP_041186.1       ----------------------------------------------
069 NP_663784.1       ----------------------------------------------
070 YP_009508443.1    ----------------------------------------------
071 YP_003987465.1    ----------------------------------------------
072 NP_861410.1       ----------------------------------------------
073 YP_009345071.1    ----------------------------------------------
074 YP_009508582.1    ----------------------------------------------
075 NP_569141.1       ----------------------------------------------
076 YP_002916057.1    ----------------------------------------------
077 NP_777384.2       ----------------------------------------------
078 YP_233110.1       ----------------------------------------------
079 NP_542258.1       ----------------------------------------------
```

```
080 YP_009130664.1   --------------------------------------------
081 YP_009508566.1   --------------------------------------------
082 YP_006607892.1   --------------------------------------------
083 YP_009506270.1   --------------------------------------------
084 NP_042513.1      --------------------------------------------
085 YP_006907834.1   --------------------------------------------
086 YP_004442827.1   --------------------------------------------
087 YP_009408594.1   --------------------------------------------
088 YP_009508561.1   --------------------------------------------
089 NP_056907.1      --------------------------------------------
090 YP_002308474.1   --------------------------------------------
091 YP_007761644.1   --------------------------------------------
092 NP_040550.1      --------------------------------------------
093 NP_758808.1      --------------------------------------------
094 YP_009345075.1   --------------------------------------------
095 NP_068729.1      --------------------------------------------
096 NP_047255.1      --------------------------------------------
097 YP_006273075.1   --------------------------------------------
098 YP_009508888.1   --------------------------------------------
099 NP_395469.1      --------------------------------------------
100 YP_009508546.1   --------------------------------------------
101 NP_056762.1      --------------------------------------------
102 YP_009508556.1   --------------------------------------------
103 NP_569153.1      --------------------------------------------
104 NP_043924.1      --------------------------------------------
105 YP_009243641.1   --------------------------------------------
106 YP_004300274.1   --------------------------------------------
107 NP_056848.1      --------------------------------------------
108 YP_006495799.1   --------------------------------------------
109 YP_009508409.1   --------------------------------------------
110 NP_687035.1      --------------------------------------------
111 NP_045937.2      --------------------------------------------
112 NP_612577.1      --------------------------------------------
113 NP_619548.1      --------------------------------------------
114 NP_056880.1      --------------------------------------------
115 YP_009121747.1   --------------------------------------------
116 YP_009113237.2   --------------------------------------------
117 YP_009508408.1   --------------------------------------------
118 NP_955579.1      --------------------------------------------
119 YP_009229919.1   --------------------------------------------
120 YP_004442833.1   --------------------------------------------
121 NP_862833.2      --------------------------------------------
122 NP_040563.1      --------------------------------------------
123 YP_443922.1      --------------------------------------------
124 NP_057933.2      --------------------------------------------
125 YP_009116631.1   --------------------------------------------
126 YP_009513249.1   --------------------------------------------
127 YP_223871.1      --------------------------------------------
128 NP_127504.1      --------------------------------------------
129 NP_044929.1      --------------------------------------------
130 NP_057860.1      --------------------------------------------
131 NP_040973.1      --------------------------------------------
132 NP_777317.1      --------------------------------------------
133 NP_054716.1      --------------------------------------------
134 YP_001856242.1   --------------------------------------------
135 NP_954565.2      --------------------------------------------
136 NP_569150.1      --------------------------------------------
137 NP_659397.1      --------------------------------------------
138 YP_009513211.1   --------------------------------------------
139 YP_233107.1      --------------------------------------------
140 YP_009109692.1   --------------------------------------------
141 NP_057849.4      --------------------------------------------
142 NP_049560.1      TGITLVALLPLVILAGPCILRQLRHLPSRVRHPHYSLINPESCL
143 YP_009506267.1   --------------------------------------------
144 NP_039820.1      --------------------------------------------
145 NP_056790.1      --------------------------------------------
146 YP_002321513.1   --------------------------------------------
147 NP_955591.1      --------------------------------------------
148 YP_001956722.2   --------------------------------------------
149 NP_056902.1      --------------------------------------------
150 NP_056886.1      --------------------------------------------
151 NP_041261.1      --------------------------------------------
```

1	2	3	4	5	6	7	8	9

Variable Average Conserved

X - Insufficient data - the calculation for this site was performed on less than 10% of the sequences.
