# Supplementary material for: An Alternative HIV-1 Non-Nucleoside Reverse Transcriptase Inhibition Mechanism: Targeting the p51 Subunit
Source: Molecules. 2020 Dec 13;25(24):5902. doi: 10.3390/molecules25245902 (PMC7763519; doi:10.3390/molecules25245902)
Supplement: Supplementary file 1 [file molecules-25-05902-s001.zip › Supplementary Materials/Supplementary Information.docx]

Alternative HIV-1 Non-Nucleoside Reverse Transcriptase Inhibition: Targeting the p51 subunit

Kwok-Fong Chan^a,#^, Chinh Tran-To Su^a,b,#^, Alexander Krah^a^, Ser-Xian Phua^a^, Joshua Yi Yeo^a,b^, Wei-Li Ling^a,b^, Peter J. Bond^a^ and Samuel Ken-En Gan^a,b,c,*^

^a^ Bioinformatics Institute (A*STAR), 30 Biopolis Street, #07-01 Matrix, Singapore 138671

^b^ Experimental Drug Development Centre (A*STAR), 10 Biopolis Road Chromos #05-01, Singapore 138670

^c^ p53 Laboratory (A*STAR), 8A Biomedical Grove, #06-04/05 Neuros/Immunos, Singapore 138648

^#^ Both authors contributed equally to the work

*Corresponding author: [Samuel_Gan@eddc.a-star.edu.sg](mailto:Samuel_Gan@eddc.a-star.edu.sg)

Tel: +65 6407 0584 (S. K-E. G.)

KEYWORDS: HIV, novel p51 drug target, NNRTIs

**SUPPLEMENTARY INFORMATION**


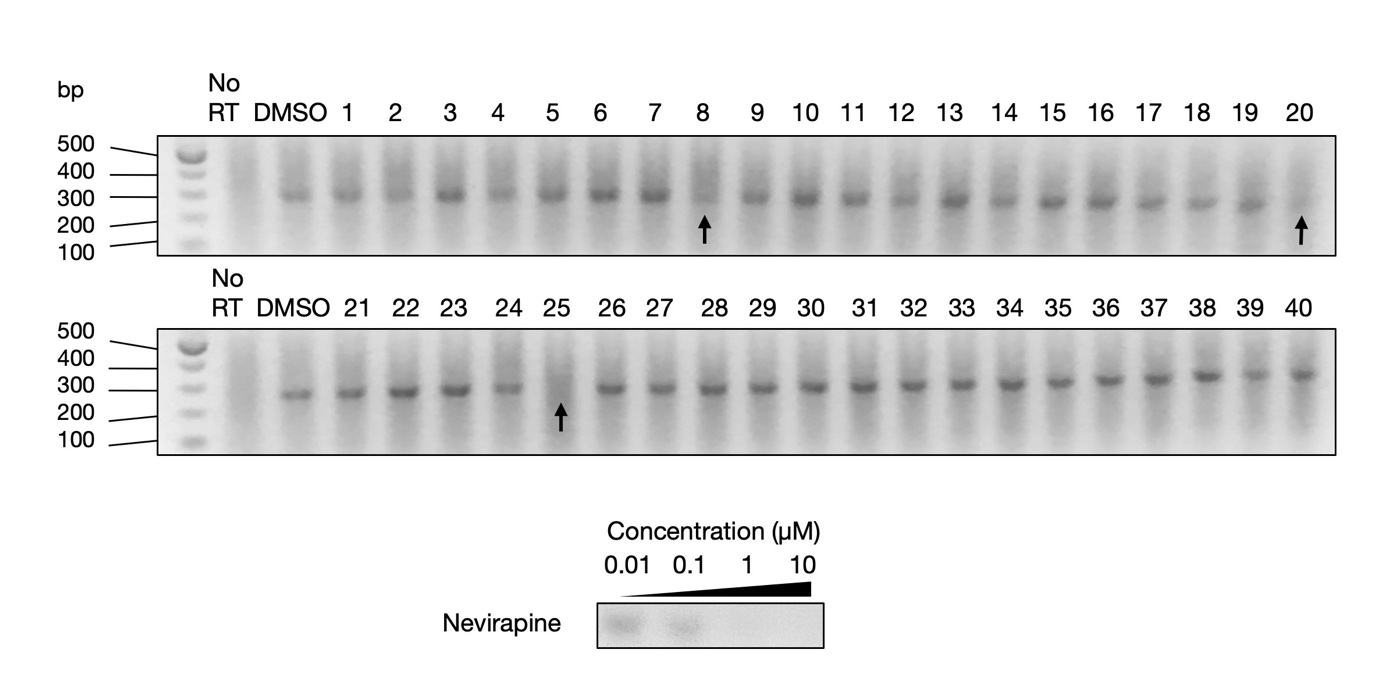


**Supplementary Figure S1**. The agarose gel electrophoresis of RT-PCR GAPDH products treated with 40 compounds from the NCI/DTP Diversity Set V. We found two compounds (labeled 8 and 20, thereafter named as compound 1 and 2, respectively) showed the RT inhibition in all independent triplicates. Note that the inhibition by compound 25 was not reproduced in all the independent triplicates and hence it was excluded in our subsequent analysis.


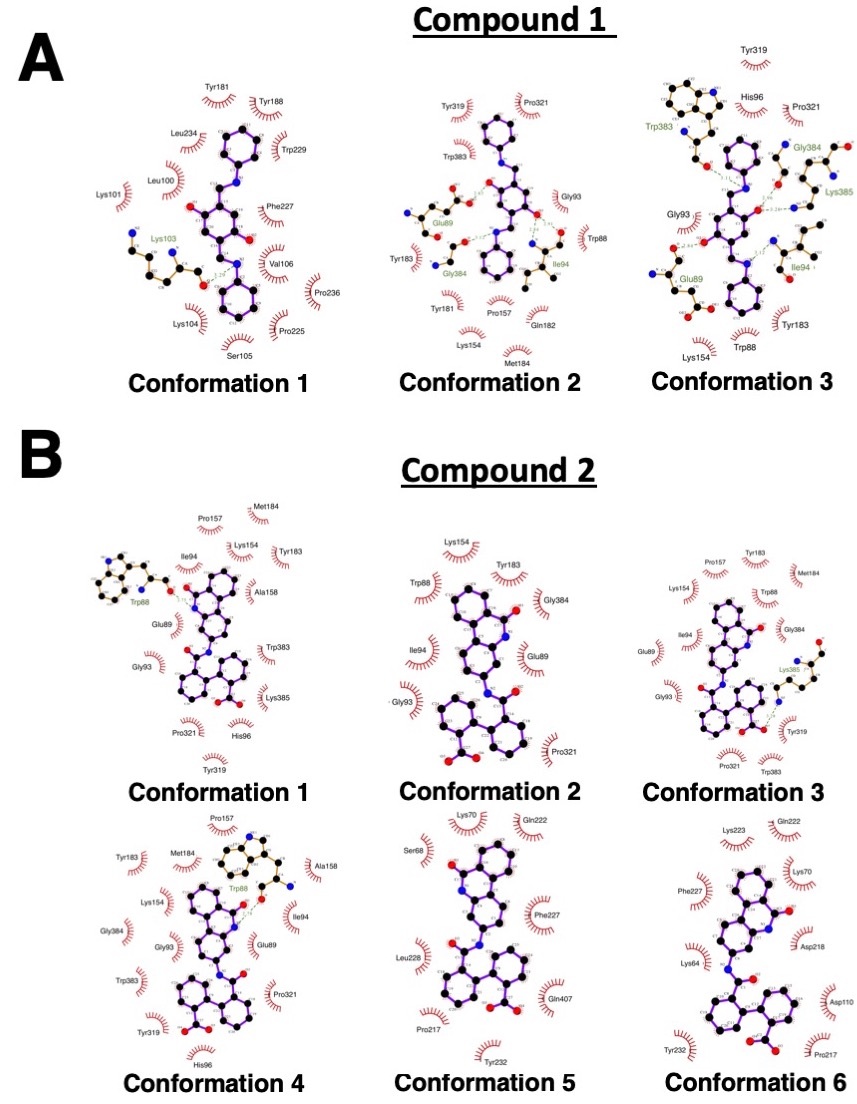


**Supplementary Figure S2**. Initial ligand bound conformations used for different setups of the MD simulations.

**
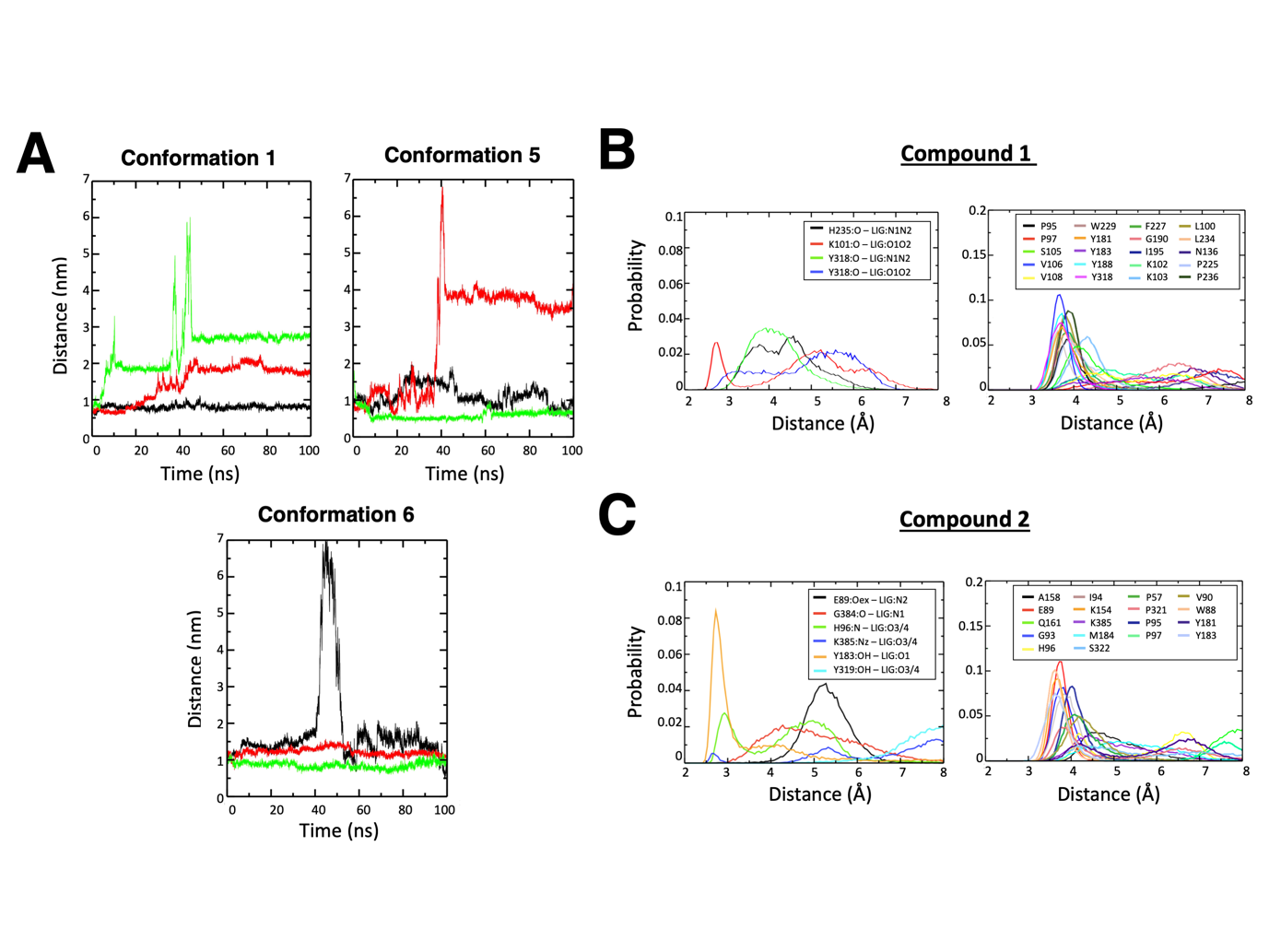
**

**Supplementary Figure S3.** Molecular simulation analyses of the binding sites for compounds 1 and 2. (A) The center-of-mass distances between the binding sites and compound 2 for three initial conformations that exhibited unstable ligand bound positions, calculated using independent triplicates (black, green and red) of 100 ns trajectories. Probability plots of interactions with their respective binding sites are shown for (B) compound 1 and (C) compound 2.

**Supplementary Table S1**. Decomposition of binding energies for compound 1 in the binding sites on RT p66 and p51 subunits. MM-PBSA calculations were carried out with the program *g_mmpbsa* [1] for the last 50 ns of each trajectory, using various internal dielectric constants ε_in_ for the solute [2-4]. Analyses were performed across independent triplicate simulations, using mean and standard deviation.

| Compound 1 | ε_int_ = 2 | | |  | ε_int_ = 8 | | |  | ε_int_ = 20 | | |
| --- | --- | --- | --- | --- | --- | --- | --- | --- | --- | --- | --- |
| (kcal/mol) | Conform. 1 (p66) | Conform. 2 (p51)^#^ | Conform. 3 (p51)^#^ |  | Conform. 1 (p66) | Conform. 2 (p51)^#^ | Conform. 3 (p51)^#^ |  | Conform. 1 (p66) | Conform. 2 (p51)^#^ | Conform. 3 (p51)^#^ |
| Total binding energy | -31.5 ± 3.3 | -16.4 ± 2.3 | -14.2 ± 1.7 |  | -39.3 ± 3.9 | -21.7 ± 2.2 | -19.7 ± 2.5 |  | -43.5 ± 4.1 | -27.8 ± 2.0 | -26.0 ± 2.3 |
| van der Waal | -44.2 ± 3.6 | -33.8 ± 1.9 | -33.1 ± 1.3 |  | -44.2 ± 3.6 | -33.8 ± 1.9 | -33.1 ± 1.3 |  | -44.2 ± 3.6 | -33.8 ± 1.9 | -33.1 ± 1.3 |
| Electrostatics | -3.1 ± 0.9 | -3.8 ± 1.0 | -5.6 ± 1.3 |  | -0.8 ± 0.2 | -1.0 ± 0.2 | -1.4 ± 0.3 |  | -0.3 ± 0.1 | -0.4 ± 0.1 | -0.6 ± 0.1 |
| Polar solvation | 20.7 ± 2.0 | 25.2 ± 3.2 | 28.6 ± 1.9 |  | 10.6 ± 0.8 | 17.0 ± 1.8 | 18.9 ± 1.2 |  | 5.9 ± 0.4 | 10.4 ± 1.0 | 11.7 ± 0.9 |
| Non-polar solvation | -4.9 ± 0.4 | -4.0 ± 0.2 | -4.1 ± 0.2 |  | -4.9 ± 0.4 | -4.0 ± 0.2 | -4.1 ± 0.2 |  | -4.9 ± 0.4 | -4.0 ± 0.2 | -4.1 ± 0.2 |

# In both conformations 2 and 3, compound 1 was found to bind to the same site on the RT p51 subunit.

**Supplementary Table S2**. Additional binding analyses for the two compounds in their binding sites on the RT p66 and p51 subunits. Hydrophobic contacts between protein and ligand were calculated using a 4.0 Å cut-off. Hydrogen bonds between protein and ligand were calculated using a cut-off of 3.5 Å and maximum angle between donor – hydrogen atom and acceptor of 30°. The number of water molecules around the ligand was determined using a cut-off of 3.5 Å. Analyses were performed across independent triplicate simulations, using mean and standard deviation.

| Compound 1 | | | |  | Compound 2 | | | |
| --- | --- | --- | --- | --- | --- | --- | --- | --- |
|  | Number of water molecules | Number of hydrogen bonds | Number of hydrophobic contacts |  |  | Number of water molecules | Number of hydrogen bonds | Number of hydrophobic contacts |
| Conform. 1 (p66) | 2.8 ± 1.5 | 0.6 ±0.6 | 20.4 ± 4.8 |  | Conform. 1 (p51) | 15.2 ± 3.5 | 1.8 ± 1.3 | 13.6 ± 5.1 |
| Conform. 2 (p51) ^a^ | 8.2 ± 2.2 | 1.3 ± 0.8 | 12.2 ± 3.2 |  | Conform. 2 (p51) | 13.3 ± 2.6 | 3.0 ± 1.0 | 16.3 ± 3.6 |
| Conform. 3 (p51) ^a^ | 7.5 ± 2.2 | 1.3 ± 0.8 | 12.2 ± 3.5 |  | Conform. 3 (p51) | 15.4 ± 2.7 | 1.5 ± 1.1 | 15.0 ± 4.0 |
|  |  |  |  |  | Conform. 4 (p51) | 14.1 ± 2.5 | 2.1 ± 1.3 | 15.7 ± 3.6 |
|  |  |  |  |  | Conform. 5 (p51) ^b^ | 15.9 ± 3.6 | 1.7 ± 1.1 | 12.3 ± 5.6 |
|  |  |  |  |  | Conform. 6 (p51) ^b^ | 15.2 ± 3.9 | 2.2 ± 1.2 | 12.8 ± 5.7 |

^a^ In both conformations 2 and 3, compound 1 was found to bind to the same site on the RT p51 subunit.

^b^ Secondary binding site (weaker and unsteady) of compound 2 on the p51 subunit.

**Supplementary Table S3**. Decomposition of binding energies for compound 2 in the binding sites on RT p51 subunits. MM-PBSA calculations were carried out with the program *g_mmpbsa* [1] for the last 50 ns of each trajectory, using various internal dielectric constants ε_in_ for the solute. Analyses were performed across independent triplicate simulations. Only results for the three setups that exhibited stable ligand binding (i.e. conformation 2, 3, and 4) are shown. Analyses were performed across independent triplicate simulations, using mean and standard deviation.

| Compound 2 | ε_int_ = 2 | | |  | ε_int_ = 8 | | |  | ε_int_ = 20 | | |
| --- | --- | --- | --- | --- | --- | --- | --- | --- | --- | --- | --- |
| (kcal/mol) | Conform. 2 (p51) | Conform. 3 (p51) | Conform. 4 (p51) |  | Conform. 2 (p51) | Conform. 3 (p51) | Conform. 4 (p51) |  | Conform. 2 (p51) | Conform. 3 (p51) | Conform. 4 (p51) |
| Total binding energy | -43.7 ± 2.0 | -49.9 ± 3.0 | -45.5 ± 2.2 |  | -13.4 ± 3.6 | -20.1 ± 2.6 | -12.2 ± 5.9 |  | -16.5 ± 2.2 | -17.2 ± 6.3 | -15.1 ± 4.4 |
| van der Waal | -38.7 ± 0.9 | -38.7 ± 0.3 | -38.2 ± 1.1 |  | -38.7 ± 0.9 | -38.7 ± 0.3 | -38.2 ± 1.1 |  | -38.7 ± 0.9 | -38.7 ± 0.3 | -38.2 ± 1.1 |
| Electrostatics | -58.4 ± 8.1 | -57.7 ± 2.8 | -70.3 ± 13.1 |  | -14.0 ± 2.0 | -14.4 ± 0.7 | -17.6 ± 3.3 |  | -5.8 ± 0.8 | -5.8 ± 0.3 | -7.0 ± 1.1 |
| Polar solvation | 57.9 ± 9.9 | 50.9 ± 5.3 | 67.4 ± 13.5 |  | 43.9 ± 5.1 | 37.1 ± 3.6 | 48.0 ± 8.0 |  | 32.5 ± 2.8 | 28.0 ± 2.4 | 34.5 ± 4.6 |
| Non-polar solvation | -4.5 ± 0.1 | -4.1 ± 0.2 | -4.3 ± 0.1 |  | -4.5 ± 0.1 | -4.1 ± 0.2 | -4.3 ± 0.1 |  | -4.5 ± 0.1 | -4.1 ± 0.2 | -4.3 ± 0.1 |

**REFERENCES**

1. Kumari, R.; Kumar, R.; Lynn, A., g_mmpbsa—A GROMACS Tool for High-Throughput MM-PBSA Calculations. Journal of Chemical Information and Modeling 2014, 54, (7), 1951-1962.

2. Genheden, S.; Ryde, U., Comparison of end-point continuum-solvation methods for the calculation of protein–ligand binding free energies. Proteins: Structure, Function, and Bioinformatics 2012, 80, (5), 1326-1342.

3. Yang, T.; Wu, J. C.; Yan, C.; Wang, Y.; Luo, R.; Gonzales, M. B.; Dalby, K. N.; Ren, P., Virtual screening using molecular simulations. Proteins 2011, 79, (6), 1940-1951.

4. Schutz, C. N.; Warshel, A., What are the dielectric “constants” of proteins and how to validate electrostatic models? Proteins: Structure, Function, and Bioinformatics 2001, 44, (4), 400-417.
